# Sphingolipid metabolic enzyme GLA expression in gliomas: prognostic implications and therapeutic potential

**DOI:** 10.3389/fonc.2026.1773193

**Published:** 2026-03-24

**Authors:** Dan Wei, Qiuyang Su, Xiangkun Li, Hongbin Li, Dongyi Jin, Zhixiong Pan, Moujie Yang, Guihong Liu, Yuming Du, Wei Dong

**Affiliations:** 1 Department of Critical Care Medicine, the First Affiliated Hospital of Zhengzhou University, Zhengzhou, Henan, China; 2 Department of Neurology, the First Affiliated Hospital of Zhengzhou University, Zhengzhou, Henan, China; 3 College of Acupuncture and Tuina, Henan University of Chinese Medicine, Zhengzhou, Henan, China; 4 Guangxi Health Commission Key Laboratory of Basic Research in Sphingolipid Metabolism Related Diseases, the Affiliated Hospital of Guilin Medical University, Guilin, Guangxi, China; 5 Department of Hepatobiliary and Pancreatic Surgery, the First Affiliated Hospital of Zhengzhou University, Zhengzhou, Henan, China

**Keywords:** endoplasmic reticulum-associated degradation, GLA, glioma, prognostic biomarker, sphingolipid metabolism

## Abstract

**Background:**

Gliomas are the most common primary brain tumors and show marked molecular heterogeneity and variable clinical outcomes, highlighting the need for reliable prognostic biomarkers.

**Methods:**

We analyzed TCGA and CGGA datasets to identify prognosis-related genes in glioma and performed single-cell analysis and functional validation in glioma cell lines.

**Results:**

Ten prognosis-related genes were identified, among which GLA showed the strongest association with survival. Elevated GLA expression was significantly associated with higher WHO grade, therapy resistance, and worse prognosis. Time-dependent ROC analysis showed favorable predictive performance for 1-, 3-, and 5-year survival. Single-cell RNA sequencing revealed preferential GLA expression in tumor-associated astrocytes and grade-dependent upregulation. GLA expression was associated with sphingolipid metabolism, hypoxia-related pathways, and ERAD-related pathways, including EDEM2 expression. In glioma cell lines, GLA knockdown suppressed cell viability and downregulated EDEM2. Cross-database analyses further supported the diagnostic and prognostic relevance of GLA across cohorts.

**Conclusion:**

GLA may serve as a prognostic biomarker in glioma and is associated with metabolic and ERAD-related alterations, providing a potential direction for further mechanistic and therapeutic investigation.

## Introduction

Gliomas represent approximately 30% of all central nervous system tumors and around 80% of all malignant brain tumors (1, 2). The incidence of gliomas varies by geographical region, with higher rates observed in North America and Europe (3). They are more common in males than in females, with a male-to-female ratio of about 1.5:1 (3, 4). Gliomas, characterized by infiltrative growth and therapeutic resistance, remain a leading cause of cancer-related mortality despite advances in molecular classification (5, 6). Prognostic stratification relies heavily on histological grading, IDH mutation status, and 1p/19q codeletion (7–9), yet interpatient heterogeneity necessitates a deeper exploration of molecular drivers.

Over the past few decades, glioma research has evolved substantially, driven by advancements in molecular biology, genetics, and imaging techniques. Gliomas cells display significant heterogeneity, with variations in gene expression patterns and alterations in key signaling pathways such as the phosphatidylinositol 3-kinase (PI3K) pathway (10, 11), the p53 pathway (12), the epidermal growth factor receptor (EGFR) amplification (13), and lipid metabolism (14).

Sphingolipids are a diverse class of lipids that play crucial roles in various cellular processes, including membrane structure, signaling, and apoptosis. In the context of the central nervous system (CNS), sphingolipid metabolism has gained significant attention due to its involvement in the pathophysiology of neurological disorders, including neurodegenerative diseases and brain tumors (15–17). As a critical component of cellular signaling, sphingolipids influence tumor growth, survival, and metastasis, making them key players in the study of malignant CNS tumors.

Dysregulated glycosphingolipid metabolism has emerged as a hallmark of tumor progression, with enzymes like α-Galactosylceramidase (GLA), a lysosomal hydrolase critical for galactose metabolism, implicated in cancer pathobiology. GLA is a lysosomal enzyme responsible for the hydrolysis of galactosylceramide into ceramide and galactose, playing a crucial role in sphingolipid metabolism (18). GLA refers to α-galactosidase, with its paralog being alpha-n-acetylgalactosaminidase (NAGA). NAGA has been reported in various tumor types, including lung cancer (19), squamous cell carcinoma (20), breast cancer (21), and gastric cancer (22). However, the role and mechanisms of GLA in cancers remain unclear, particularly in CNS tumors.

Thus, the objective of this study is to conduct a comprehensive analysis of the changes in the molecular mechanisms of gliomas, to explore potential treatment targets, to provide new insights for clinical practice, and to strive for improved survival outcomes and quality of life for glioma patients.

## Materials and methods

### Cell lines

SH-SY5Y (human neuroblastoma), U251 (human glioma cell), and U87MG (human glioblastoma cell) from Professor Hanjiang Luo’s lab. SH-SY5Y was maintained in DMEM-F12 (GIBCO, USA) supplemented with 10% fetal bovine serum (FBS), and 1% penicillin-streptomycin. U251 was maintained in DMEM (GIBCO, USA) supplemented with 10% fetal bovine serum (FBS), and 1% penicillin-streptomycin. U-87MG was maintained in MEM (GIBCO, USA) supplemented with non-essential amino acids, 10% fetal bovine serum (FBS), and 1% penicillin-streptomycin.

### Reagents

The primary antibodies used for western blotting included: GLA (15428-1-AP, Proteintech; FNab00330, Fine), GAPDH (AF0006, Beyotime), EDEM1 (26226-1-AP, Proteintech, China), EDEM2 (A17181, Abclonal; H00055741, Thermo Fisher), EDEM3 (27310-1-AP, Proteintech), HRD1 (YN5764, Immunoway), and DERL1 (YN3021, Immunoway). The goat anti-mouse IgG-HRP and goat anti-rabbit IgG-HRP antibodies were purchased from Ray Antibody Biotechnologies. Further details of materials are provided in the **Supplementary Methods**.

### Western blotting

Total protein was extracted from cultured cells using RIPA buffer (Solarbio, China) supplemented with protease inhibitors. The lysates were boiled, resolved on 10% SDS-PAGE gels, and transferred to PVDF membranes. Membranes were blocked in TBST containing 5% non-fat milk for 1 h at room temperature and incubated with primary antibodies overnight at 4 °C. After washing, membranes were incubated with HRP-conjugated secondary antibodies for 1 h at room temperature, developed using ECL reagents (Yeasen, China), and imaged with a Tanon chemiluminescence system (Tanon, China).

### Immunohistochemistry

Following the processes of drying, dewaxing, and rehydration, sections of tumor tissue embedded in paraffin were subjected to high-pressure antigen retrieval using citrate buffer (0.01 M, pH 6.0). Subsequently, the sections were treated with a 0.3% hydrogen peroxide solution for 10 minutes to inhibit endogenous peroxidase activity. Afterward, the sections were blocked with PBS-Tween containing 5% BSA for one hour and then incubated overnight at 4°C with antibodies at a dilution of 1:100. After this incubation, a secondary antibody was applied for one hour, followed by staining with 3,3’-diaminobenzidine tetrahydrochloride (DAB) and hematoxylin. Finally, the sections underwent dehydration and clearing and were mounted with coverslips, ready for examination under a microscope.

### Cell viability assay

Cell viability was assessed using the Cell Counting Kit-8 (MCE, China). Briefly, U251 and U87MG cells were seeded into 96-well plates at 2–5 × 10^3^ cells/well in 100 μL complete medium and allowed to adhere overnight. Cells were transfected with siRNA targeting GLA (siGLA) or negative control siRNA (siNC) according to the transfection protocol described above. At the indicated time points, 10 μL of CCK-8 reagent was added to each well and incubated for 1–2 h at 37°C. Absorbance was measured at 450 nm using a microplate reader.

### Glioma datasets

We downloaded and processed RNA-seq data by the STAR pipeline from the The Cancer Genome Atlas (TCGA)- Glioblastoma Multiforme (GBM) and TCGA- Low Grade Glioma(LGG) projects, and extracted TPM-format data along with clinical data from the TCGA database (https://portal.gdc.cancer.gov). **Supplementary Data**, including WHO grade, IDH mutation status, MGMT, and 1p/19q codeletion, were obtained from the study by Ceccarelli et al. (23). The prognostic data are derived from a Cell article (24). The data filtering strategy involves the removal of normal samples and the exclusion of samples lacking clinical information. The data processing method applied is log2(value + 1). Data is provided within the manuscript or supplementary information files. We also downloaded publicly available transcriptomic databases for gliomas from Clinical Proteomic Tumor Analysis Consortium (CPTAC), Human Protein Atlas (HPA), GEO, and Chinese Glioma Genome Atlas (CGGA). The CGGA database was already processed and ready to use for the downstream analysis.

### Survival analysis and statistical test

Kaplan-Meier survival curves and univariate Cox regression were performed using the R package. To examine the statistical significance between the groups, we either used the chi-square test for the 2×2 matrix or Fisher’s exact test for other combinations. For survival stratification, patients were dichotomized into GLA-high and GLA-low groups using the cohort-specific median GLA expression as the cutoff.

### Single-cell transcriptome data analysis

The single-cell data were sourced from the CGGA dataset and GSE103224. Using the Matrix package (version 1.7.0), the single-cell expression profile data were converted into a sparse matrix and subsequently transformed into a Seurat object using the Seurat package (version 5.1.0). The data was normalized, and 2,000 features were selected as highly variable genes for PCA dimensionality reduction. The Harmony package (version 1.2.1) was employed to remove batch effects from the data, followed by the selection of 20 dimensions for nonlinear UMAP dimensionality reduction and cell clustering. Cell annotation was then performed using the singleR package (version 2.6.0). To evaluate the robustness of key single-cell observations, we conducted a repeated downsampling analysis by randomly subsampling 70% of cells and re-running the core pipeline for 100 iterations; summary outputs from these iterations are provided as **Supplementary Material**.

### Cell communication analysis

CellChat (version 2.1.2) was utilized to perform cell communication analysis on the single-cell data annotated by CGGA as described above. After filtering out subpopulations with fewer than 10 cells, intercellular communication networks were constructed, and the quantity and probability intensity of cell communication were calculated. Group-wise comparisons of cell communication were conducted between the tumor group and the normal group.

### Coimmunoprecipitation

Cells from both the control and treatment groups underwent lysis using an appropriate cell lysis buffer designed for Western blotting and immunoprecipitation (P0013, Beyotime) and were allowed to homogenize on ice for 10 minutes. Subsequently, the samples were centrifuged at 13,400×g for 10 minutes at a temperature of 4°C. The collected supernatants were then incubated overnight at 4°C with 8 μg of a rabbit antibody or rabbit IgG to generate immune complexes. A total of 40 μL of protein A/G-magnetic beads was washed three times using the cell lysis buffer for Western blotting and IP, added to the aforementioned immunocomplexes, and rotated for 8 hours at 4°C. Following this incubation, the magnetic beads were isolated using a magnetic separator and rinsed five times on ice with prechilled cell lysis buffer. The resulting pellet was resuspended in 40 μL of 2×SDS loading buffer, thoroughly mixed by vortexing, and boiled at 100°C for 5 minutes. Finally, the supernatants were collected and utilized as samples for target protein detection via Western blotting.

### Transwell assays for migration and invasion

Cell migration and invasion were assessed using transwell chambers with 8.0-μm pore polycarbonate membranes (Corning, USA). U251 and U87MG cells were transfected with siRNA targeting GLA (siGLA) or negative control siRNA (siNC) as described above.

For the migration assay, inserts were used without Matrigel. A total of 2 × 10^4^ cells in 200 μL serum-free medium were seeded into the upper chamber, and 600 μL complete medium containing 10% FBS was added to the lower chamber as a chemoattractant. After incubation for 24 h at 37°C, non-migrated cells on the upper surface were removed with a cotton swab. Migrated cells on the lower surface were fixed with 4% paraformaldehyde for 15 min and stained with 0.1% crystal violet for 15 min.

For the invasion assay, the upper membrane was pre-coated with Matrigel diluted in serum-free medium and allowed to polymerize at 37 °C for 30 min. A total of 5 × 10^4^ cells were seeded into the Matrigel-coated upper chamber in serum-free medium, and the lower chamber contained complete medium with 10% FBS. After incubation for 48 h, invaded cells were fixed and stained as described above.

Cells were imaged under a light microscope, and migration and invasion were quantified by counting cells in five random fields per insert. All experiments were performed with at least three independent biological replicates. Data are presented as mean ± SD, and statistical comparisons between two groups were performed using a two-tailed Student’s t-test. P < 0.05 was considered statistically significant.

### Statistical analysis

Analyses were performed using GraphPad Prism 8 (GraphPad Inc, USA). Student’s t test or analysis of variance (ANOVA) was used to analyze the statistical significance of the differences. The proportional hazards (PH) assumption was assessed using Schoenfeld residuals. When the PH assumption was violated for a covariate, we applied weighted Cox regression to estimate an average hazard ratio under non-proportional hazards. For correlation and pathway analyses, P values were adjusted for multiple testing using the Benjamini–Hochberg false discovery rate (FDR) procedure. The evaluated significance levels were denoted by *** when p values < 0.001; by ** when p values < 0.01; or by * when p values < 0.05.

## Results

### The screening of differential genes related to prognosis in gliomas

**Figure 1:**
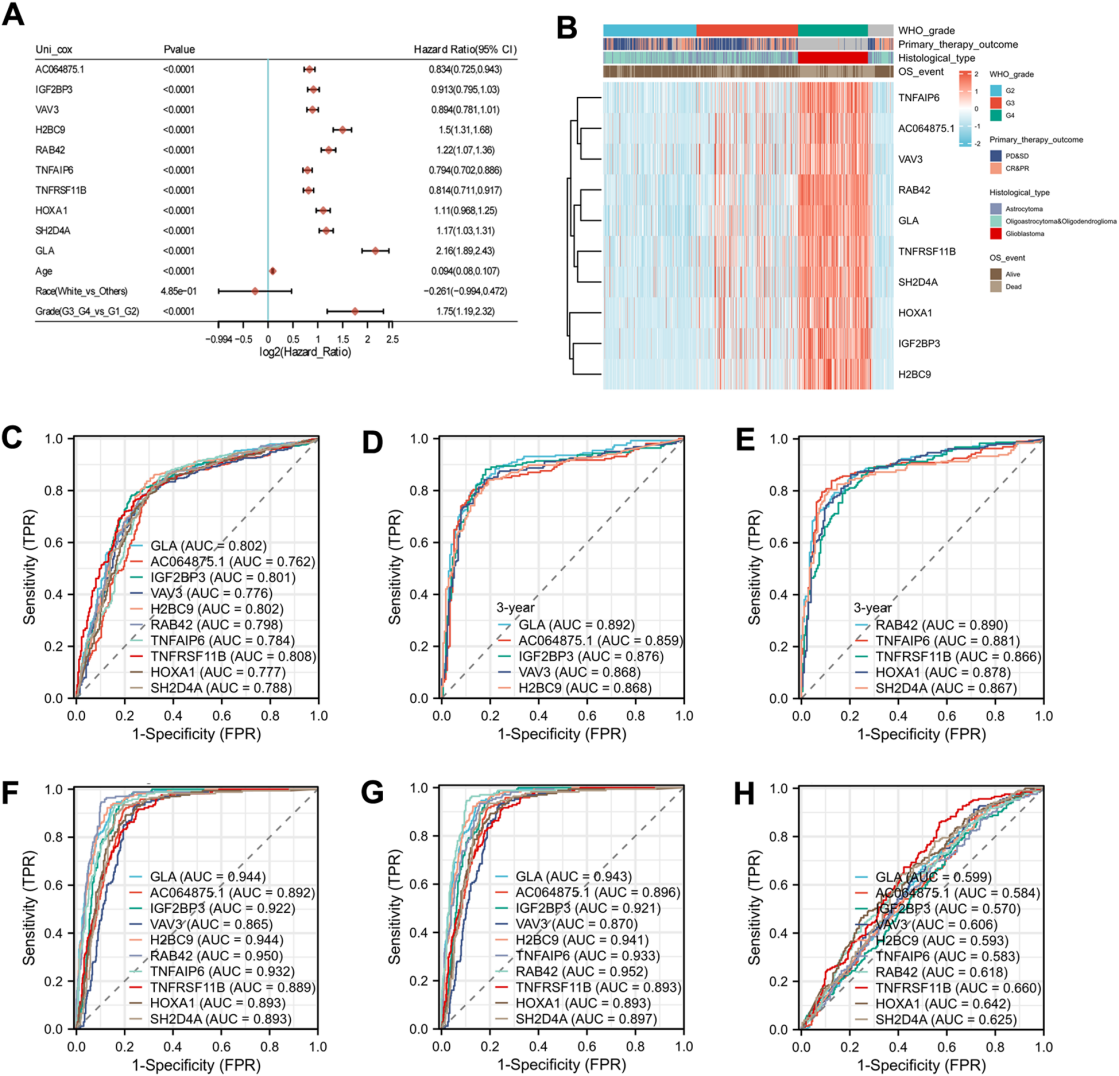
Screening of differential genes associated with prognosis in glioma. **(A)** The forest plot depicting the 10 genes most significantly associated with glioma prognosis. **(B)** The heatmap illustrating the 10 genes most significantly associated with glioma prognosis alongside various clinical indicators. **(C-H)** Among the 10 prognostic genes, the time-dependent ROC analysis related to survival outcomes **(C)**, the 3-year survival rate **(D, E)**, WHO grade **(F)**, histological type **(G)**, and treatment outcomes **(H)** for the 10 prognostic genes.

To investigate the genes associated with glioma prognosis, we initially analyzed the differential genes related to the prognosis of glioma patients using the TCGA database. We identified ten prognostic genes, including AC064875.1, IGF2BP3, VAV3, H2BC9, RAB42, TNFAIP6, TNFRSF11B, HOXA1, SH2D4A, and GLA. An univariate Cox analysis revealed that GLA exhibited the strongest correlation with the prognosis of glioma patients (**Figure 1A**, **Supplementary Table 1**). Furthermore, we analyzed the expressions of these ten genes across various groups categorized by WHO grade, primary therapy outcome, histological type, and overall survival (OS) event. The majority of these genes exhibited increased expression in groups with higher malignancy and poorer survival prognosis (**Figure 1B**). Additionally, focusing on the OS event, 3-year survival rate, WHO grade, and histological type of glioma patients, time-dependent ROC analysis demonstrated that GLA had the highest AUC for prediction (**Figures 1C–G**). While in the predictive grouping based on treatment outcomes, the diagnostic capability of GLA showed a decline (**Figure 1H**). These results suggest that GLA possesses favorable diagnostic efficacy in assessing the malignancy and prognosis of gliomas.

### The evaluation of the predictive value of GLA for glioma patients’ outcomes

**Figure 2:**
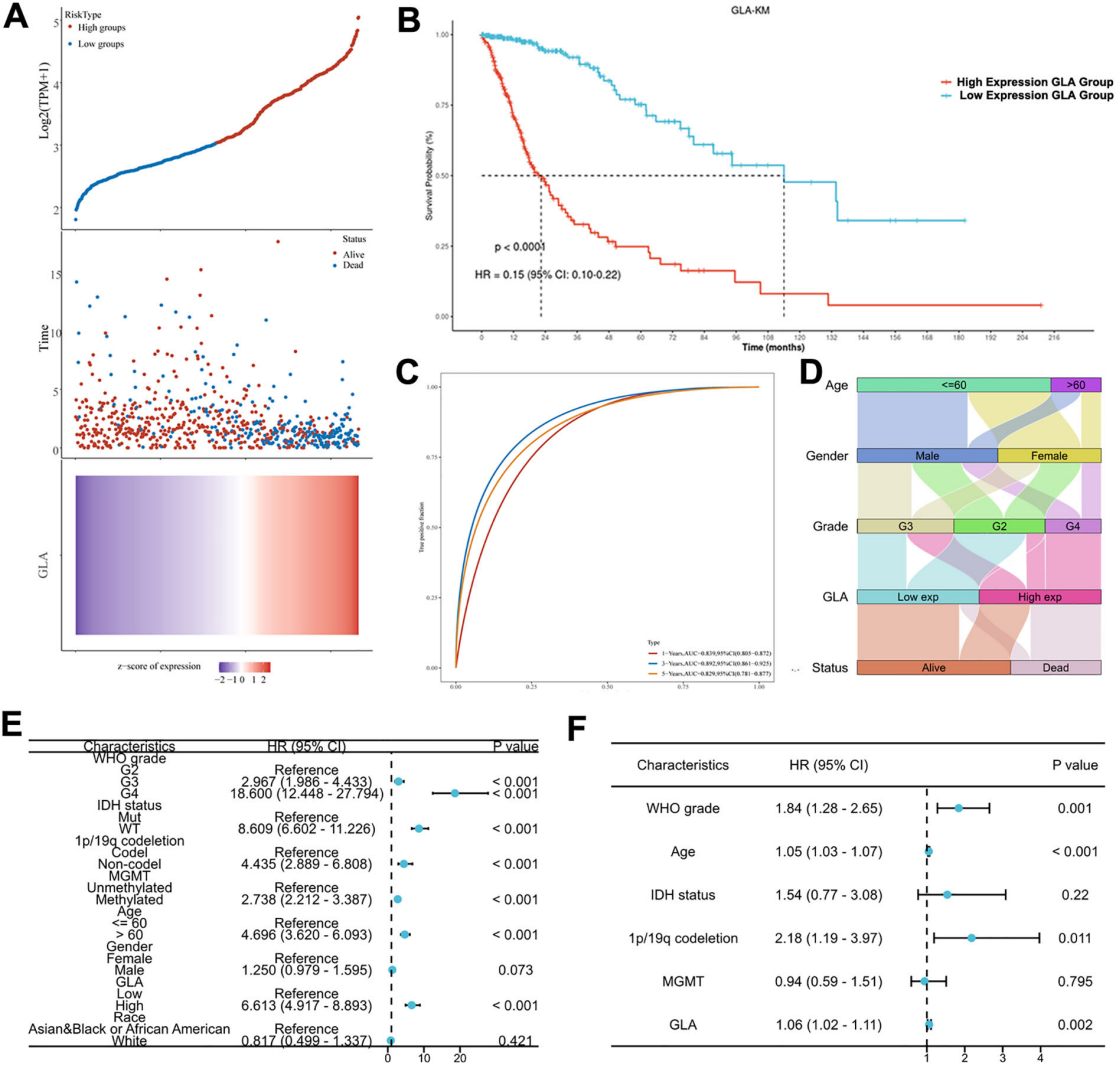
The predictive value of GLA regarding the prognosis of glioma patients. **(A)** The relationship between GLA expression and survival time and status in TCGA data is examined. The top graph presents a scatter plot of gene expression levels ranging from low to high, with distinct colors representing different expression groups. The middle graph illustrates the distribution of survival time and status corresponding to gene expression across various samples. The bottom graph displays a heatmap of gene expression. **(B)** The Kaplan-Meier survival analysis of GLA in TCGA data. **(C)** Time-dependent ROC curves evaluating the predictive performance of GLA expression for 1-, 3-, and 5-year overall survival. **(D)** The Sankey diagram illustrates the relationship between GLA expression and clinical characteristics, where the width of the branches signifies the magnitude of data flow. **(E, F)** The univariate and weighted COX regressions are conducted on several clinical indicators in conjunction with GLA expression.

After establishing the diagnostic value of GLA for gliomas, we examined the association between GLA expression and survival outcomes. Patients were dichotomized into GLA-high and GLA-low groups using the cohort-specific median as the cutoff. High GLA expression was associated with limited overall survival (**Figures 2A, B**), and time-dependent ROC analysis indicated that GLA expression had predictive value for 1-, 3-, and 5-year survival (**Figure 2C**). Consistently, the GLA-high group was enriched for high WHO grade tumors and adverse survival status (**Figure 2D**). We first performed univariate Cox analyses for clinical variables and GLA (**Figure 2E**). We subsequently evaluated the proportional hazards assumption. Because violations of this assumption were observed for IDH status and GLA, we used a weighted Cox regression approach to estimate average hazard ratios (AHRs) in the multivariable model. In the adjusted analysis, WHO grade, age, 1p/19q codeletion, and GLA remained significantly associated with patient outcomes (**Figure 2F**).

### The analysis of prognosis-related genes combined with clinical indicators

**Figure 3:**
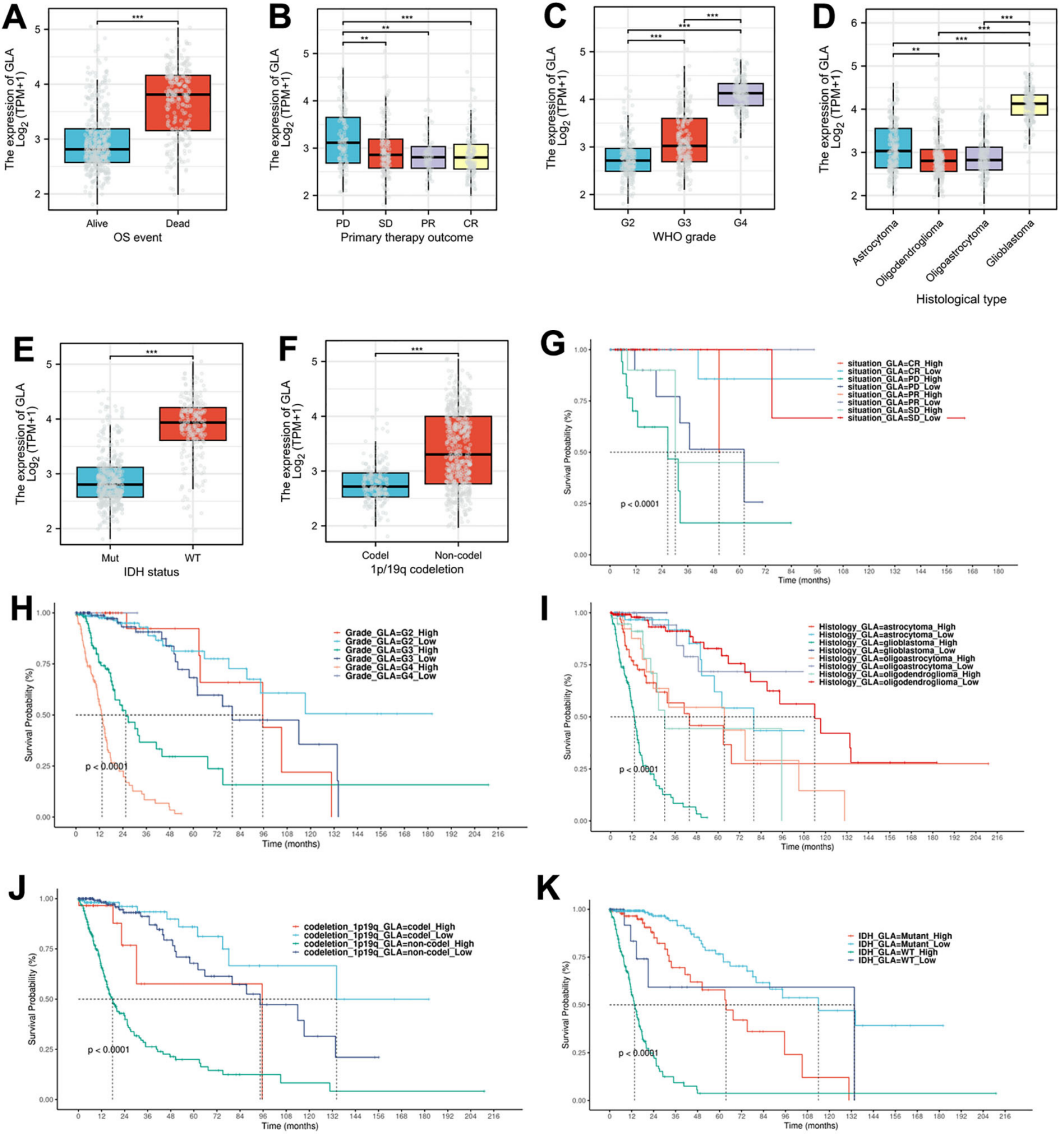
The analysis of prognosis-related genes in conjunction with clinical indicators. **(A-F)** The expression differences of GLA across various groups categorized by OS event **(A)**, primary therapy outcome **(B)**, WHO grade **(C)**, histological type **(D)**, IDH status **(E)**, and 1p/19q codeletion **(F)**. **(G-K)** The Kaplan-Meier survival analysis of GLA expression in therapy outcome **(G)**, WHO grade **(H)**, histological type **(I)**, IDH wild type **(K)**, or 1p/19q non-codeletion **(K)**.

We also analyzed the expression differences of GLA across various groups categorized by WHO grade, primary therapy outcome, histological type, and overall survival (OS) event. The results revealed that, in the OS event, the expression of GLA in the group of dead was significantly higher (p<0.001) than that in the group of alive (**Figure 3A**). In the primary therapy outcome, the expression of GLA in the PD group was significantly higher (p<0.001) than that in the groups of SD, PR and CR (**Figure 3B**). In the WHO grade, the expressions of GLA were significantly higher (p<0.001) than that in the groups of SD, PR and CR (**Figure 3B**). In terms of WHO grade, the expression of GLA progressively increases with higher WHO grades (**Figure 3C**). In terms of histological type, although GLA expression is significantly elevated in glioblastoma patients who exhibit the highest malignancy and poorest prognosis, the GLA expression in astrocytomas is higher than that in oligodendrogliomas and oligoastrocytomas (**Figure 3D**). In terms of IDH status and 1p/19q codeletion, the groups of IDH wild type and non-codeletion of 1p/19q exhibited a significant increase in GLA expression, which correlates with poor treatment efficacy clinically (**Figures 3E, F**). Through Kaplan-Meier survival analysis, we found that the differentiation of GLA expression on survival prognosis is significant for the subgroups of therapy outcome, WHO grade, histological type, IDH wild type, or 1p/19q non-codeletion (**Figures 3G–K**). These results suggest that GLA expression is of value for diagnosing malignancy and assessing prognosis in glioma patients.

### CGGA data validation of GLA expression in glioma

**Figure 4:**
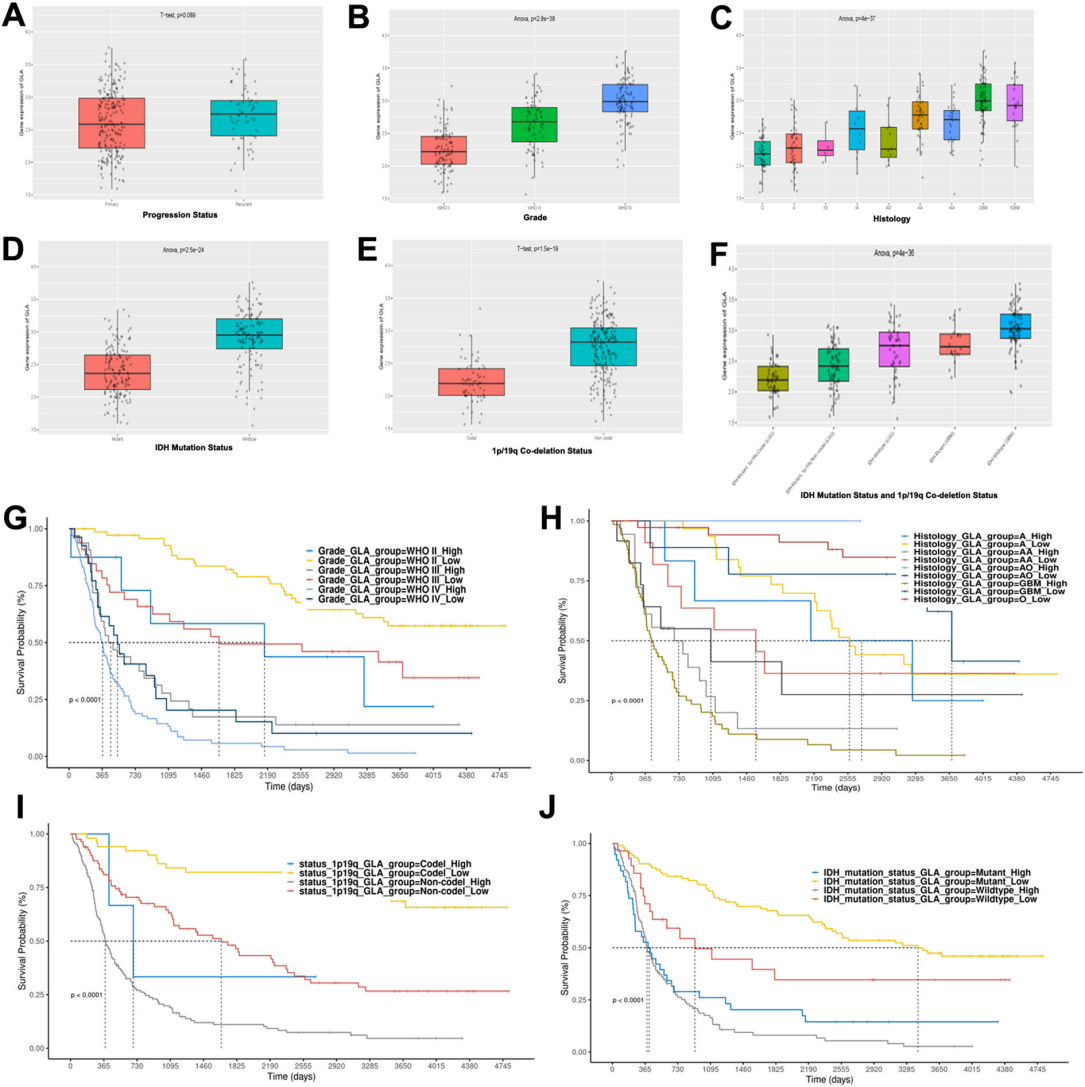
CGGA data validation of GLA expression in glioma. **(A-F)** The expression levels of GLA in the CGGA data among the groups of progression status **(A)**, WHO grade **(B)**, histological type **(C)**, IDH mutation status **(D)**, and 1p/19q co-deletion status **(E, F)**. **(G-J)** The Kaplan-Meier survival analysis of GLA expression among the groups of WHO grade **(G)**, histological type **(H)**, 1p/19q co-deletion **(I)**, and IDH mutation status **(J)**.

Previously, we discovered the diagnostic and prognostic value of GLA for the malignancy of glioma patients in the TCGA database. However, we also noted that in the TCGA database analysis, there was a significant racial disparity, with the vast majority of TCGA cases being white, which may not comprehensively and systematically reflect the glioma situation. Based on this, we additionally analyzed the expression of GLA in the CGGA. The expression levels of GLA in the CGGA exhibited a highly significant correlation with the degree of malignancy (**Figures 4A–F**), particularly with histology (**Figure 4C**) and the combined status of IDH mutation and 1p/19q co-deletion (**Figure 4F**). In the Kaplan-Meier survival analysis, we found that the differentiation of GLA expression on survival prognosis is remarkably significant among the groups of WHO grade, histological type, 1p/19q co-deletion status, and IDH mutation status (**Figures 4G–J**). These results corroborate the phenomena observed in the TCGA database.

### The GLA expression in various glioma cell types

After clarifying the association of GLA expression with clinical characteristics in bulk cohorts, we explored cell-type–associated expression patterns using the CGGA scRNA-seq dataset. A total of 6,148 cells passed quality control and were embedded and clustered, yielding nine annotated cell populations. We acknowledge that 6,148 cells is modest by current scRNA-seq standards; however, this dataset was generated with a multi-region sampling design to capture spatial heterogeneity across many tumor regions from multiple patients, which can reduce per-region cell yield. To address stability concerns, we randomly performed sampling 70% of cells and repeated the core analysis 100 times (**Supplementary Material**). Accordingly, we interpret the single-cell results as descriptive and hypothesis-generating.

**Figure 5:**
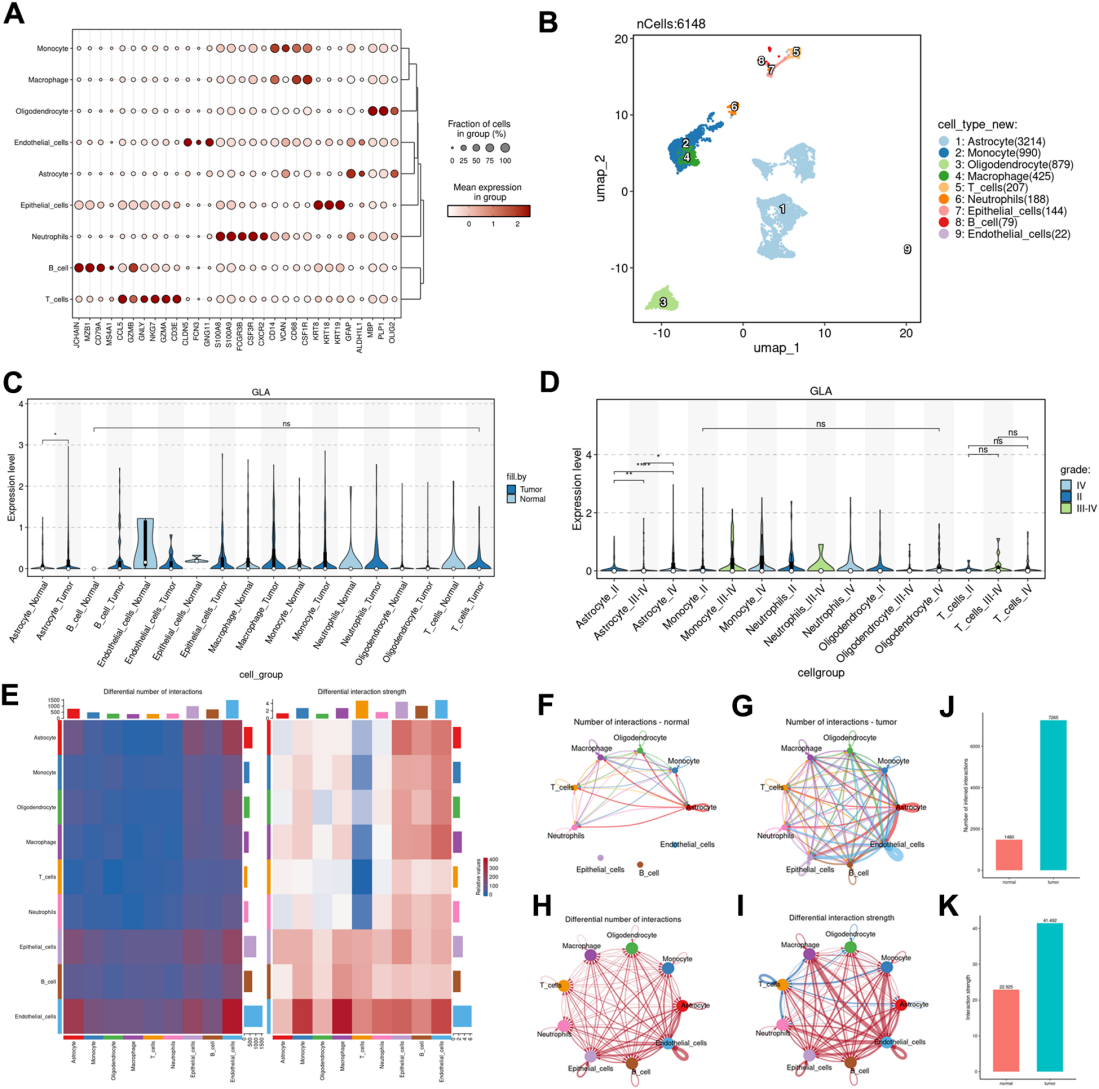
The GLA expression in various glioma cell types. **(A)** The heatmap of marker genes from single-cell RNA sequencing is presented. **(B)** The UMAP plot of the CGGA single-cell data unveils nine distinct cell types. **(C)** The violin plot illustrates the differential expression levels of GLA across various cell subpopulations in both tumor and normal tissues. **(D)** The violin plot illustrates the differential expression levels of GLA across various cell subpopulations in different WHO stages. **(E)** The heatmap illustrates the differential interaction numbers and intensities between various cell types, as derived from CellChat analysis. The vertical axis represents the sender cells, while the horizontal axis denotes the receiver cells. **(F, G)** The chord diagrams derived from CellChat analysis depict the differential interaction quantities among various cell types in tumor tissue **(F)** and normal tissue **(G)**, where the thickness of the chords represents the number of interactions between sender and receiver cells. **(H, I)** Chordal graph (by CellChat analysis) of differential interaction numbers **(H)** and interaction strength **(I)** of each cell type between tumor and normal tissue, where the thickness degree indicates the interaction numbers or interaction strength between sender and receiver cells. **(J, K)** Bar plots showing interaction numbers and strengths in the tumor **(J)** and normal group **(K)**.

As shown by marker-gene annotation (**Figures 5A, B**), these subpopulations included astrocytes (3,214 cells, 52.3%), monocytes (990 cells, 16.1%), oligodendrocytes (879 cells, 14.3%), macrophages (425 cells, 6.9%), T cells (207 cells, 3.4%), neutrophils (188 cells, 3.1%), epithelial cells (144 cells, 2.3%), B cells (79 cells, 1.3%), and endothelial cells (22 cells, 0.4%). Across annotated populations, GLA expression showed cell type-associated heterogeneity, with relatively higher expression in the astrocyte-like compartment compared with most immune and vascular populations. When stratifying by tissue status, the overall difference in GLA expression was modest, with a more apparent tumor-associated increase within astrocyte-like cells (**Figure 5C**). When grouped by WHO grade, astrocyte-like cells exhibited a grade-associated trend (**Figure 5D**). Given the limited cell number and uneven representation of certain populations, these observations should not be interpreted as definitive cell-type–specific drivers of malignant progression. Finally, CellChat analysis suggested increased and rewired intercellular communication patterns in tumor tissue compared with normal tissue (**Figures 5E–K**). We additionally validated the direction of GLA-associated signals using an independent scRNA-seq dataset (GSE103224) and multiple GEO cohorts (GSE12657, GSE43378, and GSE45921) (**Supplementary Figure 1**).

### Analysis of GLA-related pathways

**Figure 6:**
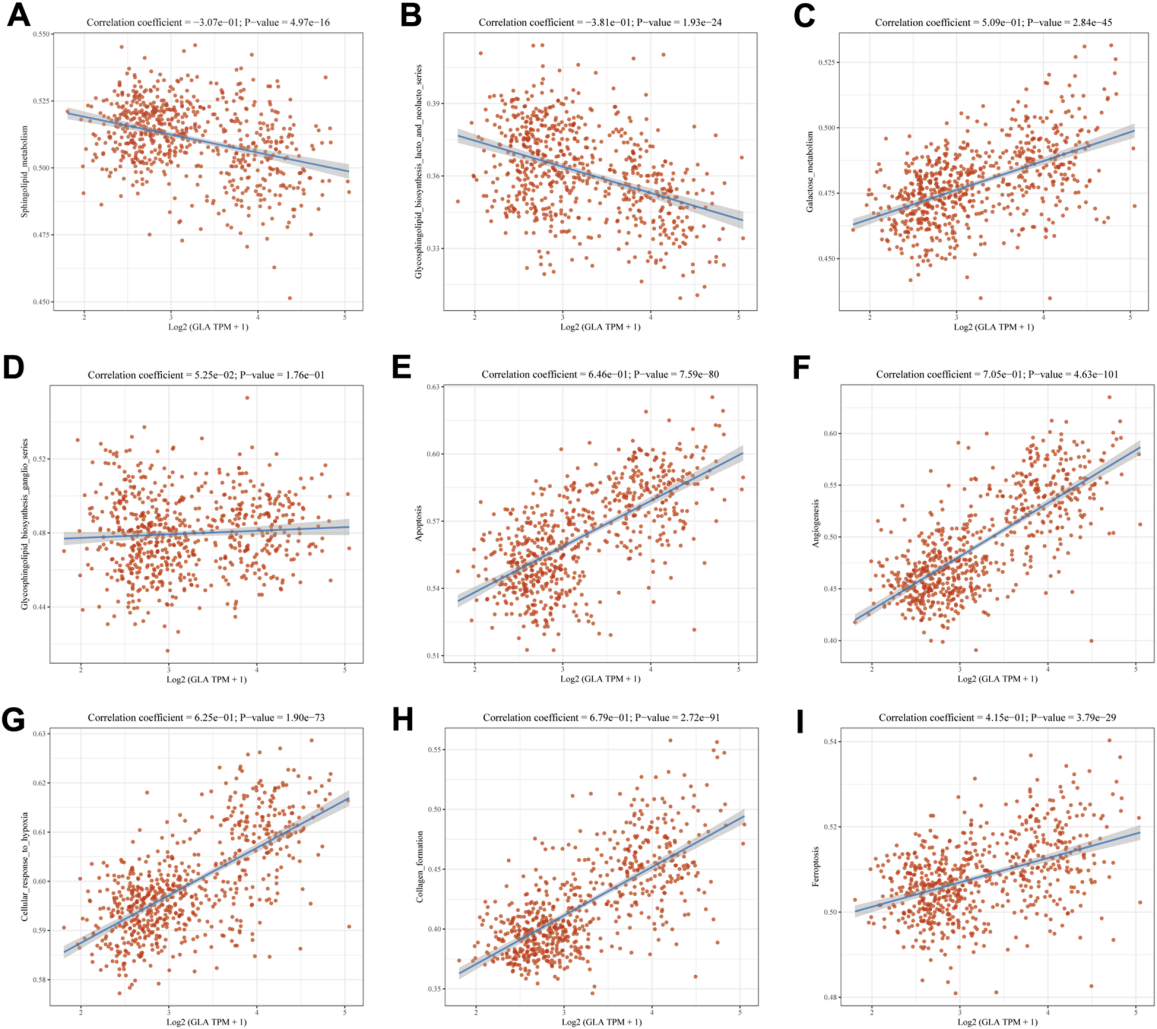
Analysis of GLA-related pathways. The scatter plots illustrating the correlation analysis of GLA with sphingolipid metabolism **(A)**, glycosphingolipid biosynthesis **(B)**, galactose metabolism **(C)**. ganglio series glycosphingolipid biosynthesis **(D)**, apoptosis **(E)**, angiogenesis **(F)**, cellular responses to hypoxia **(G)**, collagen formation **(H)**, and ferroptosis **(I)**.

It is well known that GLA hydrolyzes ceramide trihexoside (Gb3), contributing to glycosphingolipid catabolism. Consequently, we examined correlations between GLA and sphingolipid-related pathways. GLA was negatively correlated with sphingolipid metabolism and glycosphingolipid biosynthesis (lacto and neolacto series) (**Figures 6A, B**), while being positively correlated with galactose metabolism and showing no clear association with ganglio-series glycosphingolipid biosynthesis (**Figures 6C, D**). We further evaluated pathways related to glioma progression and observed that higher GLA expression was associated with signatures linked to apoptosis, angiogenesis, cellular responses to hypoxia, collagen formation, and ferroptosis (**Figures 6E–I**). P values for these correlation/pathway analyses were adjusted using the Benjamini–Hochberg FDR procedure.

### Exploration of the specific mechanism of GLA regulating glioma cell proliferation and apoptosis

**Figure 7:**
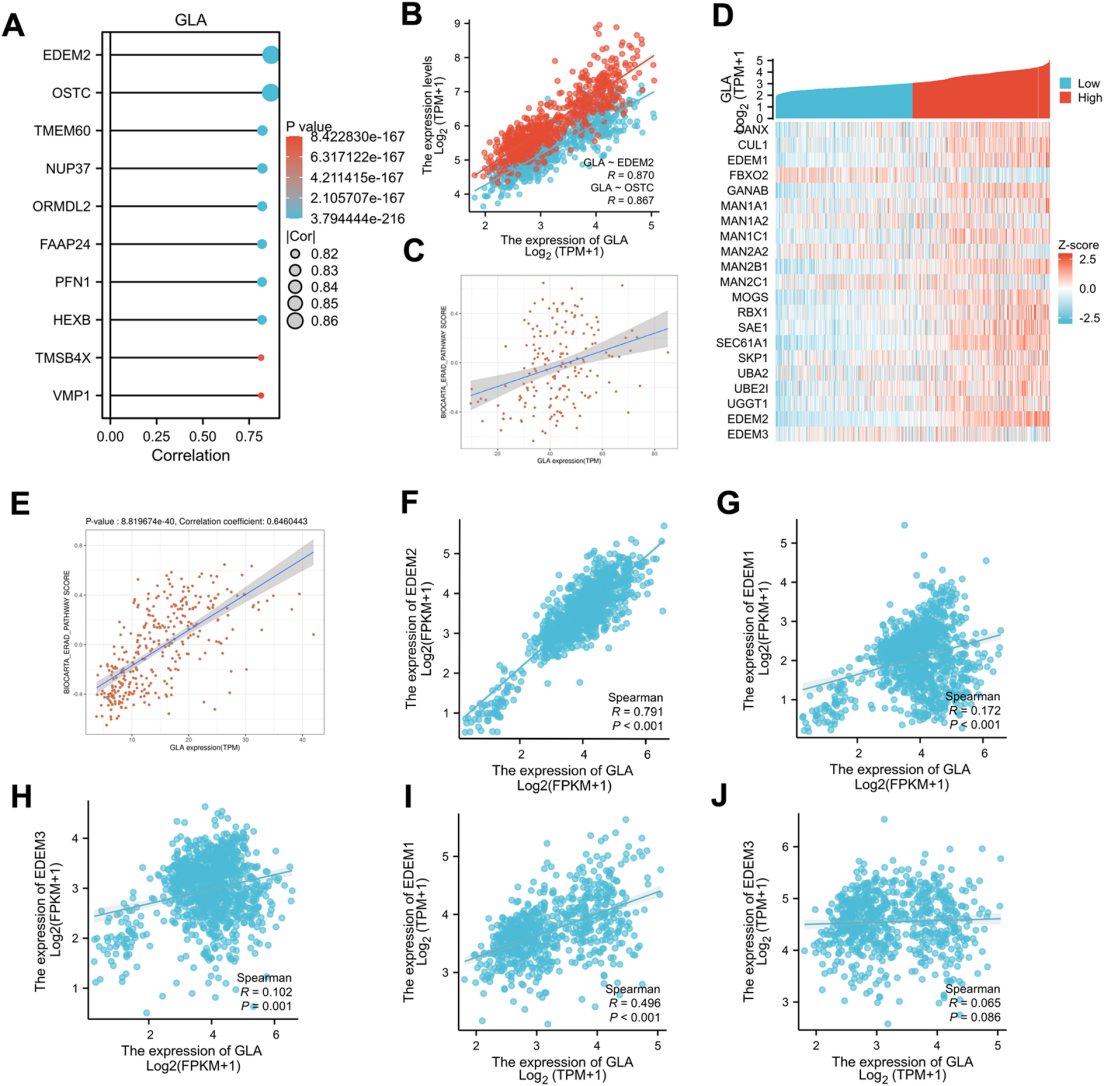
Exploration of the specific mechanism of GLA regulating glioma cell proliferation and apoptosis. **(A)** Lollipop plots depicting the ten genes most closely associated with GLA expression are presented. **(B)** The scatter plots illustrate the correlation analysis of GLA with EDEM2 and OSTC in TCGA. **(C)** The scatter plots demonstrate the correlation analysis of GLA with the ERAD pathway in TCGA. **(D)** The heatmap shows the co-expression of GLA with ERAD pathway-related genes in TCGA. **(E)** The scatter plots illustrate the correlation analysis of GLA with the ERAD pathway. **(F-H)** The scatter plots illustrate the correlation analysis of GLA with EDEM2 **(F)**, EDEM1 **(G)**, and EDEM3 **(H)** in CGGA. Expression values in CGGA are presented as transcripts per million (TPM). **(I-J)** The scatter plots illustrate the correlation analysis of GLA with EDEM1 **(I)** and EDEM3 **(J)** in CGGA. Expression values in TCGA are presented as FPKM (fragments per kilobase of transcript per million fragments).

We analyzed the molecular mechanisms by which GLA regulates gliomas. Initially, through correlation analysis, we found that EDEM2 and OSTC exhibited the highest correlation with GLA, with Spearman coefficients exceeding 0.85 (**Figures 7A, B**). Given that EDEM2 is a key protein in endoplasmic reticulum-associated degradation (ERAD), we explored the relationship between the ERAD pathway and GLA, and discovered a strong correlation between the two (**Figure 7C**). Based on this finding, we conducted a co-expression analysis of ERAD-related genes with GLA and discovered that among the EDEM family members, EDEM2 demonstrated the highest co-expression correlation with GLA (**Figure 7D**). Additionally, we validated the correlation of GLA with the ERAD pathway in the CGGA dataset (**Figure 7E**). We validated the expression of ERAD, EDEM1, EDEM2, EDEM3, and GLA in the CGGA dataset (**Figures 7F–J**). These results indicate that GLA is closely related to EDEM2, suggesting that EDEM2 may serve as a regulatory target of GLA.

### The knockdown of GLA inhibits the proliferation of glioma cells

**Figure 8:**
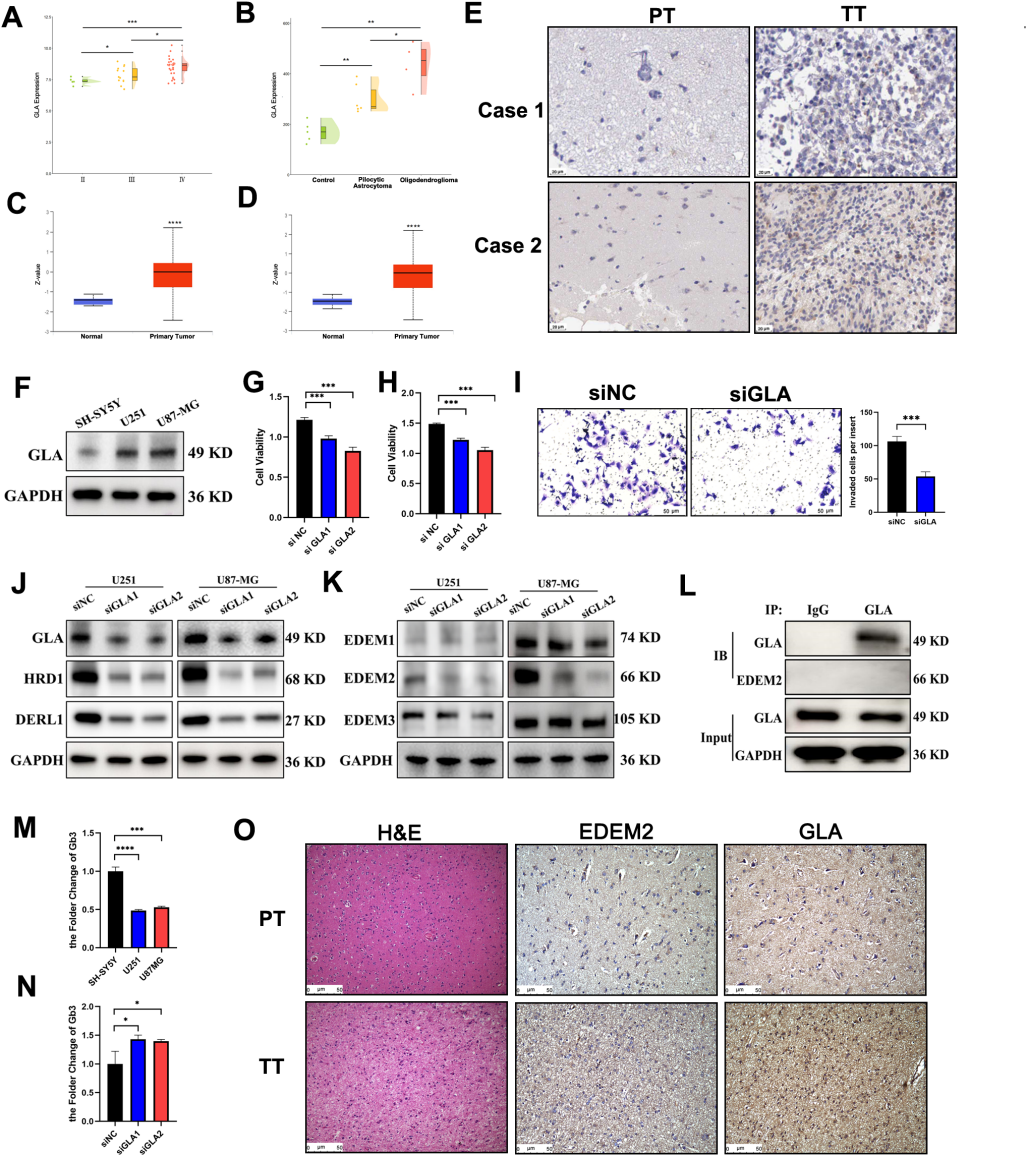
The knockdown of GLA inhibits the proliferation of glioma cells. **(A, B)** The mRNA expression of GLA among GSE43378 **(A)** and GSE12657 **(B)**. **(C, D)** The protein expression of GLA among two cohorts of CPTAC, GBM **(C)** and GBM-extended **(D)**. **(E)** Representative images of IHC staining for GLA in glioma patients from the HPA database. Scale bar: 20 μm. **(F)** The results of Western blotting regarding GLA expression in various nervous system tumor cell lines are presented. **(G, H)** The impact of GLA knockdown on cell viability was assessed in U251 **(G)** and U87-MG **(H)**. **(I)** The impact of GLA knockdown on cell invasion was assessed in U251 cells. **(J)** The expressions of GLA and ERAD-related markers in the GLA knockdown groups were validated in both the U251 and U87-MG cell lines. **(K)** The effects of GLA knockdown on the EDEM family were evaluated by western blotting. **(L)** The co-IP results of GLA and EDEM2 are presented. **(M)** The changes of Gb3 in various nervous system tumor cell lines. **(N)** The changes in Gb3 following the knockdown of GLA are shown by ELISA. **(O)** Representative images of H&E and IHC staining for GLA and EDEM2 in glioma patients. Scale bar: 50 μm. **(A, B)** one-way ANOVA. **(C, D, G–I, M, N)** Student's t test. *p < 0.05, **p < 0.01, ***p < 0.001. PT, peritumoral tissue; TT, tumor tissue.

Finally, analysis of multiple GSE datasets consistently demonstrated that GLA mRNA is upregulated in glioma and shows a stepwise increase with increasing malignancy grade (**Figures 8A, B**, **Supplementary Figure 1C**). Moreover, the validation of protein expression using two cohorts of CPTAC dataset, GBM and GBM-extended, further supported higher GLA expression in glioma samples (**Figures 8C, D**). This phenomenon was also confirmed by IHC. This was also validated by IHC from HPA database (**Figure 8E**). GLA expression across various nervous system cancer cell lines was observed at significantly elevated levels in U251 and U87MG cells (**Figure 8F**). The efficiency of GLA knockdown using siRNA was confirmed by Western blotting (**Figure 8J**), and this knockdown significantly reduced cell viability, migration, and invasion (**Figures 8G–I**, **Supplementary Figure 2**).

Following GLA knockdown, key ERAD-related markers HRD1 and DERL1 decreased (**Figure 8J**). Among EDEM family members, EDEM2 showed a selective reduction, whereas EDEM1 and EDEM3 changed minimally (**Figure 8K**). We did not detect a stable physical interaction between GLA and EDEM2 via co-IP assay (**Figure 8L**), supporting an indirect relationship rather than direct binding.

Given the role of GLA in glycosphingolipid metabolism, we quantified Gb3 and observed that compared with SH-SY5Y, Gb3 significantly decreased in U251 and U87-MG cells (**Figure 8M**), and GLA silencing led to the accumulation of Gb3 (**Figure 8N**). Finally, IHC staining confirmed higher expression of both GLA and EDEM2 in tumor tissue compared with peritumoral tissue (**Figure 8O**). Together, these results support an association between GLA perturbation, glycosphingolipid homeostasis, and ERAD-related changes, while the causal intermediate steps require further study.

## Discussion

The present study systematically elucidates the prognostic significance, functional mechanisms, and clinical implications of GLA in gliomas, integrating multi-omics data and experimental validation. Our findings position GLA as a robust biomarker associated with aggressive phenotypes, therapy resistance, and poor survival outcomes. The intersection of sphingolipid metabolism, ERAD pathways, and glioma progression further highlights its mechanistic relevance, offering novel insights into therapeutic targeting.

GLA’s prognostic value was rigorously validated across TCGA and CGGA cohorts, transcending ethnic disparities in glioma biology. Its elevated expression in higher WHO grades, therapy-resistant subgroups, and deceased patients underscores its role as a surrogate marker of malignancy. The time-dependent ROC analysis highlights GLA’s superior accuracy in predicting short- and long-term survival, outperforming conventional markers like IDH status or 1p/19q codeletion in multivariate models. This suggests that GLA captures distinct molecular features of gliomas that complement existing stratification systems. Numerous studies have reported alterations in the metabolism of oligosaccharide chains within tumor tissue (25–27). Research indicated that the expression and enzymatic activity of β-hexosaminidase and its isoenzymes A and B, β-galactosidase, and α-mannosidase were significantly elevated in gliomas (28). Furthermore, lysosomal β-galactocerebrosidase (GALC) removes β-galactose from β-galactocerebroside. Recent observations suggest that GALC may exert opposing effects on tumor growth and differentiation, acting either as an oncogenic enzyme or as an anti-oncogenic enzyme (29) and raising questions about its contribution to sphingolipid metabolism in cancer cells and its role in tumor progression (30). This duality appears to depend on various factors, including the experimental approach, the stage of research, and the specific types of tumors being studied. However, it is crucial to note that β-galactosidase and α-galactosidase are not paralogs. The hydrolytic substrate of α-Galactosylceramidase is Gb3, whereas GALC primarily hydrolyzes a broader range of substrates (31), including galactocerebroside, galactosylsphingosine, lactosylceramide, and monogalactosyl diglyceride. GLA refers to α-galactosidase, with its paralog being NAGA. The mechanisms through which GLA and NAGA function in gliomas remain unclear. Although it was previously thought that glycosphingolipids influence tumor invasion, migration, and immune infiltration, our bioinformatics analysis has revealed that in gliomas, GLA expression is primarily concentrated in astrocytes themselves, rather than in other inflammatory or epithelial cells.

The negative association with lacto and neolacto series glycosphingolipid biosynthesis further suggests that GLA overexpression disrupts the synthesis of complex gangliosides, which are critical for cell-cell interactions (32, 33) and immune responses (34, 35). For instance, GD2, a tumor-associated ganglioside, enhances glioma stemness and suppresses microglial activation (36–39). By hydrolyzing precursor glycosphingolipids, GLA may indirectly attenuate the production of immunosuppressive ganglioside, yet paradoxically promote malignancy through alternative pathways. This dichotomy underscores the context-dependent roles of sphingolipid metabolism in gliomas and warrants further investigation into GLA’s substrate specificity and downstream effectors.

Sphingolipids, critical regulators of membrane integrity and cell signaling, are increasingly implicated in tumorigenesis. The primary enzymatic function of GLA is the lysosomal degradation of Gb3. Our pathway correlation analysis aligns with this role, showing a significant negative association between GLA expression and sphingolipid/glycosphingolipid metabolism. This suggests that high GLA activity in gliomas may lead to accelerated catabolism of specific glycosphingolipids like Gb3. Importantly, glycosphingolipids, including Gb3, are not merely structural components but are increasingly recognized as key modulators of signal transduction, cell adhesion, and immune responses. Accumulation of Gb3 has been implicated in altering membrane microdomain dynamics and facilitating pro-survival signaling. Conversely, its efficient degradation by GLA could reshape the lipid landscape of tumors and tumor-associated cells, potentially influencing interactions with immune cells. For instance, Gb3 and related lipids can modulate antigen presentation and T-cell activation. Therefore, GLA-driven Gb3 turnover might represent a novel metabolic axis through which glioma cells regulate local immune surveillance.

Beyond metabolism, GLA’s strong co-expression with EDEM2 (Spearman >0.85) unveils a mechanistic link to ERAD, a quality-control system that eliminates misfolded proteins. EDEM2, a key ERAD component, facilitates the recognition and retrotranslocation of aberrant glycoproteins for proteasomal degradation. Our data demonstrate that GLA knockdown significantly downregulates EDEM2 in glioma cells, suggesting a regulatory interplay between GLA and ERAD efficiency. In gliomas, chronic ER stress due to hypoxia, nutrient deprivation, or rapid proliferation necessitates robust ERAD activity to maintain proteostasis. By modulating EDEM2, GLA may enhance ERAD capacity, enabling tumor cells to survive proteotoxic stress and resist apoptosis. This aligns with our observation that GLA positively correlates with apoptosis-related pathways, potentially reflecting ERAD-mediated evasion of unfolded protein response (UPR)-triggered cell death.

Notably, ERAD activation has been linked to temozolomide resistance in glioblastoma by clearing drug-induced misfolded proteins (40–42). Thus, the GLA-EDEM2 axis may represent a therapeutic vulnerability: targeting GLA could disrupt ERAD, sensitizing tumors to chemotherapy or radiation. The lack of impact on EDEM1/EDEM3 underscores the specificity of this interaction, highlighting EDEM2 as a unique downstream effector of GLA. Although co-IP did not demonstrate a direct physical interaction, silencing of GLA resulted in a decrease in its metabolic substrate, Gb3. This observation leads us to propose that GLA, through its enzymatic degradation of Gb3, influences the cellular lipid environment, potentially stabilizing or enhancing the expression and activity of EDEM2 and the ERAD pathway. ERAD efficiency is increasingly recognized as lipid-sensitive: perturbations of lipid homeostasis can impair ERAD substrate glycan trimming and dislocation while activating the unfolded protein response, and specific lipid remodeling events, such as elevated ceramides, can directly restrict ERAD by limiting substrate extraction from the ER membrane (43). In lysosomes, glycosphingolipid accumulation has been linked to ER stress/UPR engagement and enhanced reliance on proteostasis pathways, and Gb3 accumulation has been discussed in the persistence of ER stress and ERAD activation (44). In parallel, misprocessing/ER retention of α-galactosidase A variants can itself induce ER stress and UPR signaling, providing an additional route by which GLA perturbation could intersect with ER homeostasis without requiring direct binding to ERAD components (45).

Both temozolomide (TMZ) and radiotherapy can impose substantial proteotoxic and metabolic stress on glioma cells, and accumulating evidence indicates that activation of ER stress and UPR signaling and downstream quality-control pathways (including ERAD) contributes to adaptive survival under these insults. In particular, ER stress-inducing strategies have been reported to sensitize glioma cells to TMZ through modulation of DNA repair-related factors, suggesting that the ER stress state can shape TMZ responsiveness. Moreover, irradiation has been linked to adaptive activation of ER stress response pathways, which may promote post-irradiation viability and resistance phenotypes. In this context, our observation that GLA expression correlates with ERAD-related signatures and that GLA perturbation is accompanied by changes in ERAD-associated components raises the possibility that high GLA activity could be coupled to an enhanced capacity to maintain proteostasis under therapy-induced ER stress. Although we did not directly evaluate TMZ or radiation combinations in the present study, these considerations support a biologically grounded hypothesis that GLA-high gliomas may exhibit a distinct adaptive state and could potentially benefit from therapeutic strategies that disrupt ER stress tolerance or proteostasis buffering. Future work combining GLA modulation with TMZ or radiation, together with direct readouts of ER stress and ERAD activity, will be required to establish causality and assess therapeutic relevance.

GLA expression was specifically enriched in tumor-associated astrocytes, particularly in higher-grade tumors. Astrocytes are essential components of the tumor microenvironment, known to secrete factors that promote invasion and suppress immune function. Elevated GLA activity in these cells may modify their glycosphingolipid profile, thereby influencing their interactions with tumor cells and infiltrating immune cells, such as macrophages and T cells. The CellChat analysis, which reveals more intensive intercellular communication in tumors, supports the idea that GLA-high astrocytes may serve as key signaling hubs. Consequently, GLA may regulate glioma immunity not only intrinsically within tumor cells but also extrinsically by reprogramming the astrocytic compartment.

From a therapeutic perspective, small-molecule inhibitors of GLA or EDEM2 could disrupt proteostasis and sphingolipid metabolism, synergizing with standard therapies. The cell type-specific expression of GLA in astrocytes further suggests that tumor-associated glia may be critical mediators of treatment resistance, warranting exploration of glial-targeted therapies. Additionally, the correlation between GLA and ferroptosis pathways introduces intriguing possibilities, as ferroptosis inducers are being tested in glioma trials. GLA’s role in galactose metabolism may also influence the efficacy of dietary interventions targeting carbohydrate availability.

In conclusion, GLA emerges as a multifaceted regulator of glioma progression, bridging sphingolipid metabolism, ERAD pathways, and clinical aggressiveness. Its prognostic robustness, conserved across ethnic cohorts, and mechanistic novelty position it as a promising candidate for biomarker-driven therapy. By unraveling the GLA-EDEM2 axis and its metabolic implications, this study may offer a novel therapeutic strategy for aggressive gliomas characterized by high GLA expression.

## Conclusion

In conclusion, our analysis demonstrates that GLA serves as a crucial prognostic biomarker and functional driver in gliomas. Elevated levels of GLA are predictive of aggressive phenotypes, characterized by higher WHO grades, therapy resistance, and poor survival outcomes. Mechanistically, GLA regulates the glycosphingolipid metabolism to upregulate EDEM2, although the mechanistic relationship remains to be fully established. Overall, our findings identify GLA as a promising candidate biomarker and a potential biological contributor to glioma progression.

## Data Availability

The raw data supporting the conclusions of this article will be made available by the authors, without undue reservation.

## Supplementary material

Supplementary Figure 1 The GLA expression in GSE103224. **(A)** The UMAP plot of the GSE103224 single-cell data unveils distinct cell types. **(B)** The GLA expression in various groups of GSE103224.

Supplementary Figure 2 The impact of GLA knockdown on cell migration was assessed in U251 cells. Migrated cells were quantified and analyzed by a two-tailed Student’s t-test. ****p* < 0.001.
