# Supplementary figures and images for: Sphingolipid metabolic enzyme GLA expression in gliomas: prognostic implications and therapeutic potential
Source: Front Oncol. 2026 Mar 24;16:1773193. doi: 10.3389/fonc.2026.1773193 (PMC13053272; doi:10.3389/fonc.2026.1773193)

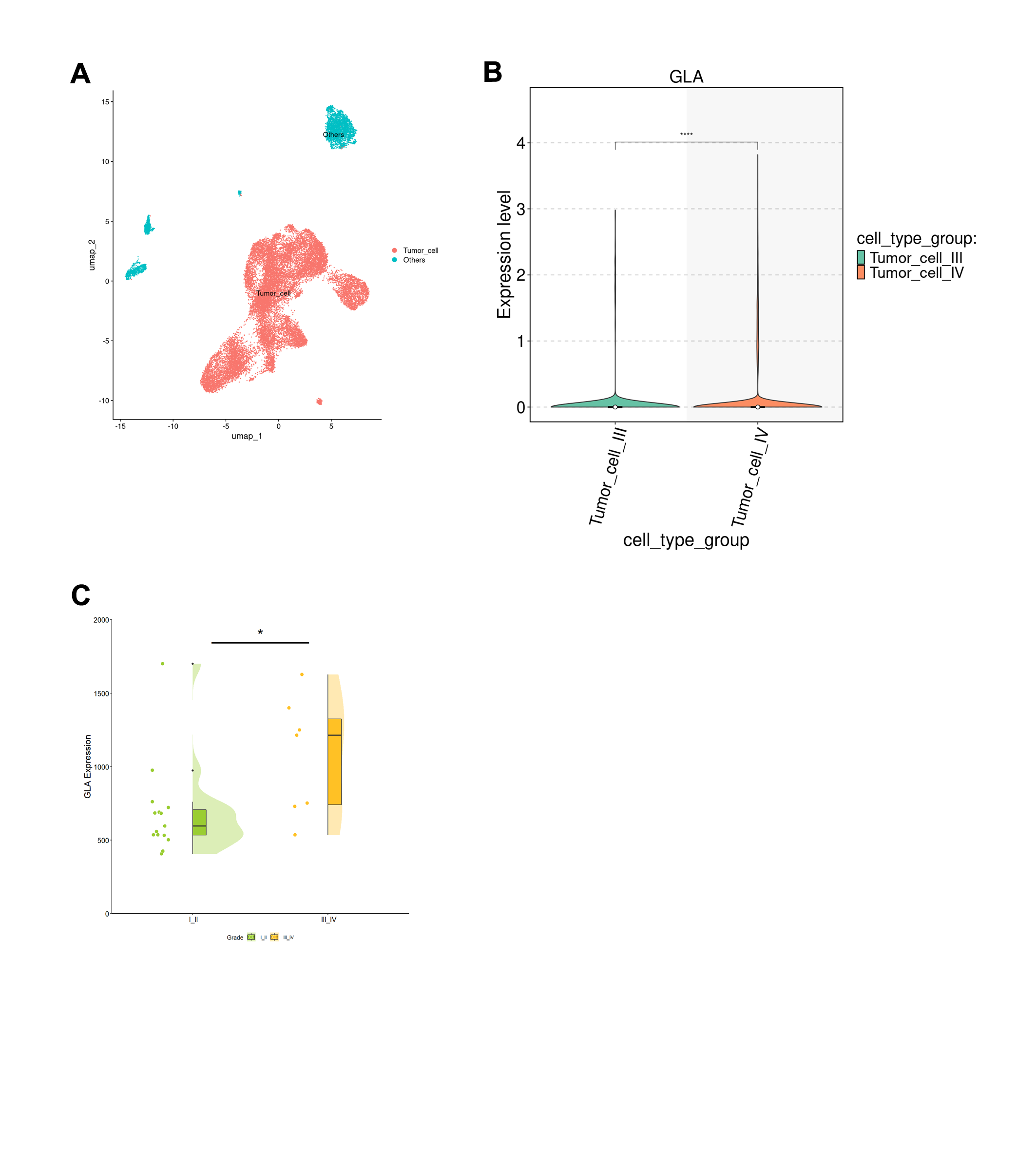

Supplement: Supplementary Figure 1 — The GLA expression in GSE103224. (A) The UMAP plot of the GSE103224 single-cell data unveils distinct cell types. (B) The GLA expression in various groups of GSE103224. [file Image1.tif]

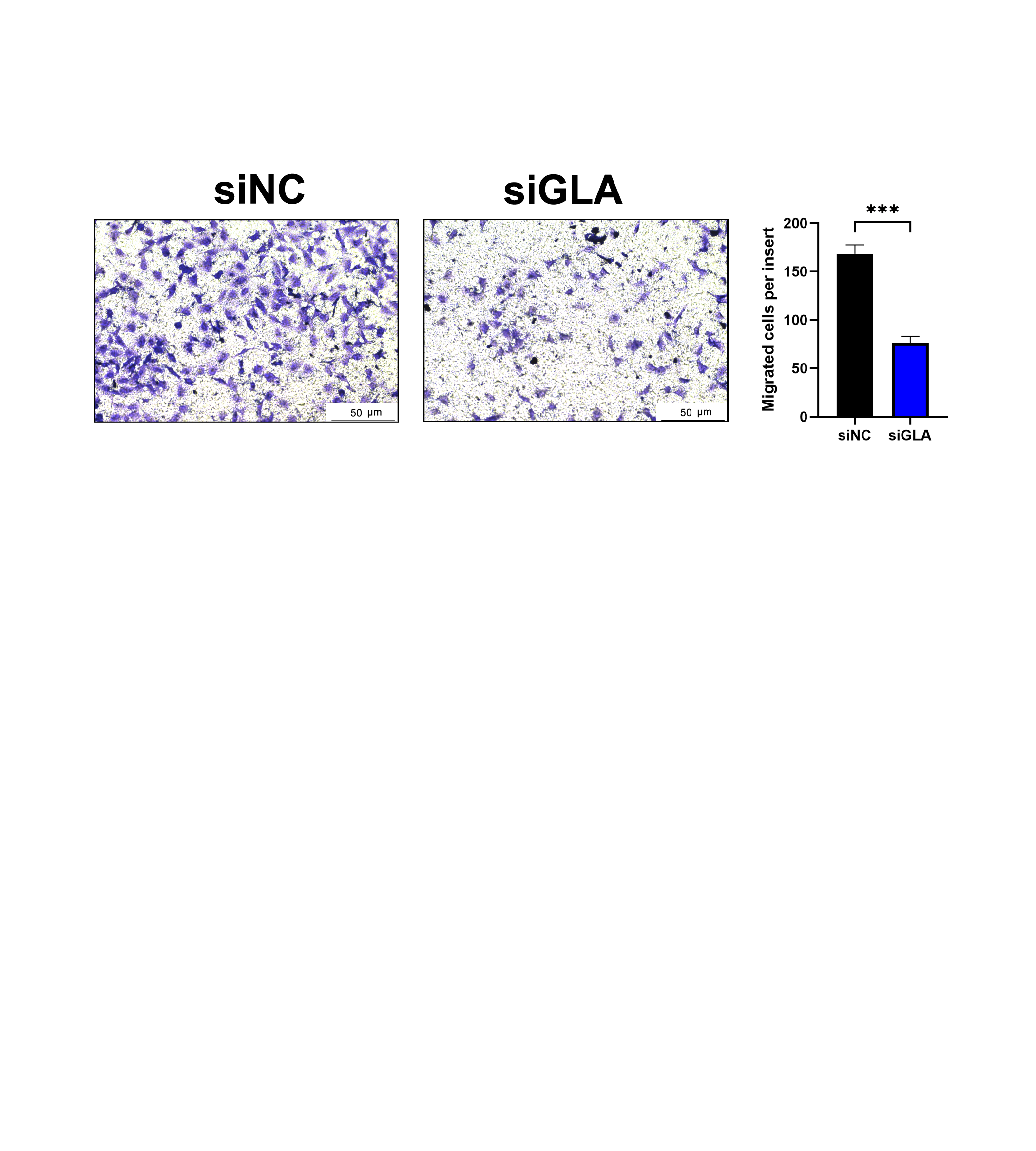

Supplement: Supplementary Figure 2 — The impact of GLA knockdown on cell migration was assessed in U251 cells. Migrated cells were quantified and analyzed by a two-tailed Student’s t-test. ***p < 0.001. [file Image2.tif]

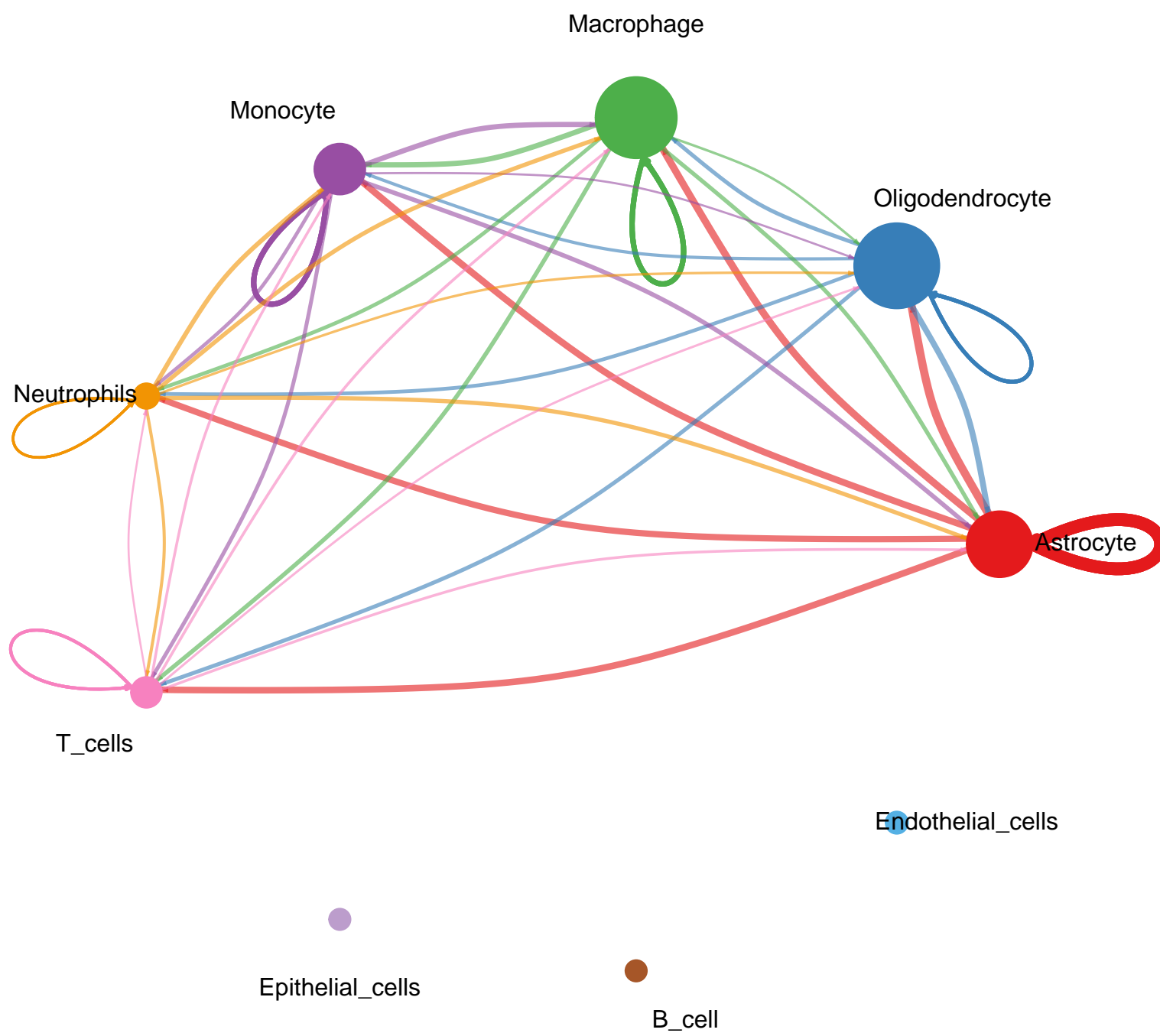

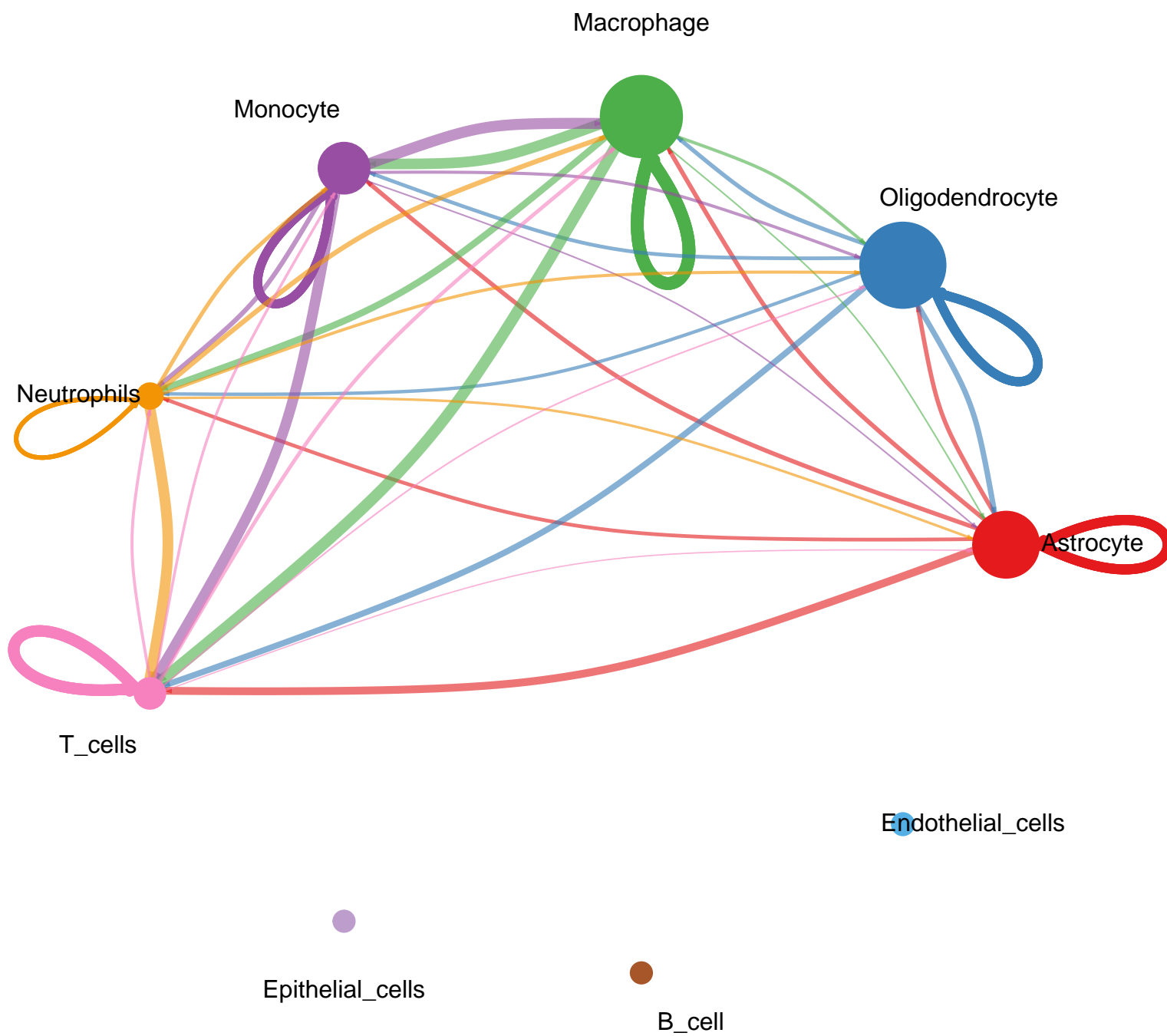

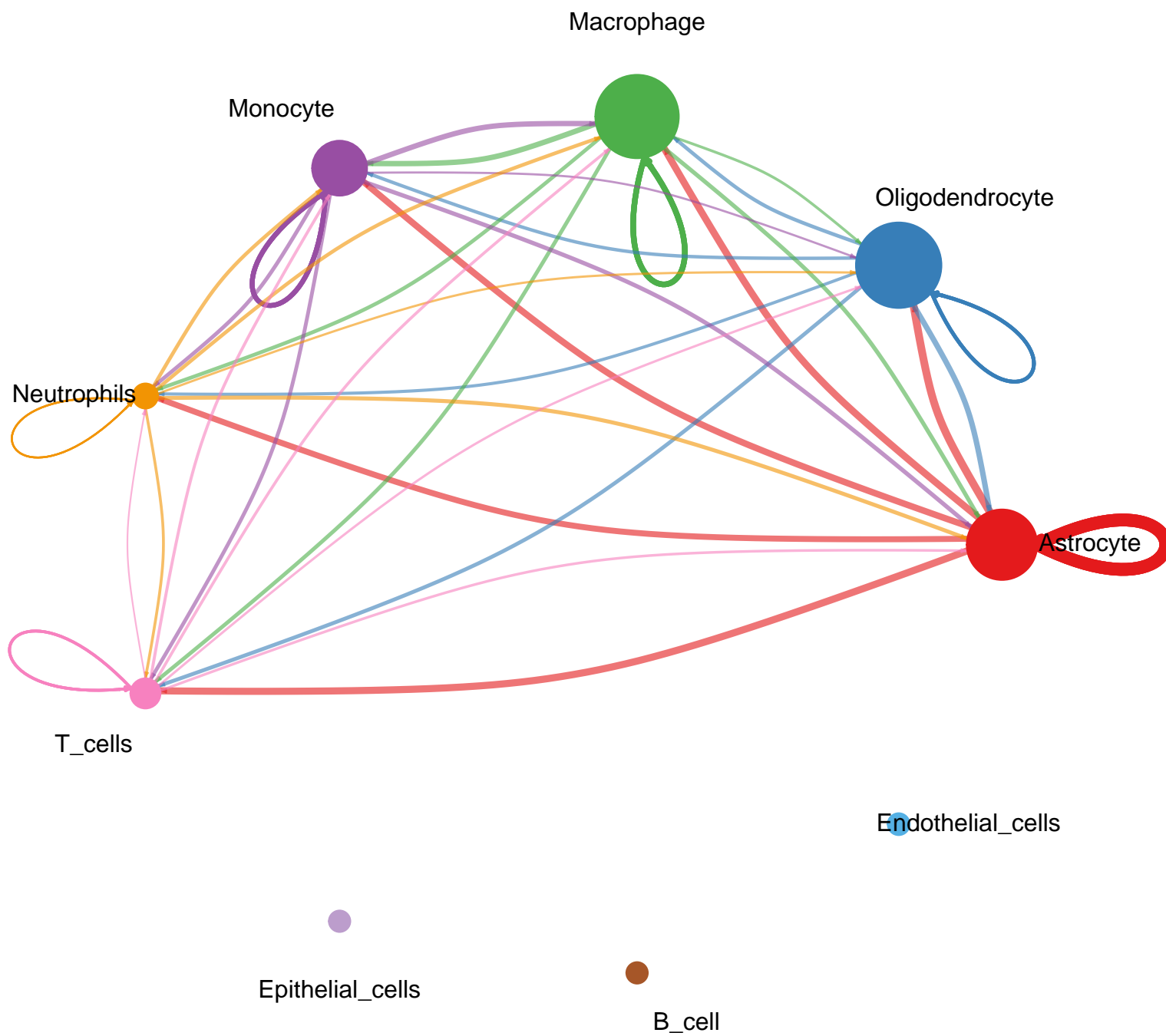

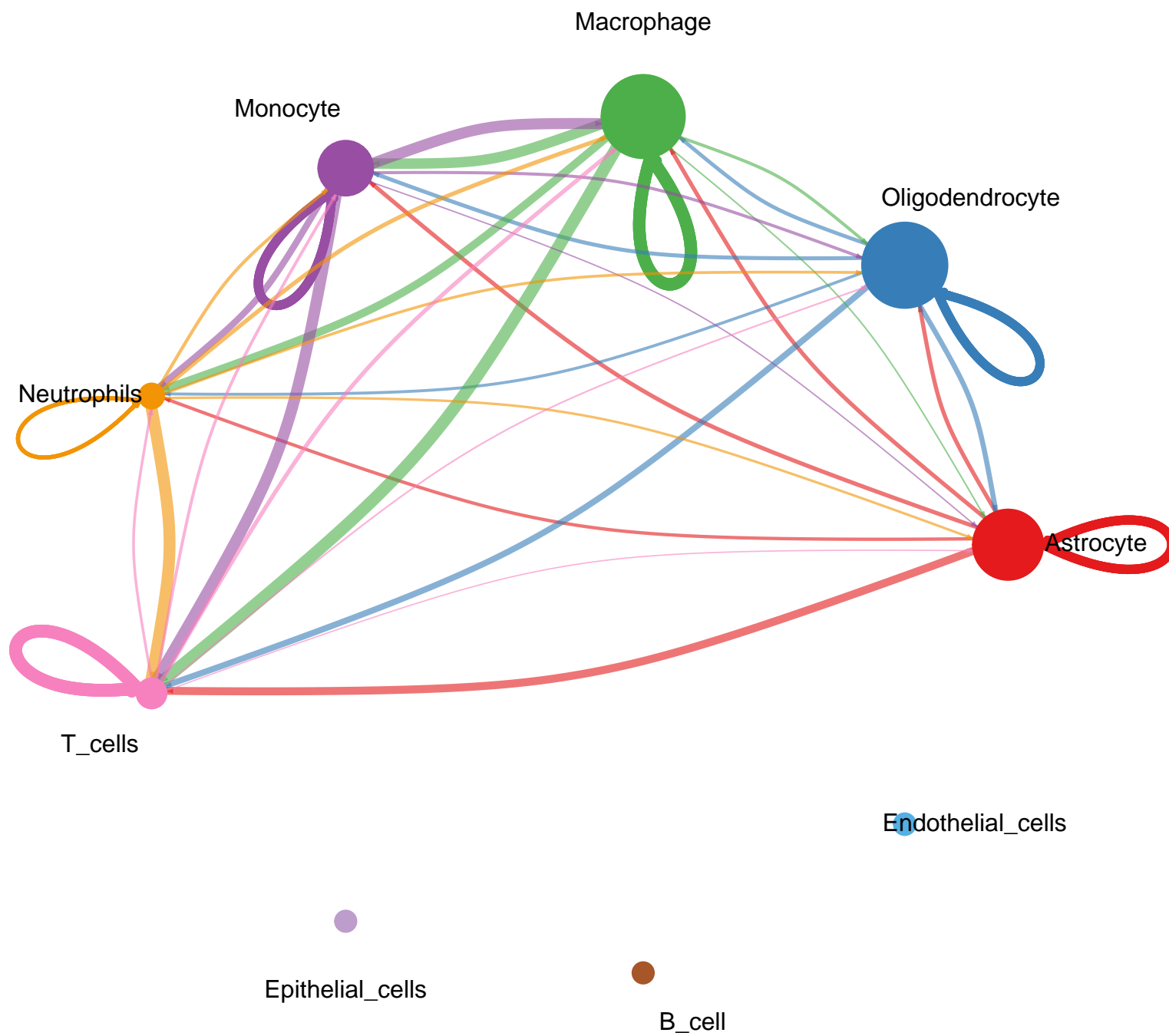

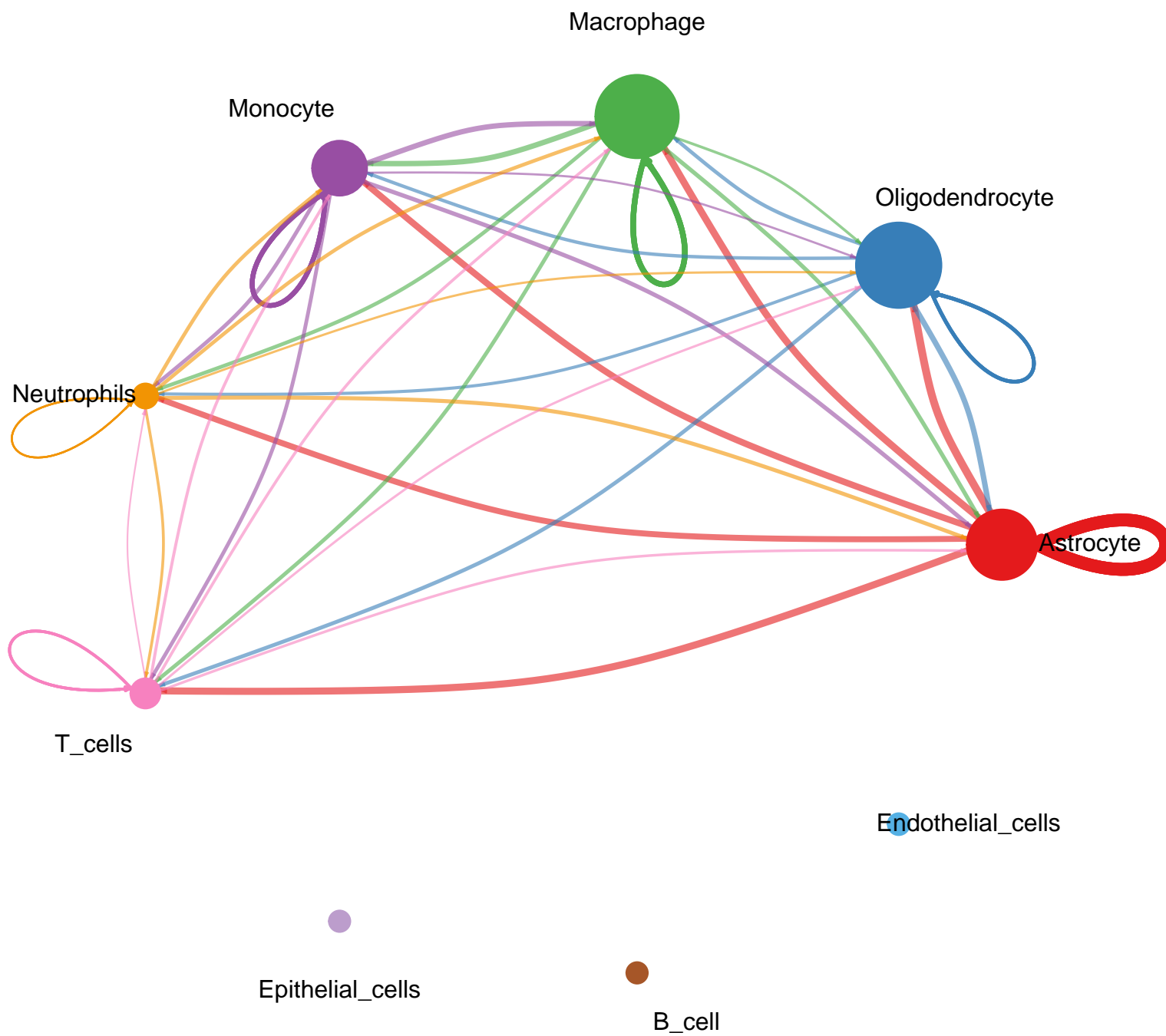

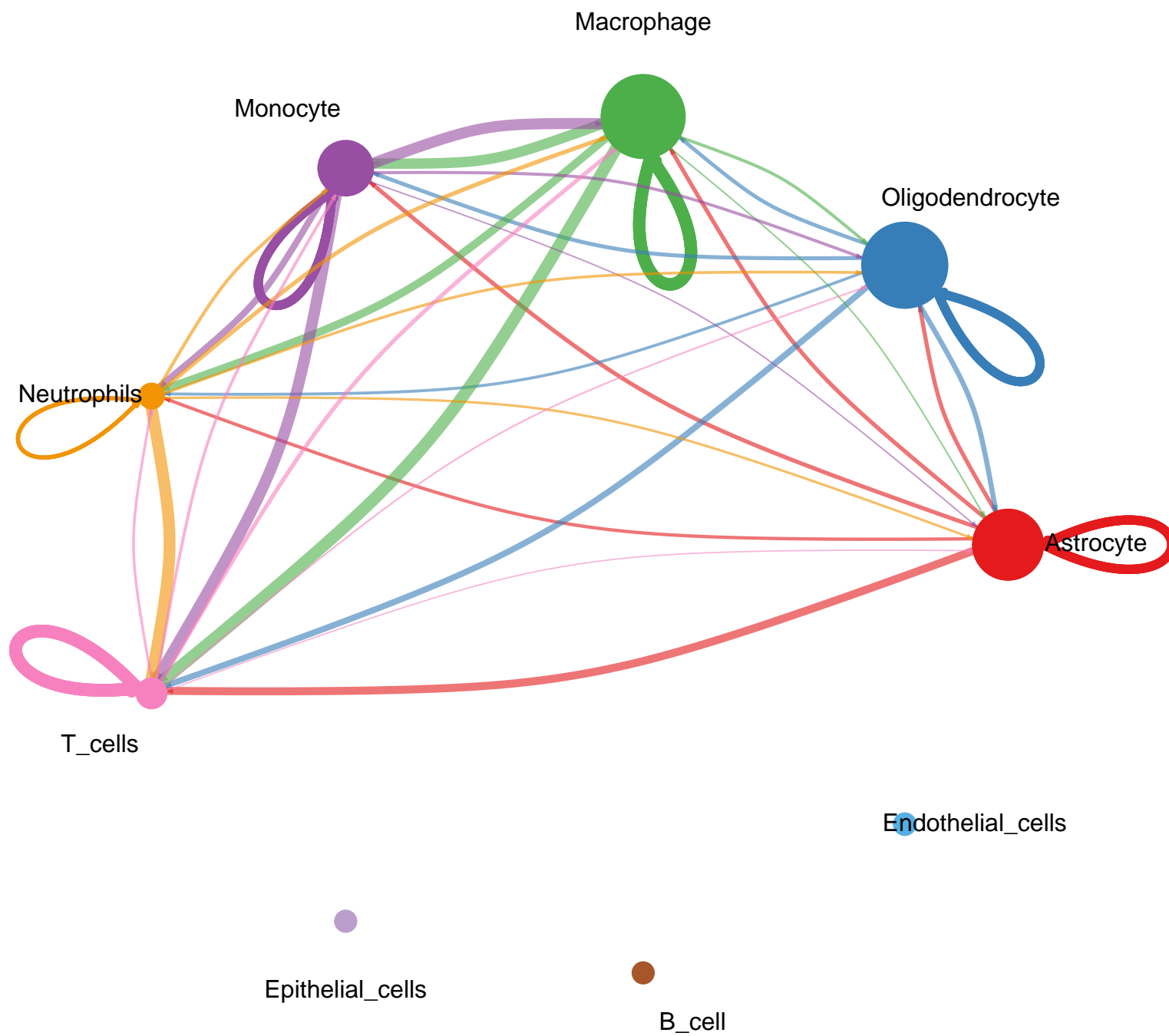

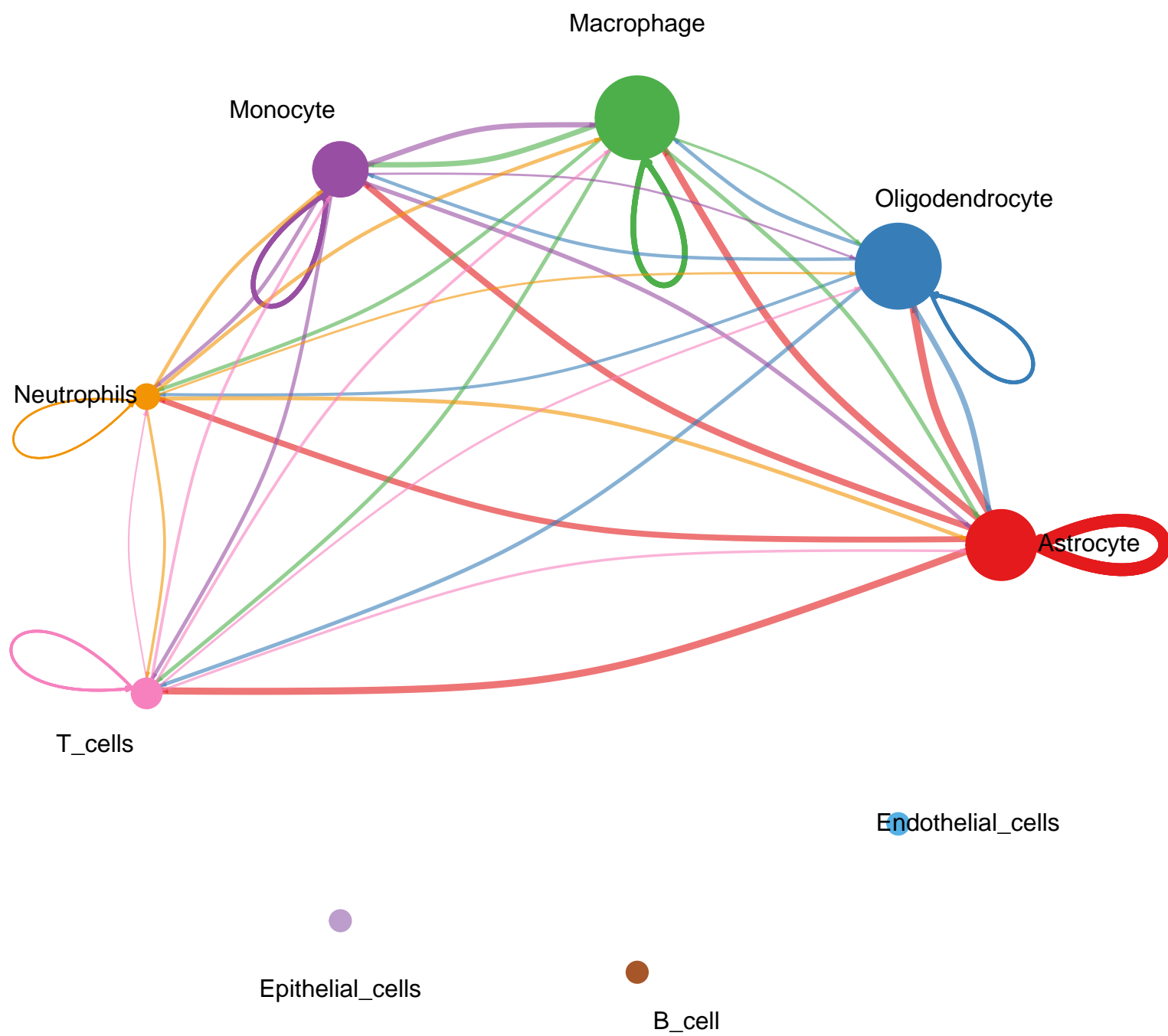

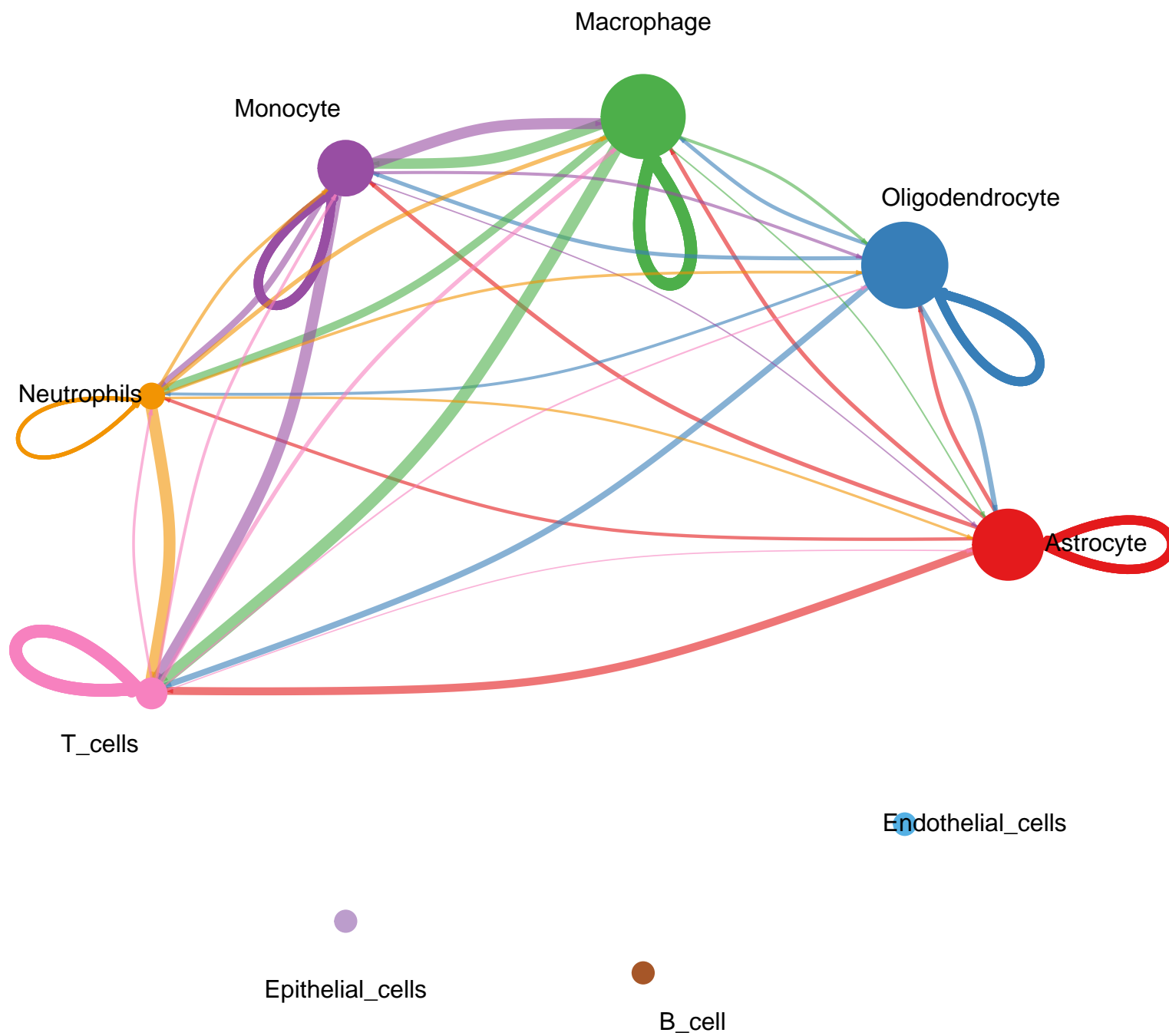

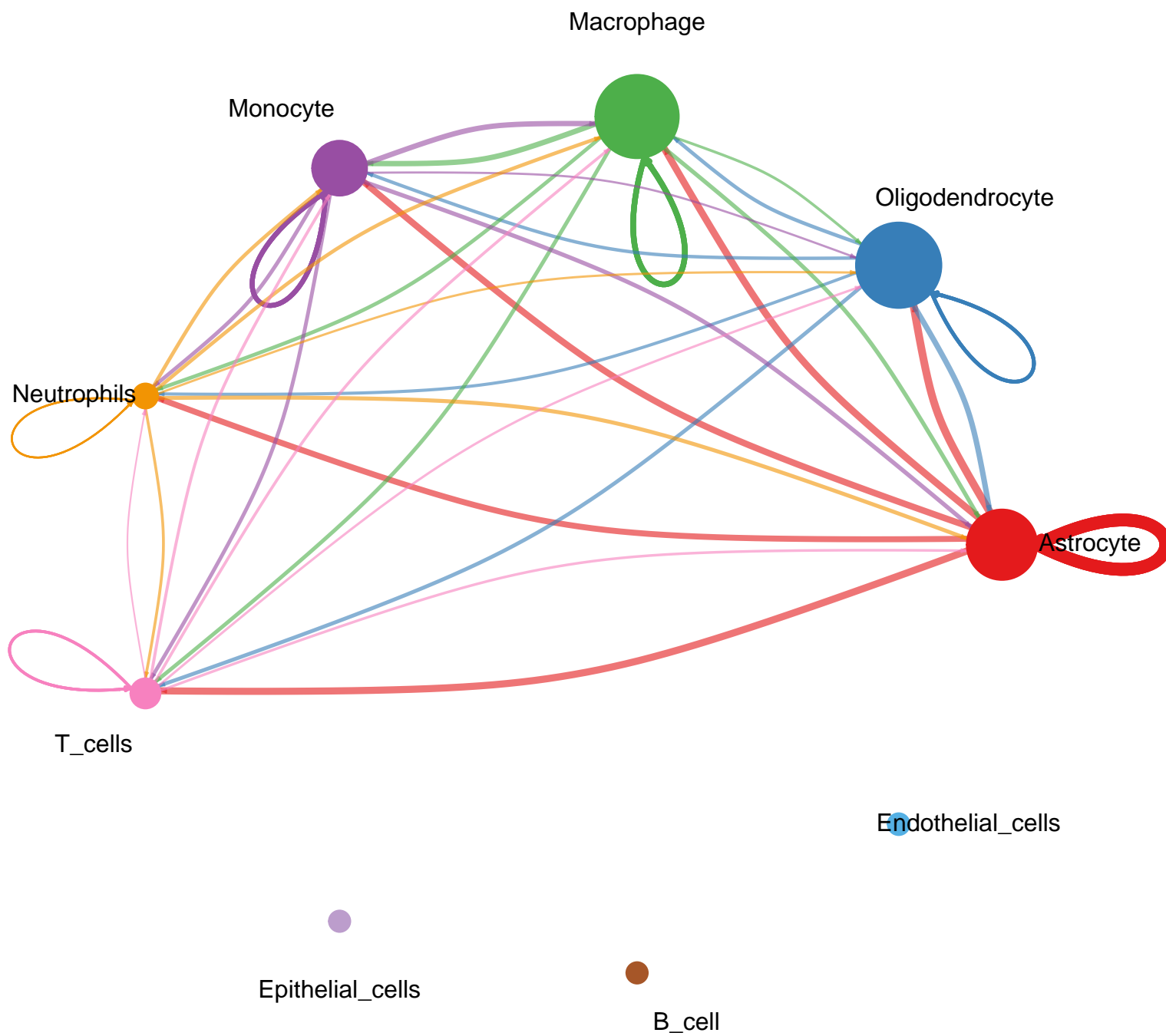

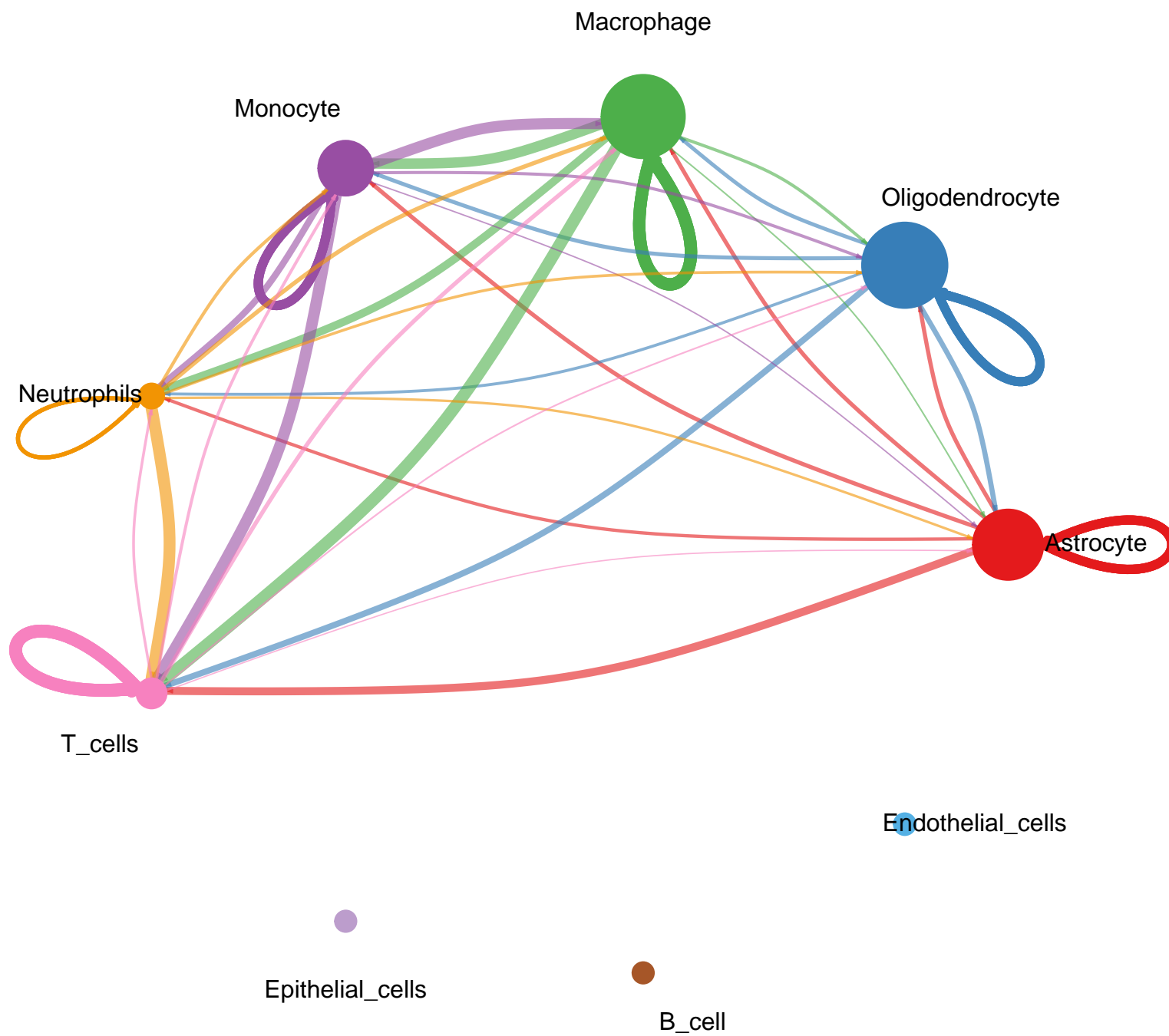

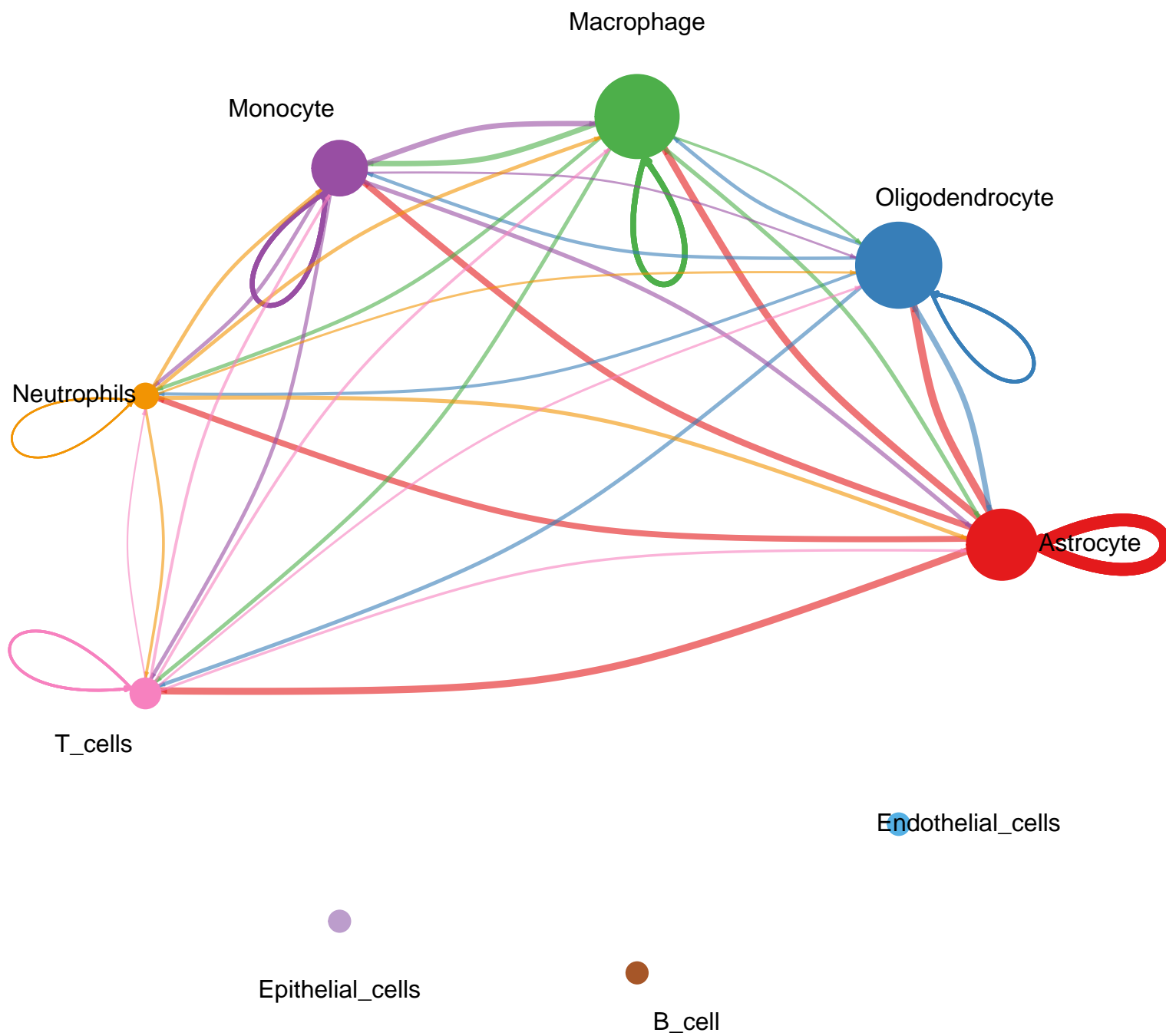

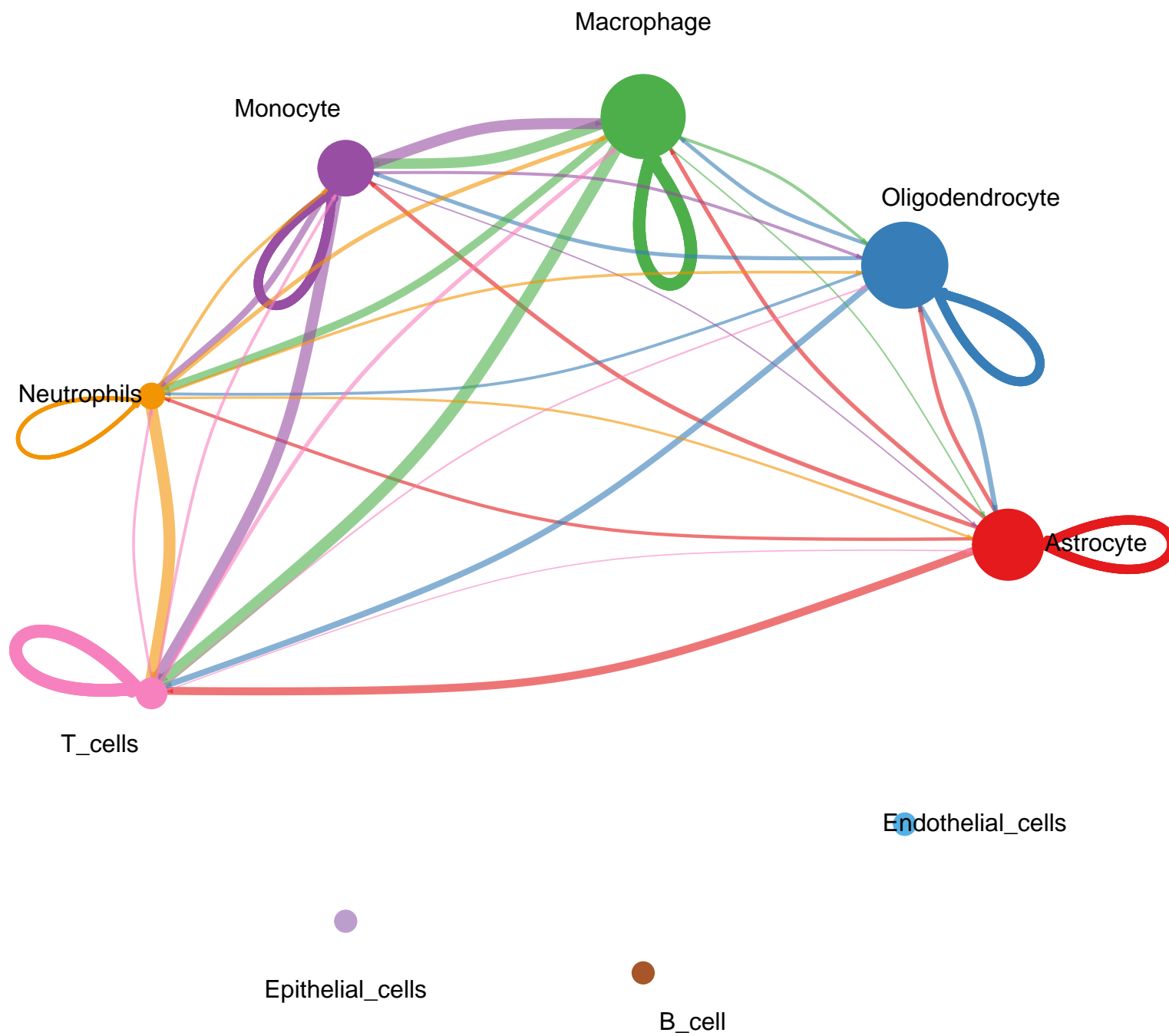

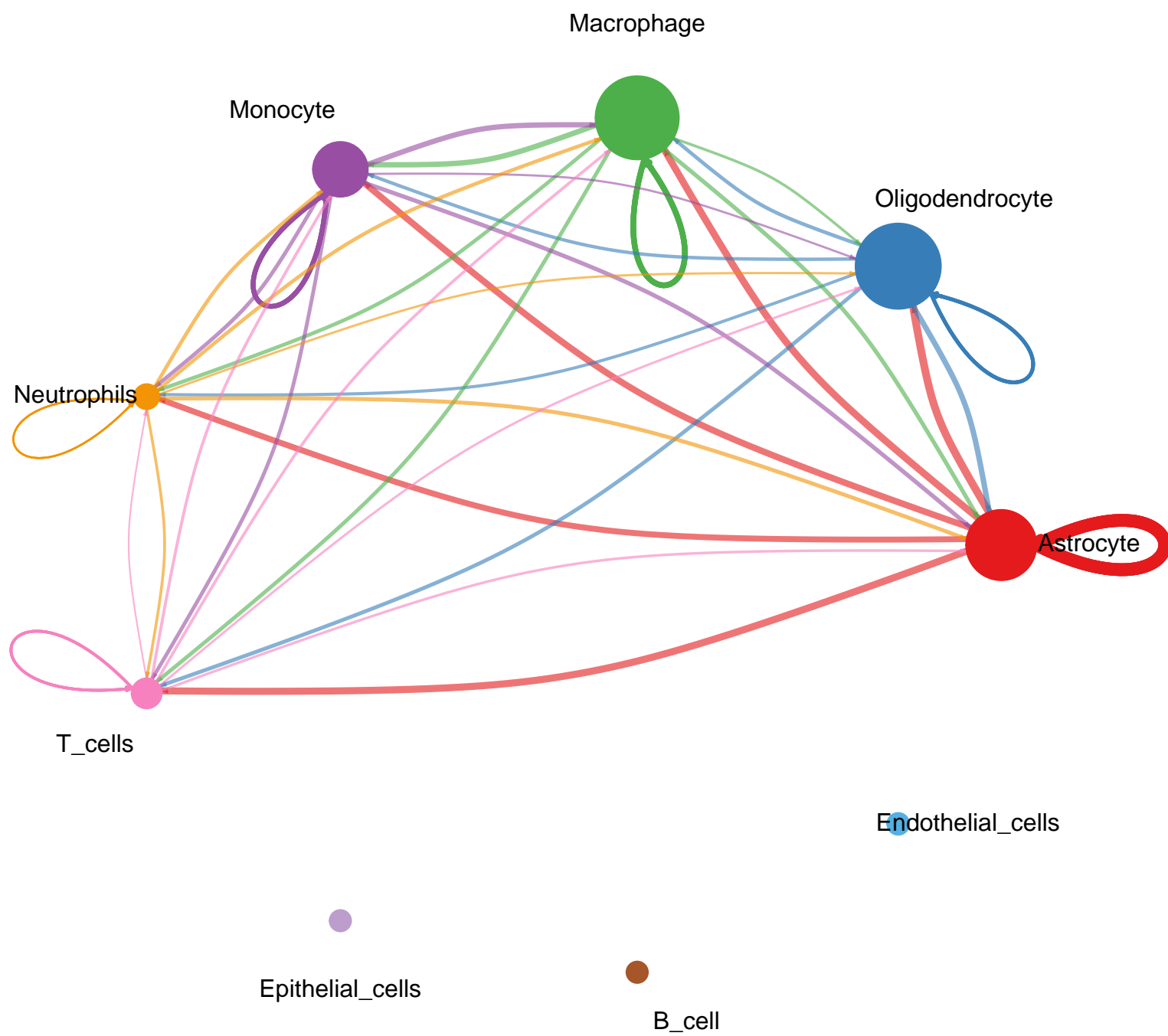

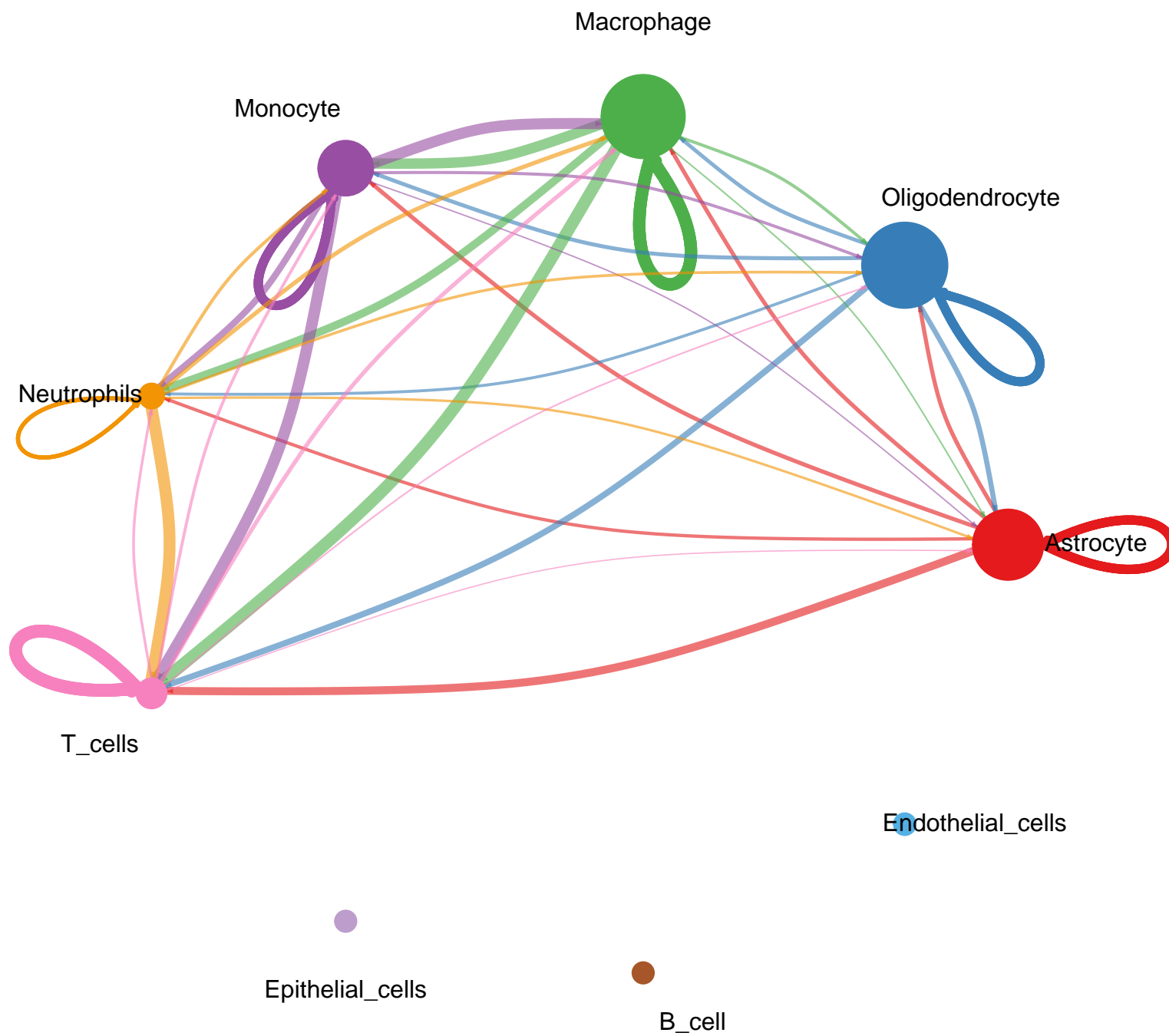

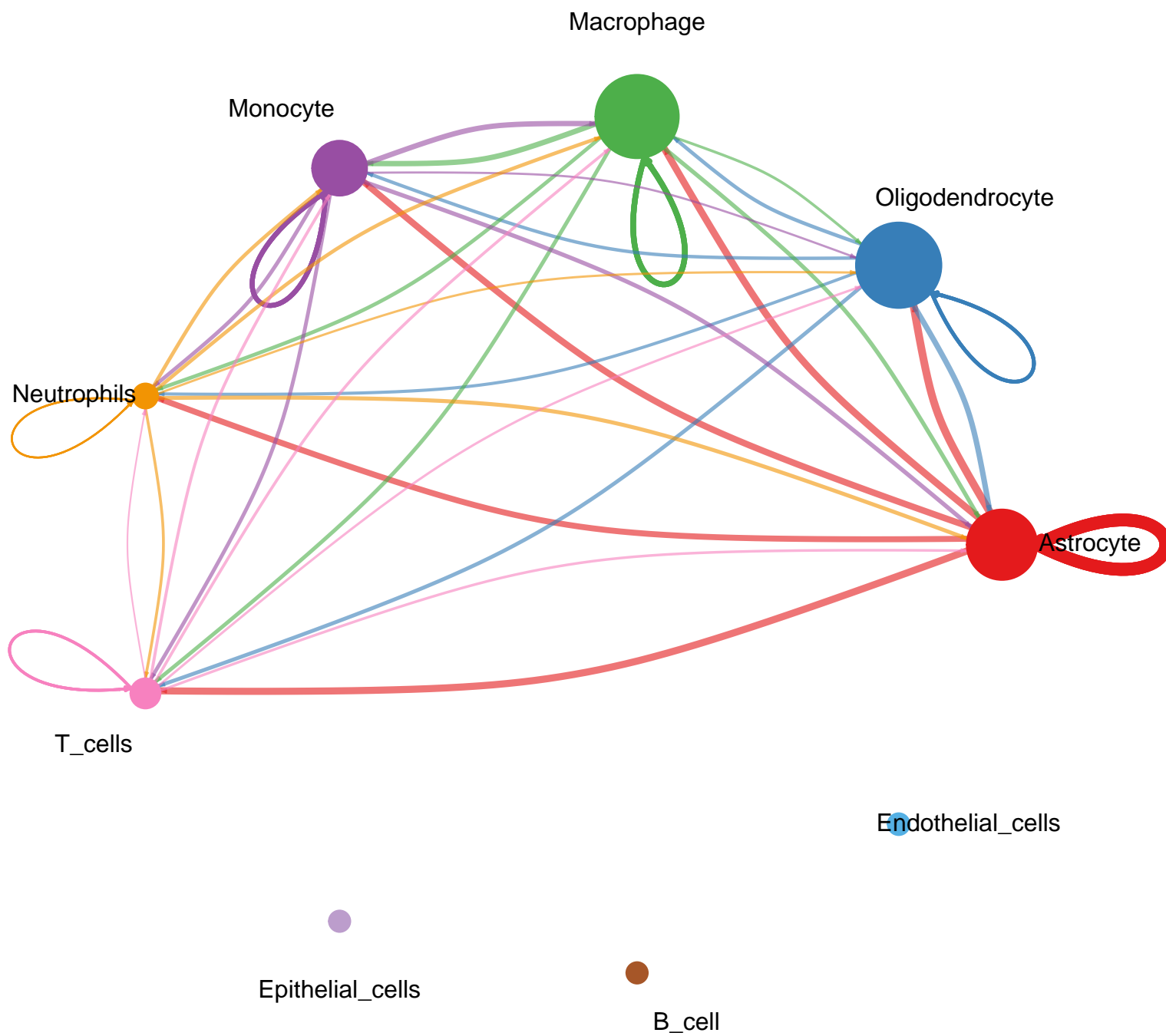

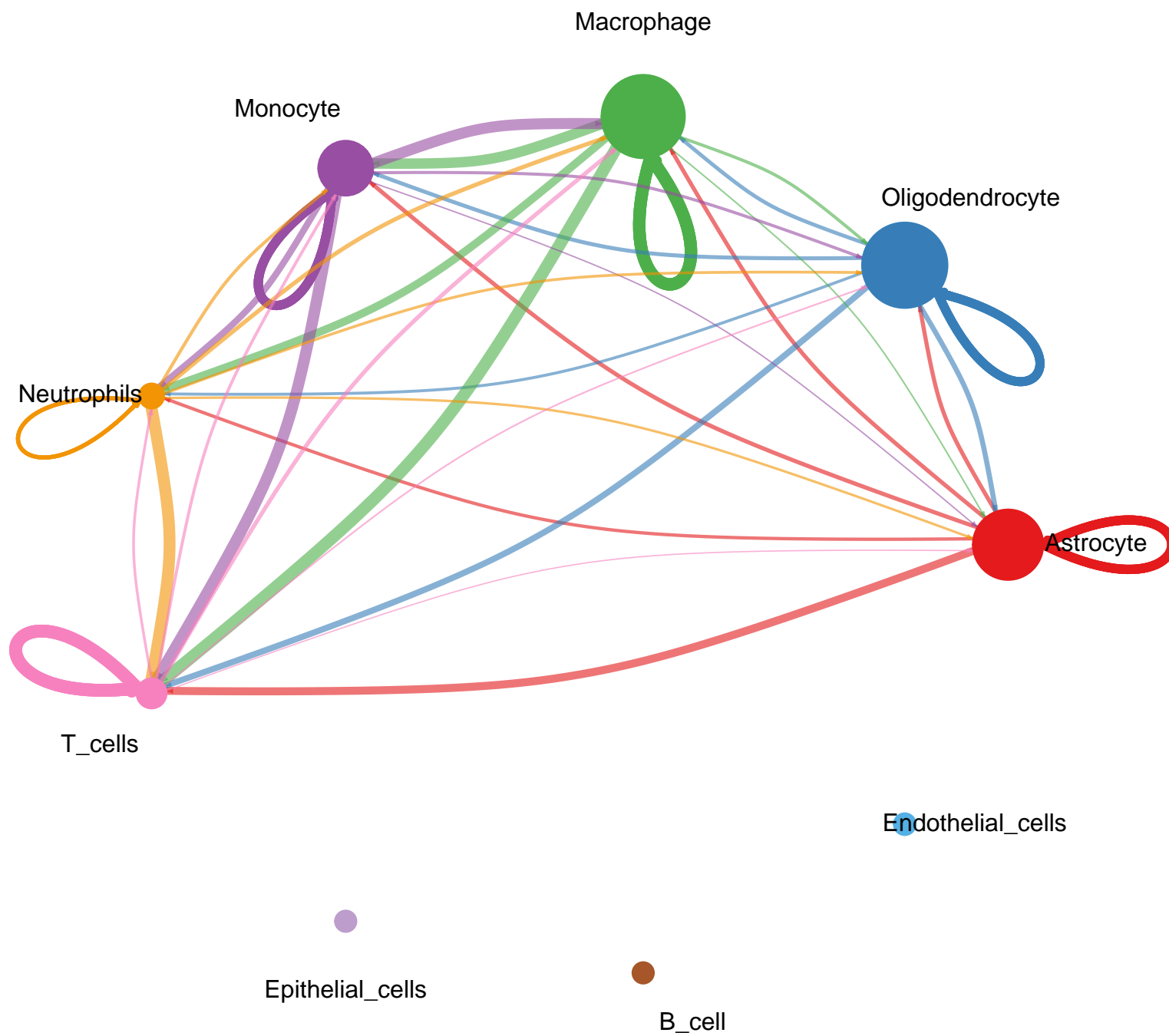

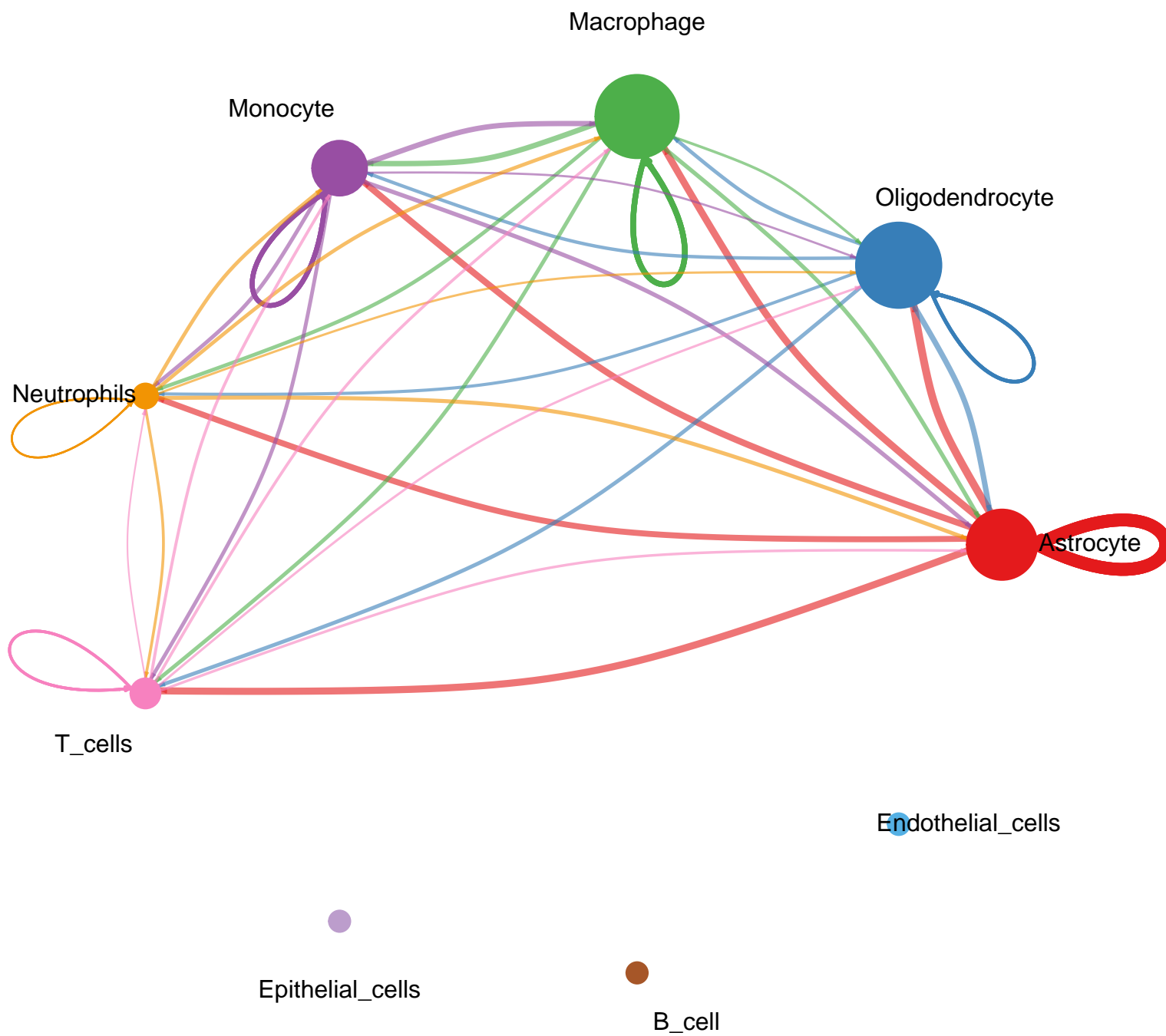

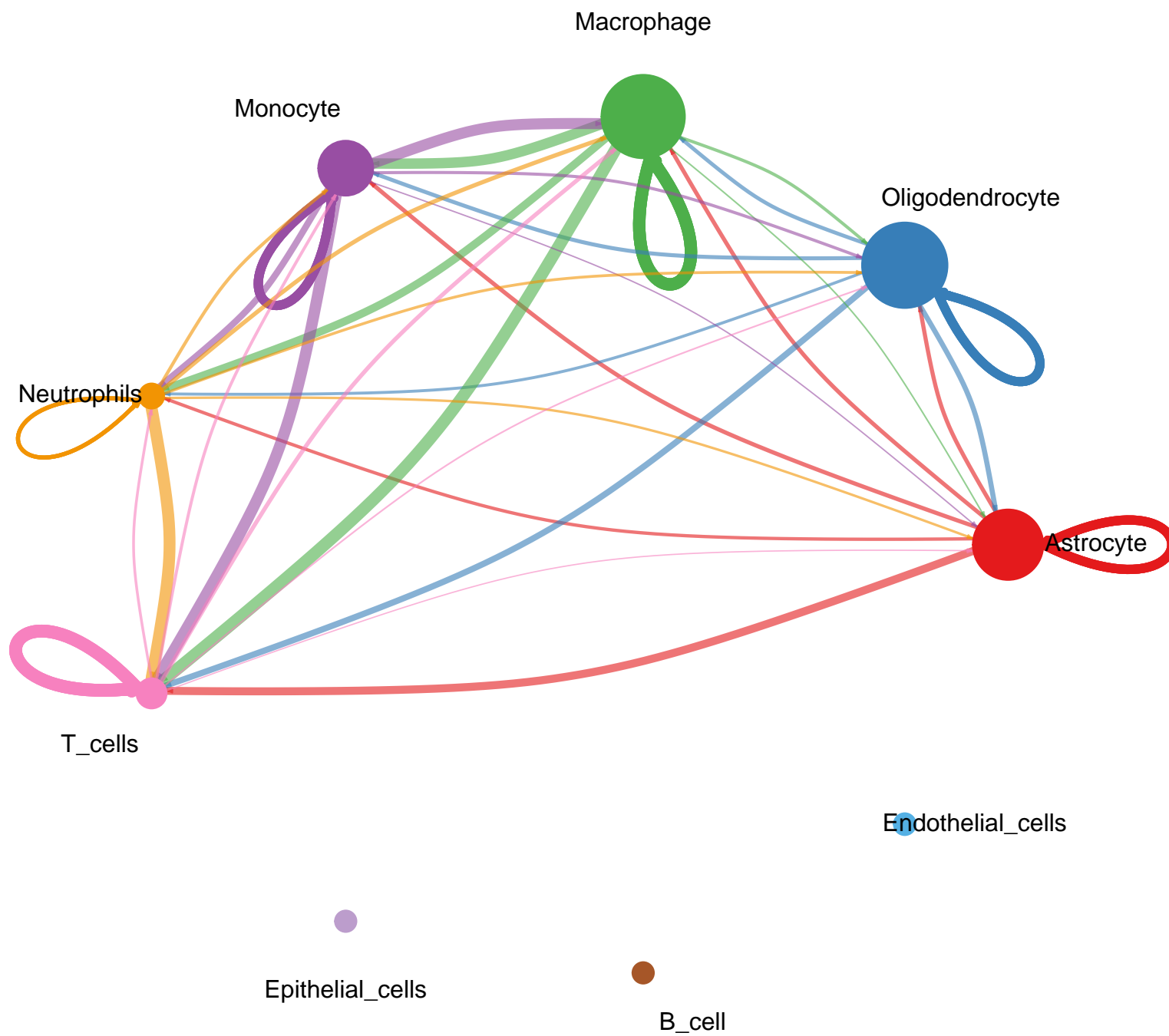

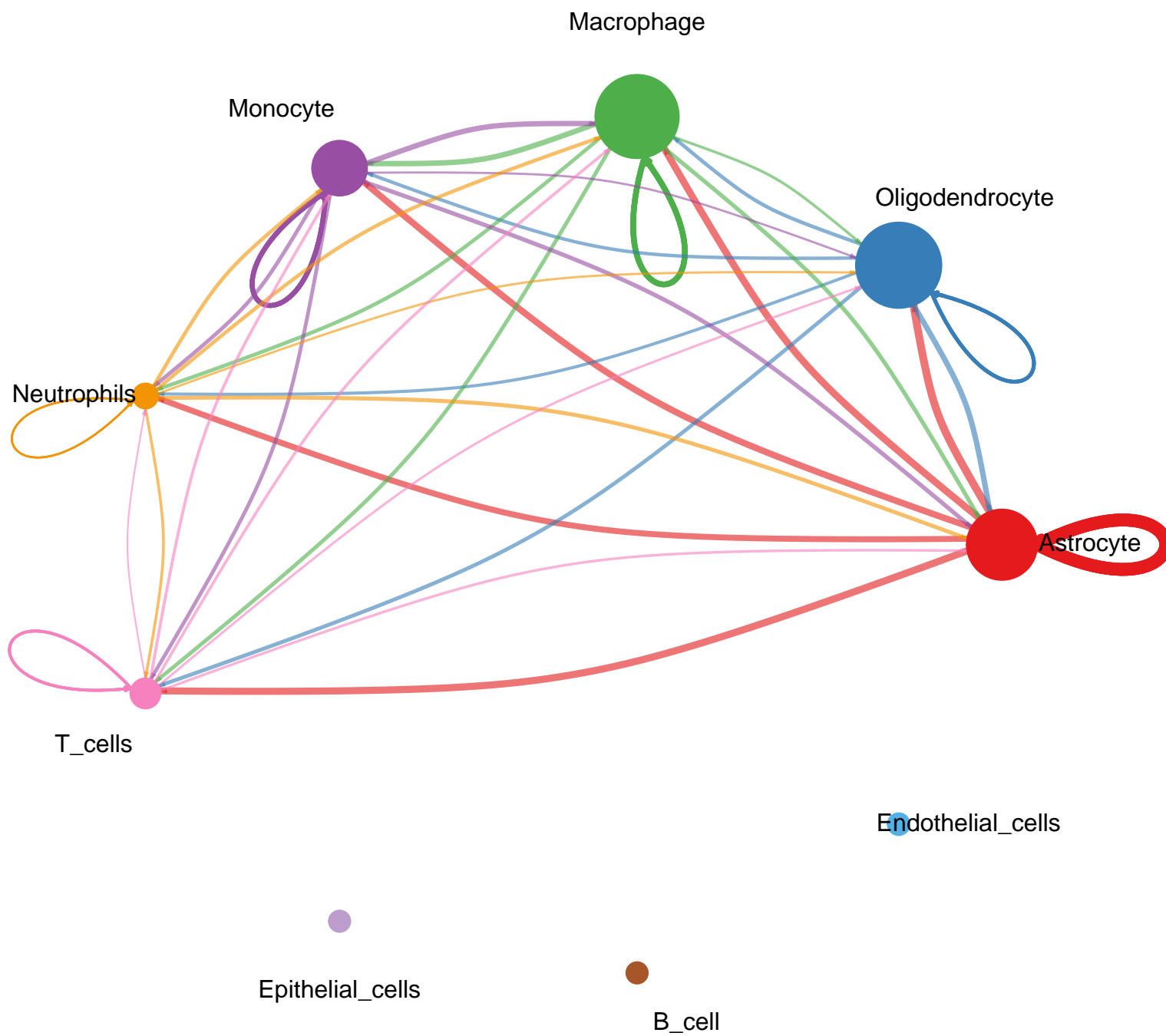

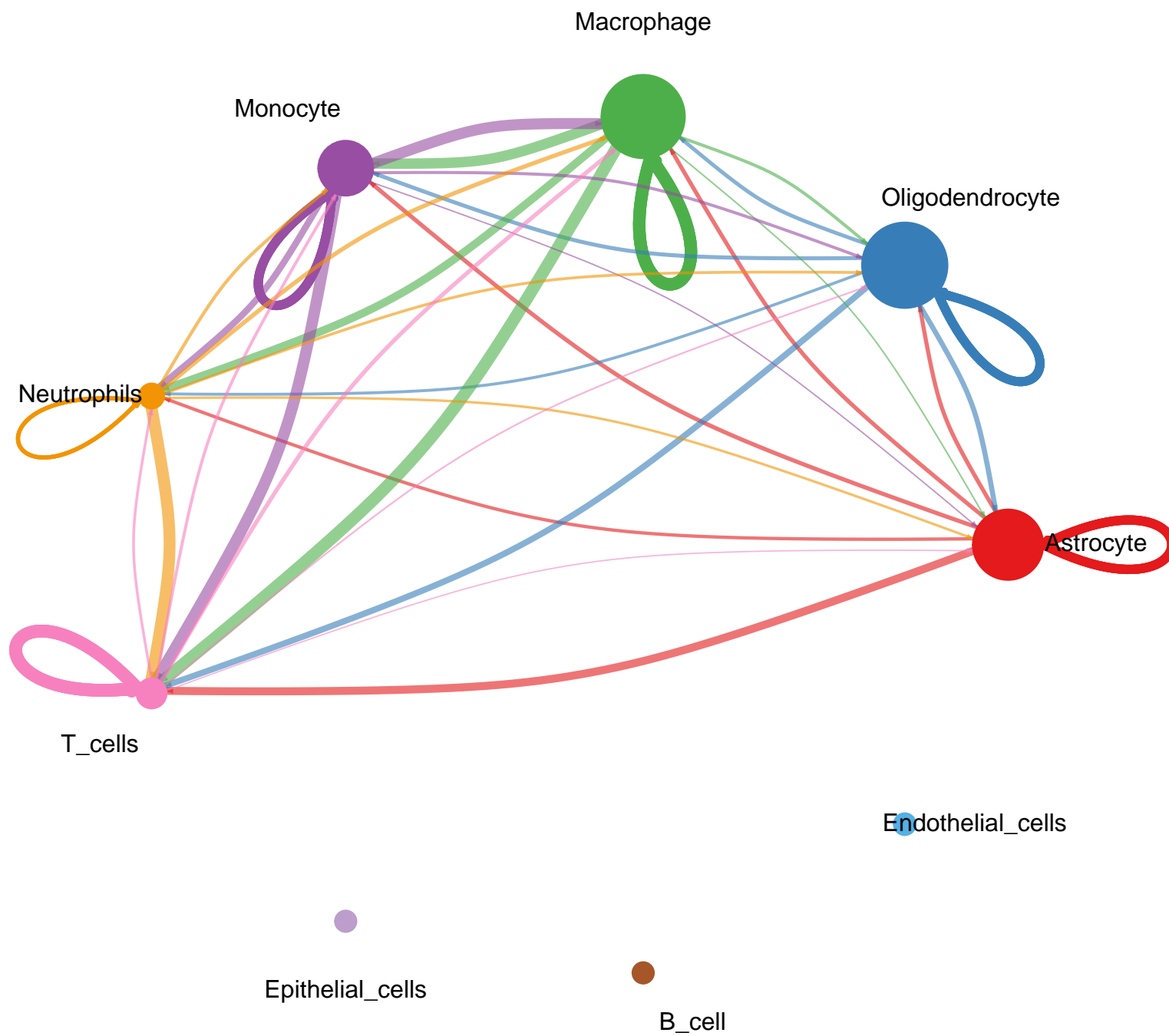

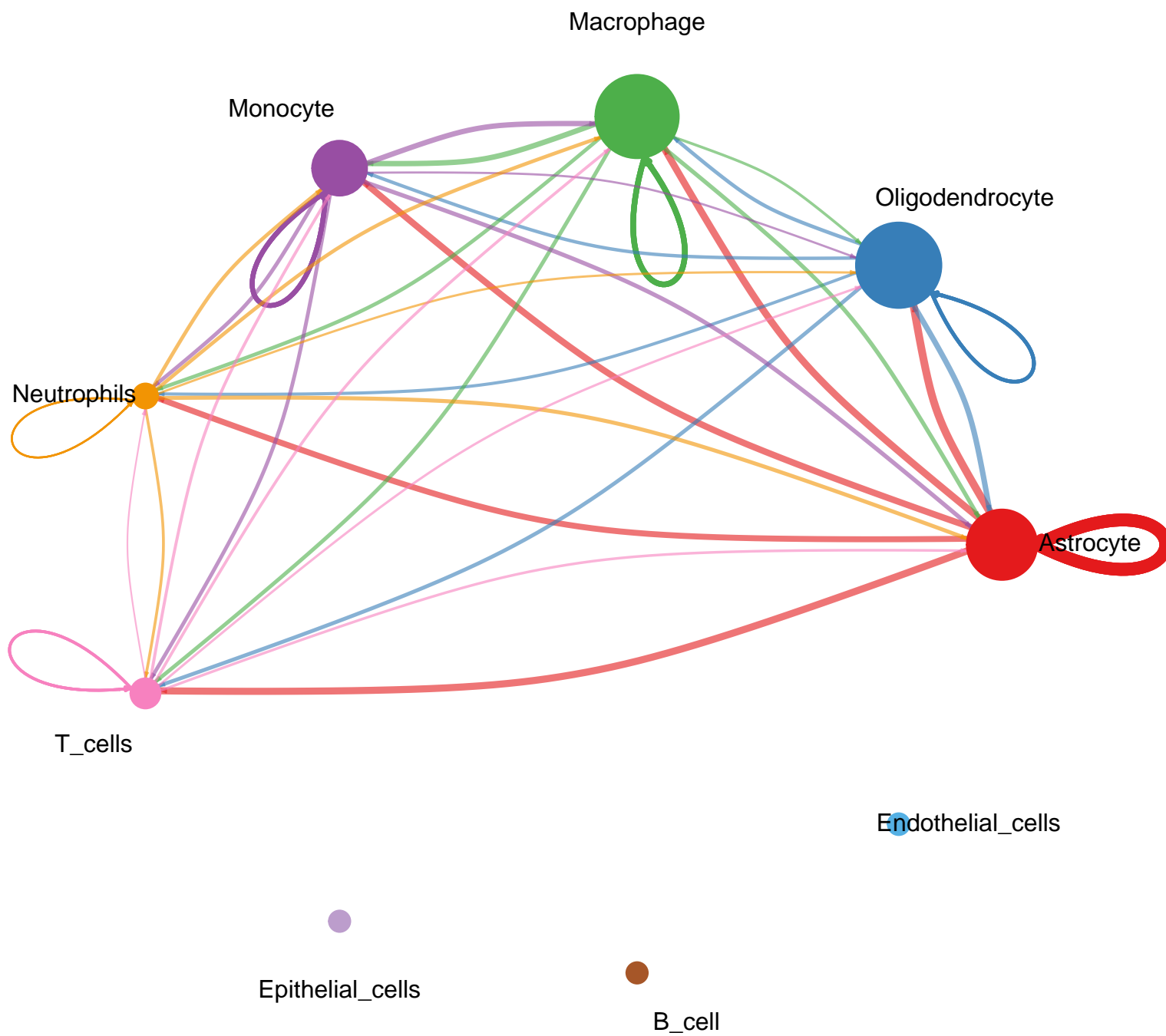

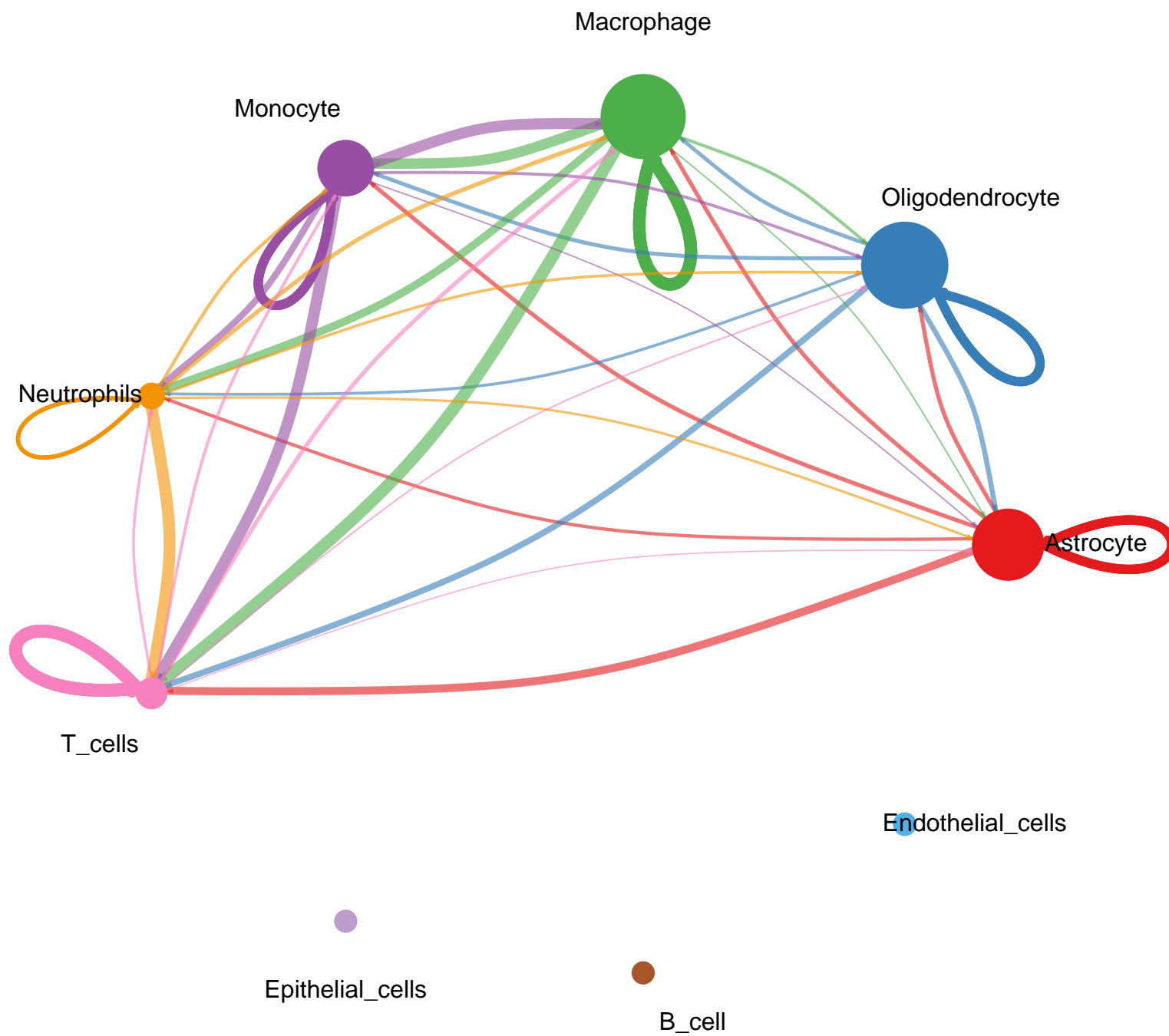

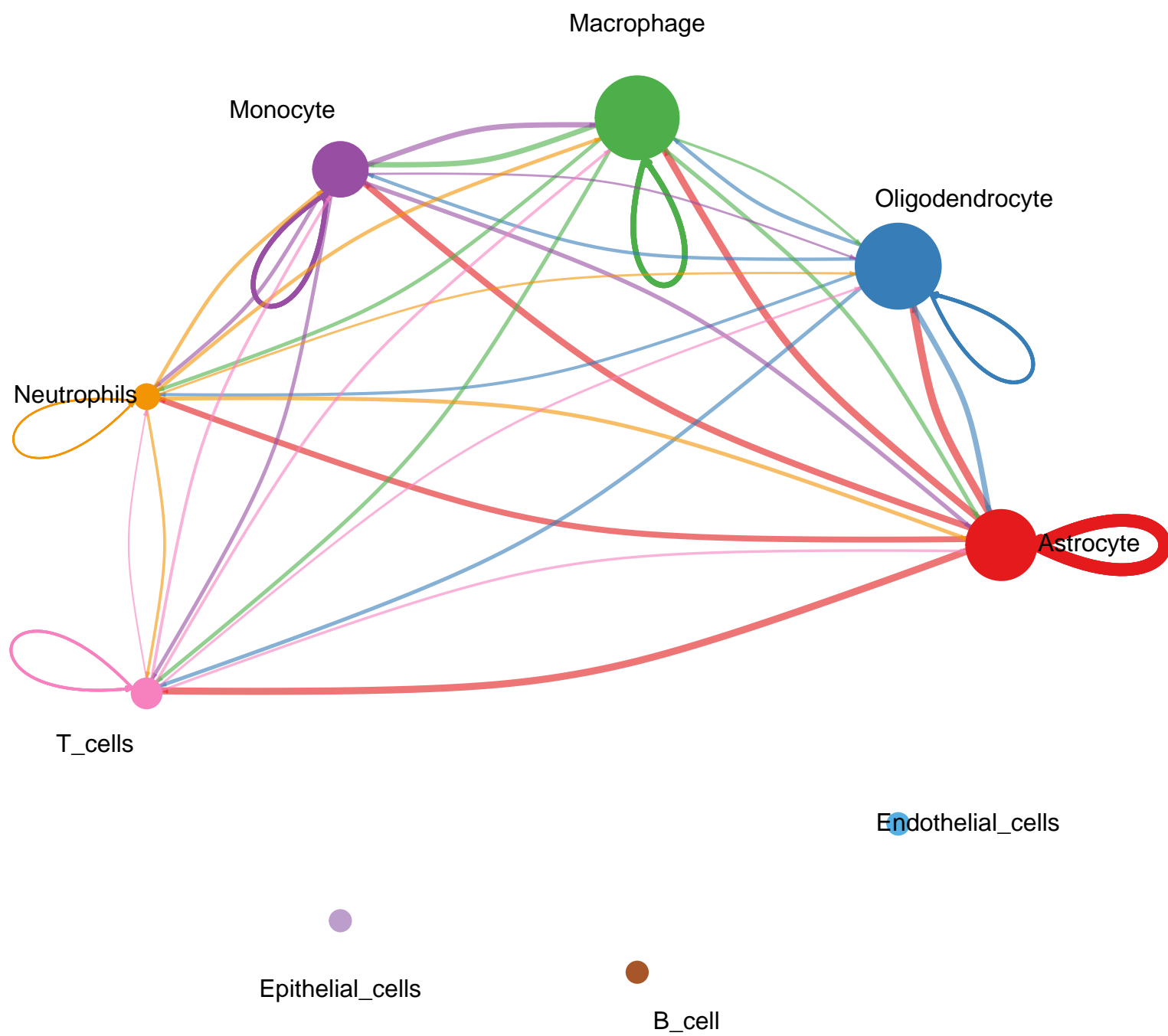

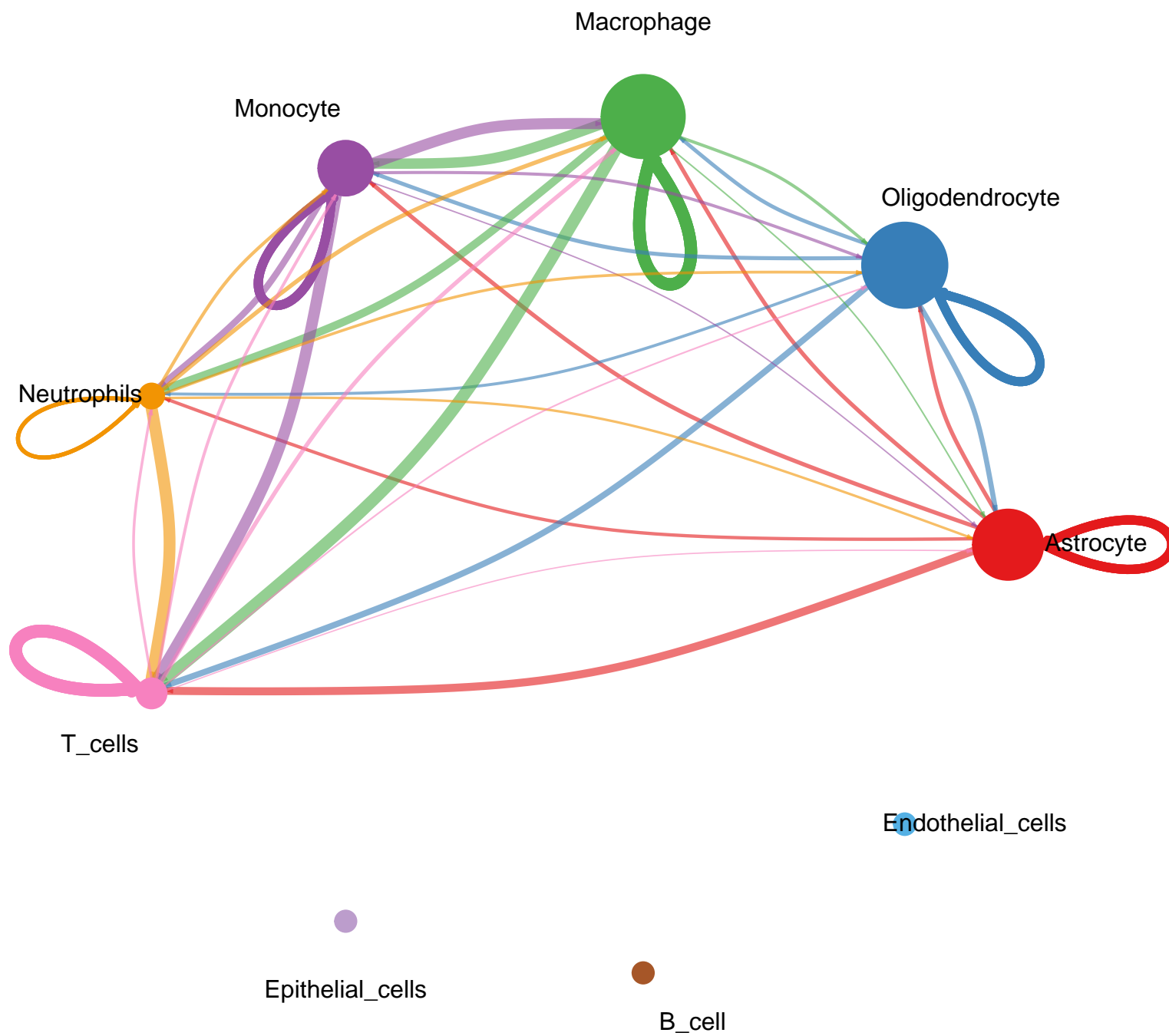

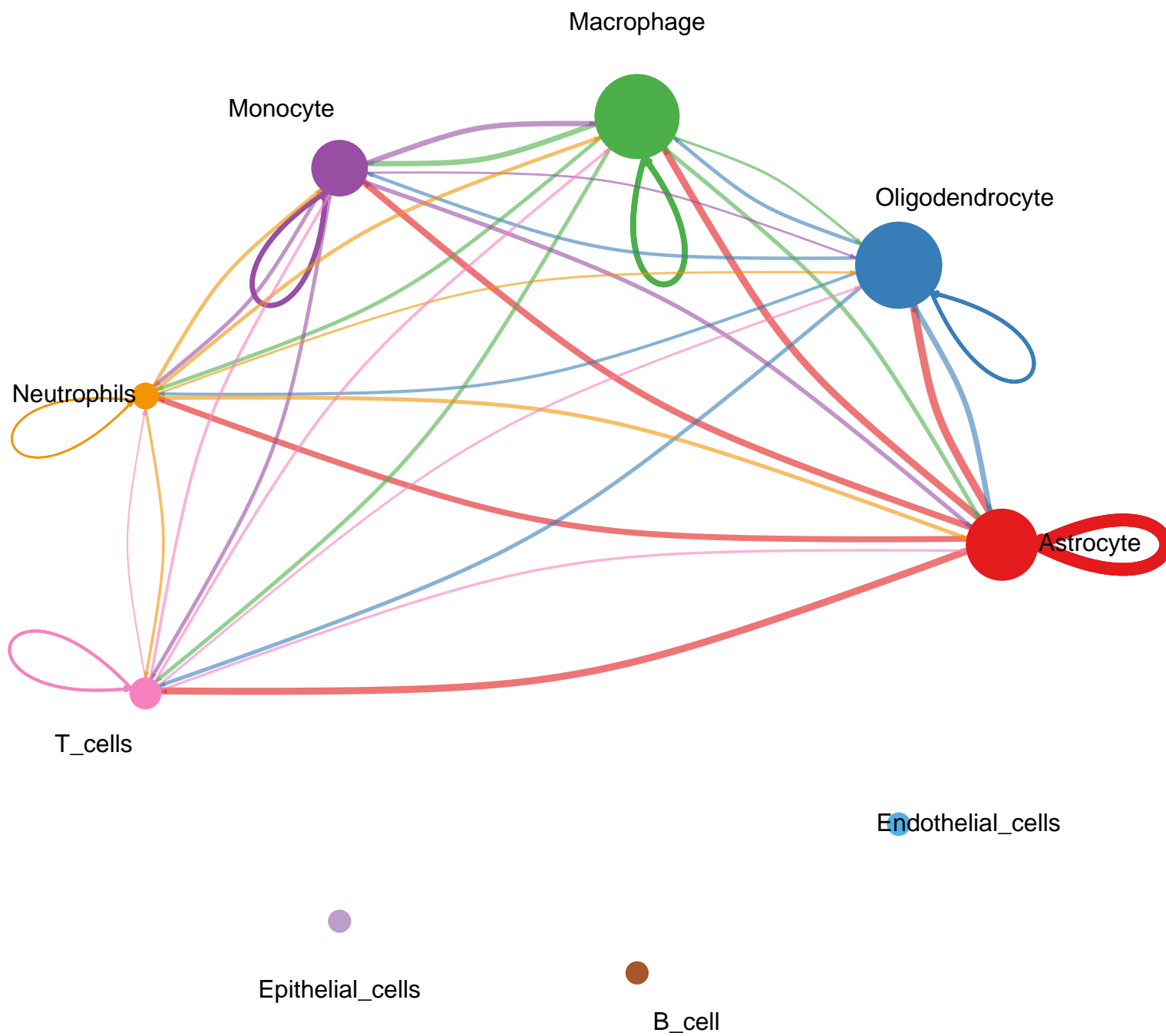

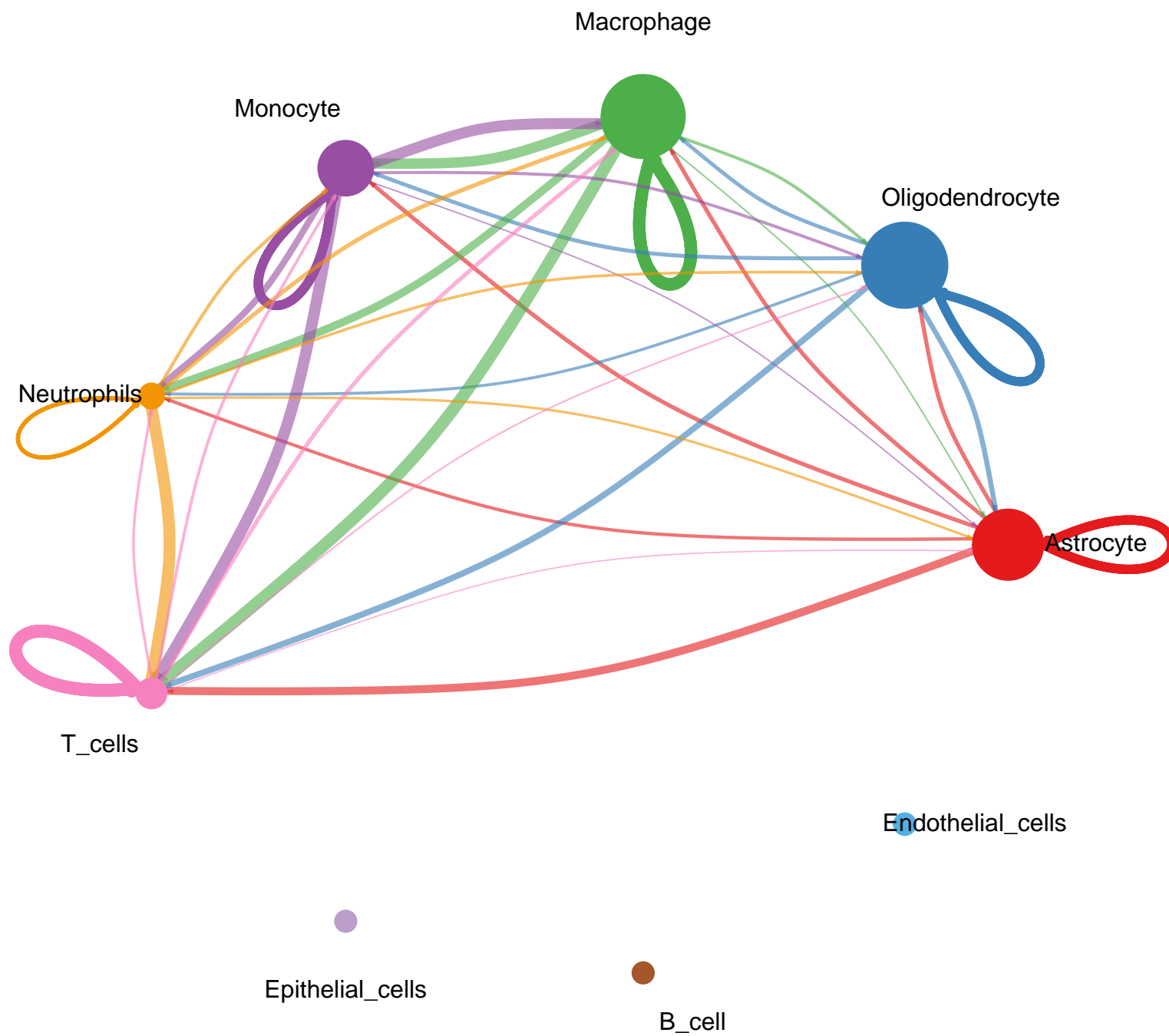

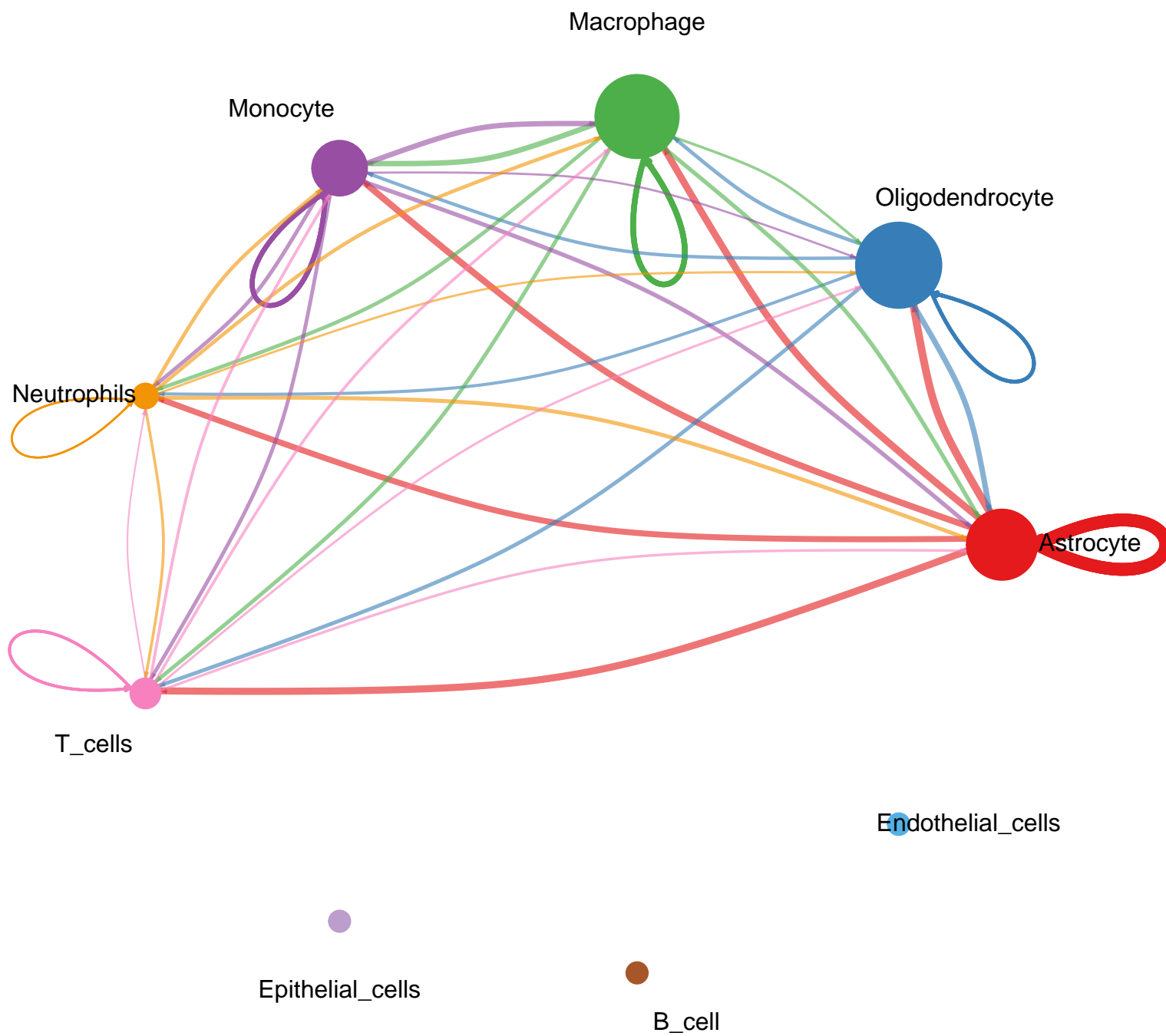

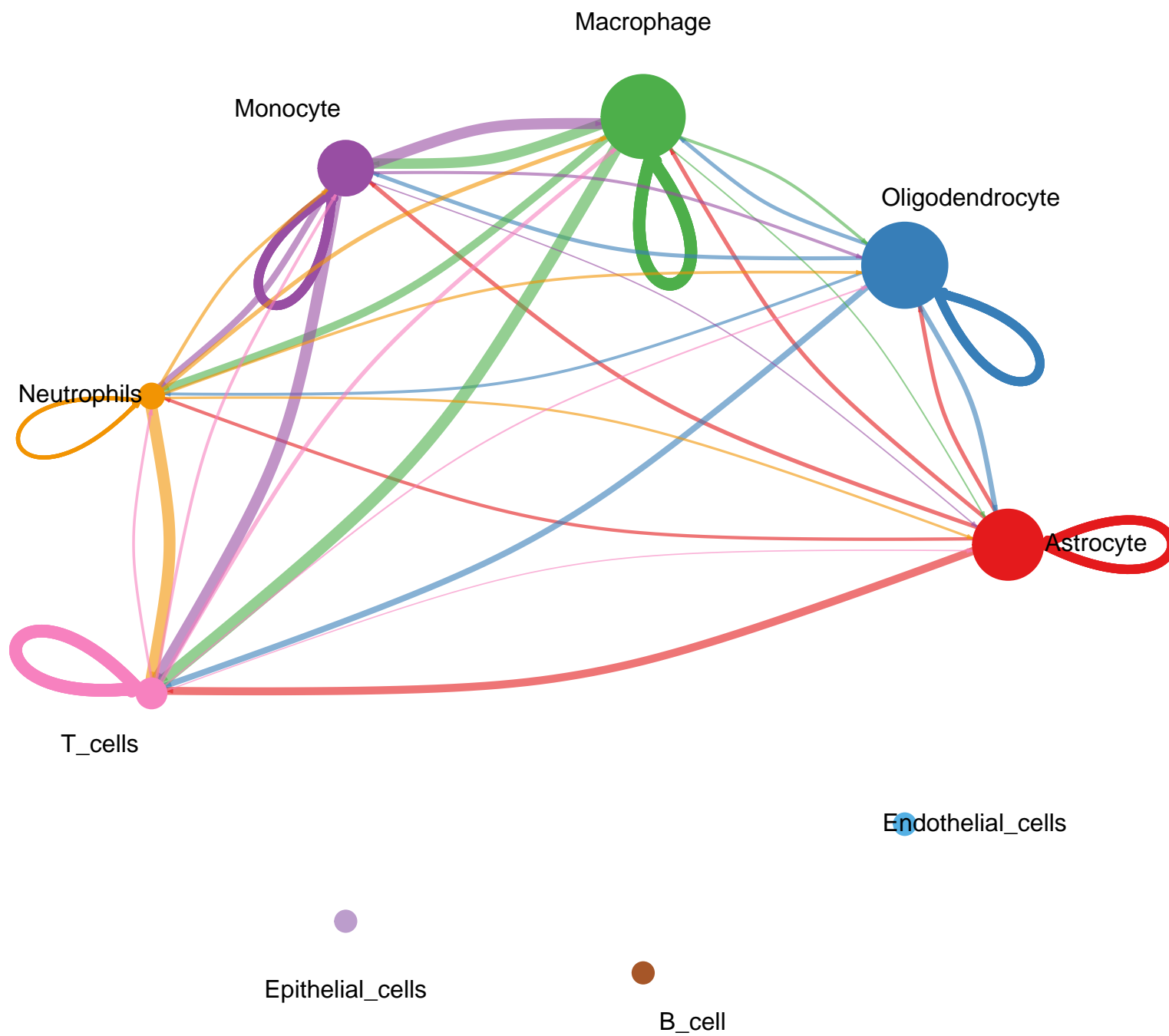

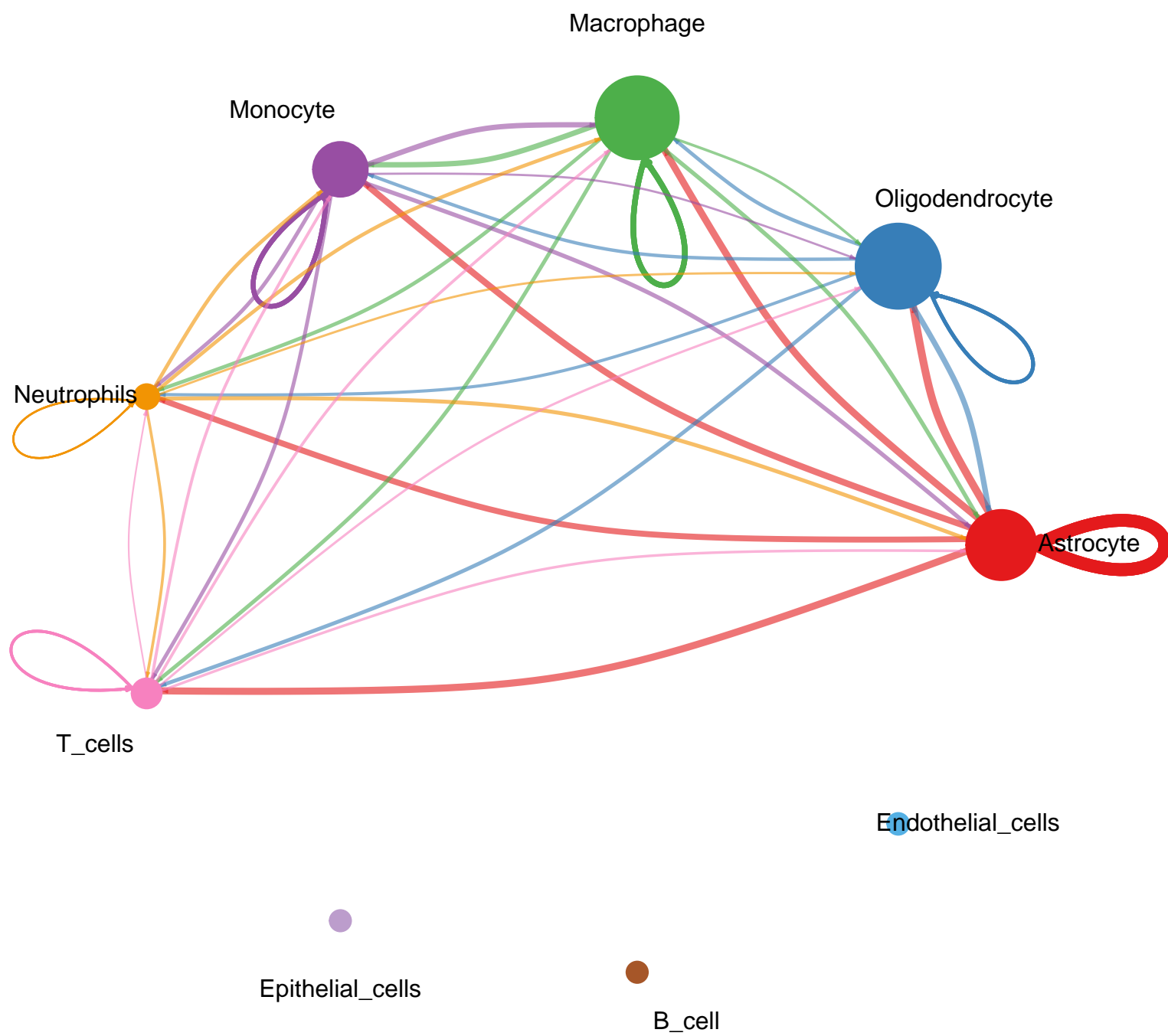

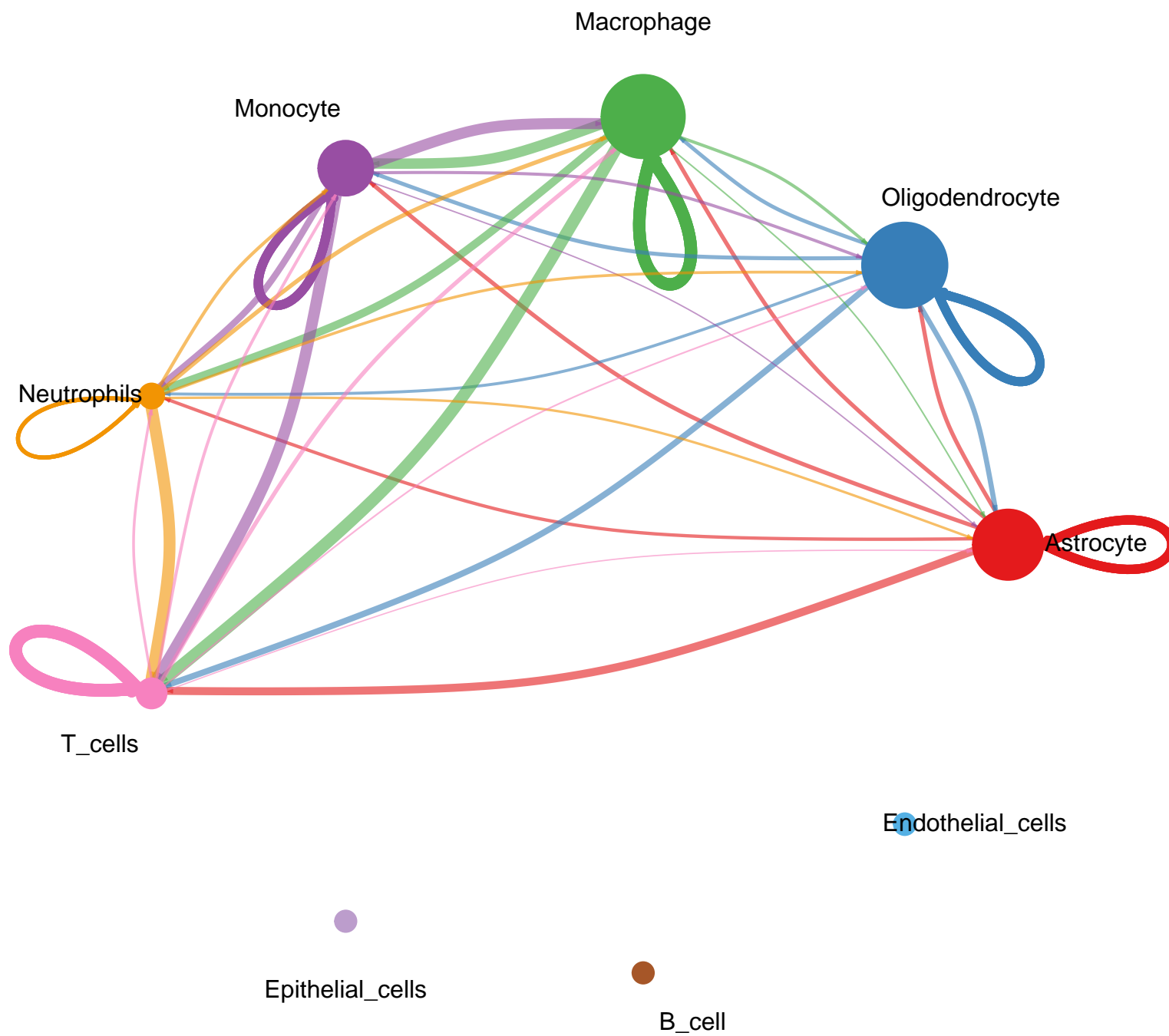

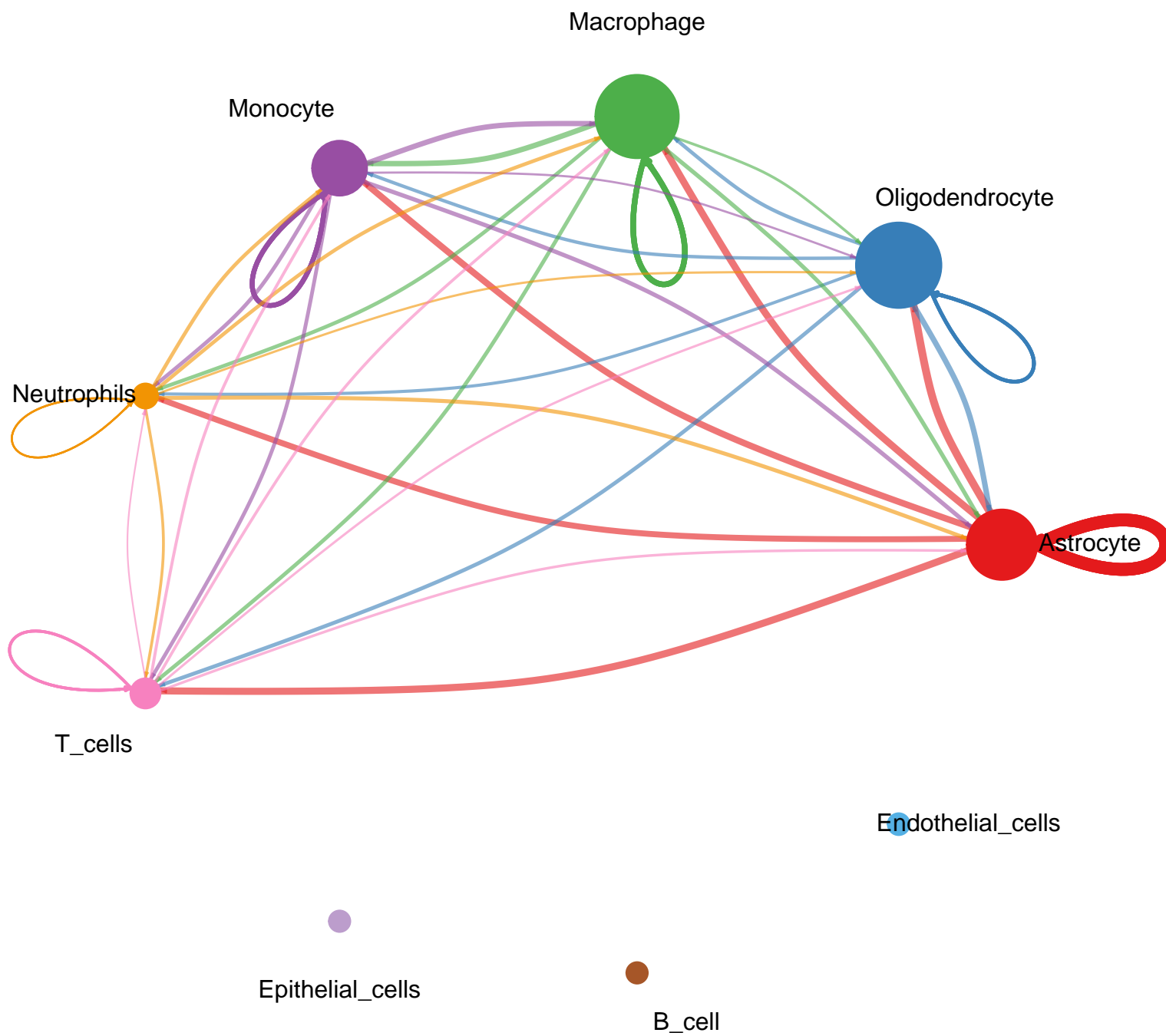

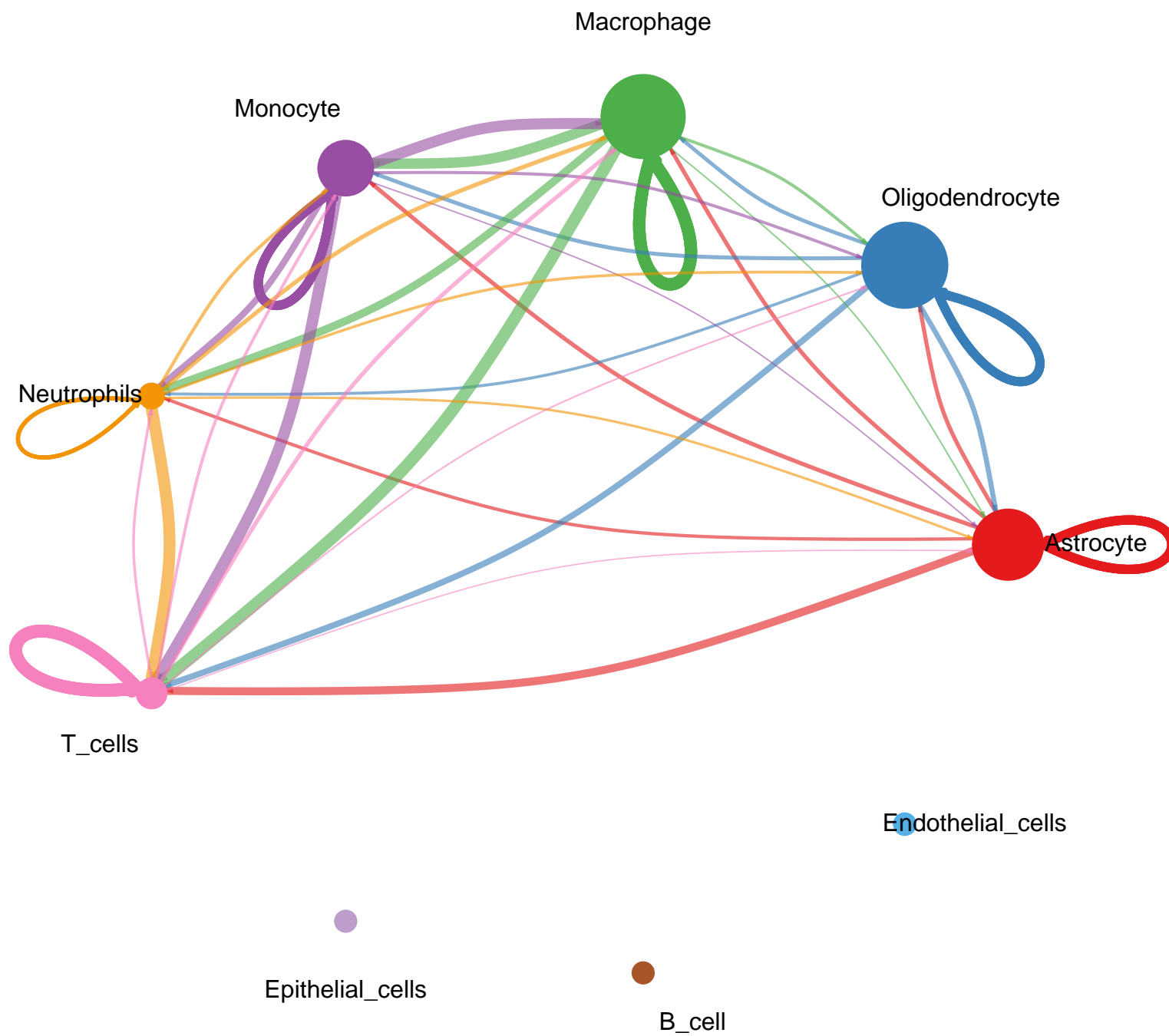

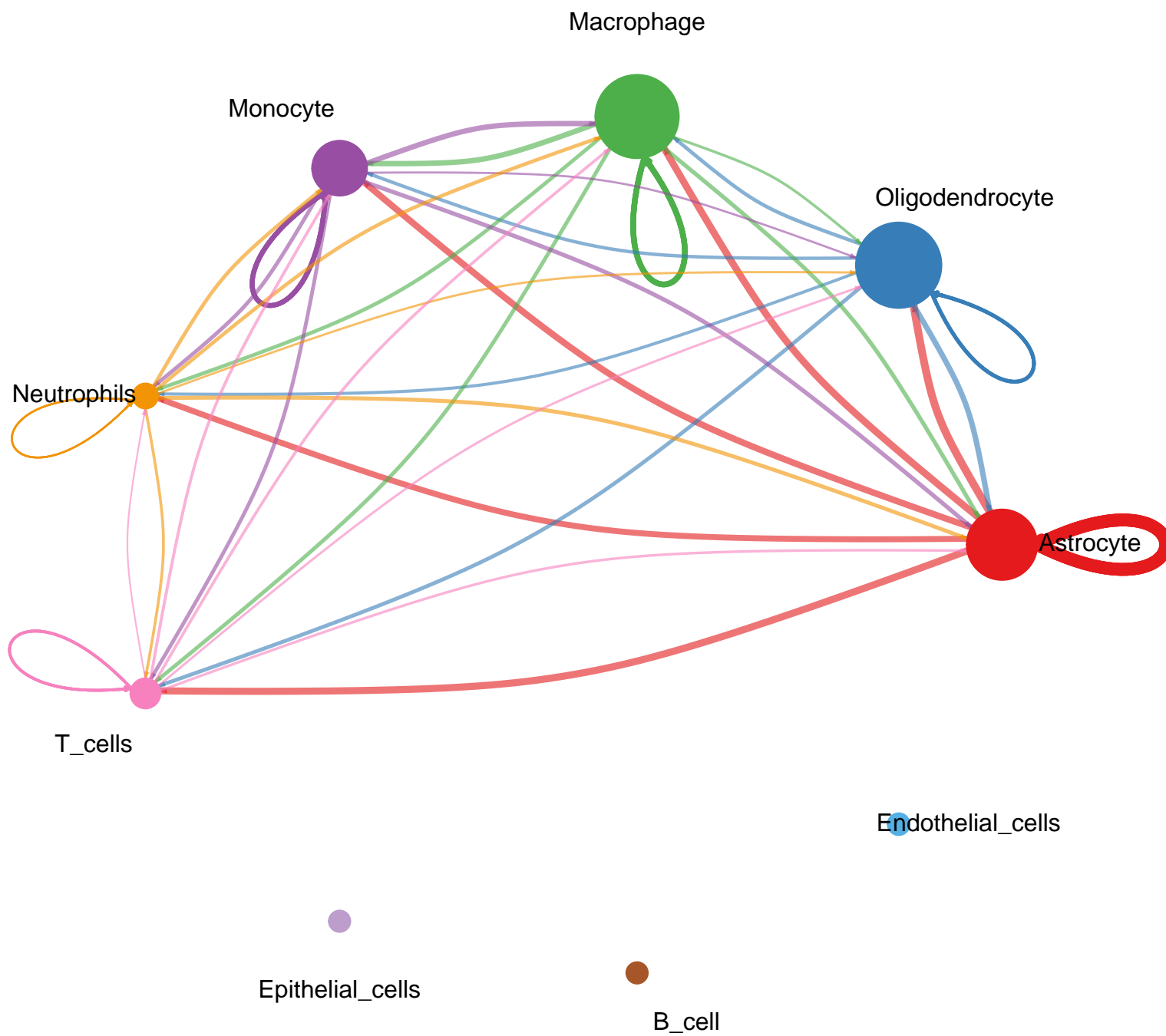

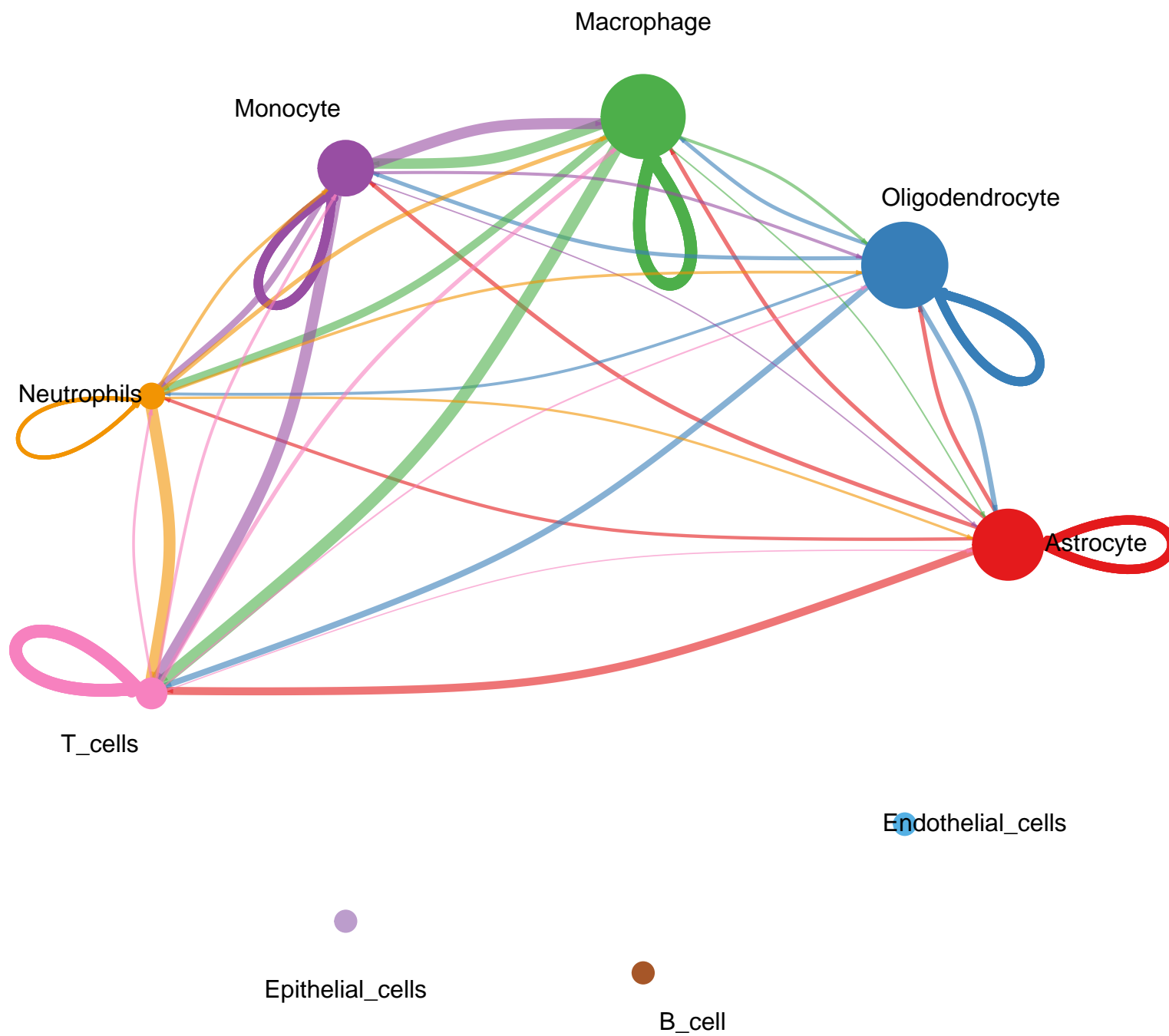

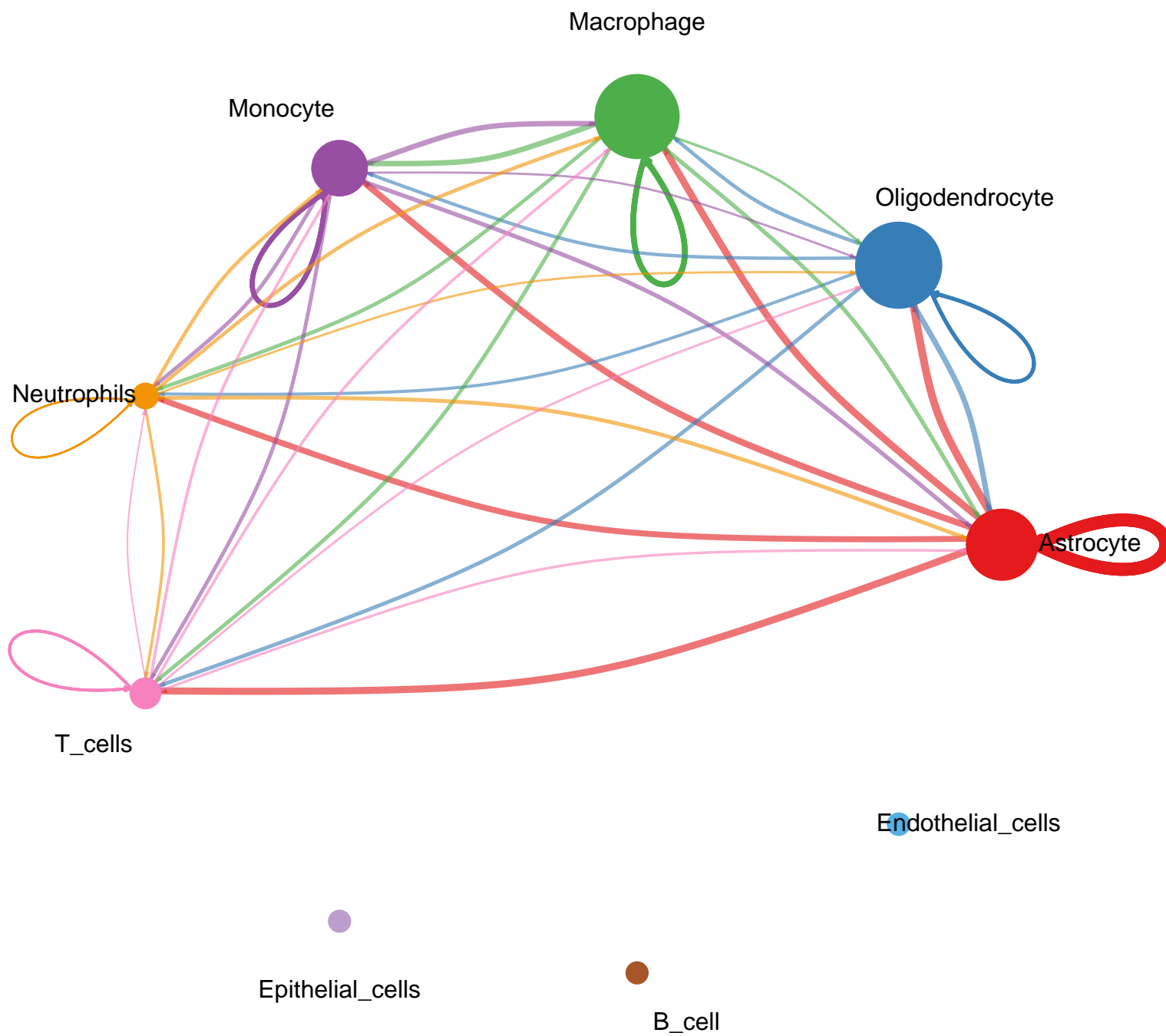

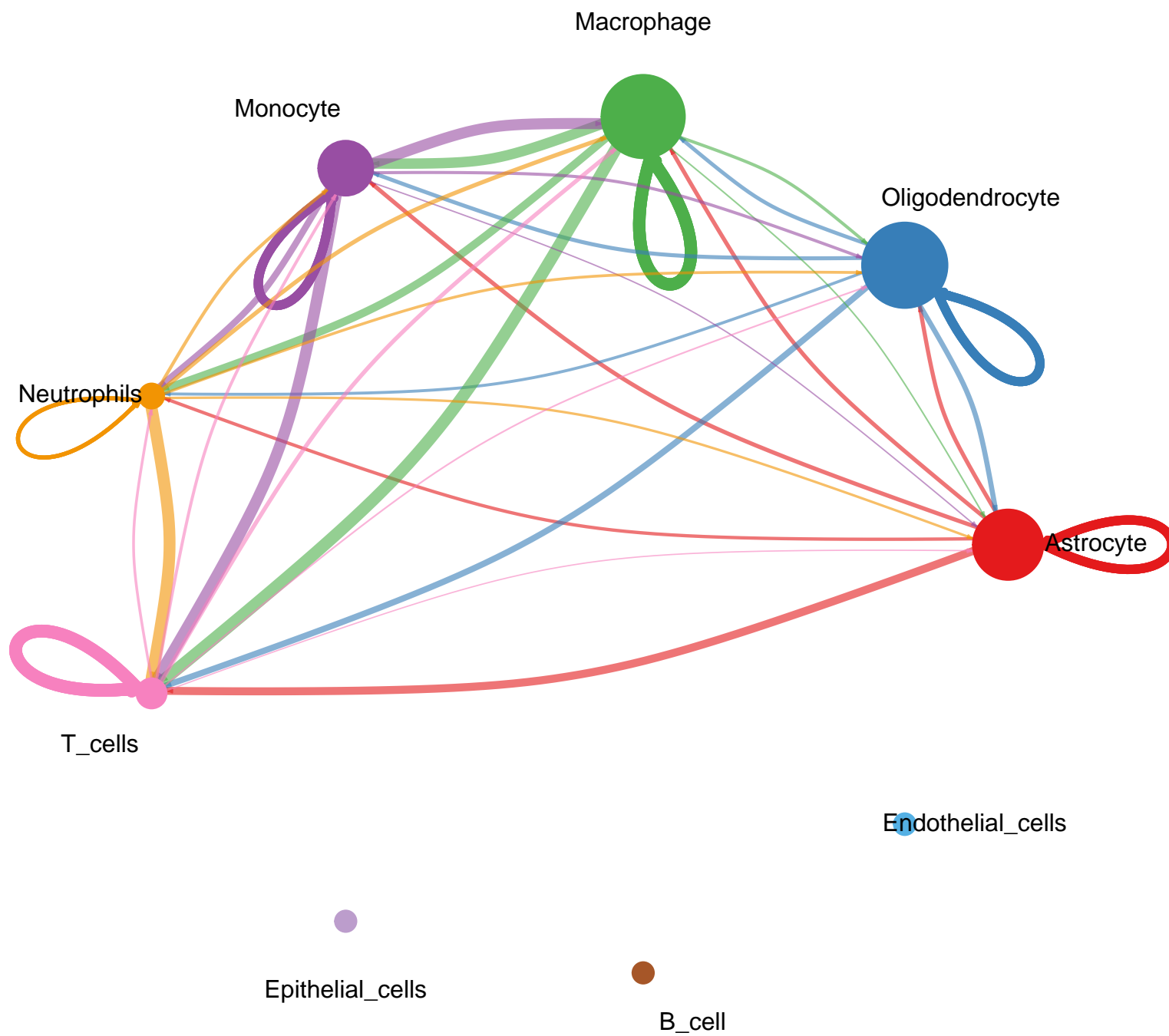

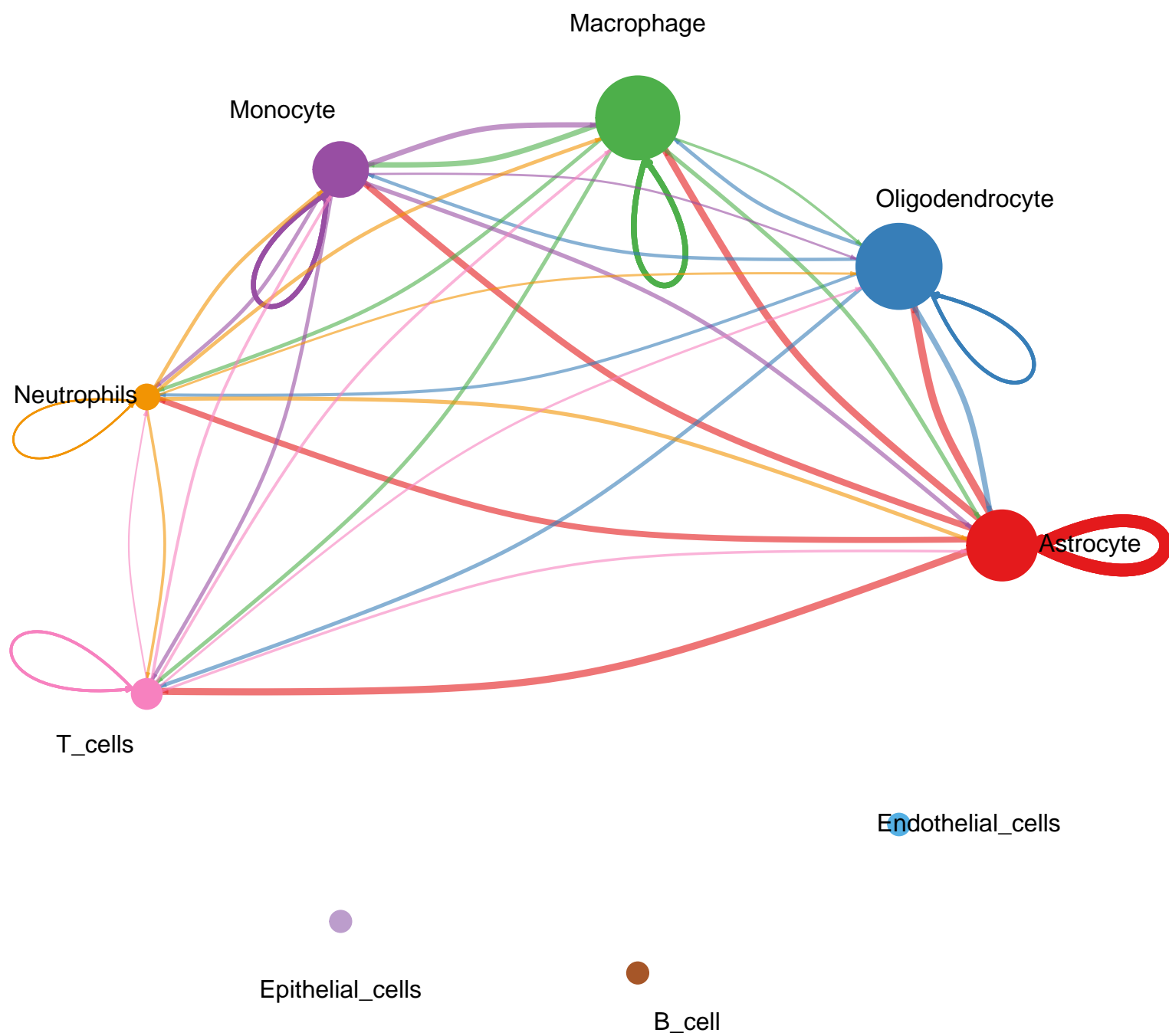

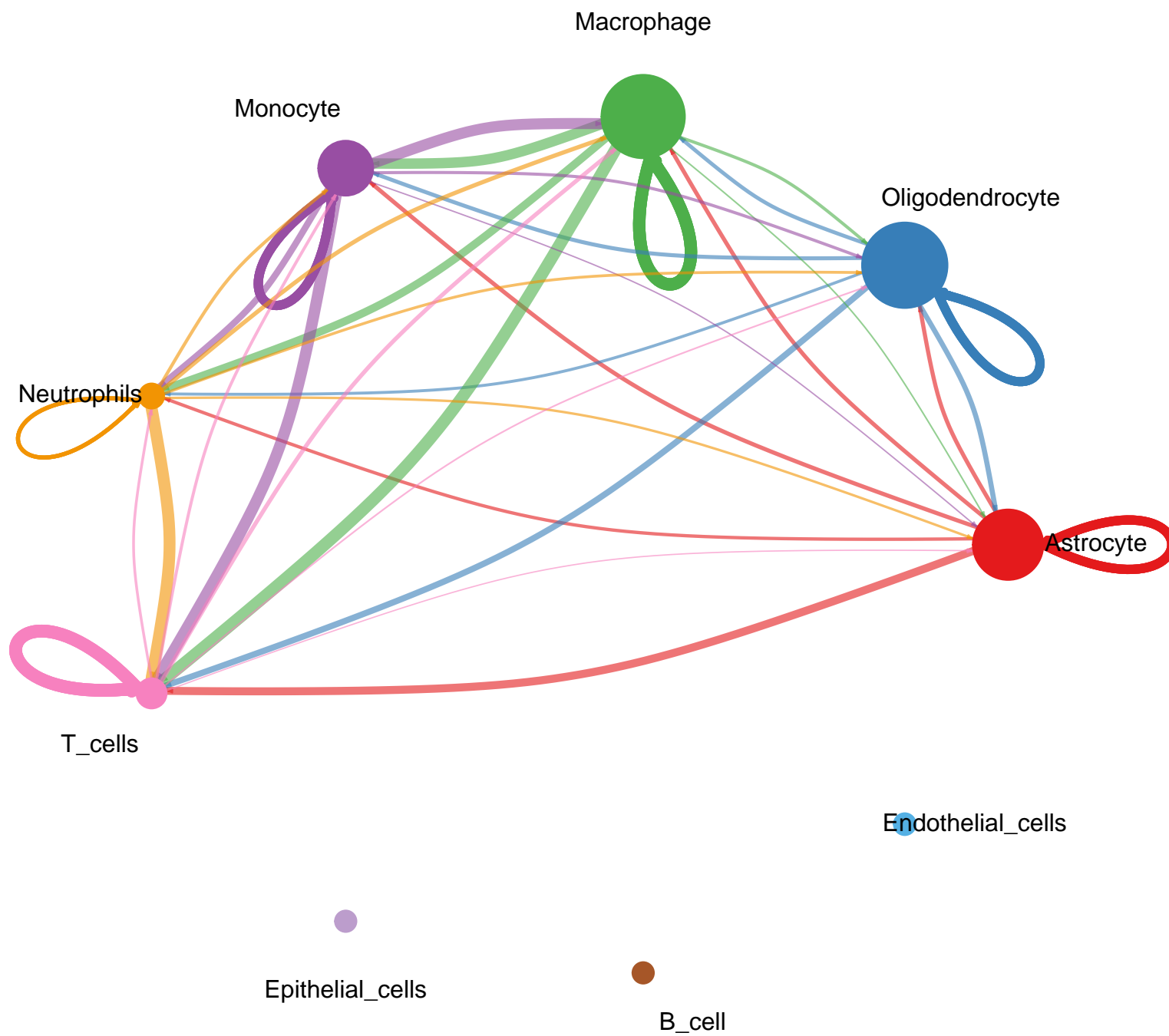

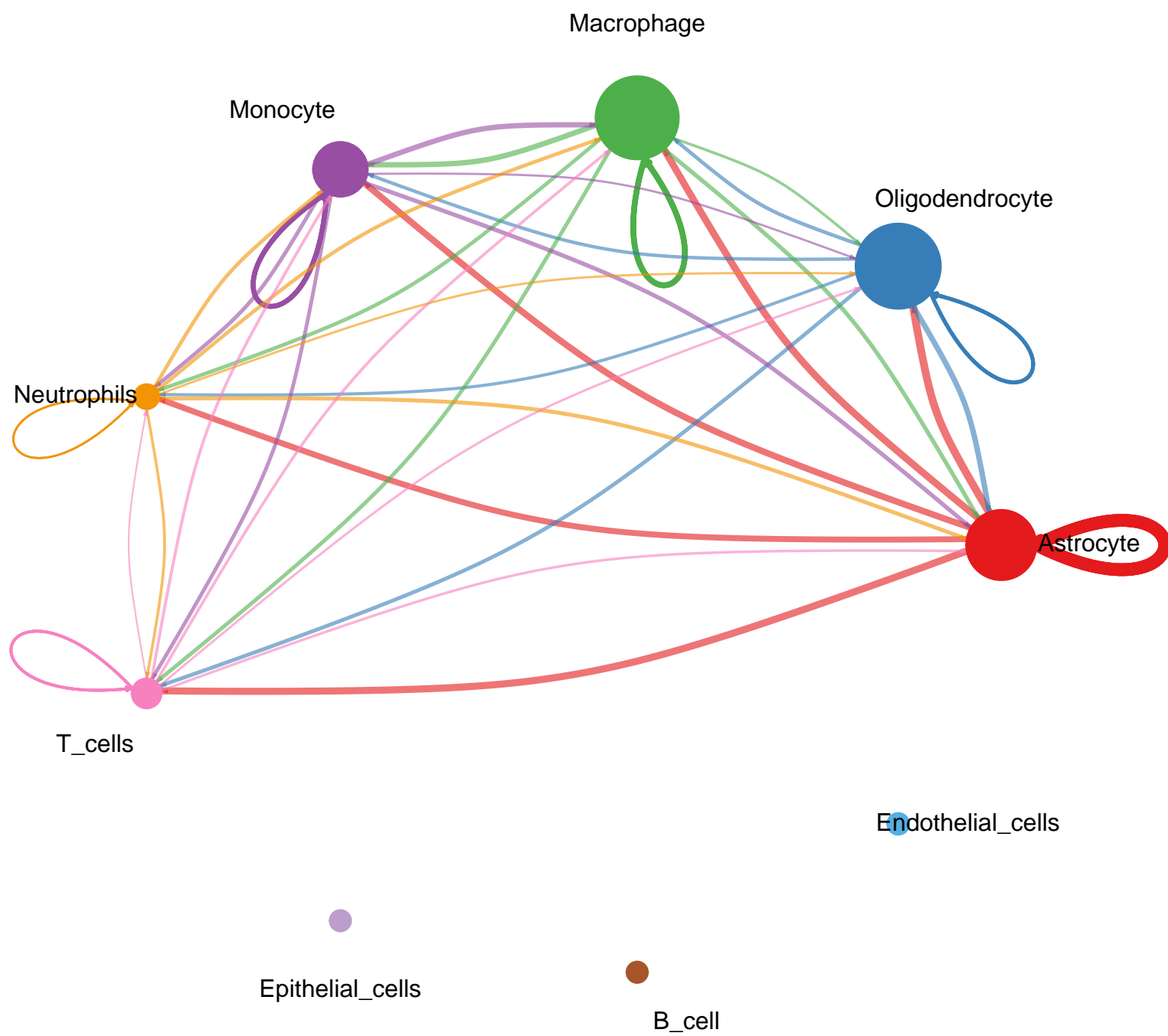

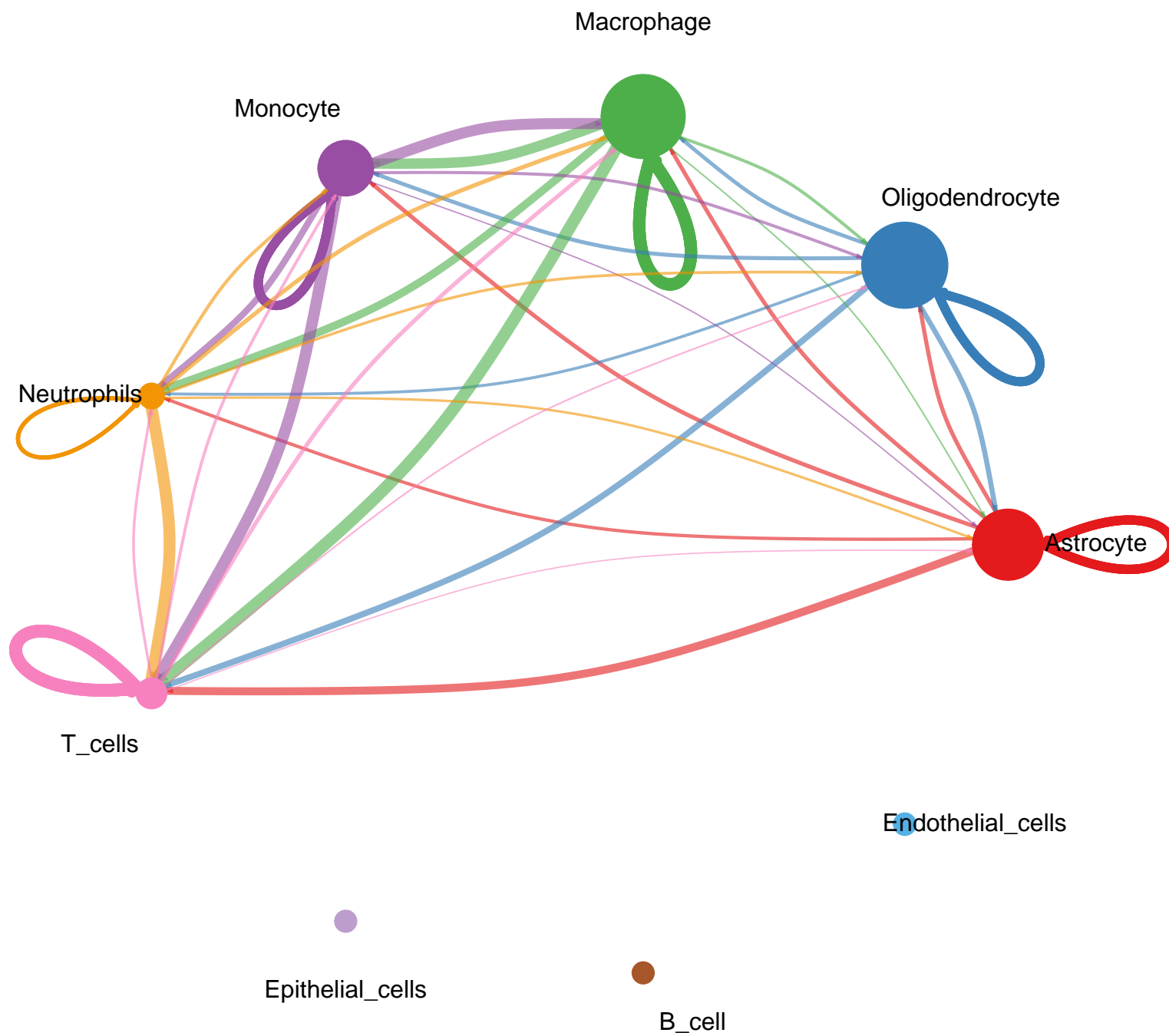

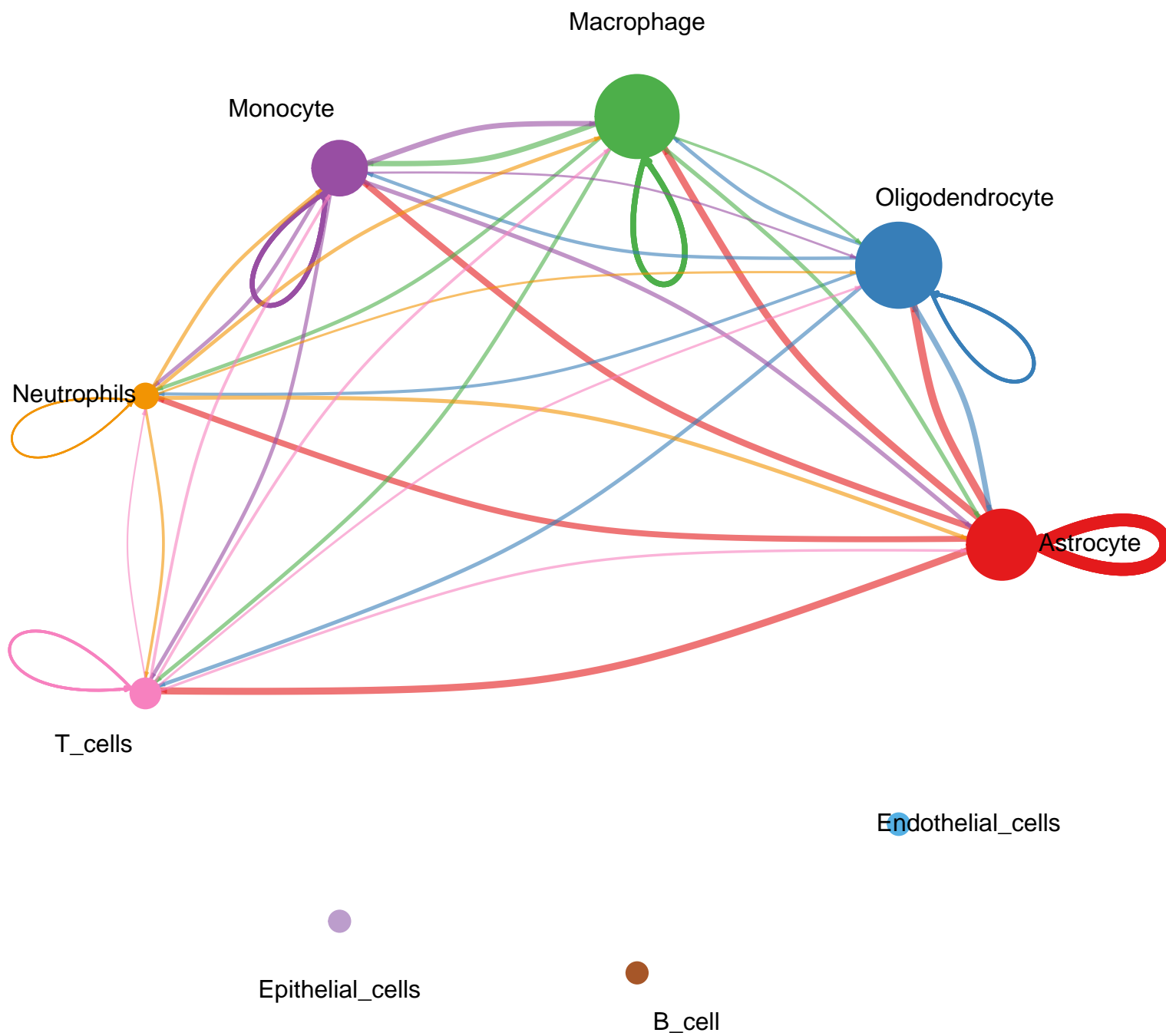

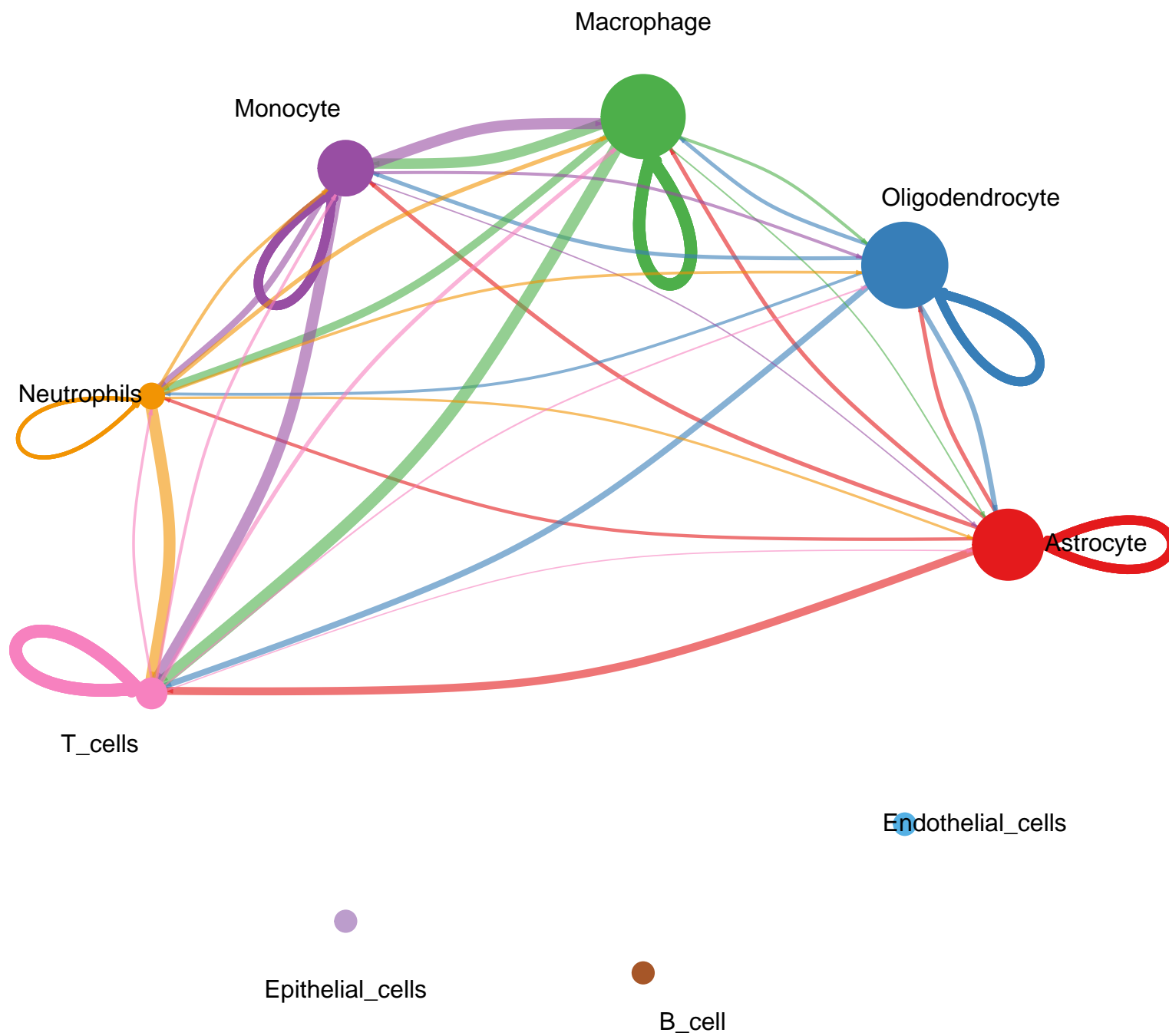

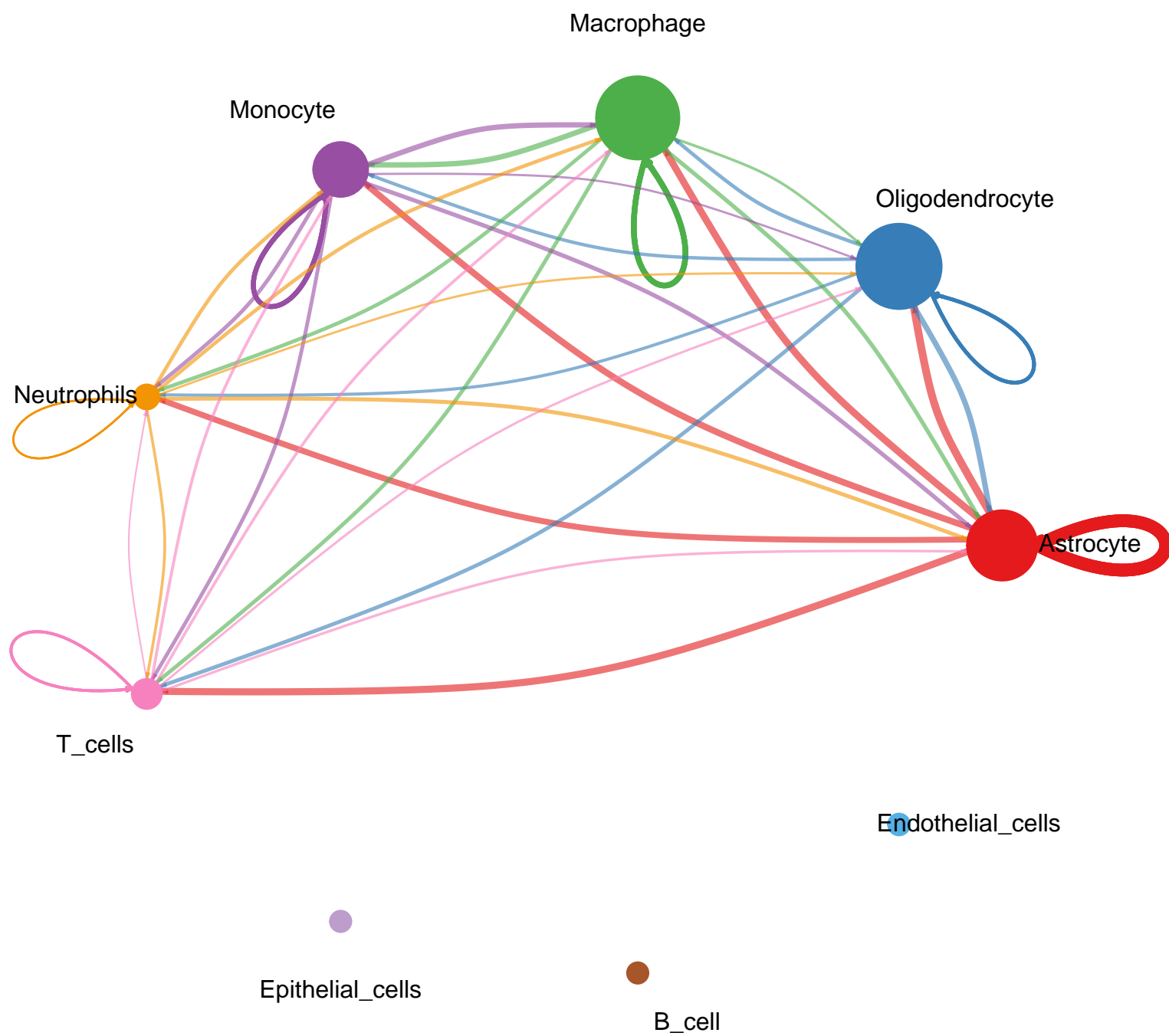

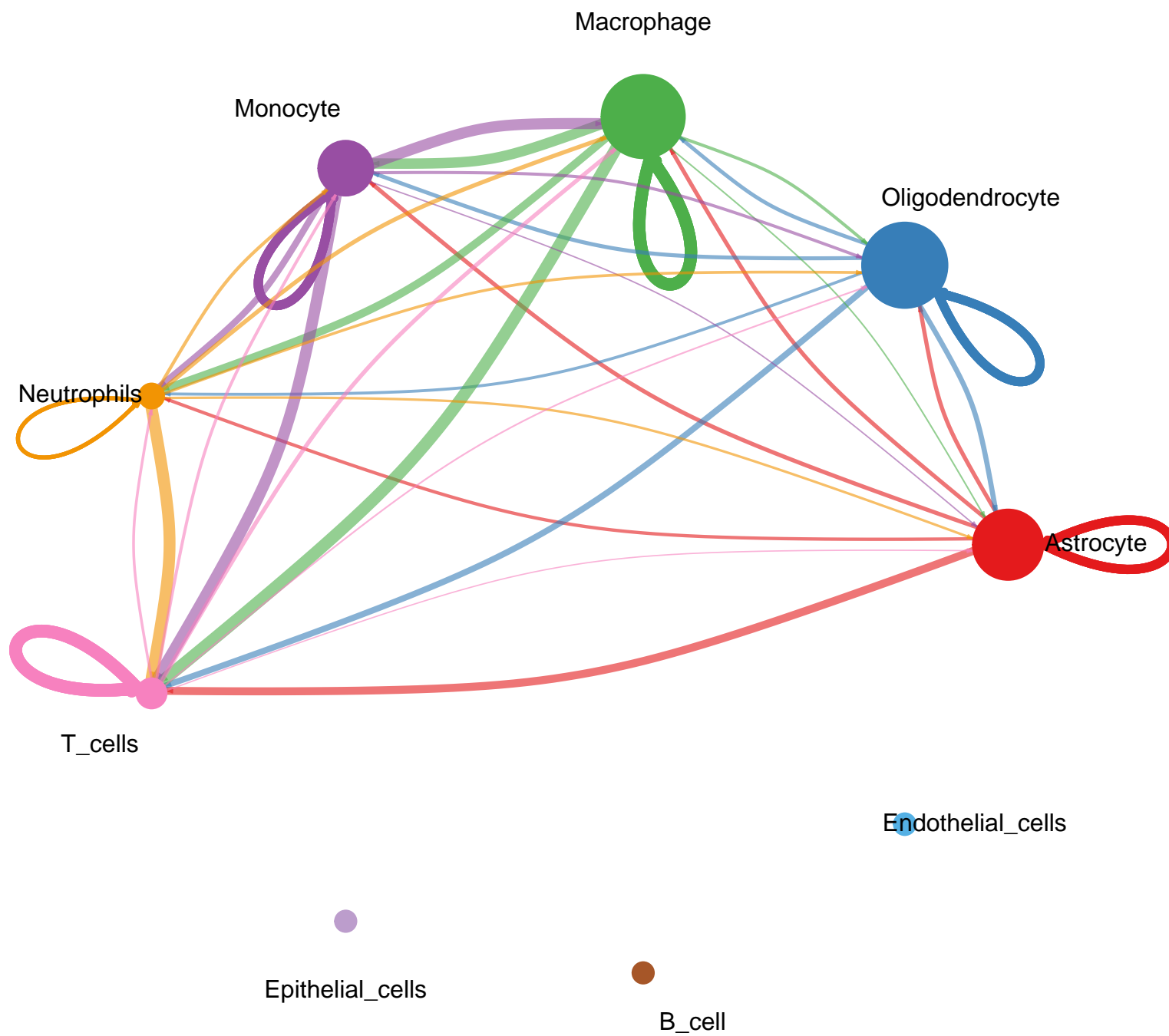

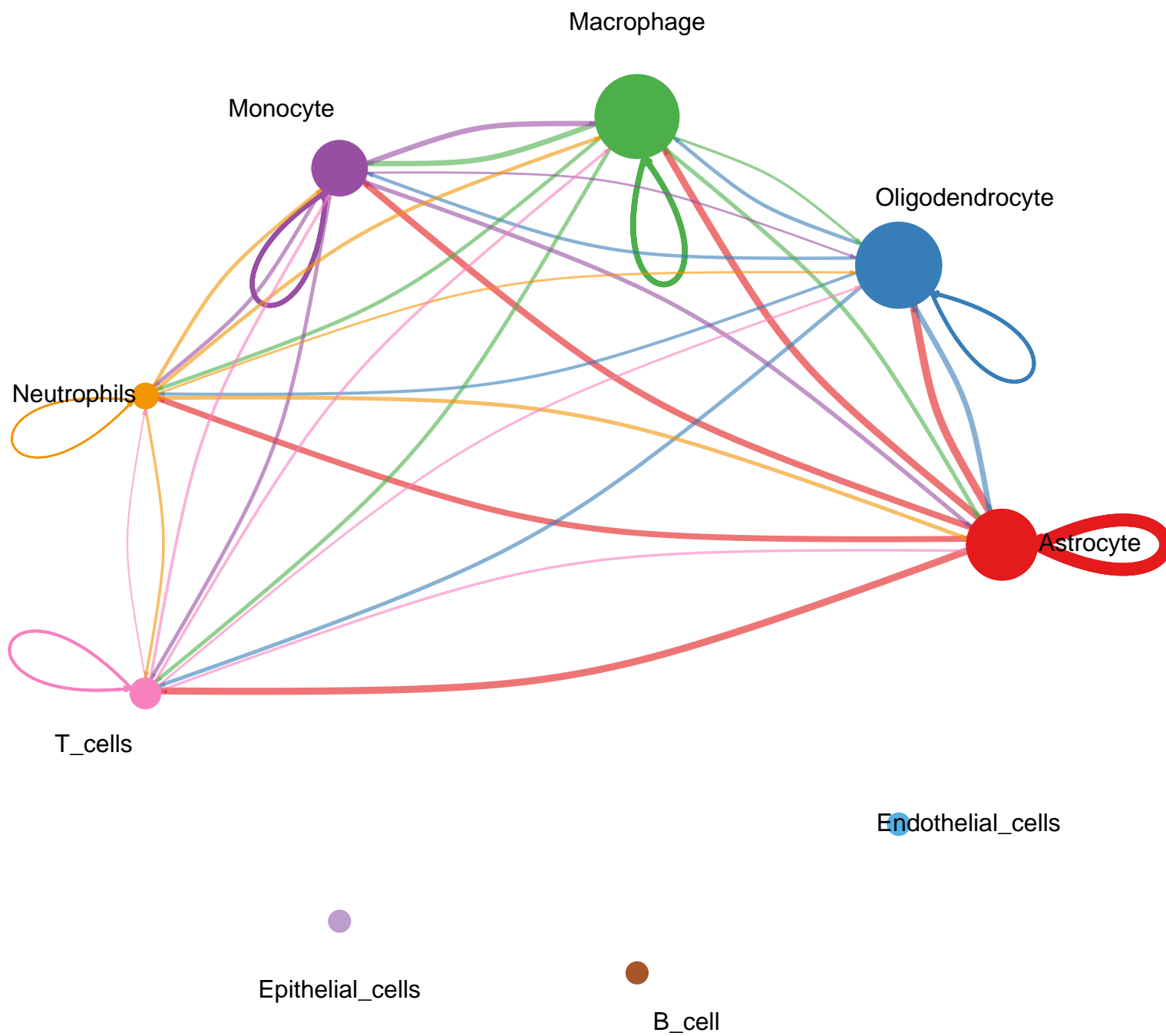

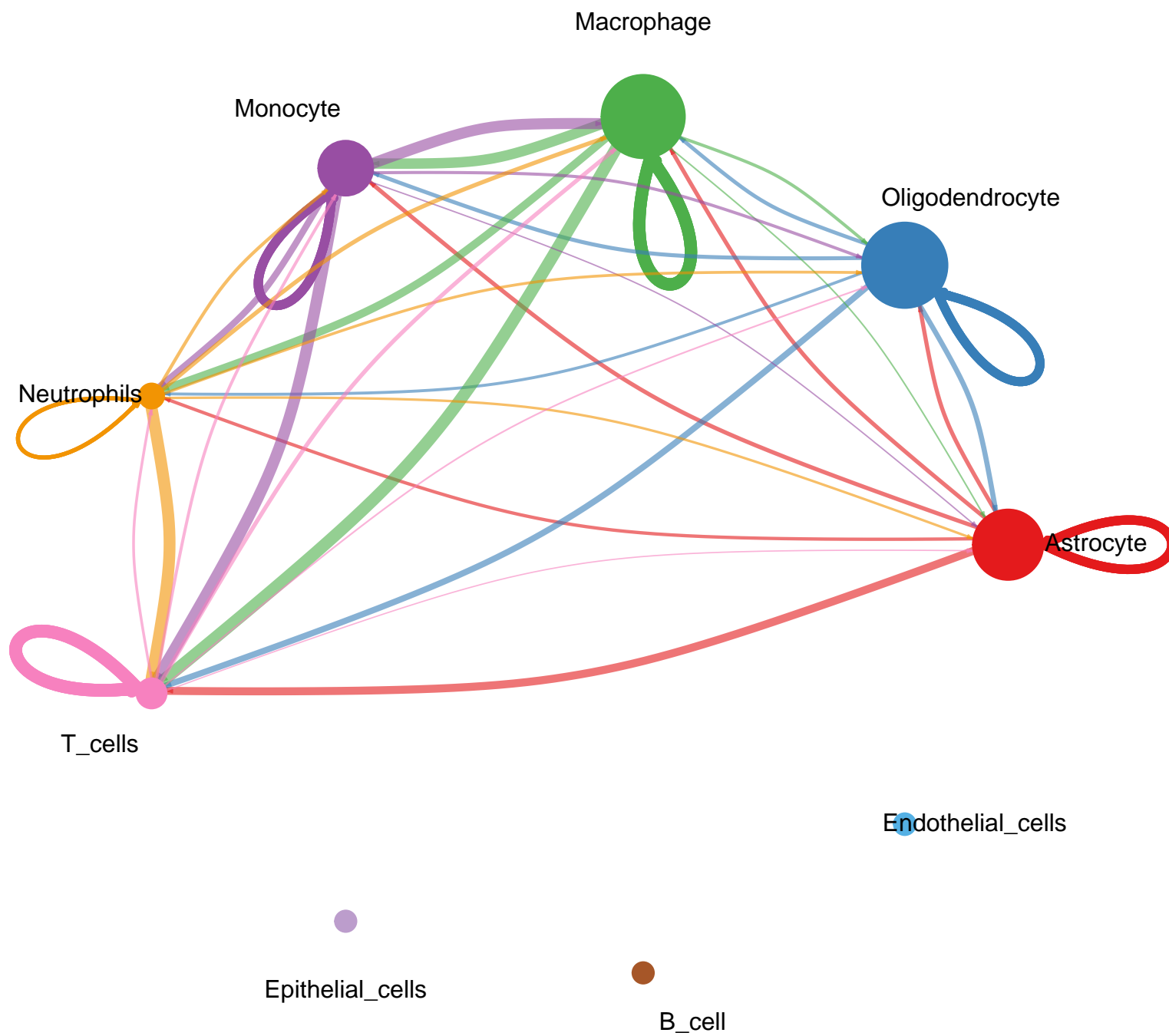

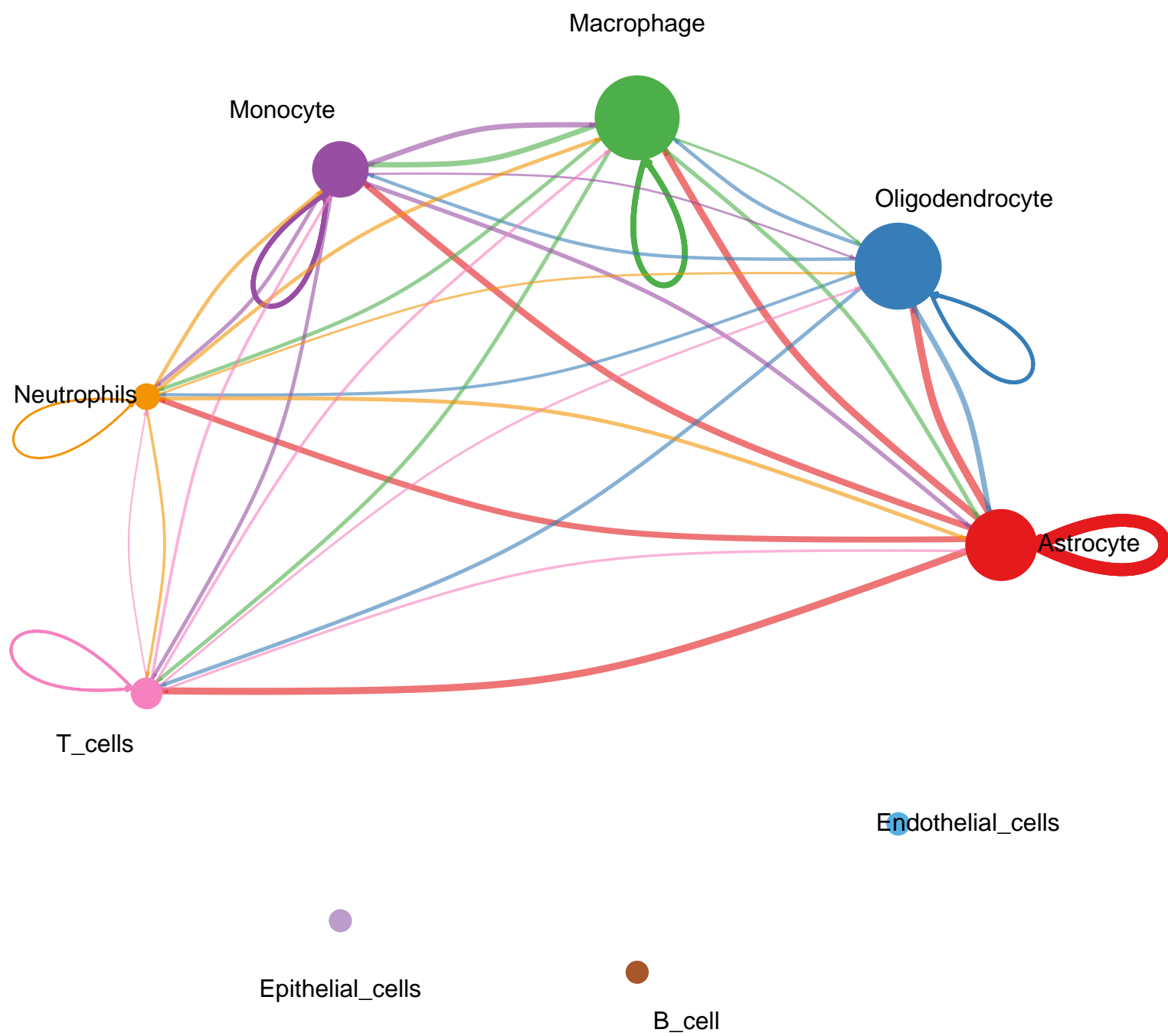

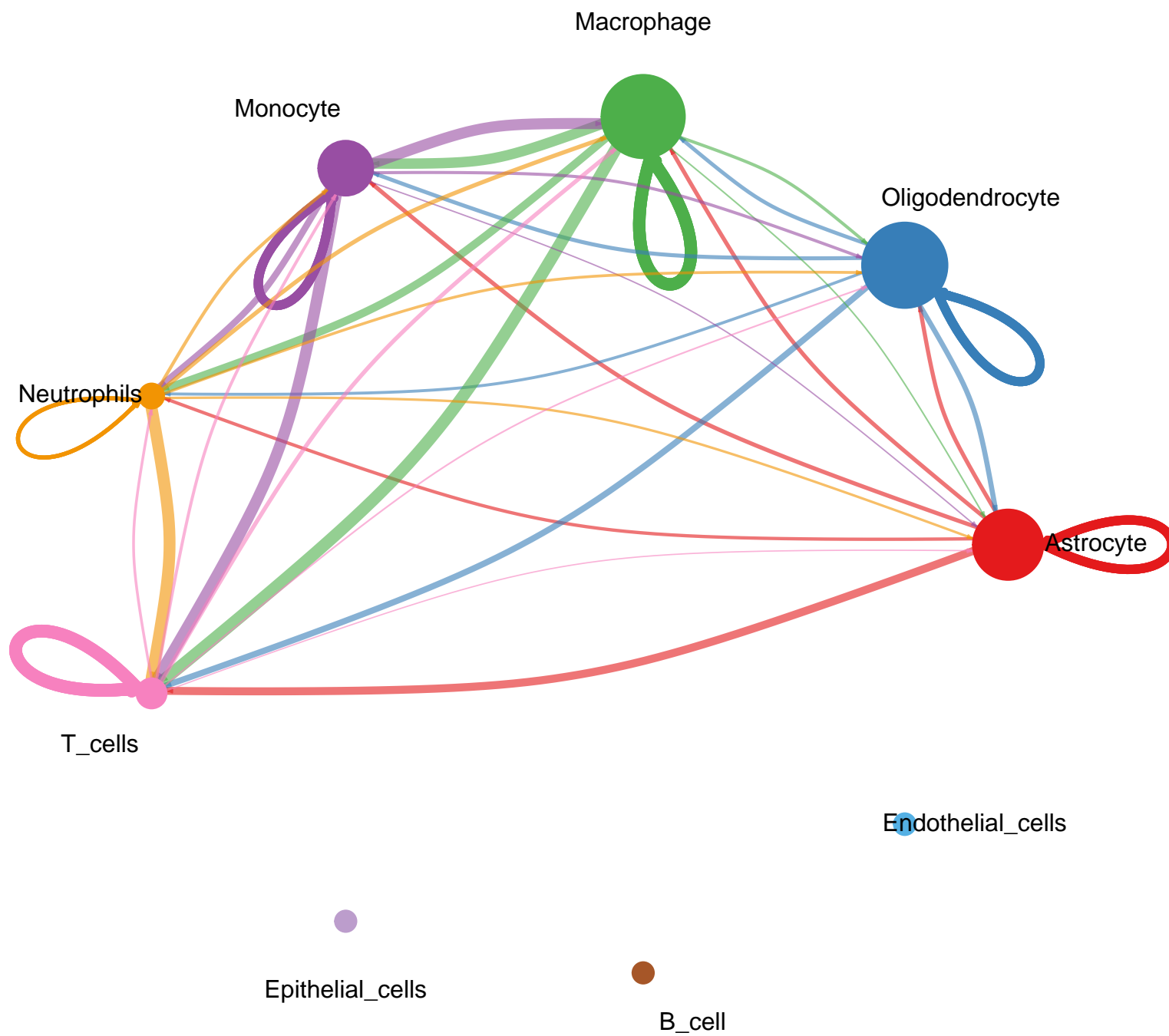

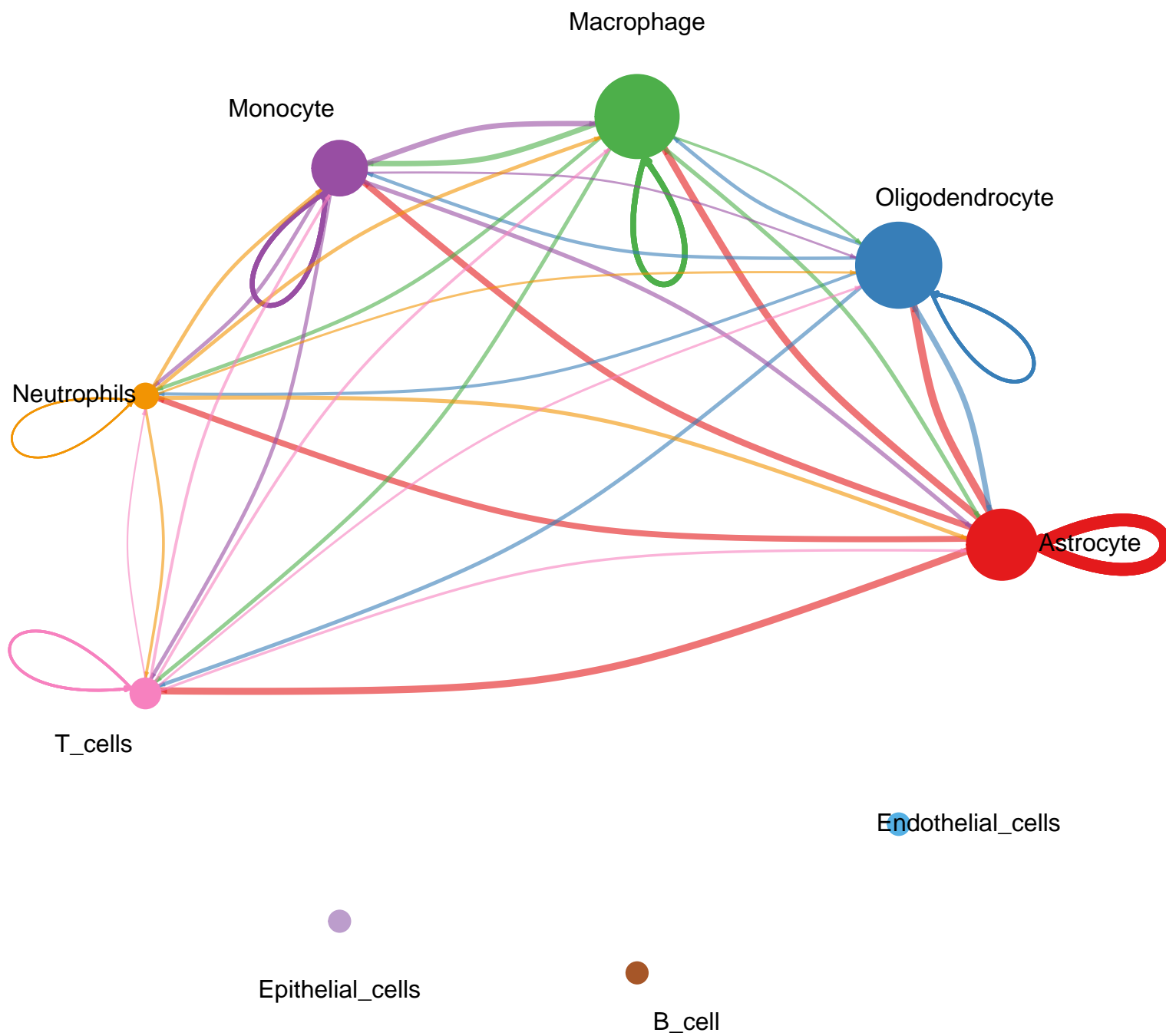

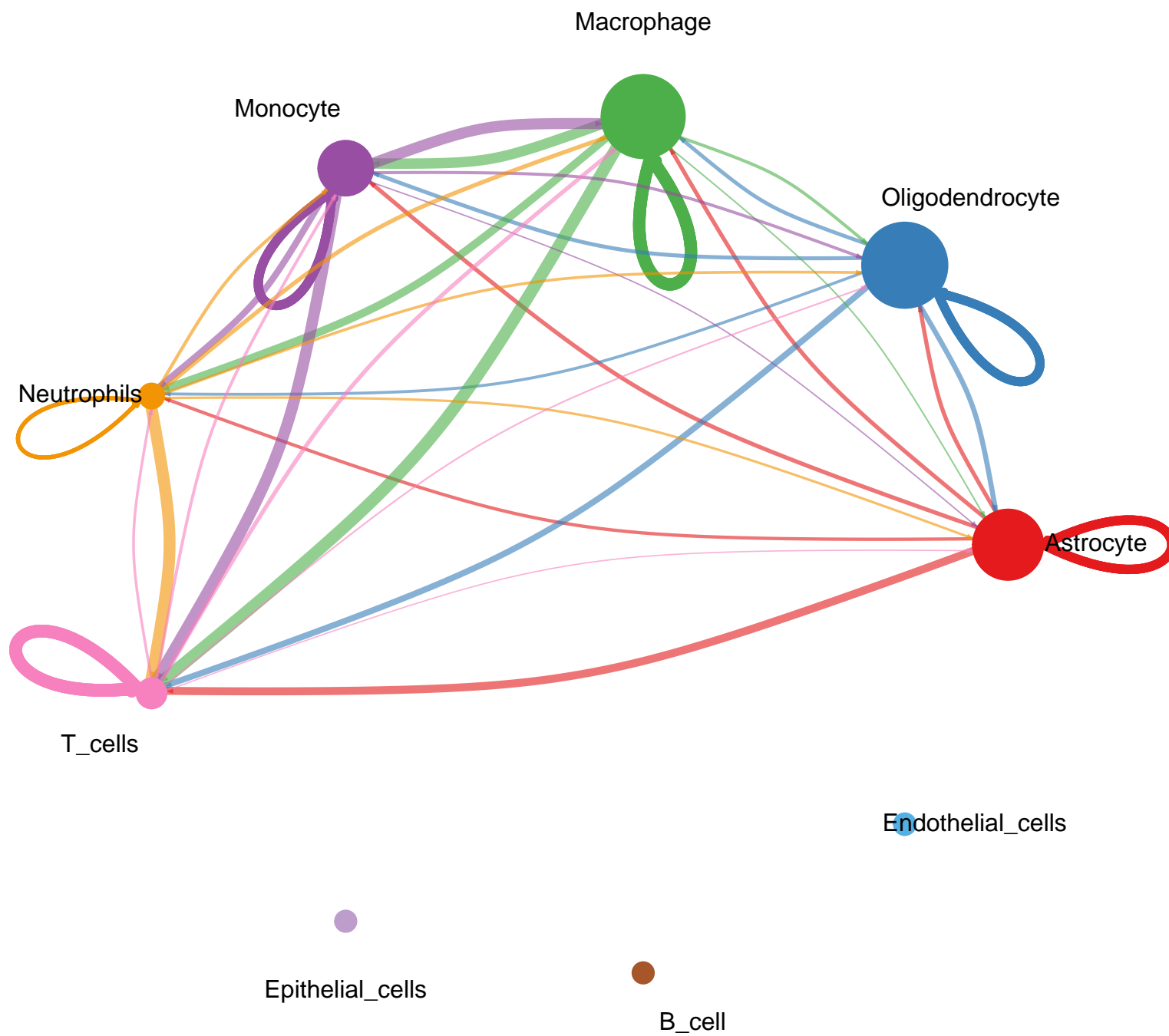

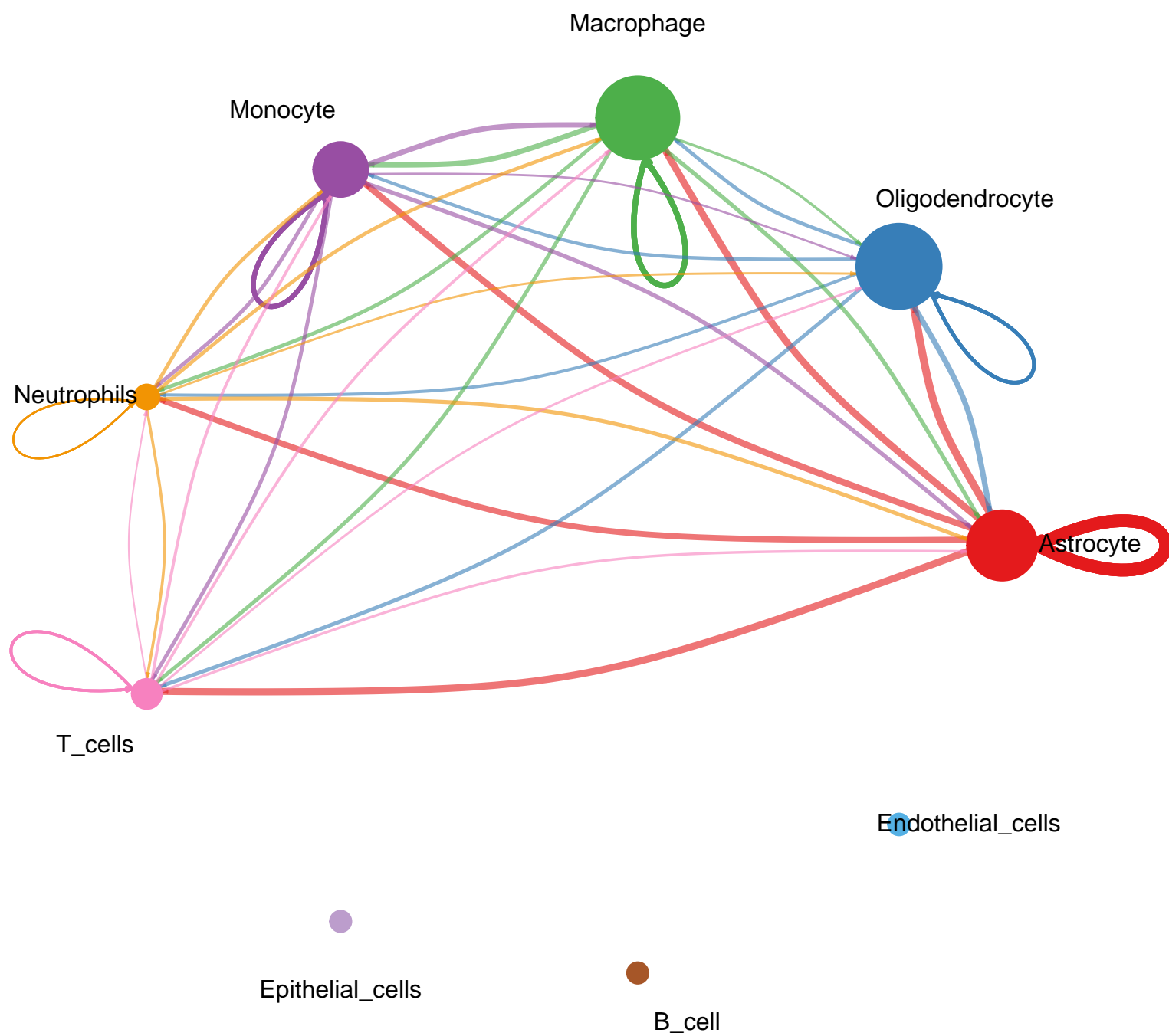

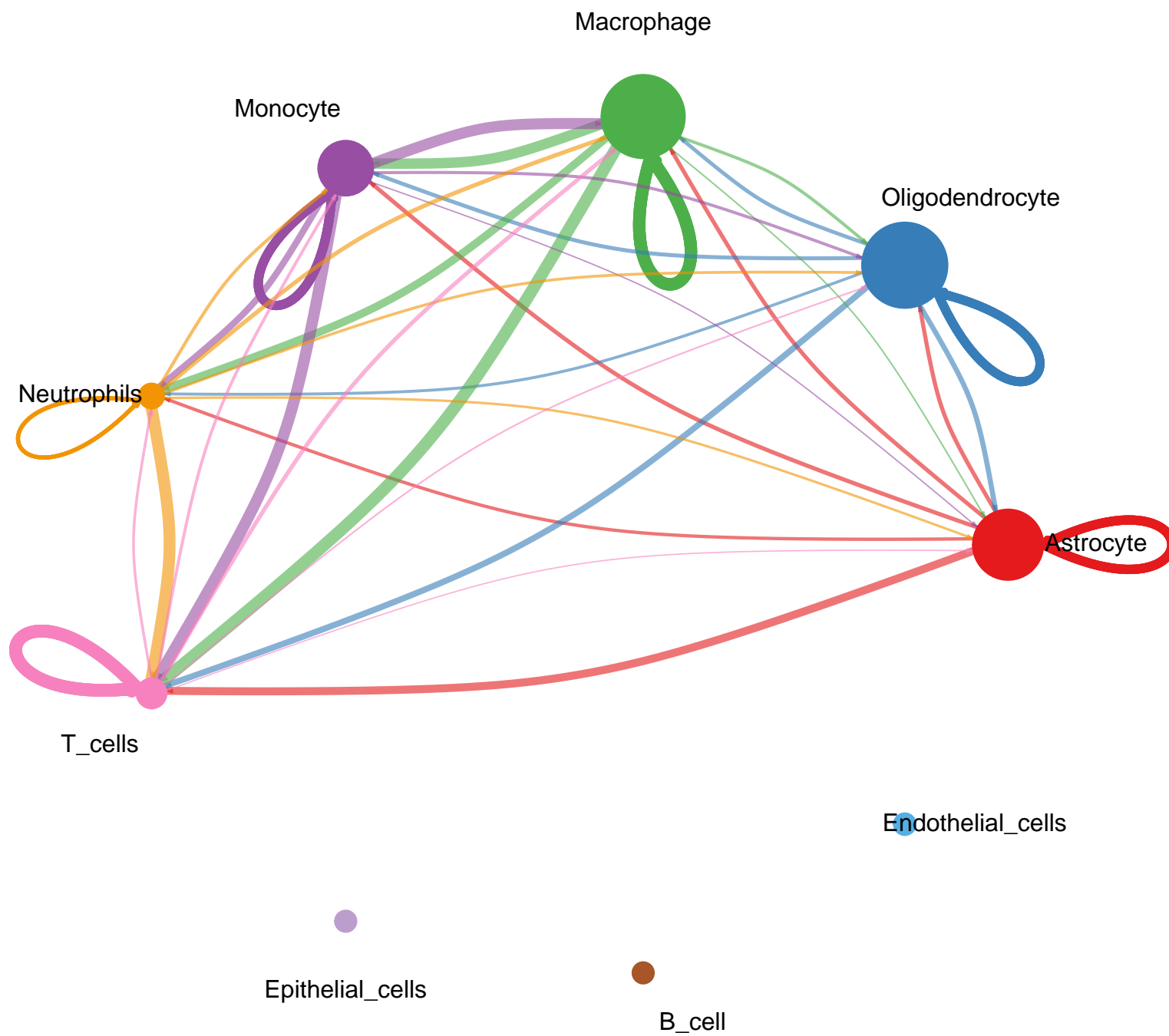

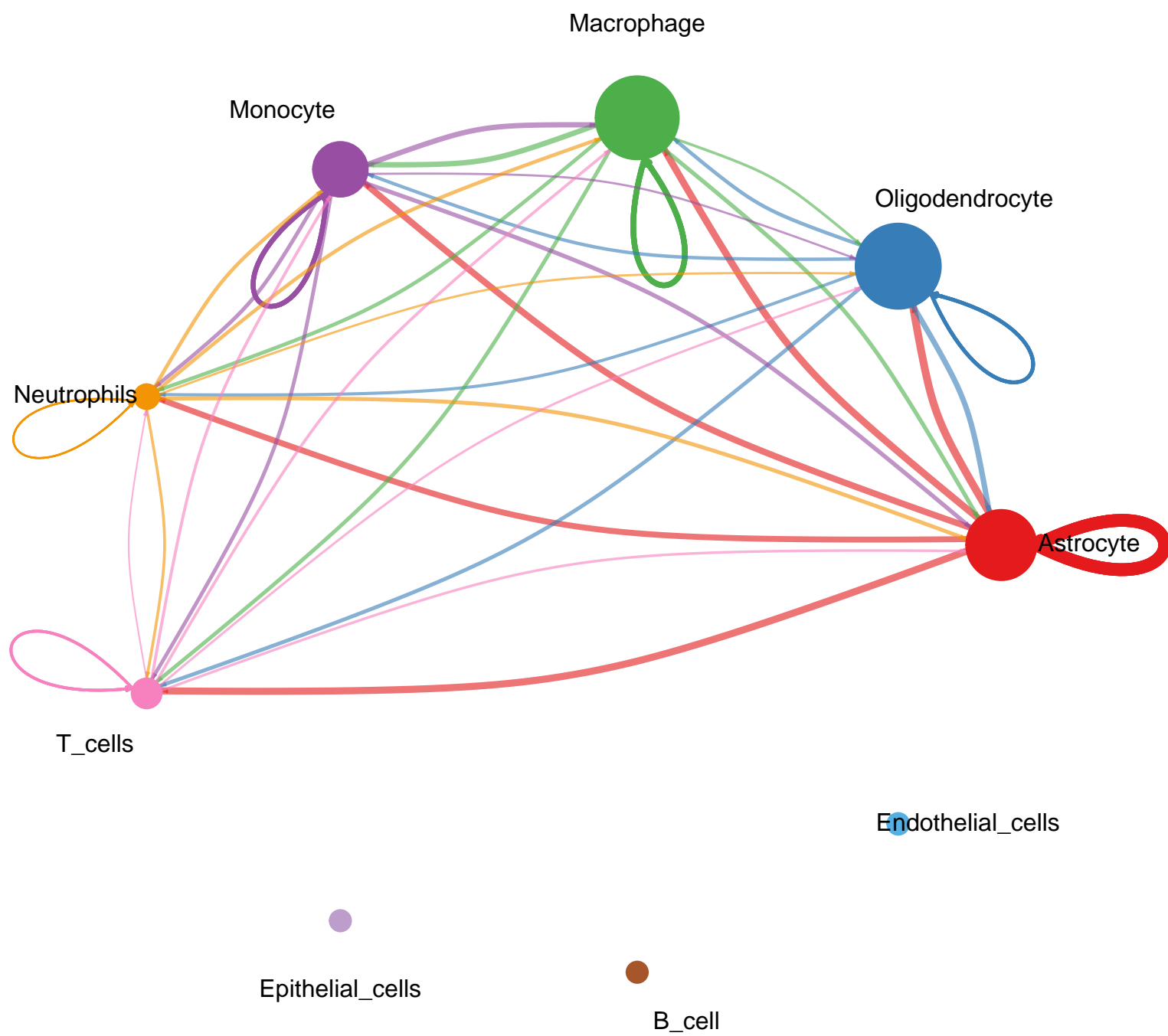

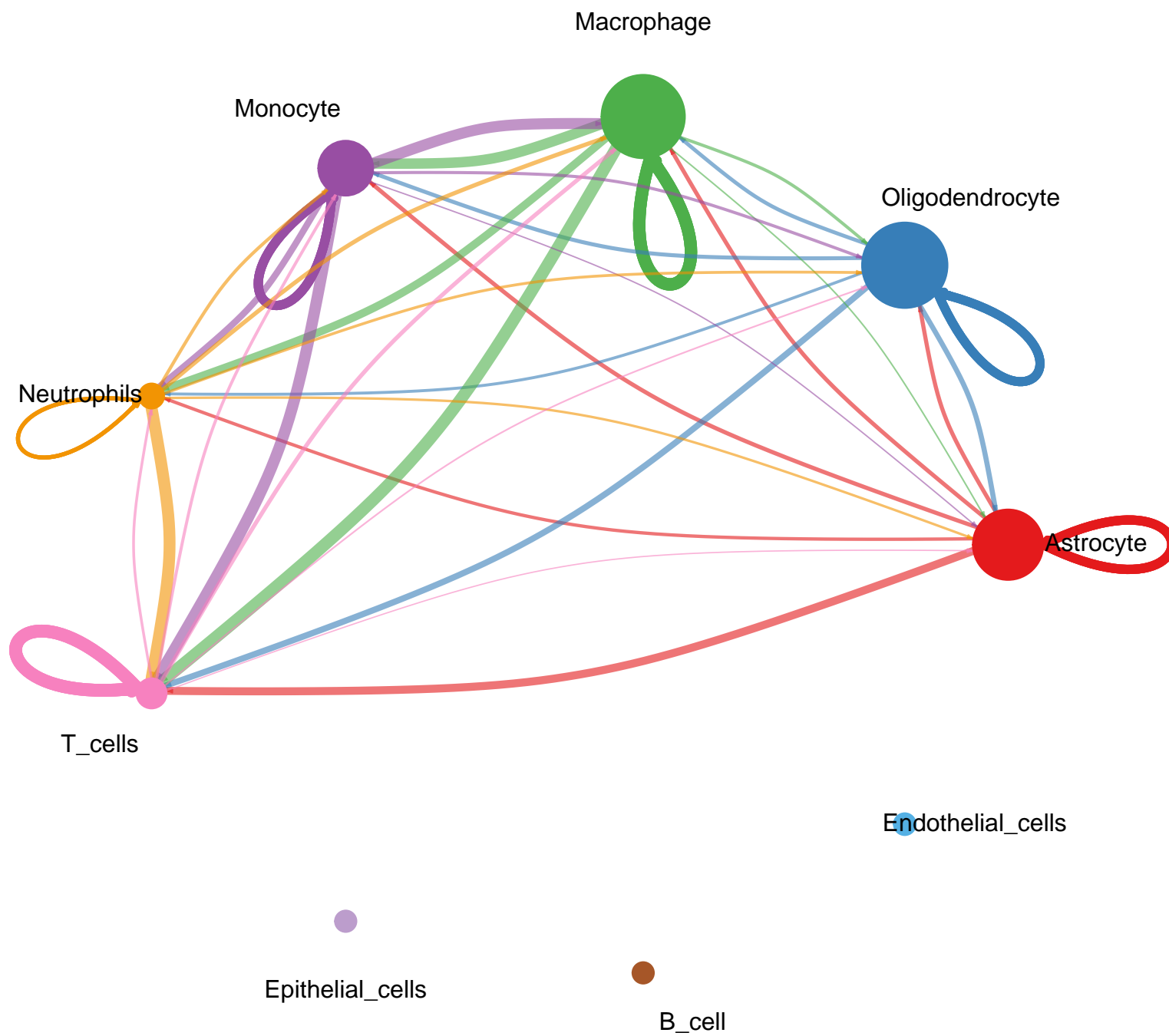

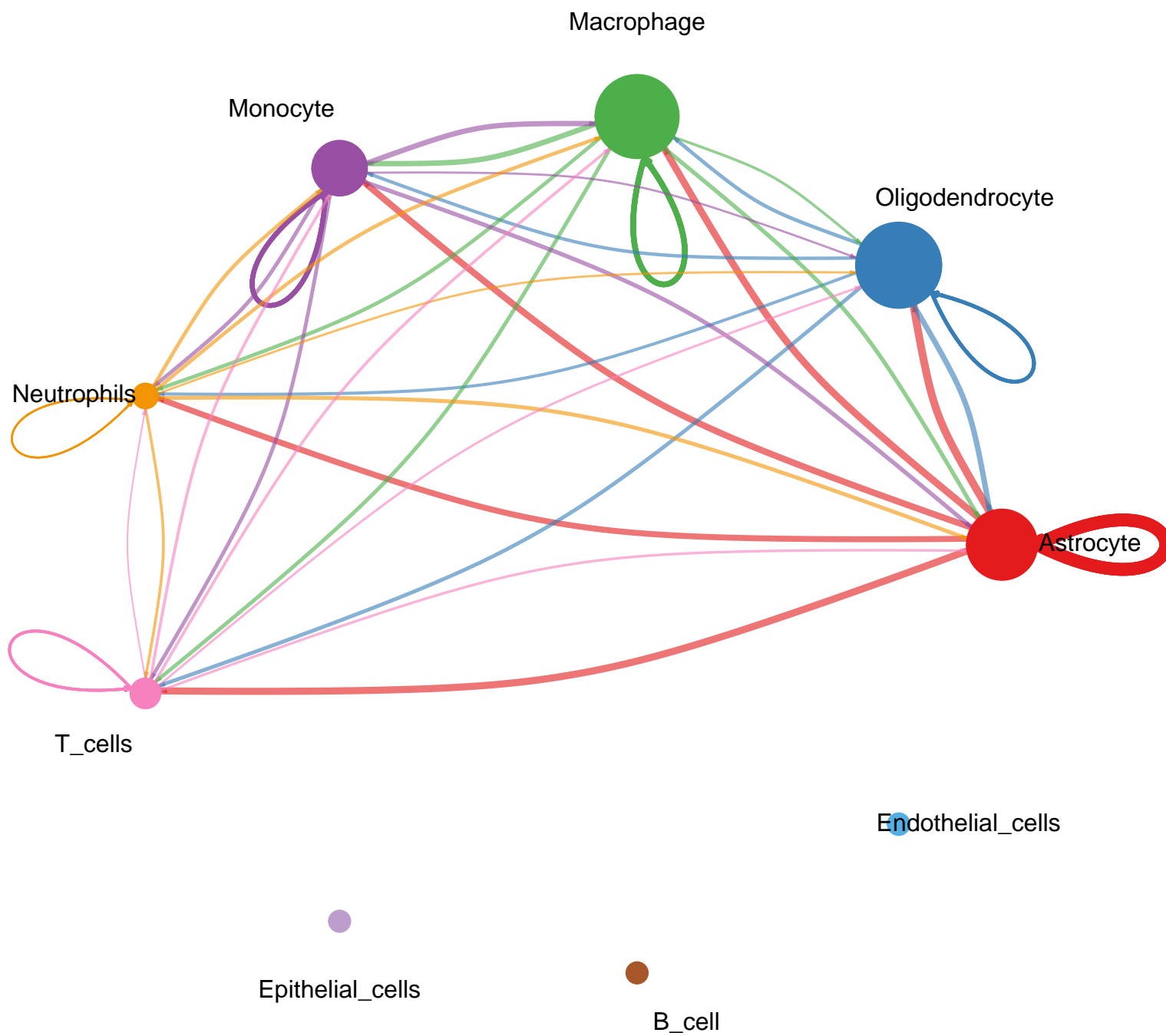

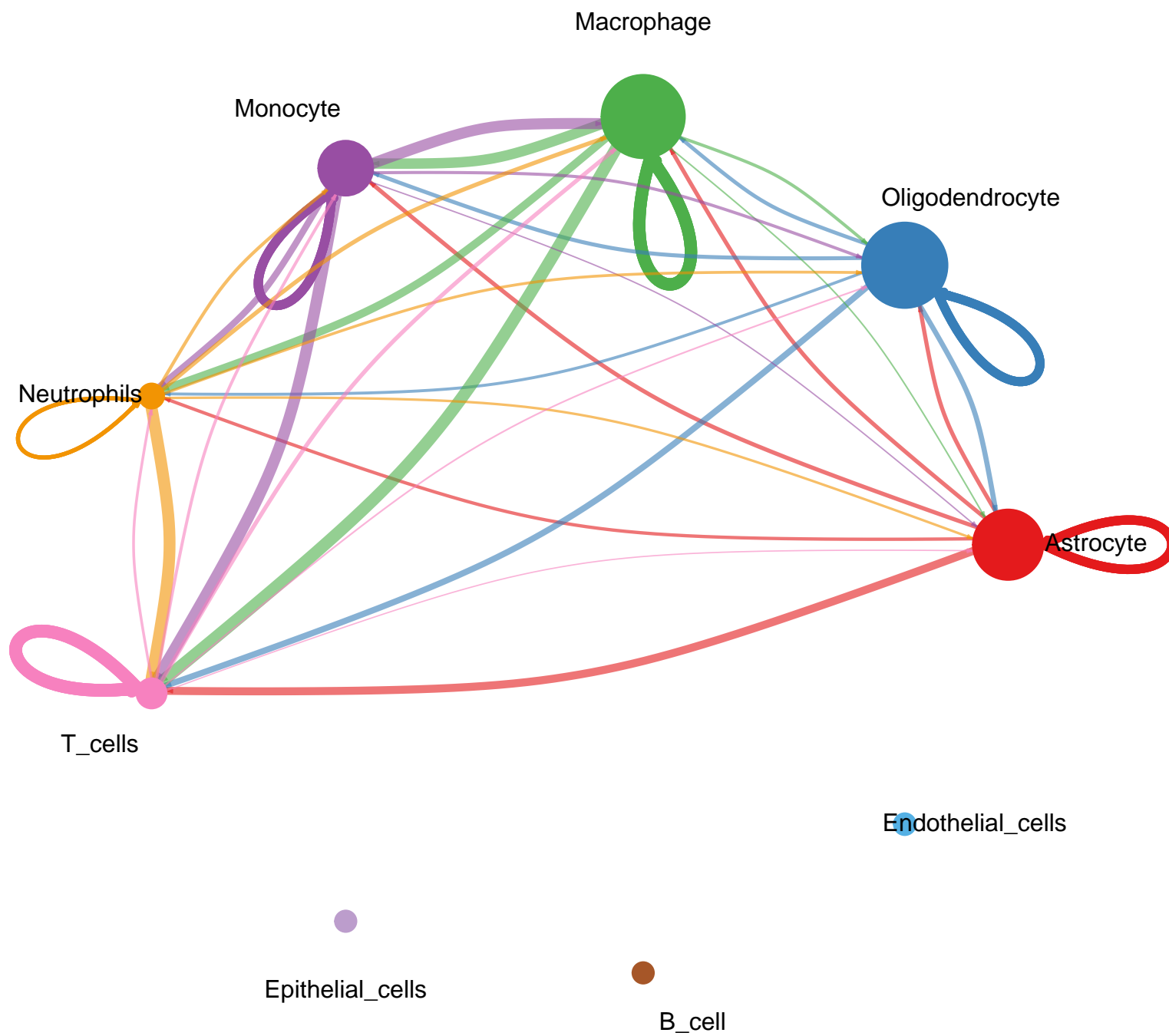

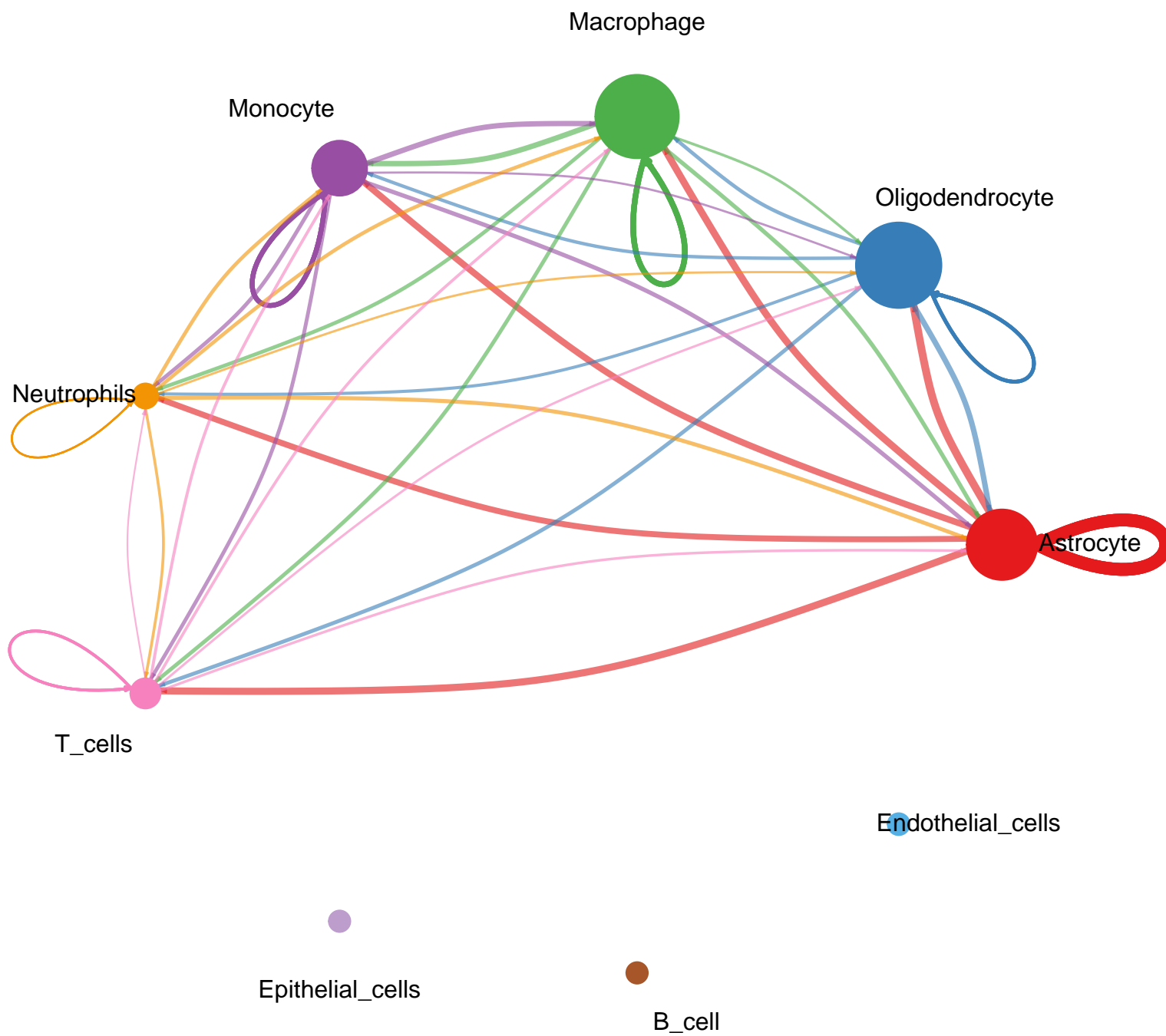

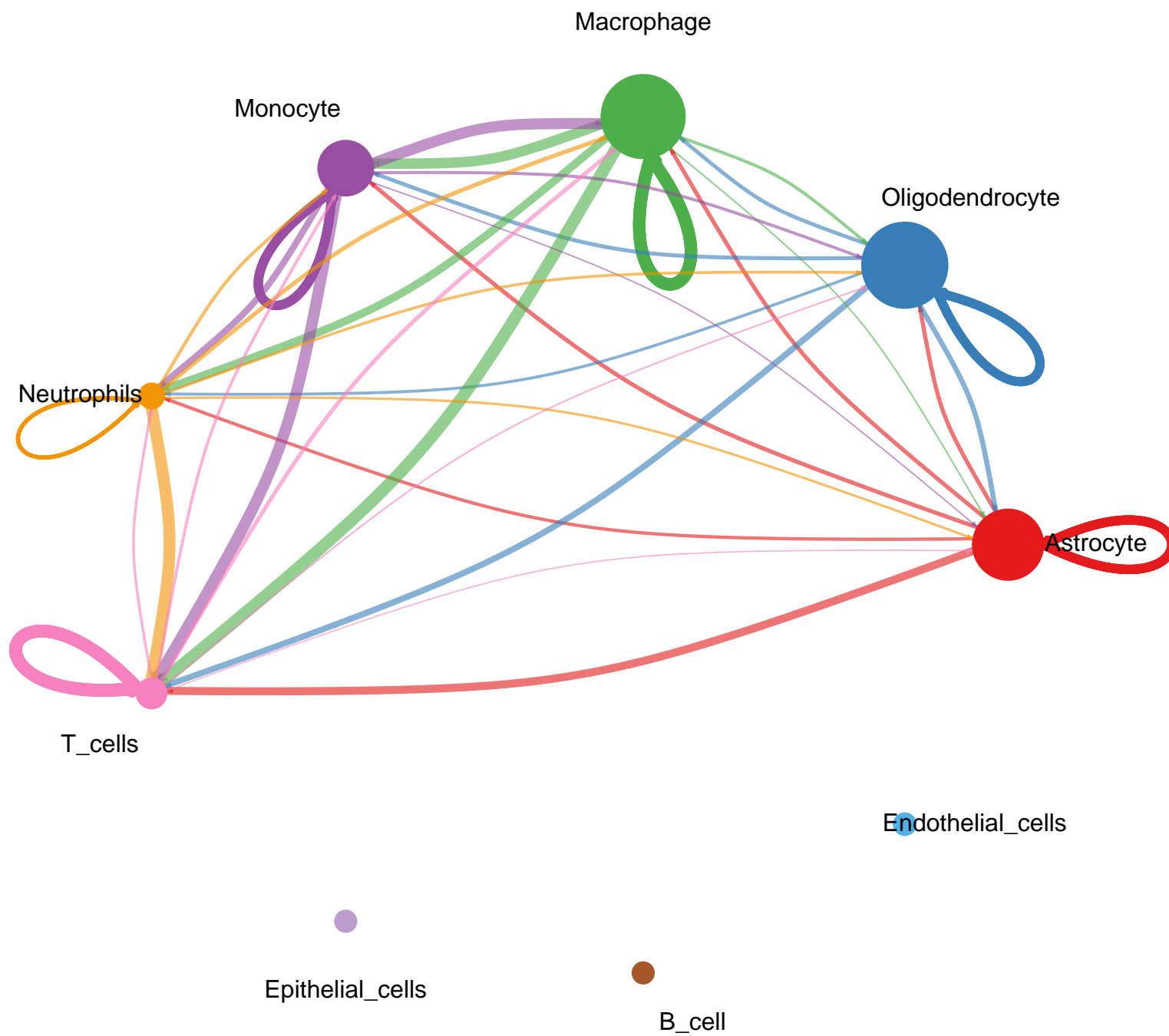

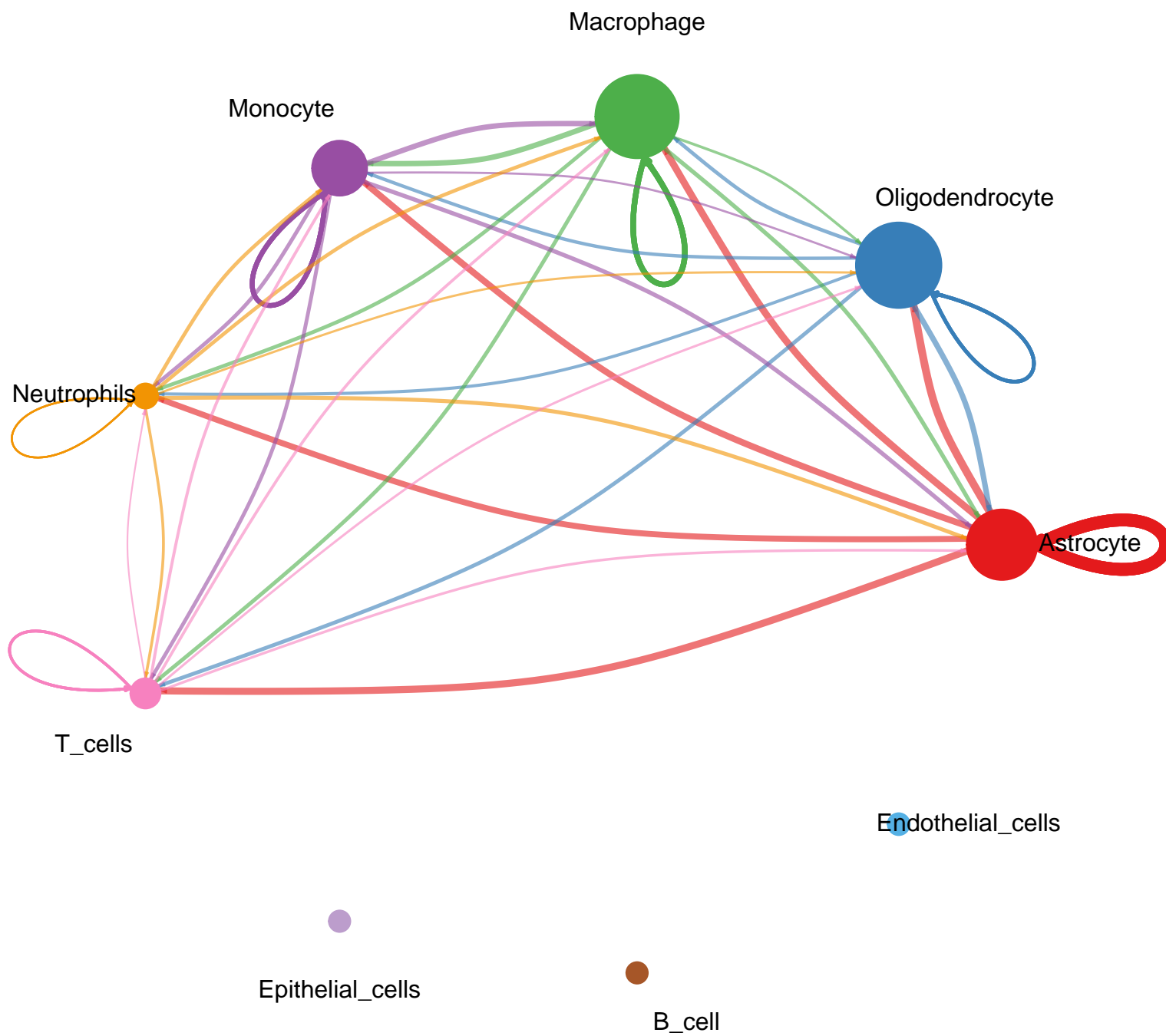

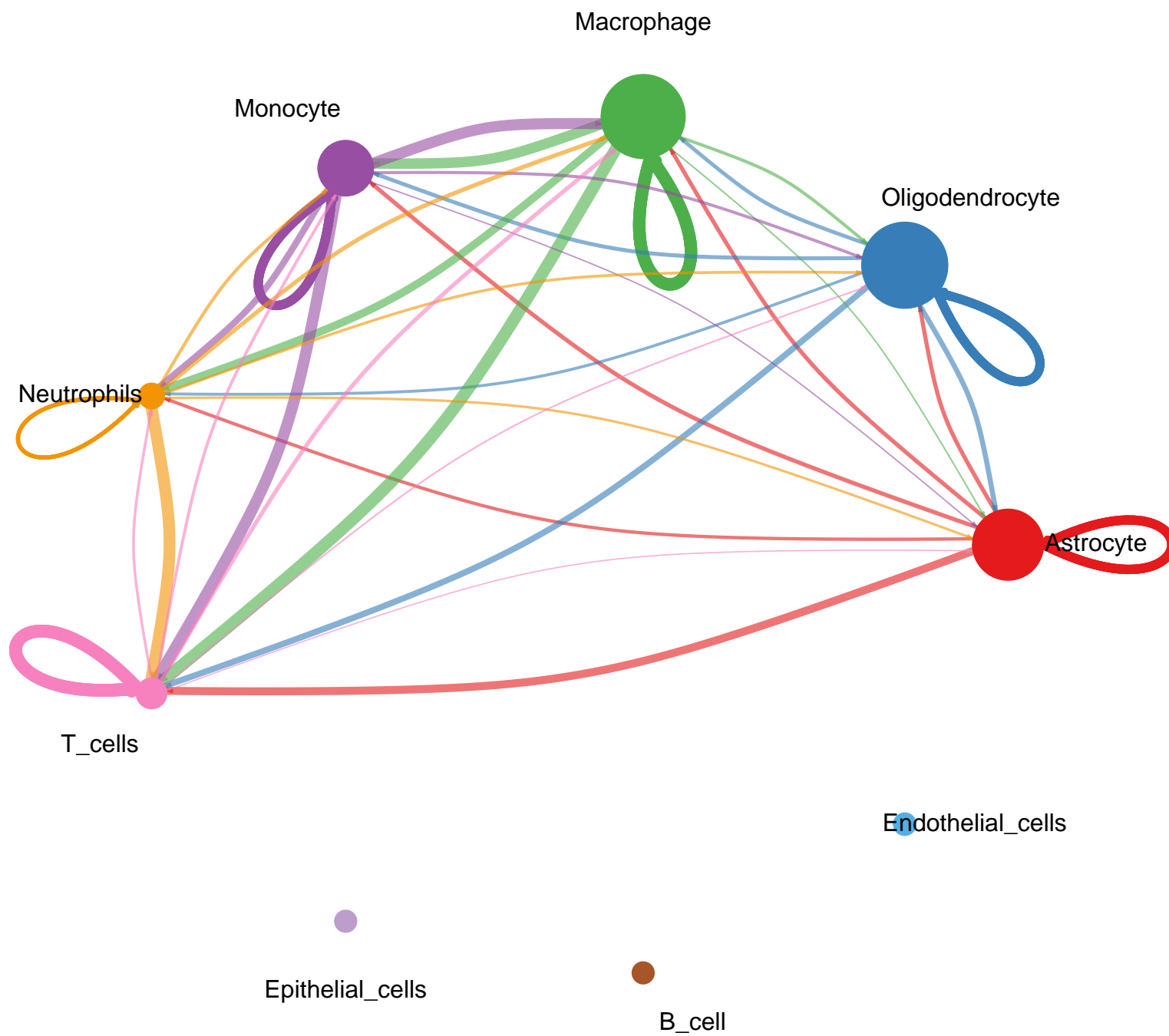

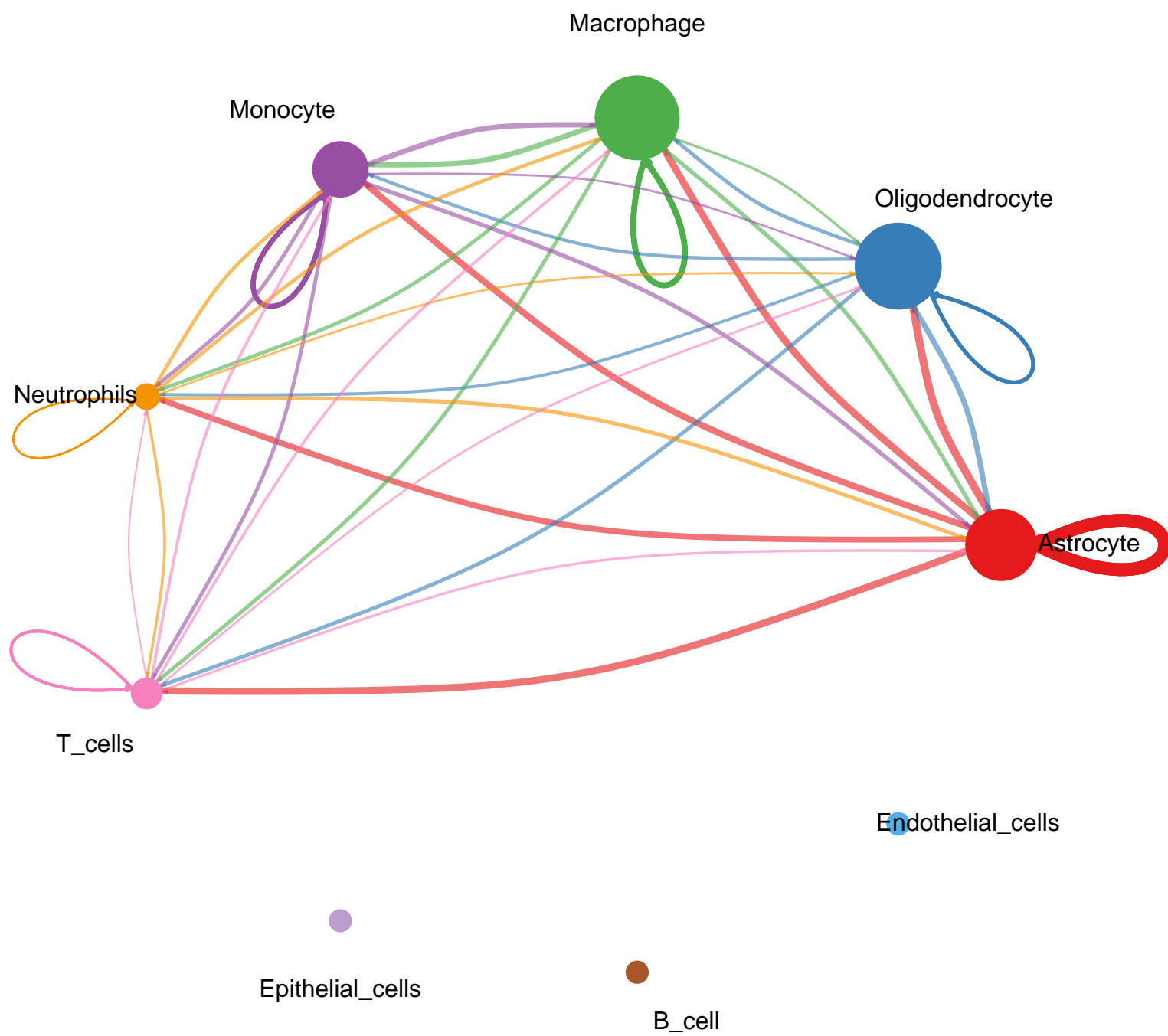

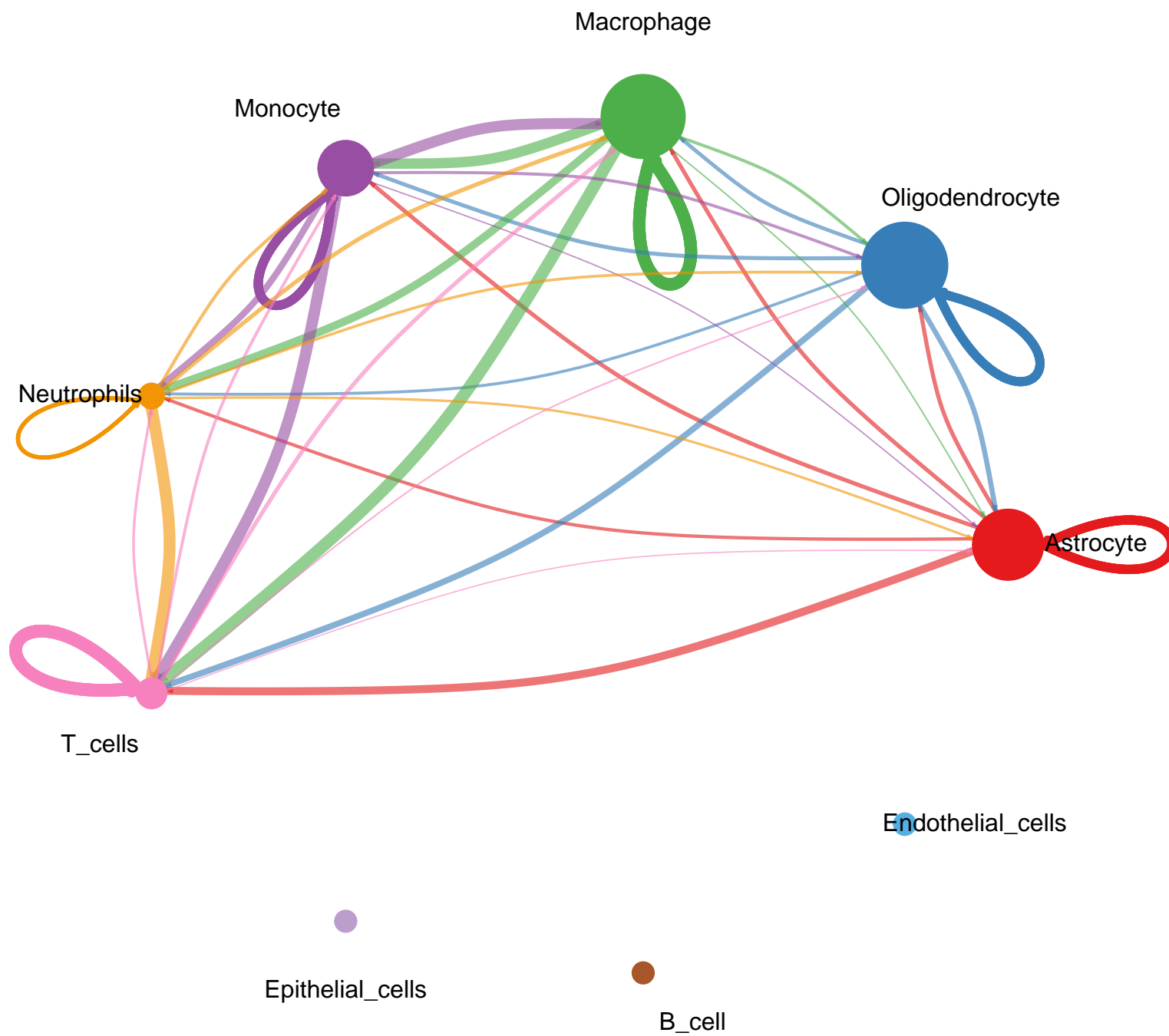

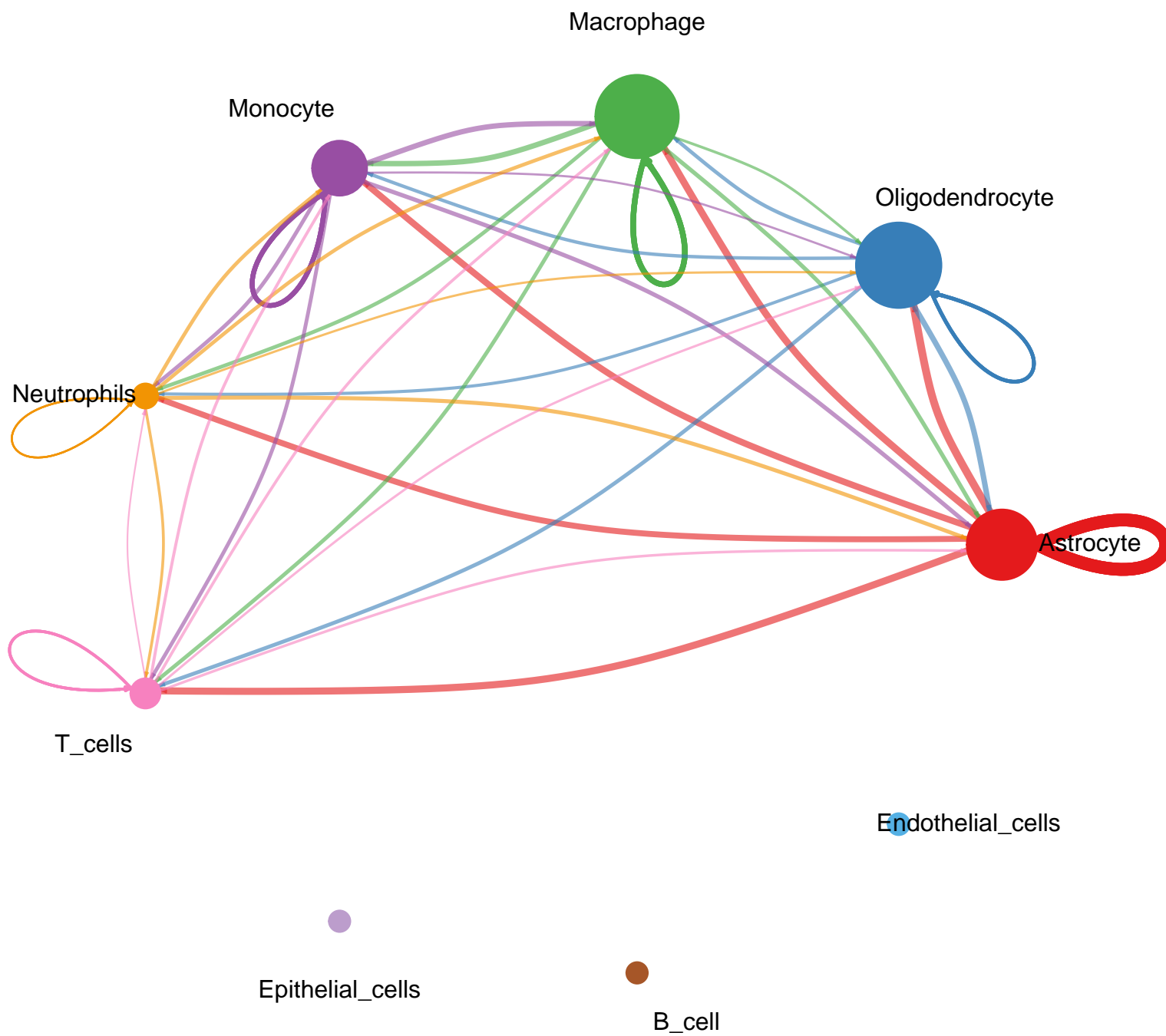

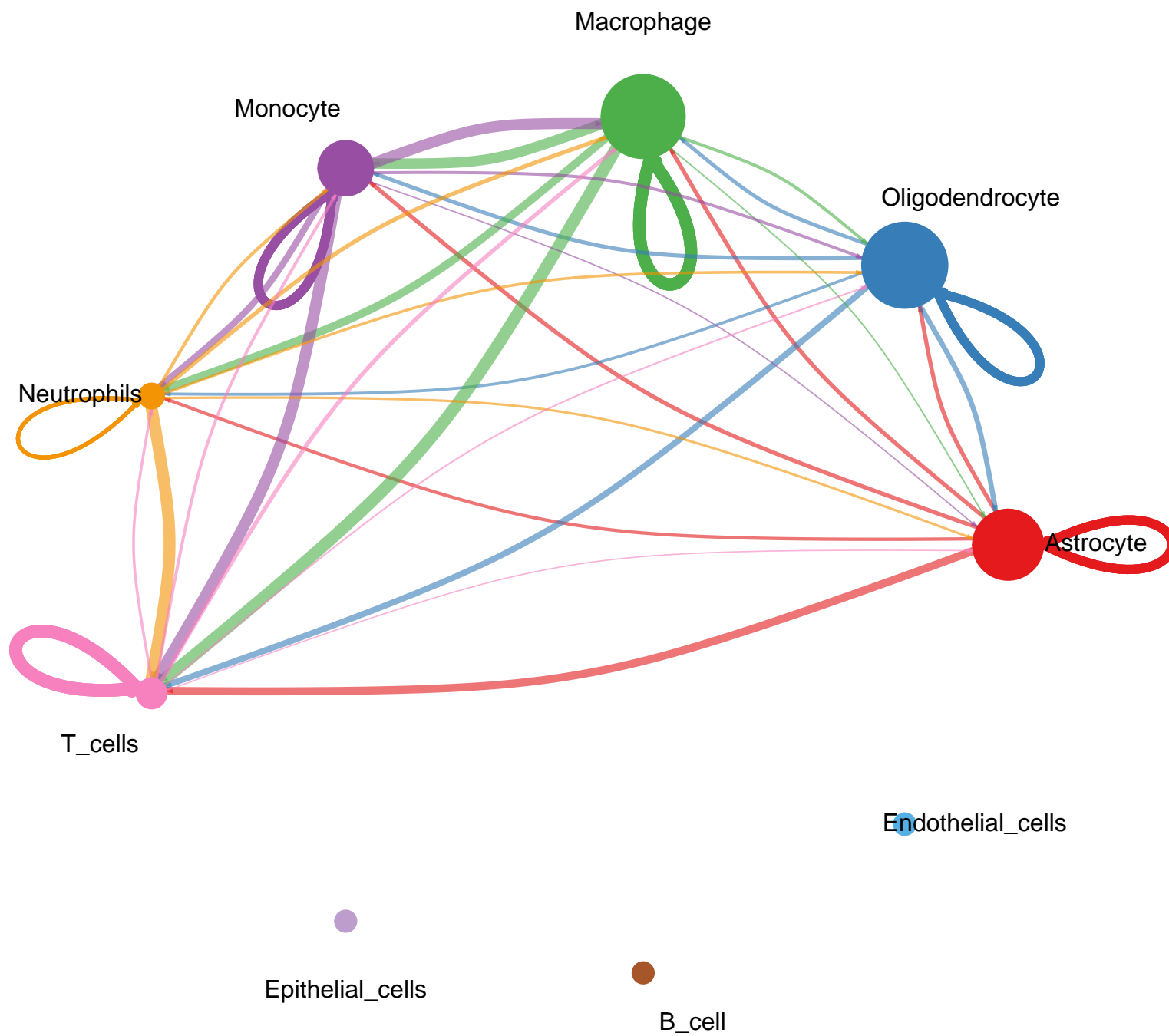

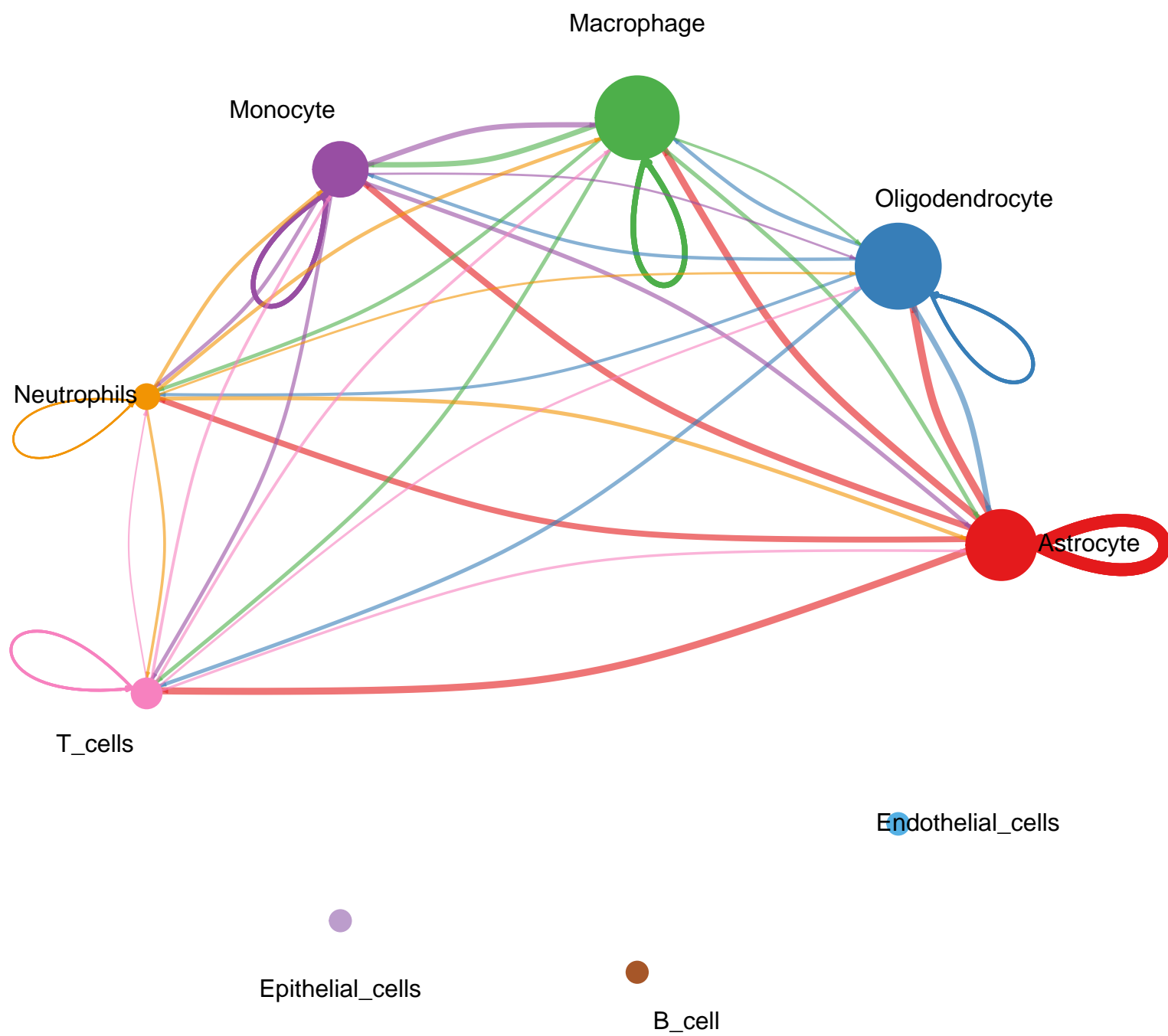

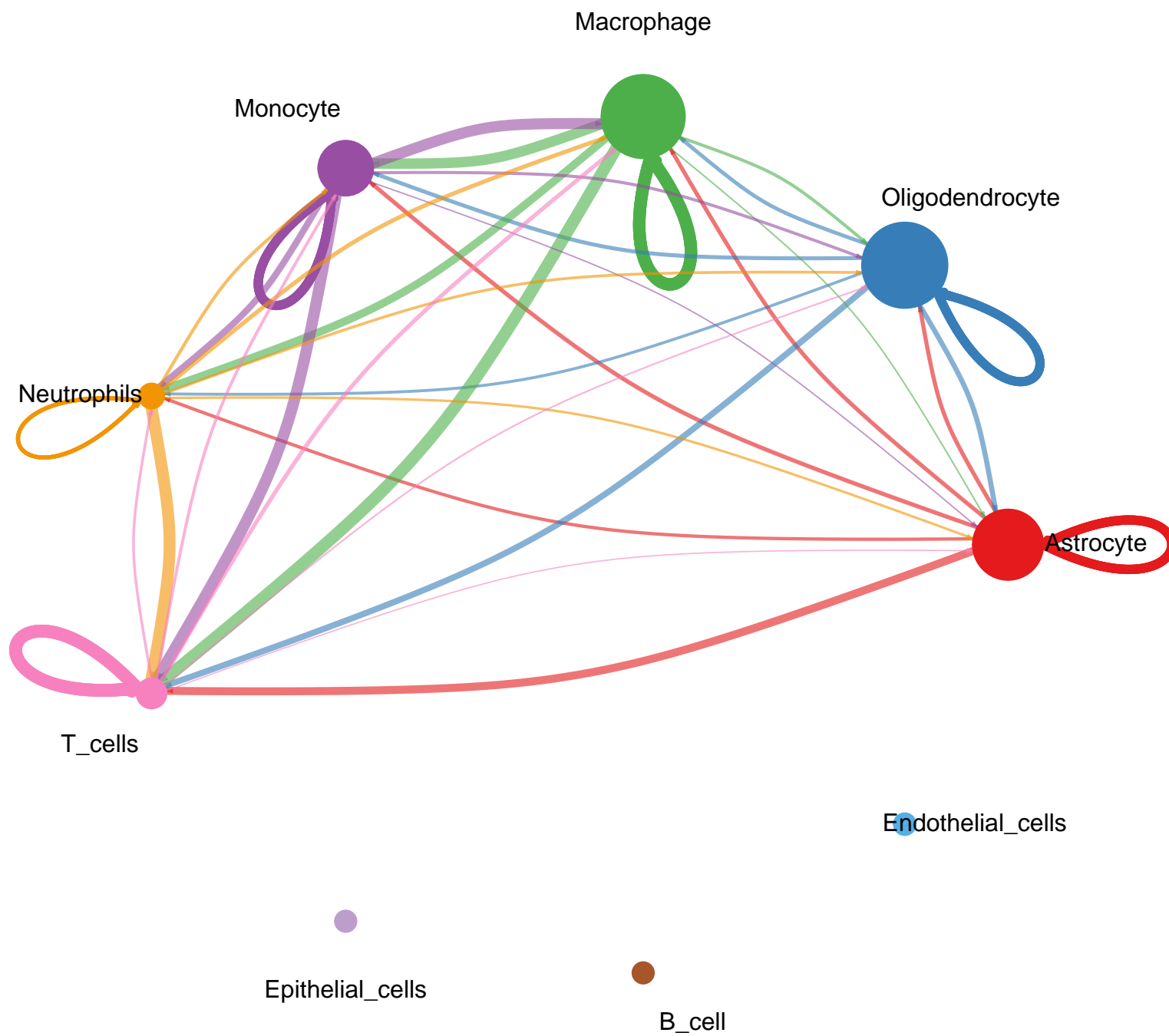

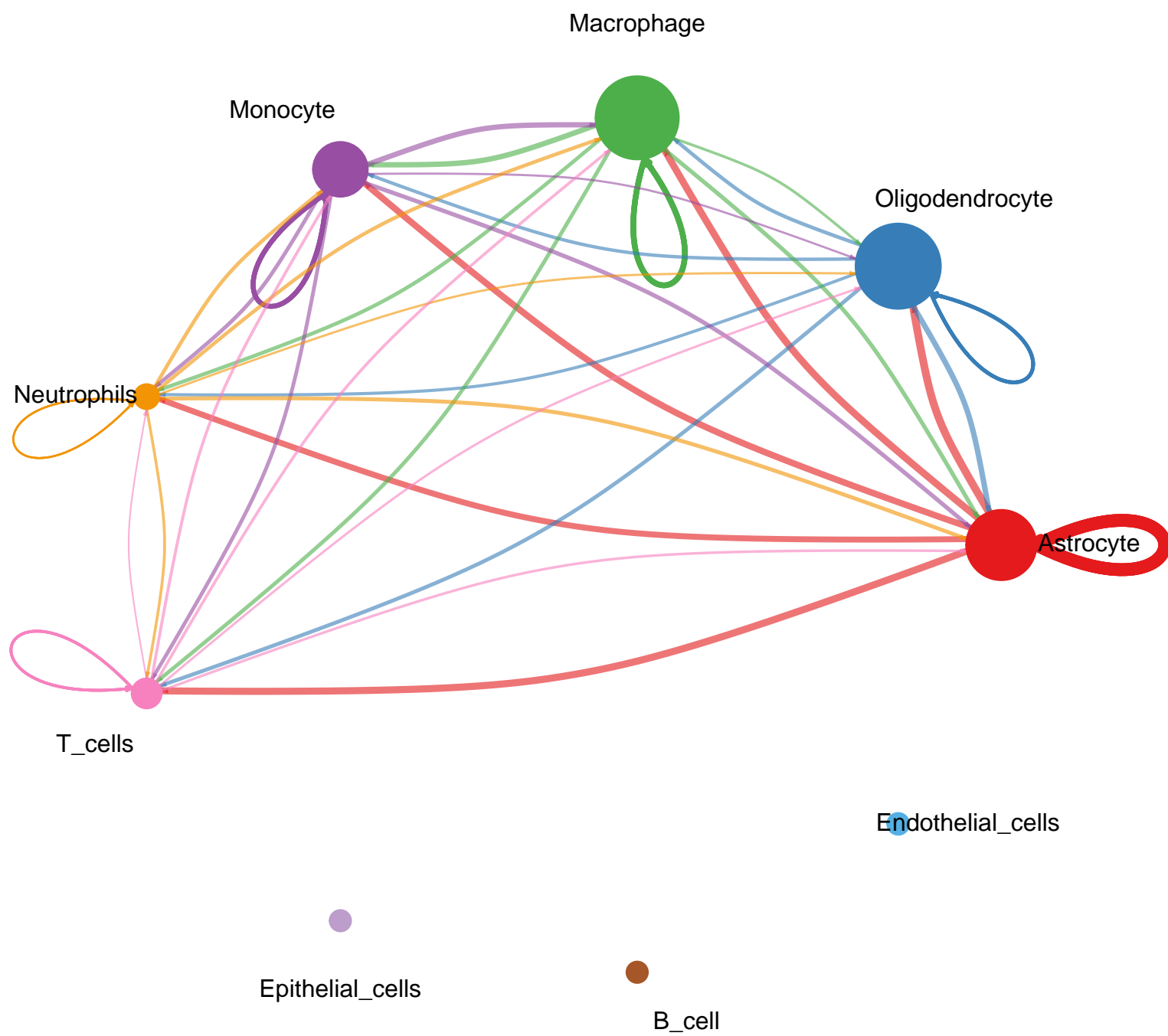

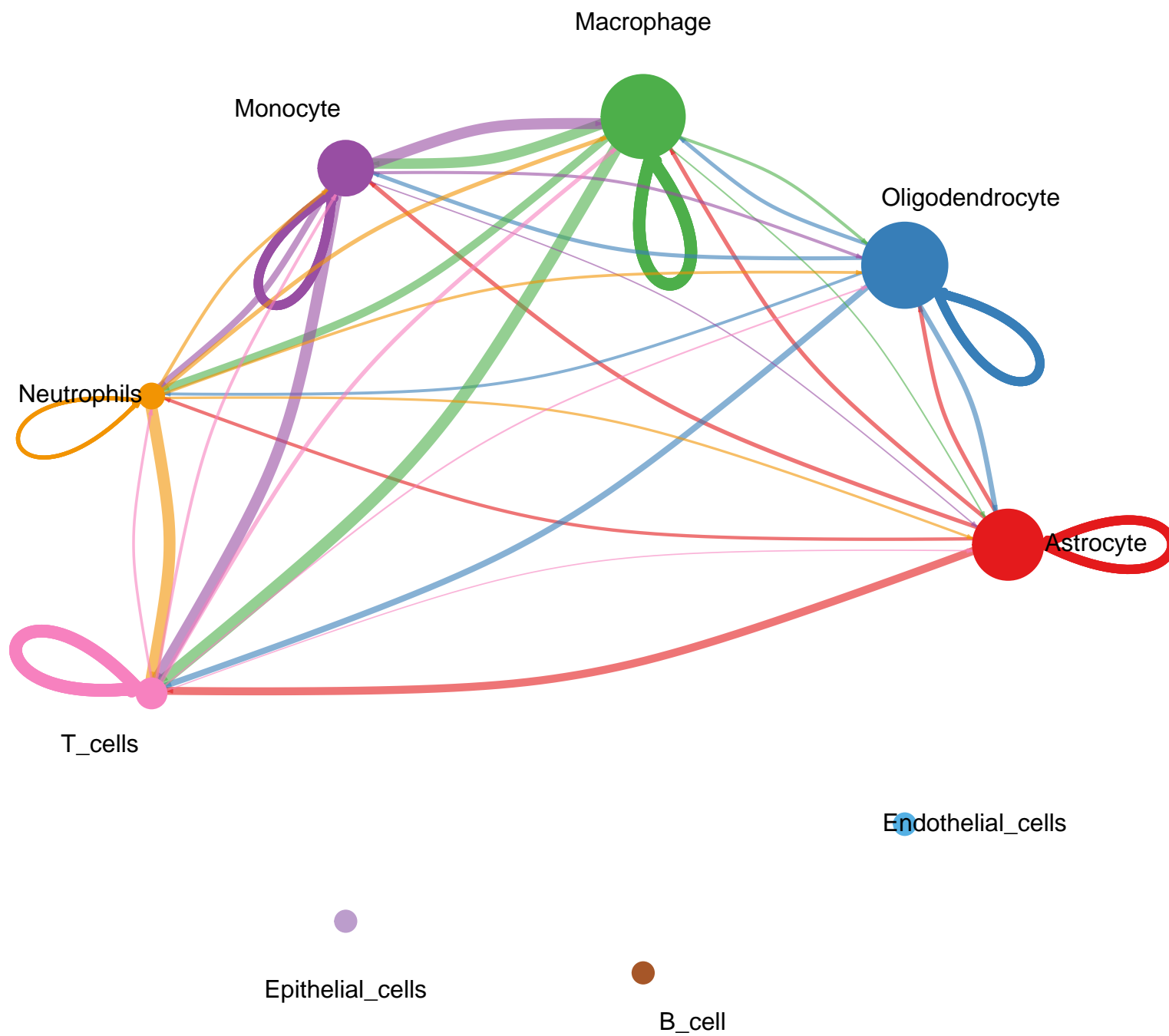

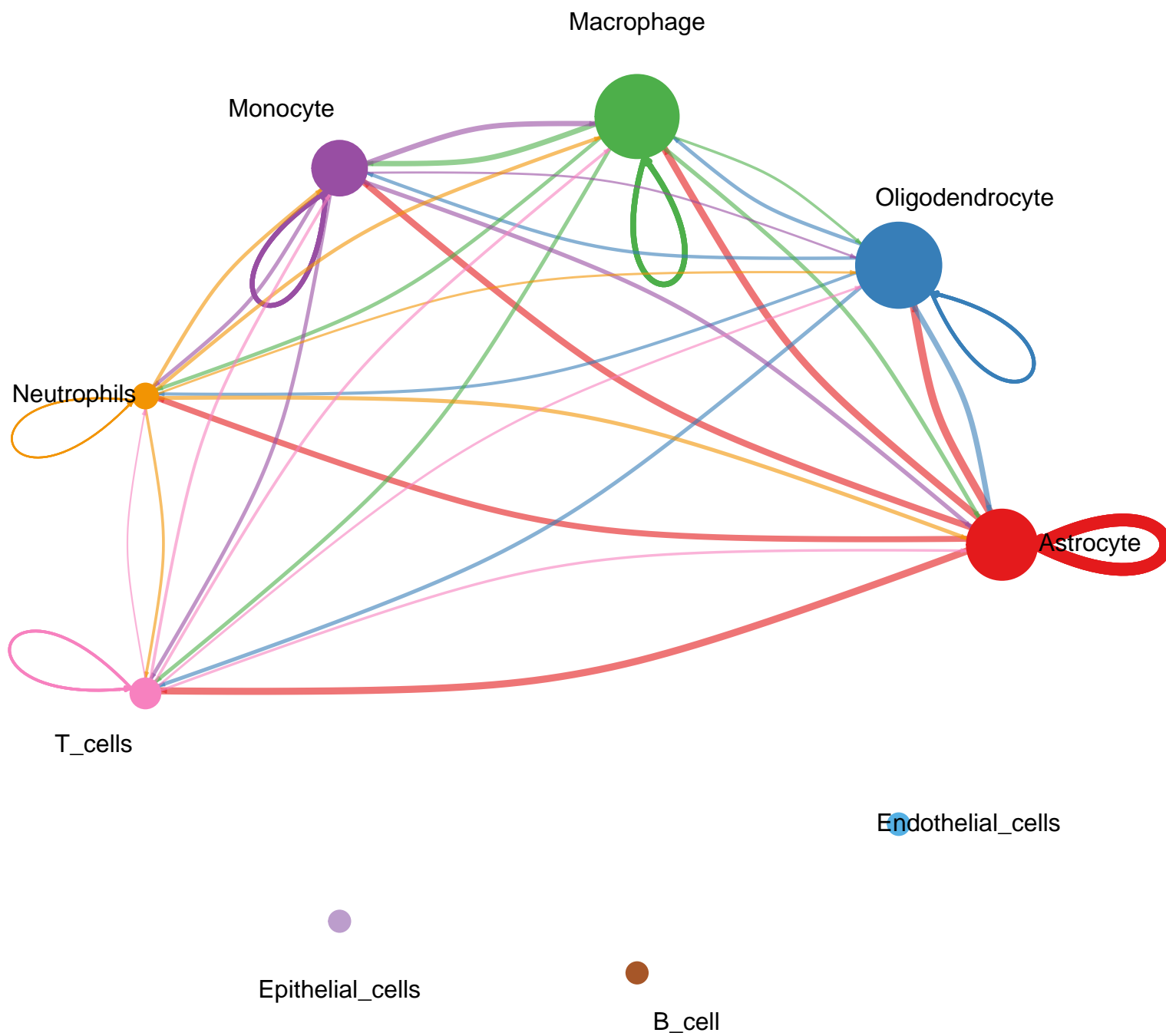

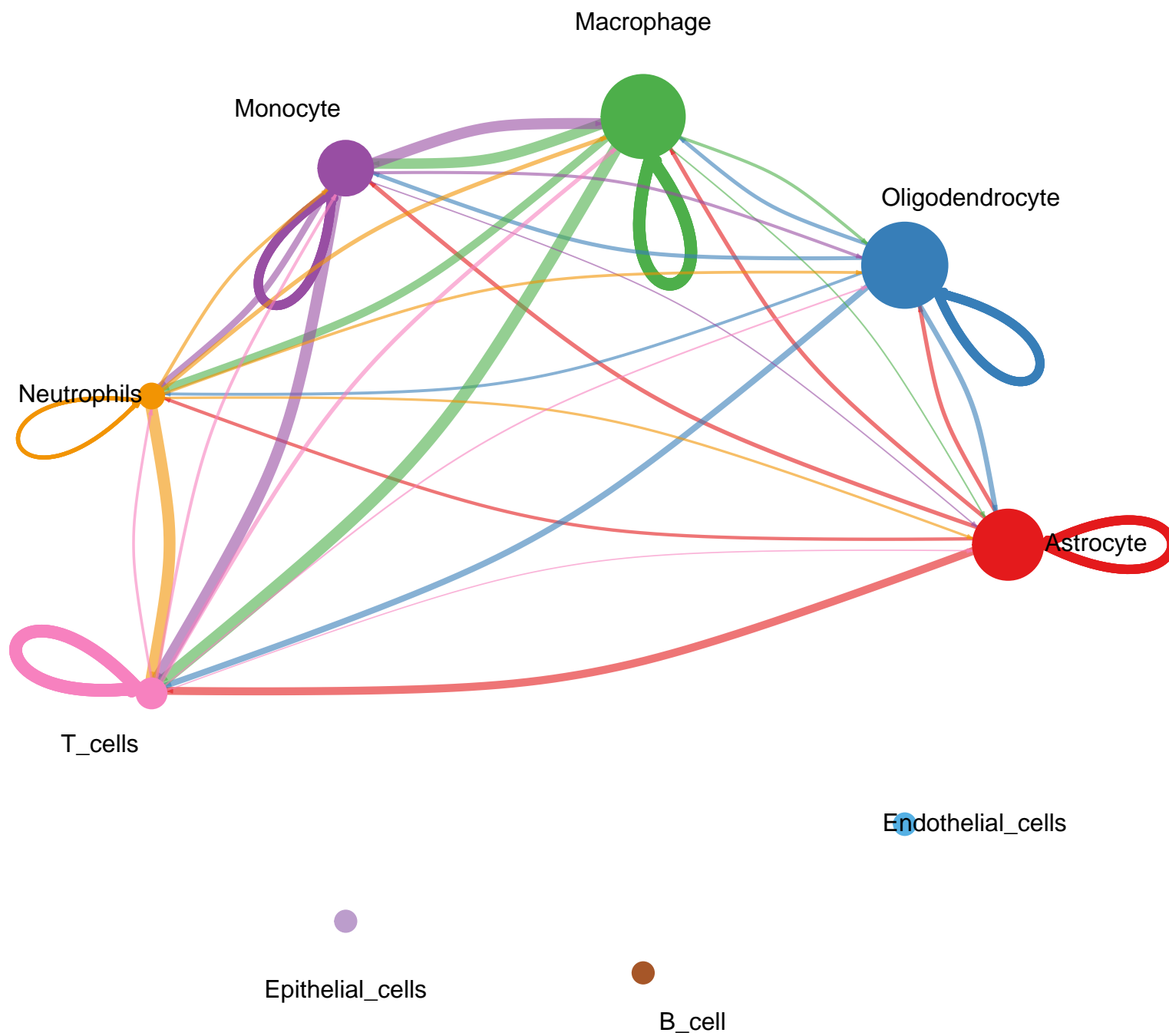

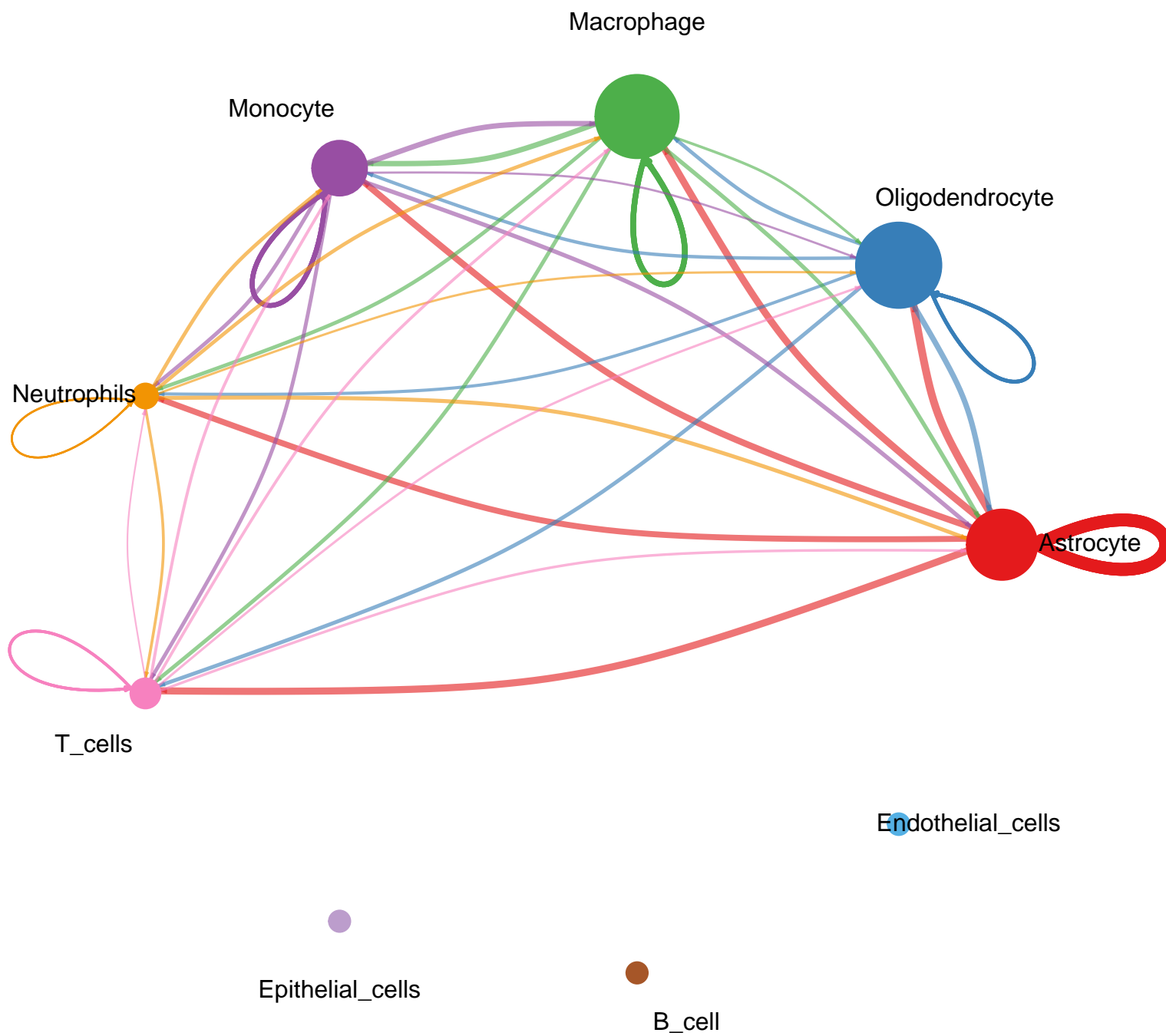

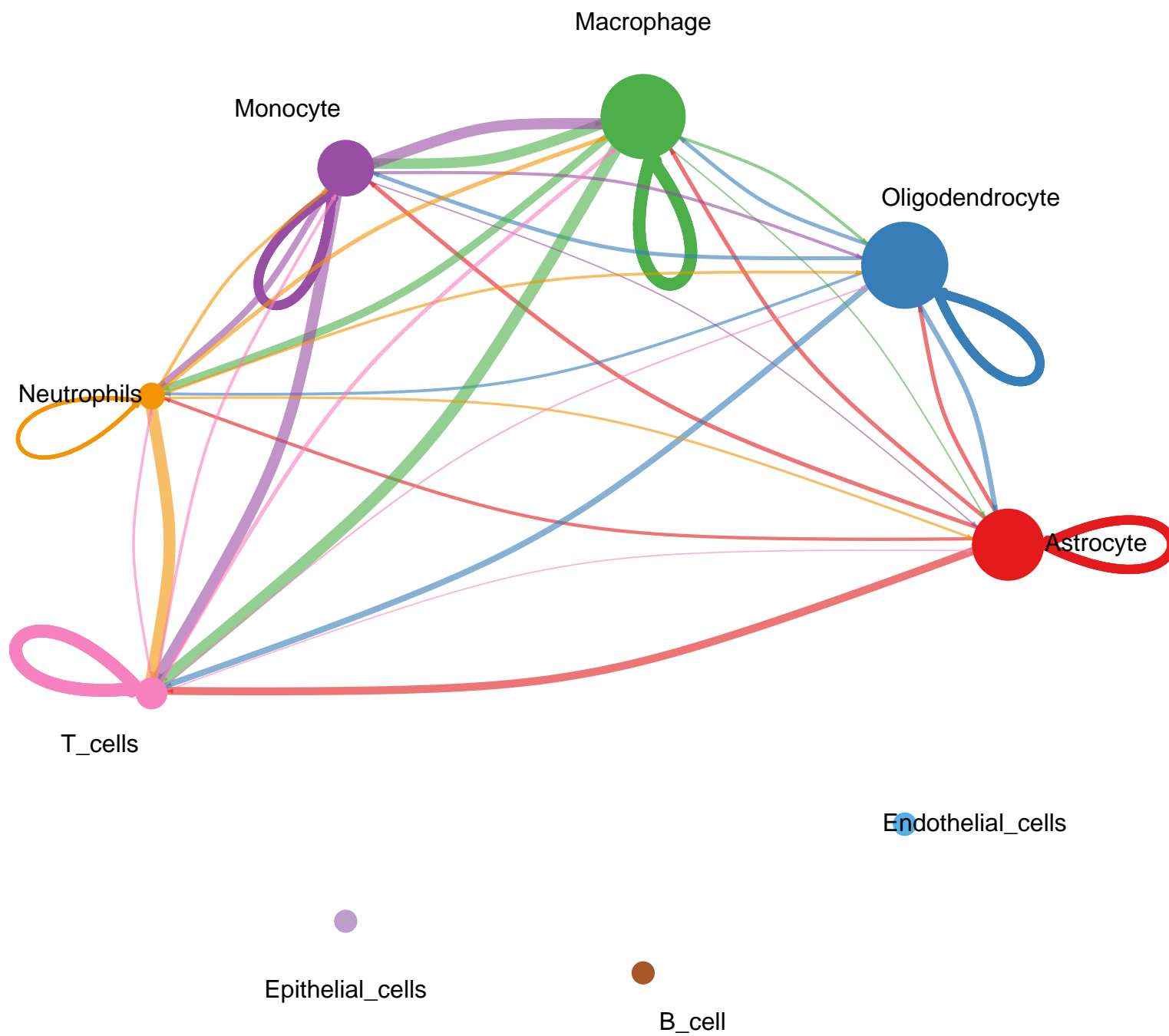

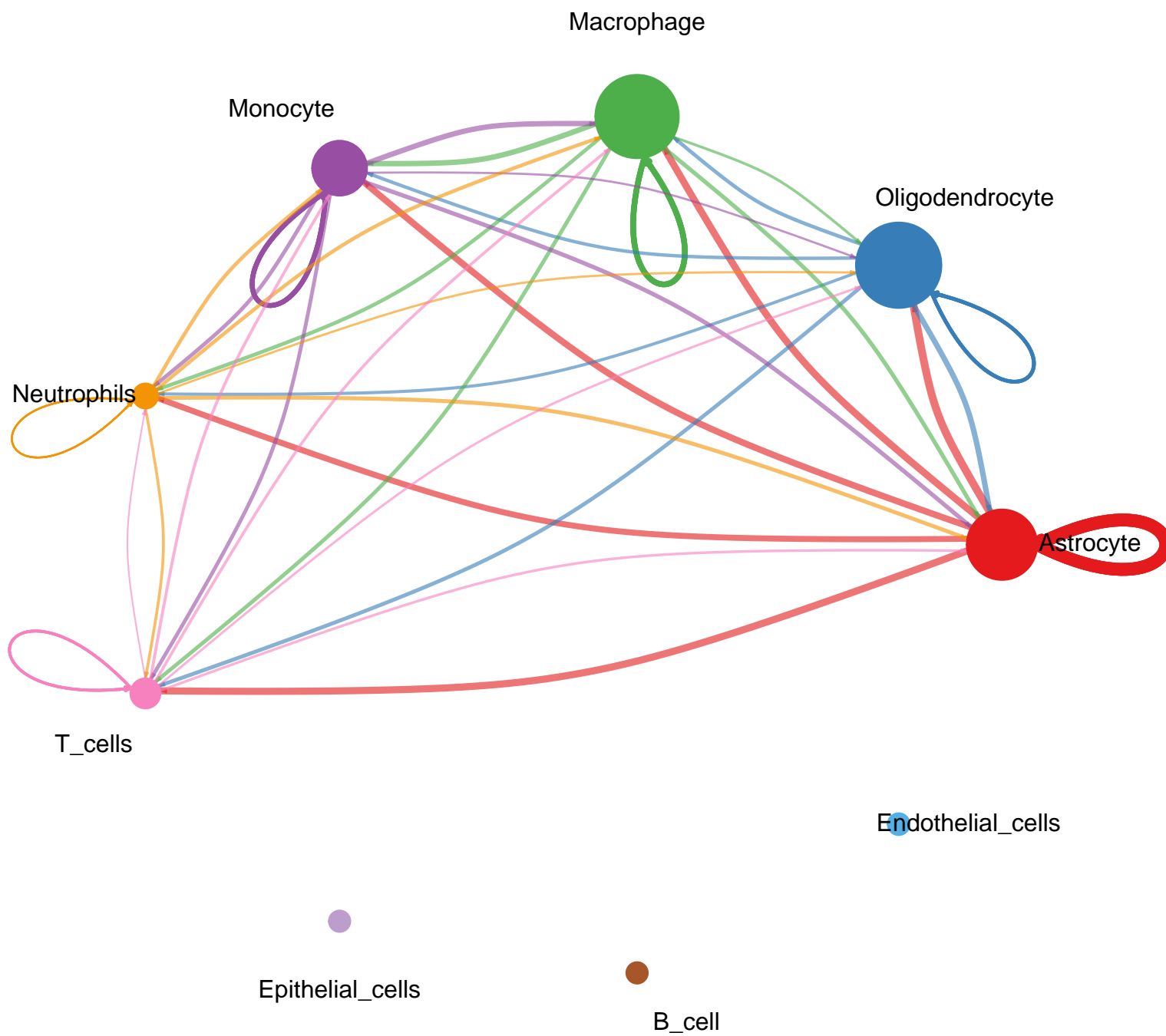

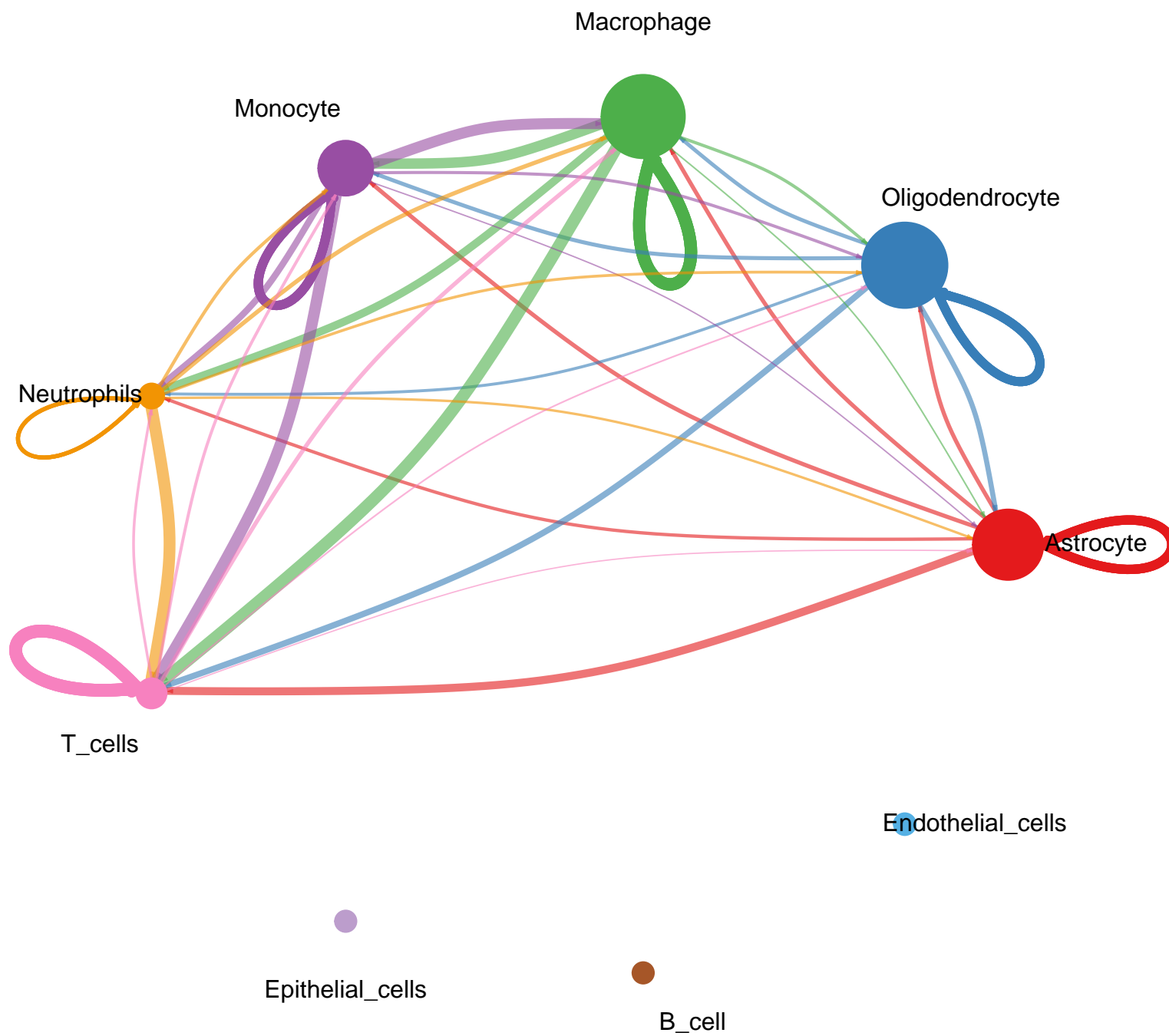

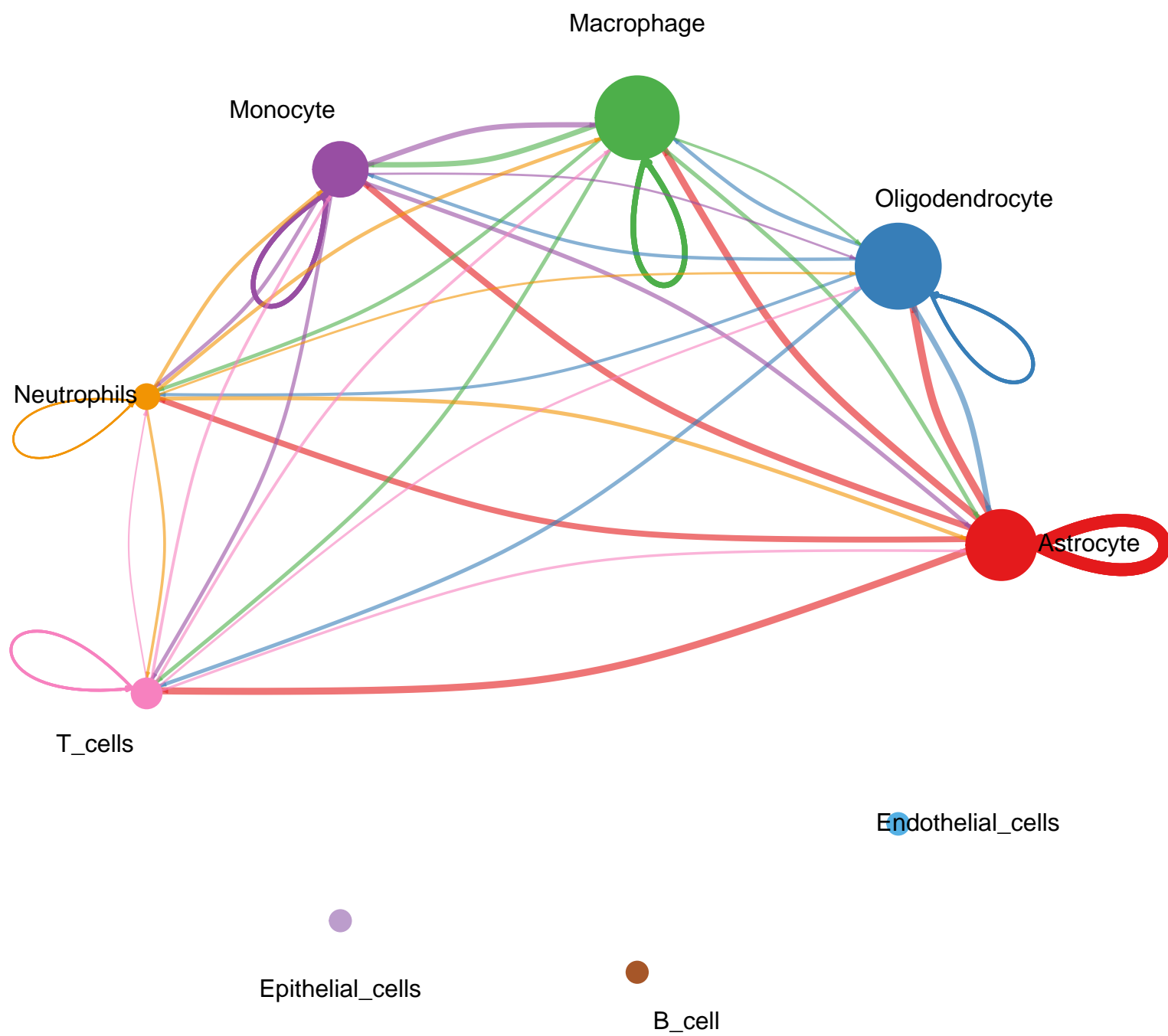

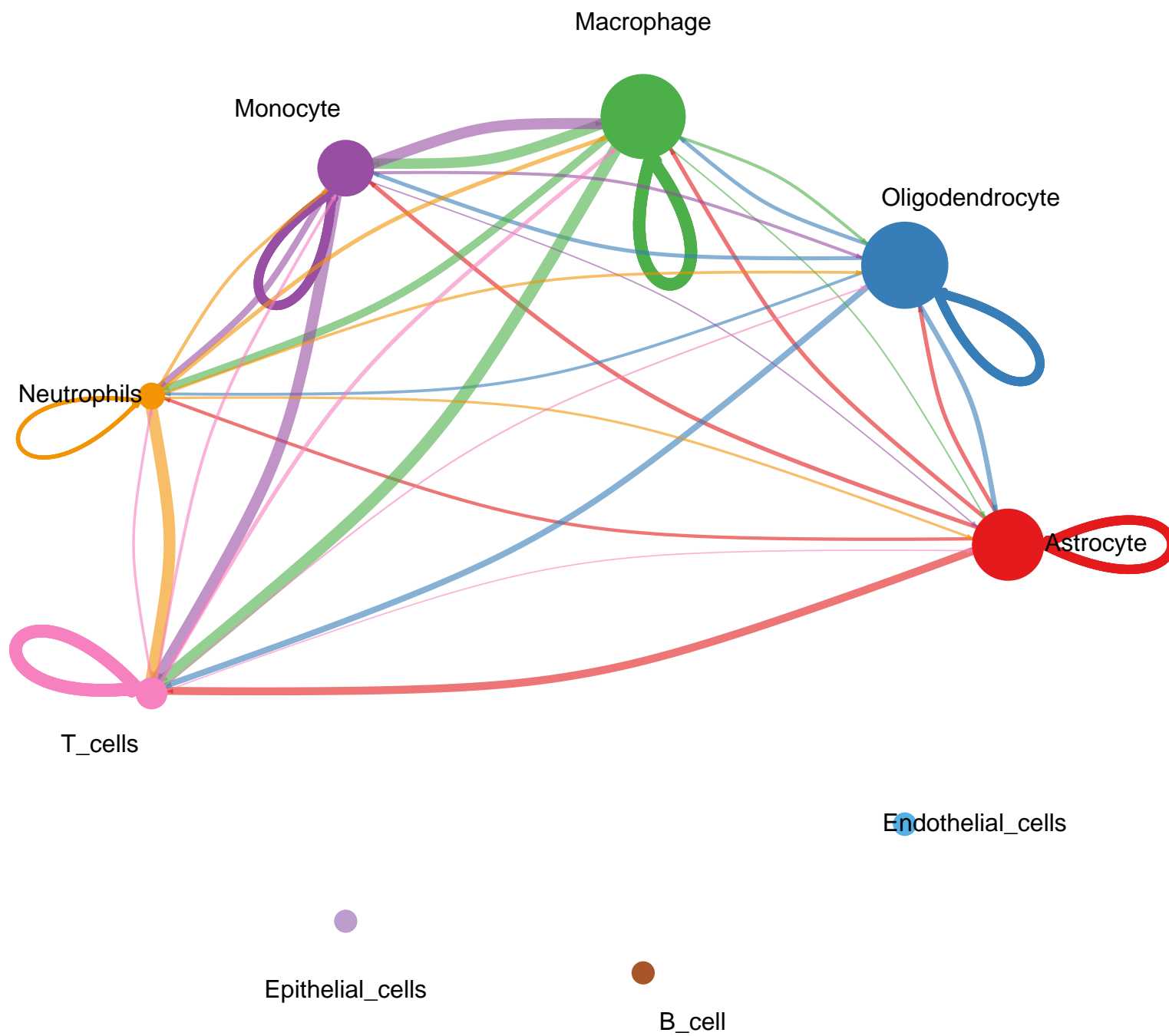

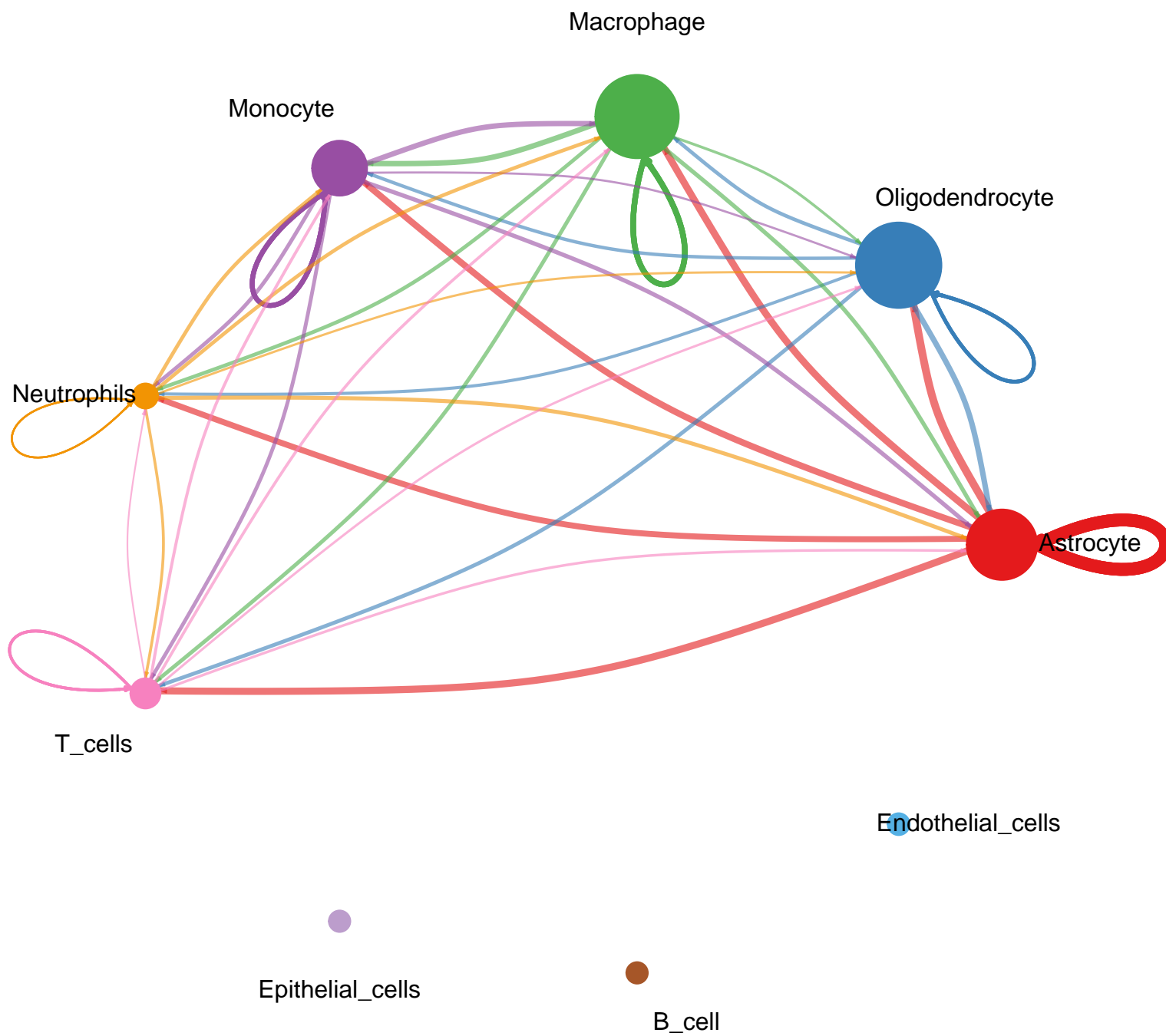

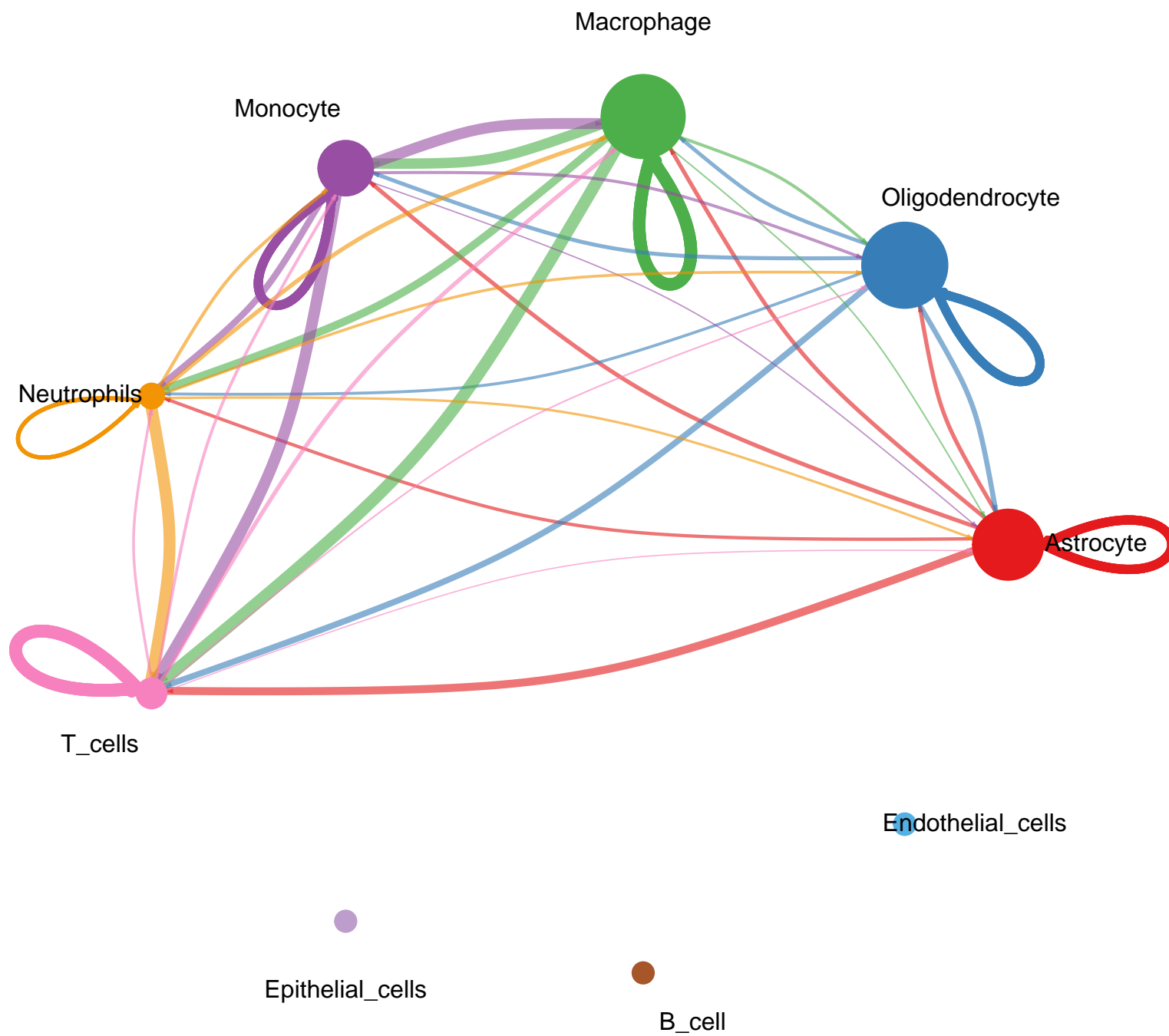

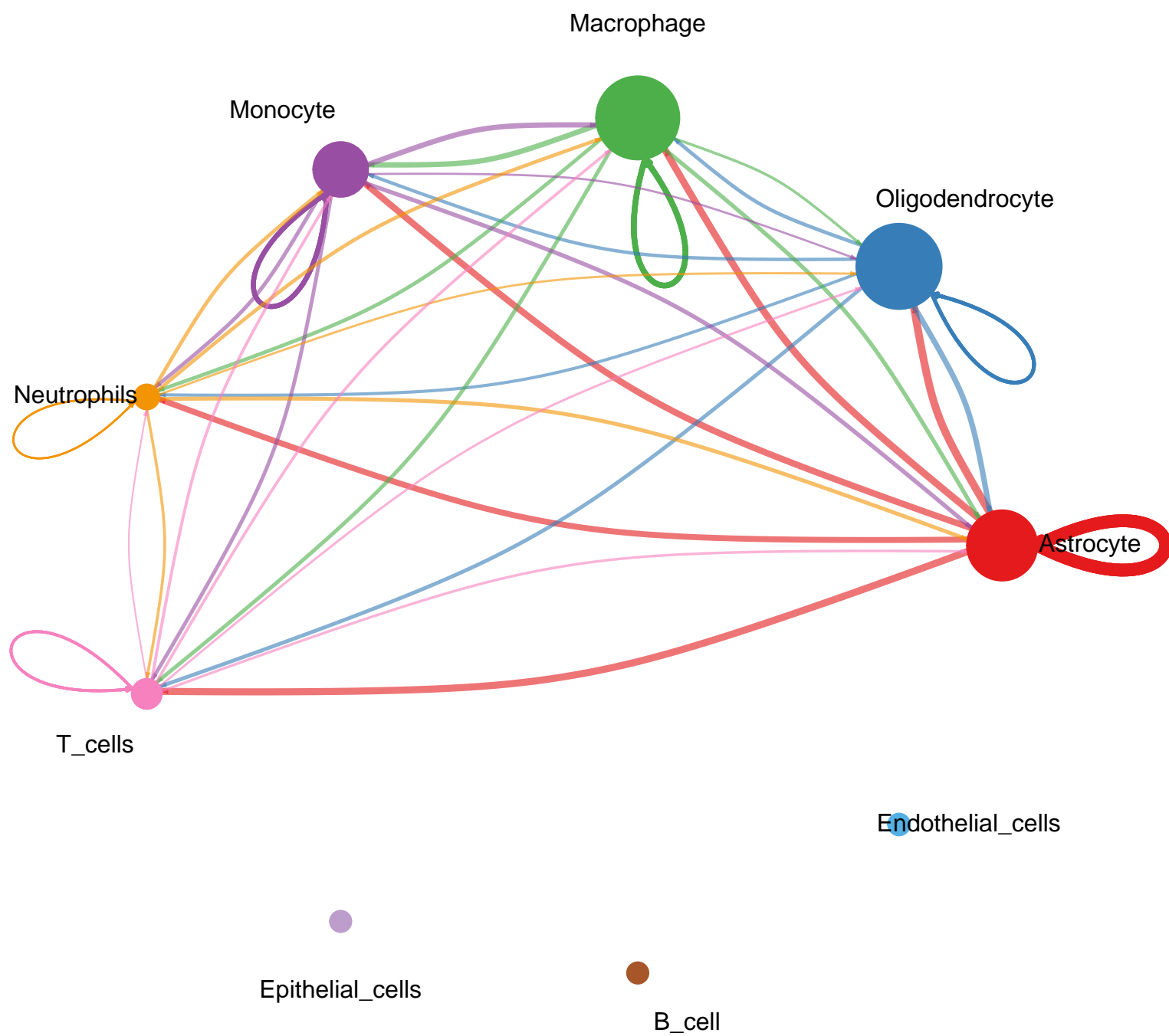

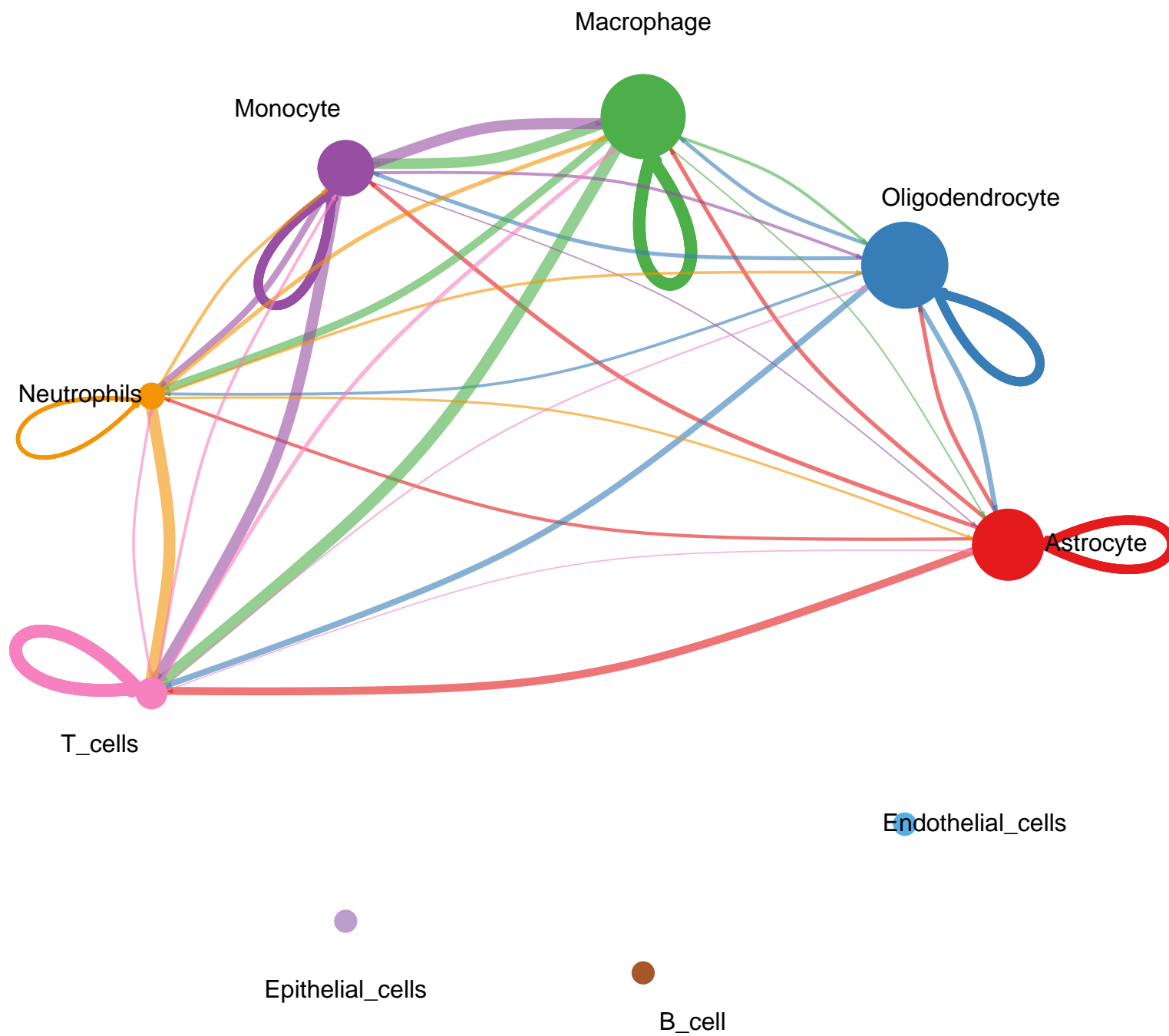

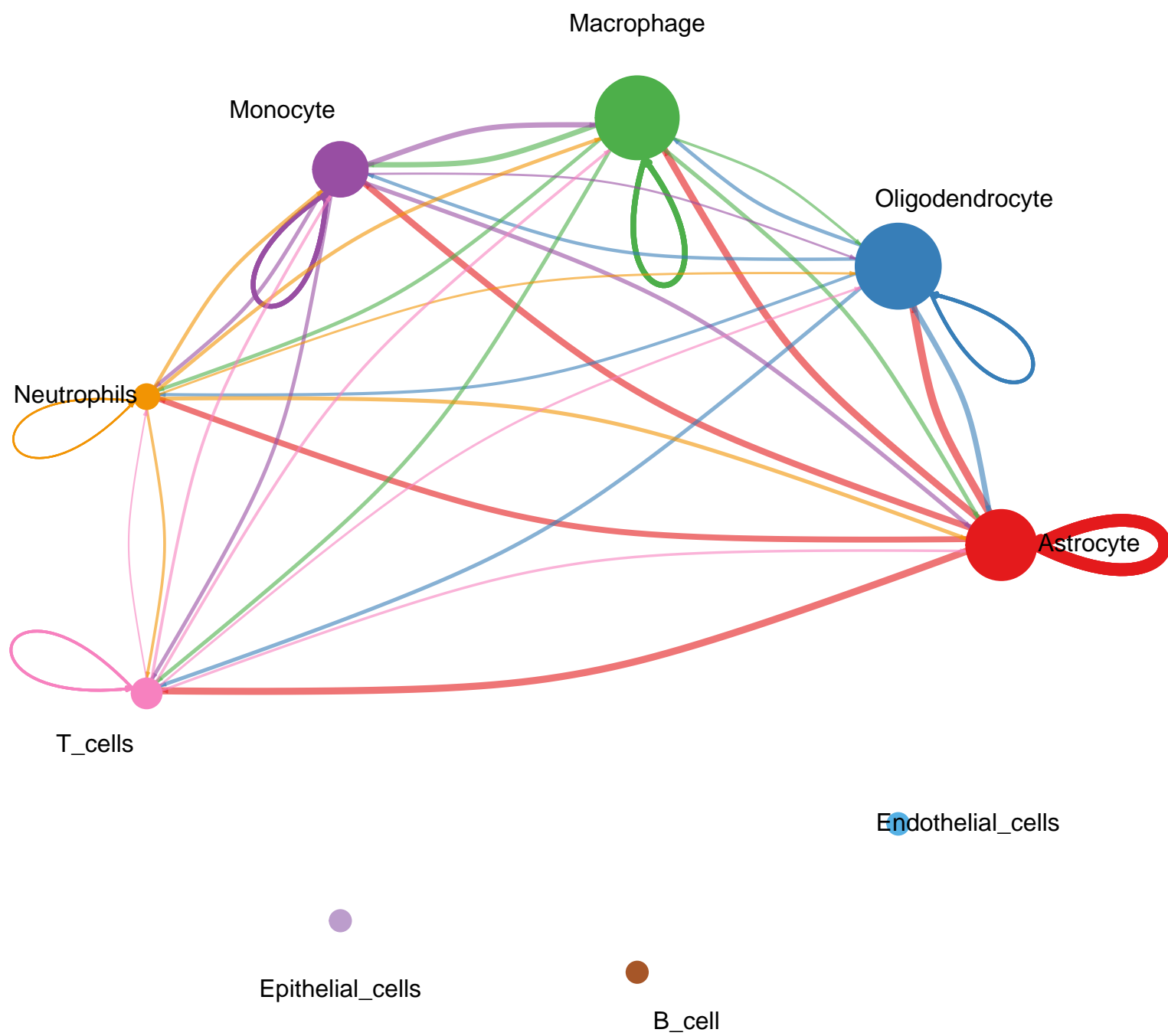

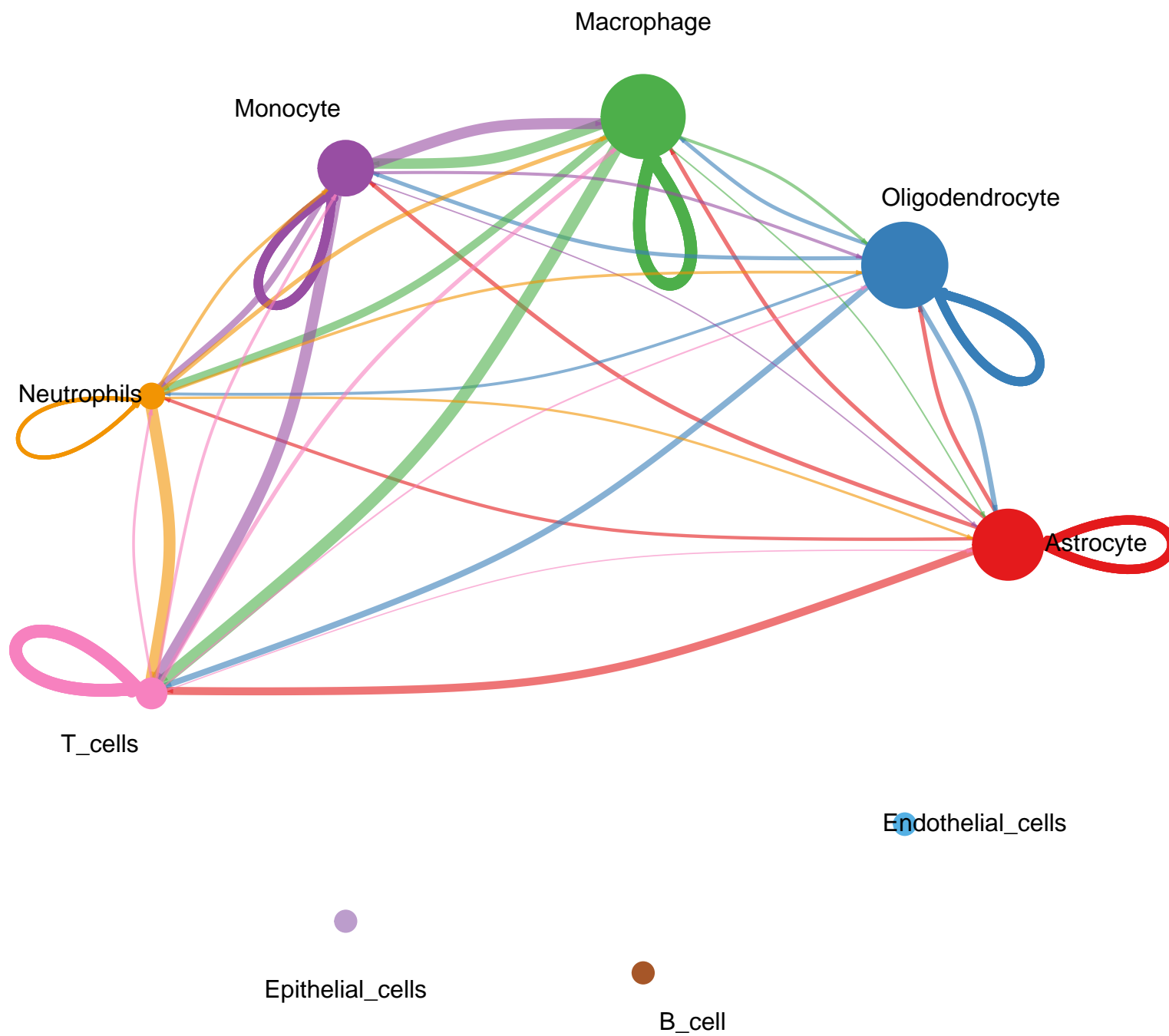

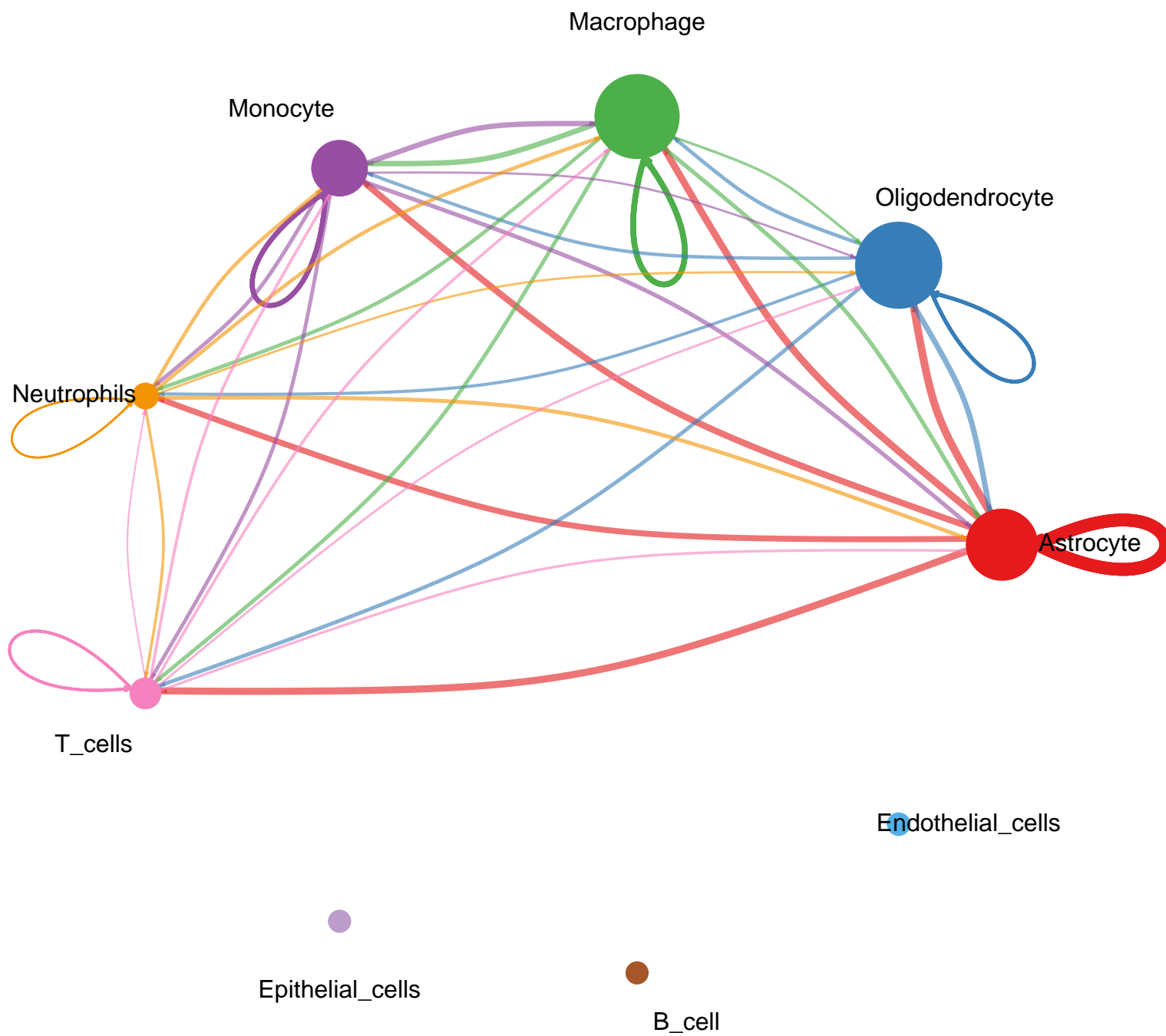

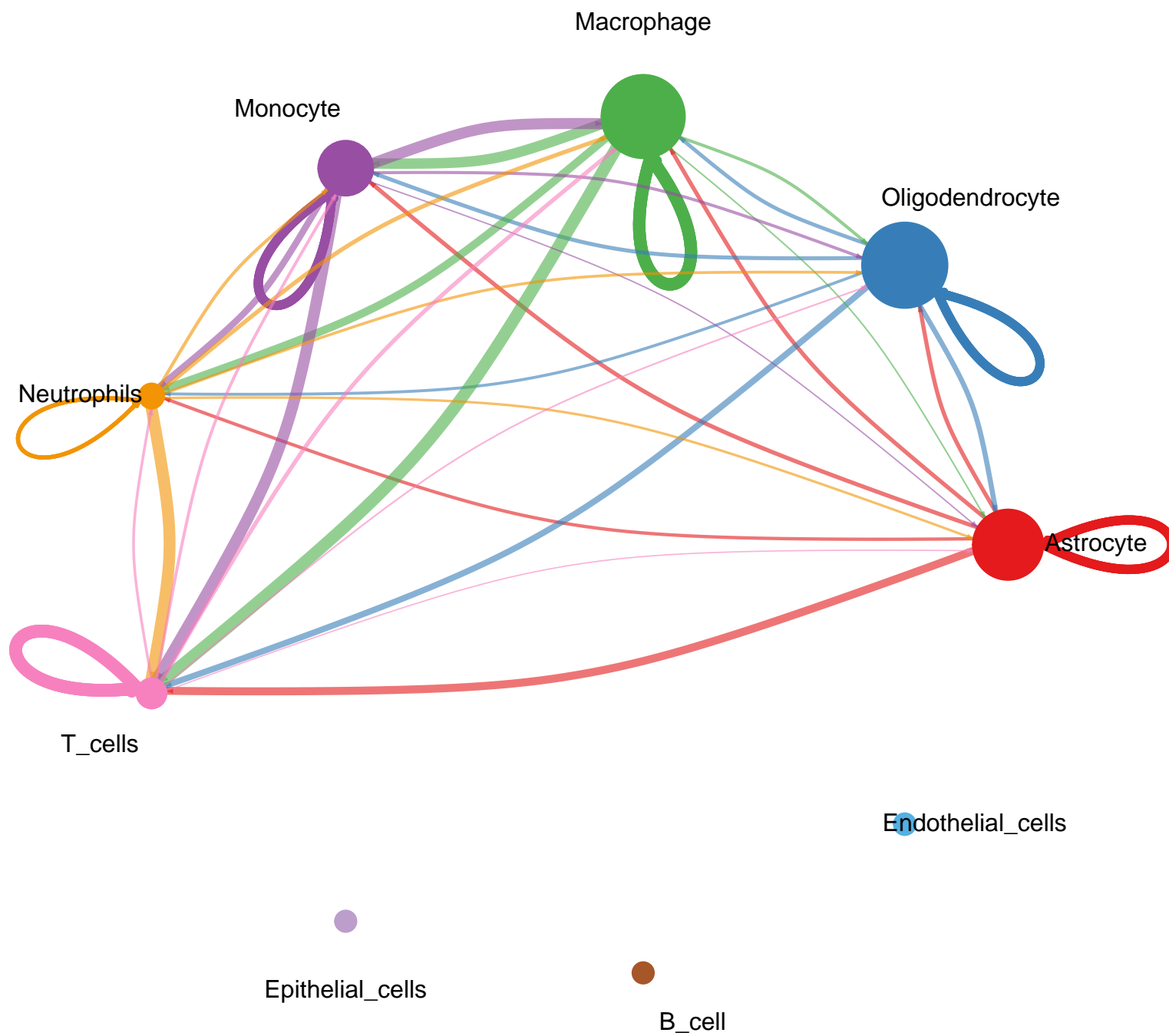

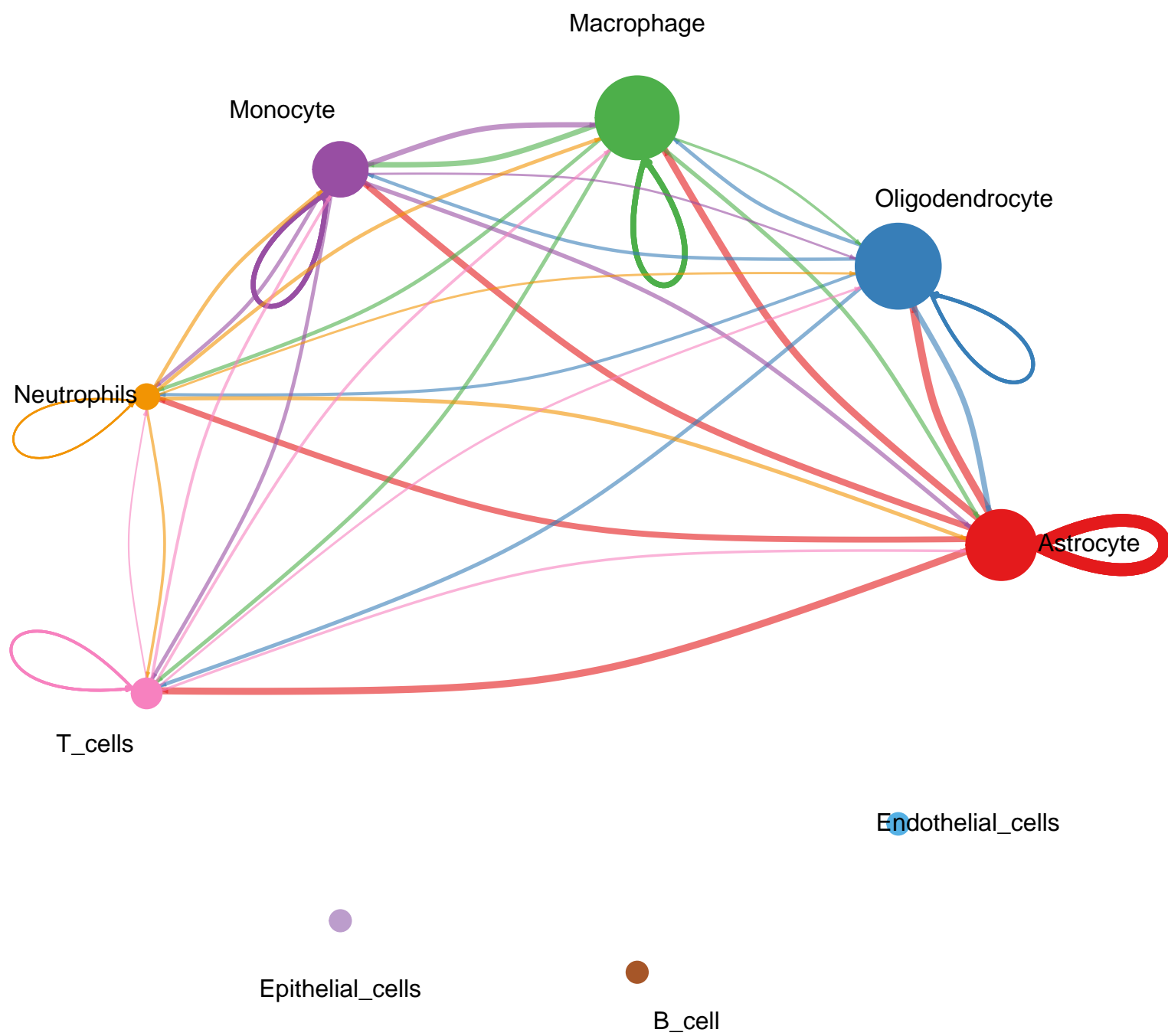

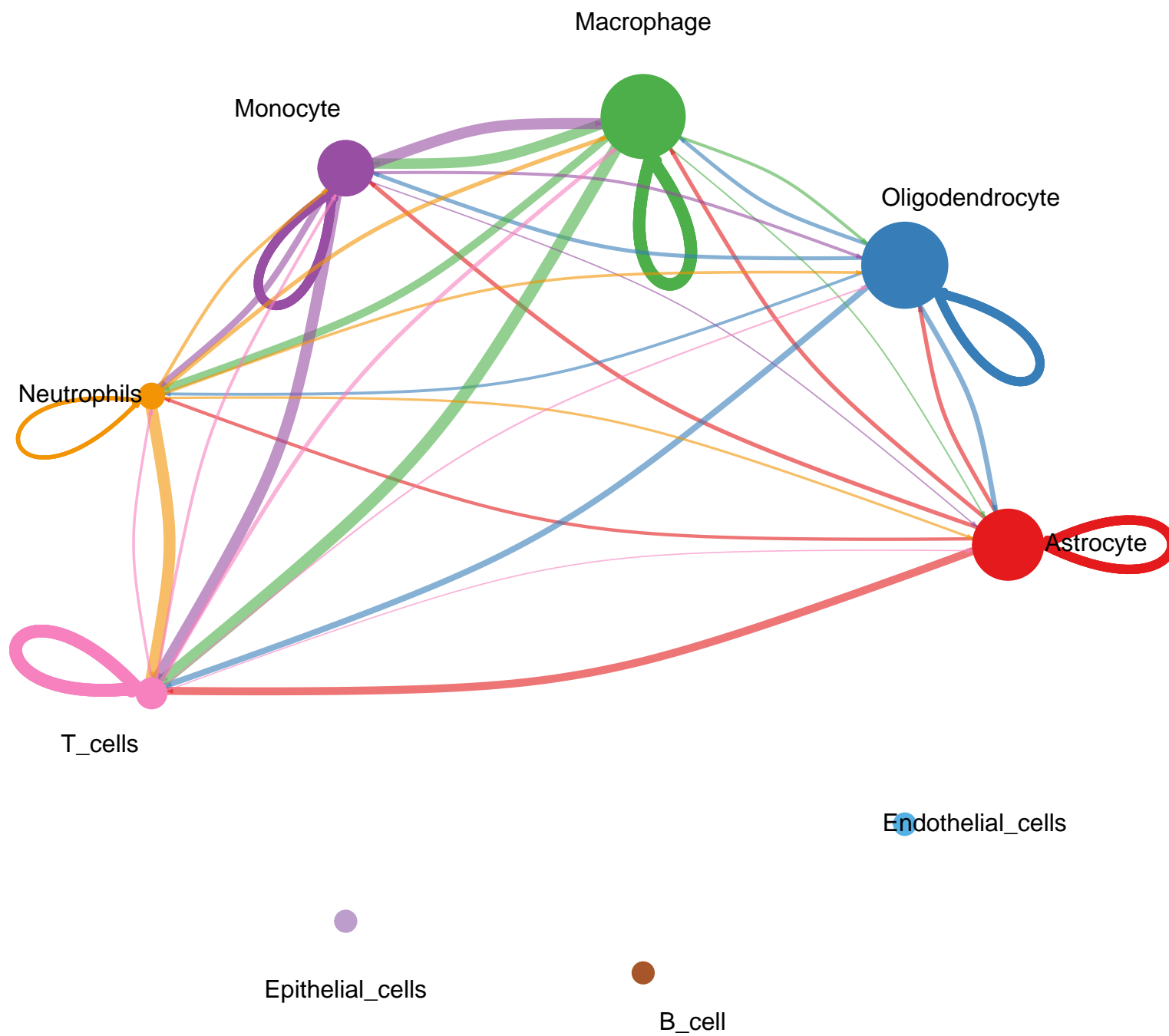

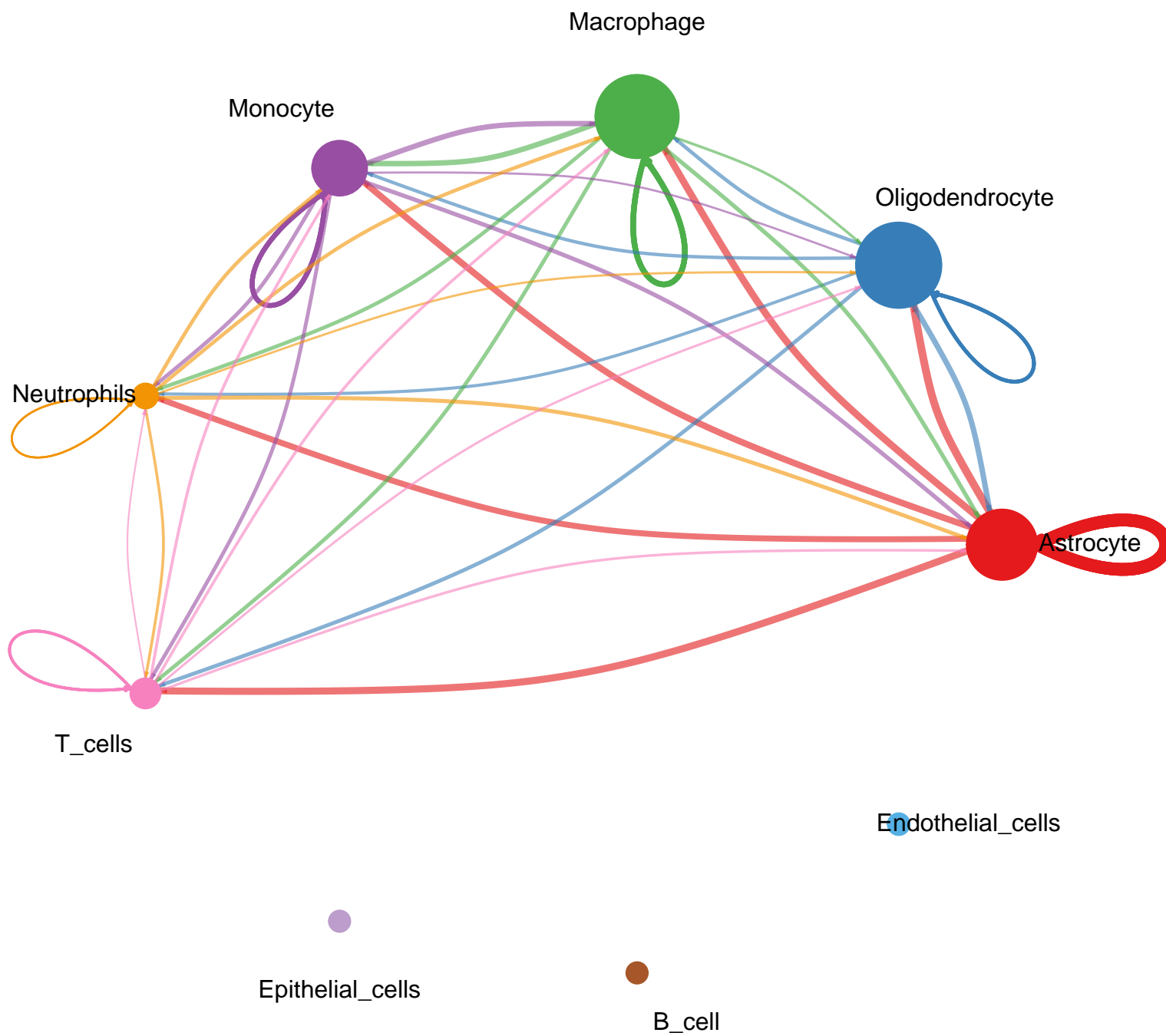

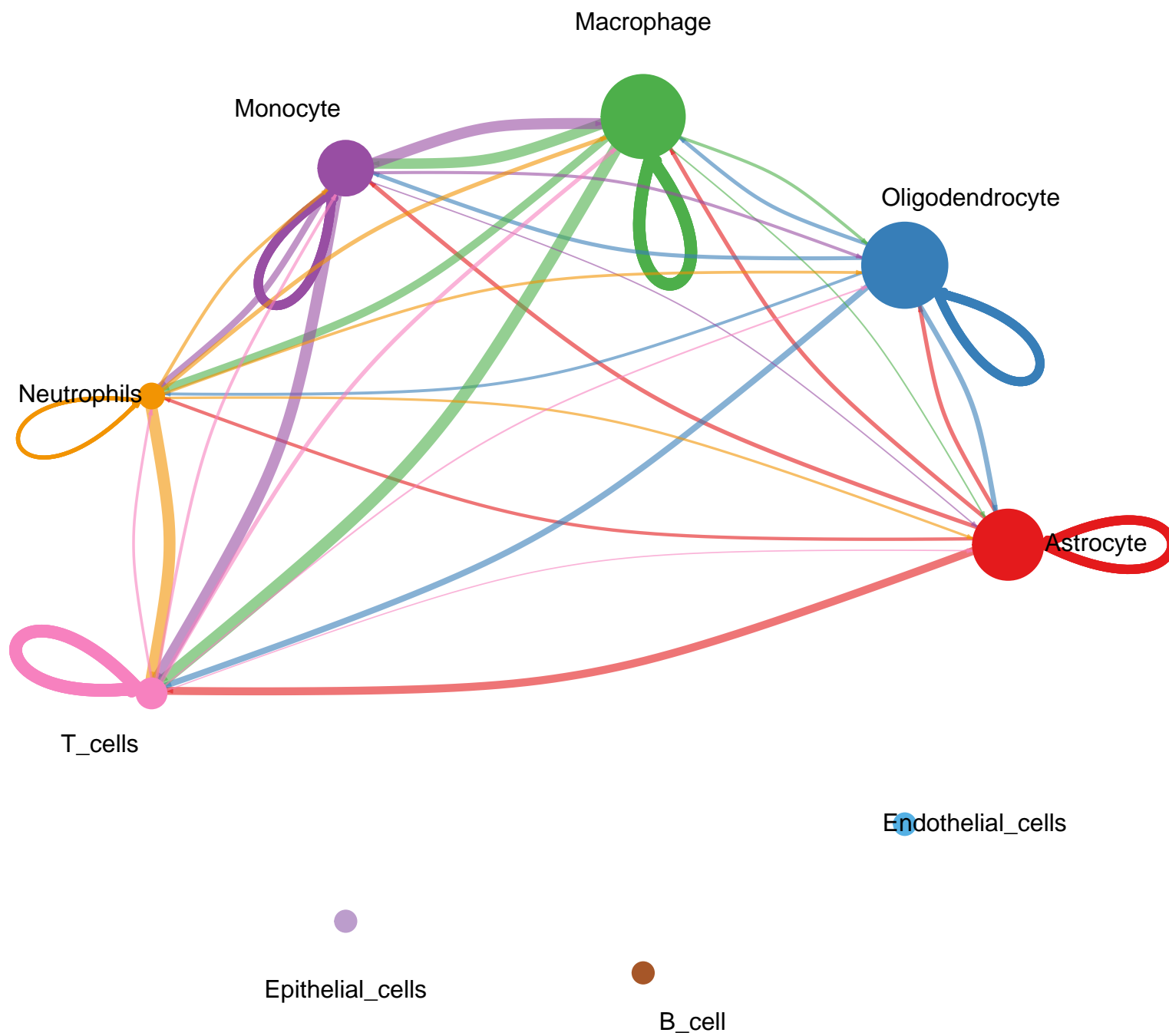

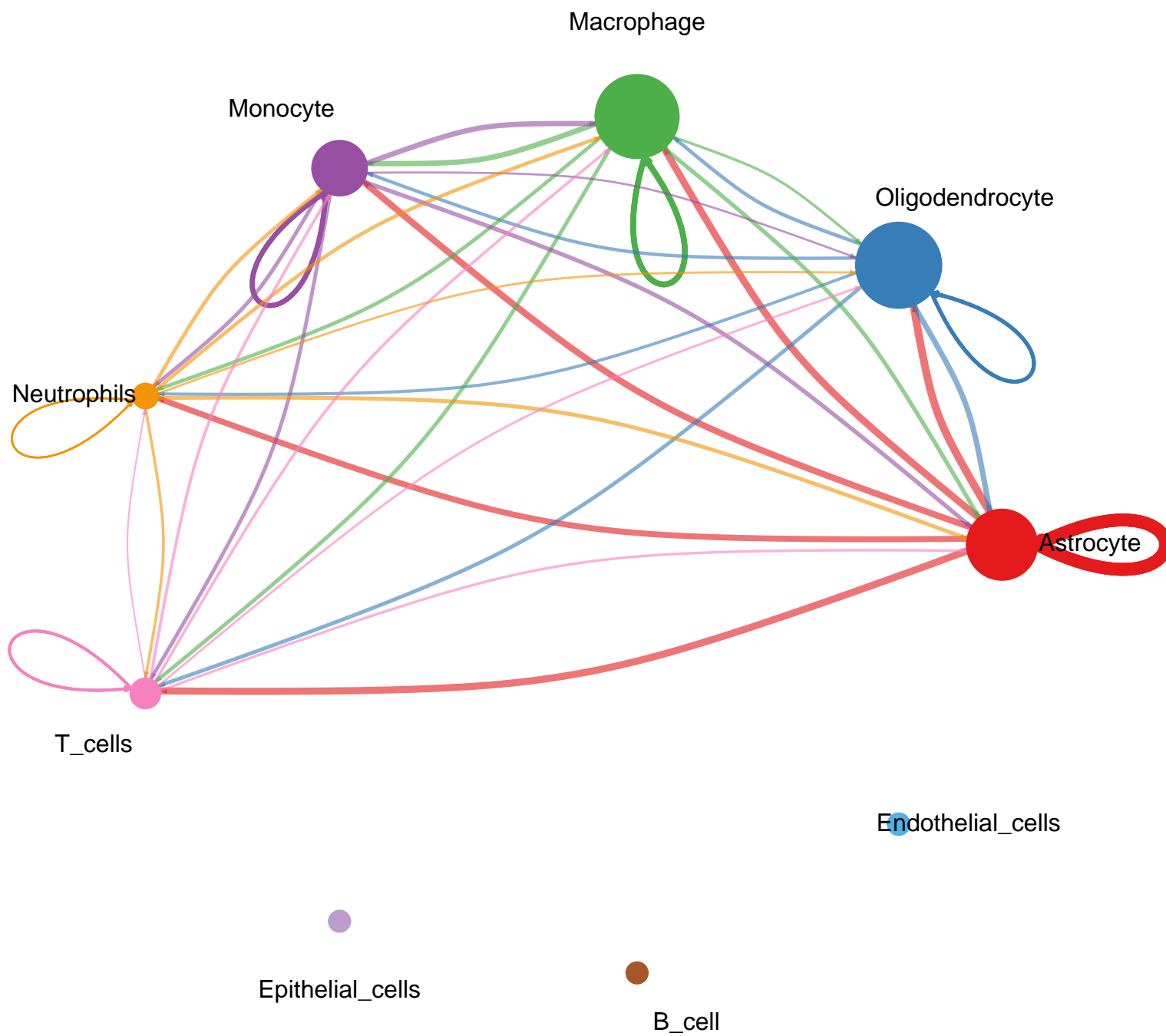

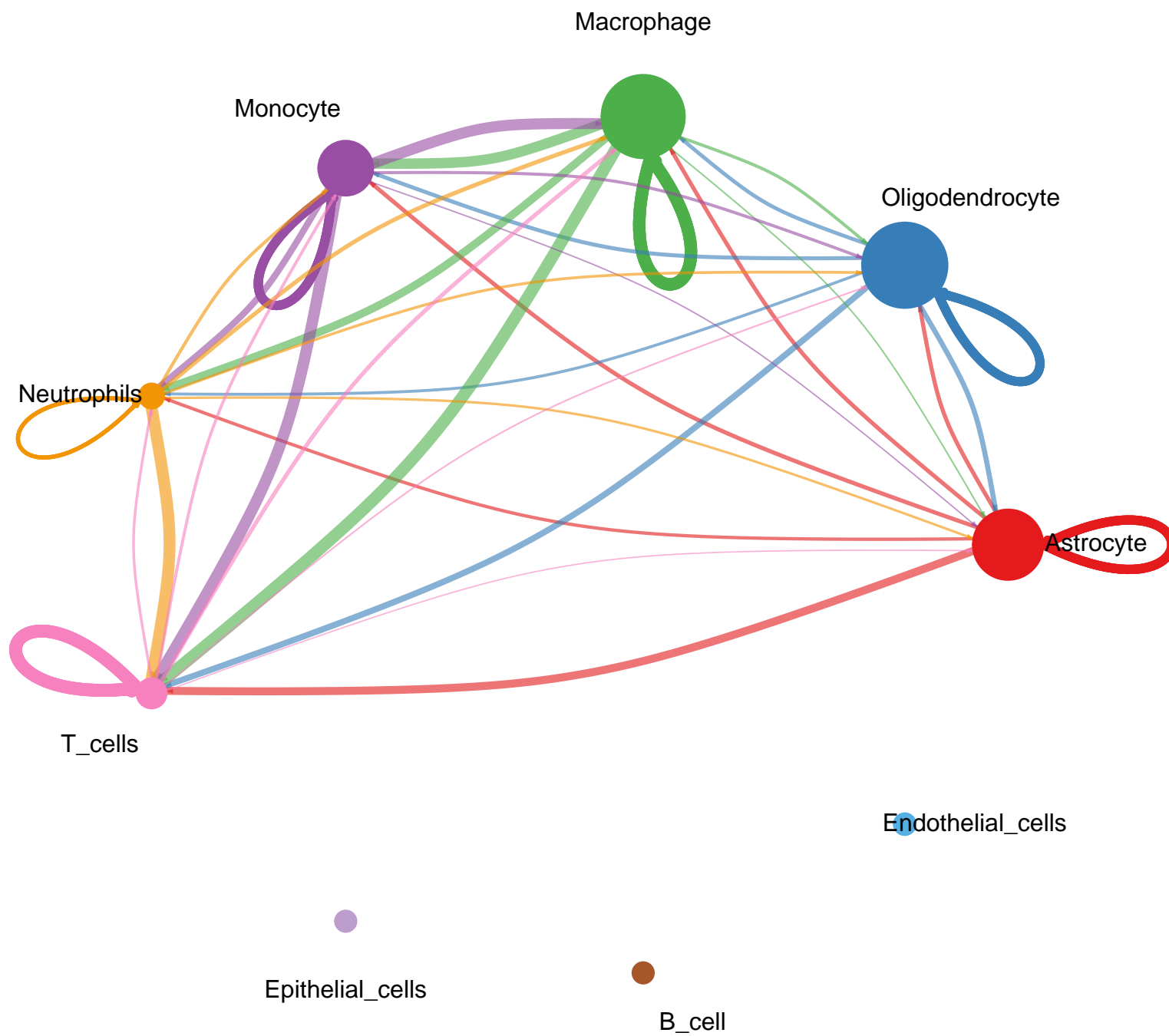

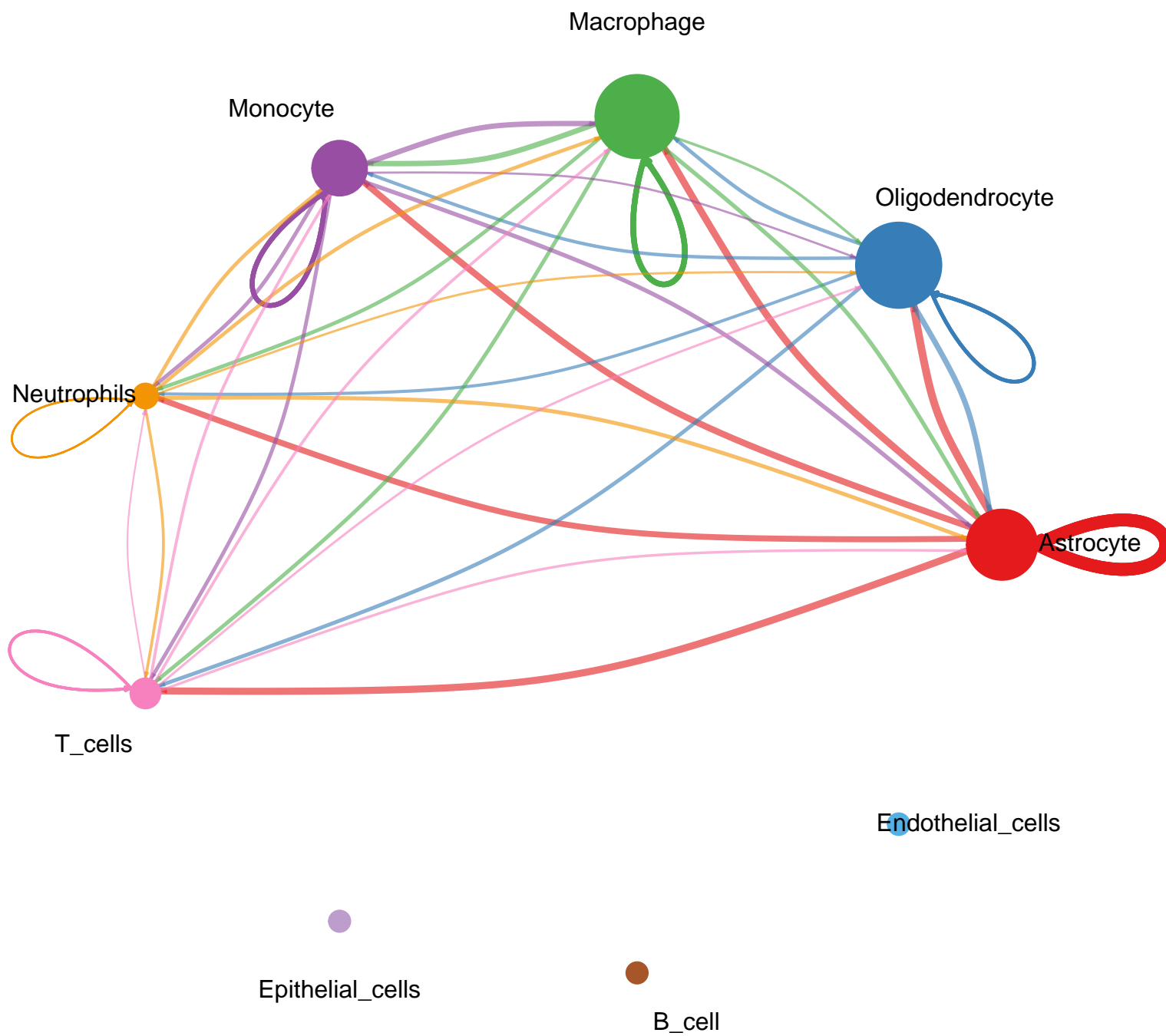

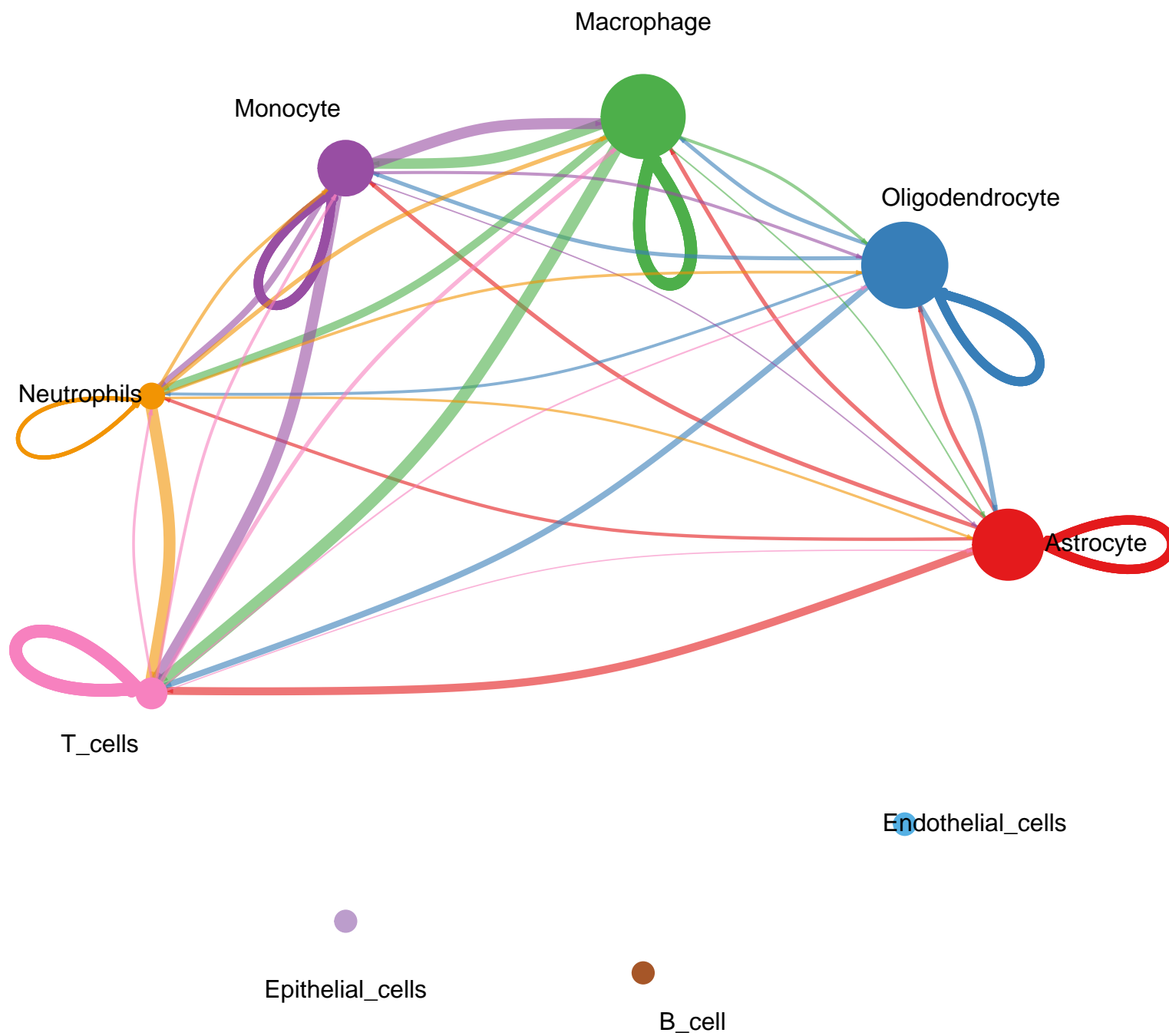

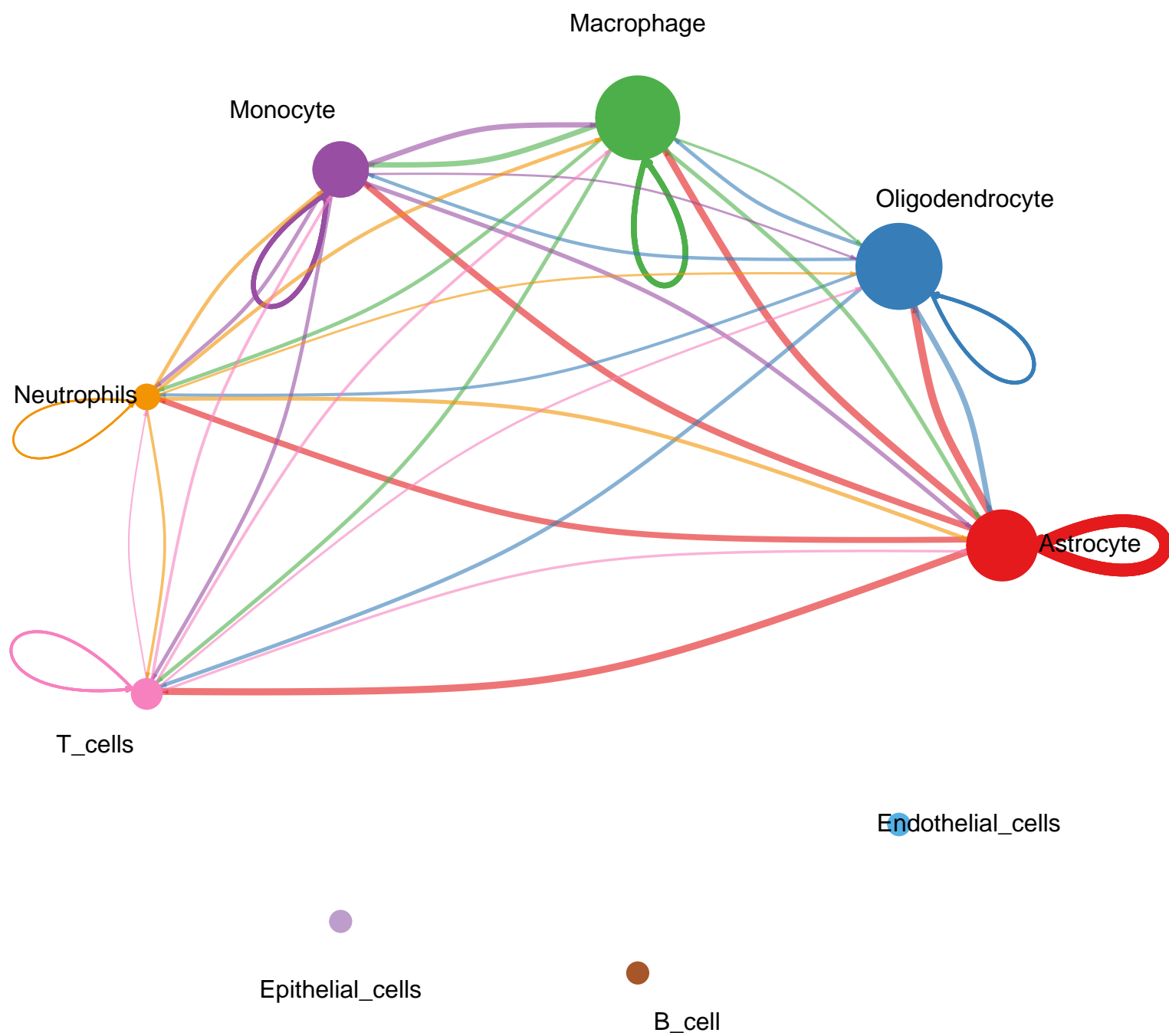

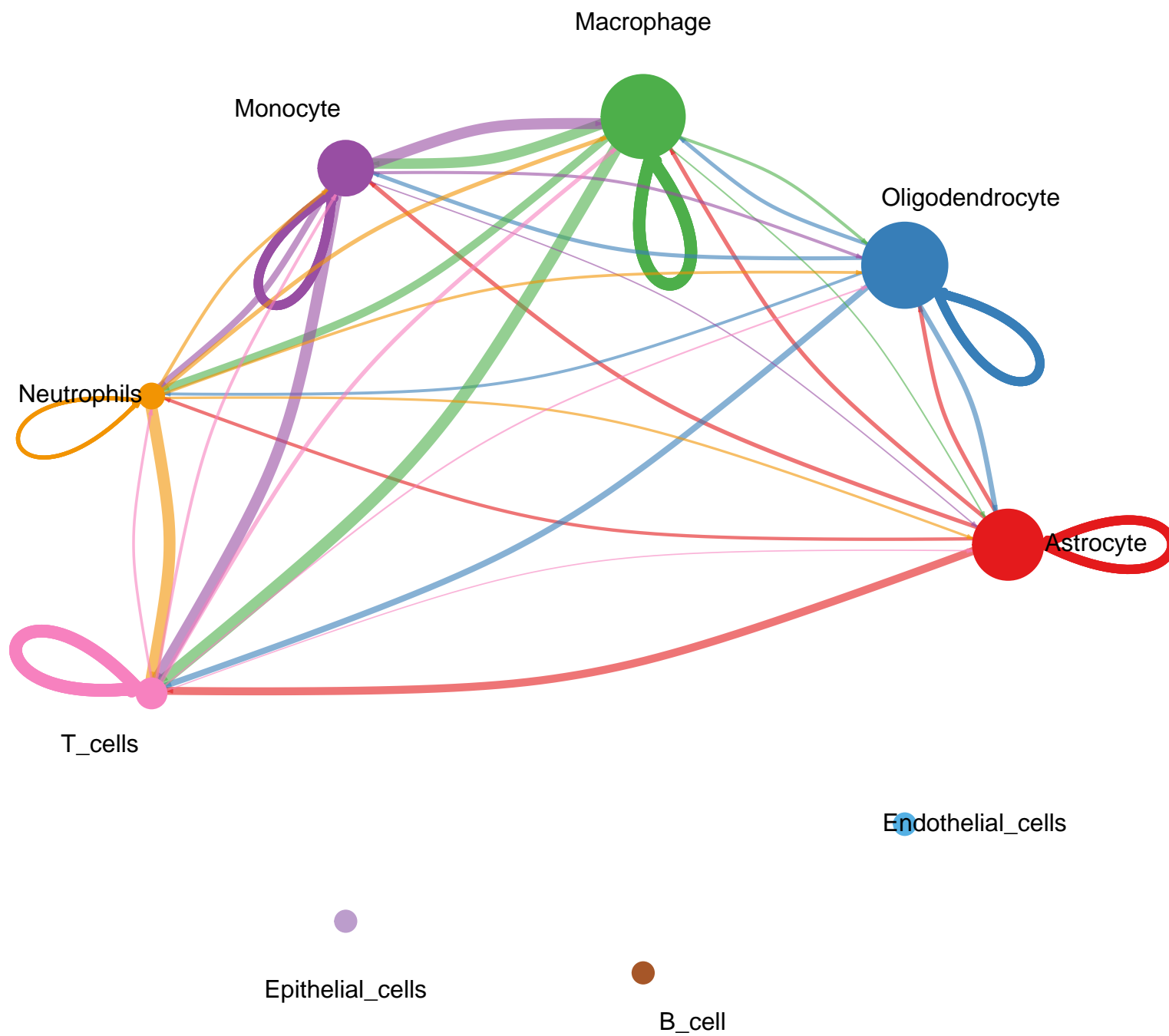

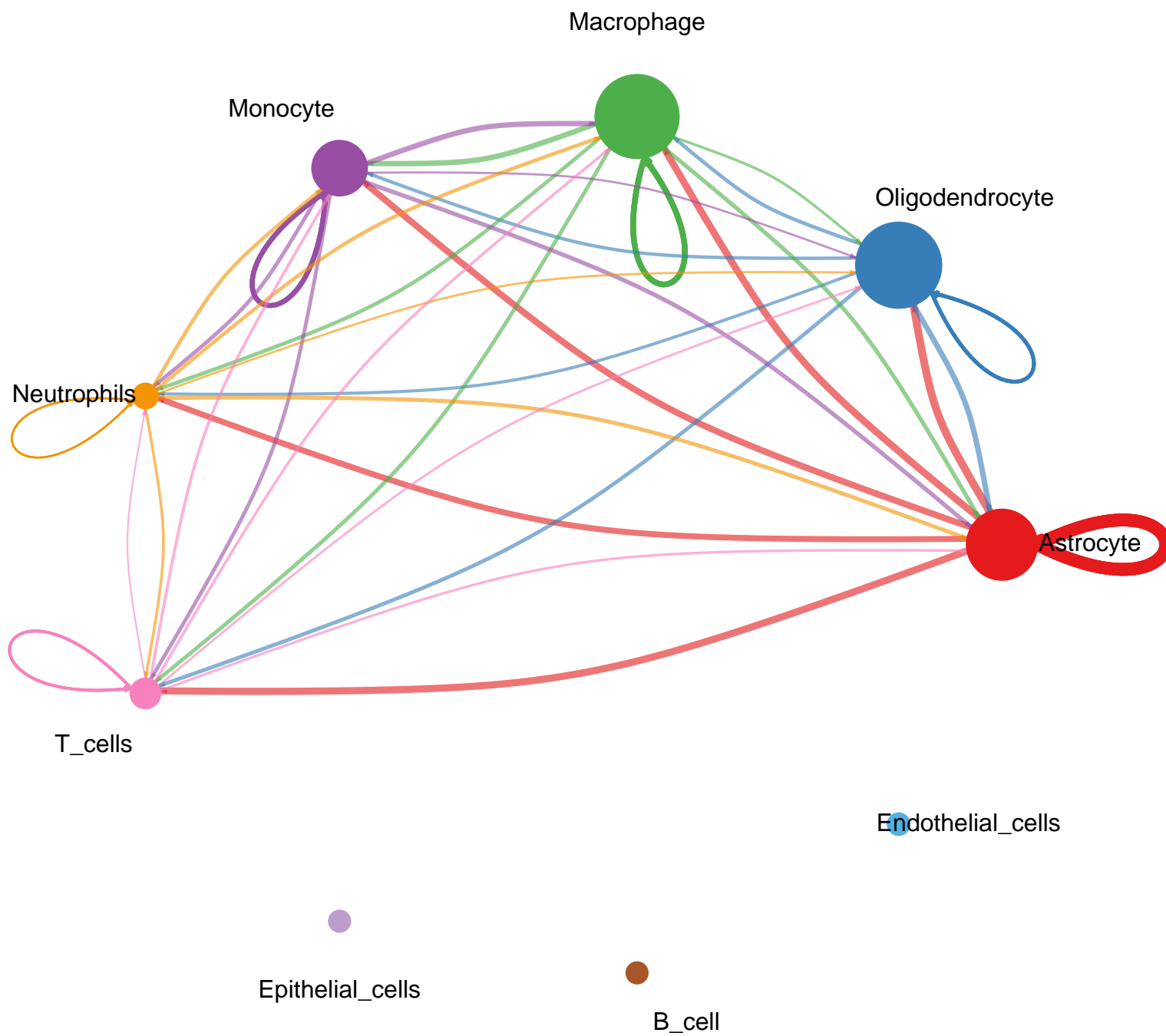

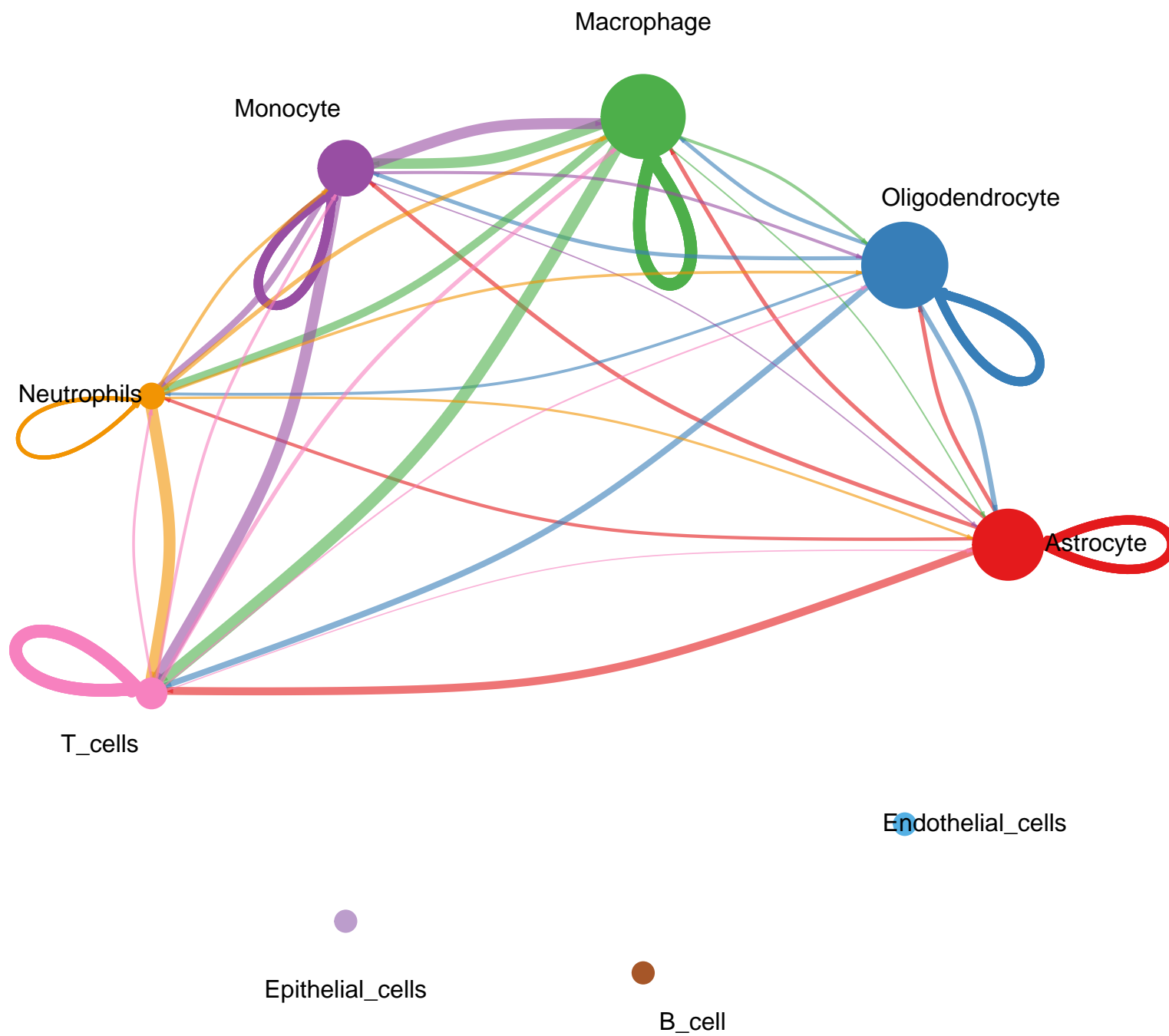

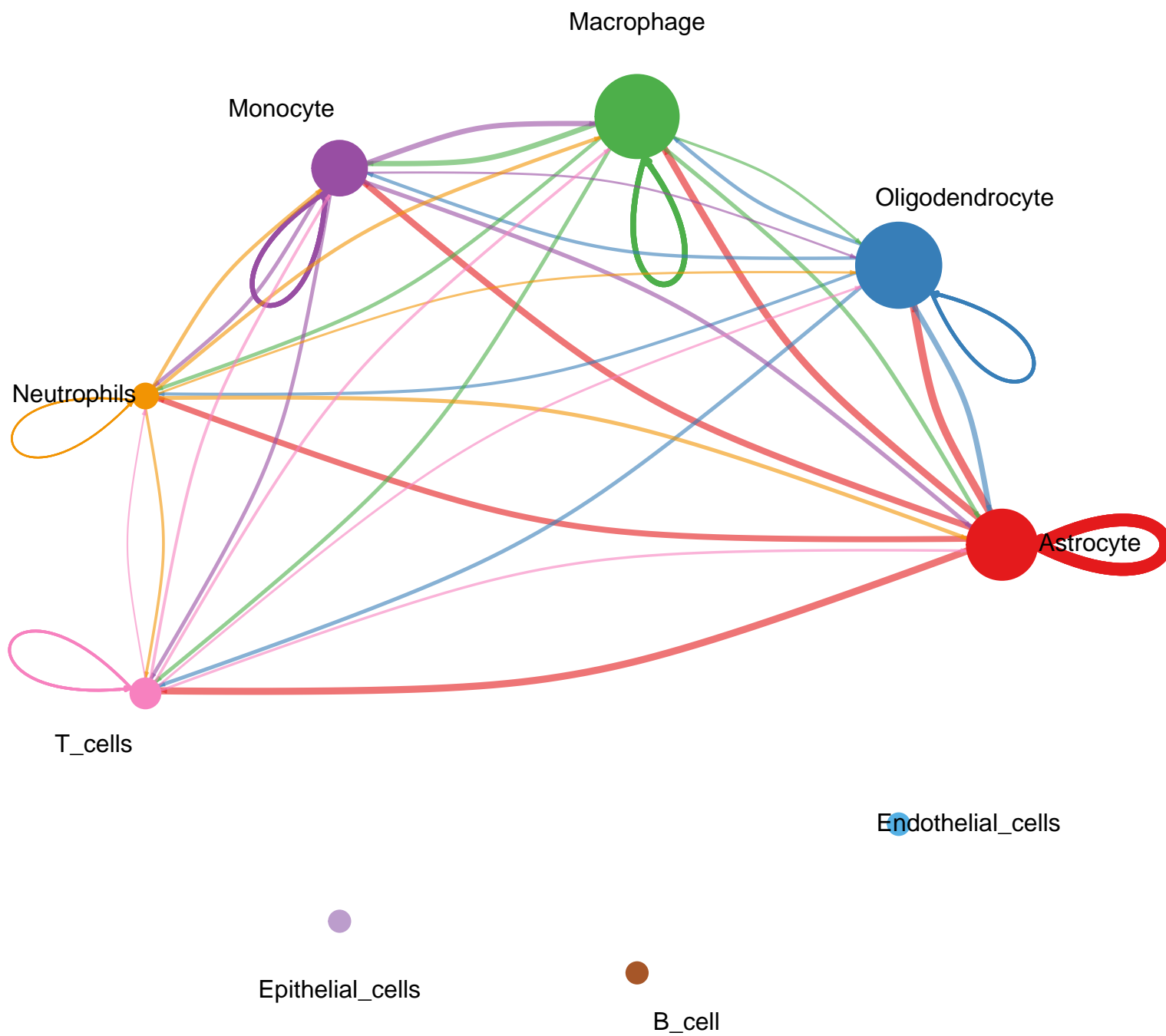

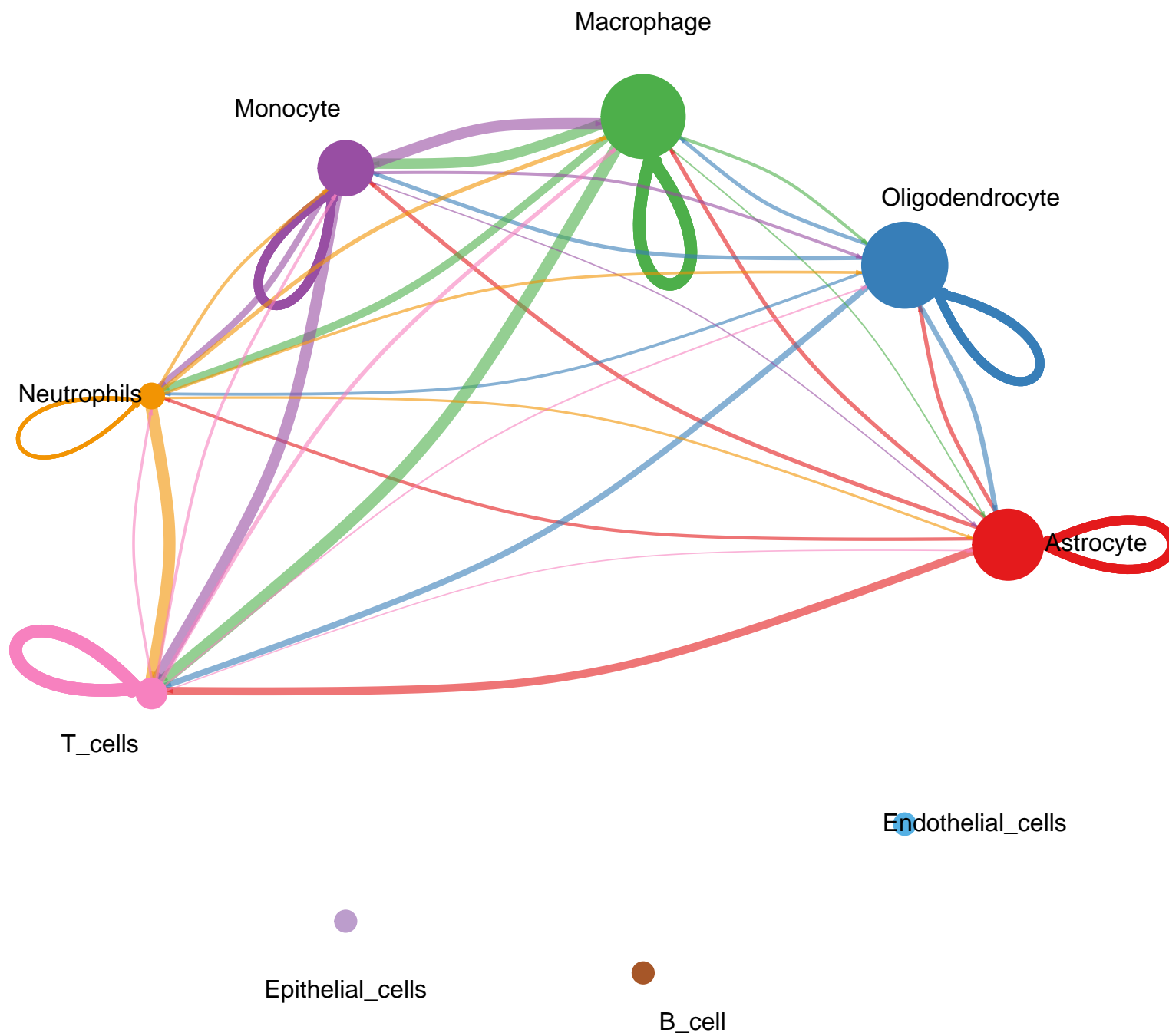

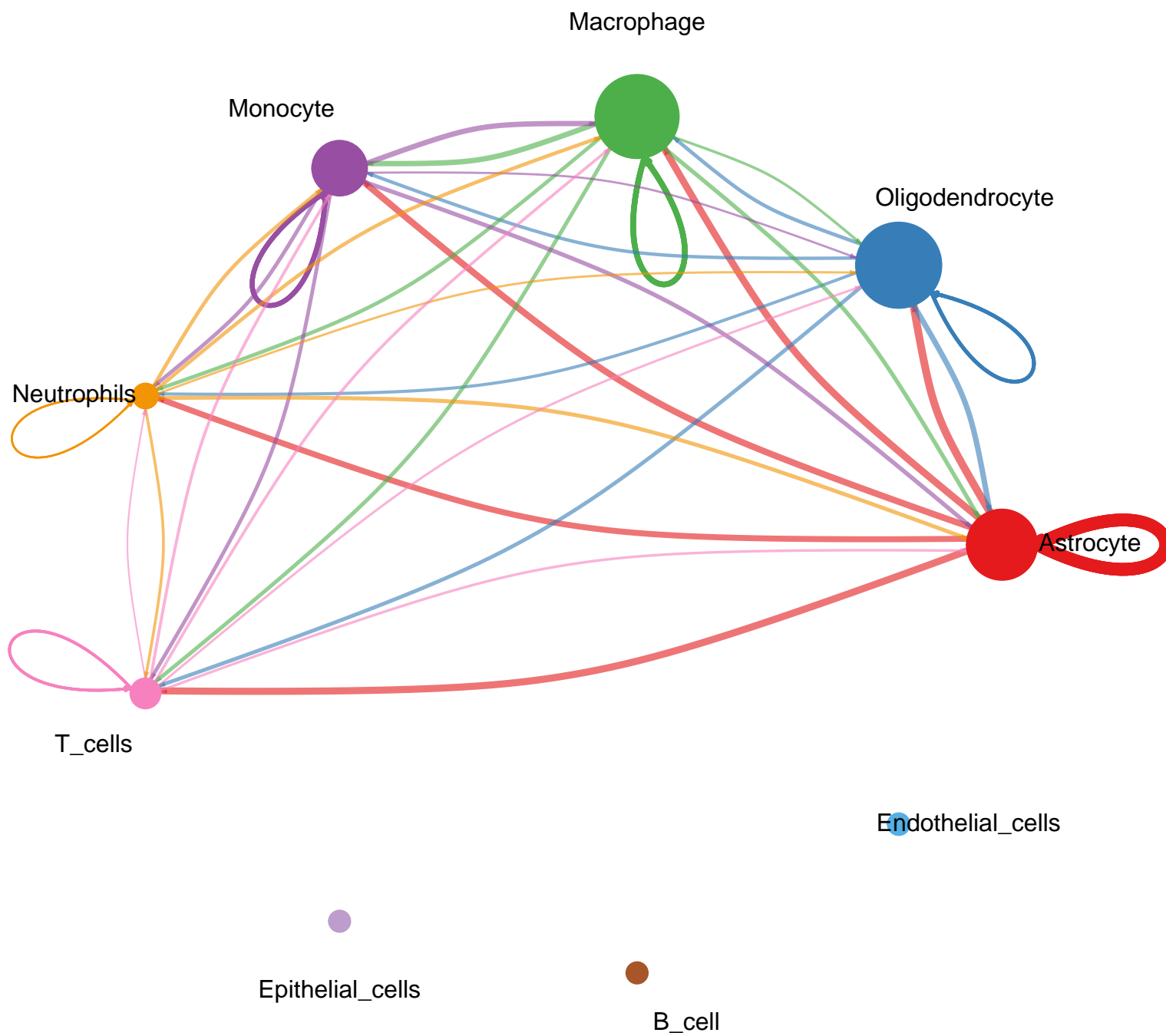

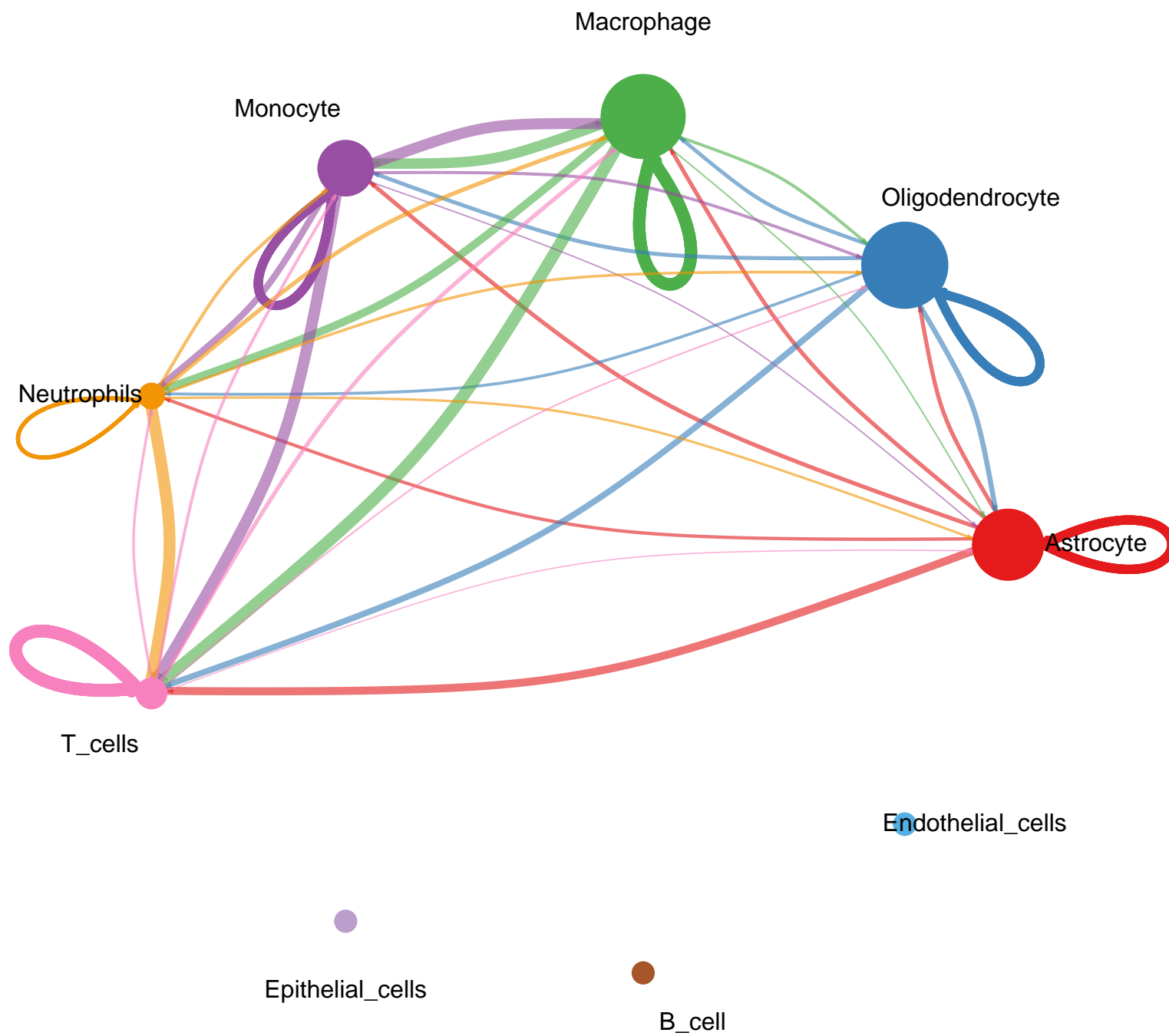

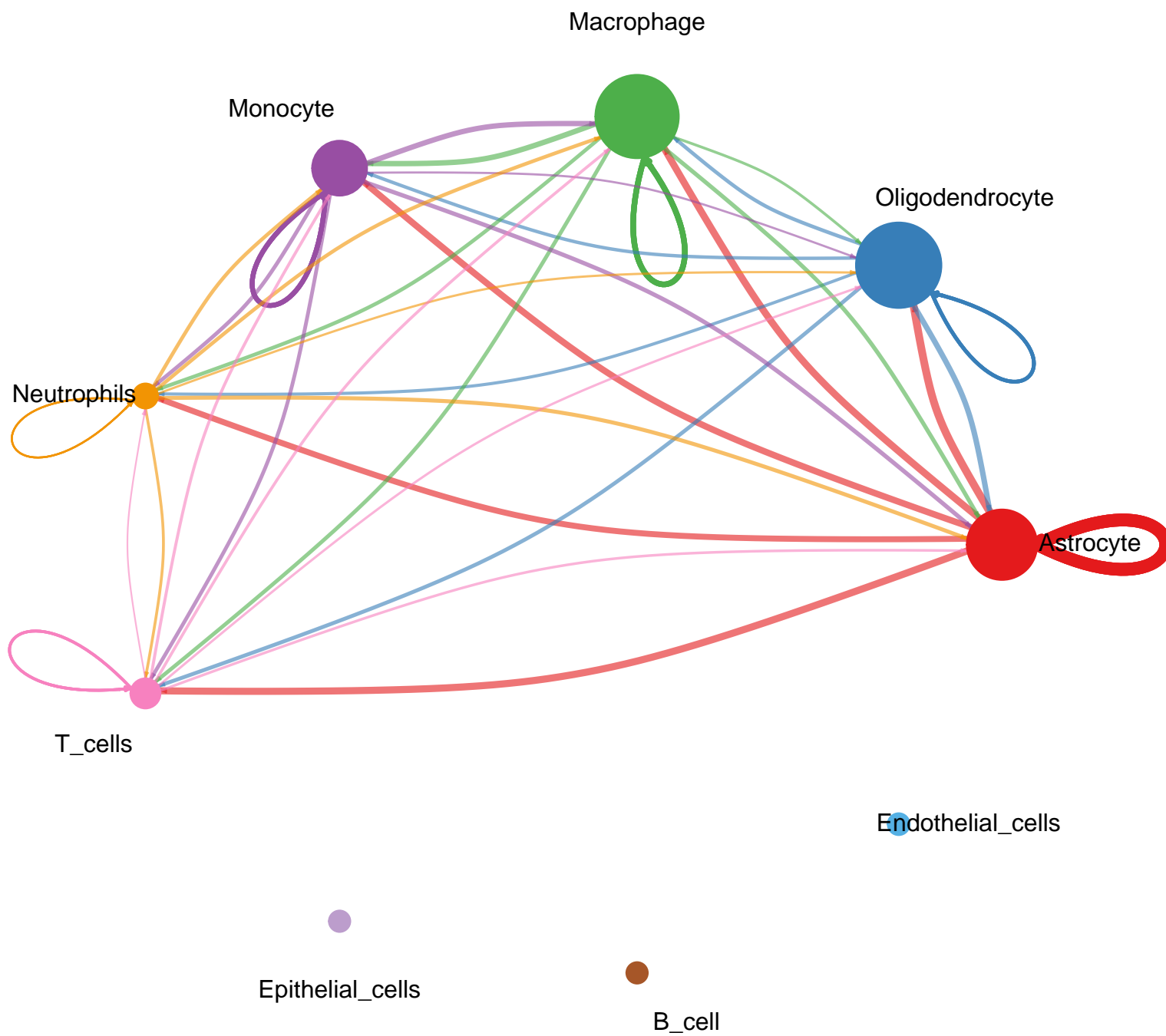

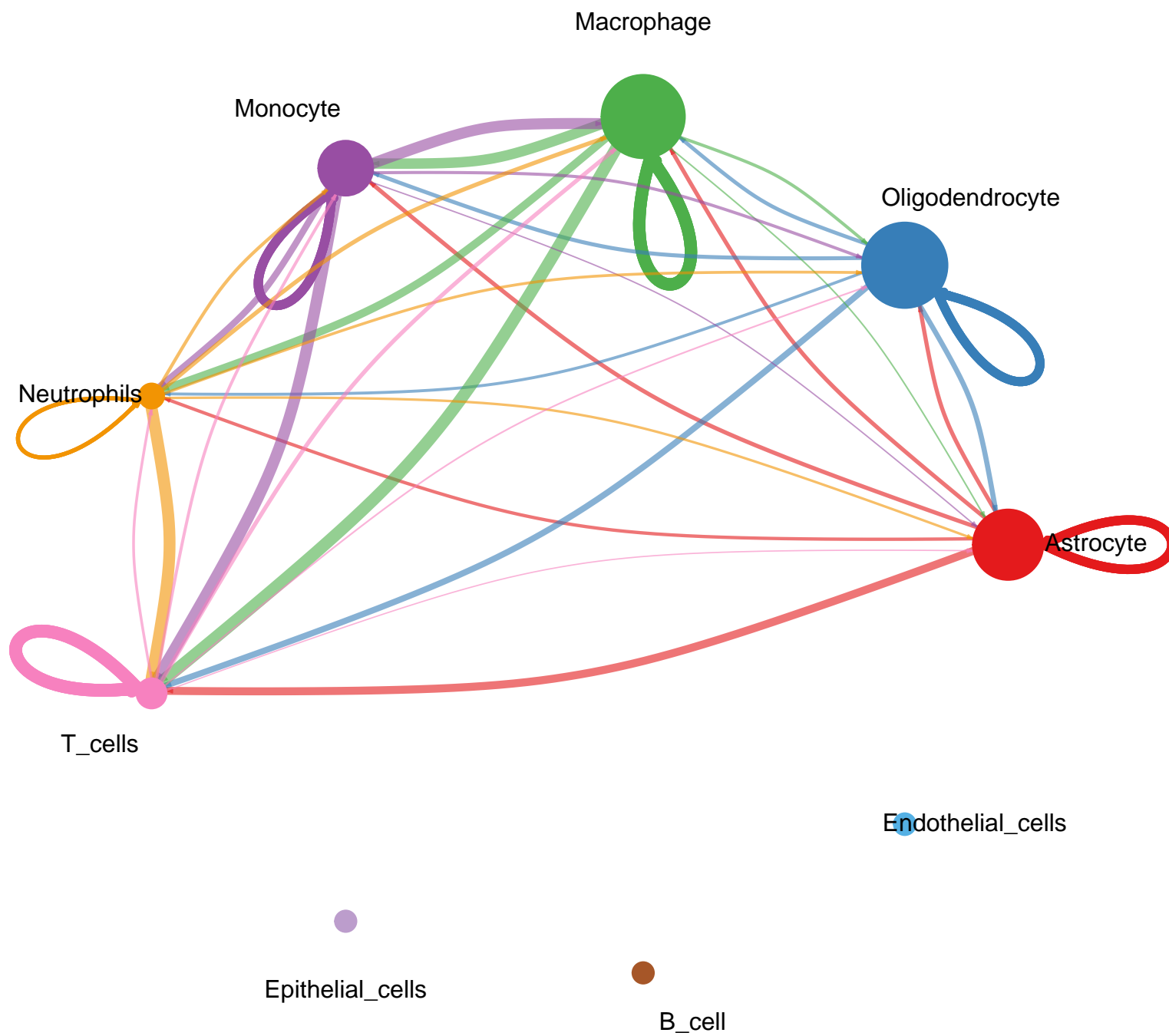

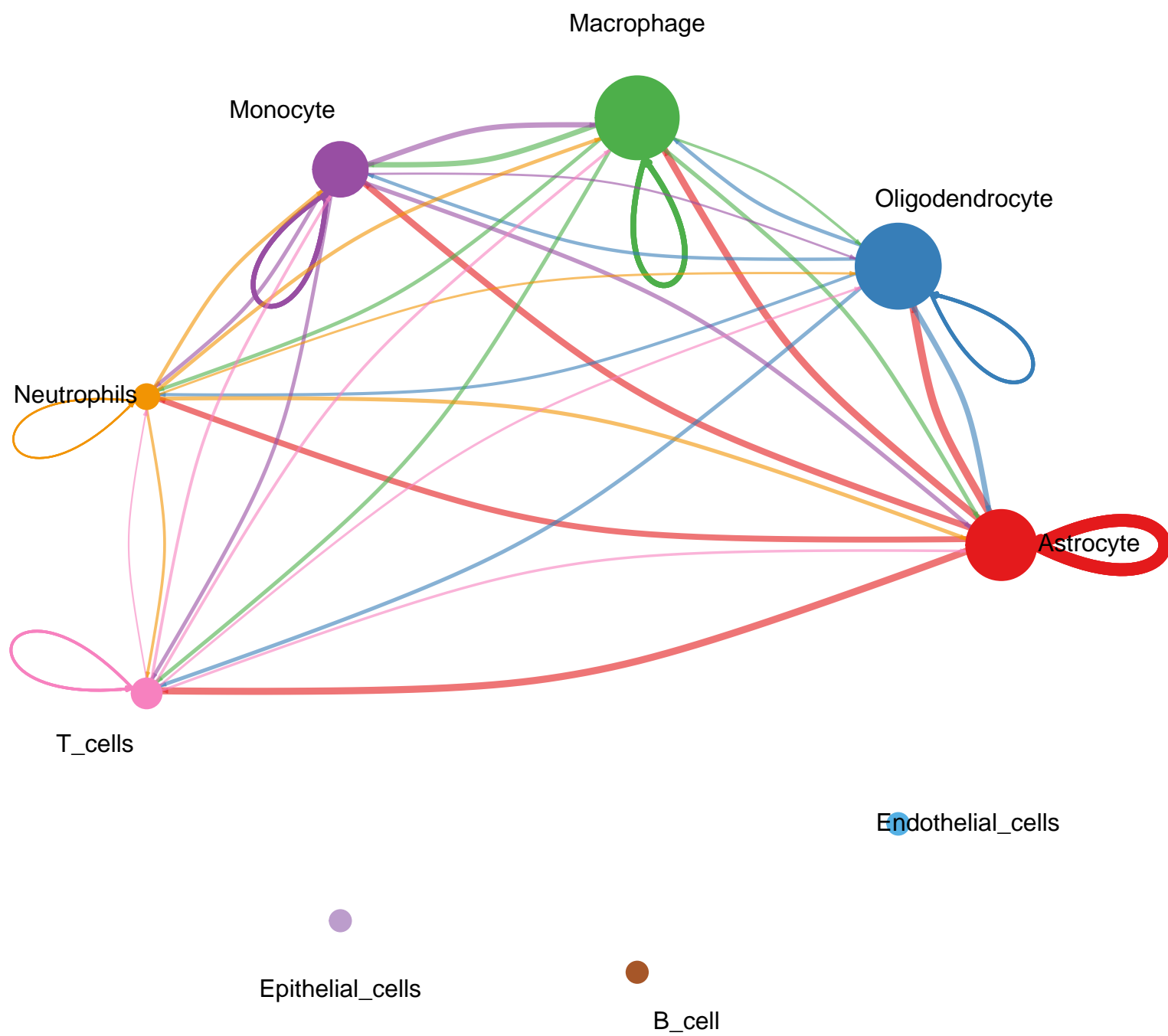

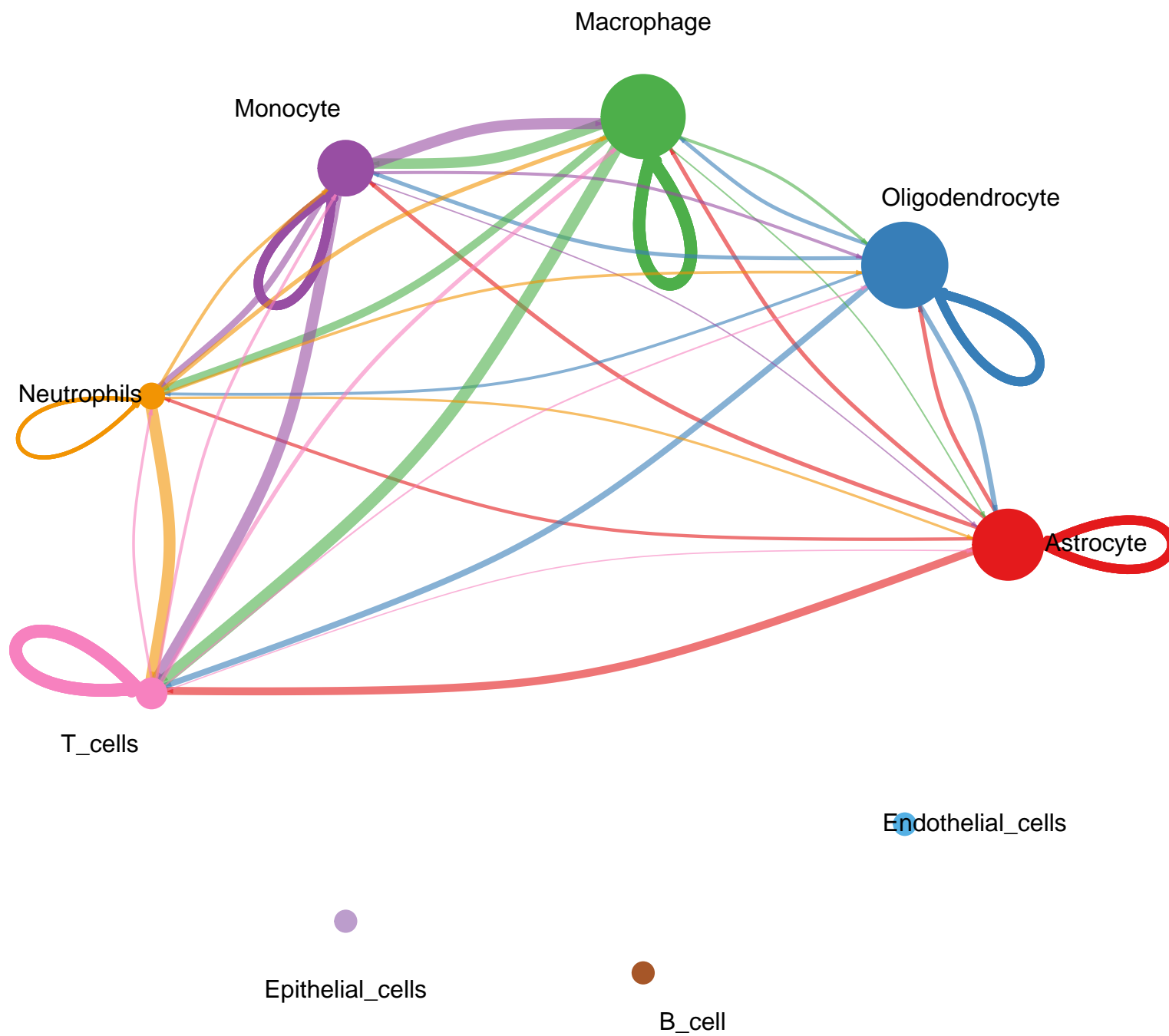

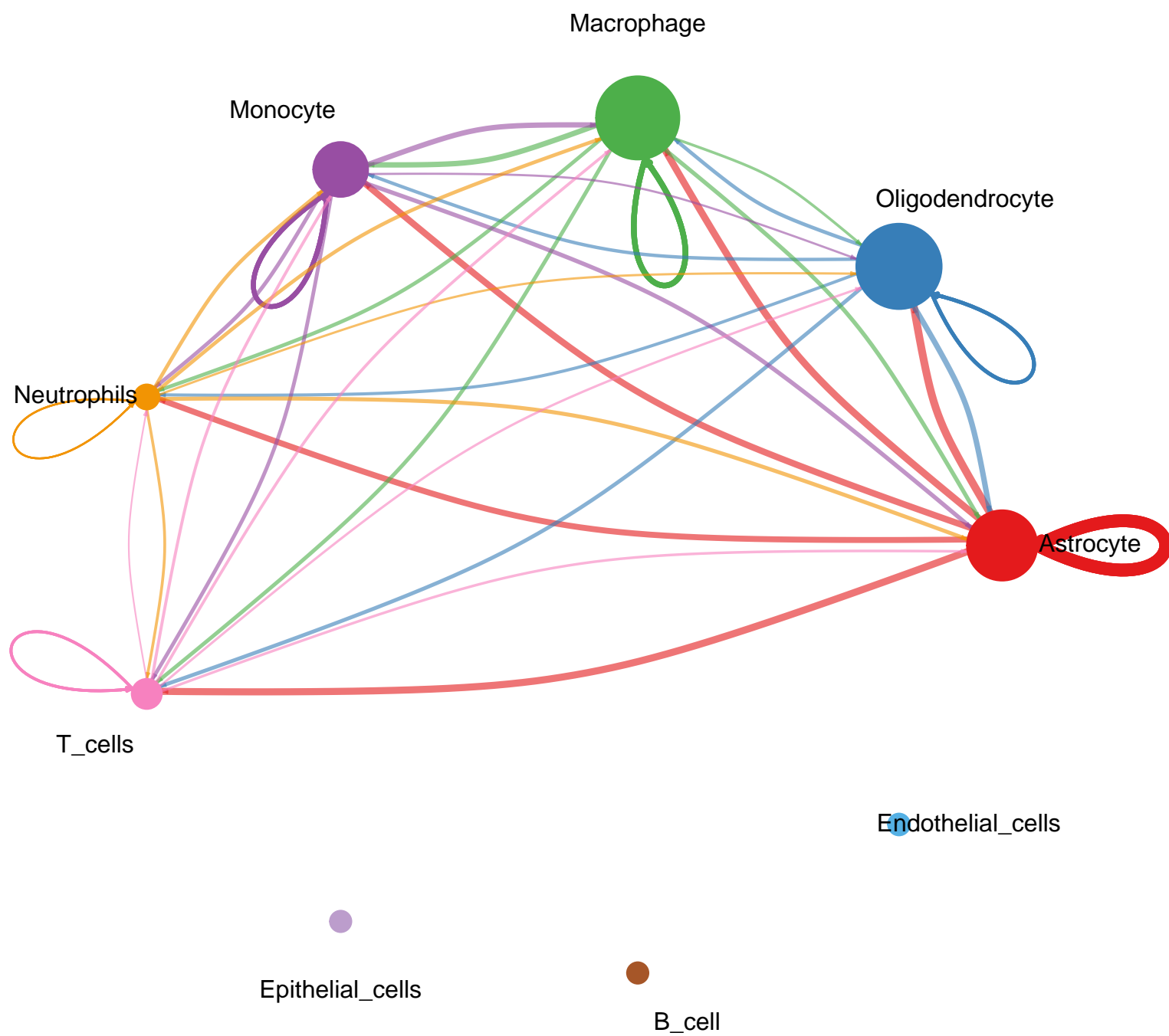

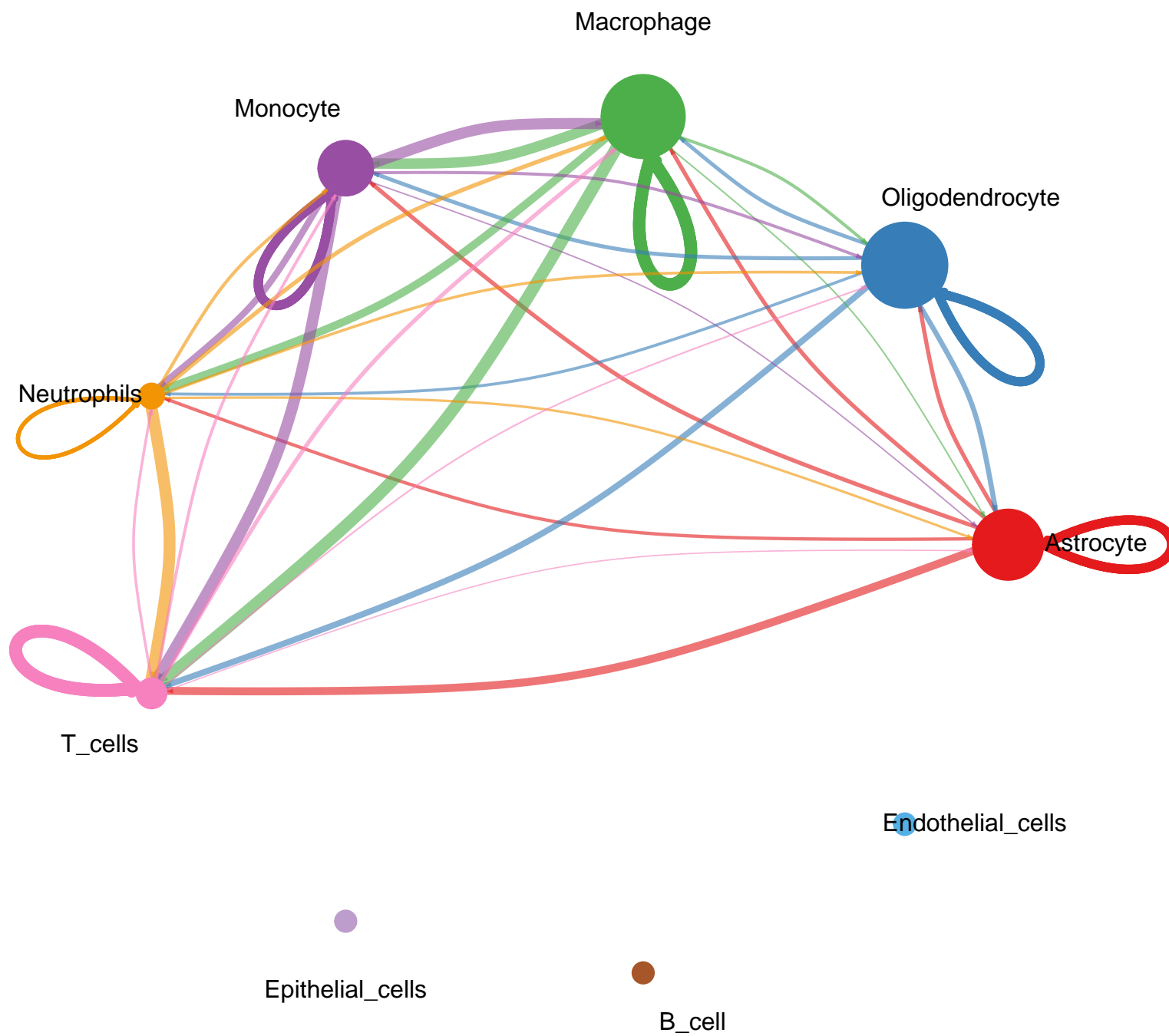

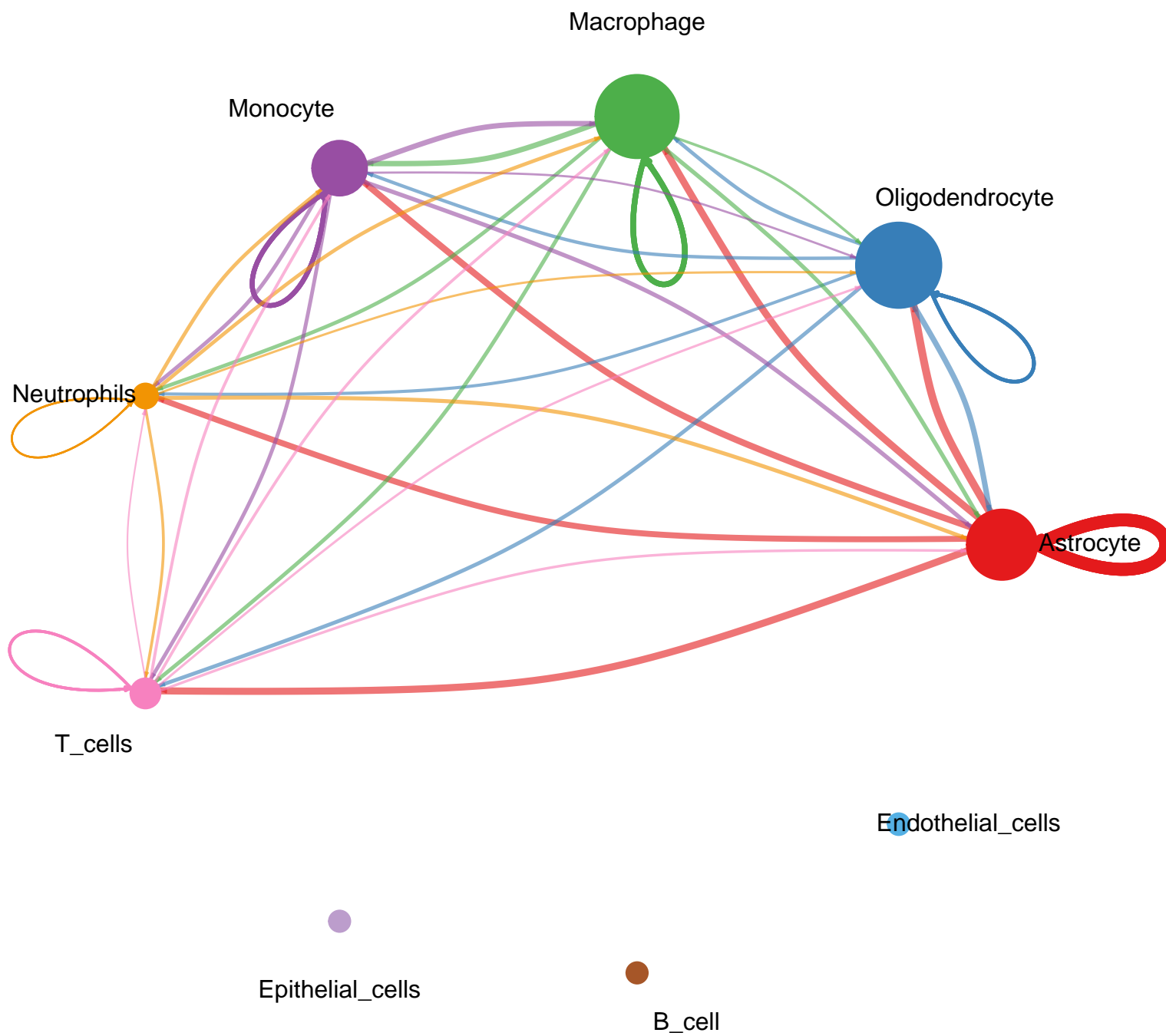

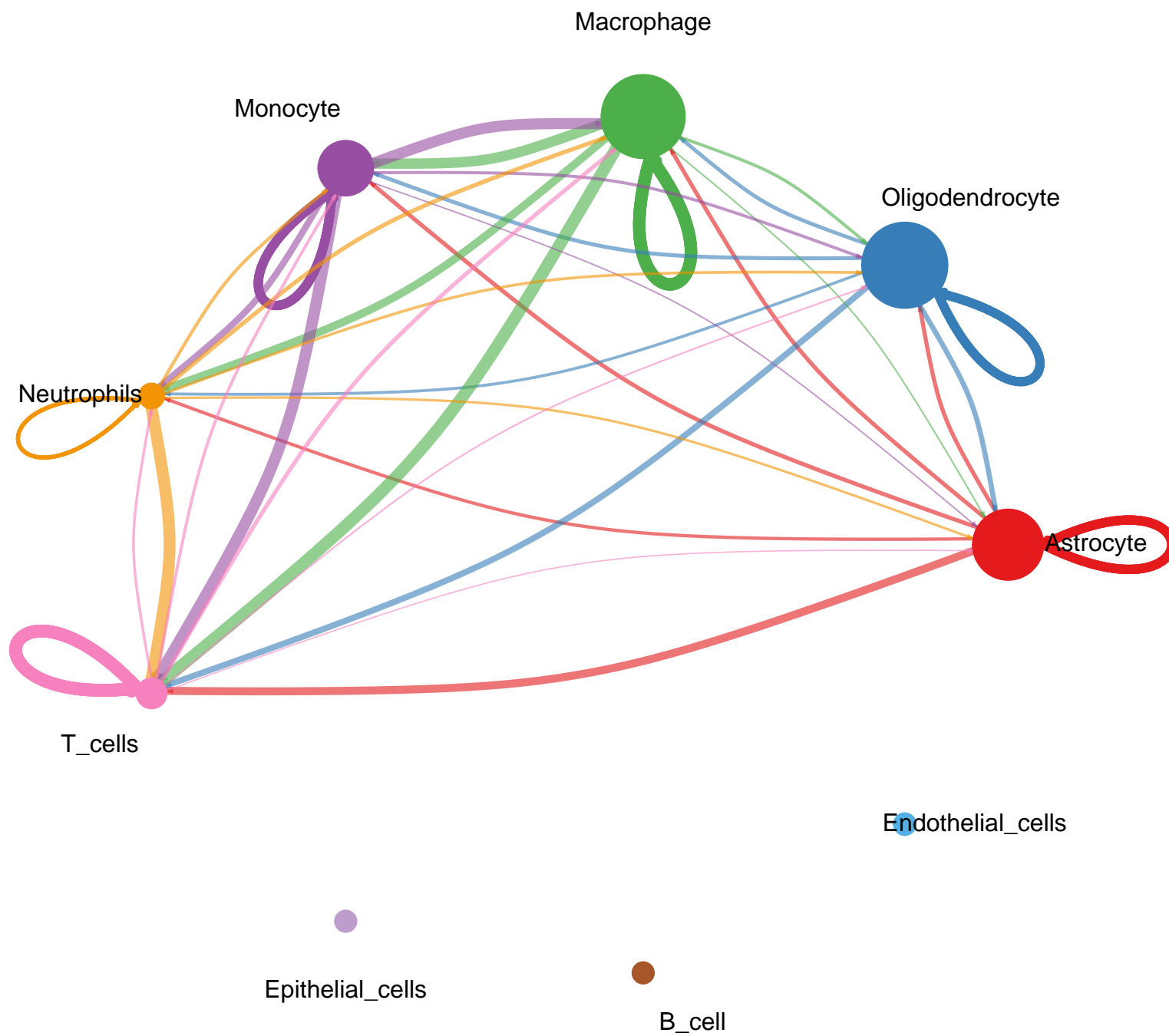

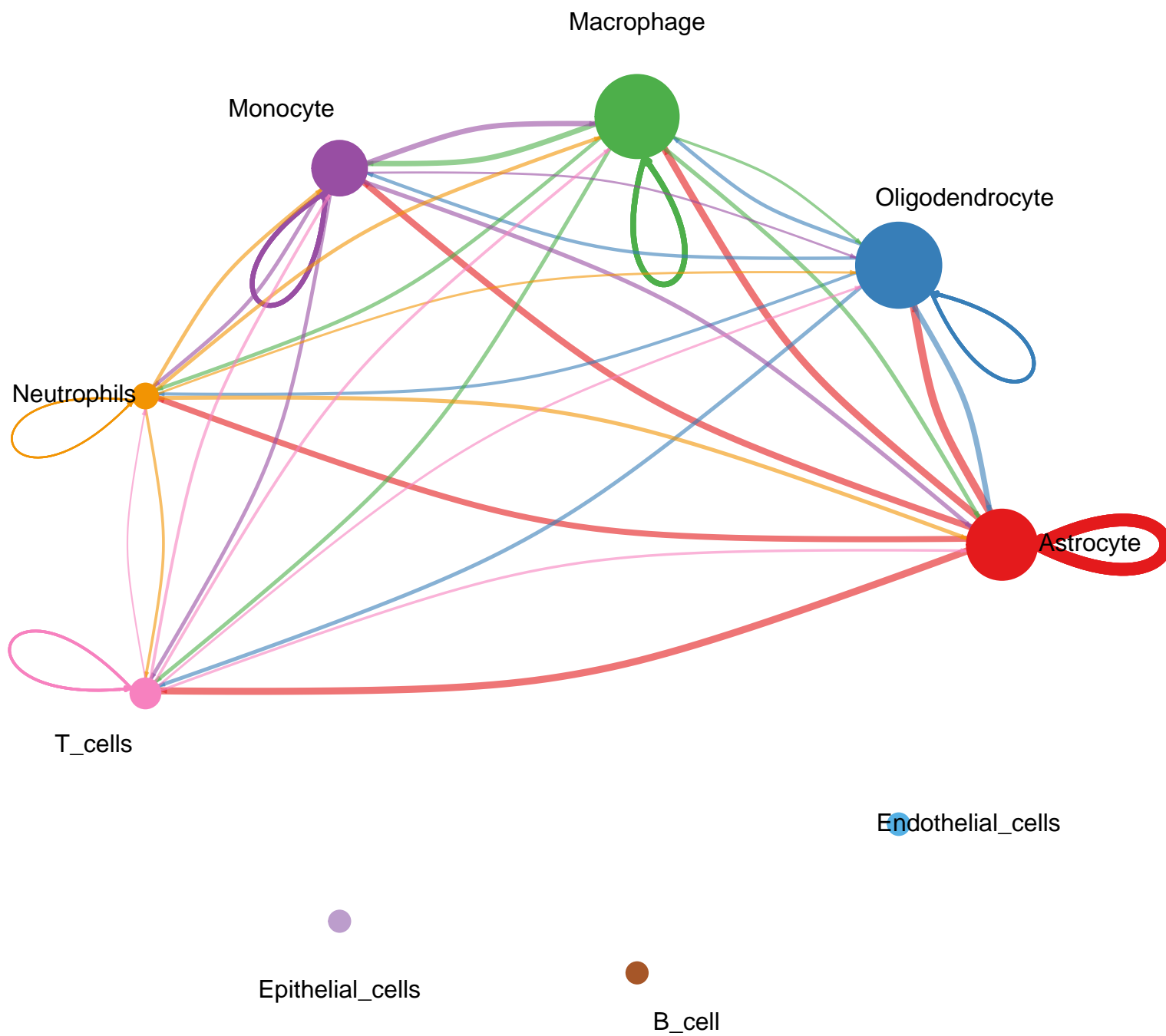

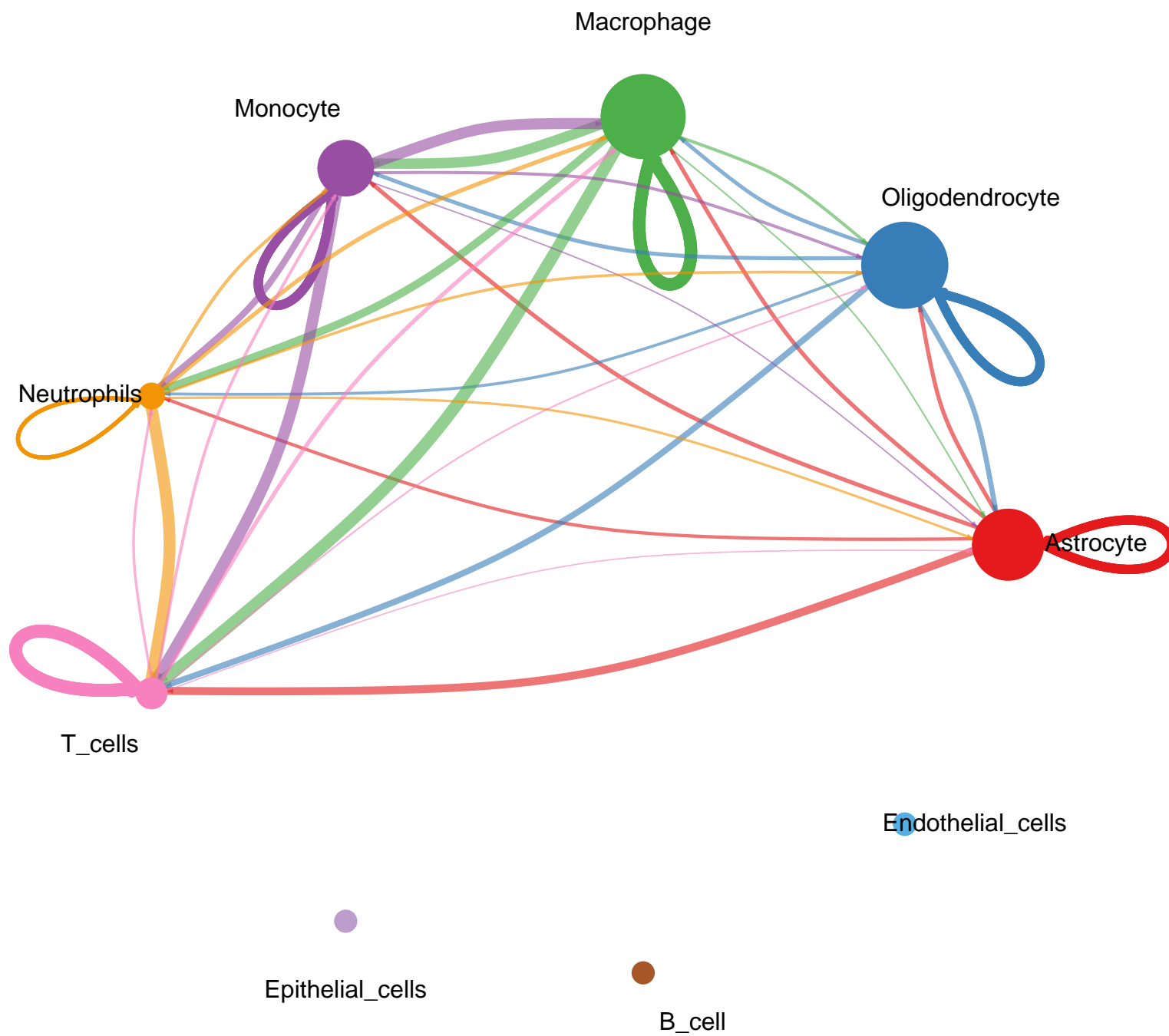

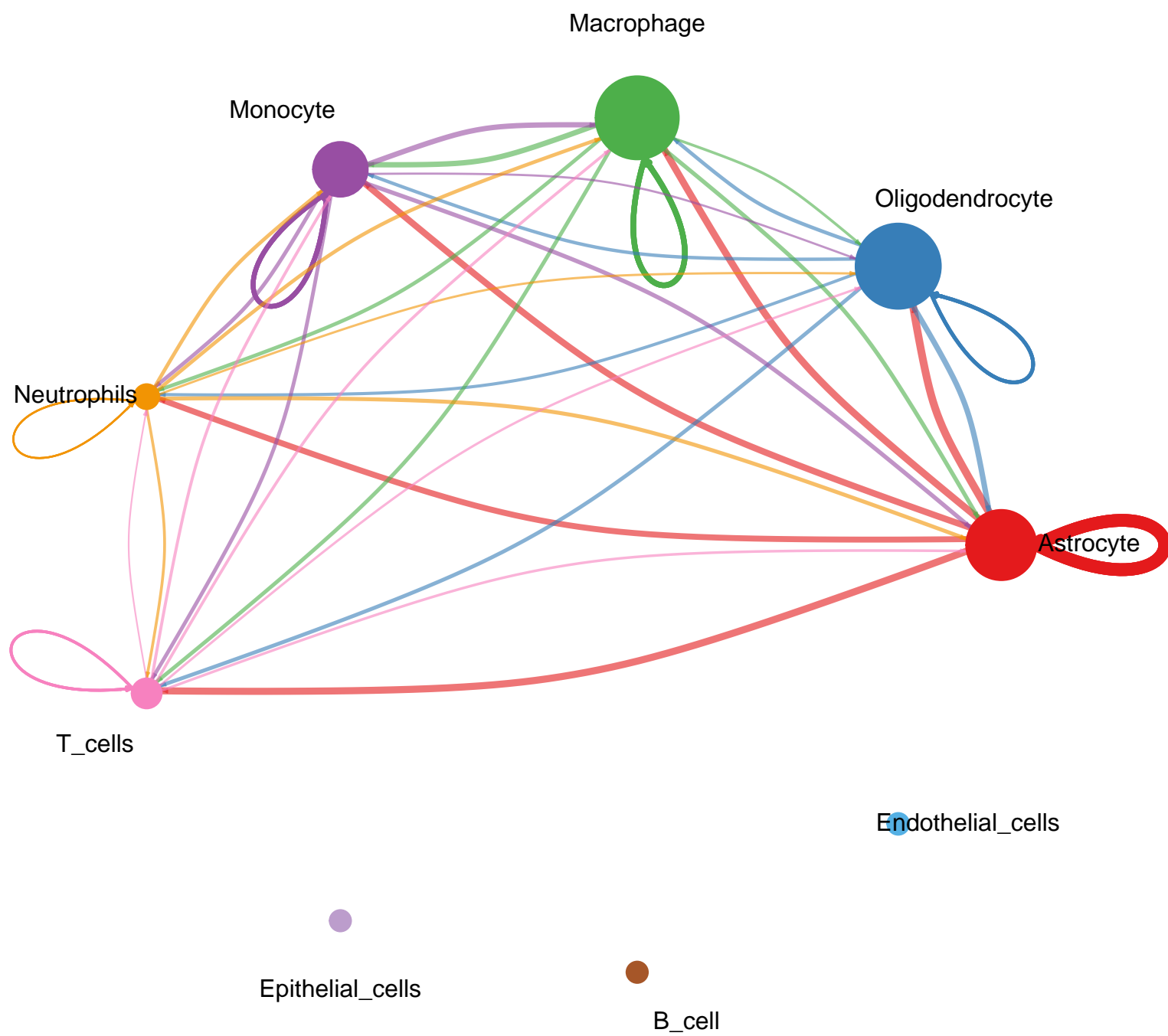

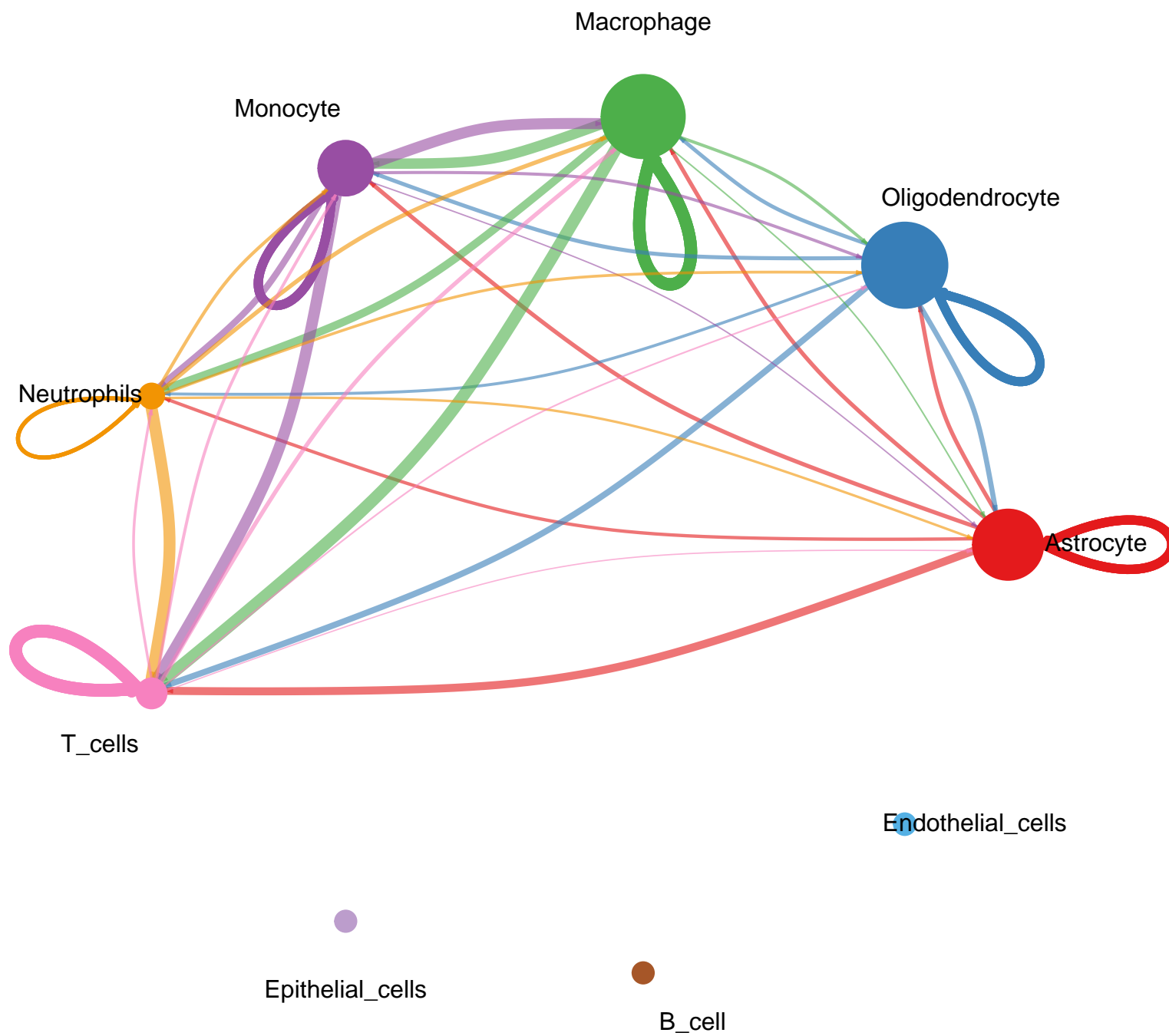

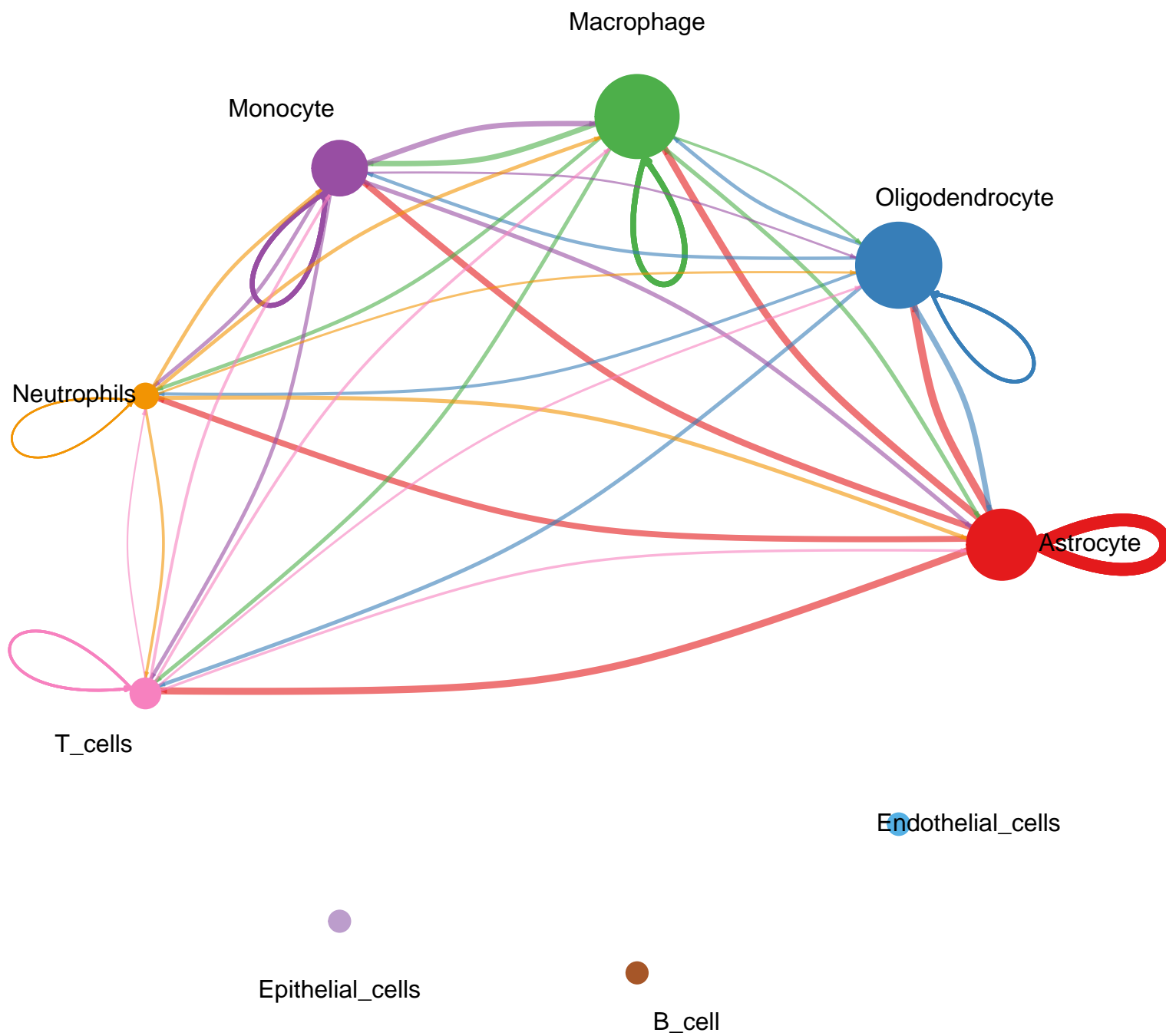

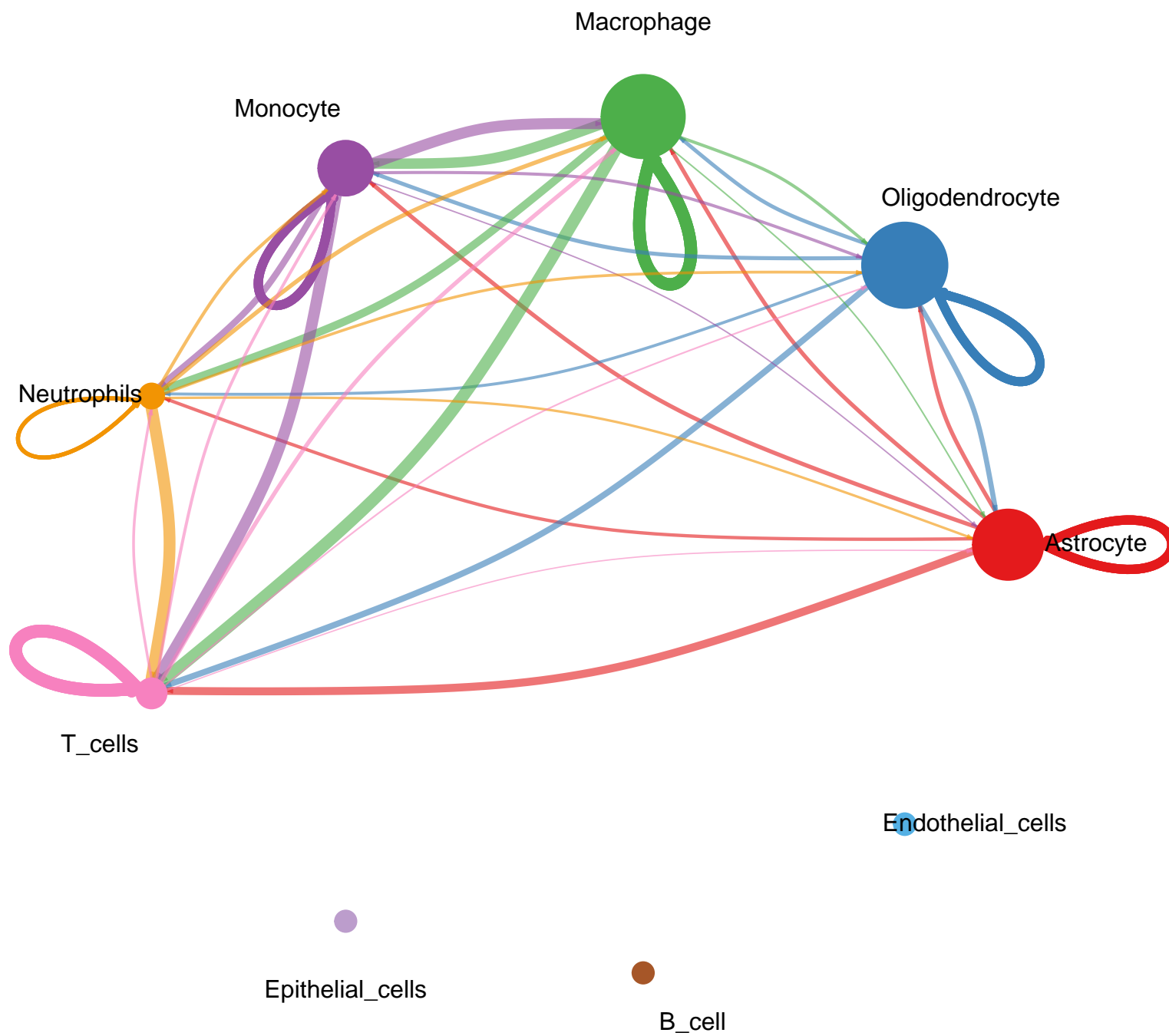

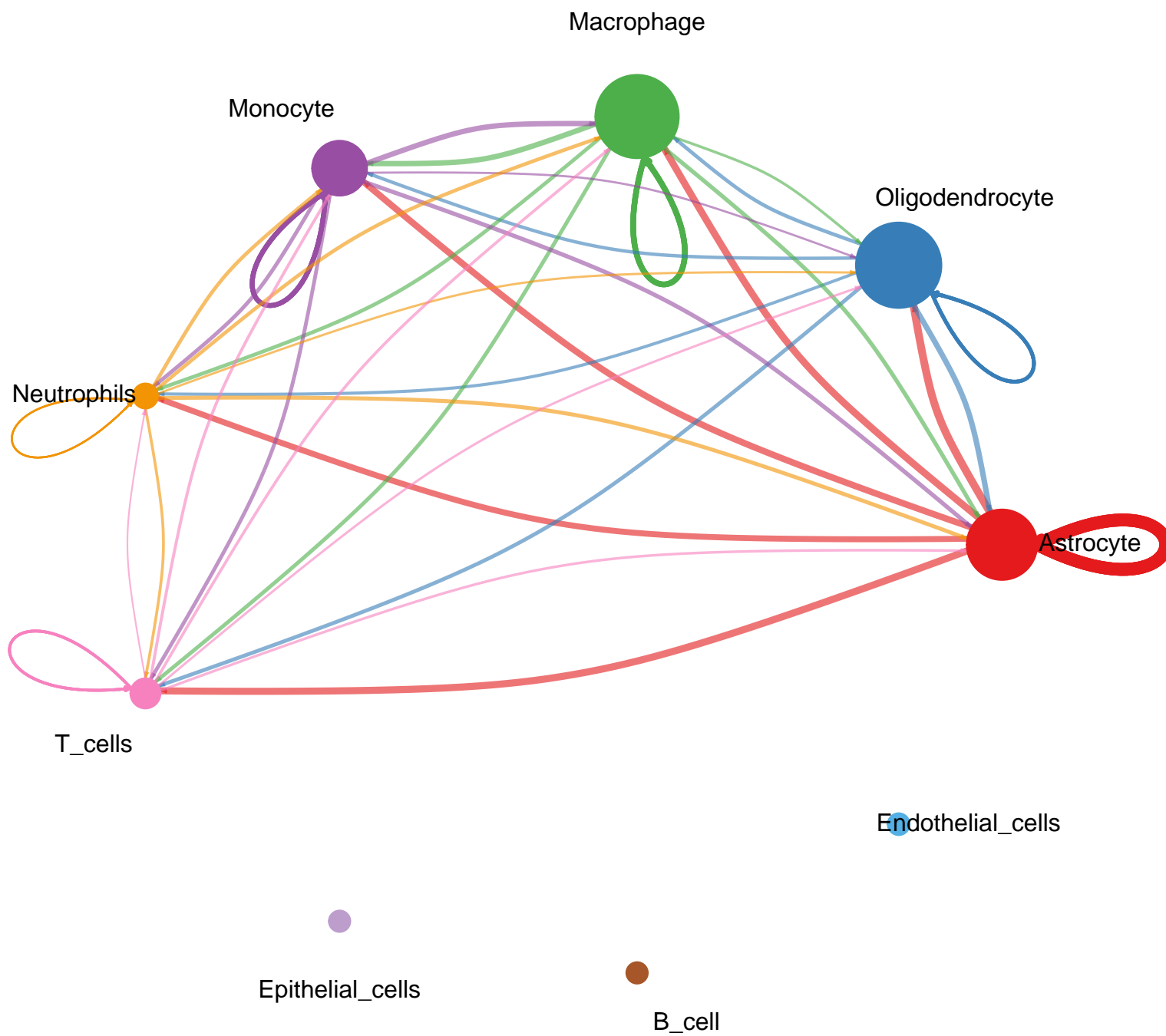

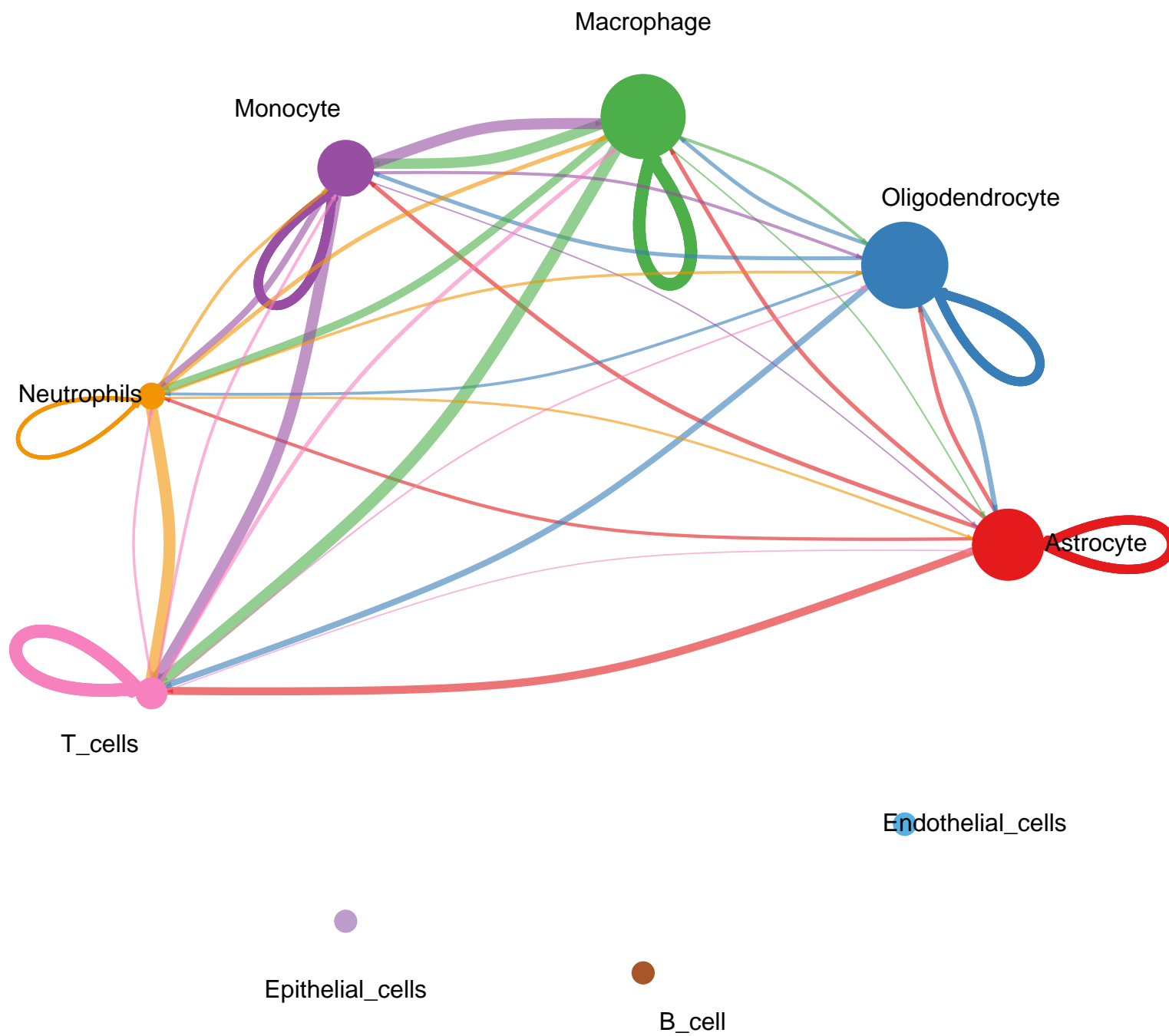

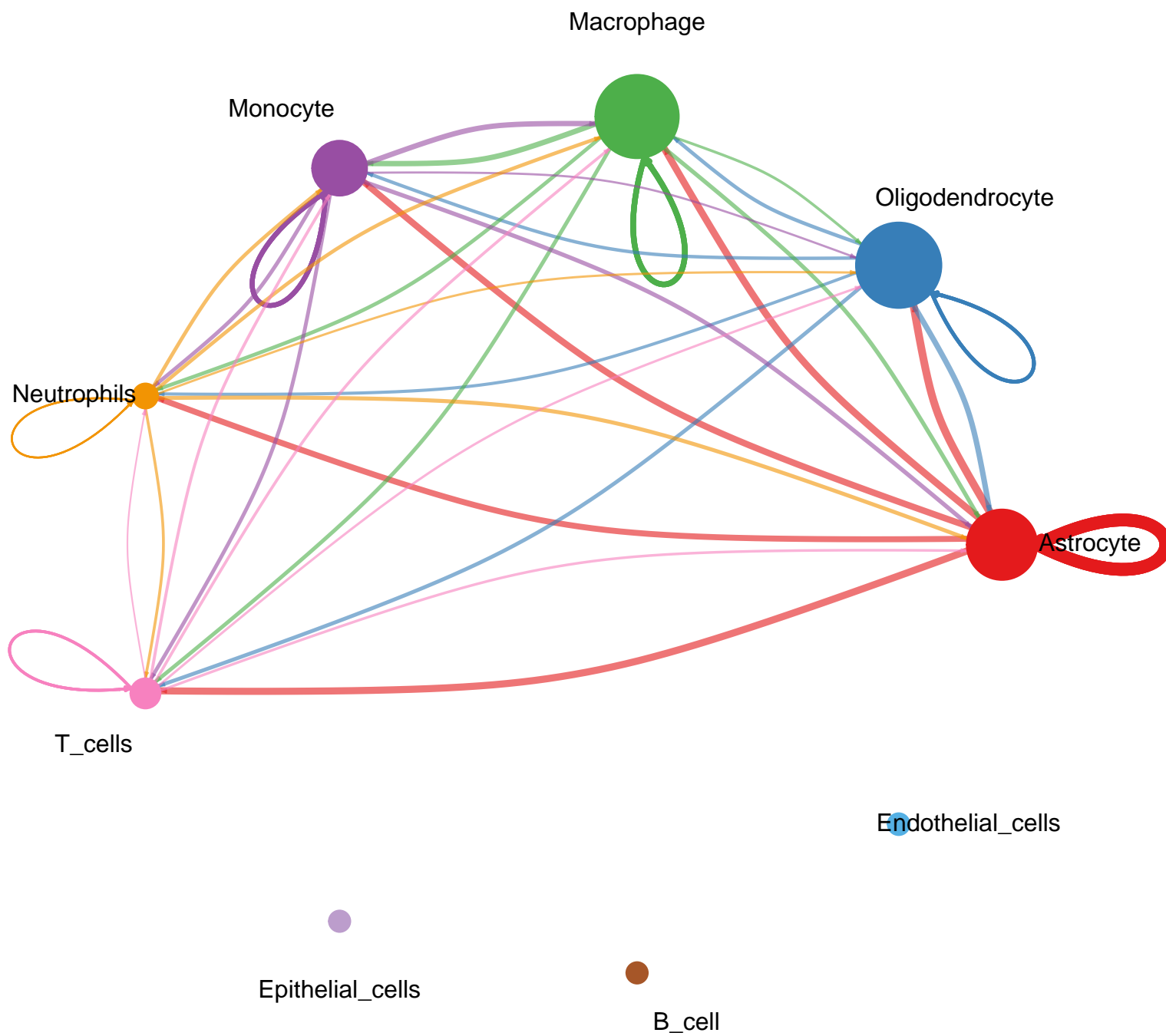

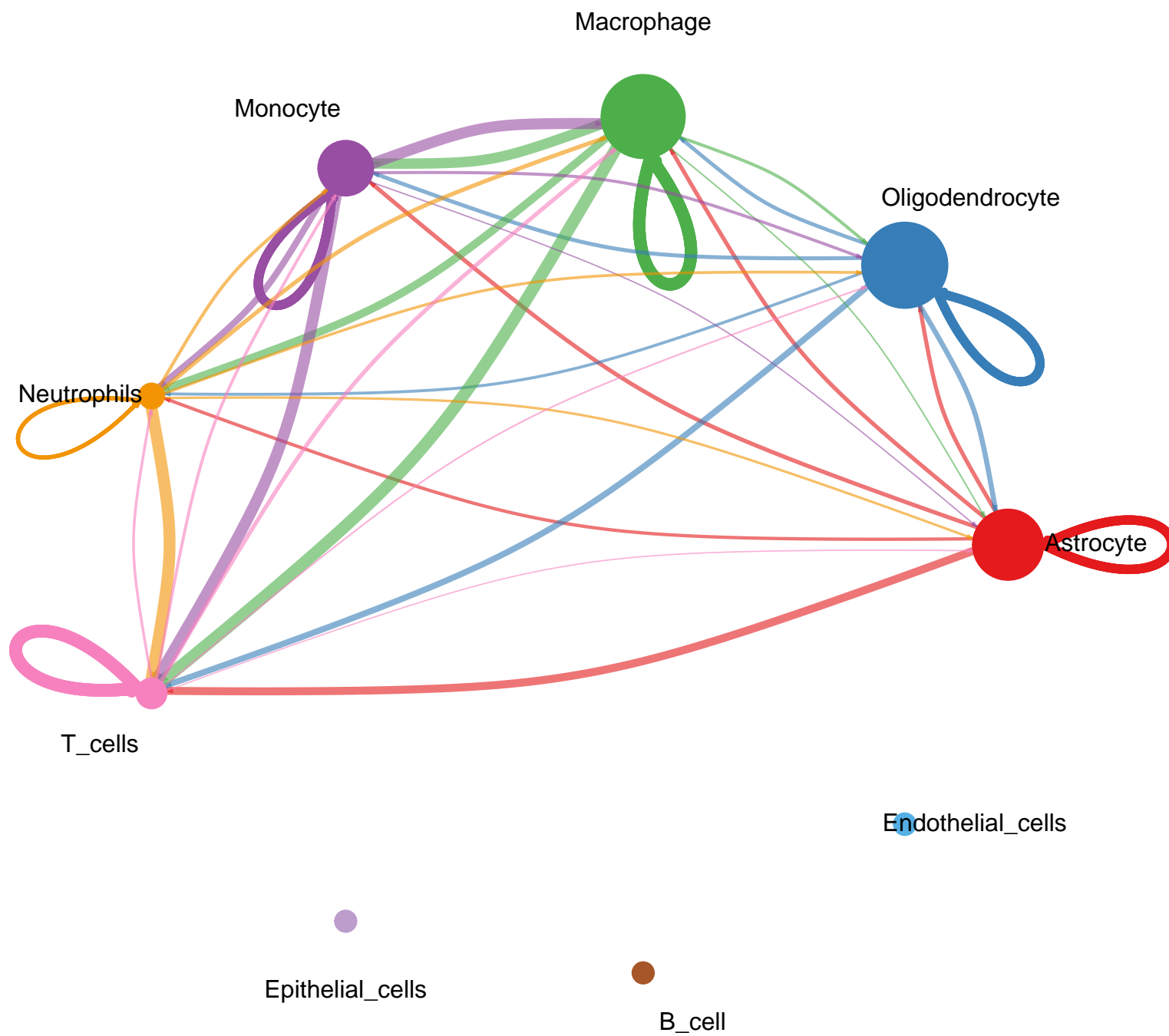

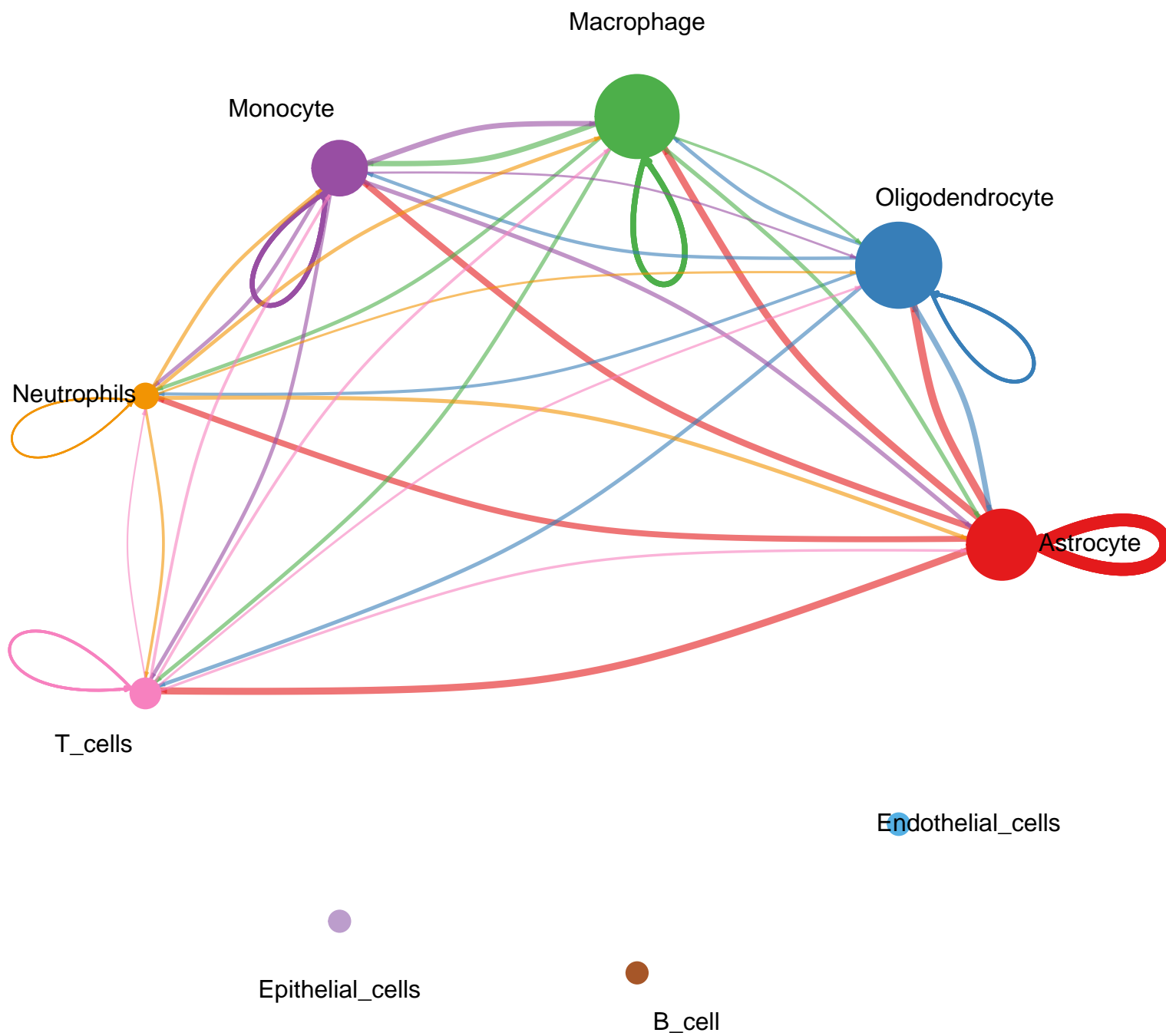

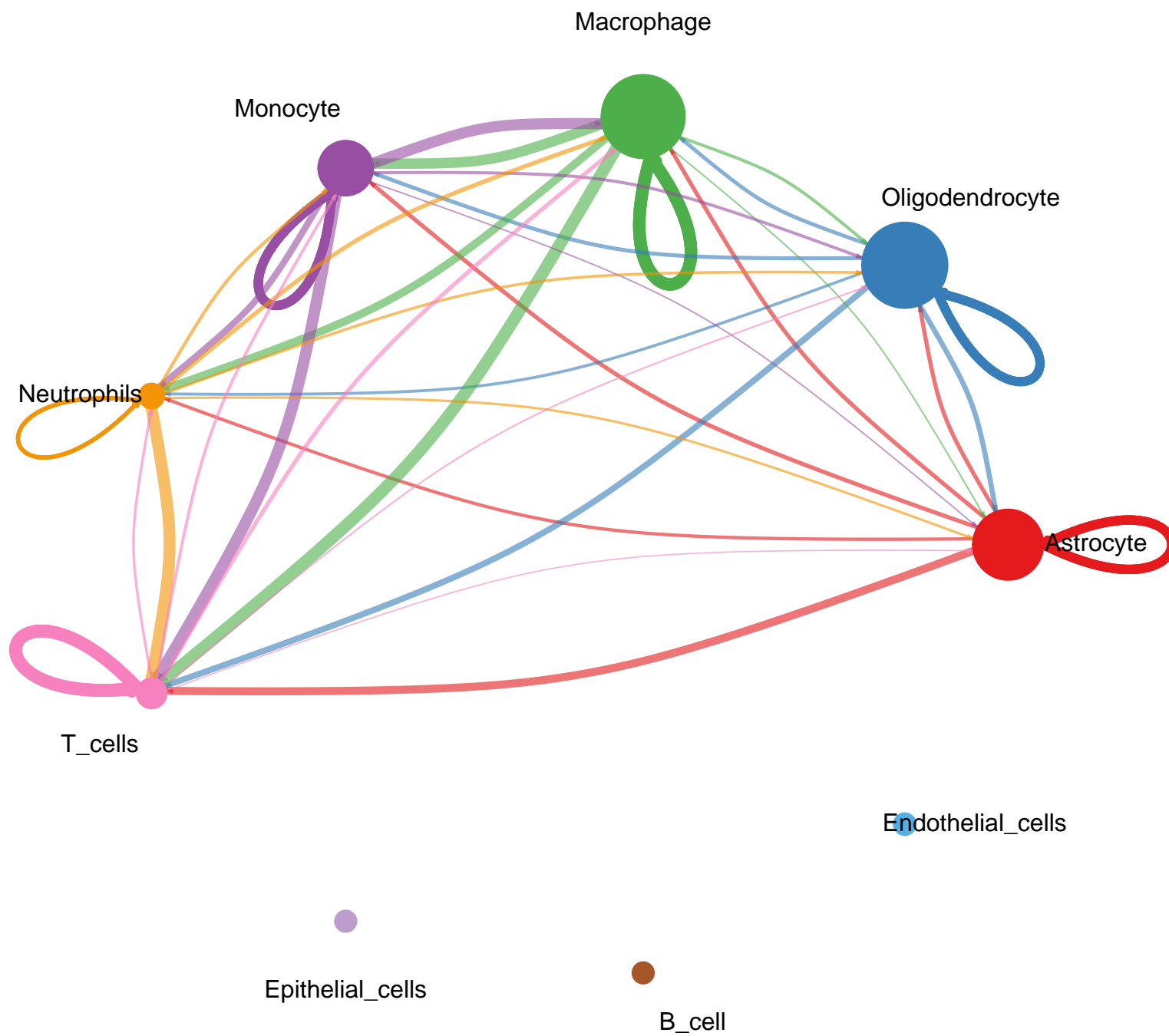

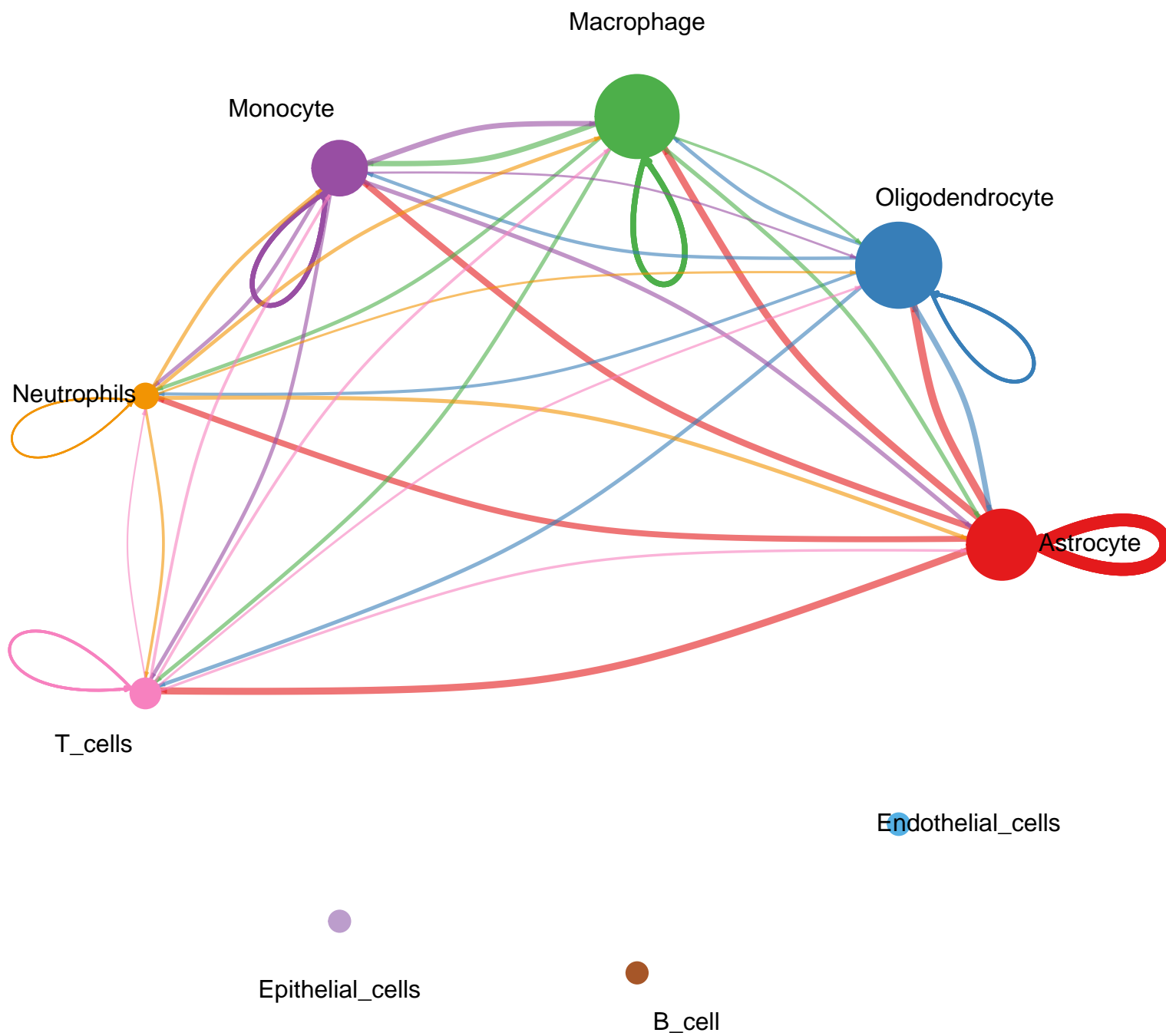

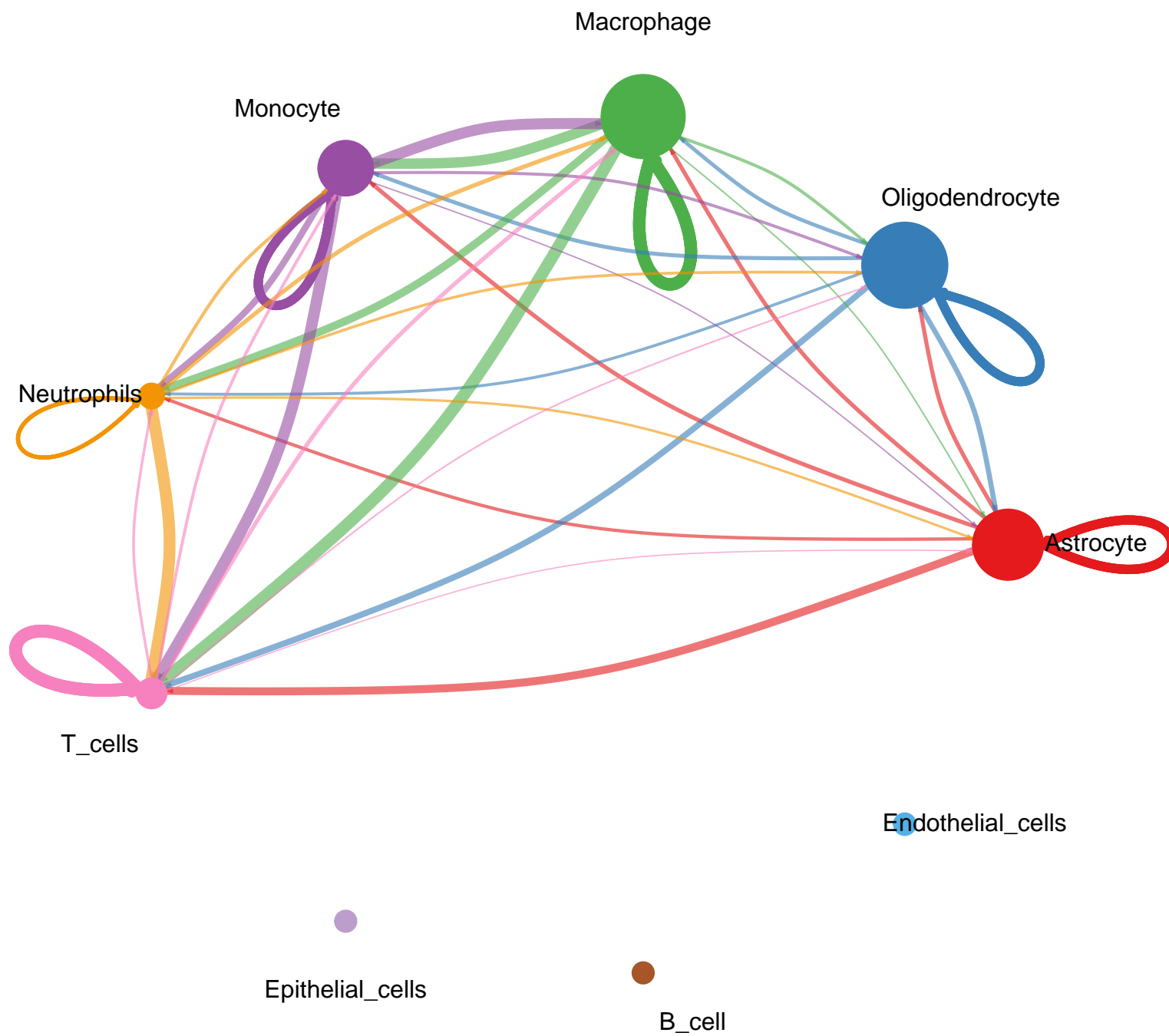

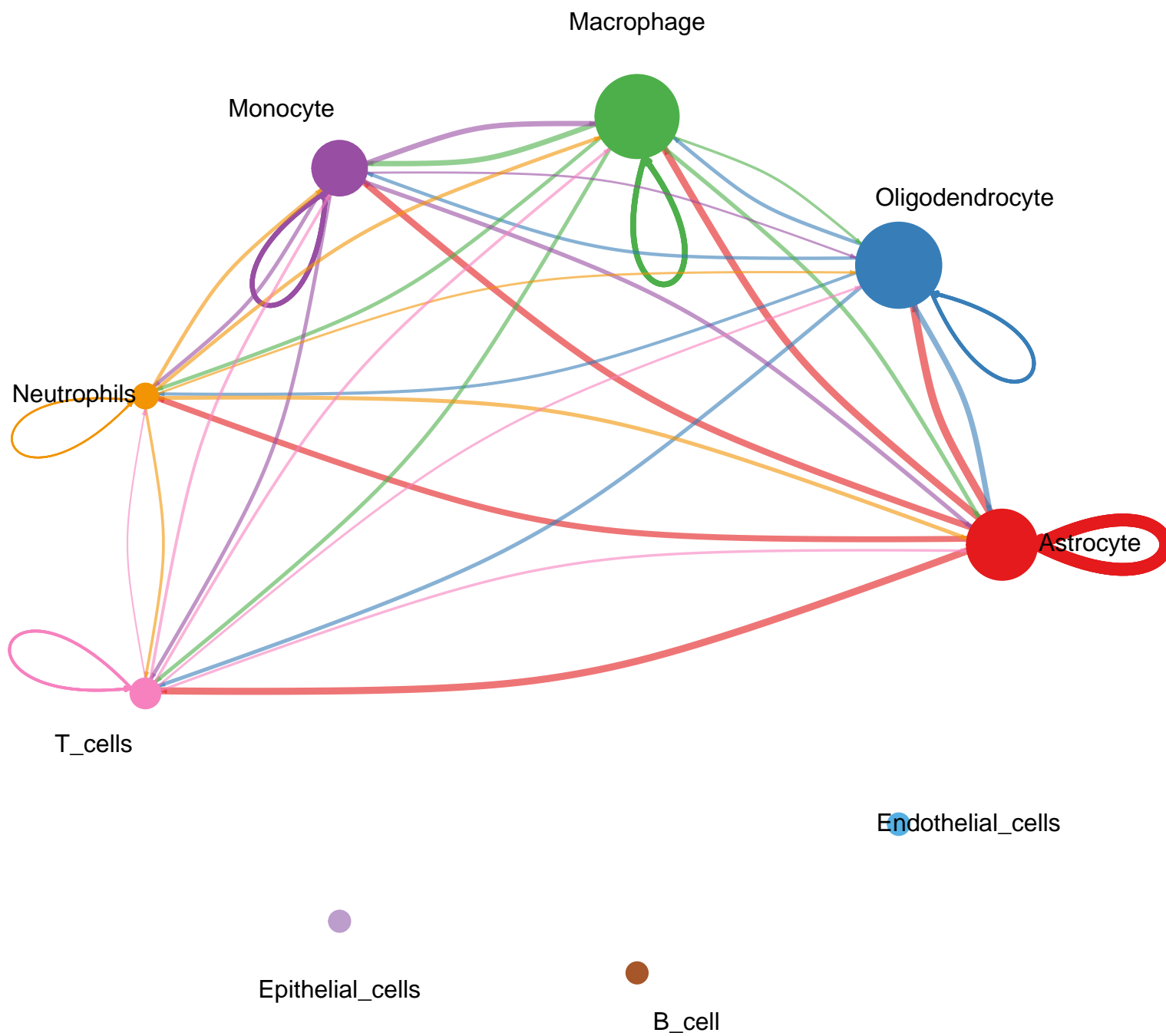

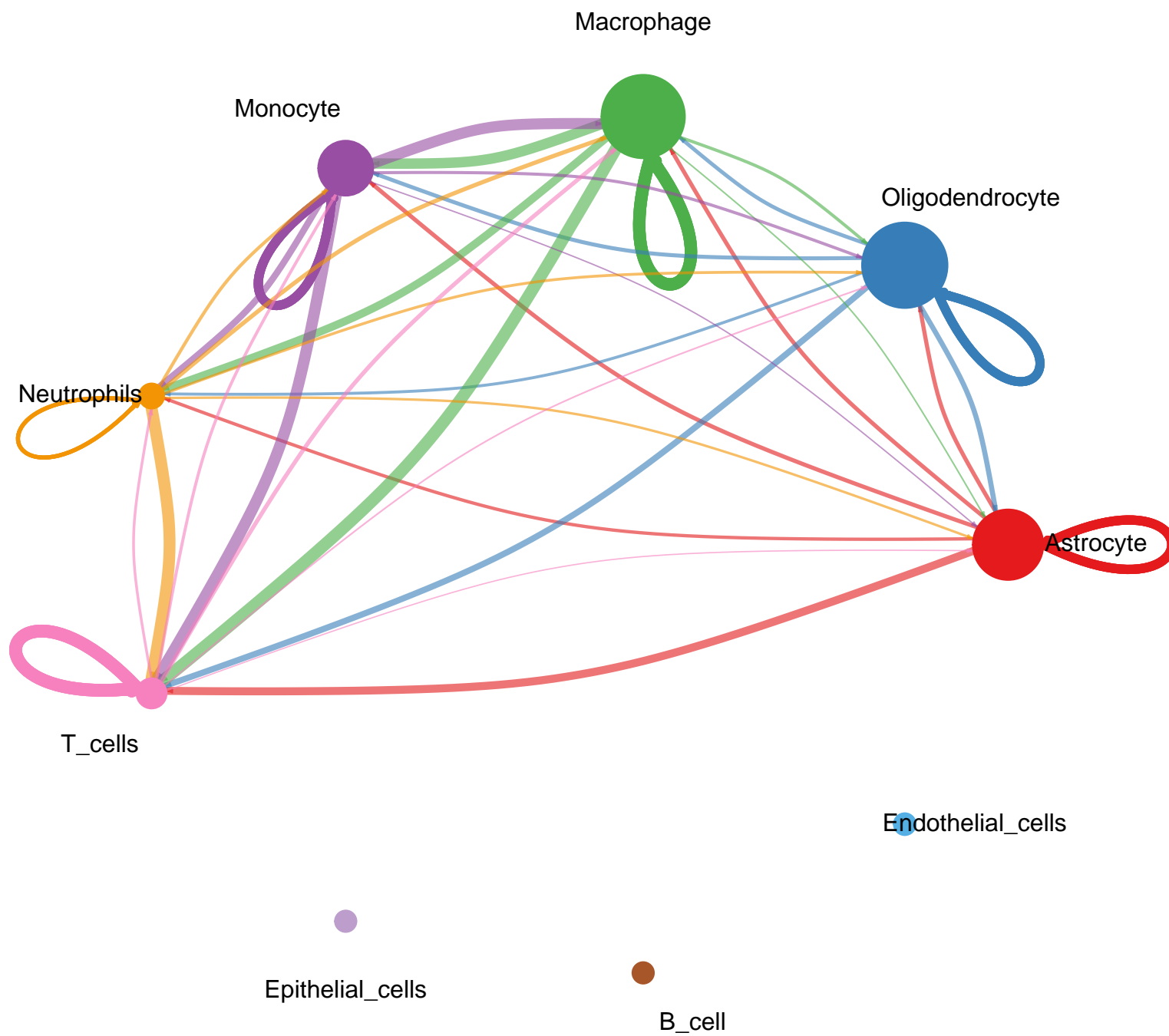

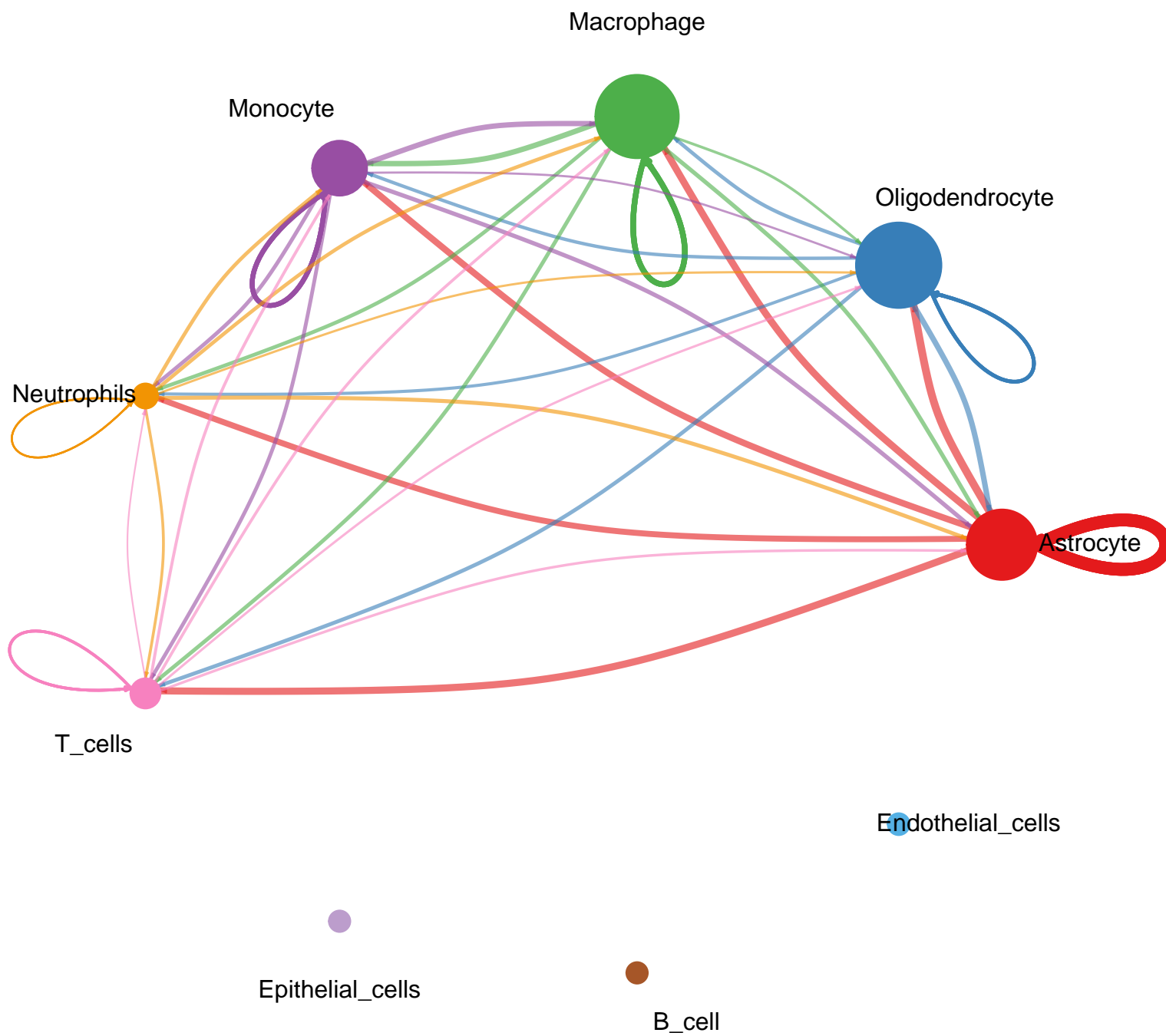

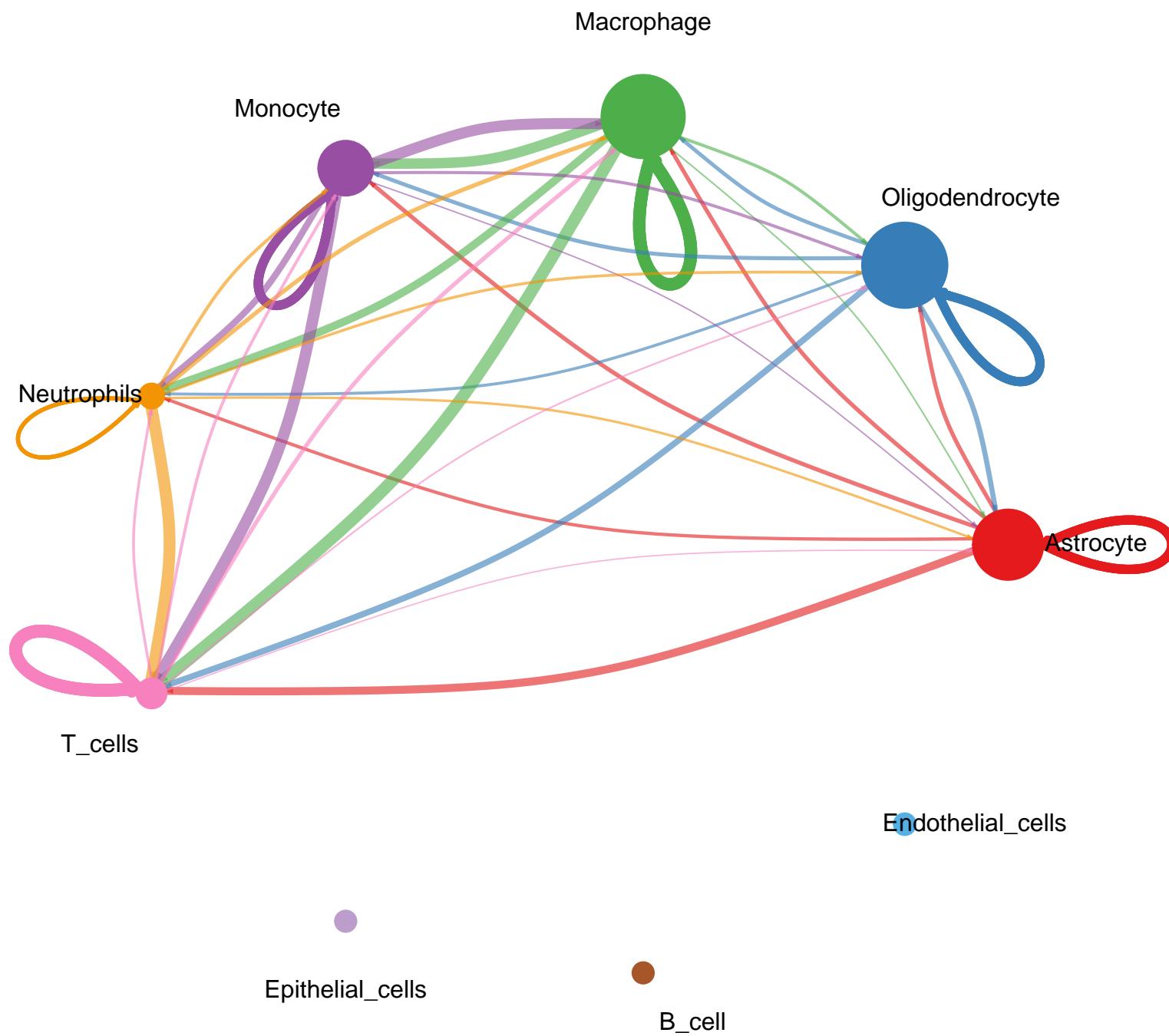

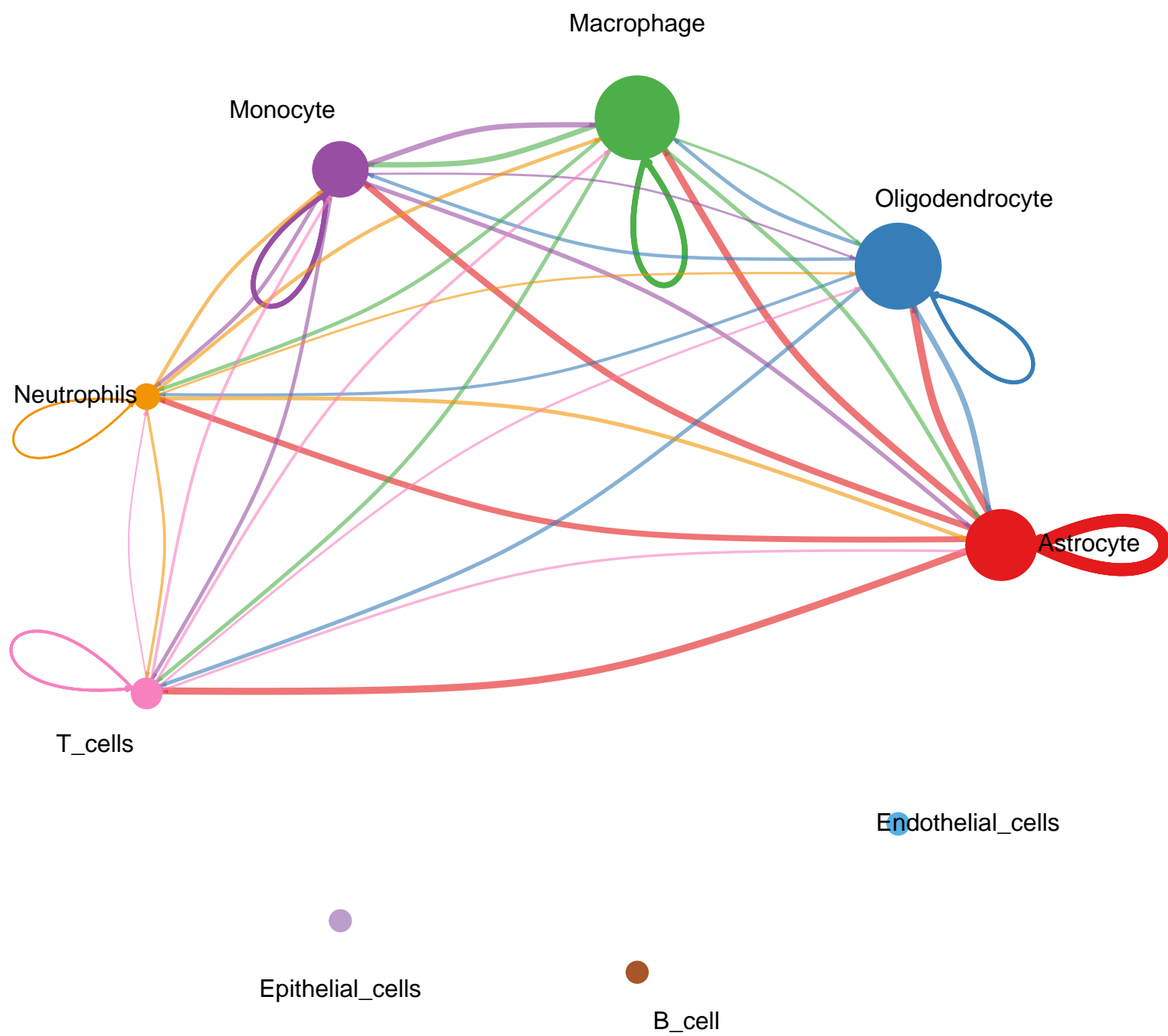

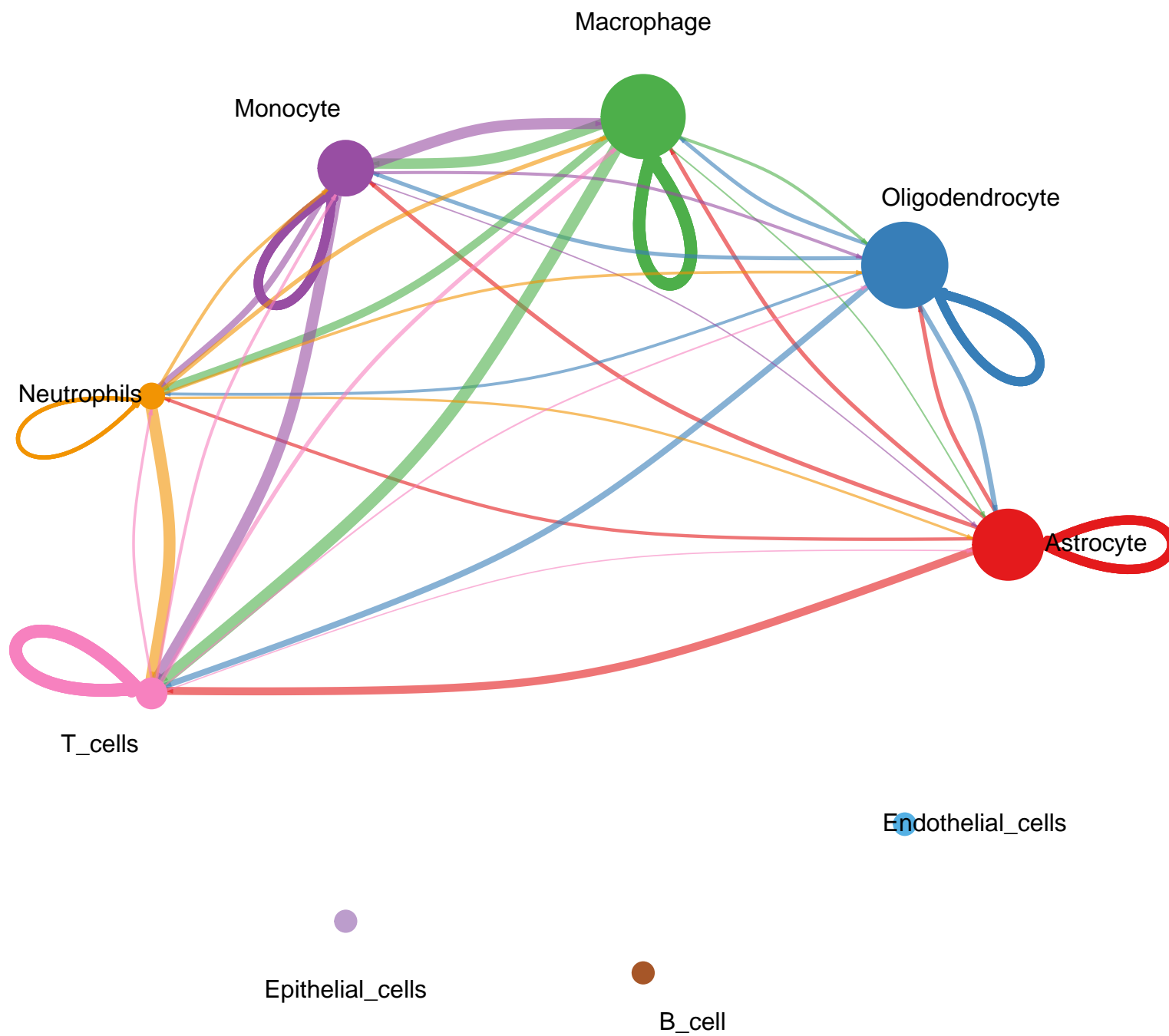

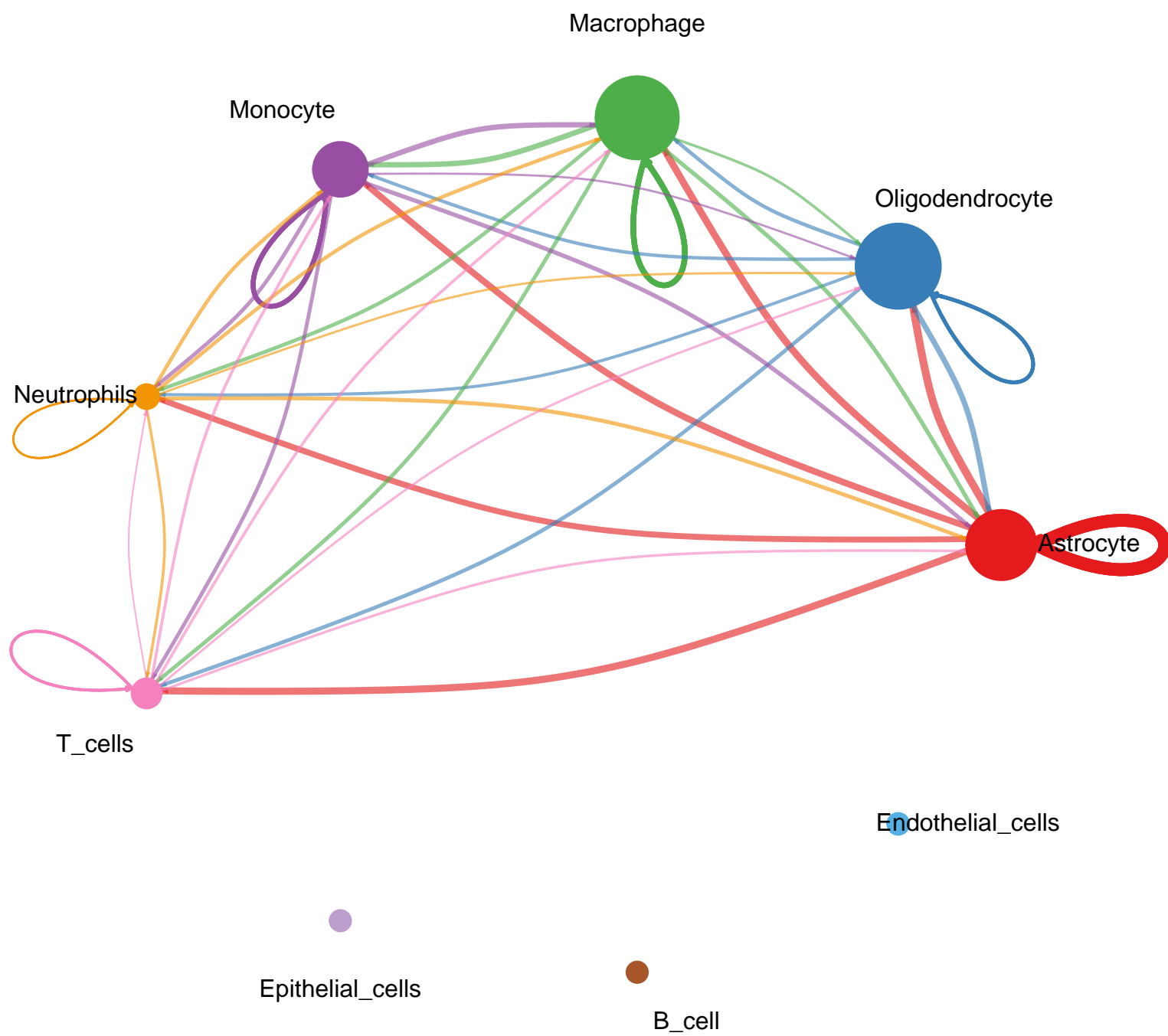

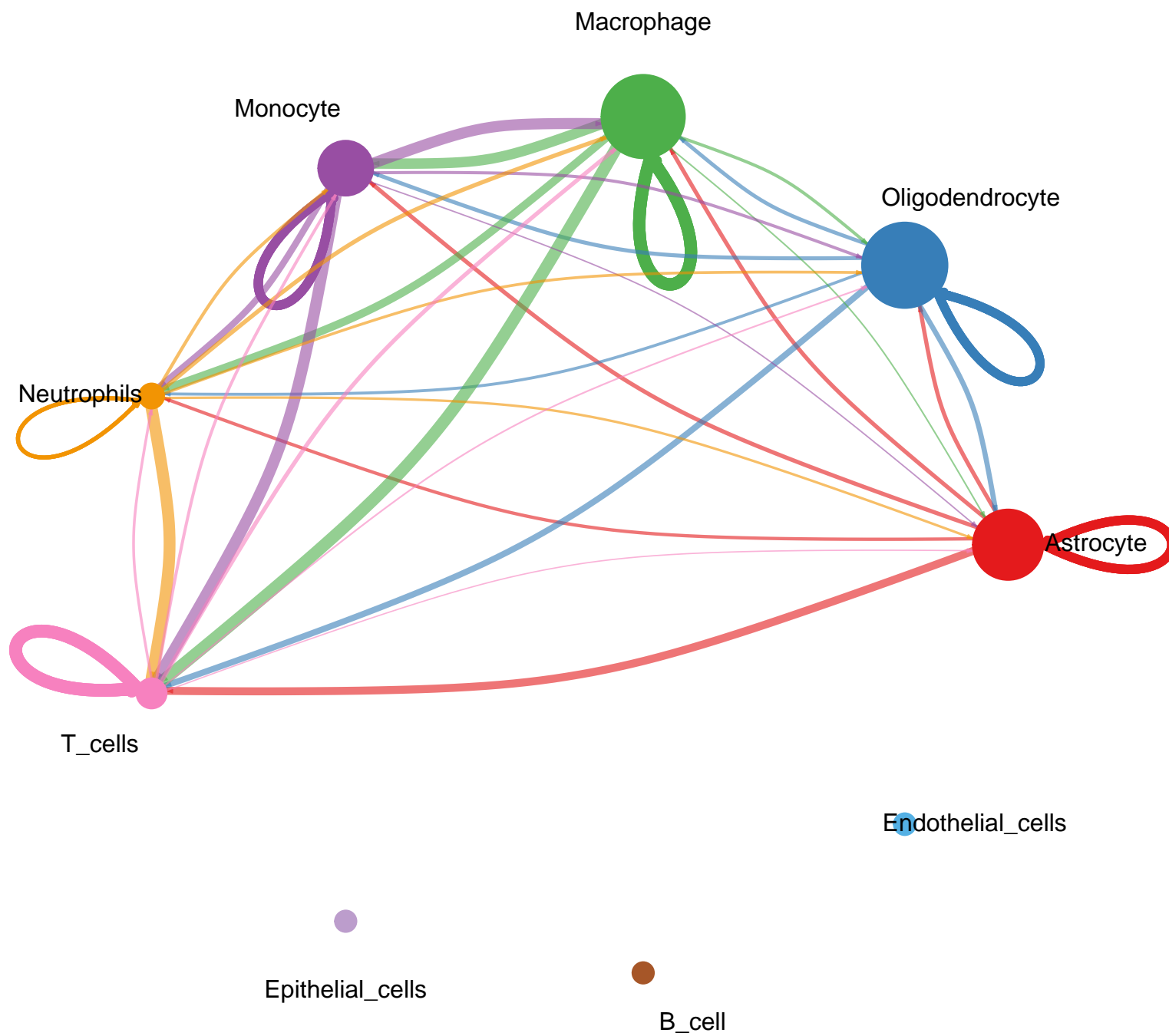

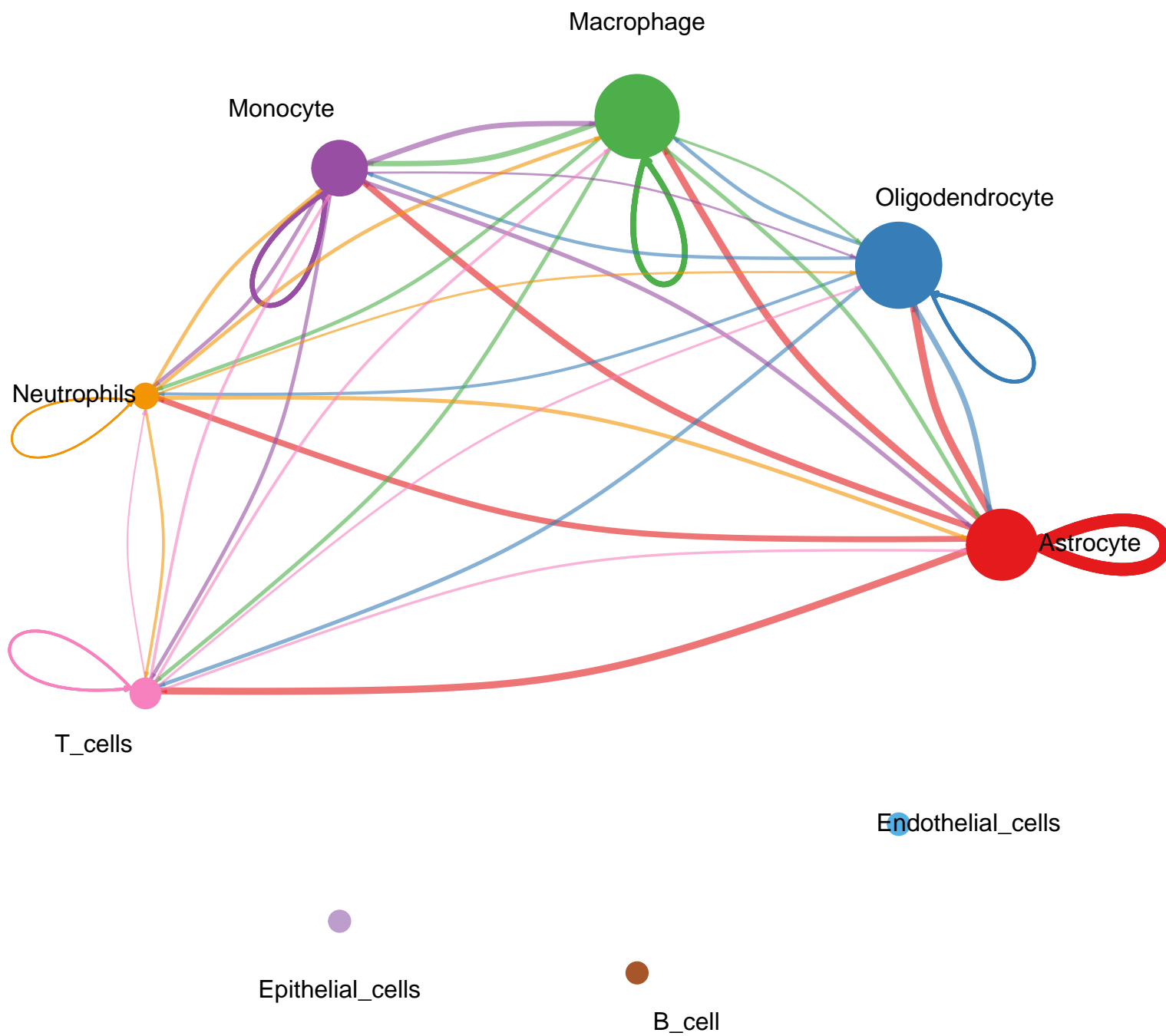

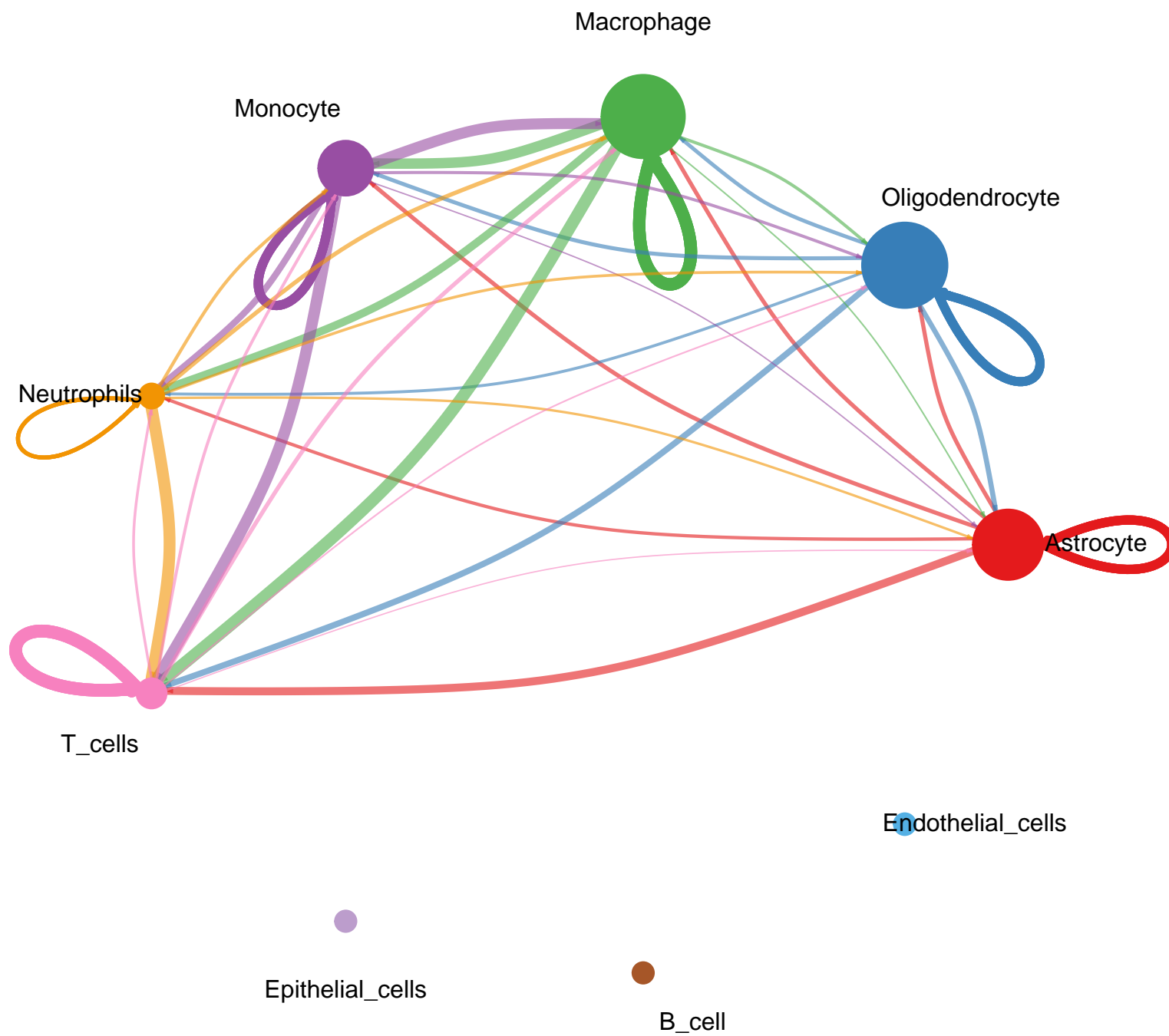

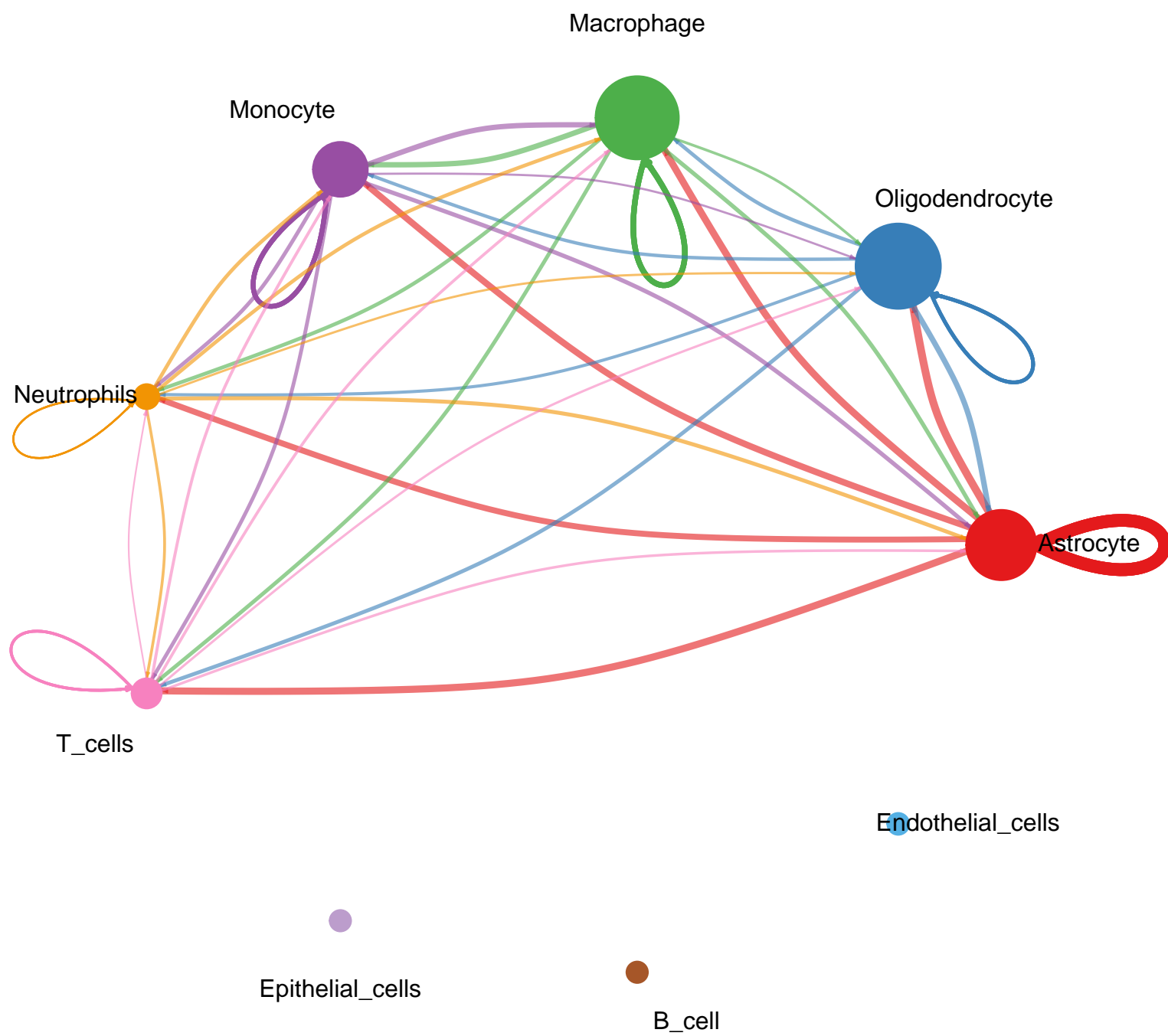

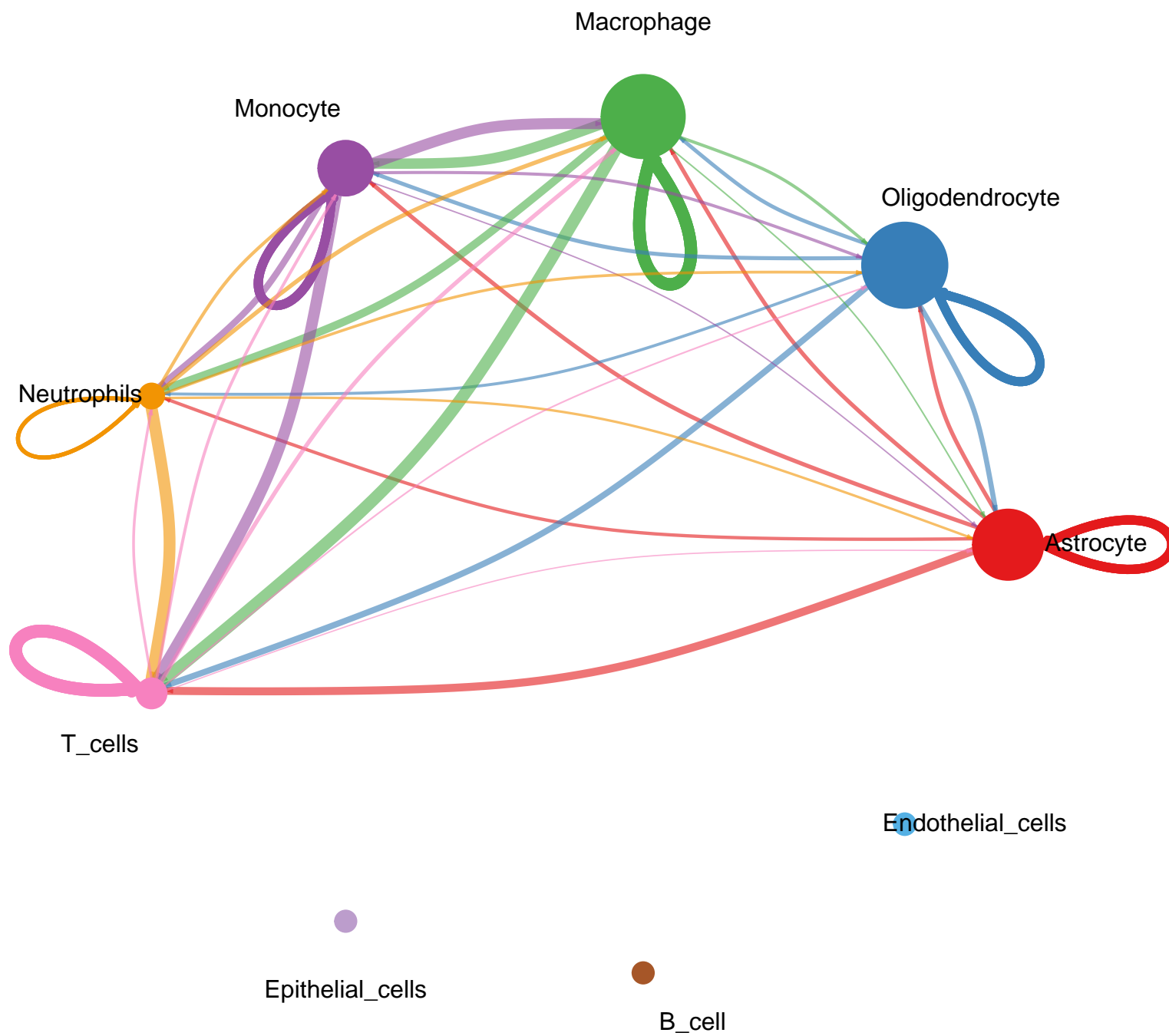

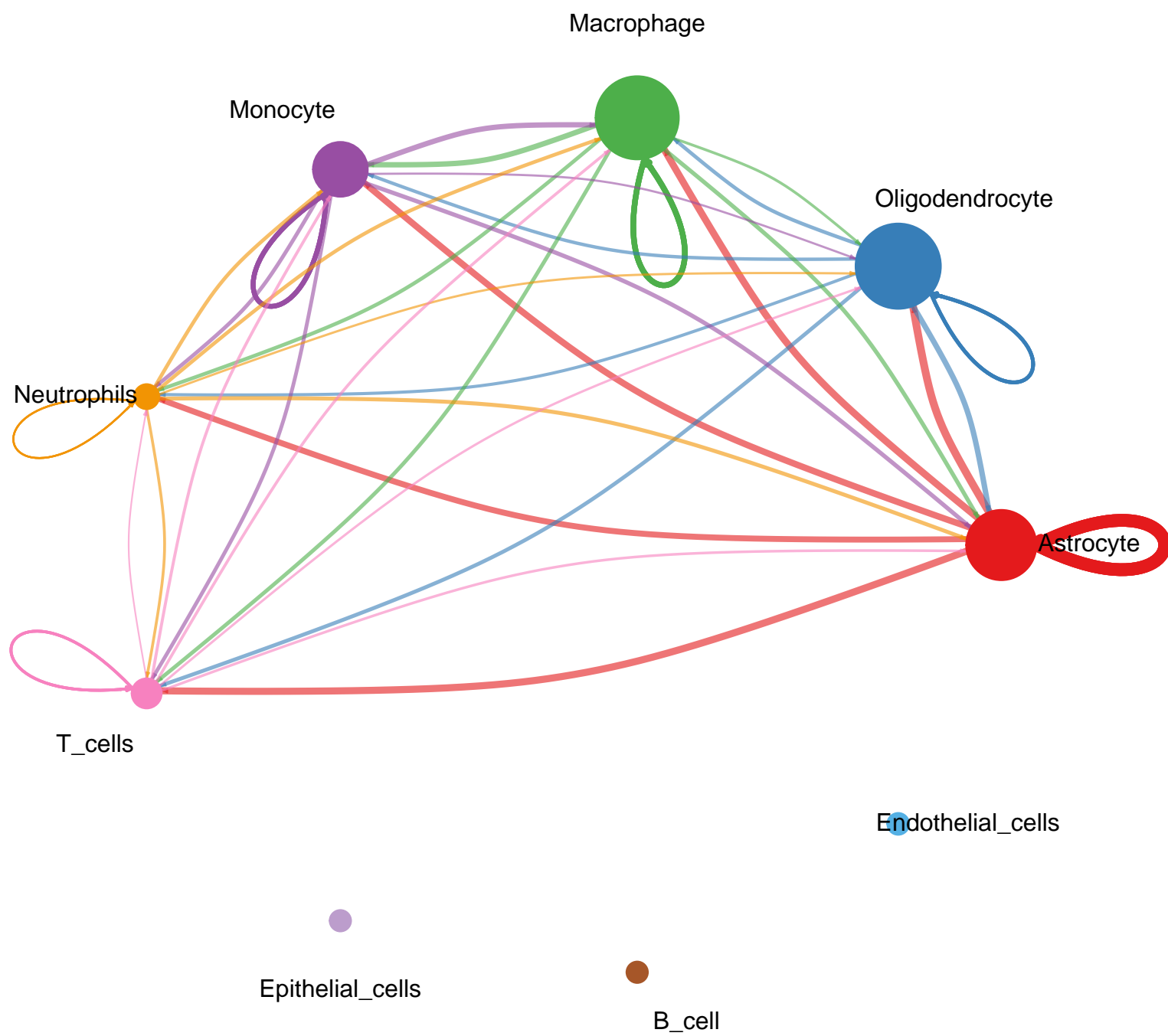

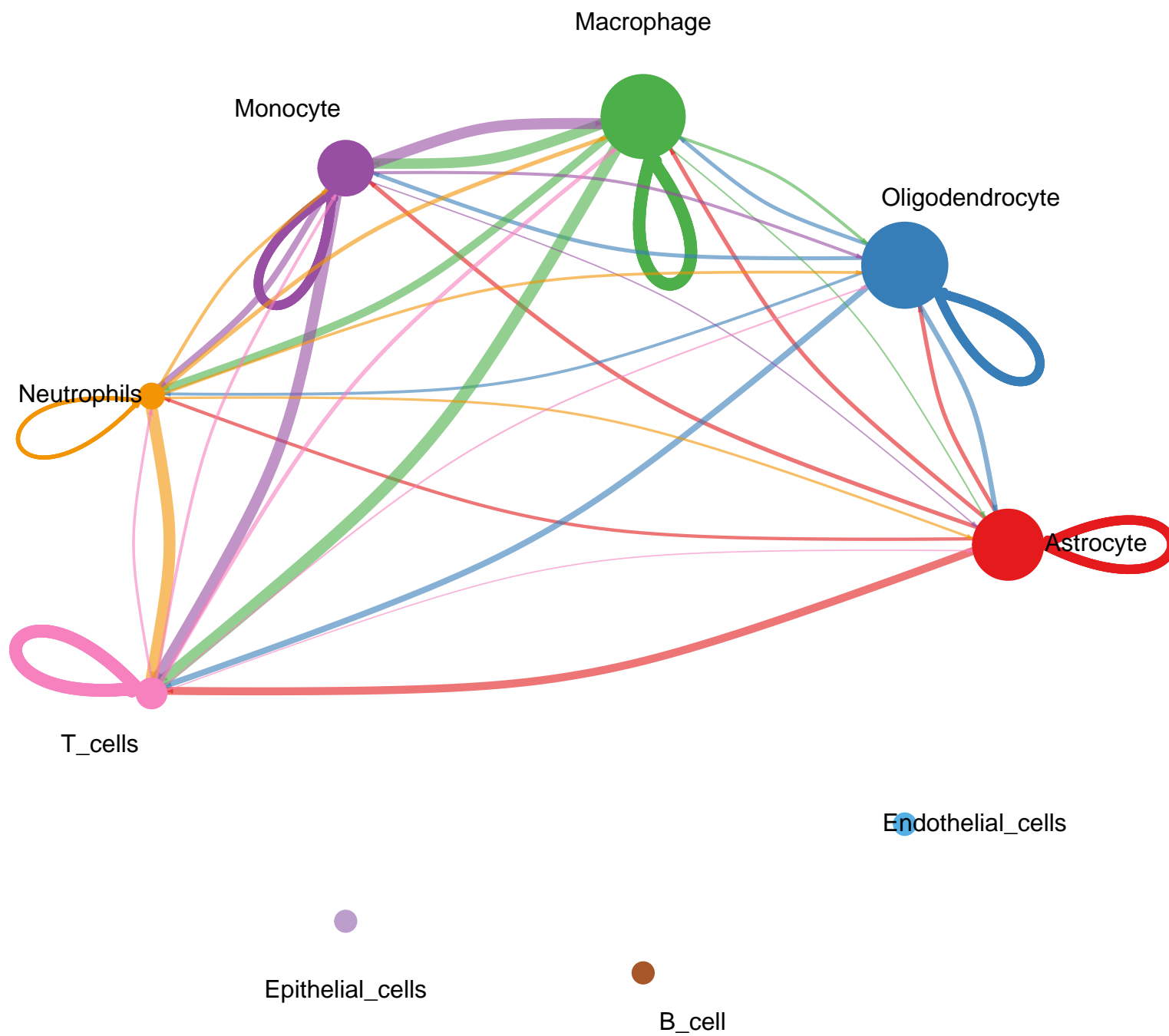

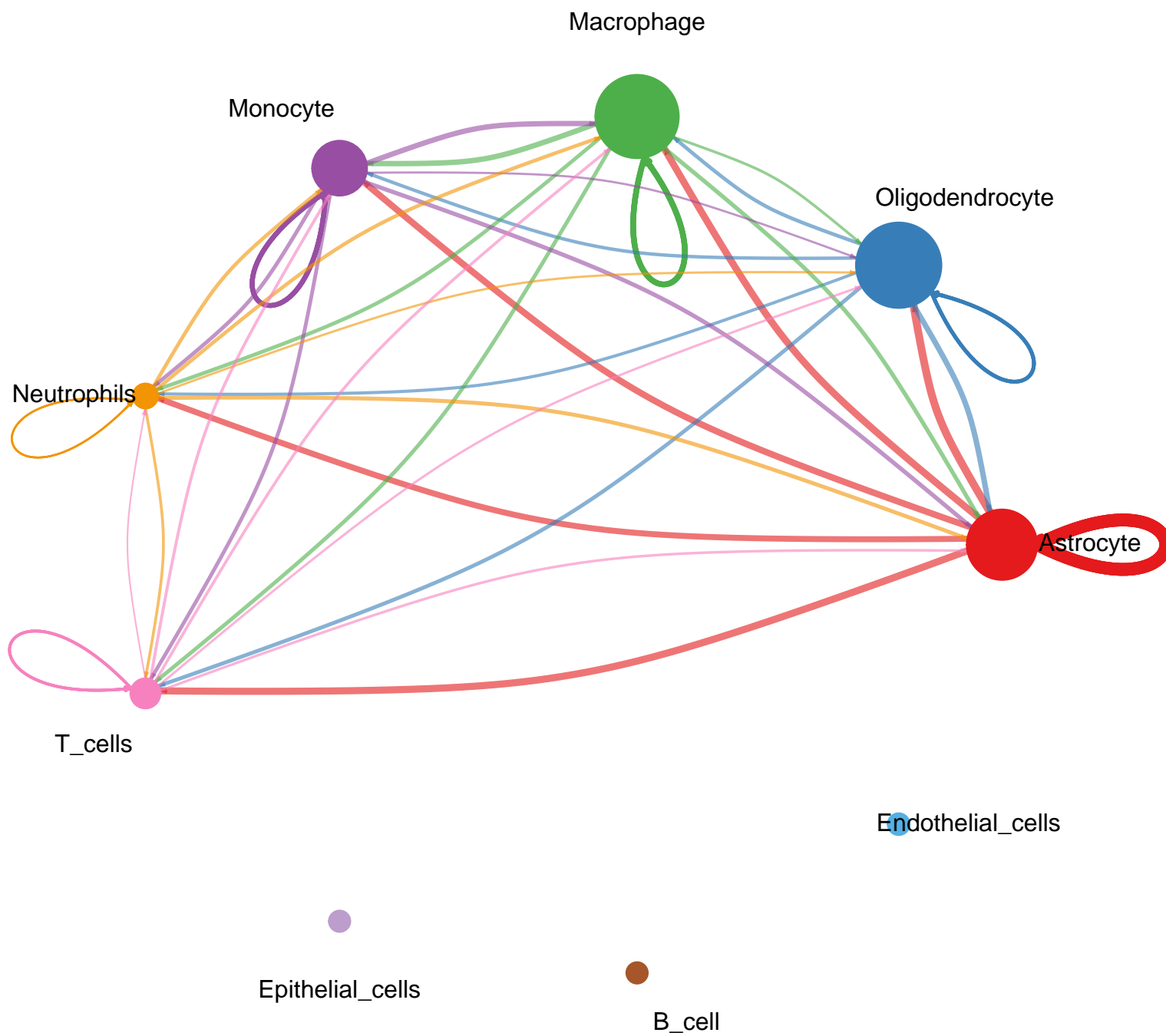

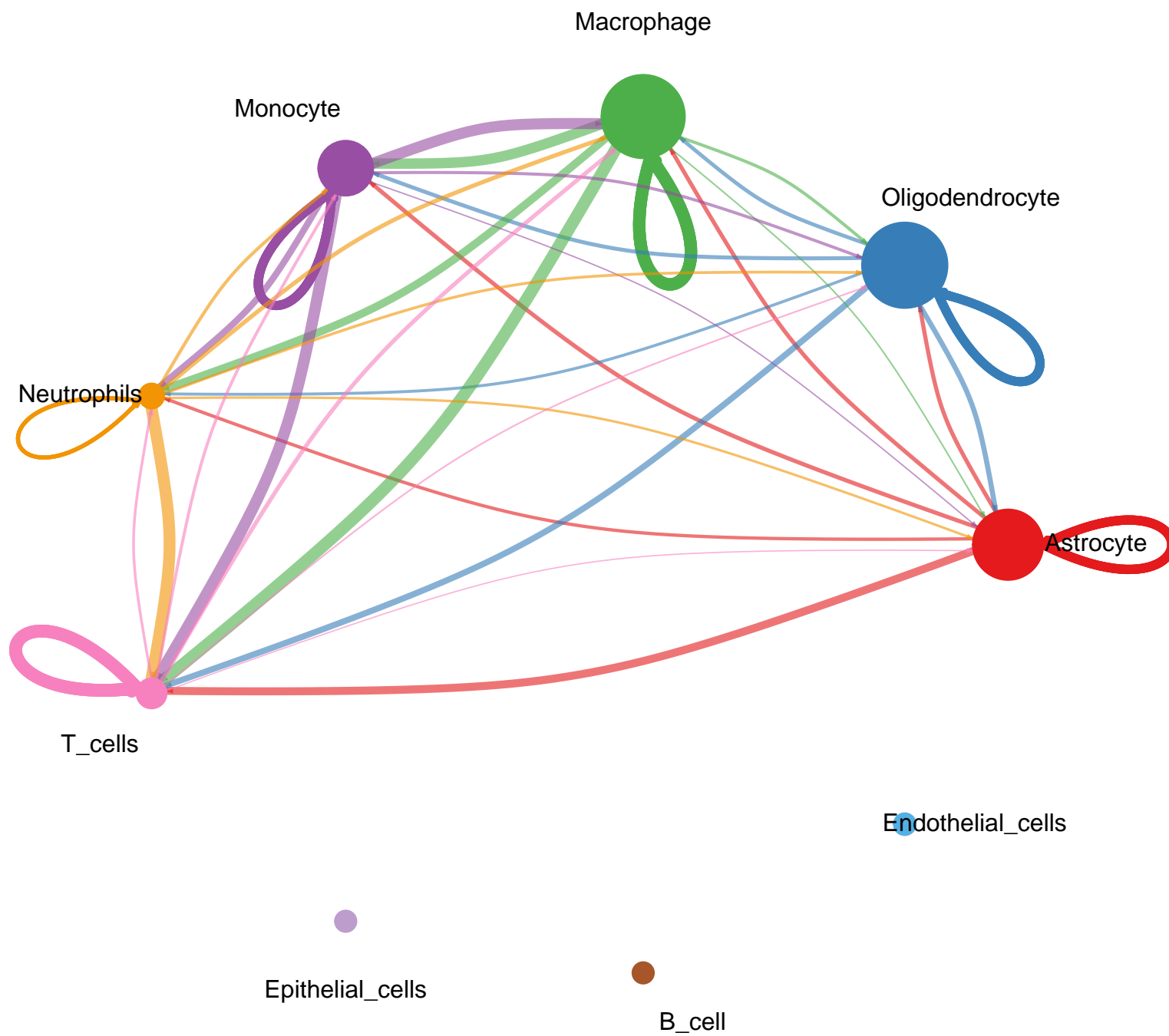

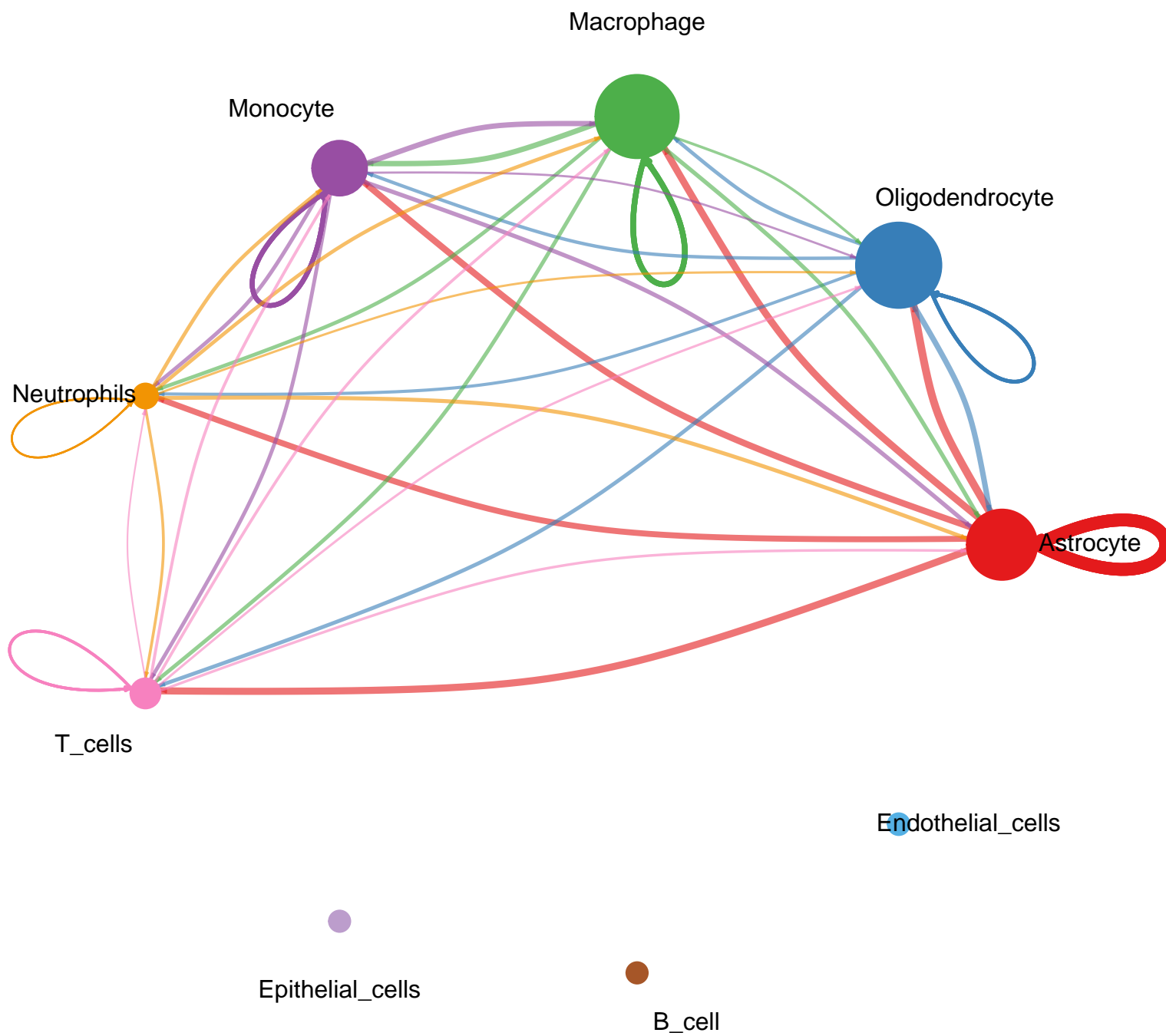

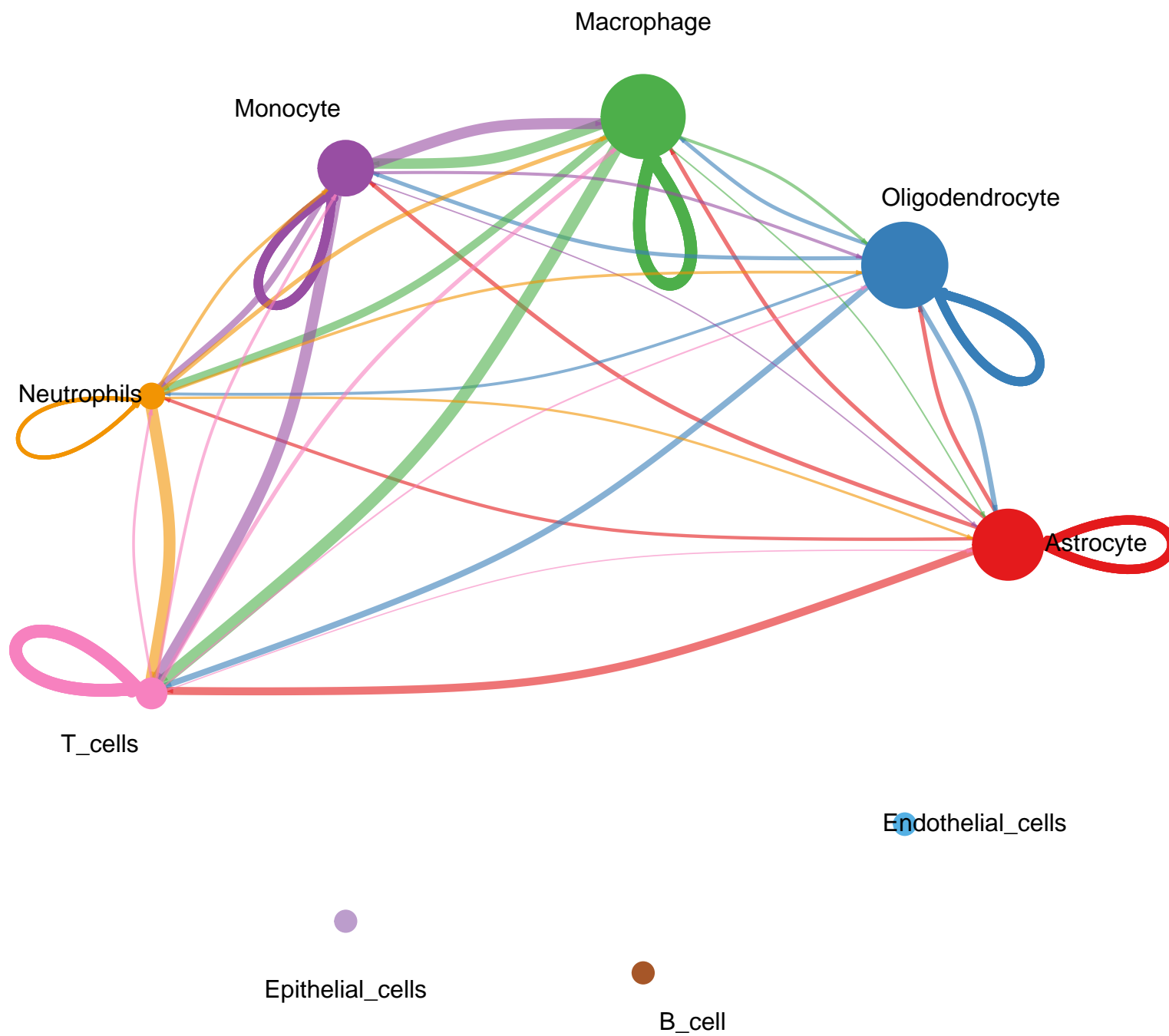

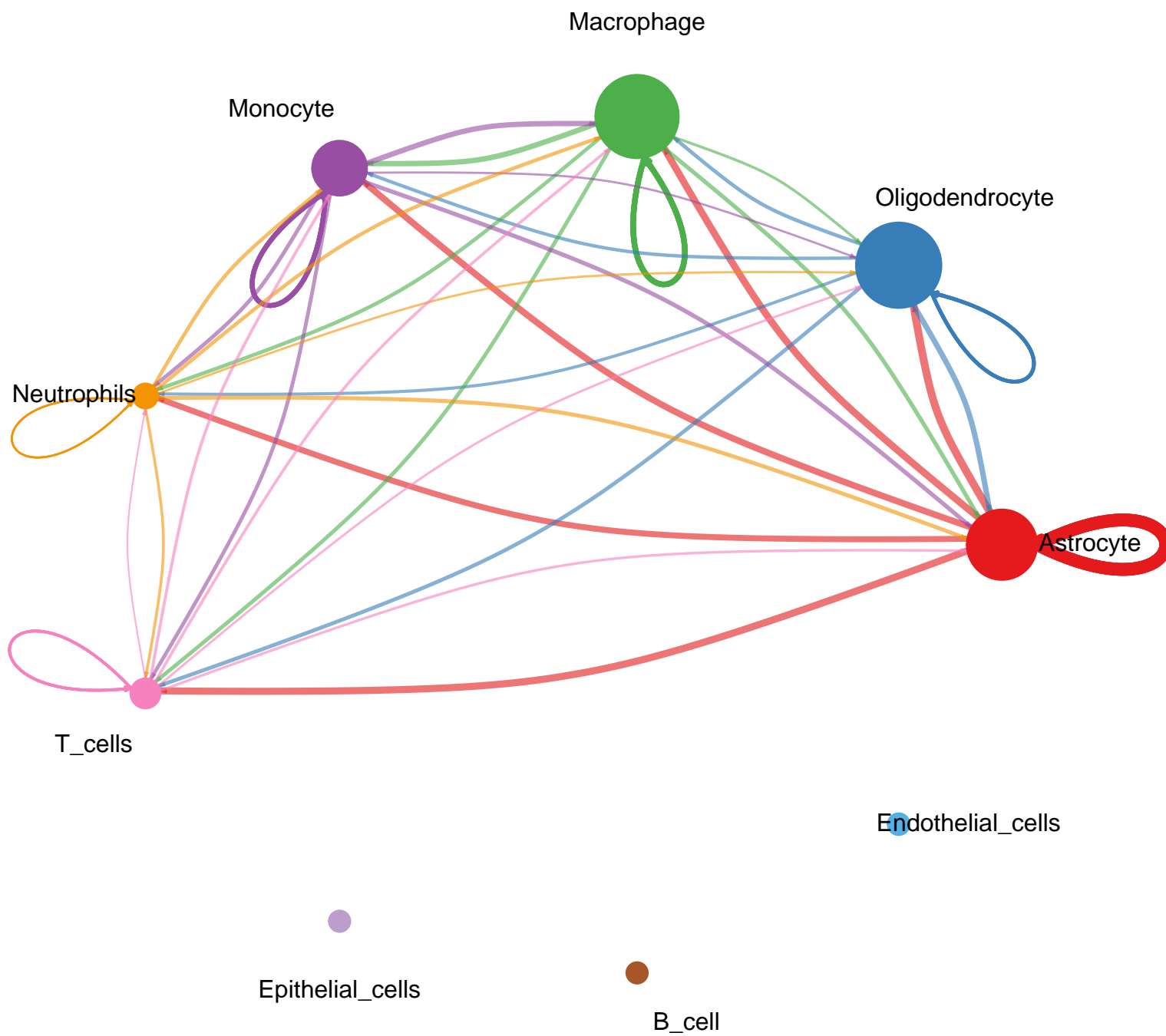

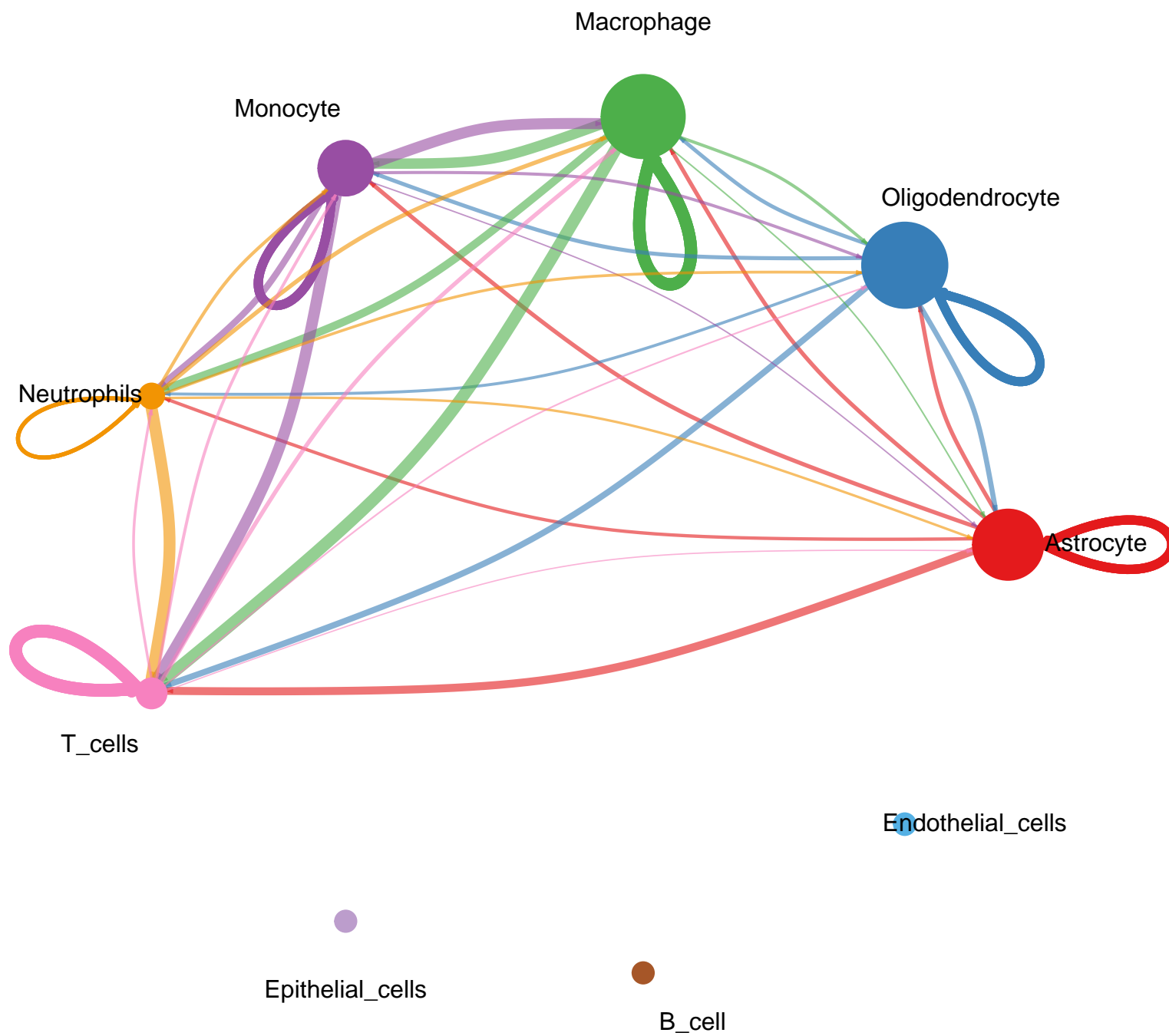

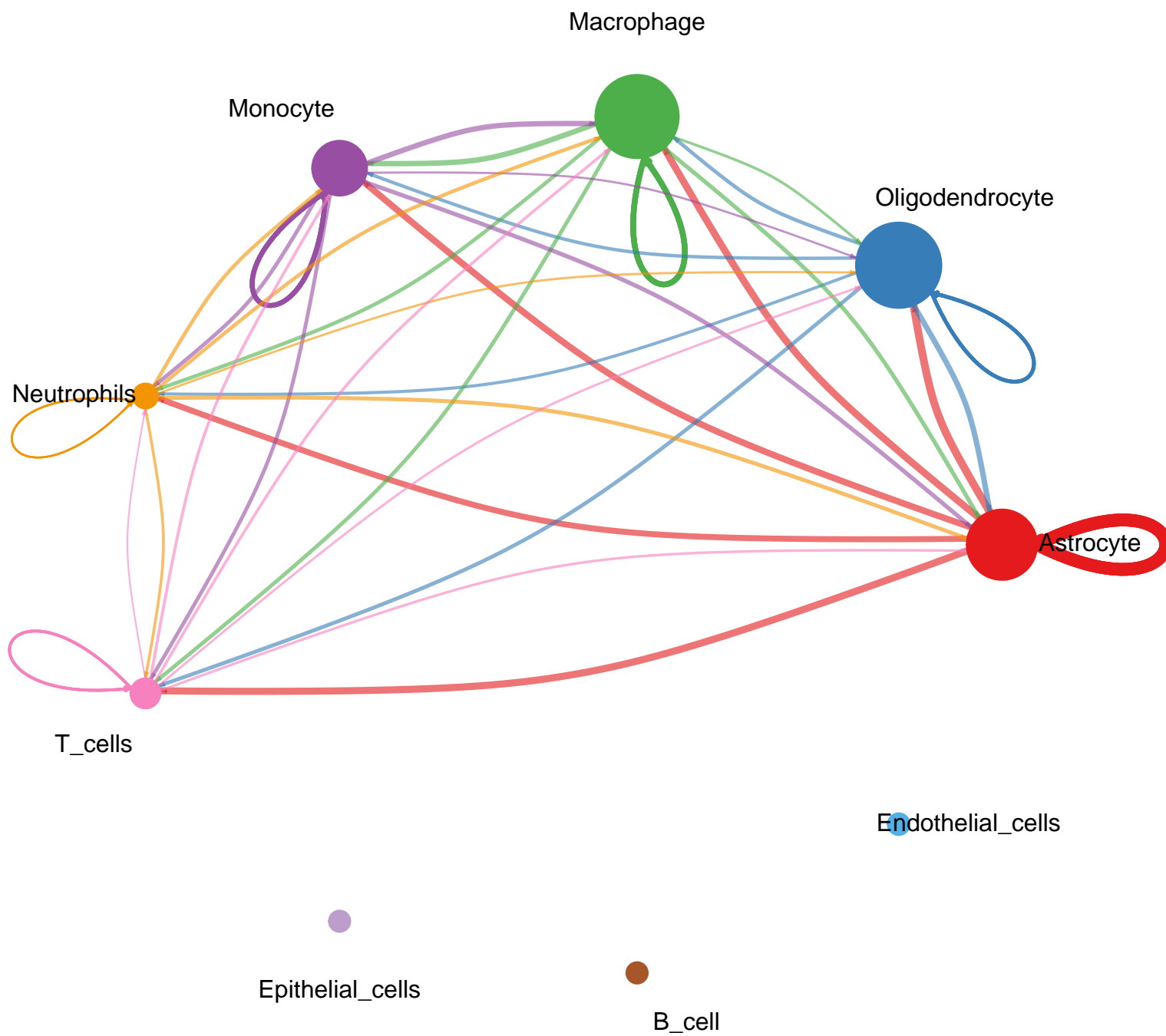

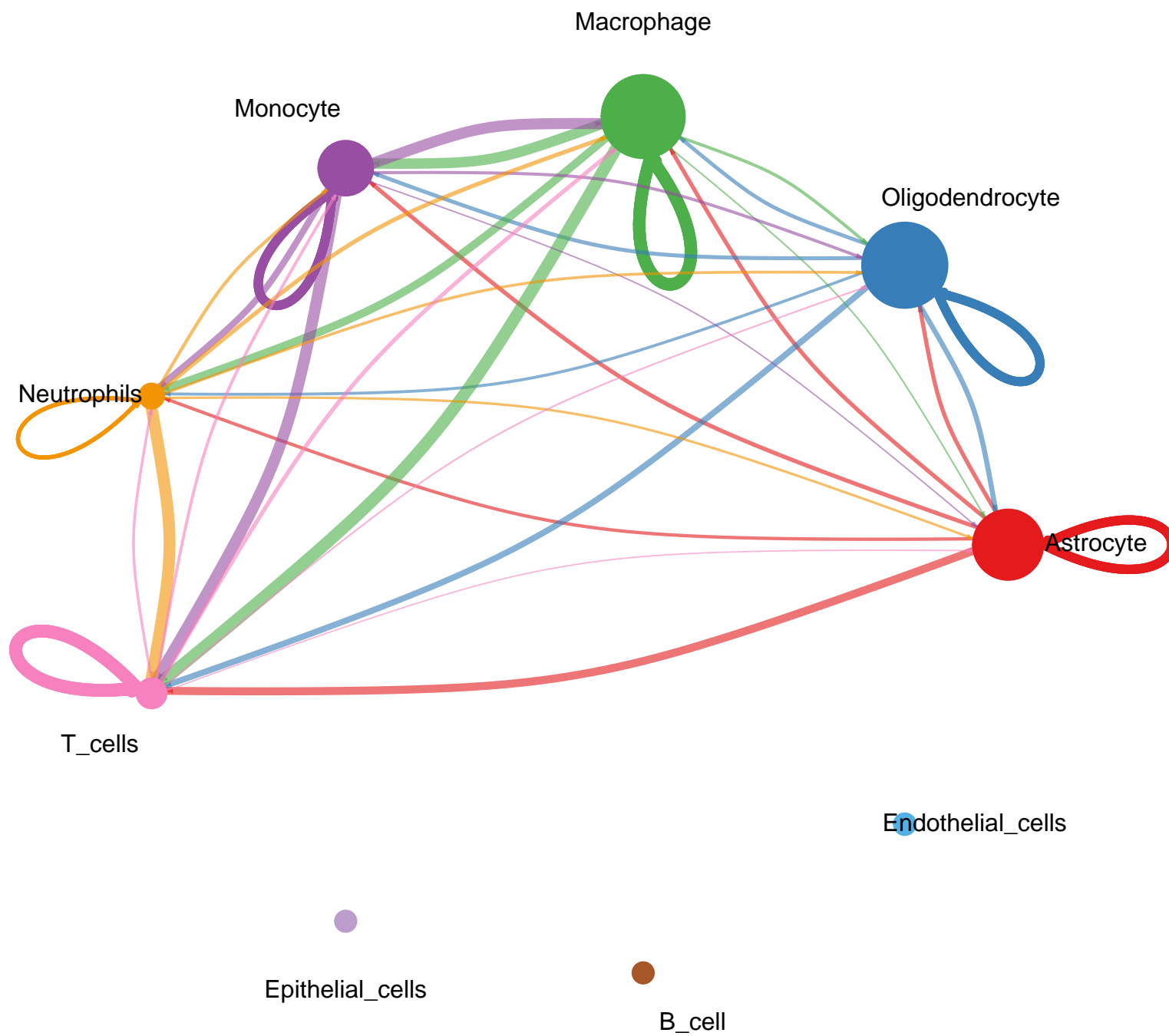

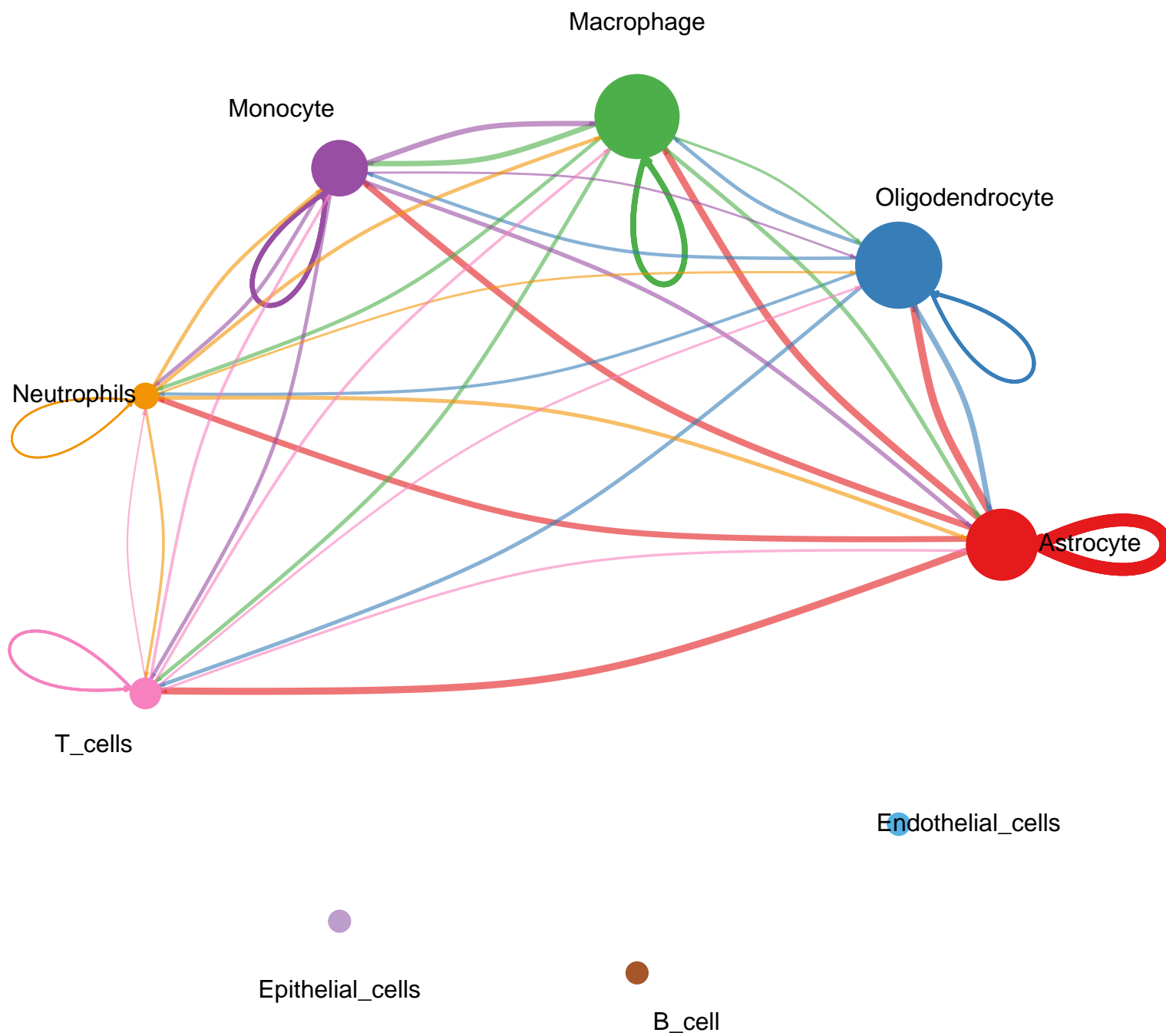

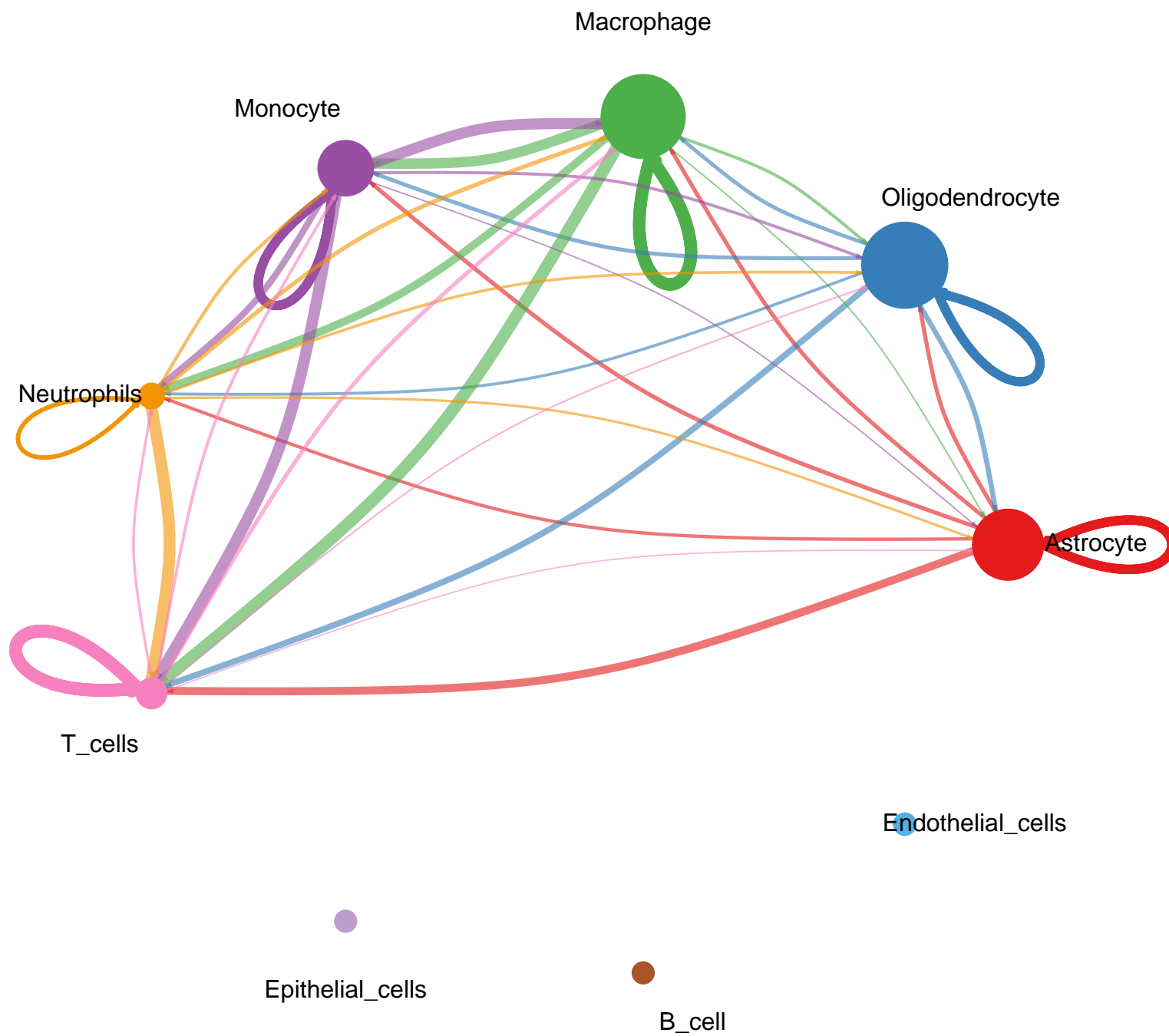

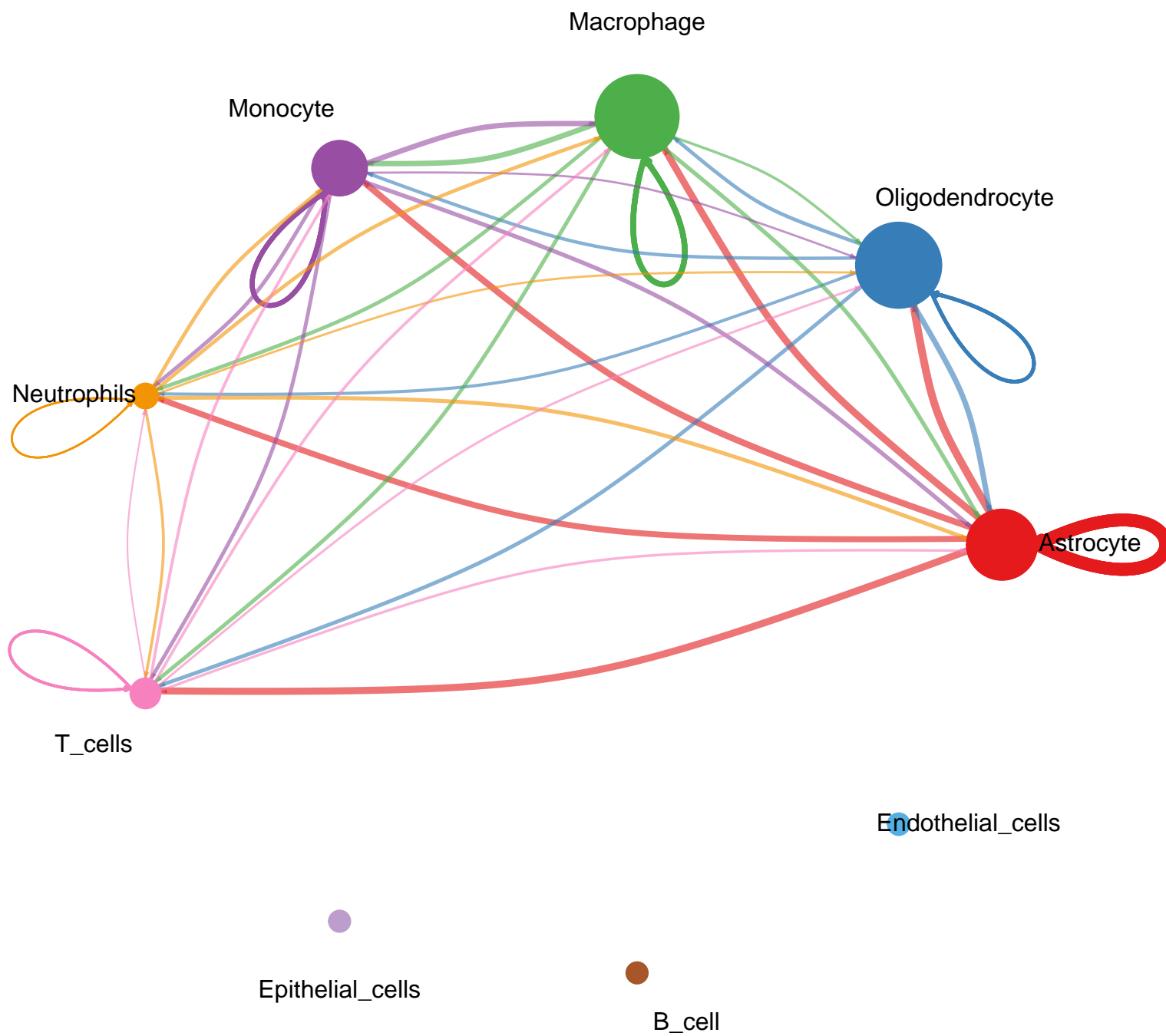

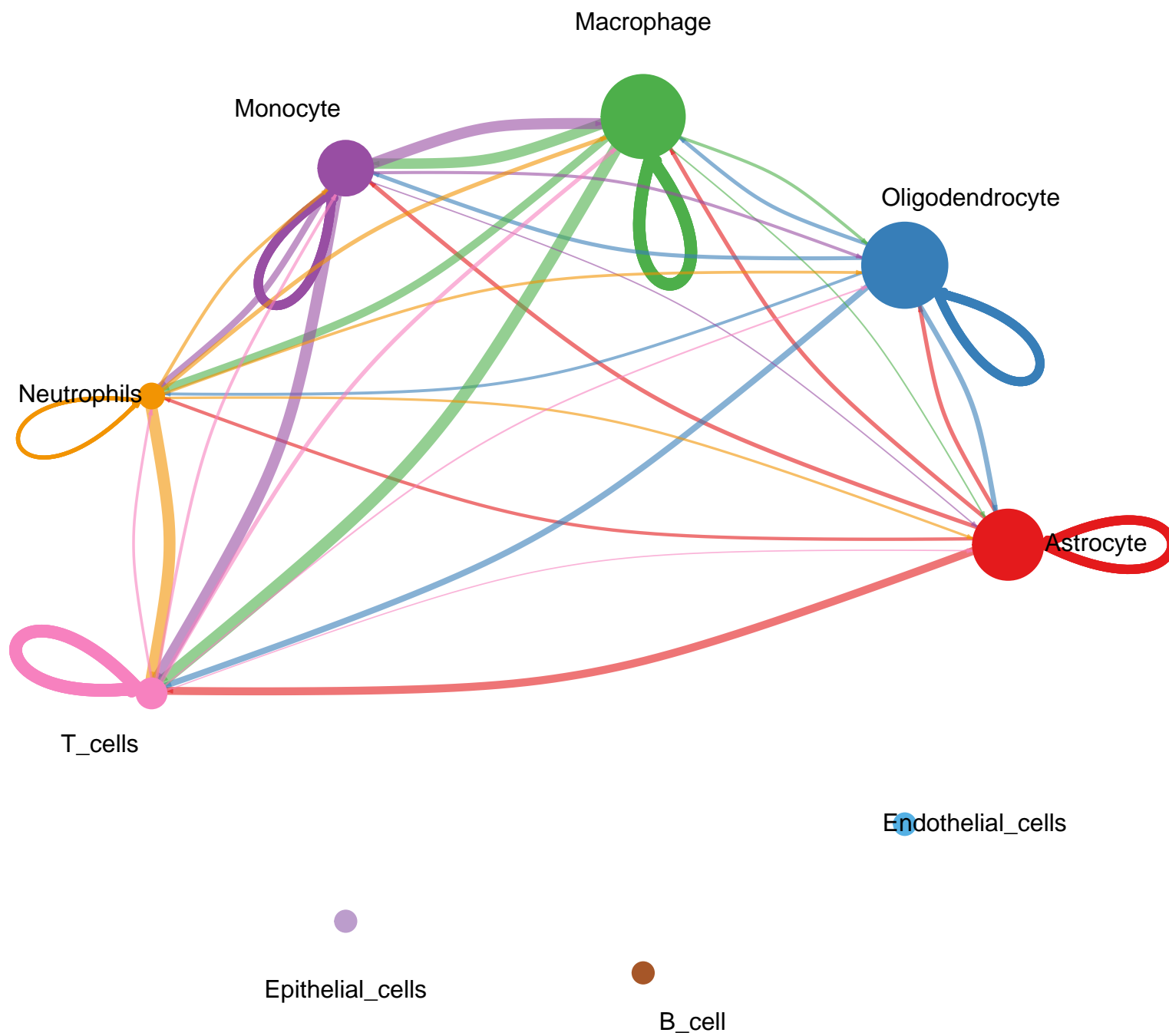

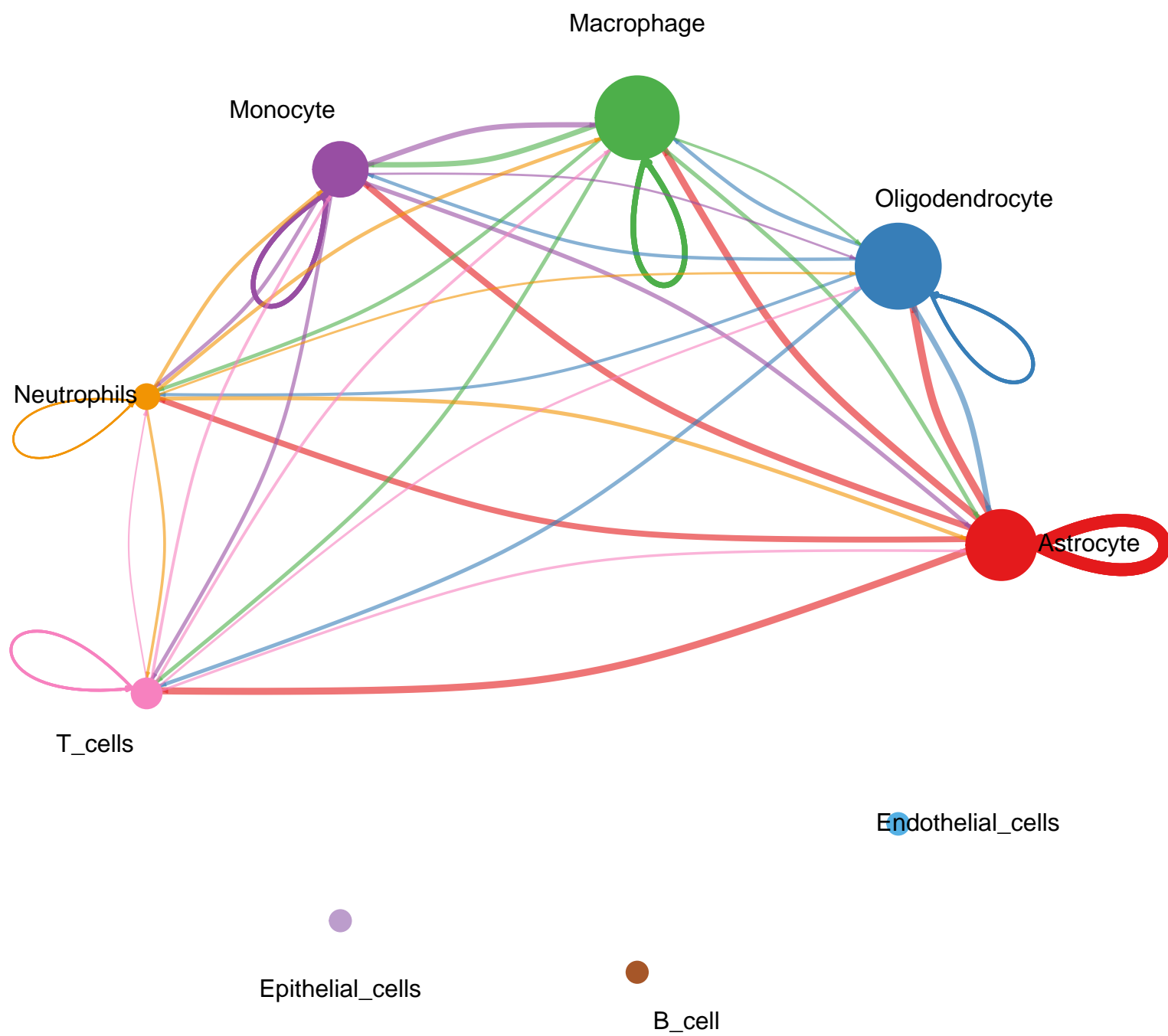

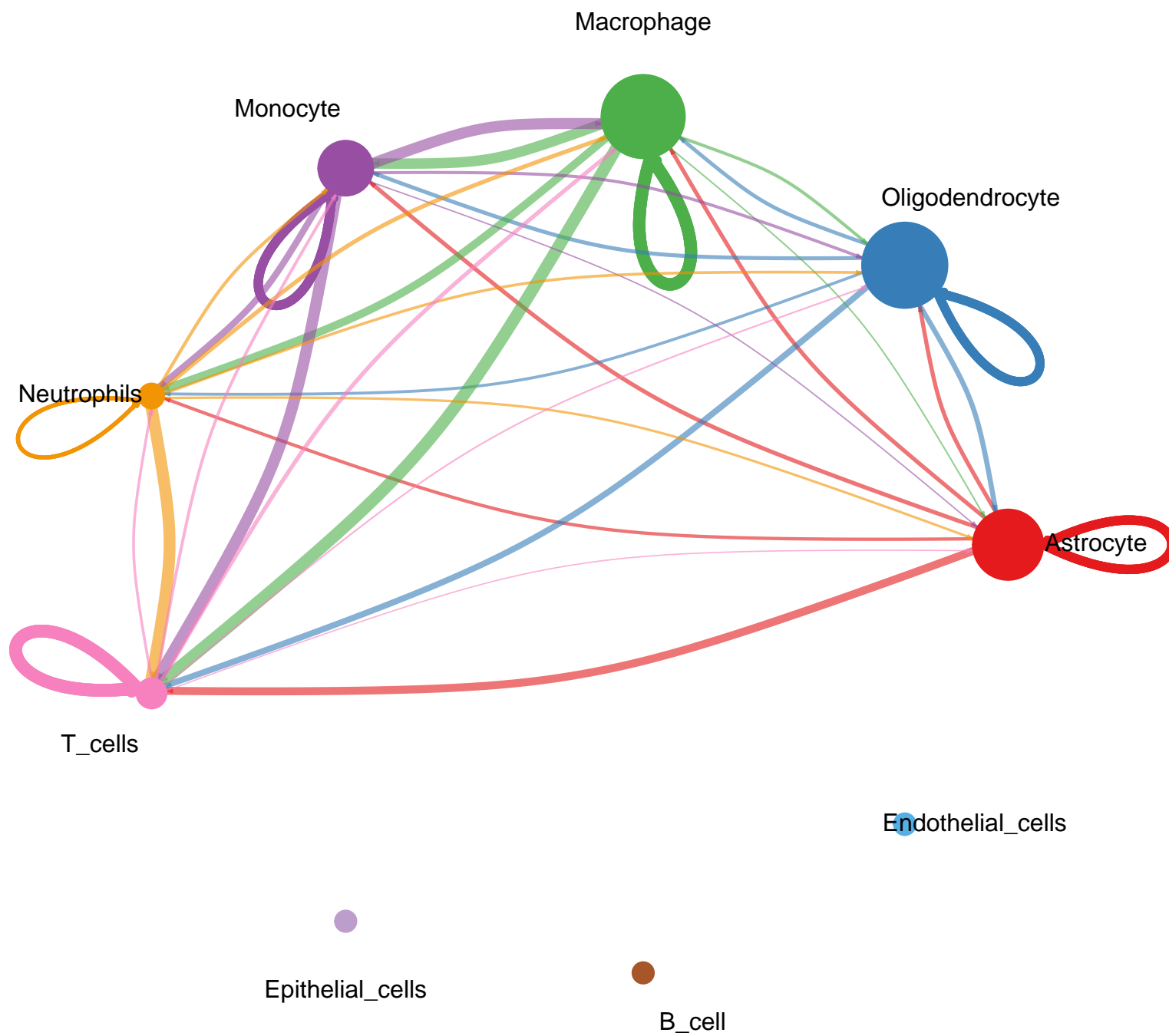

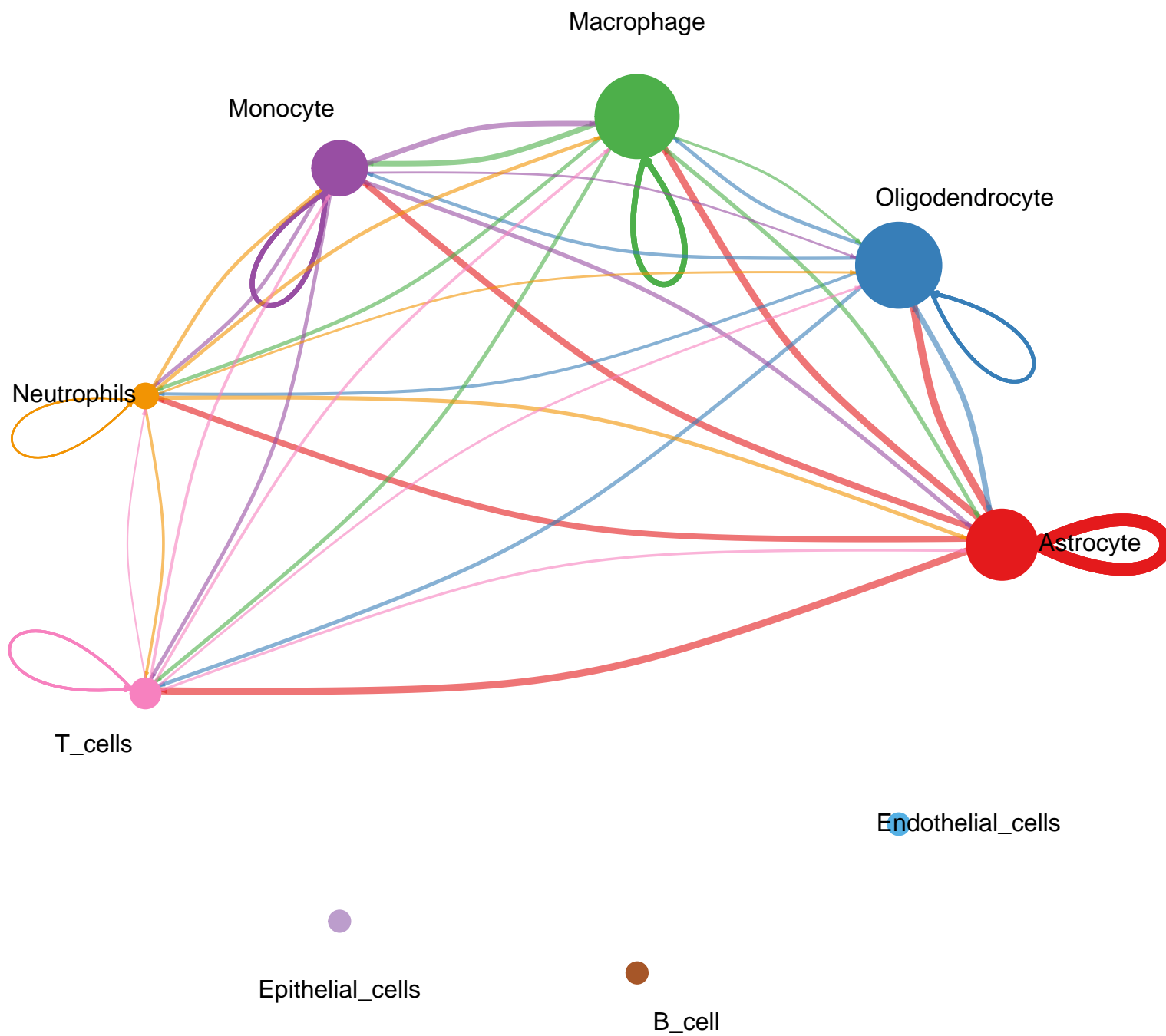

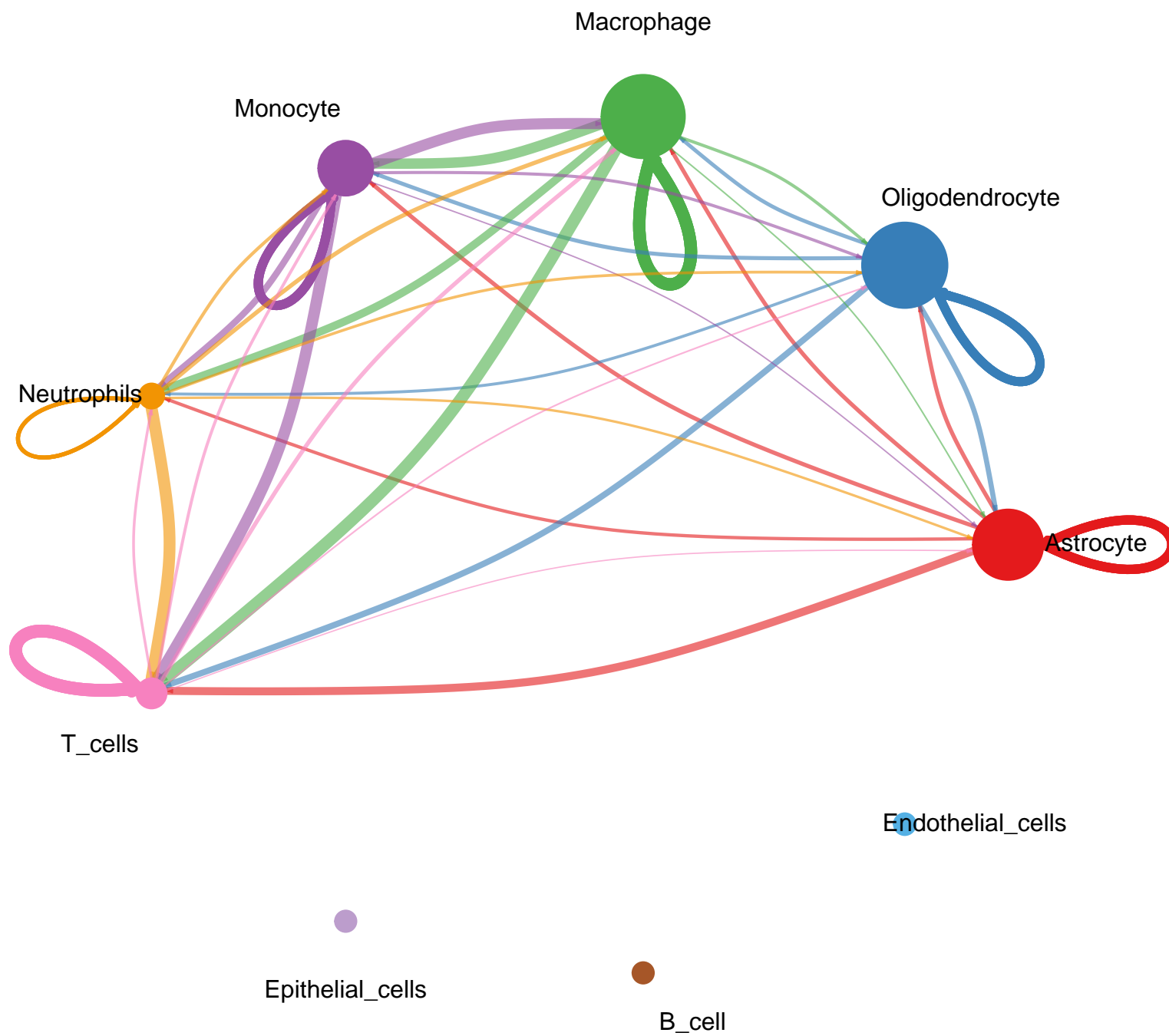

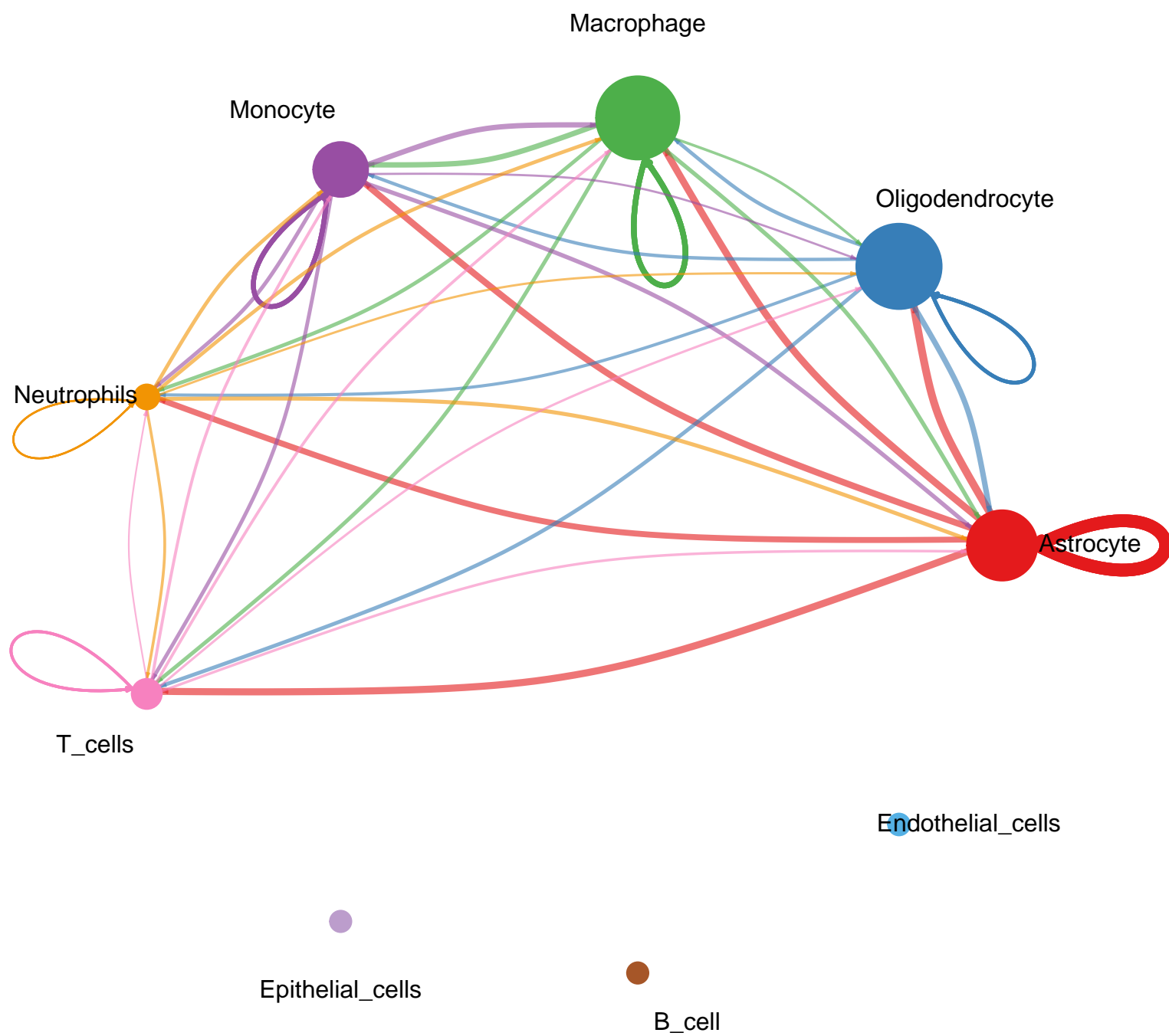

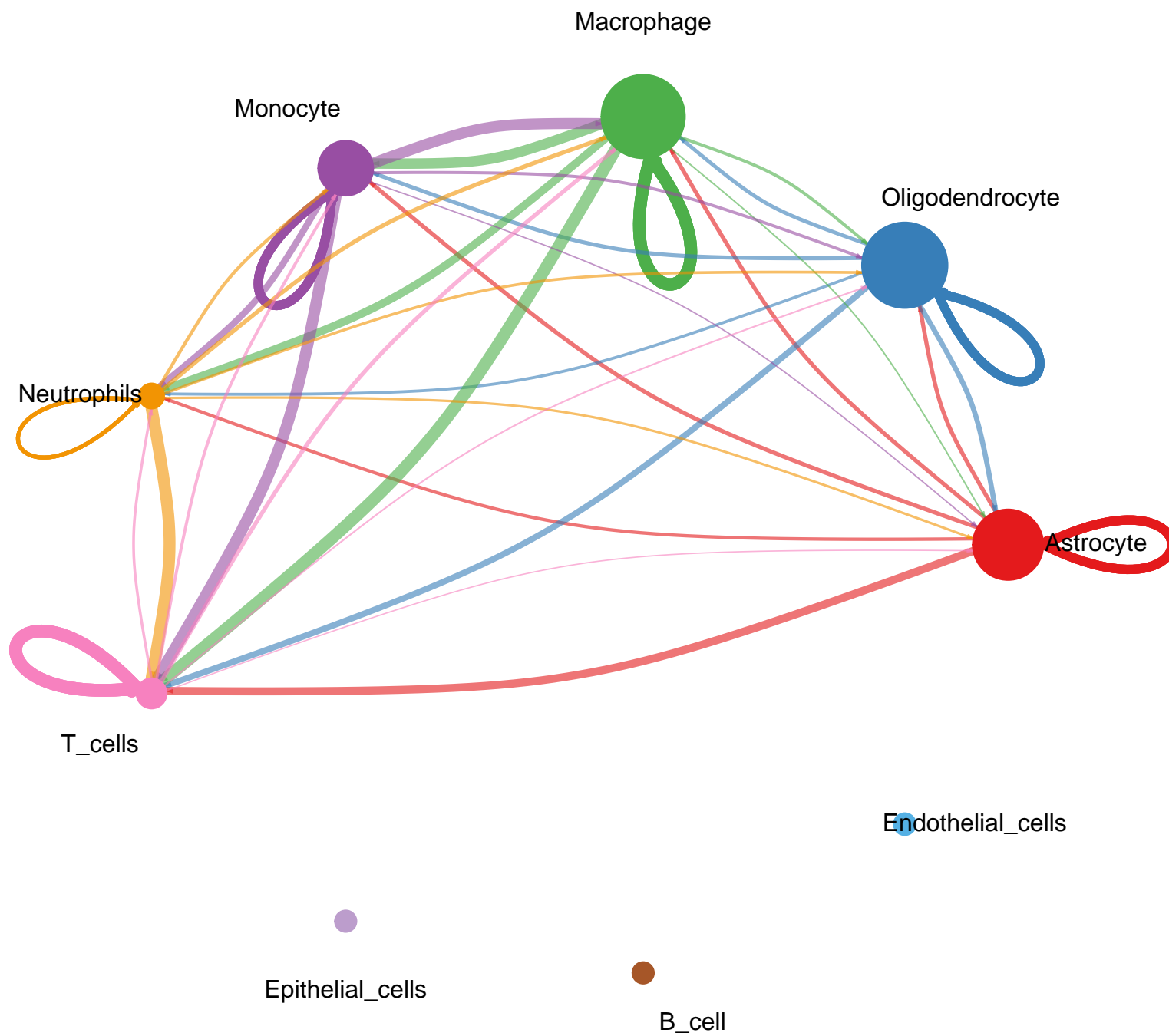

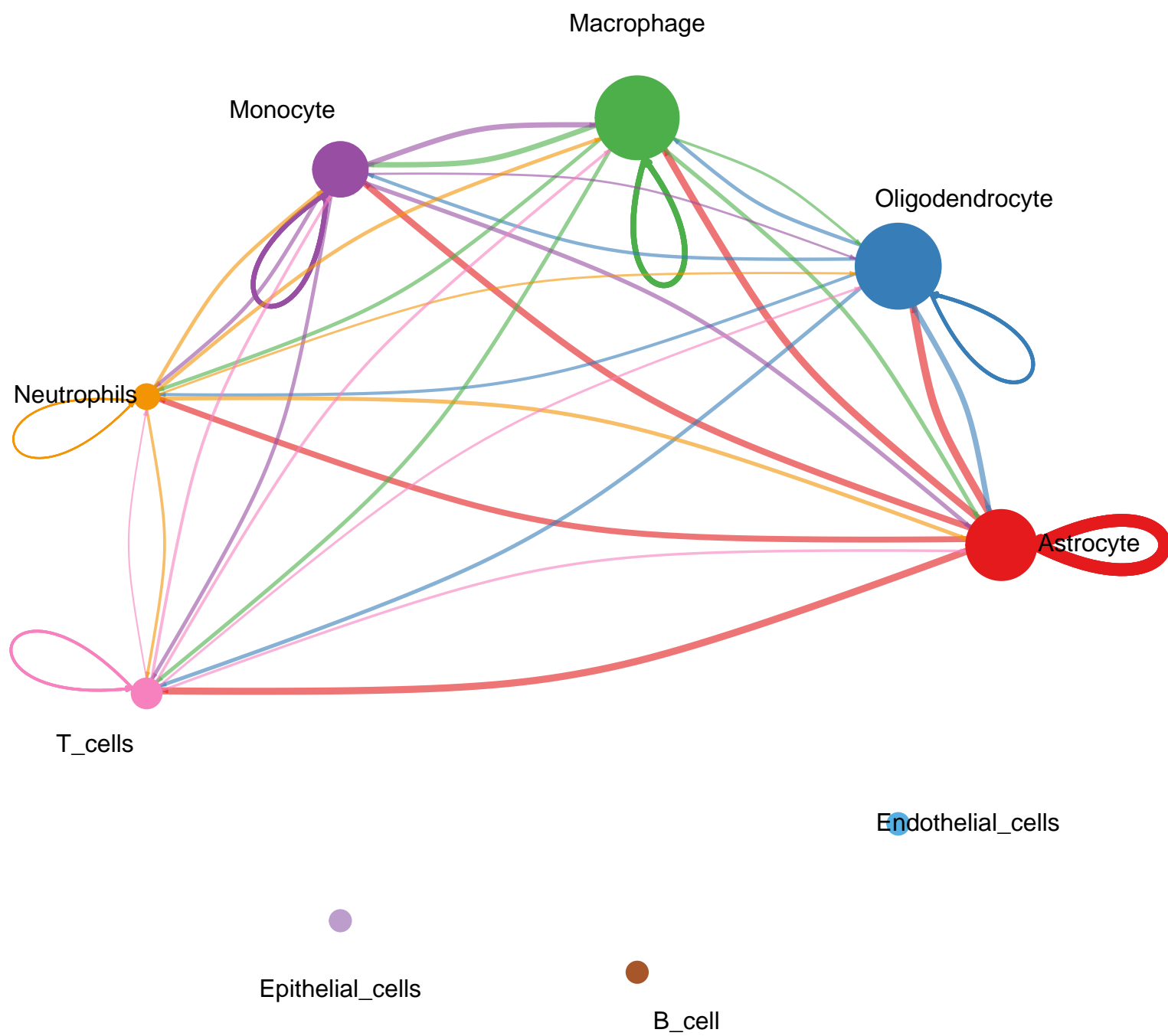

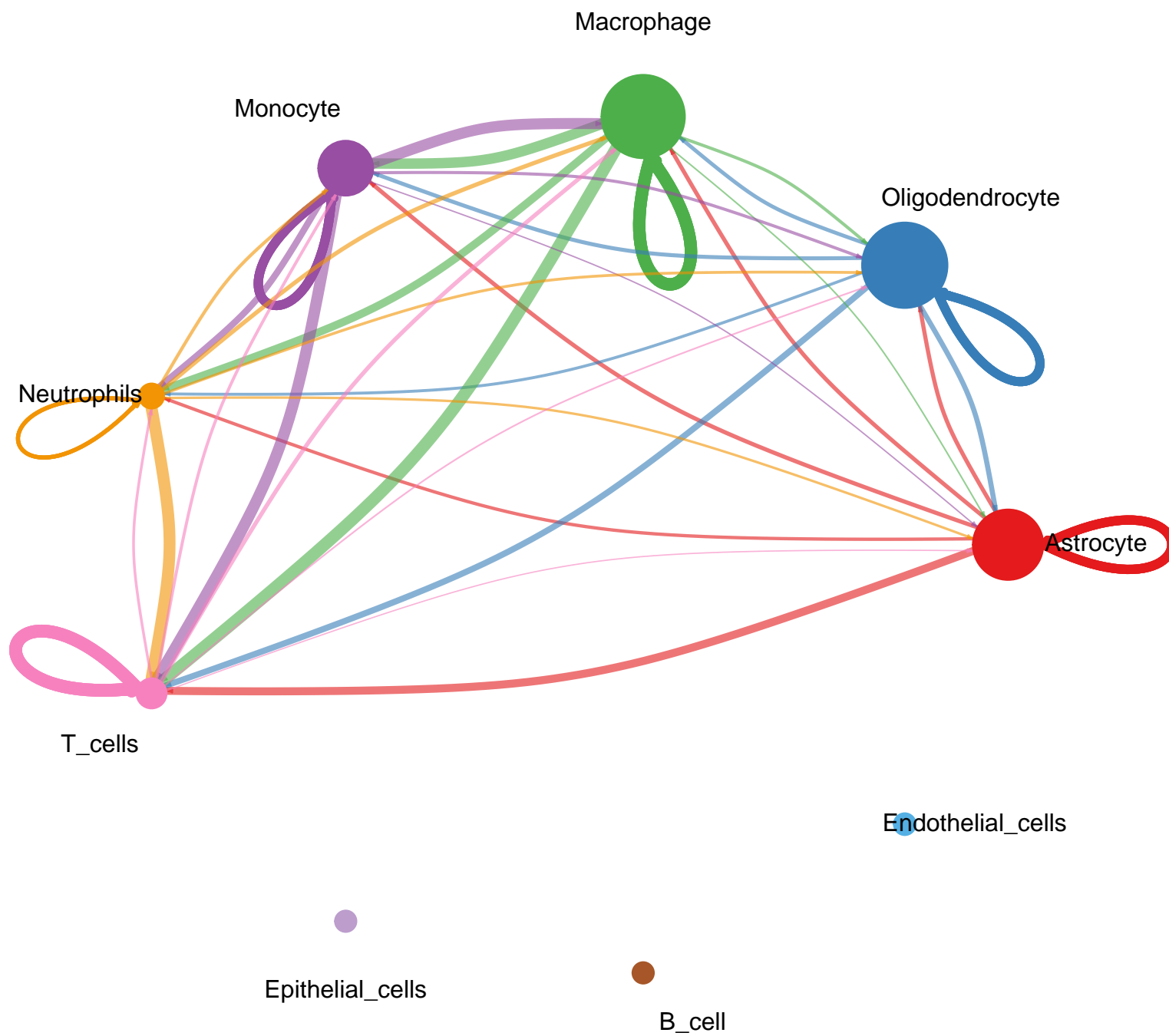

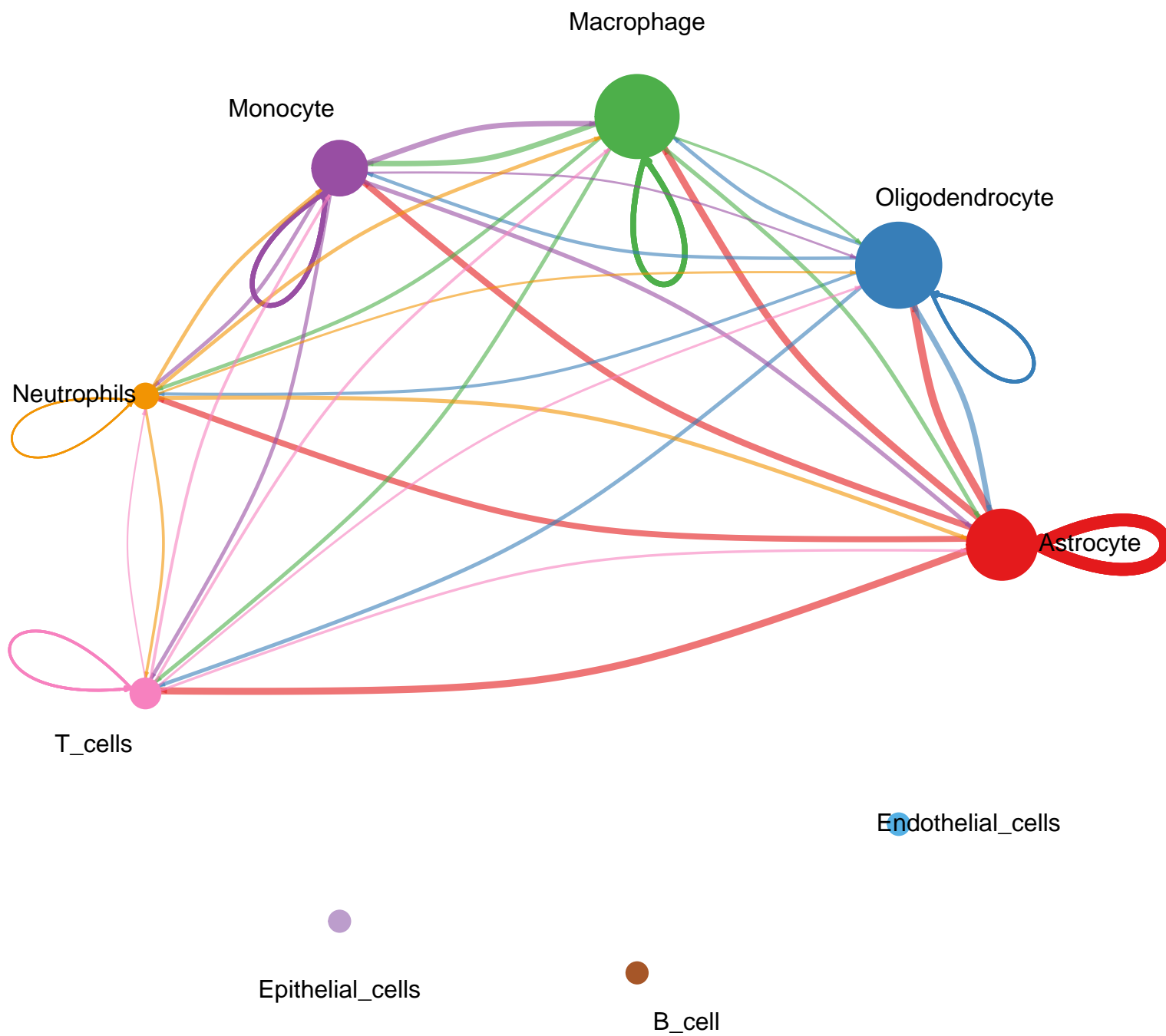

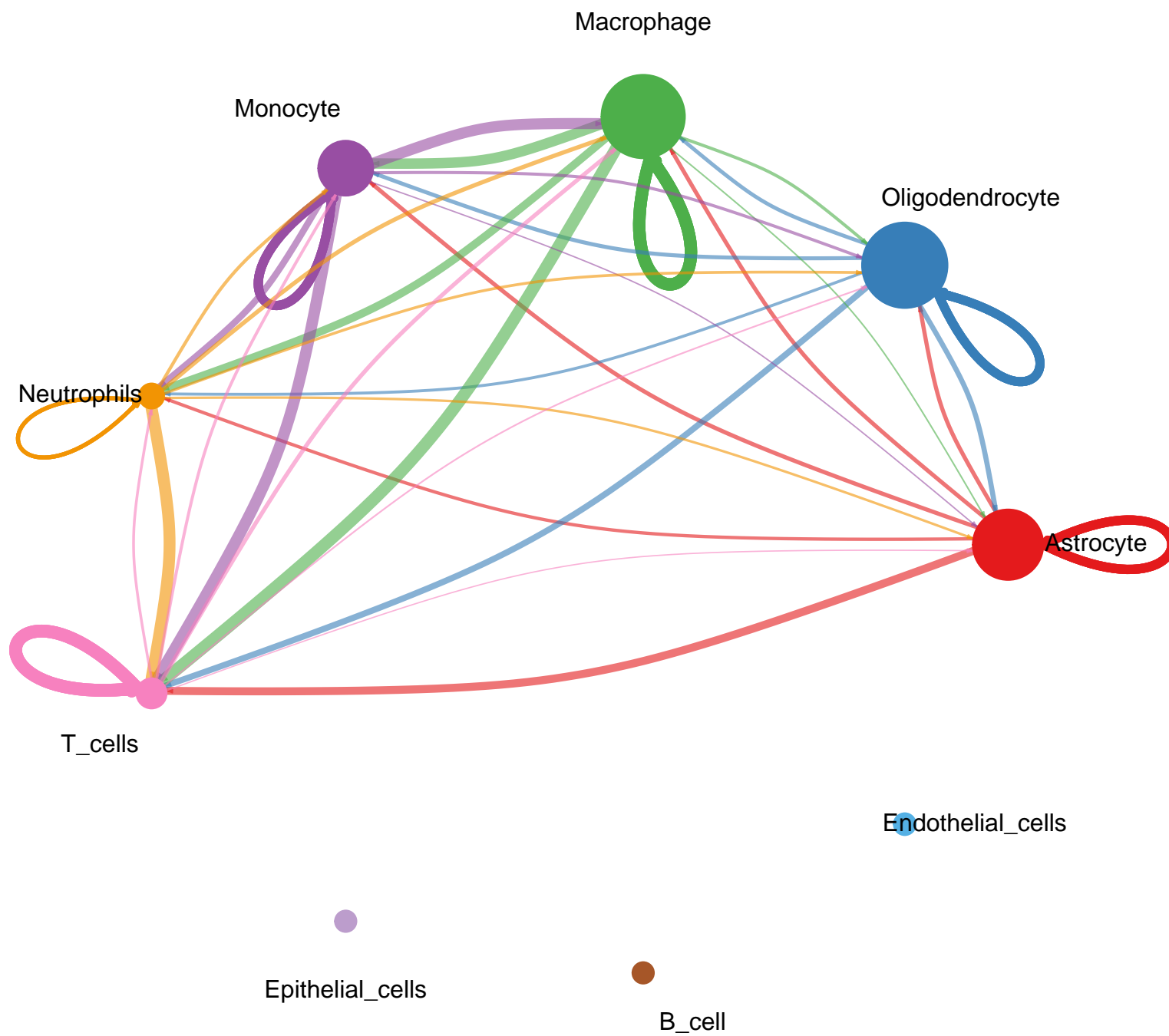

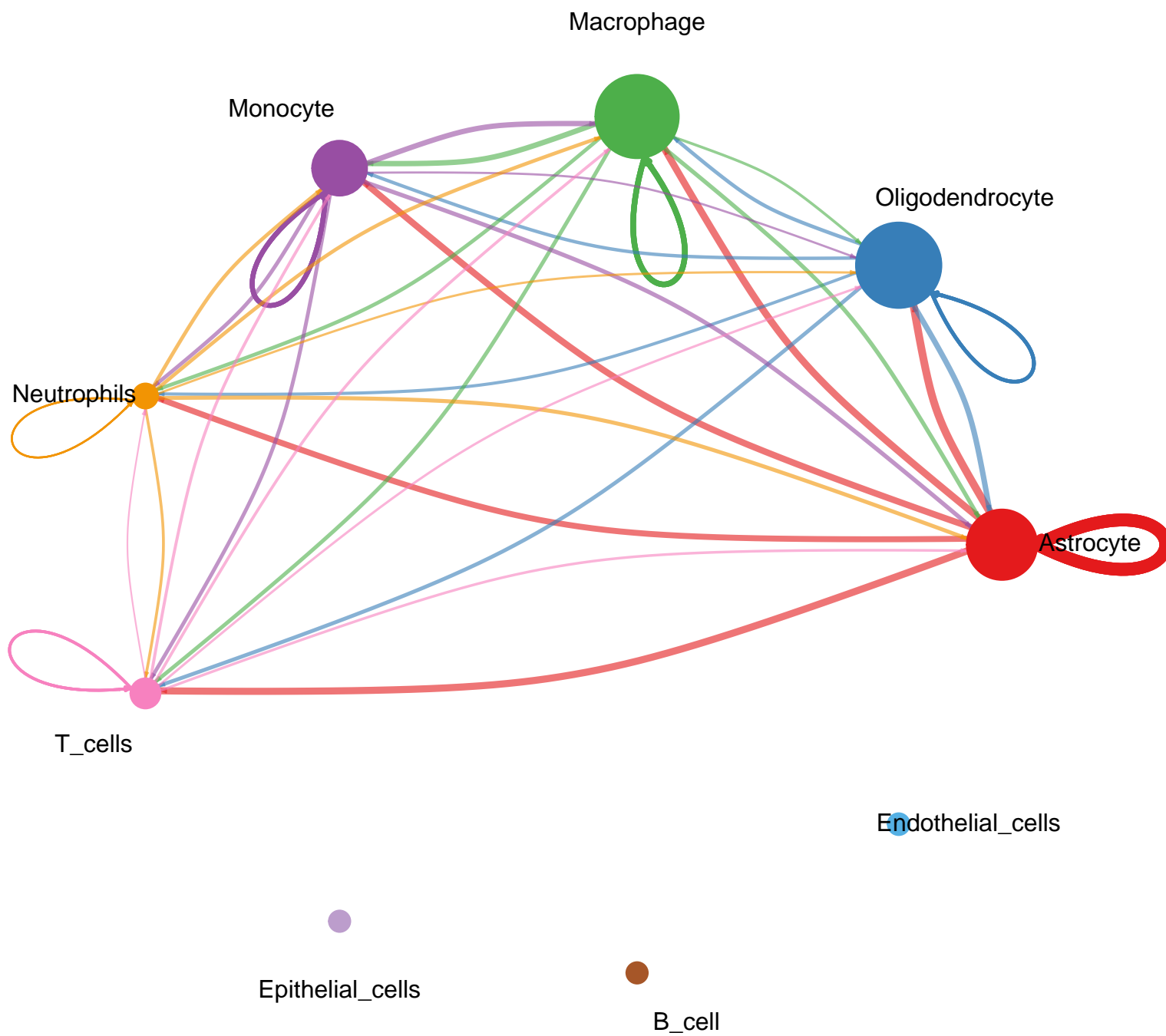

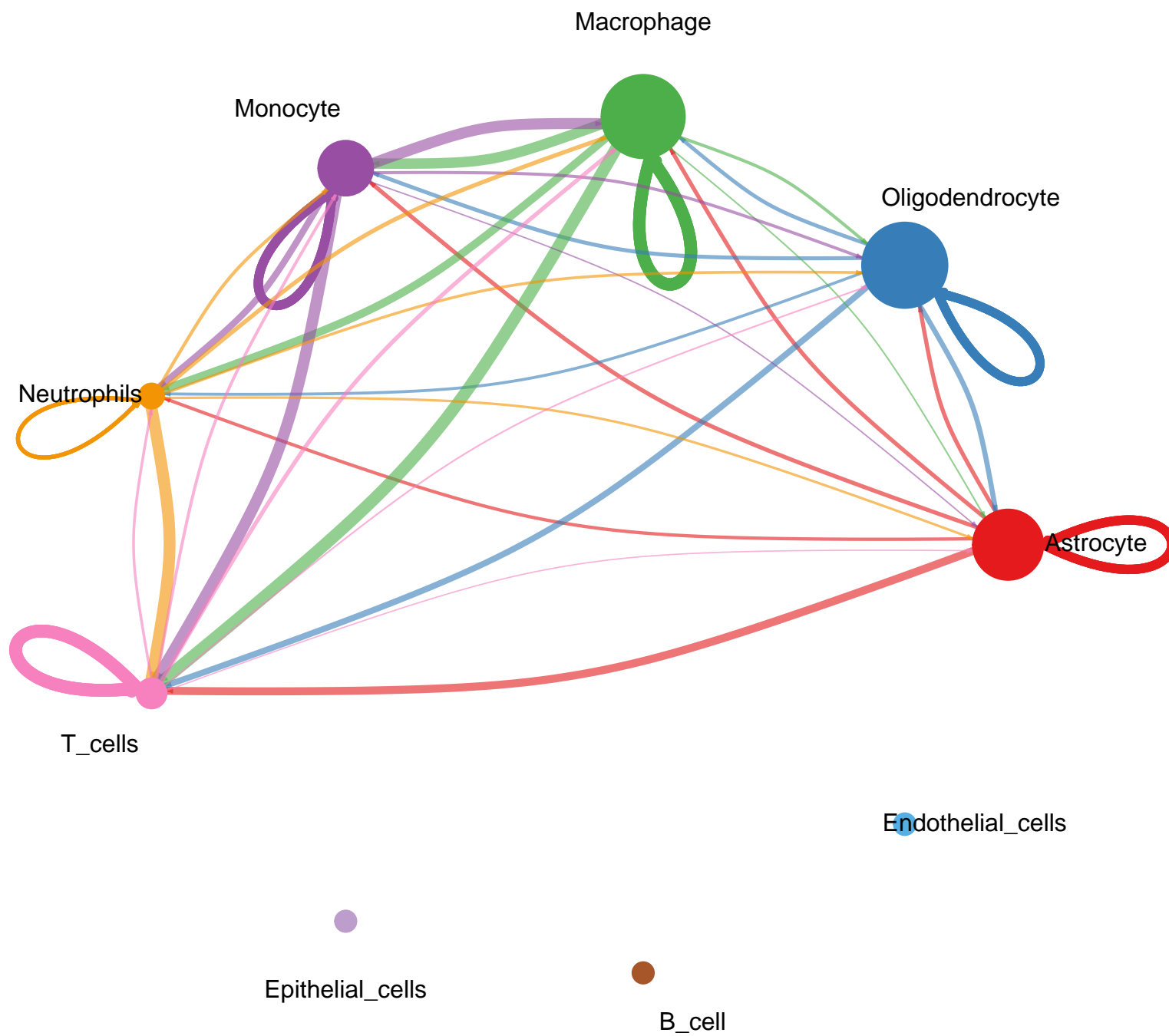

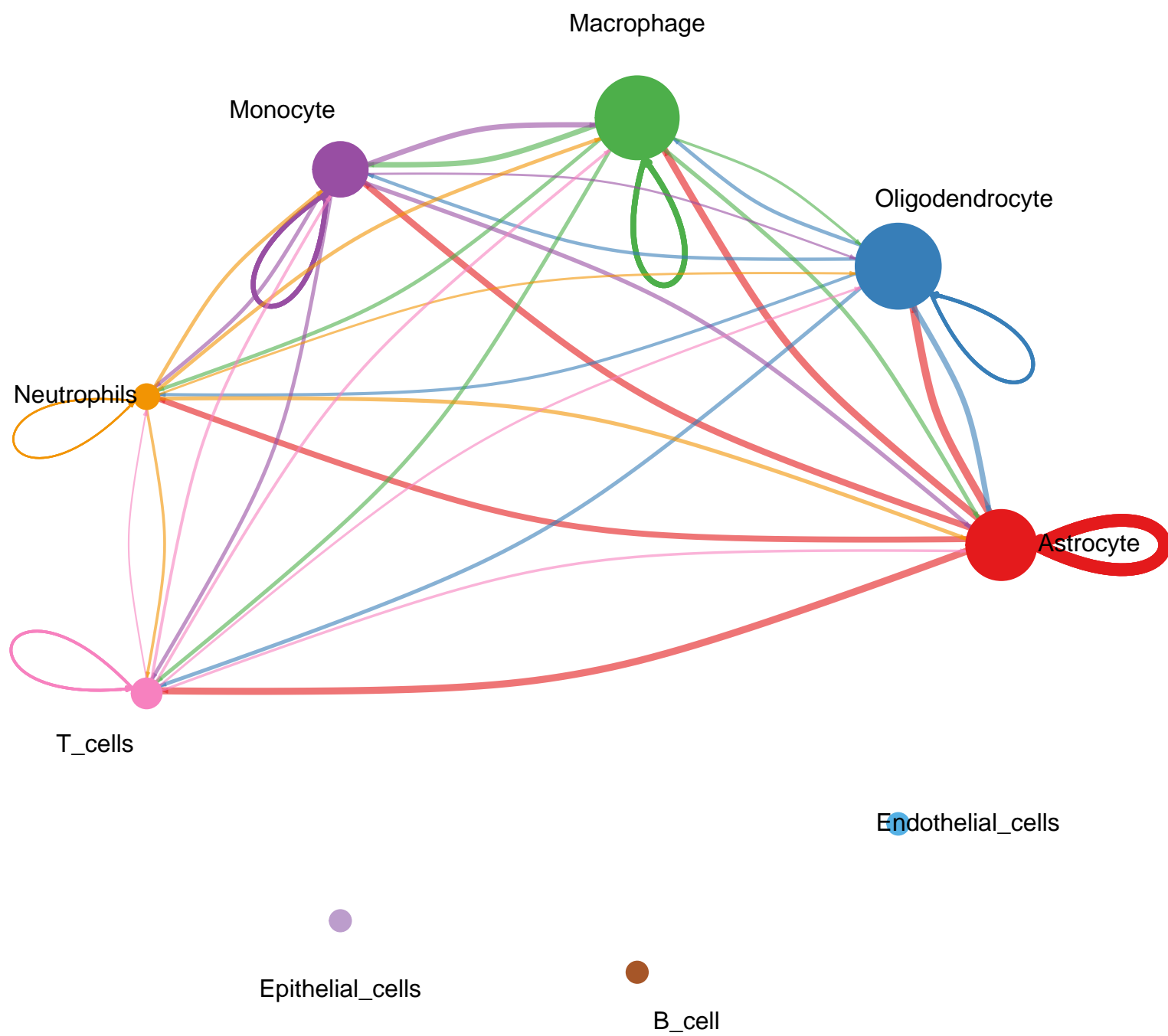

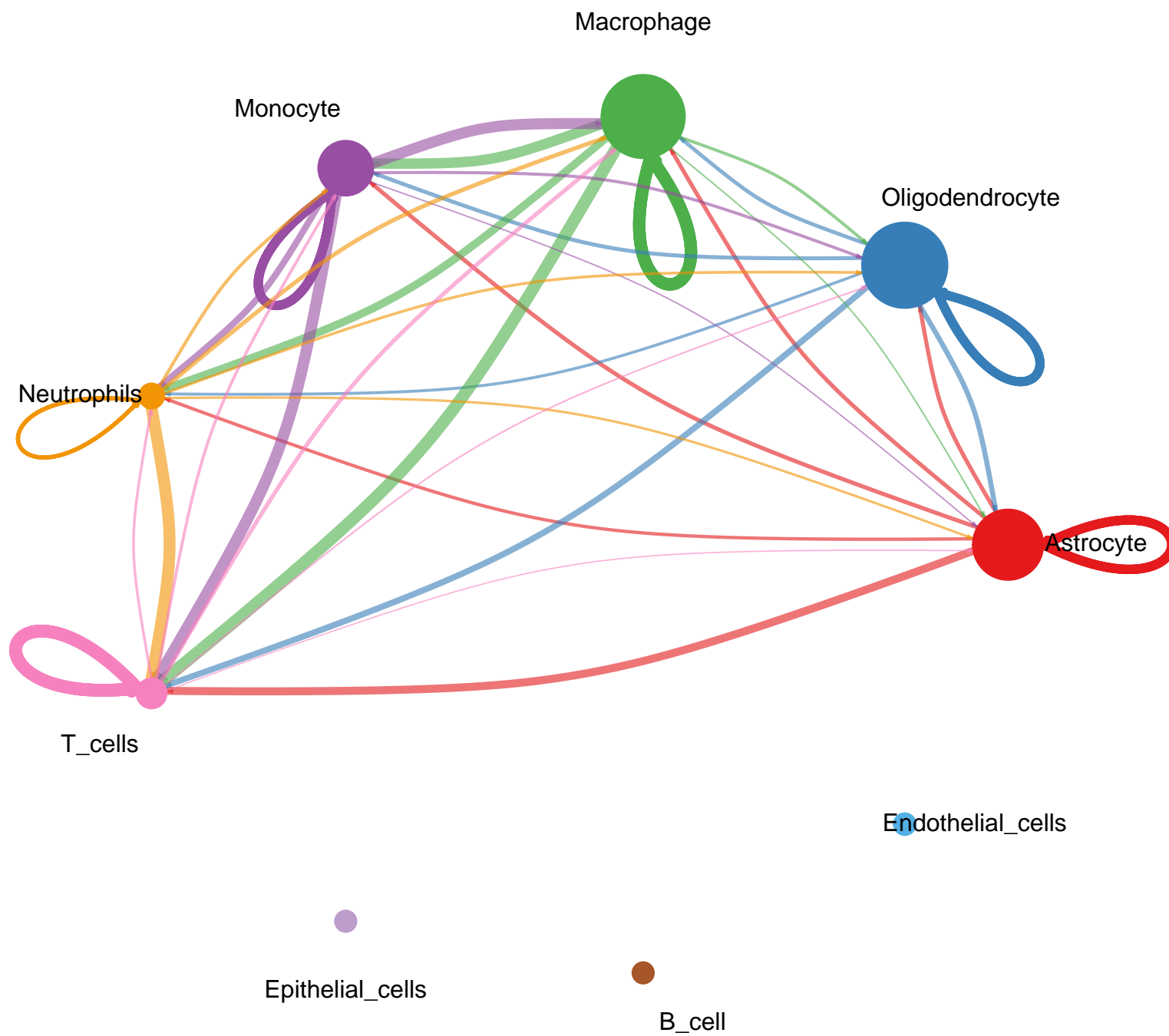

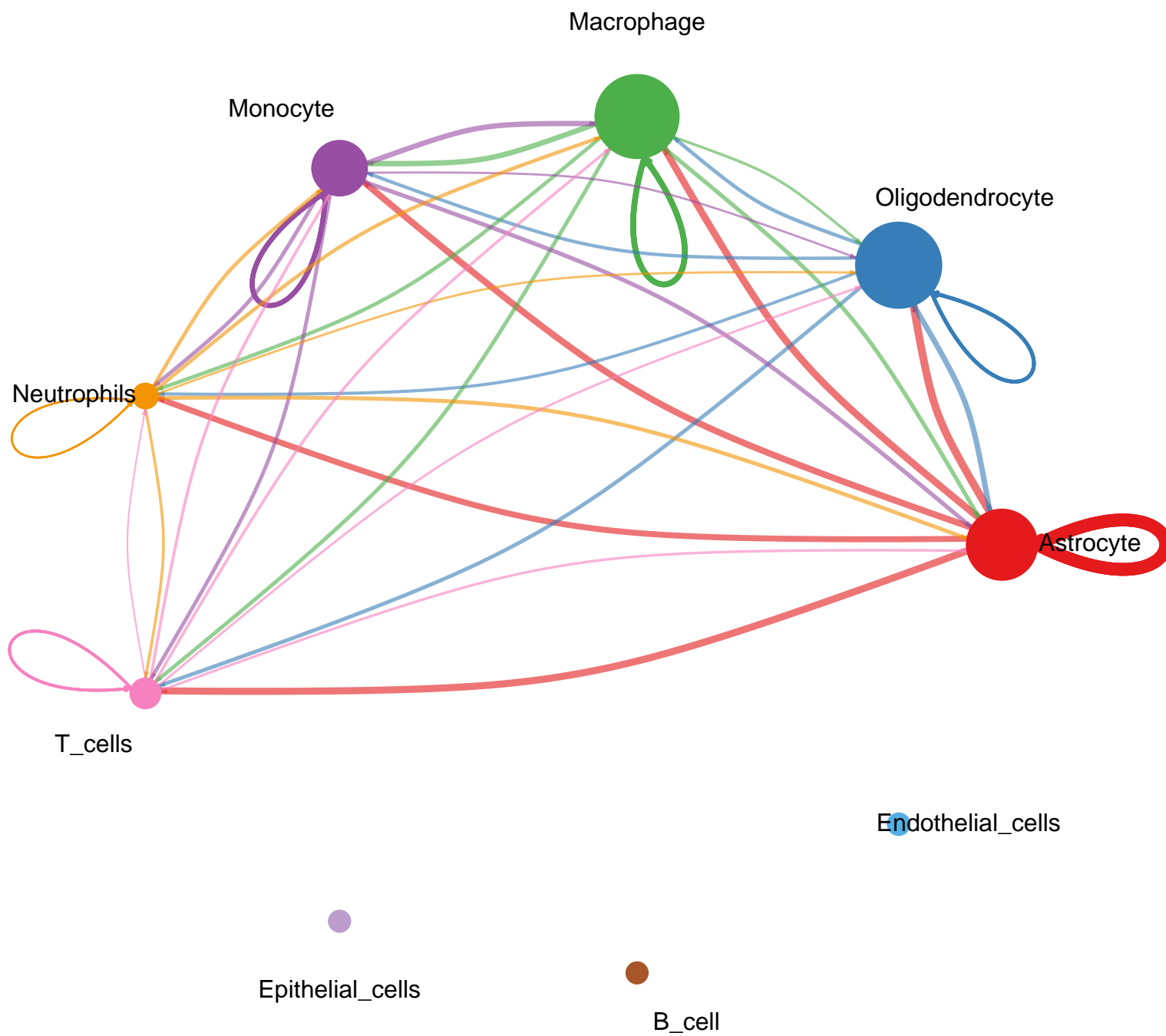

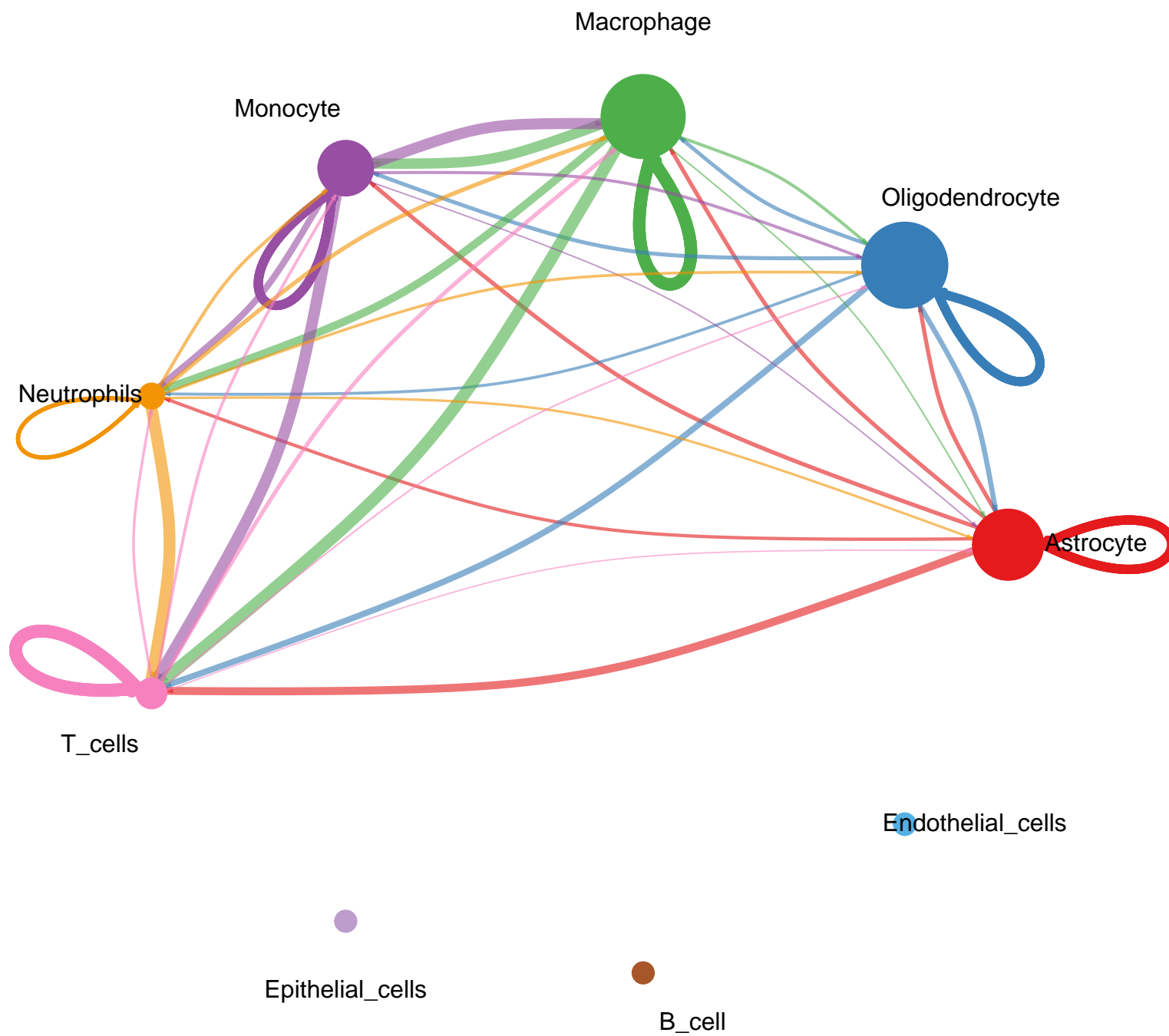

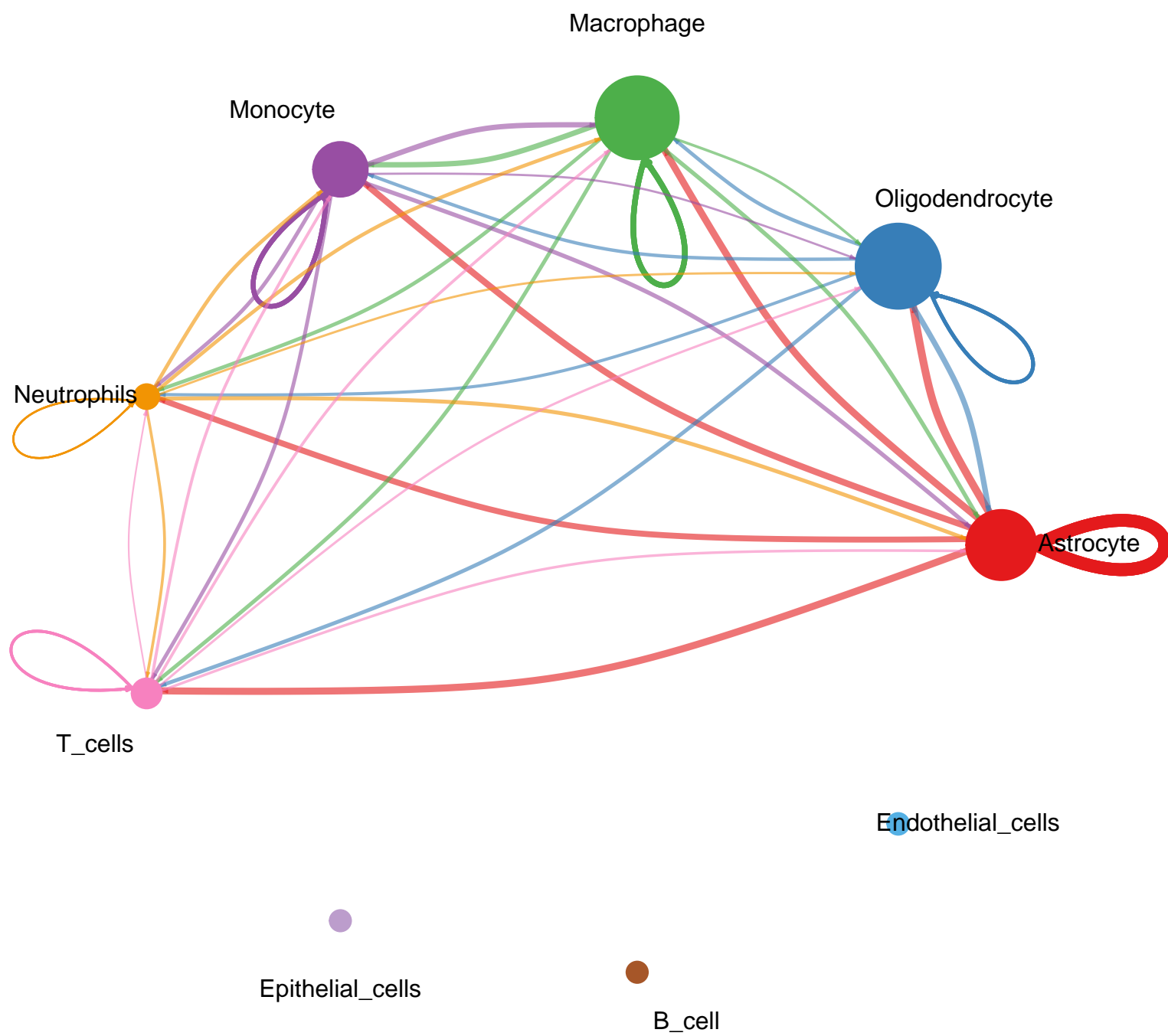

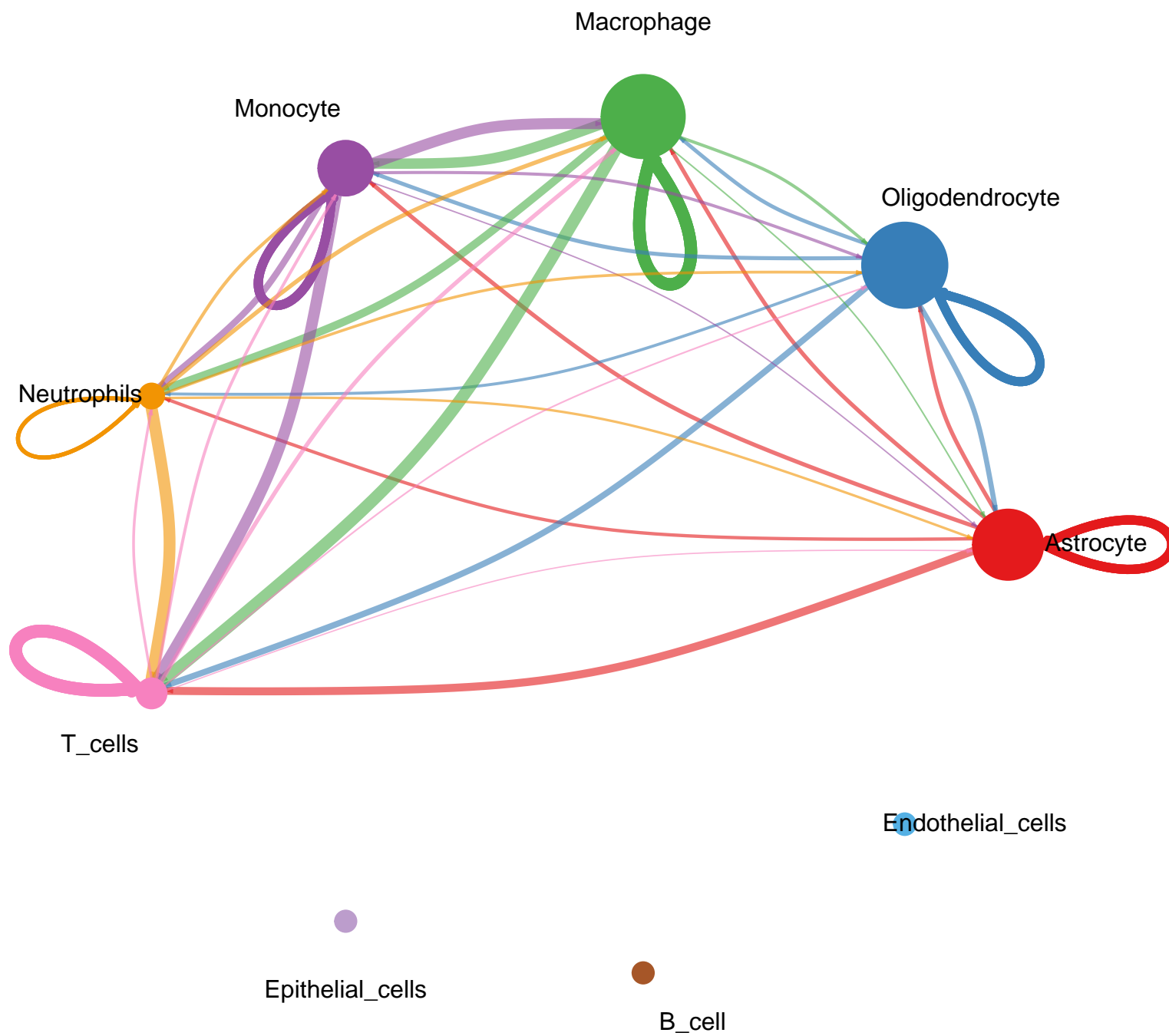

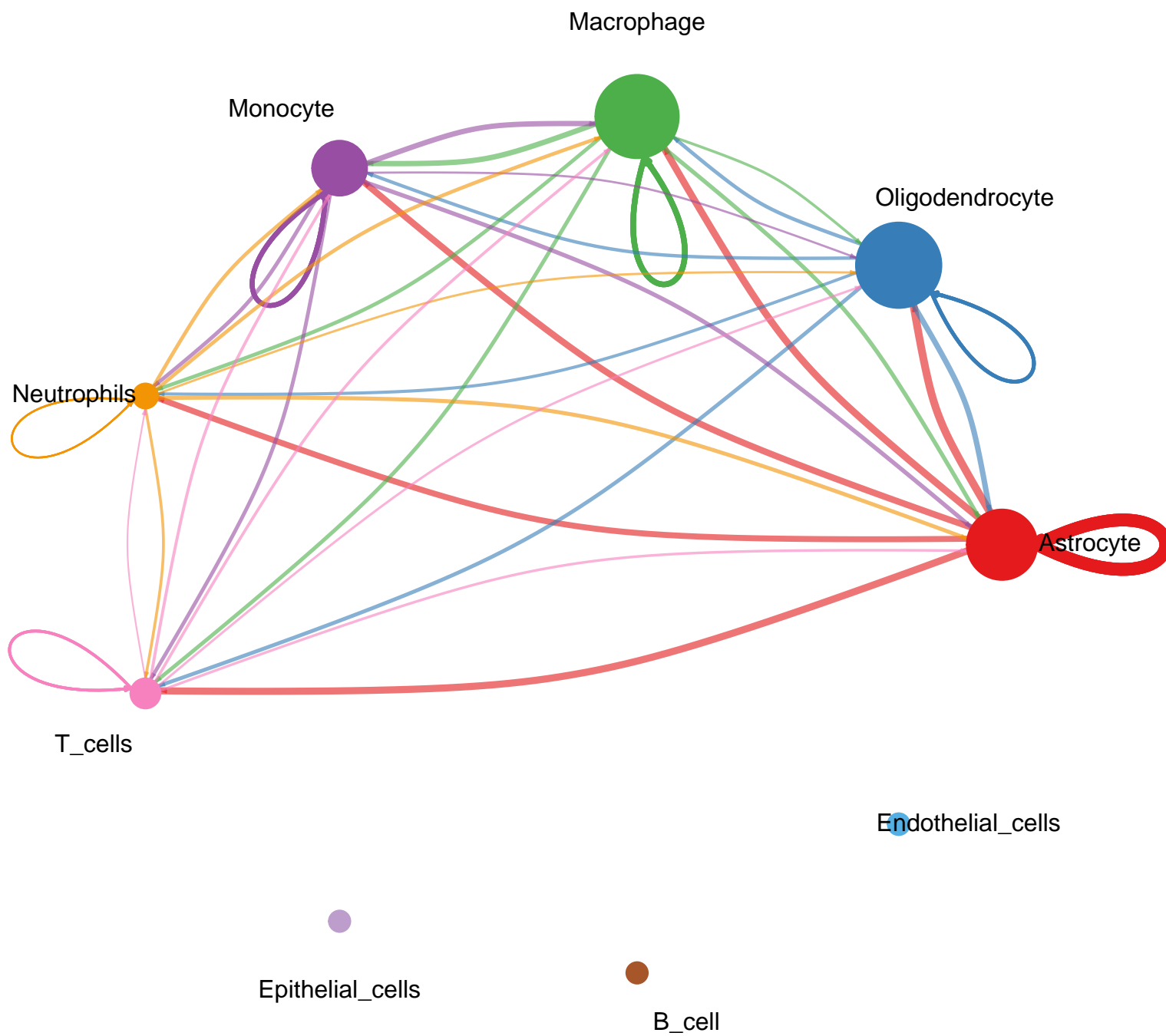

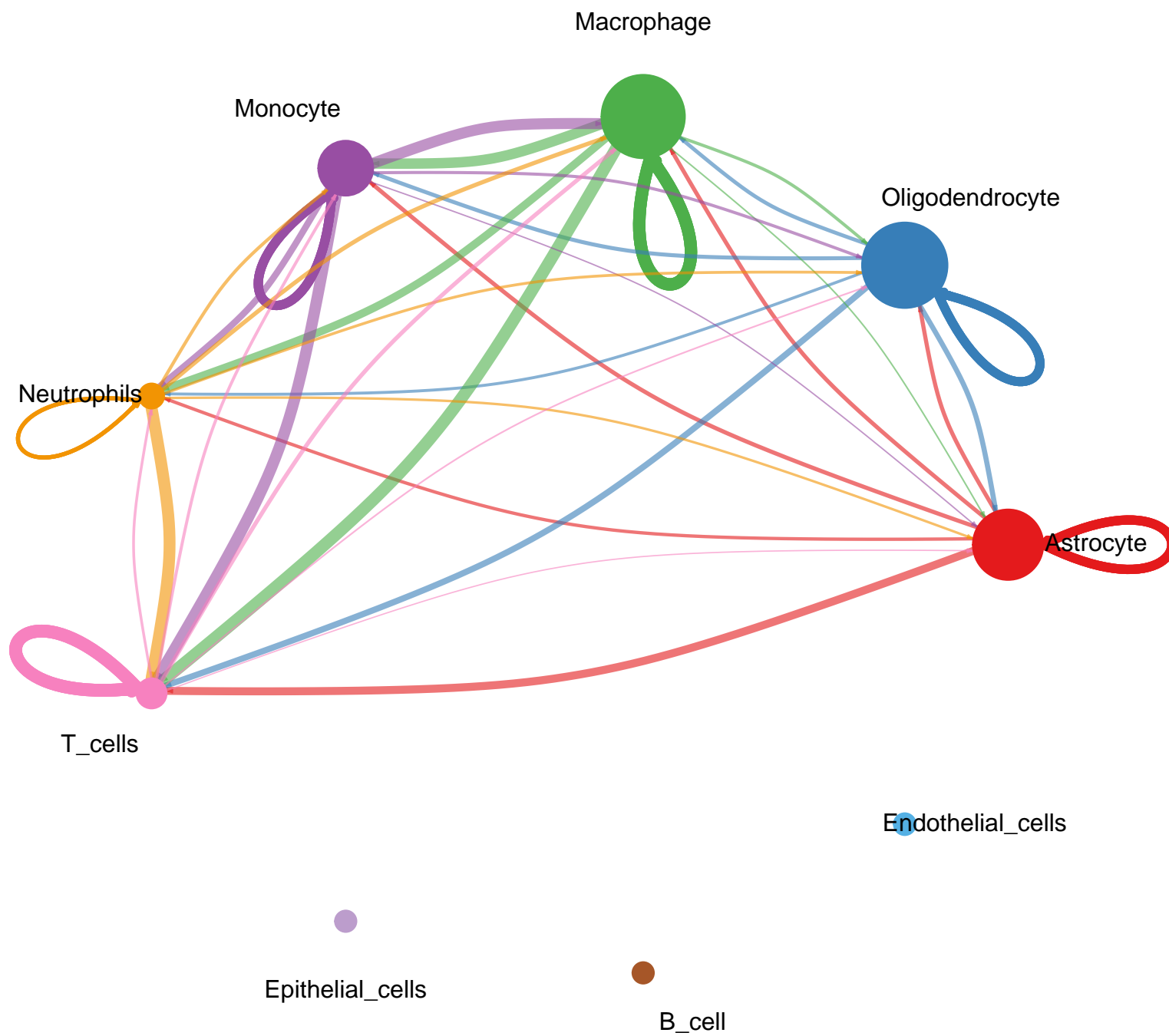

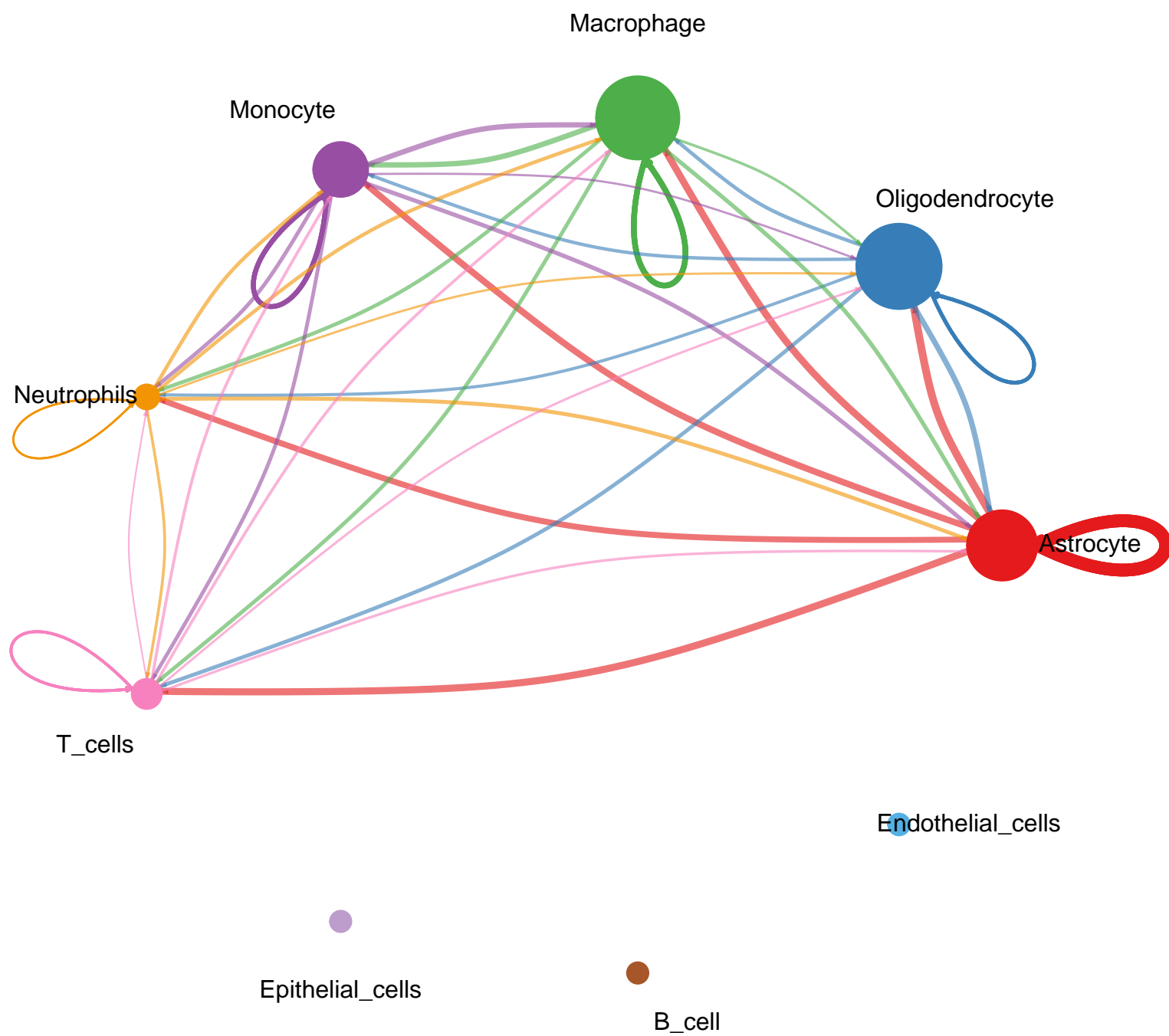

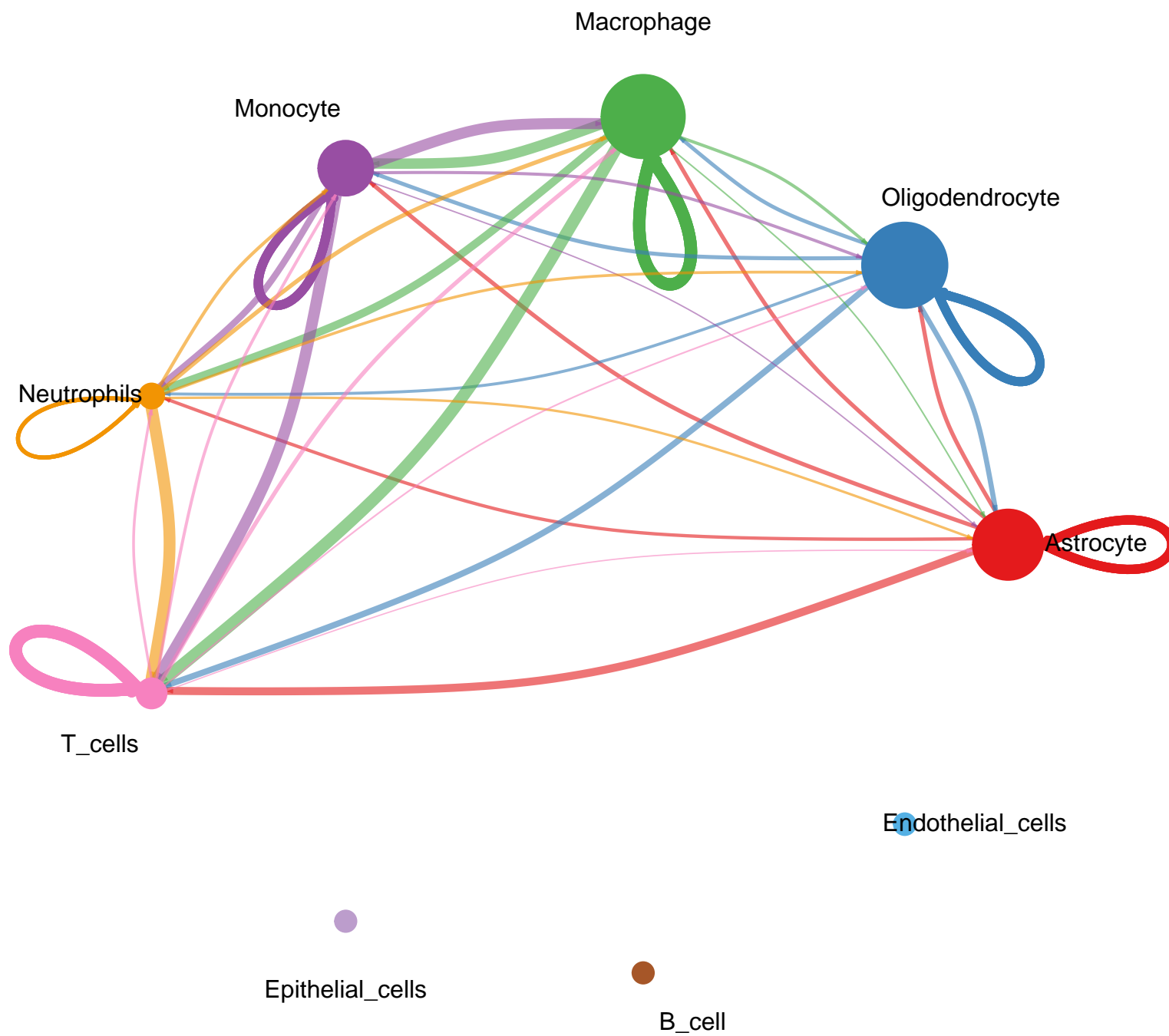

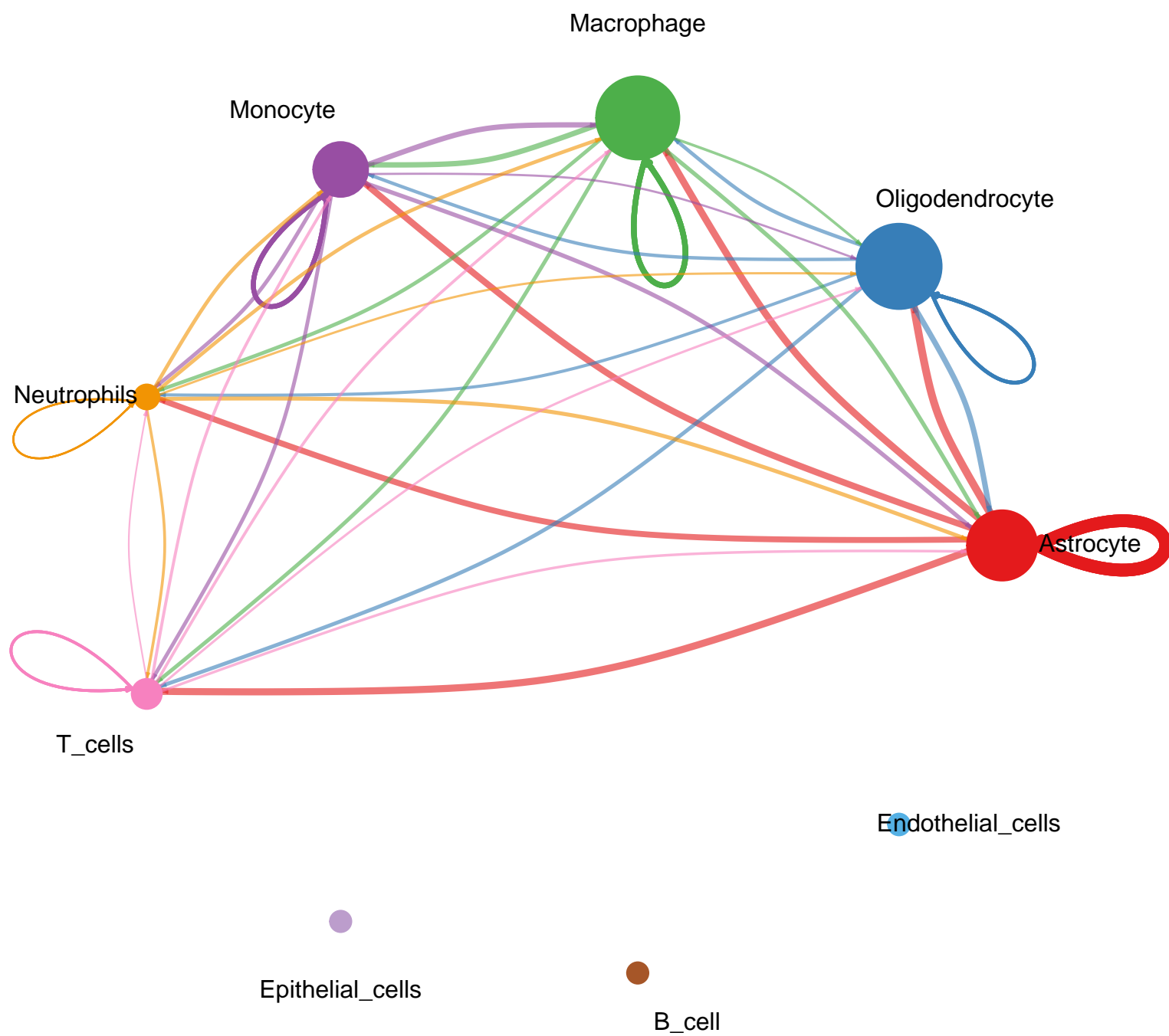

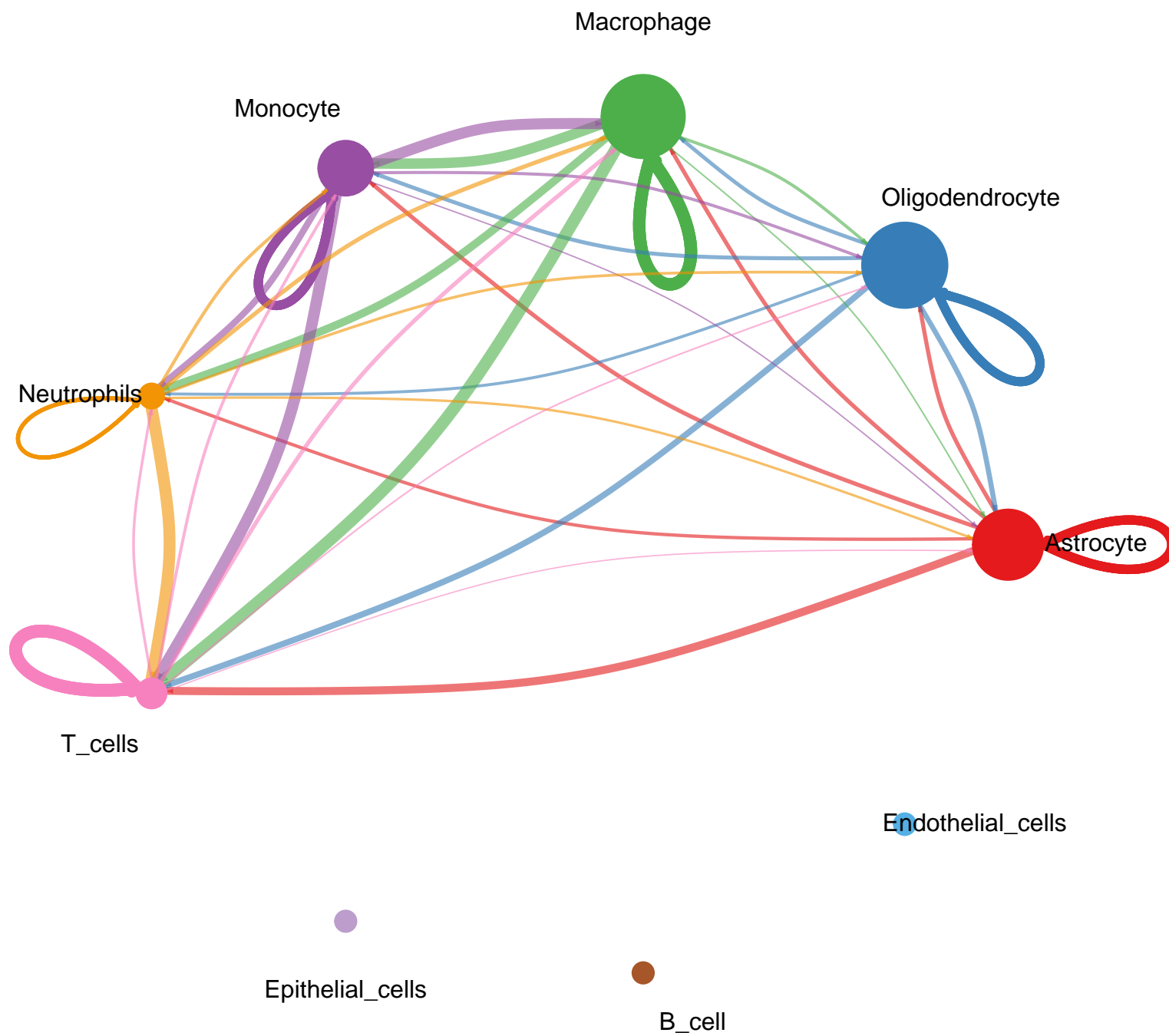

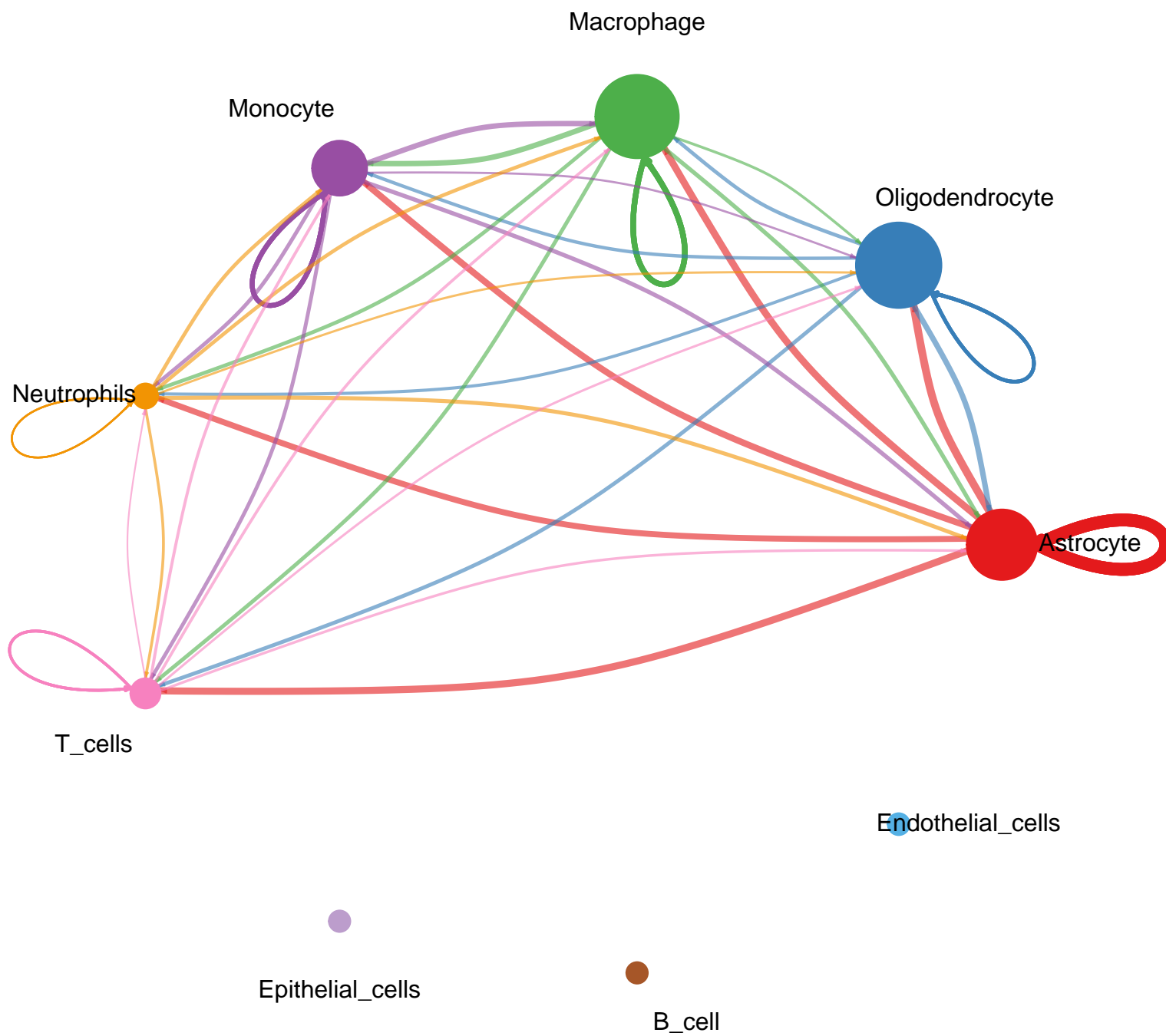

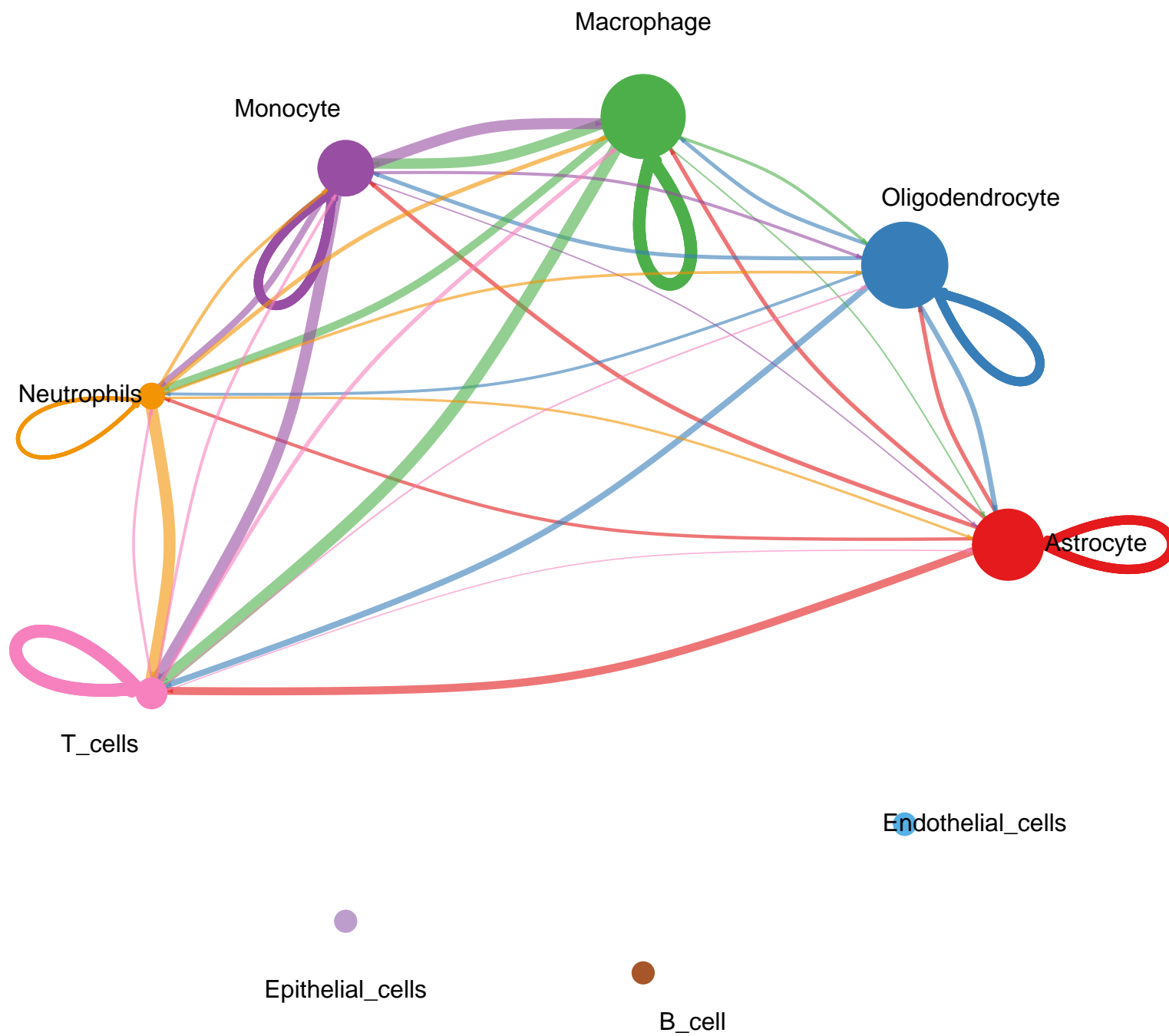

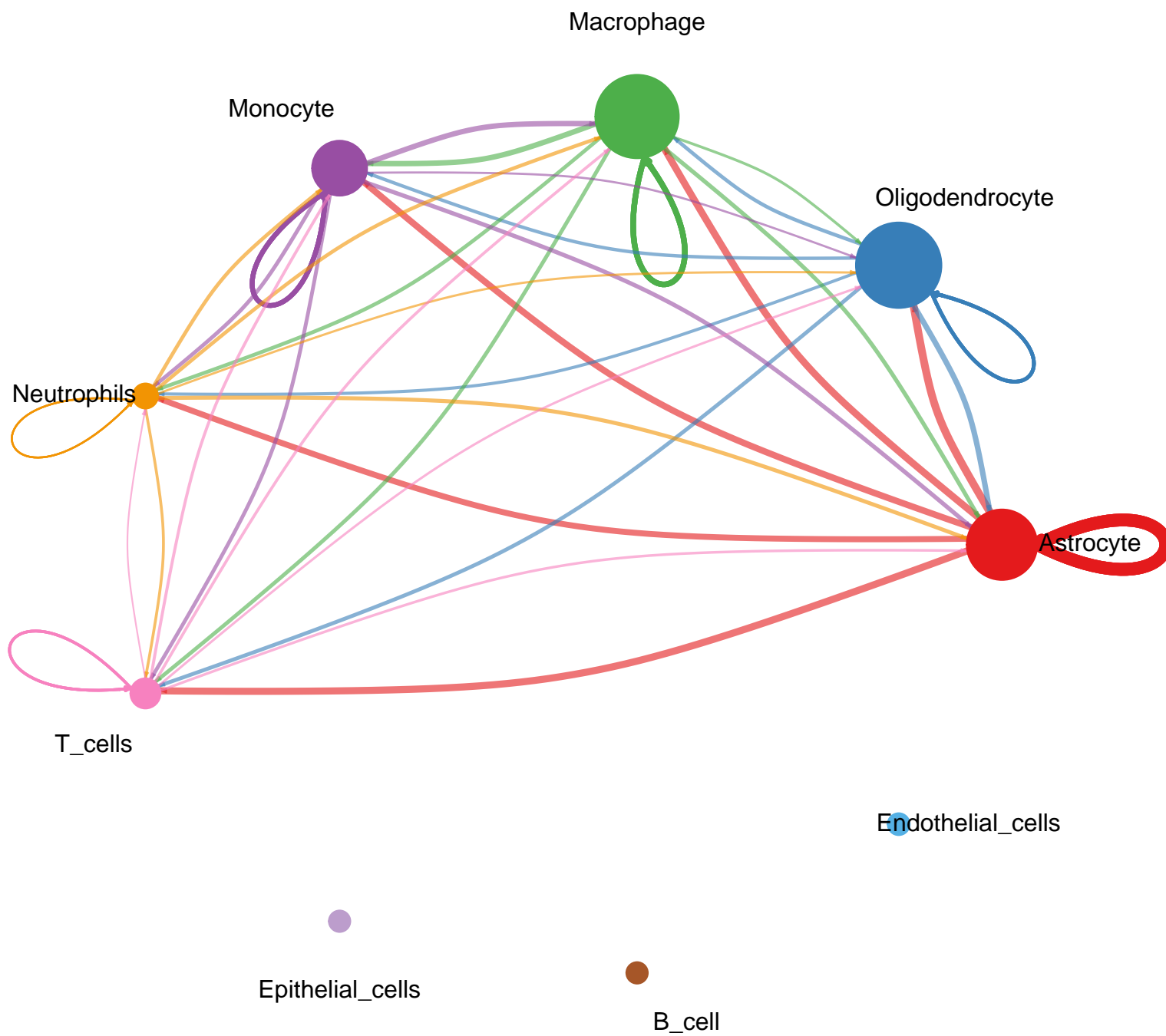

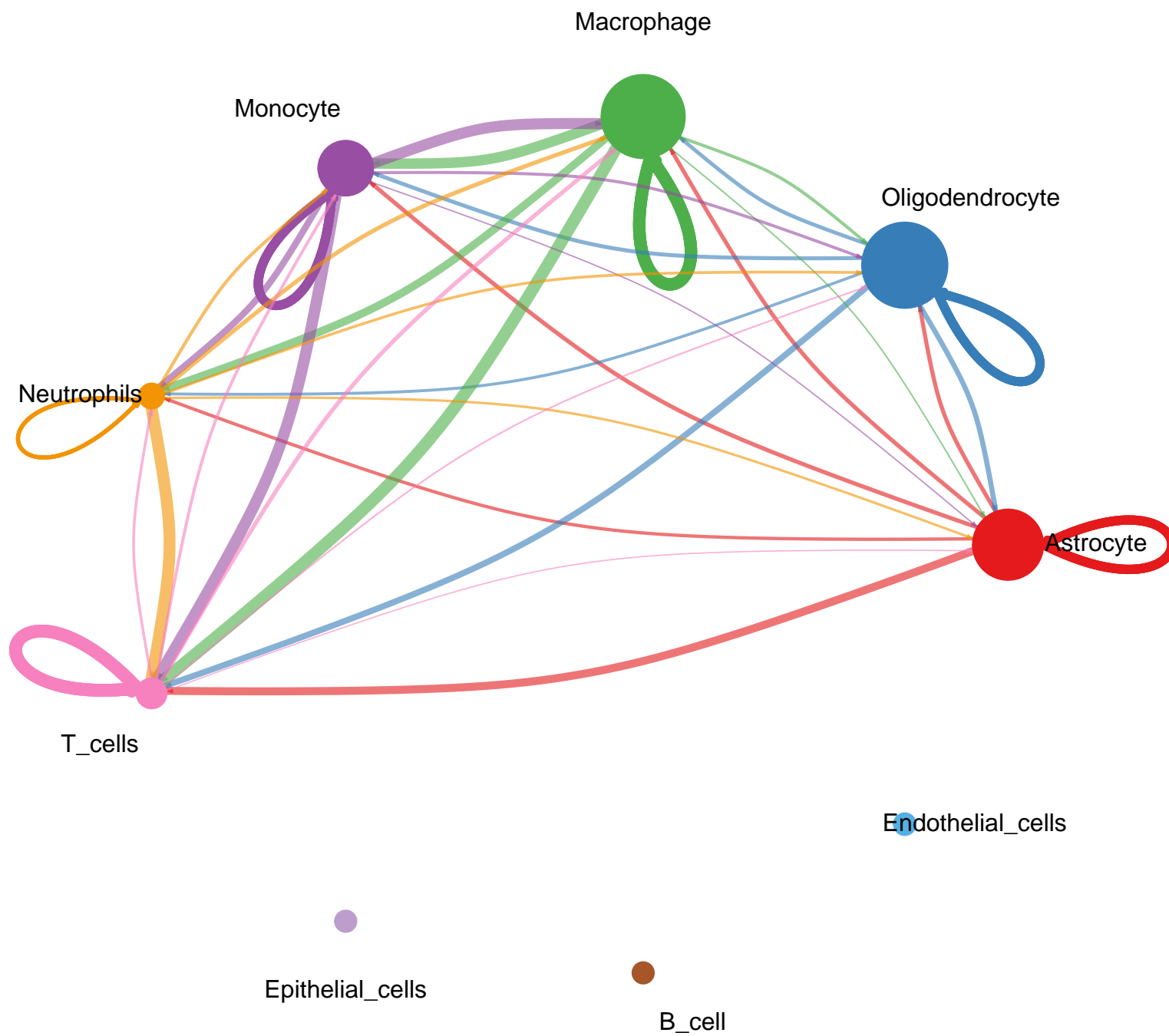

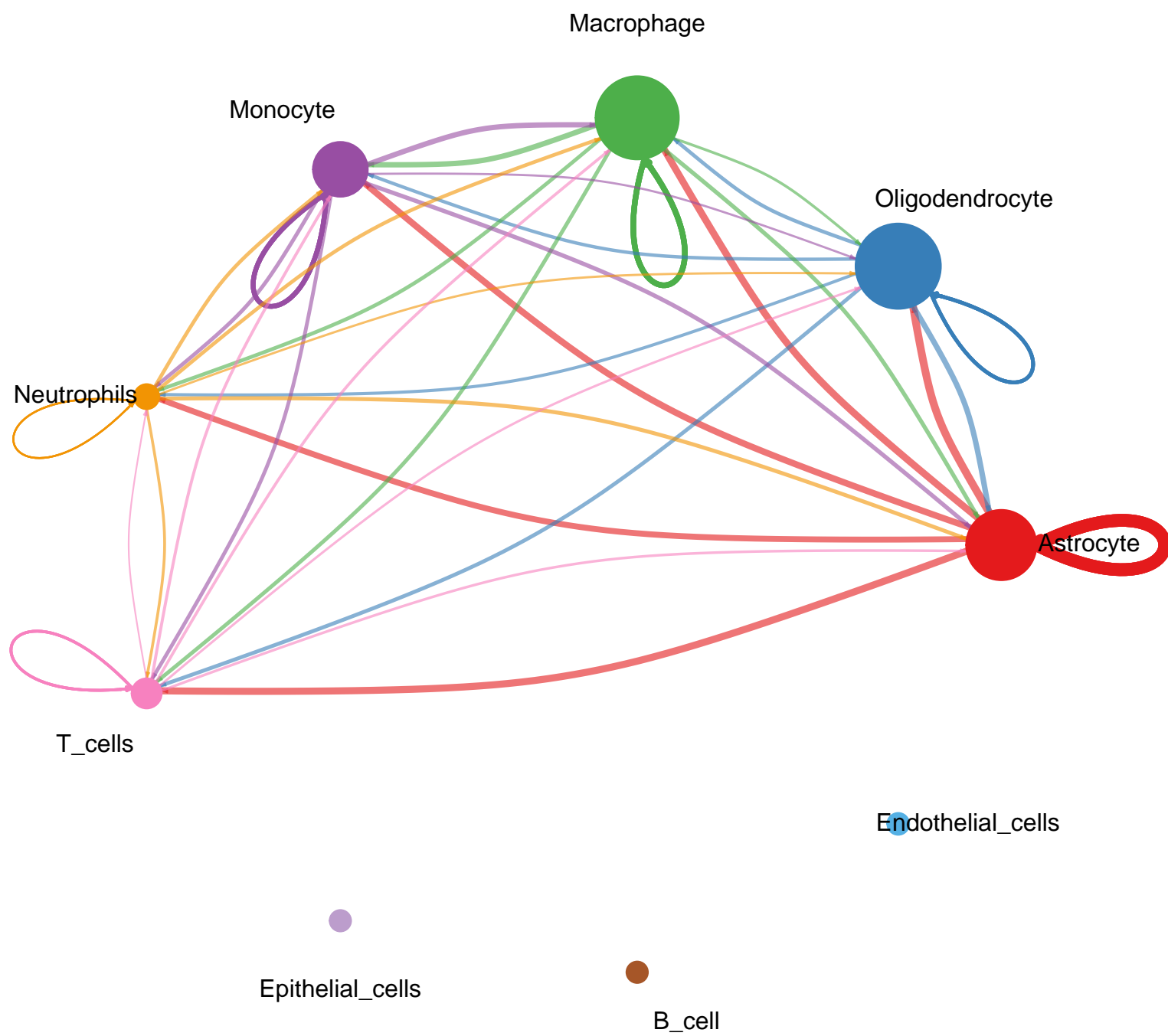

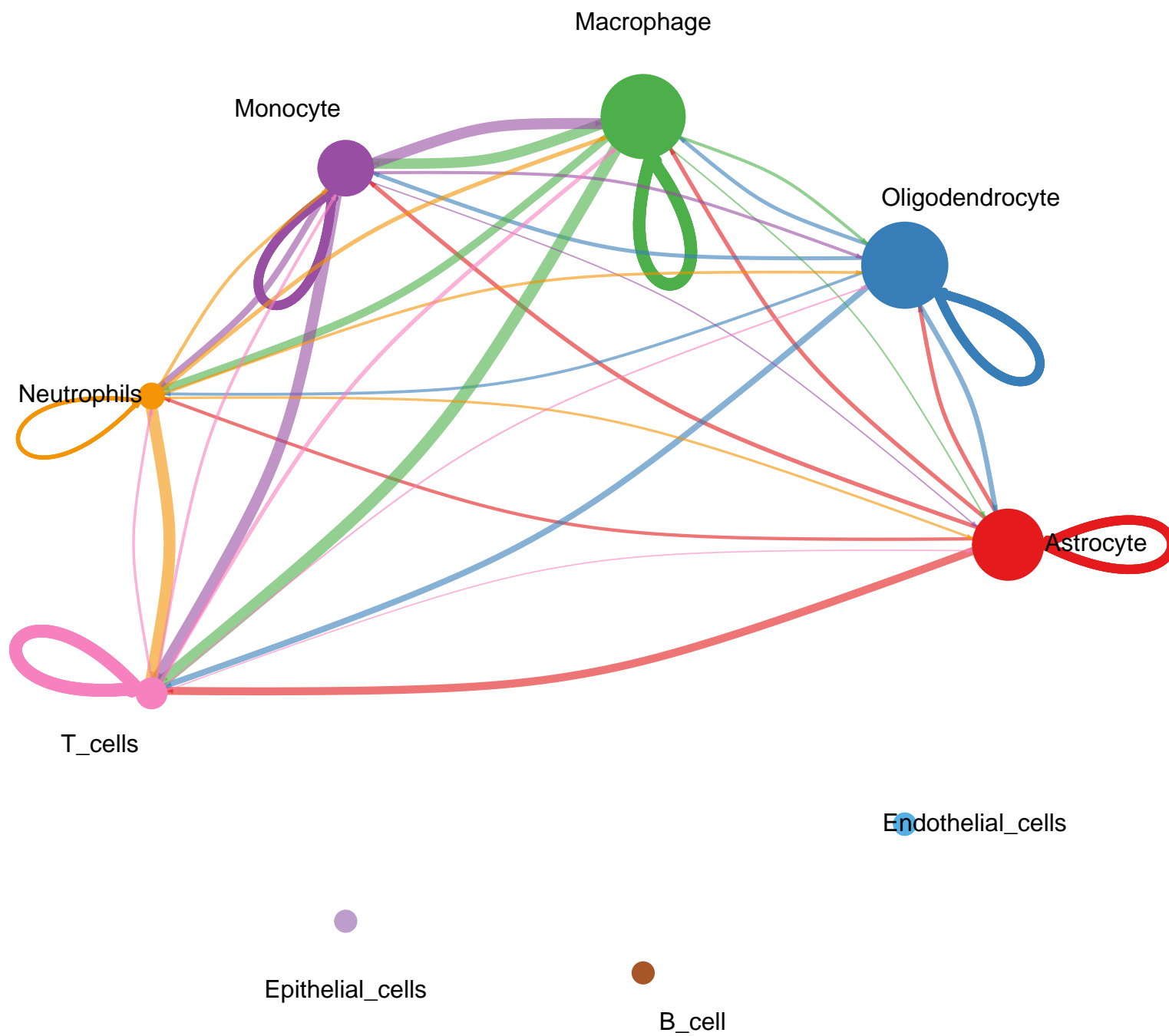

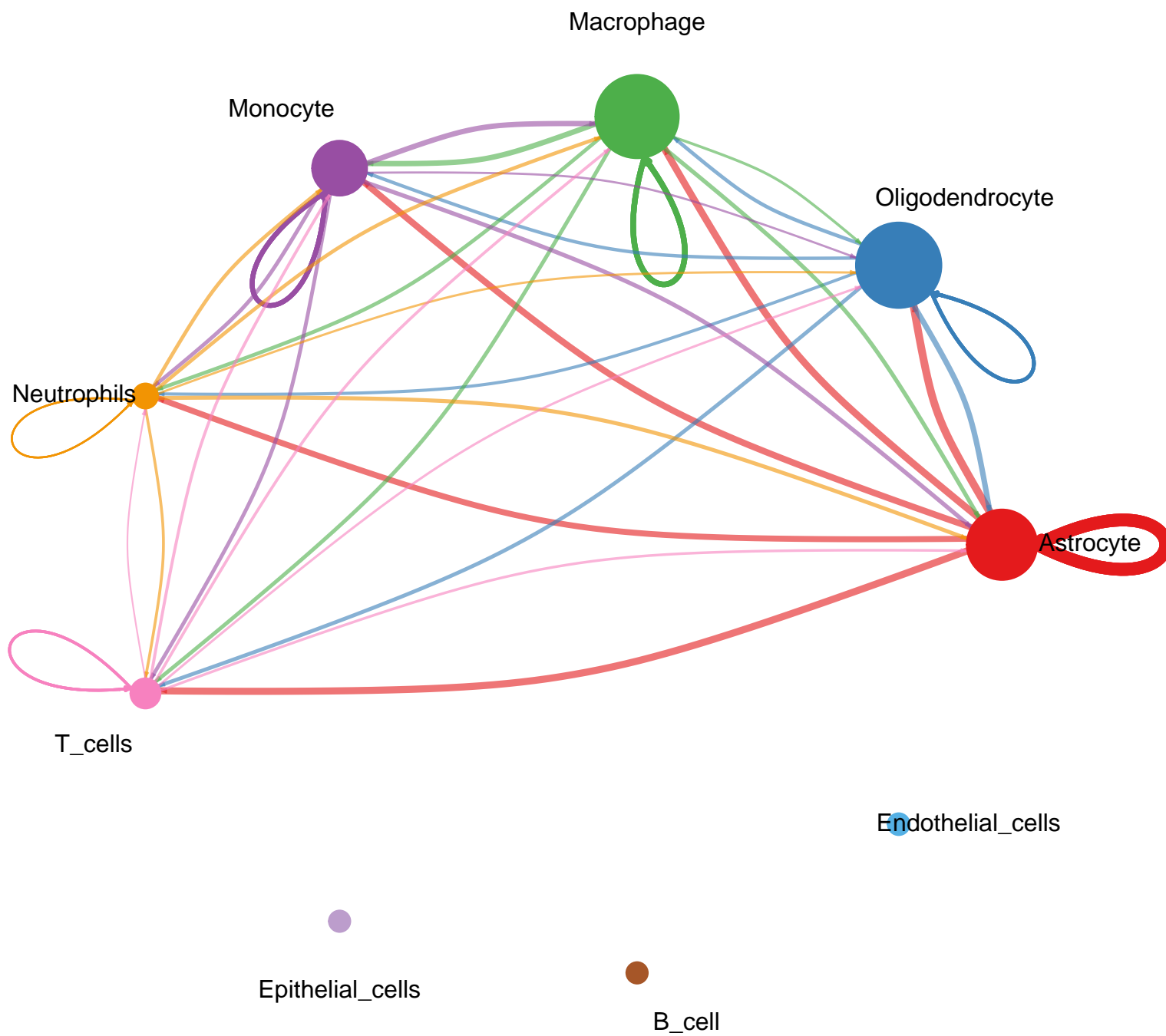

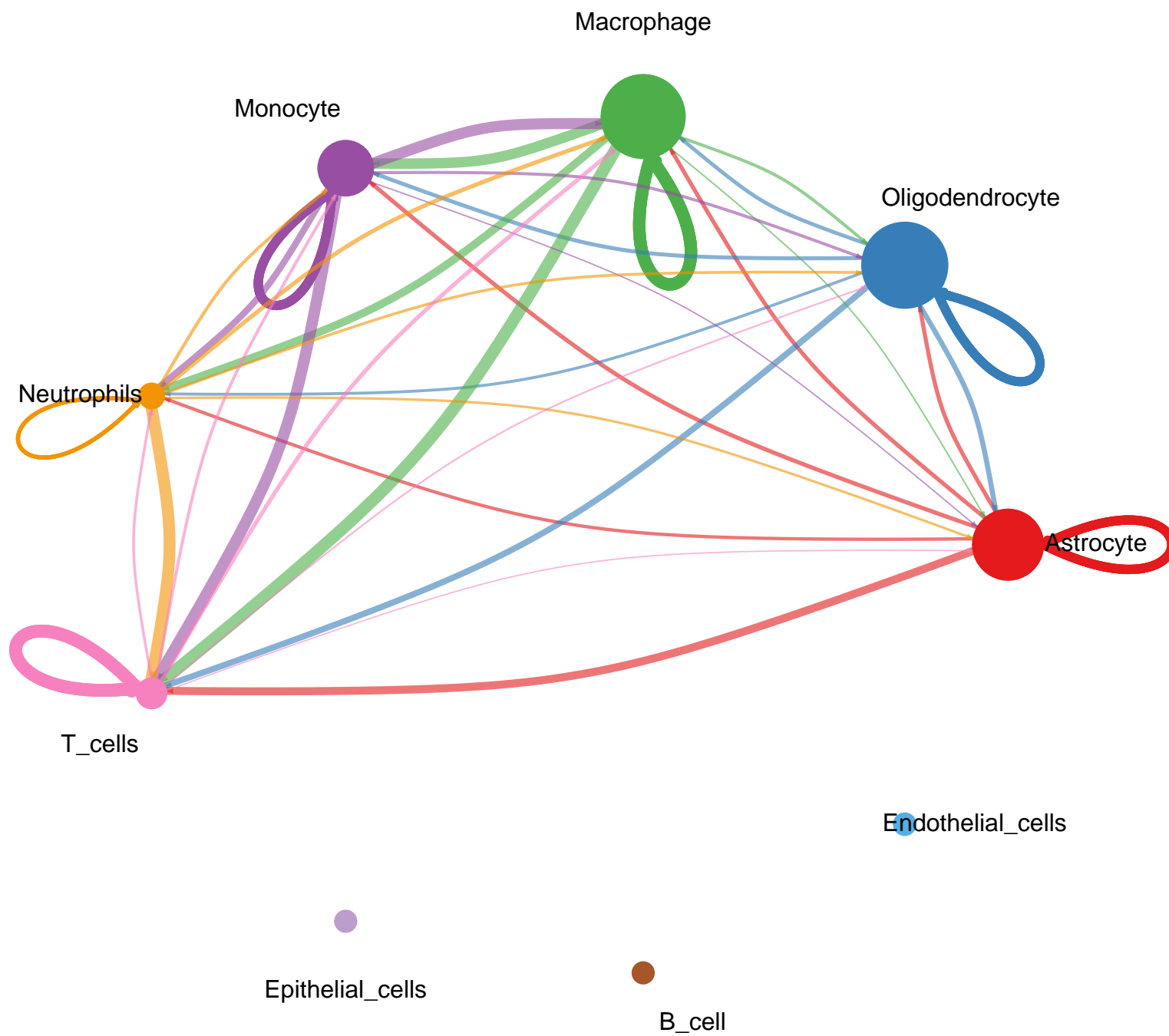

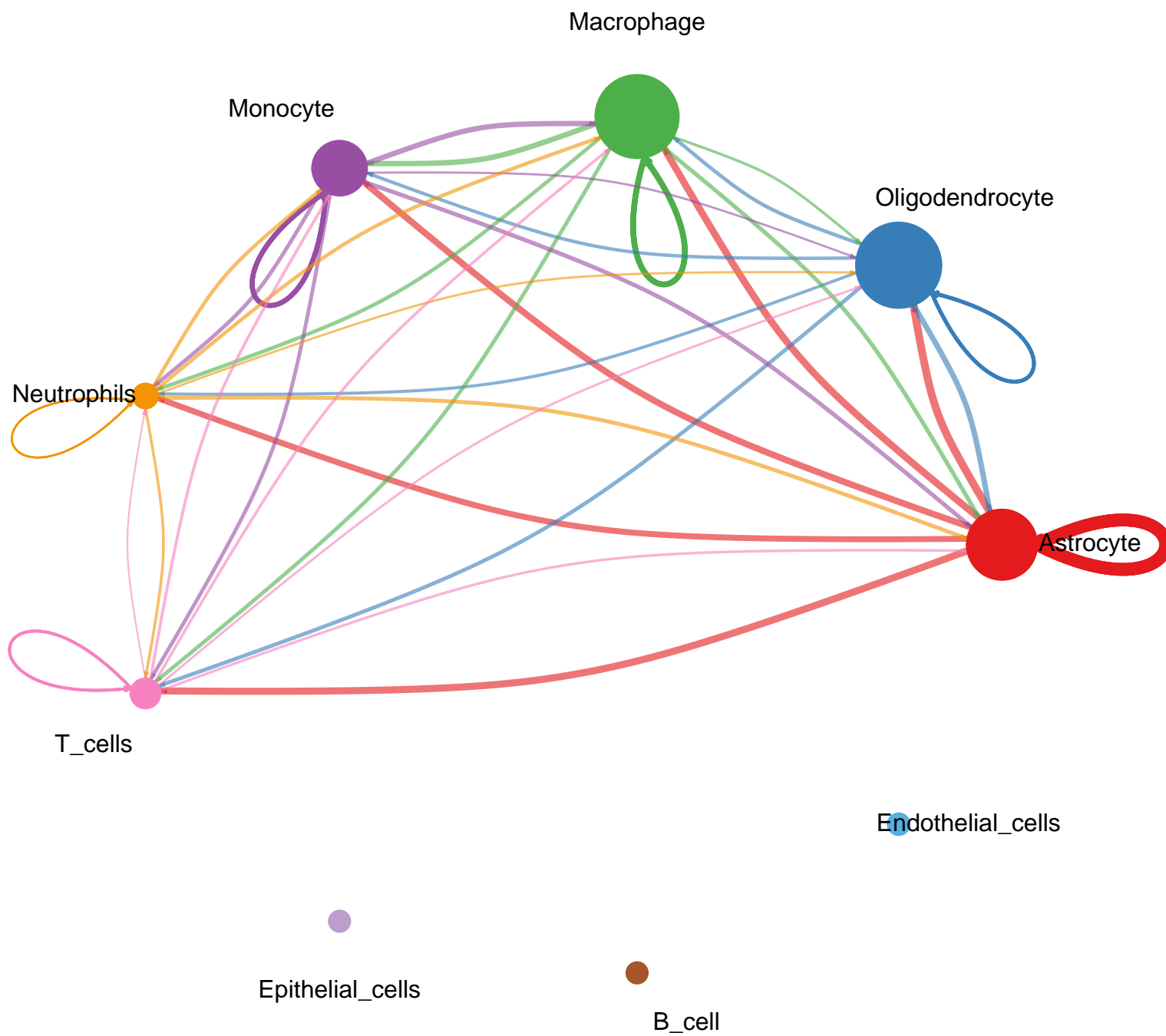

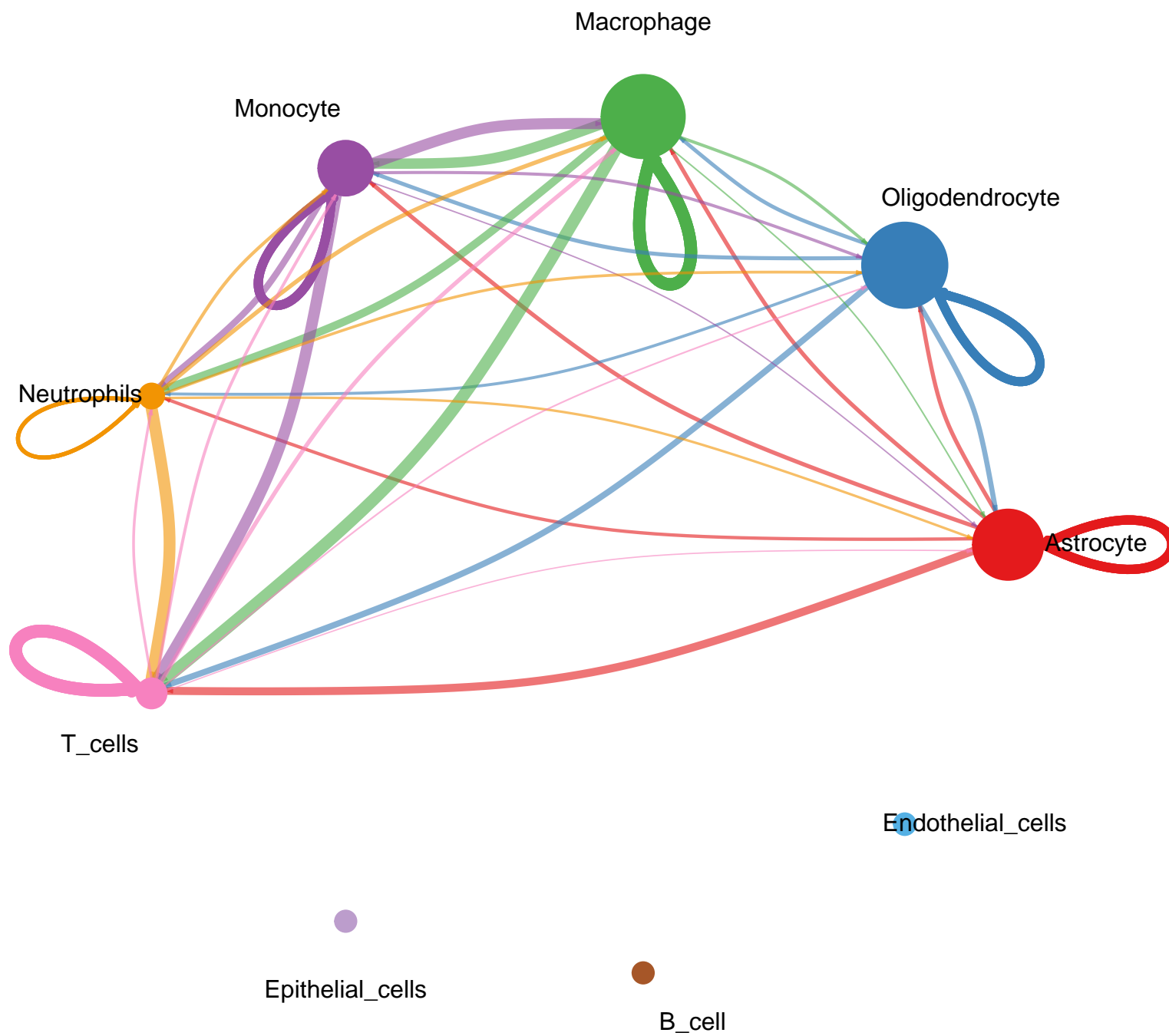

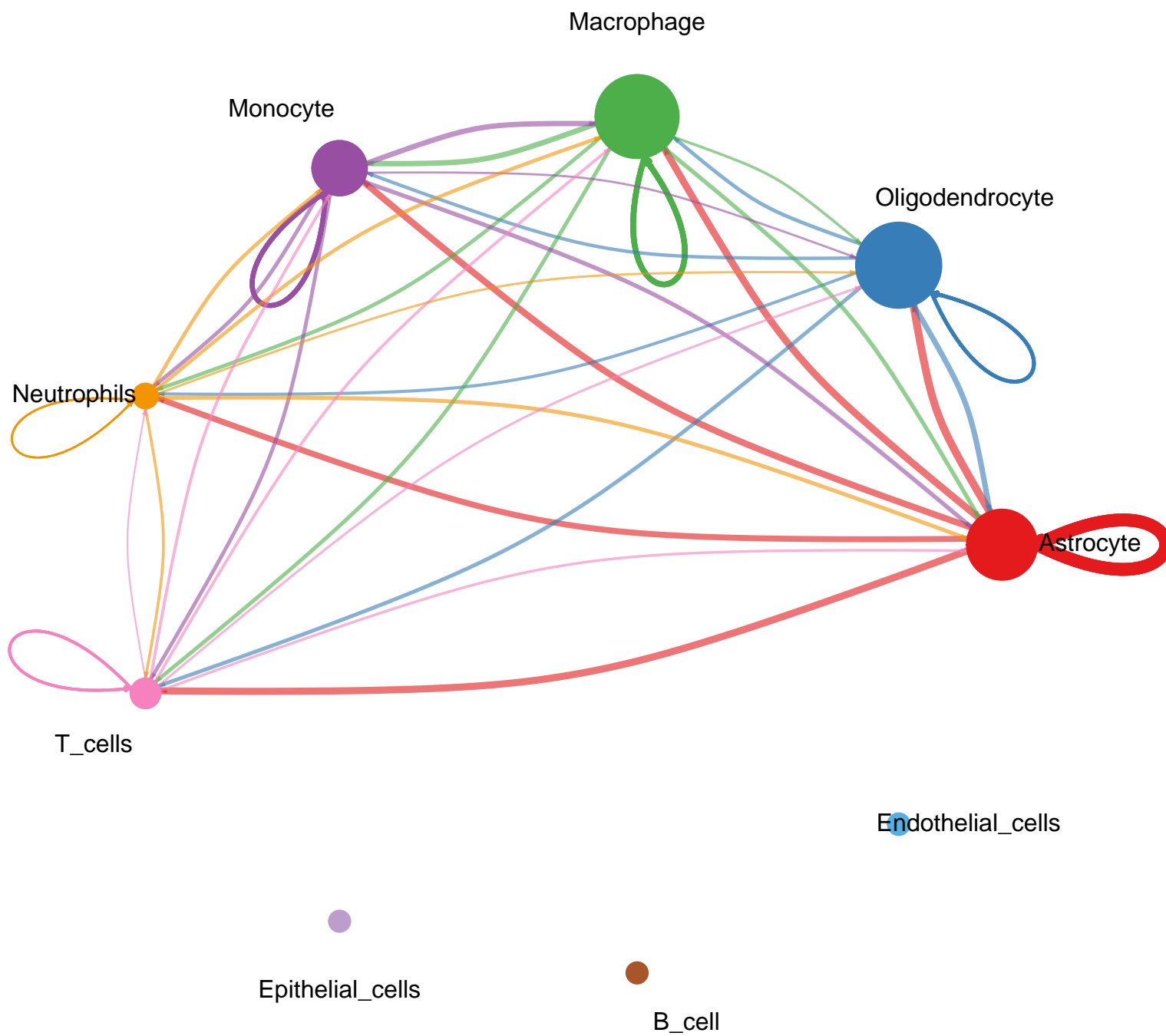

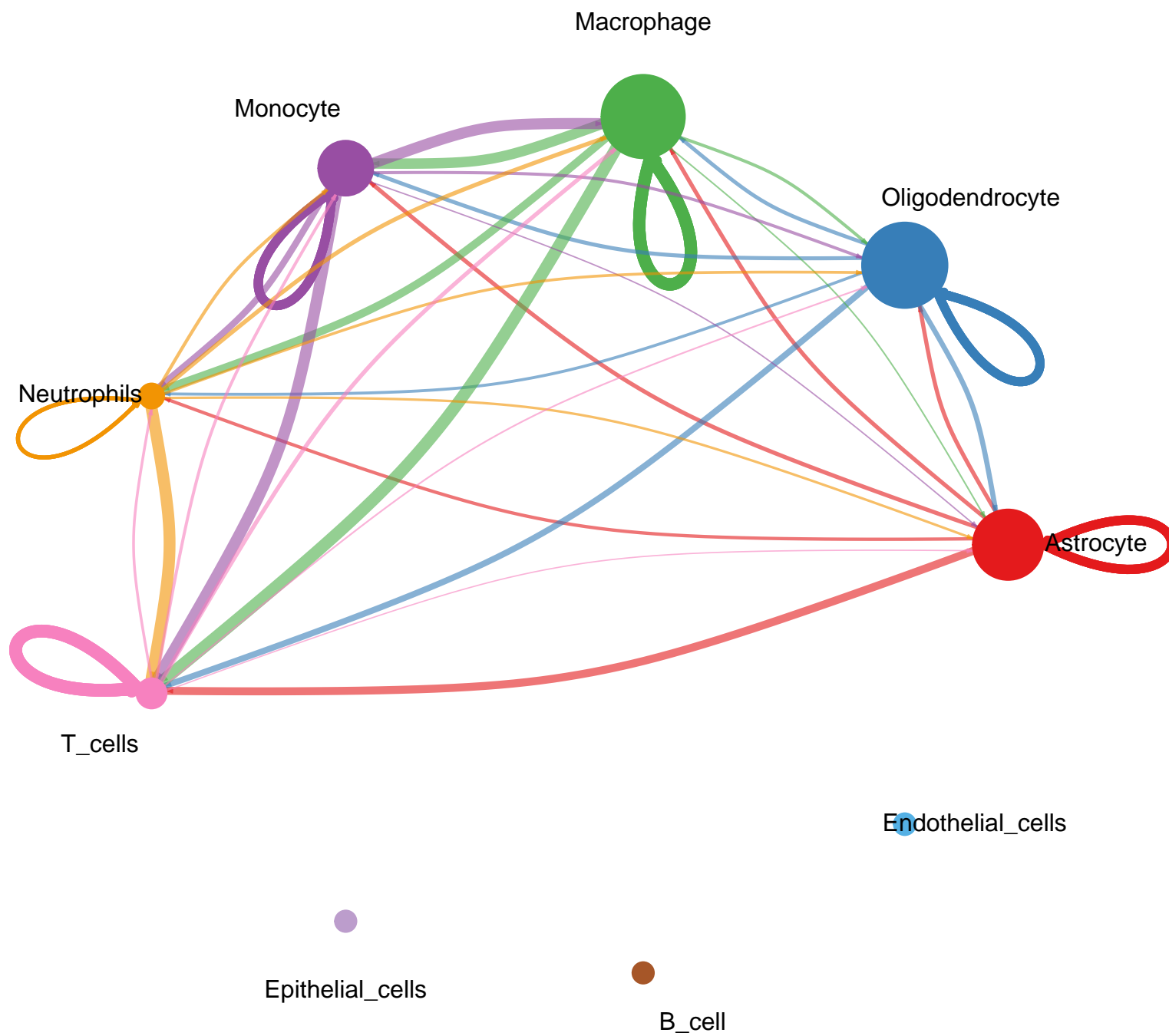

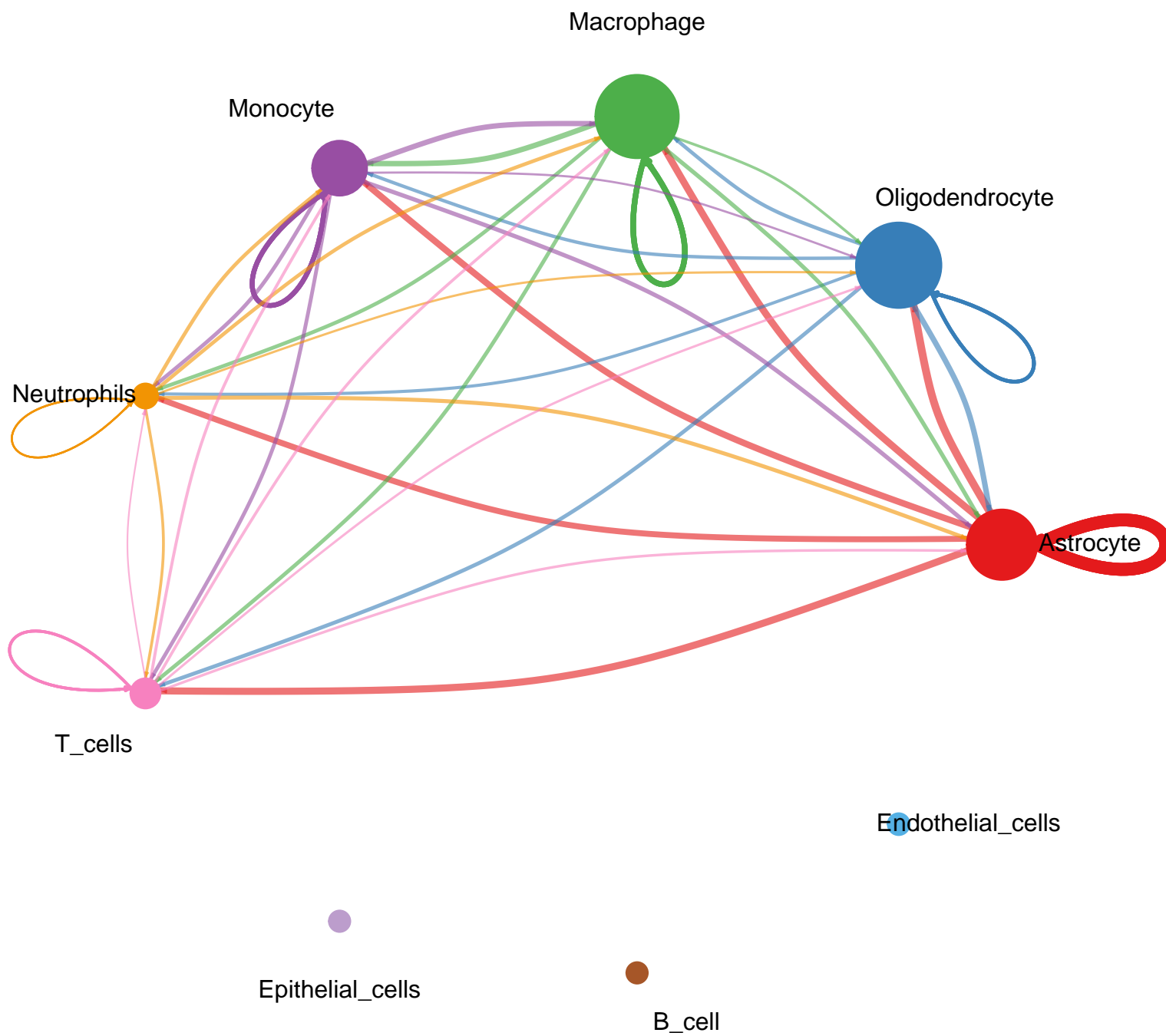

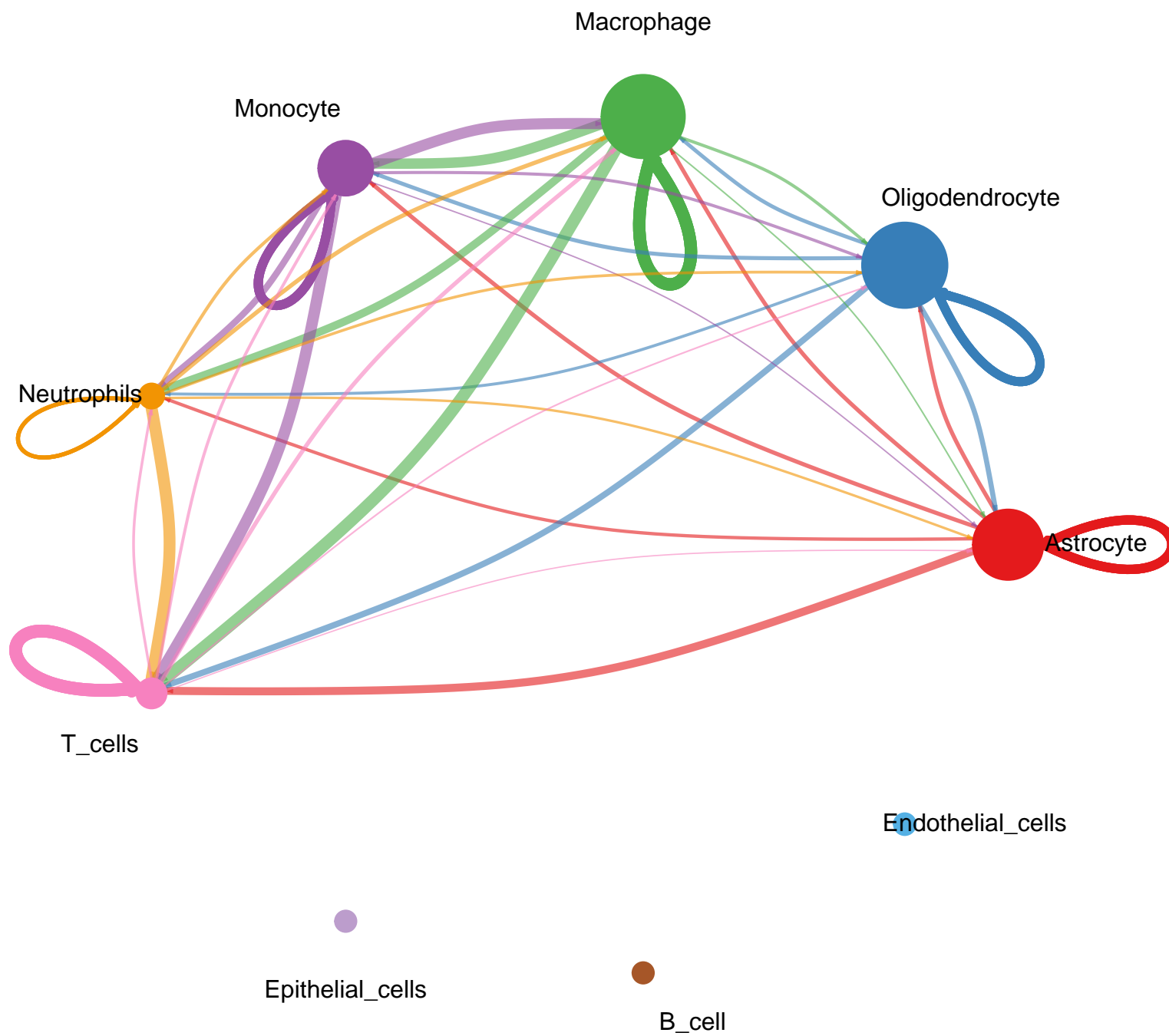

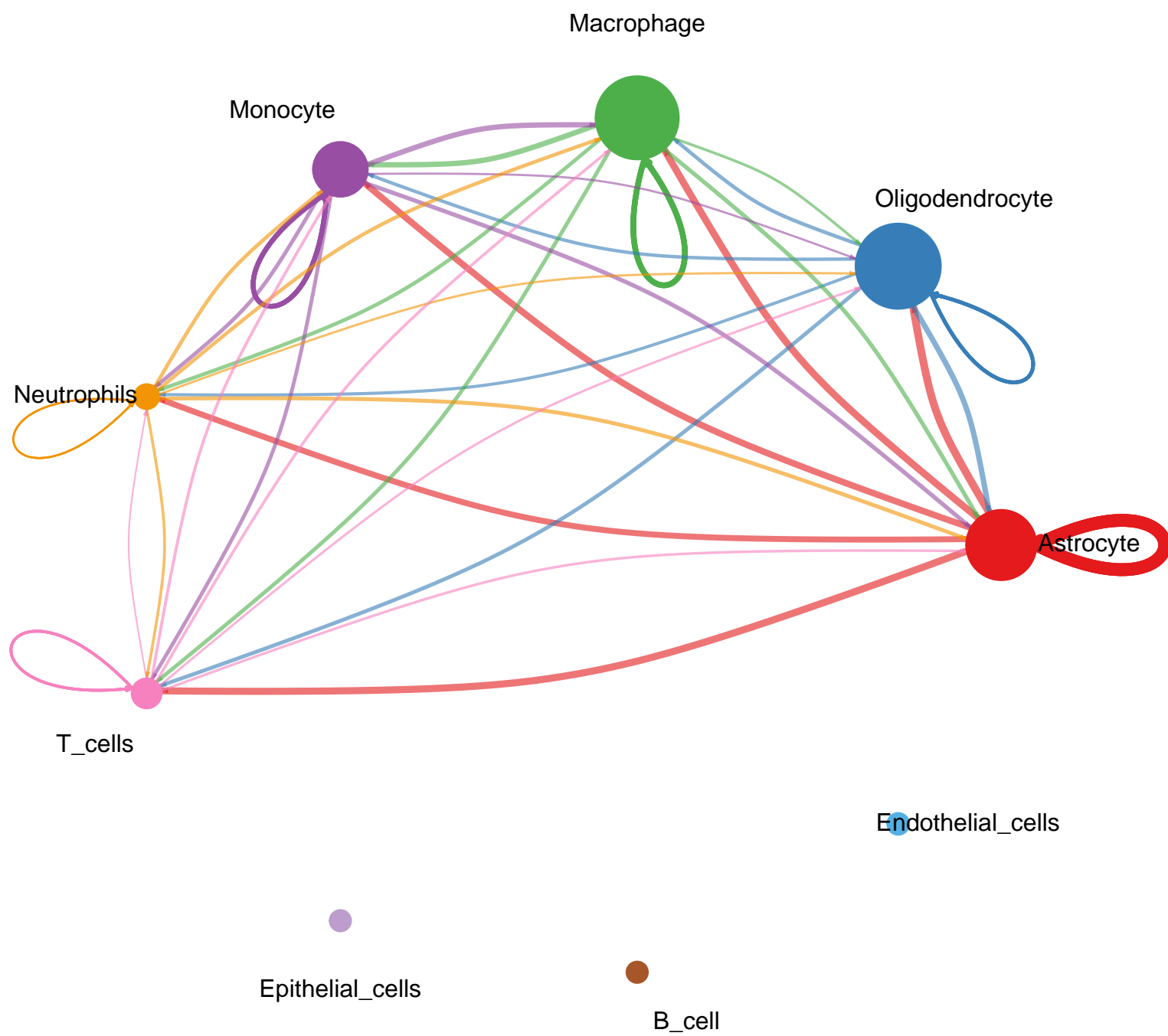

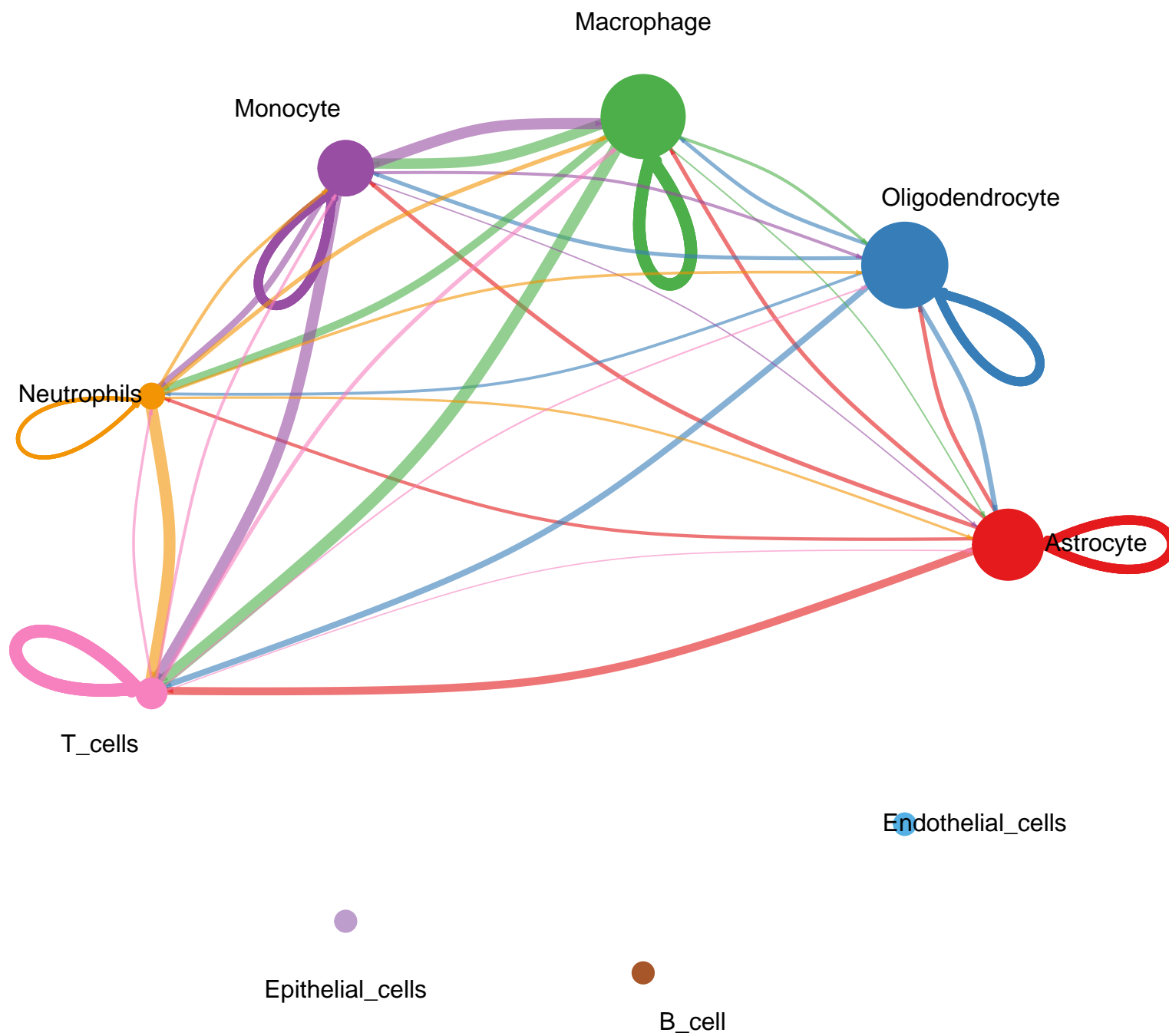

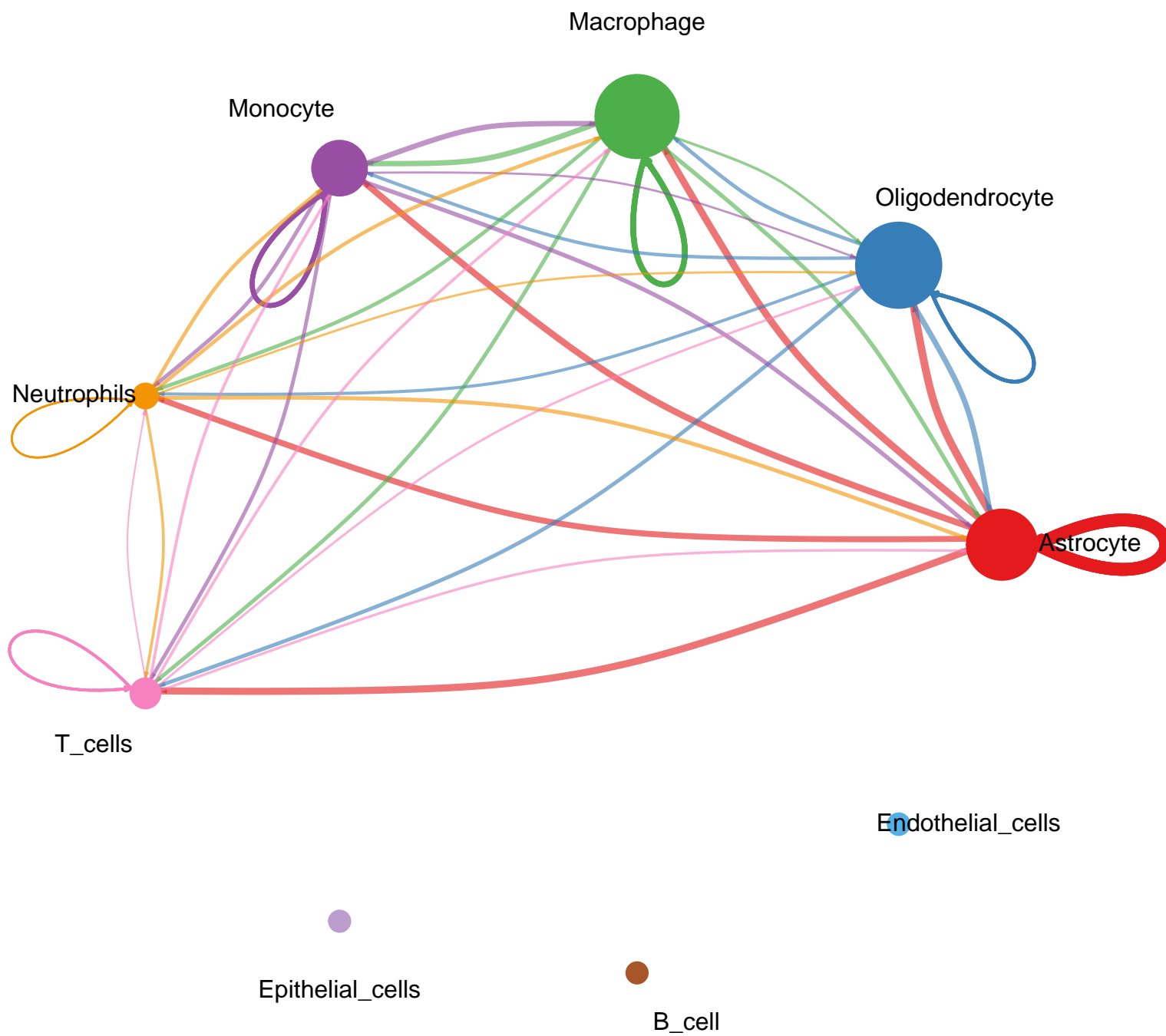

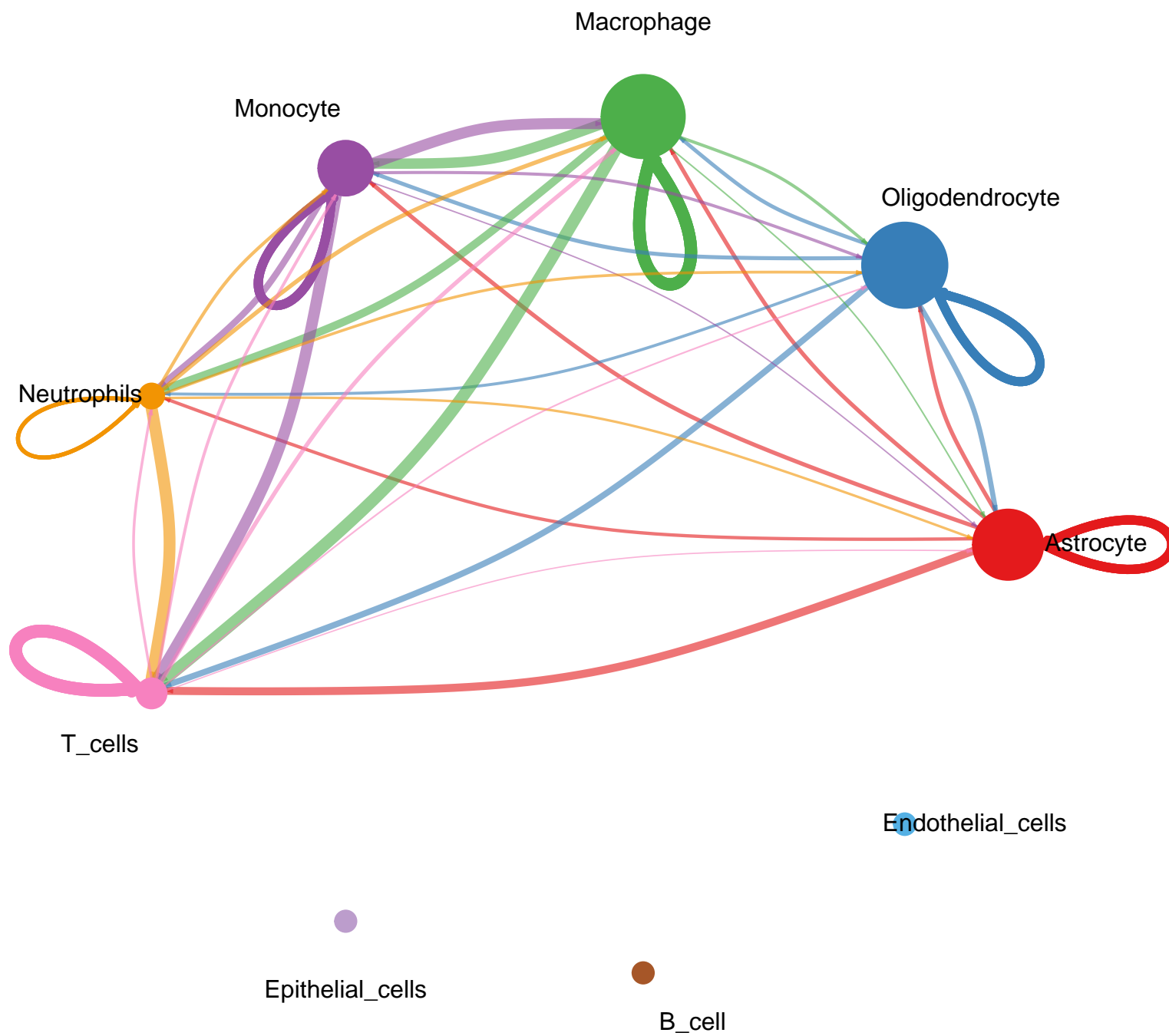

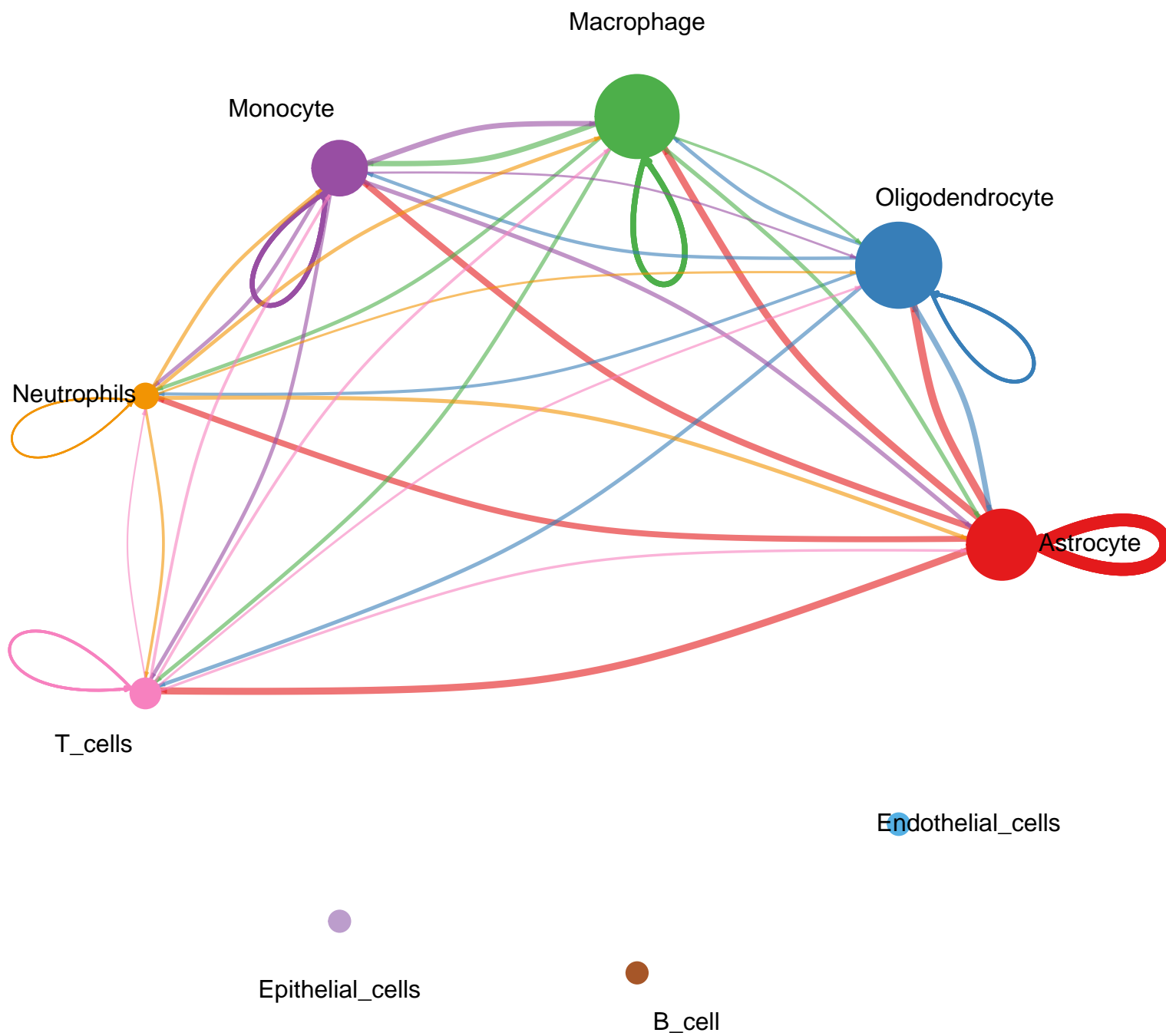

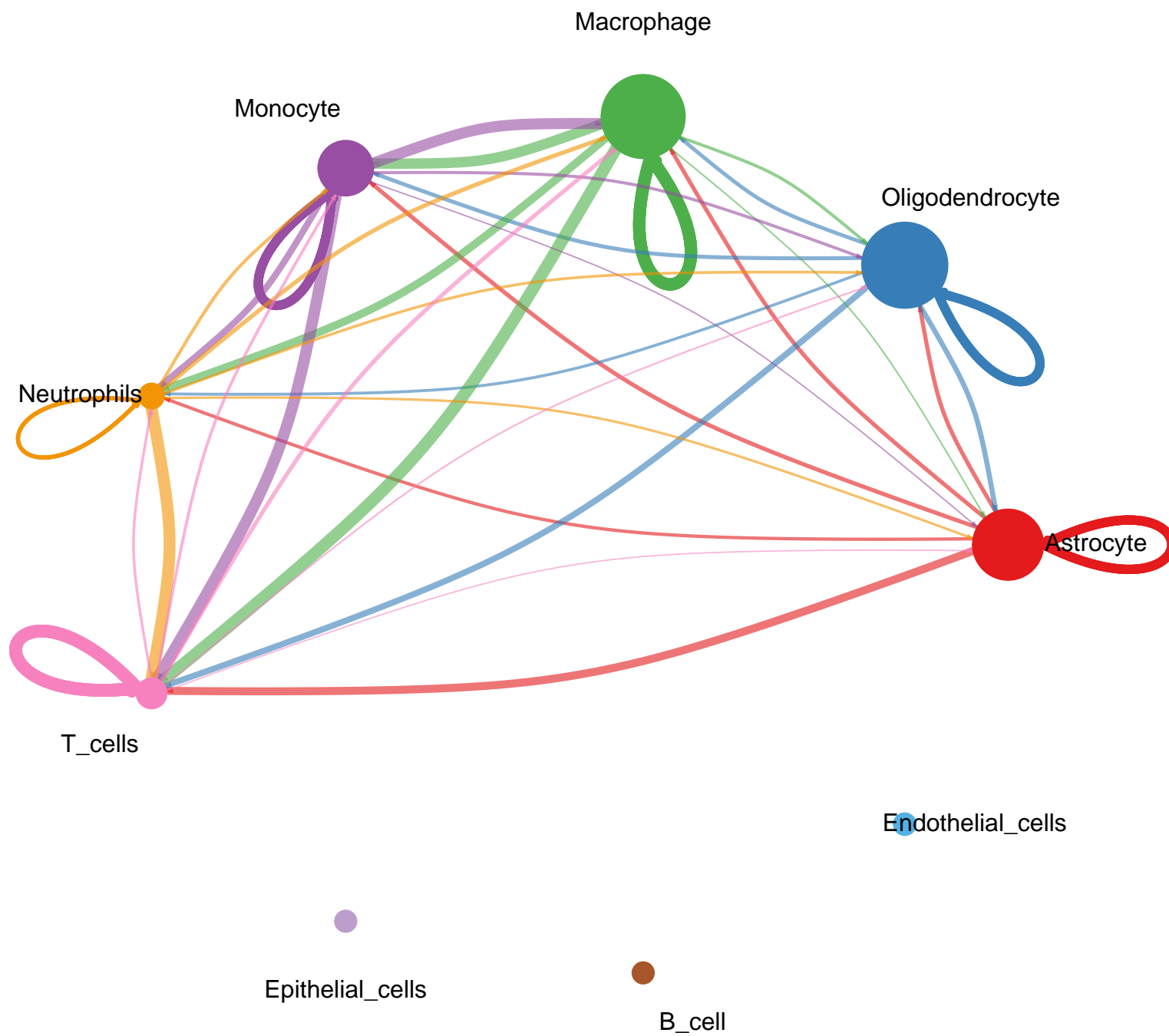

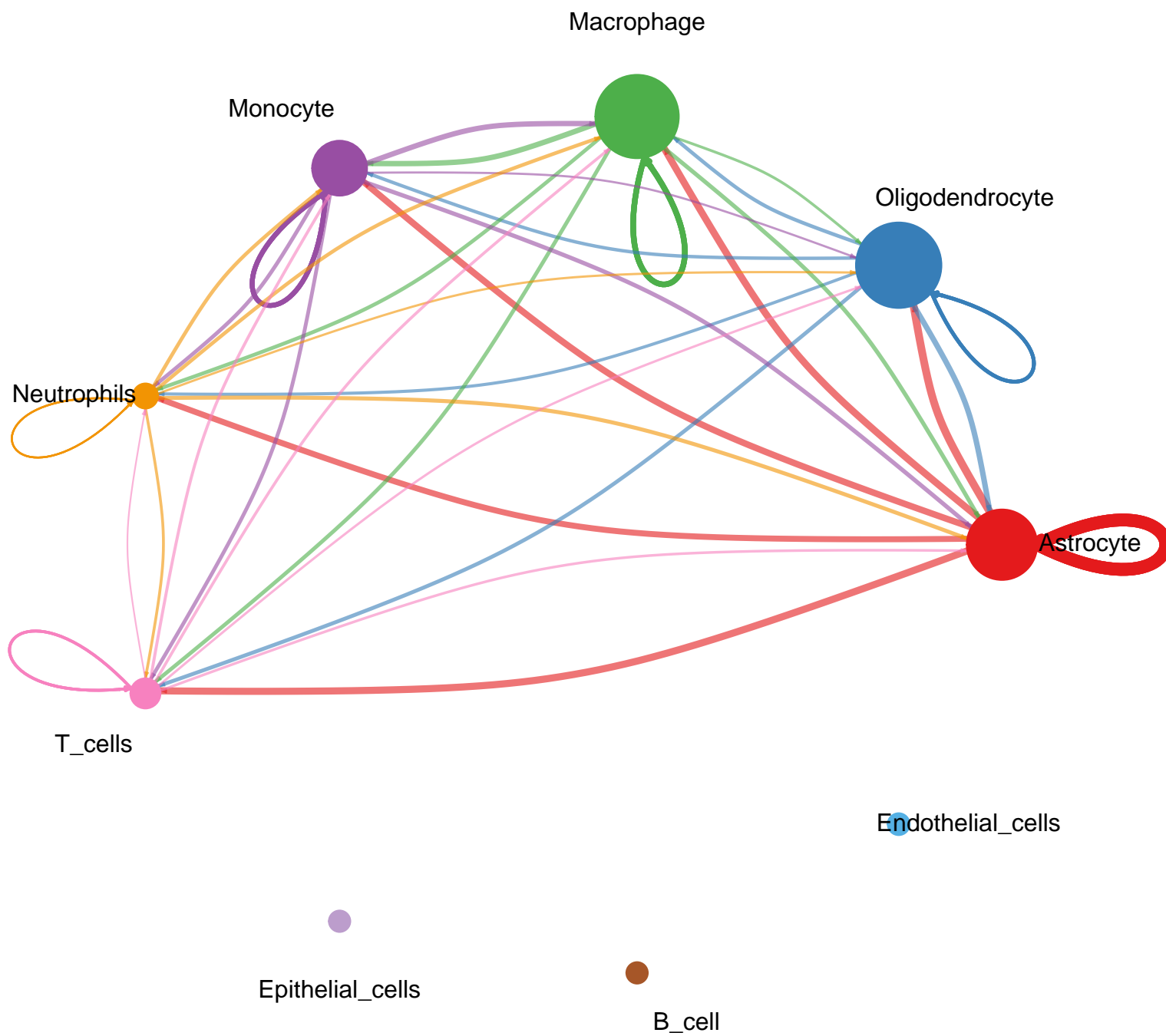

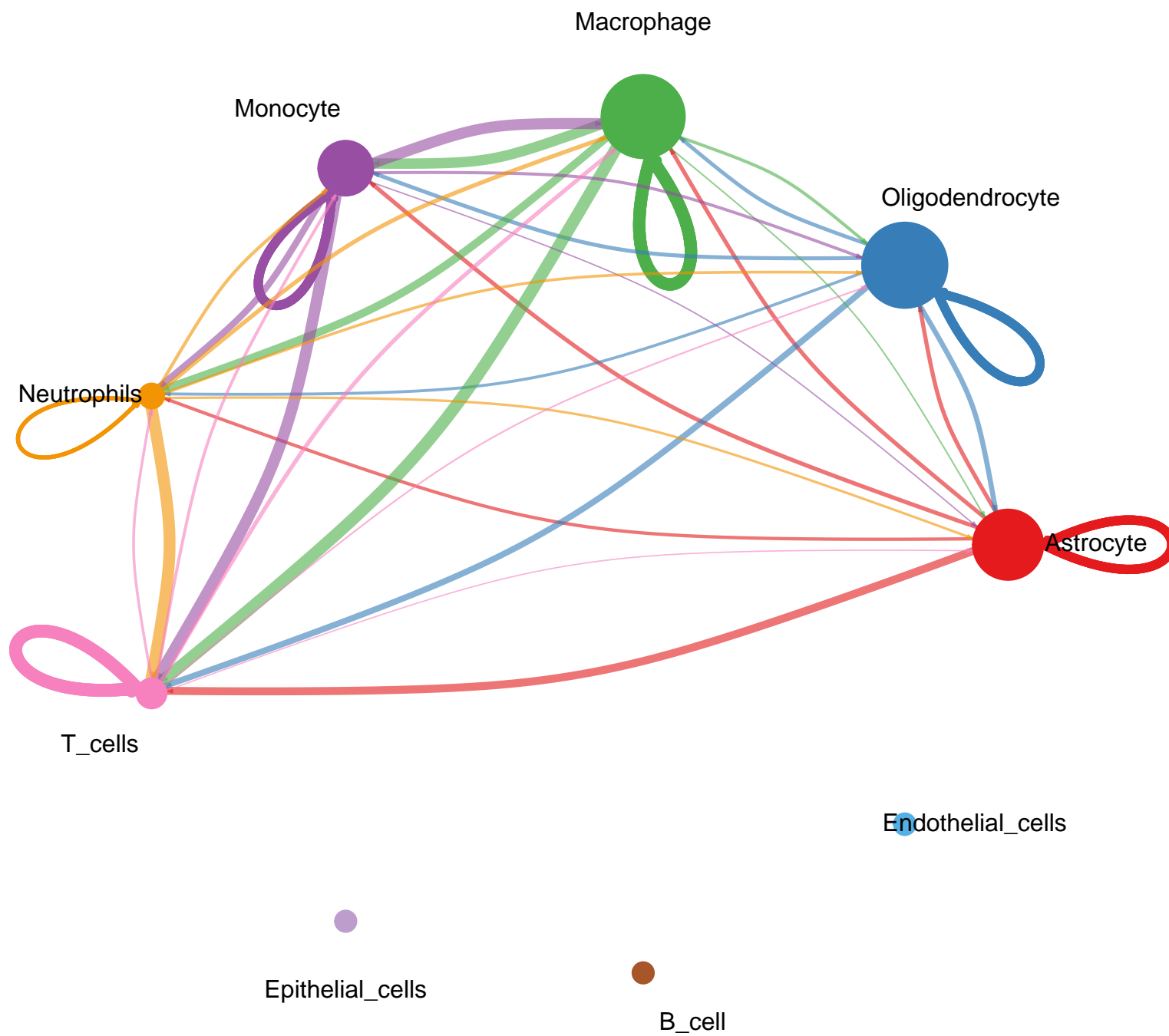

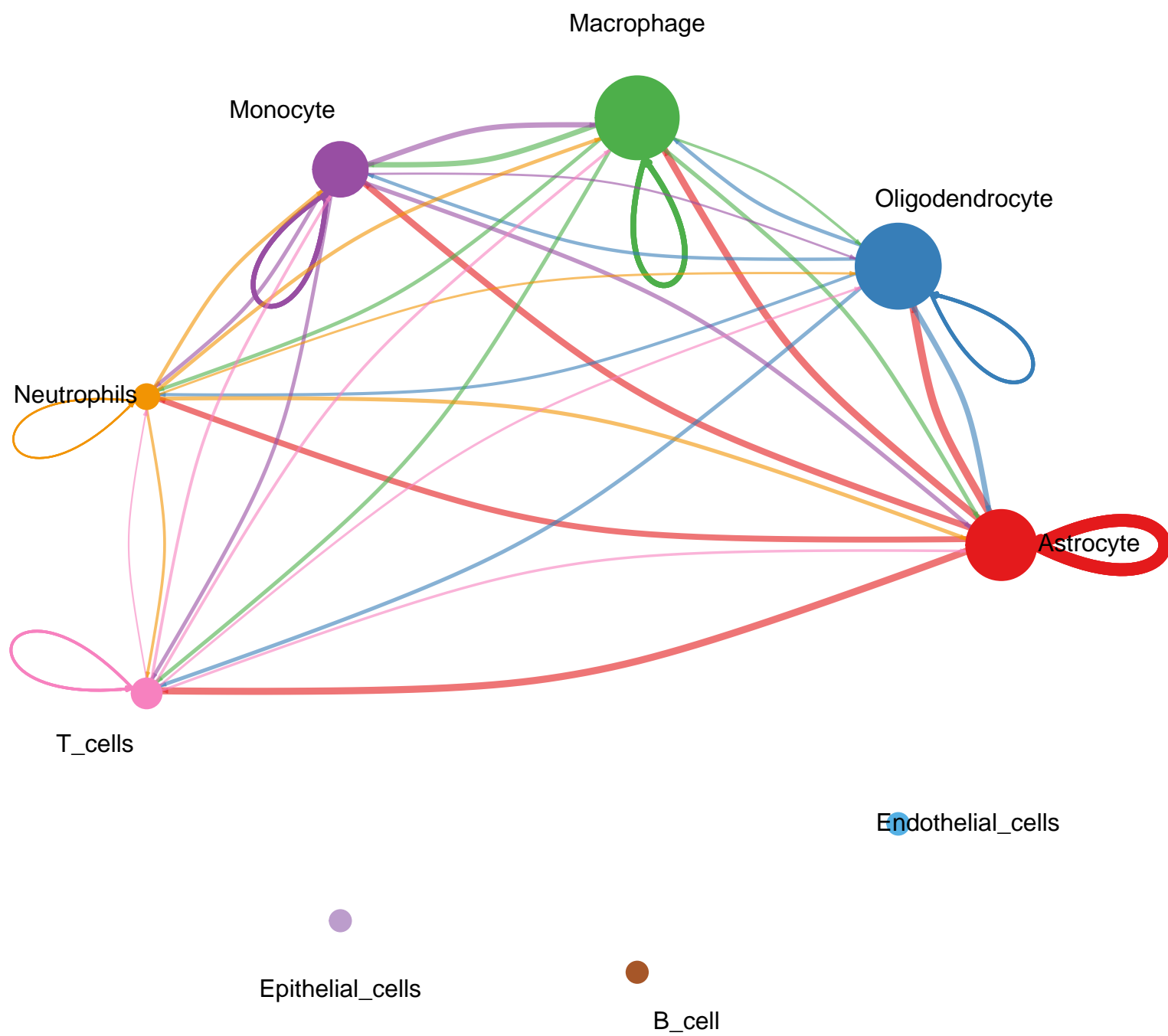

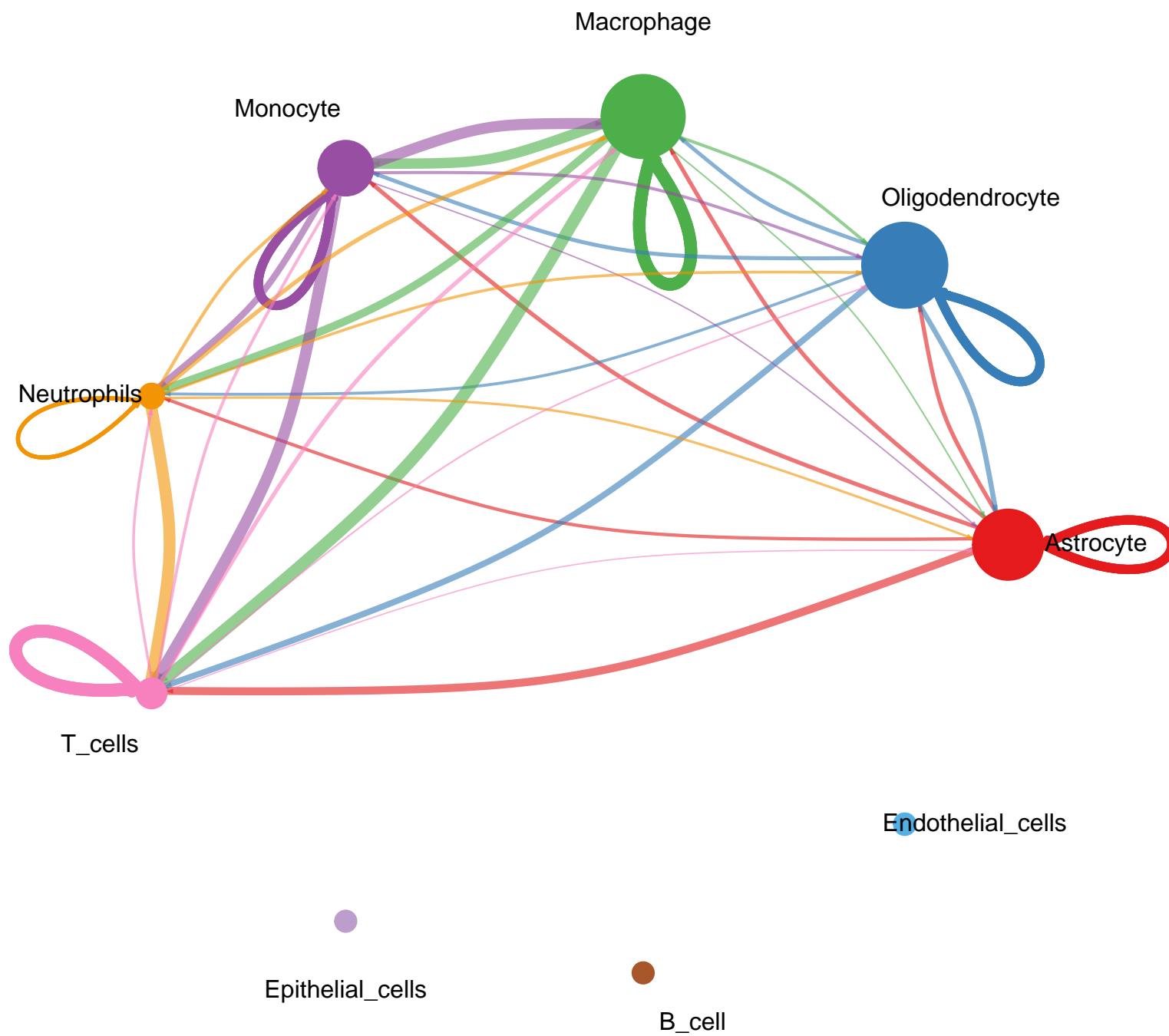

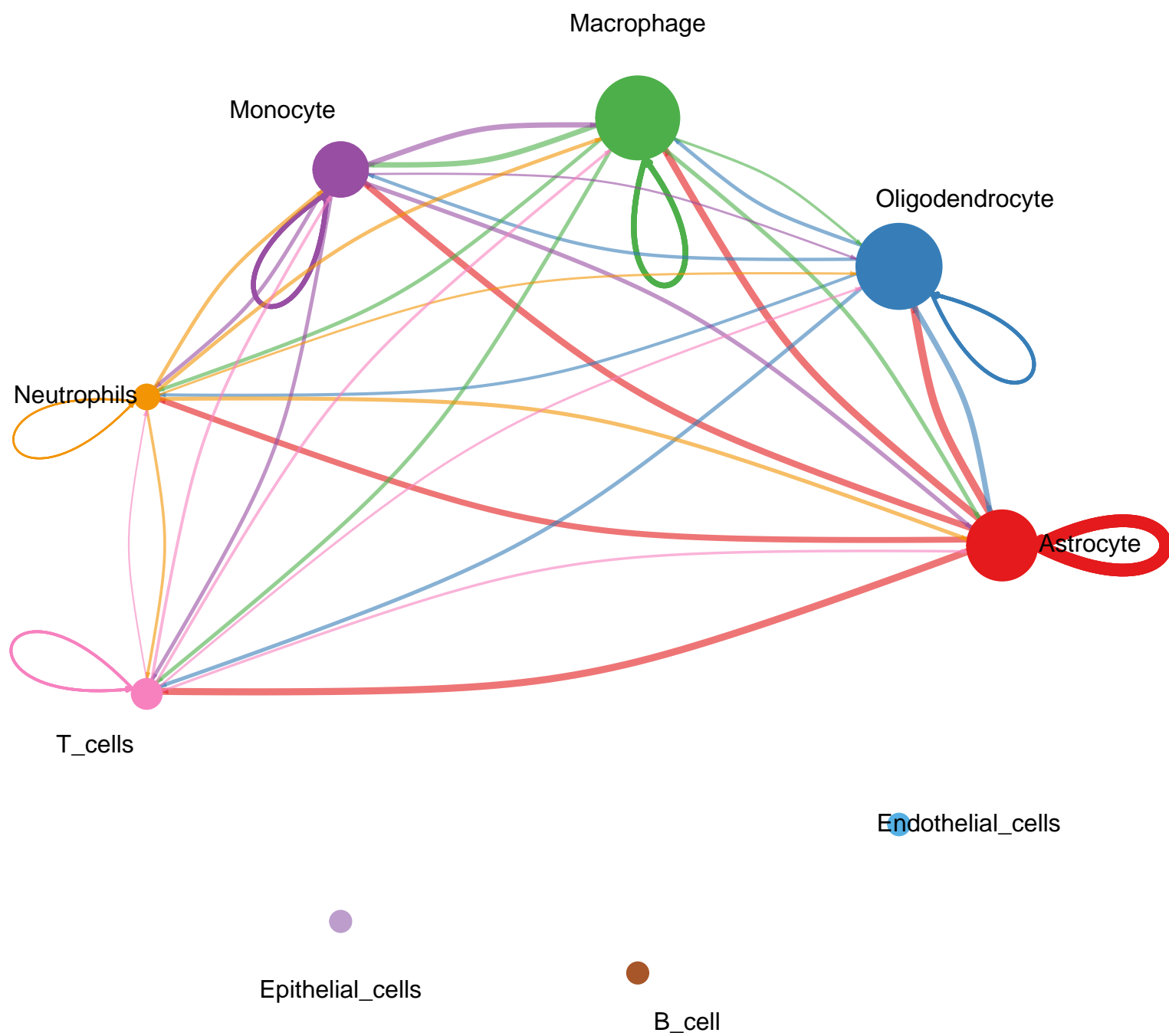

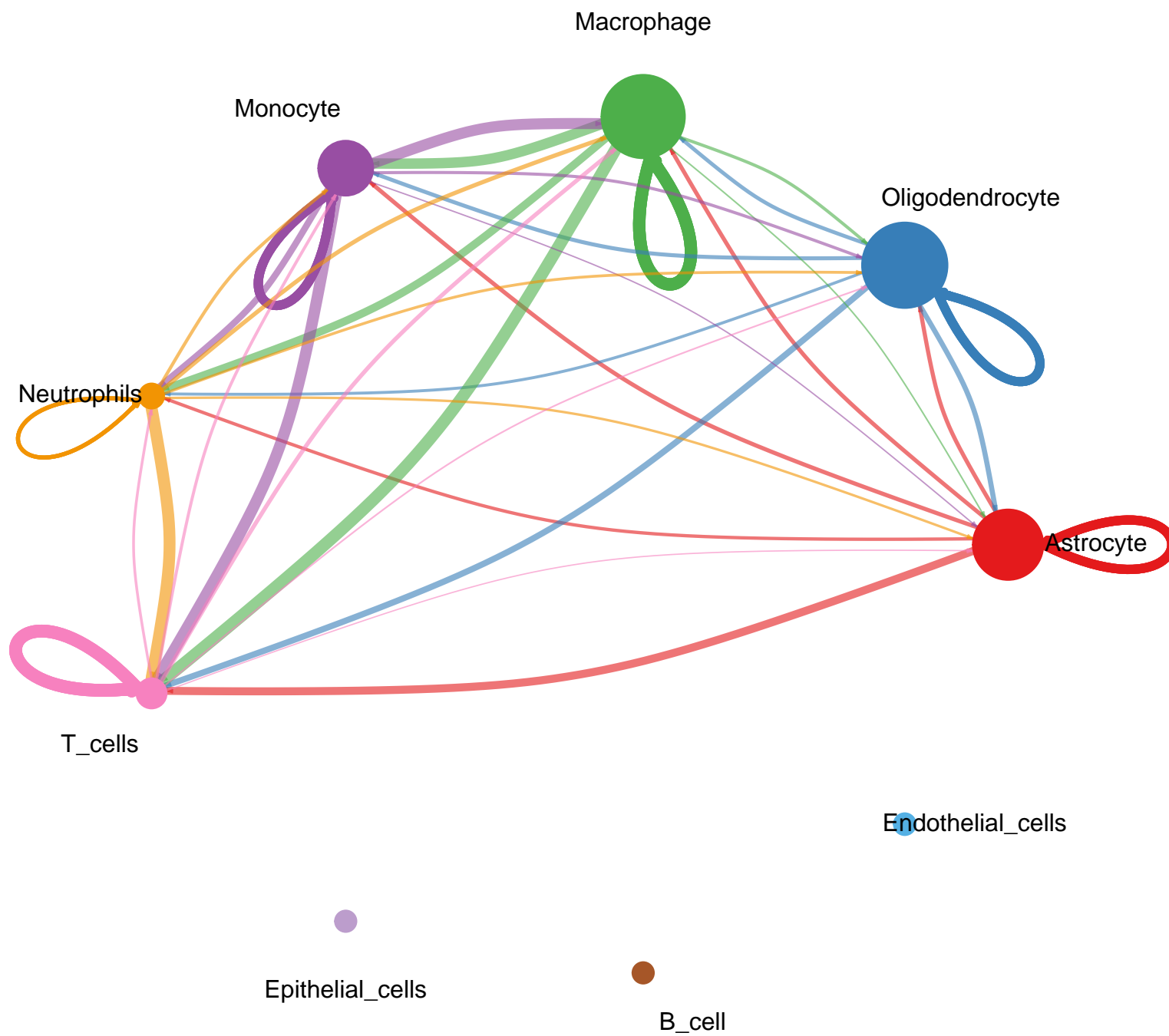

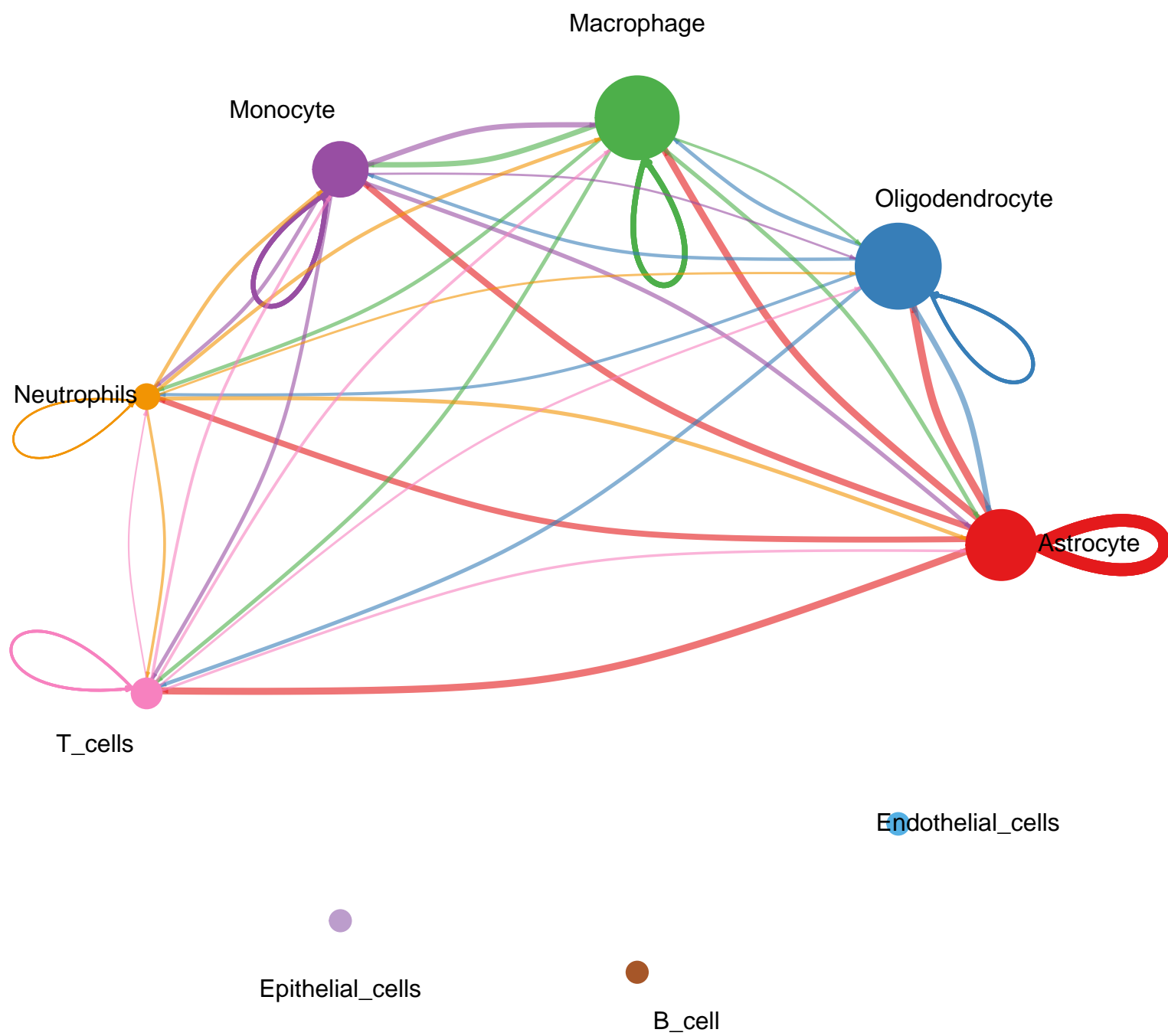

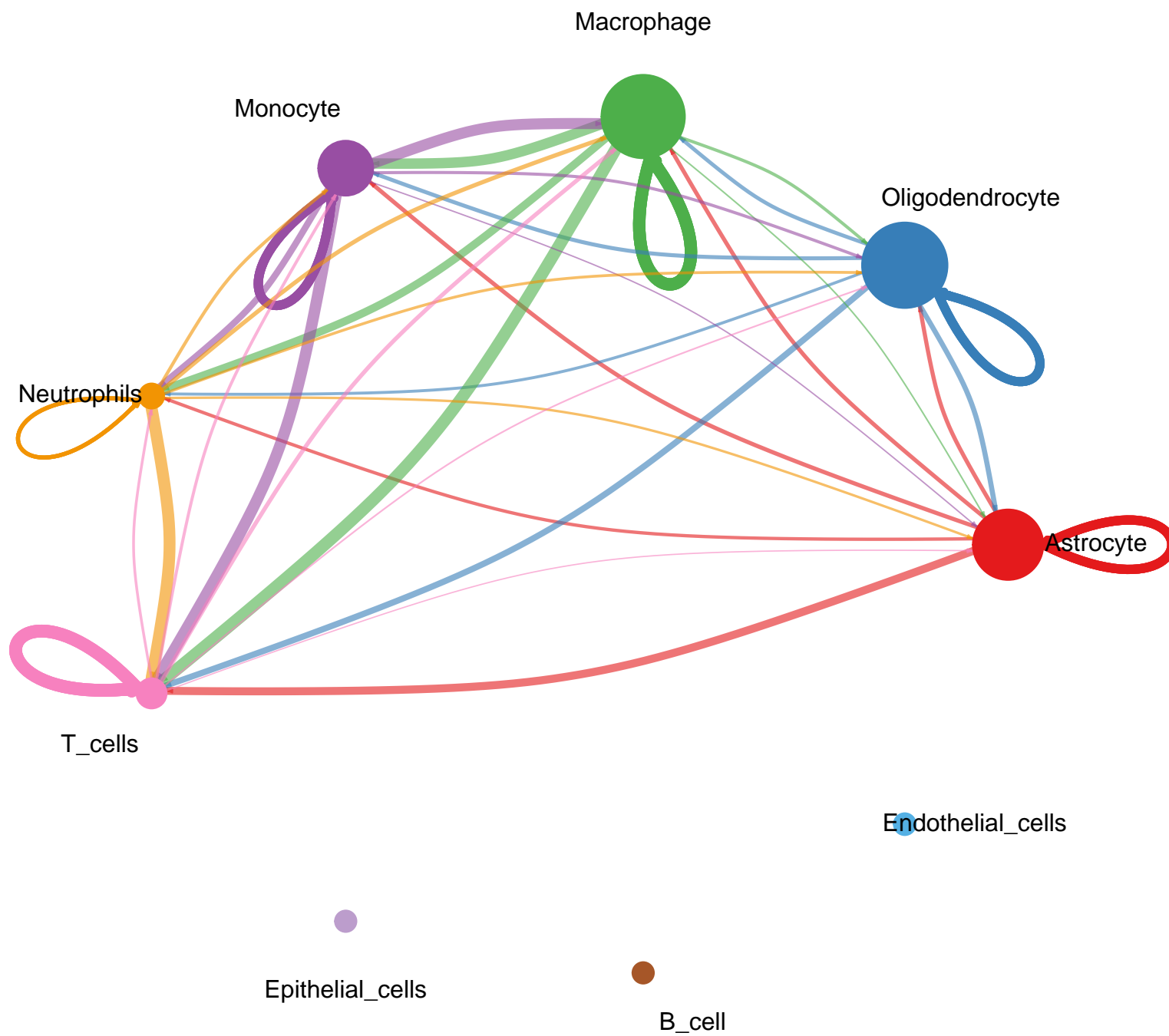

Supplement: Supplementary file 3 [file DataSheet1.pdf]

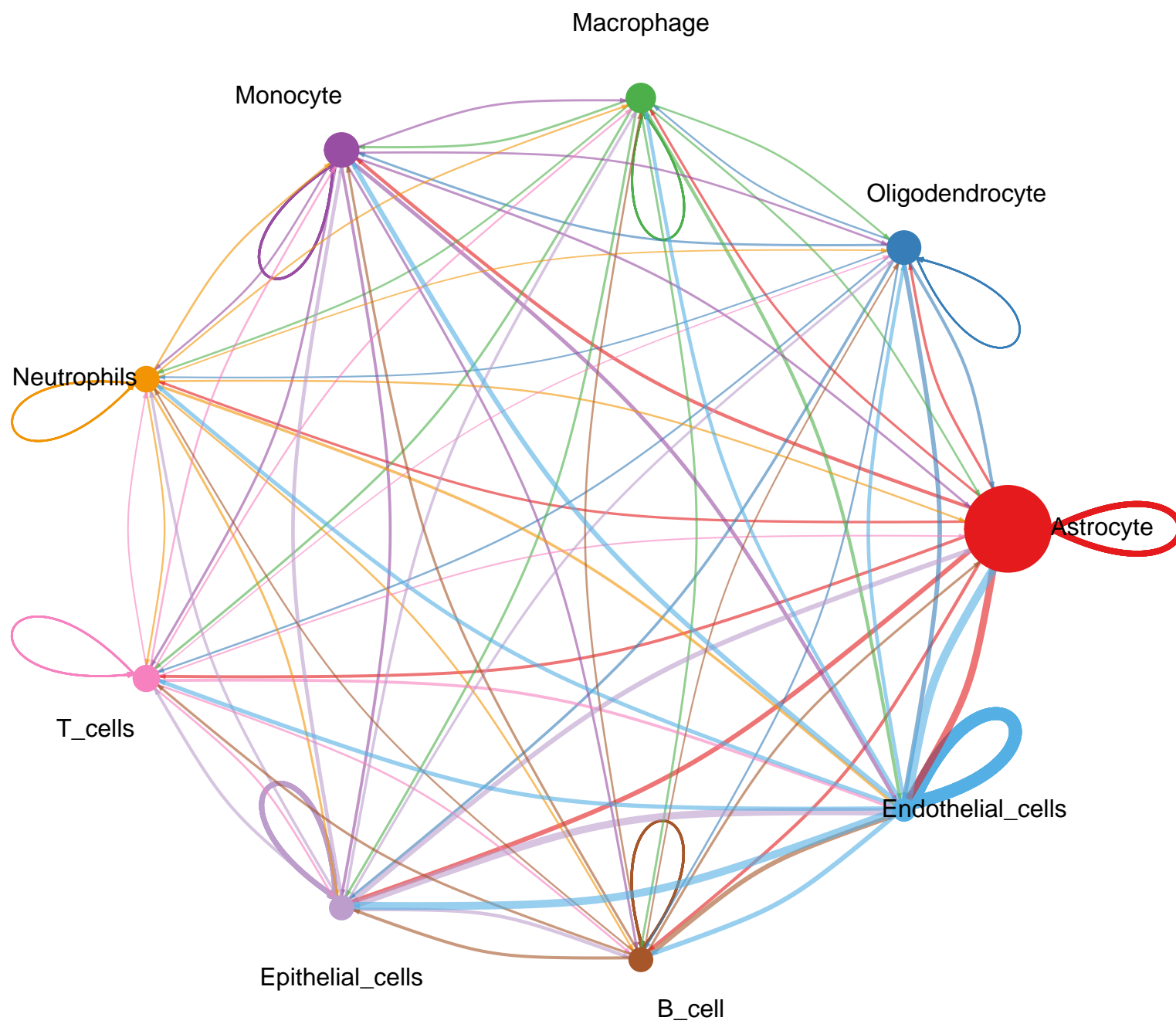

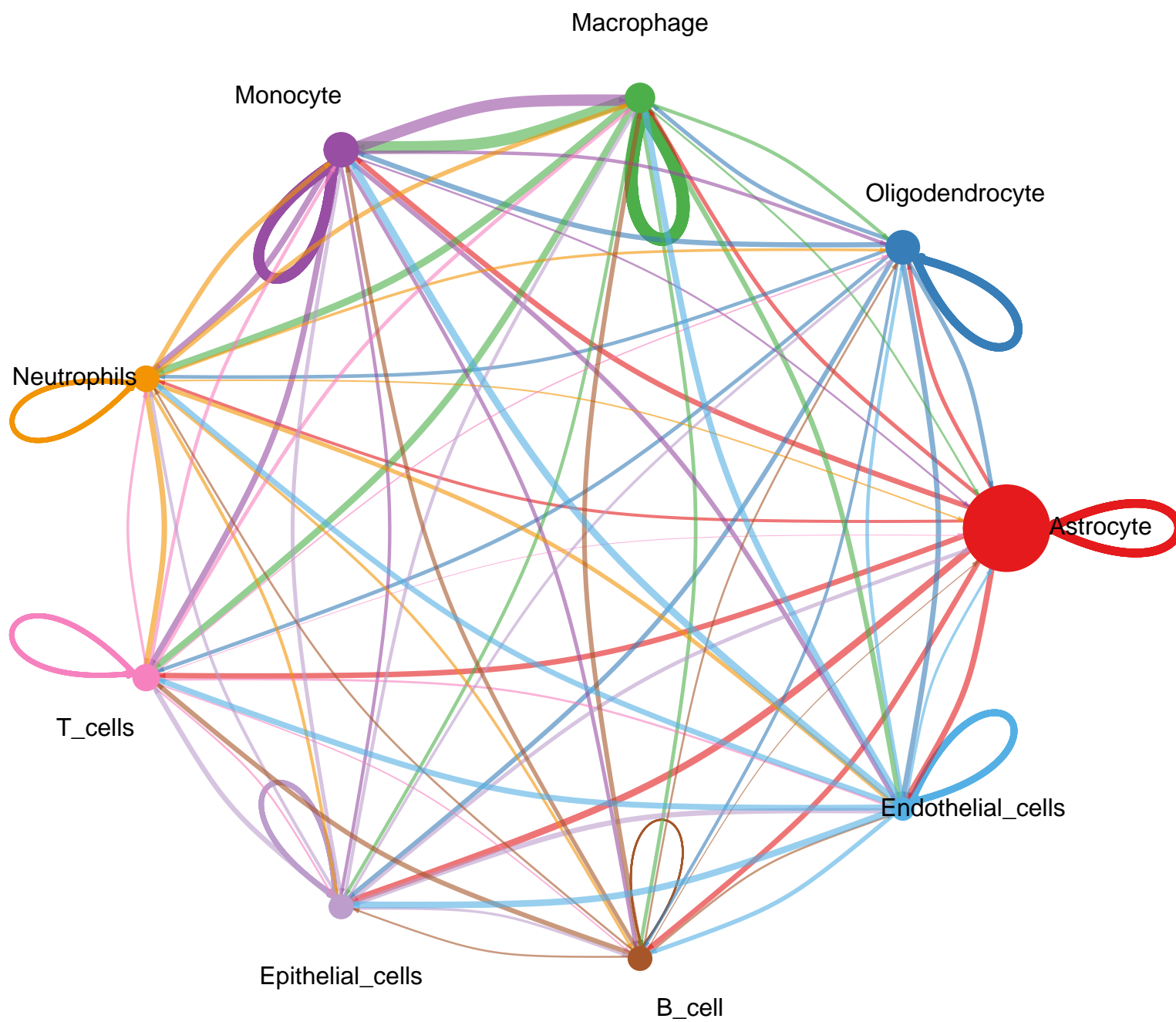

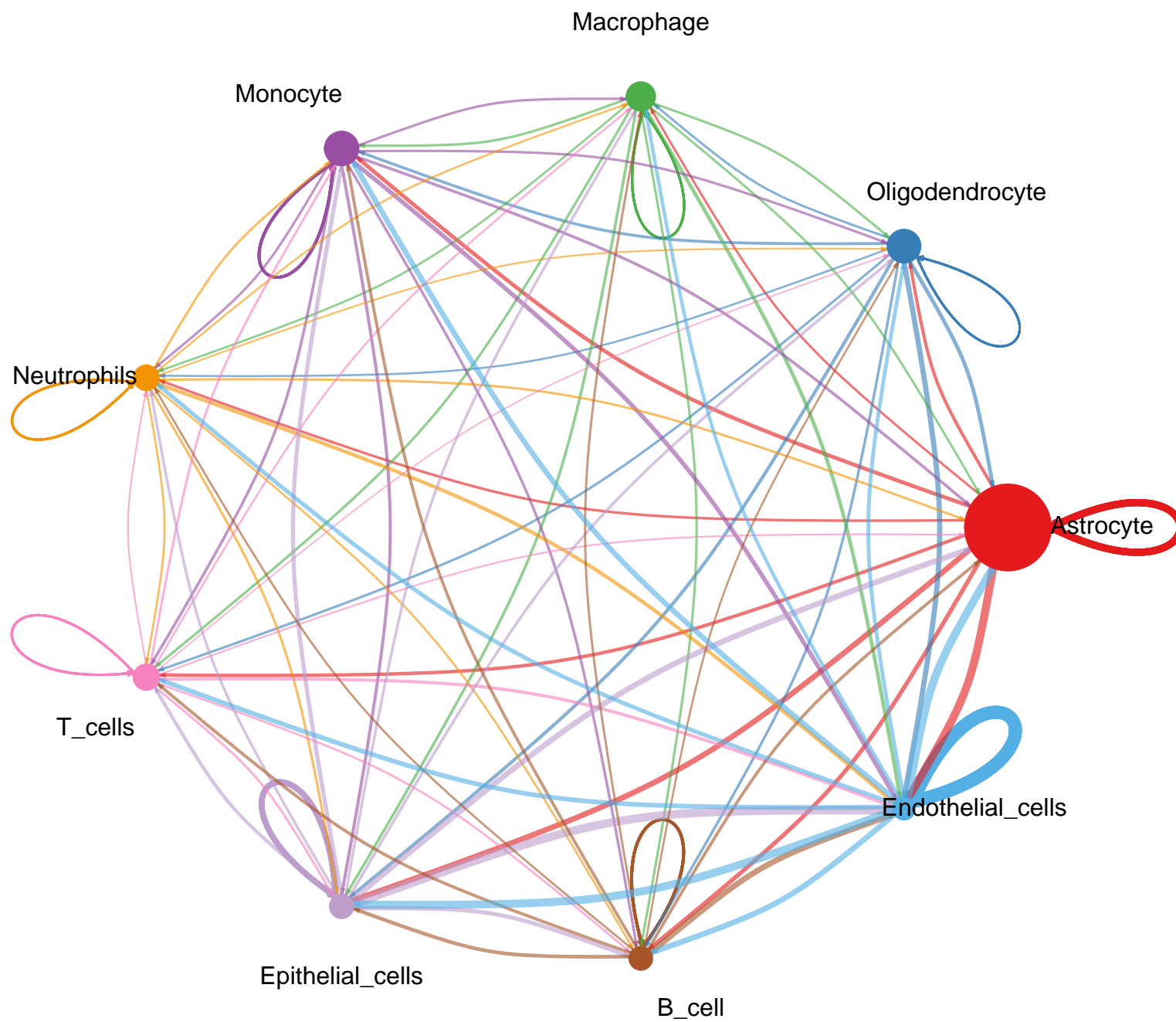

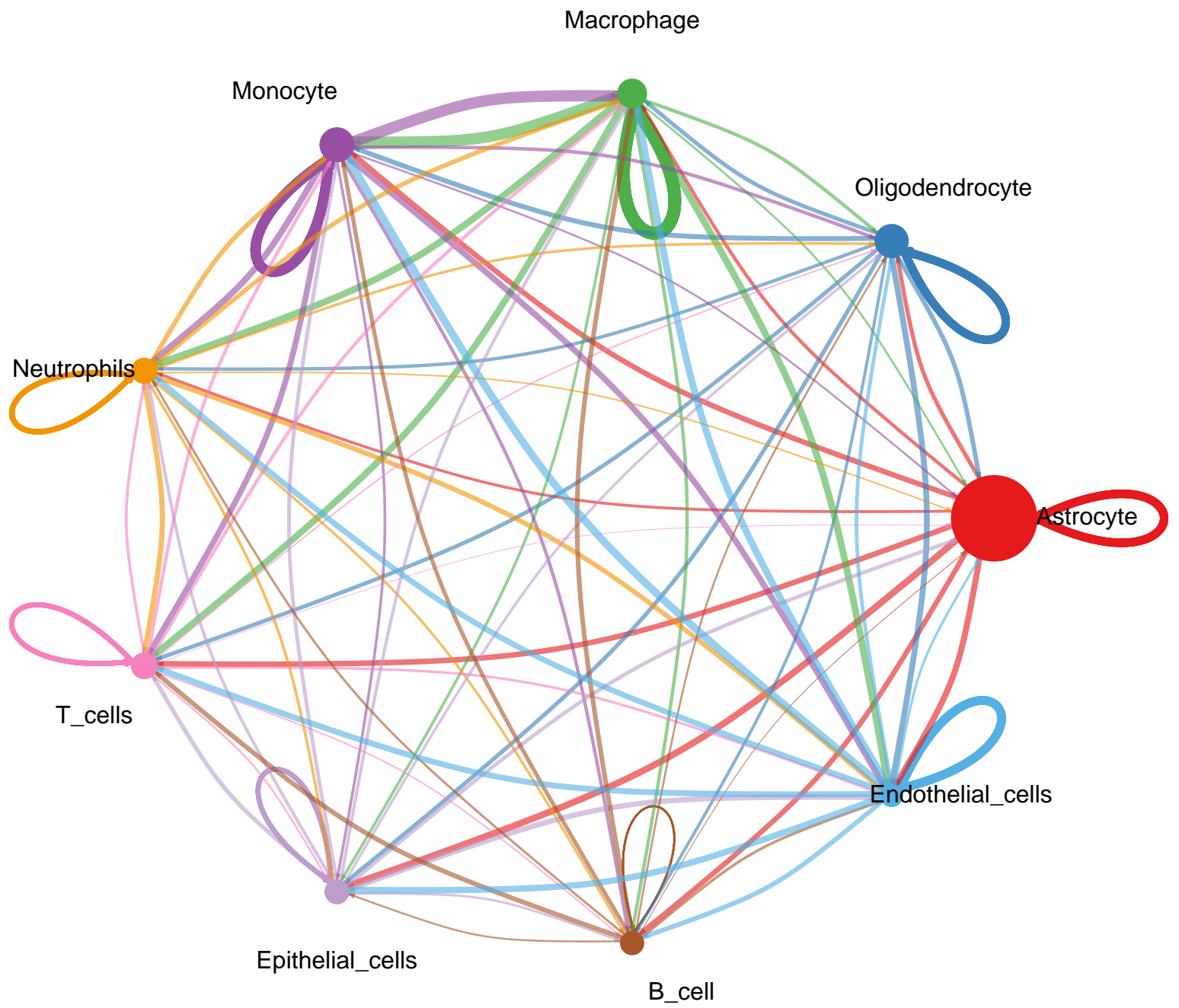

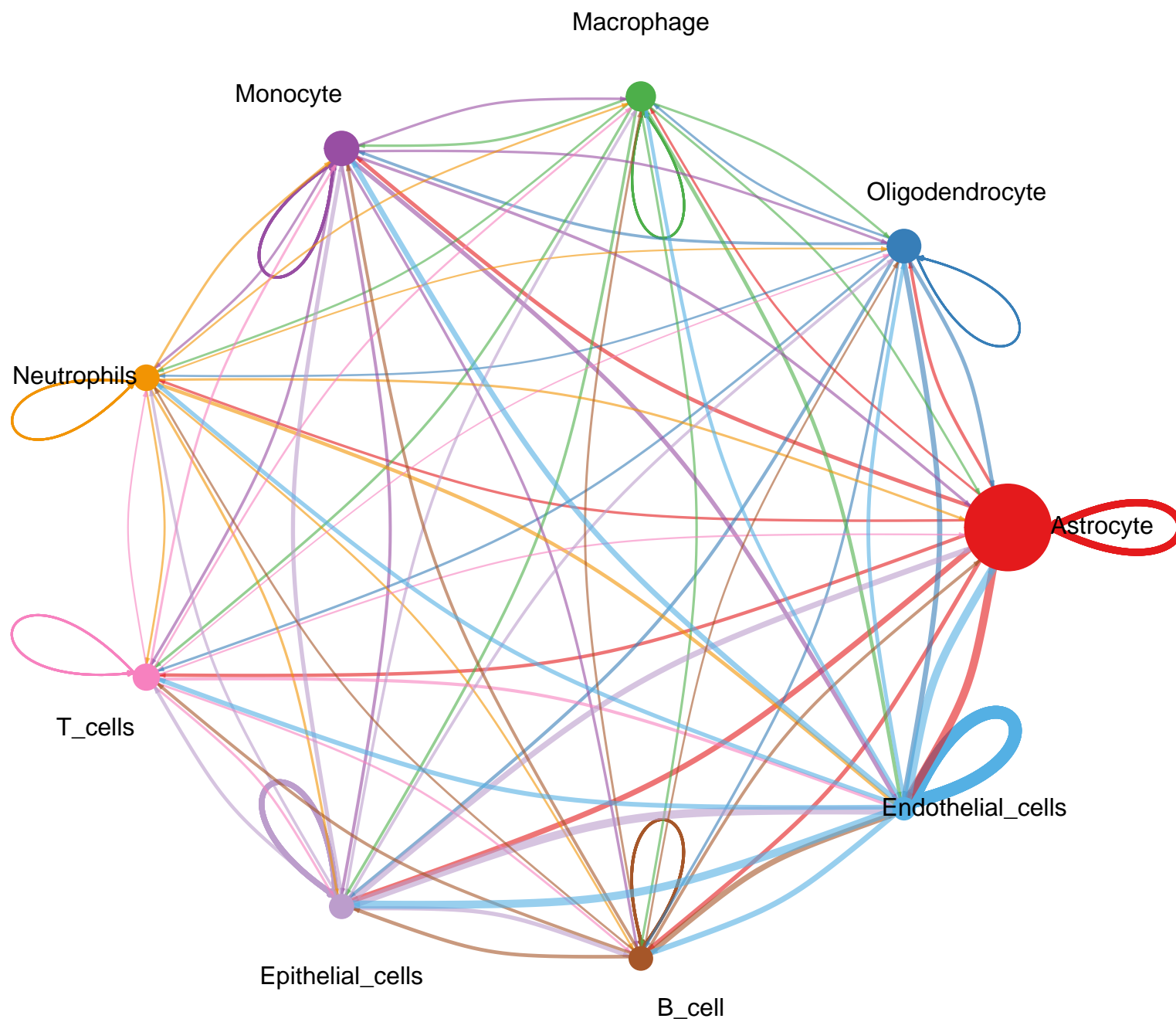

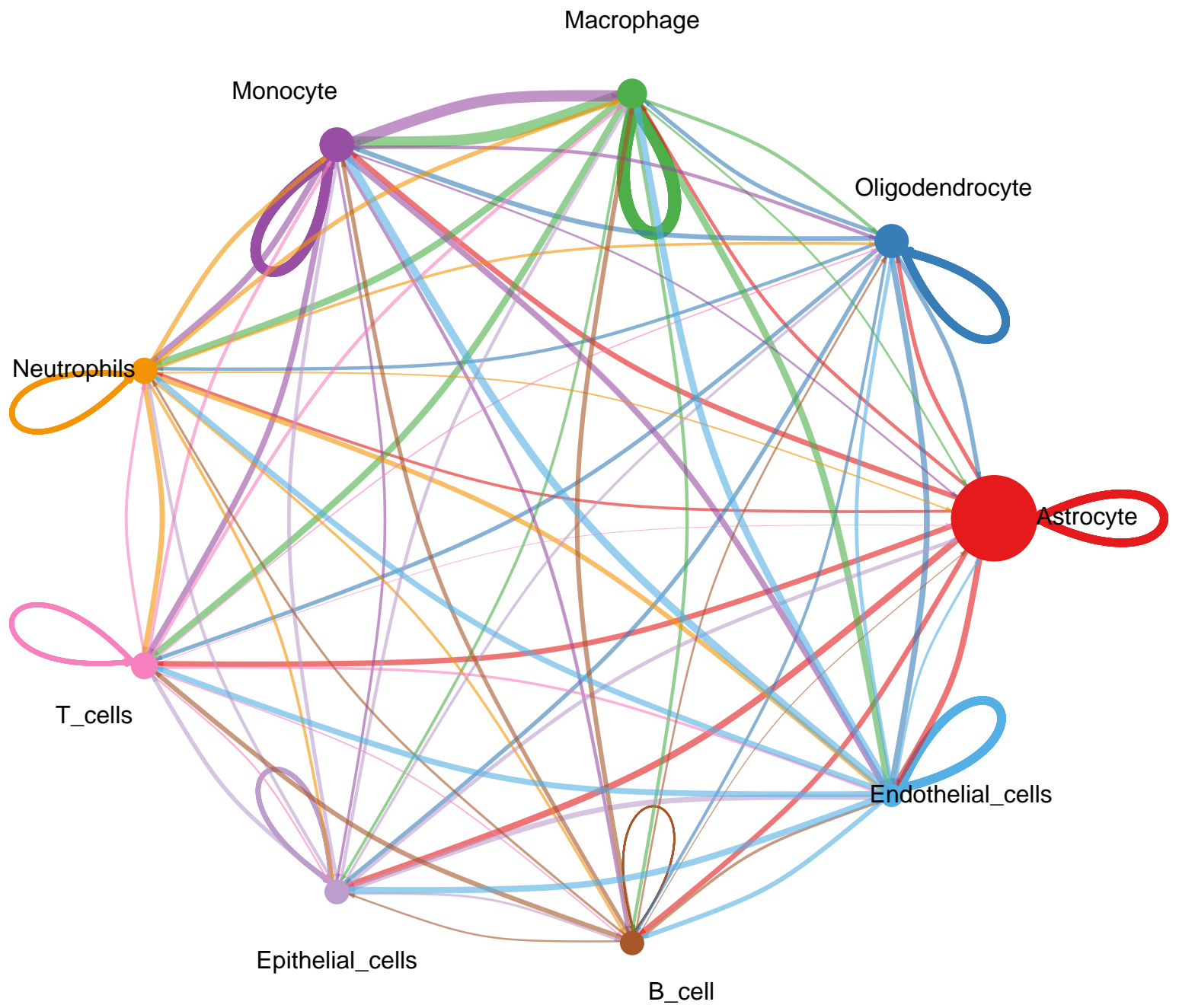

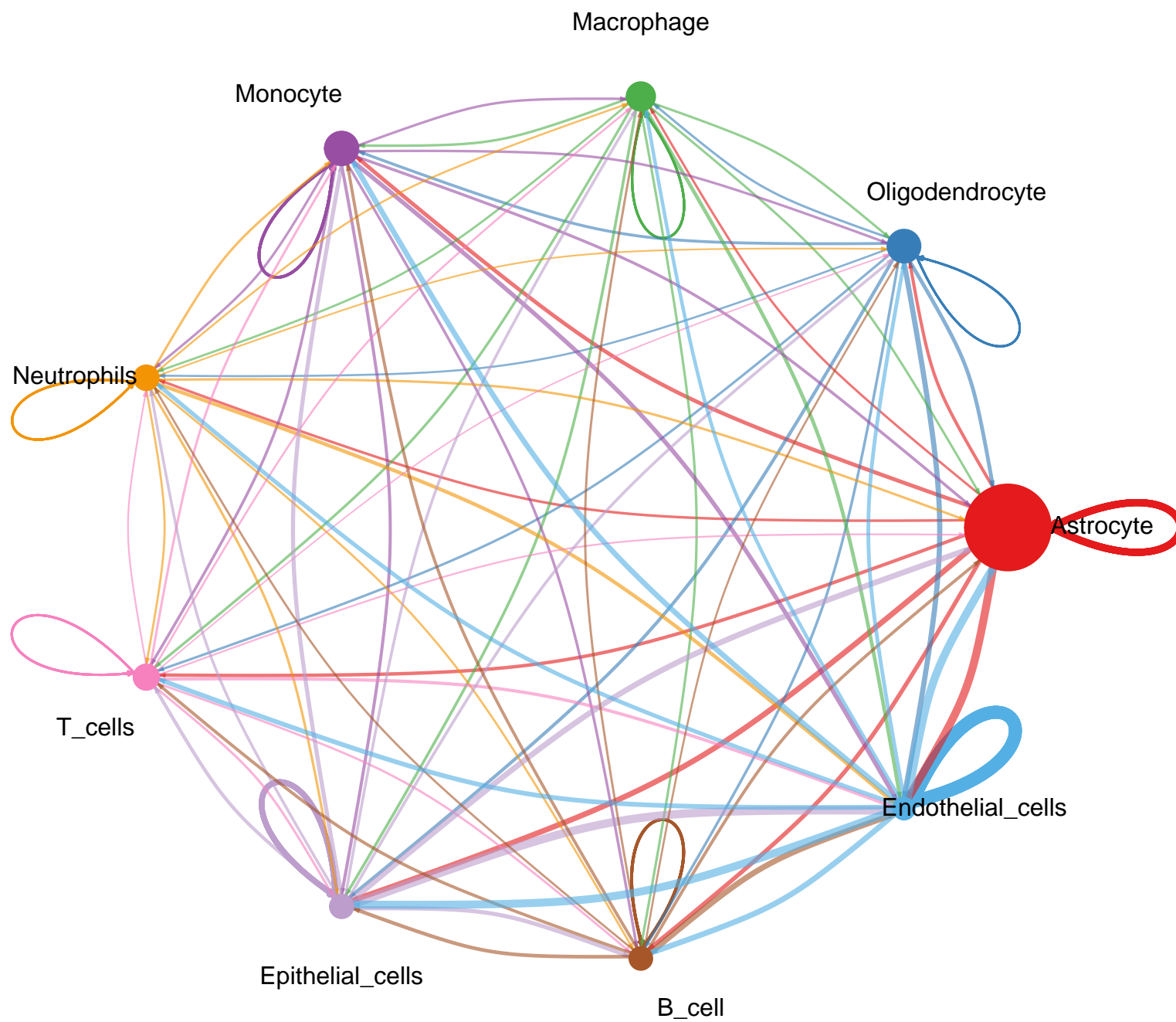

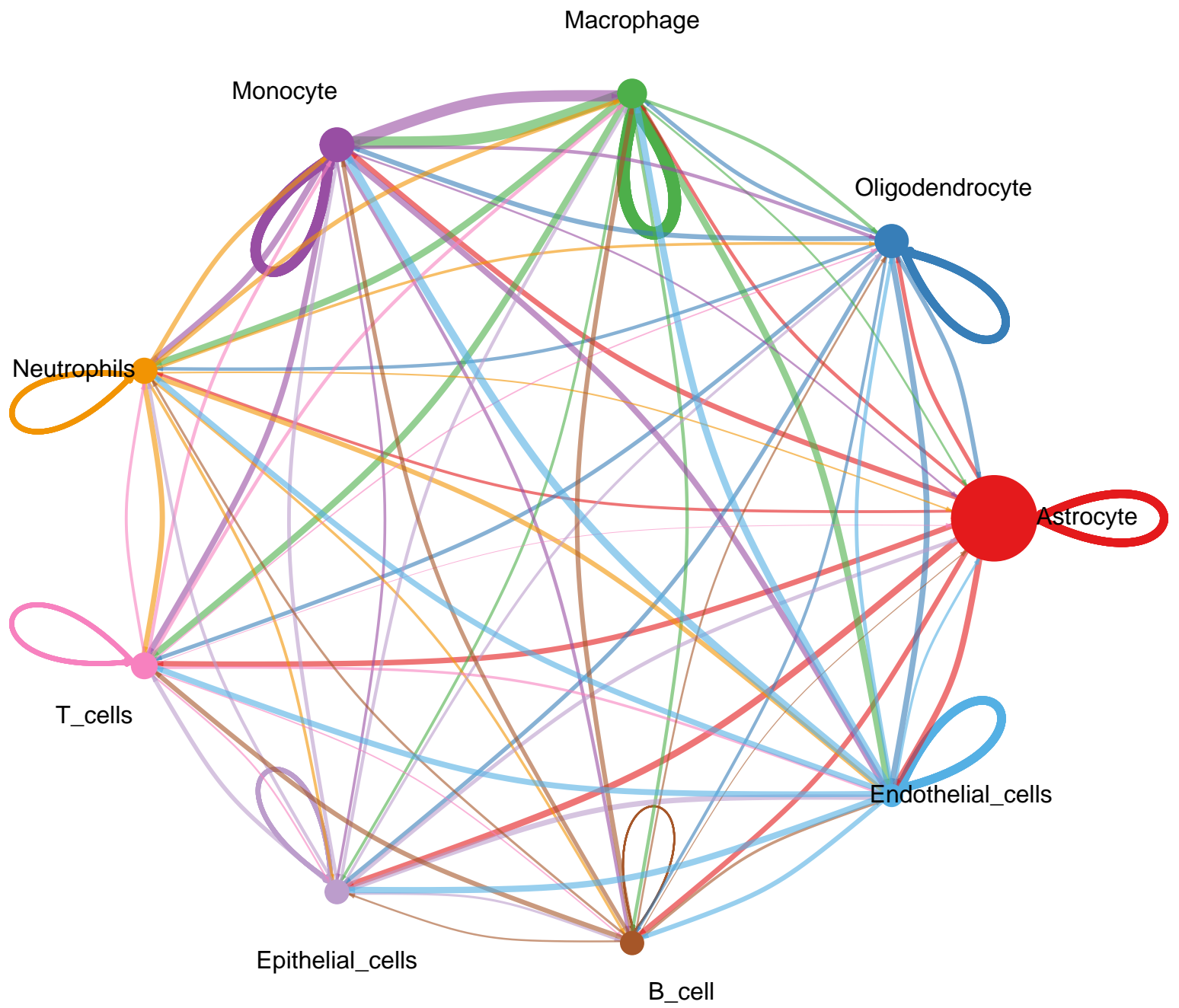

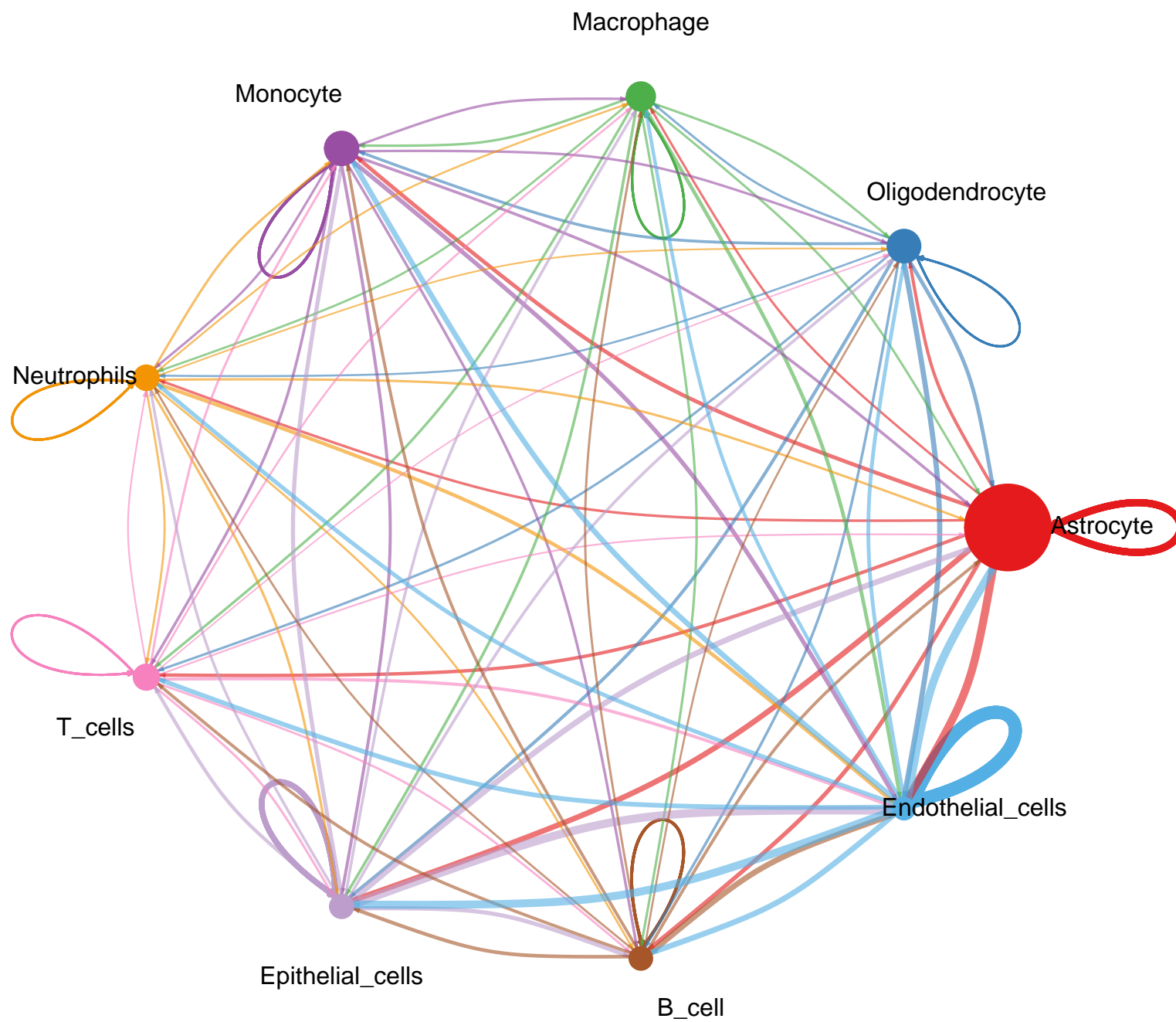

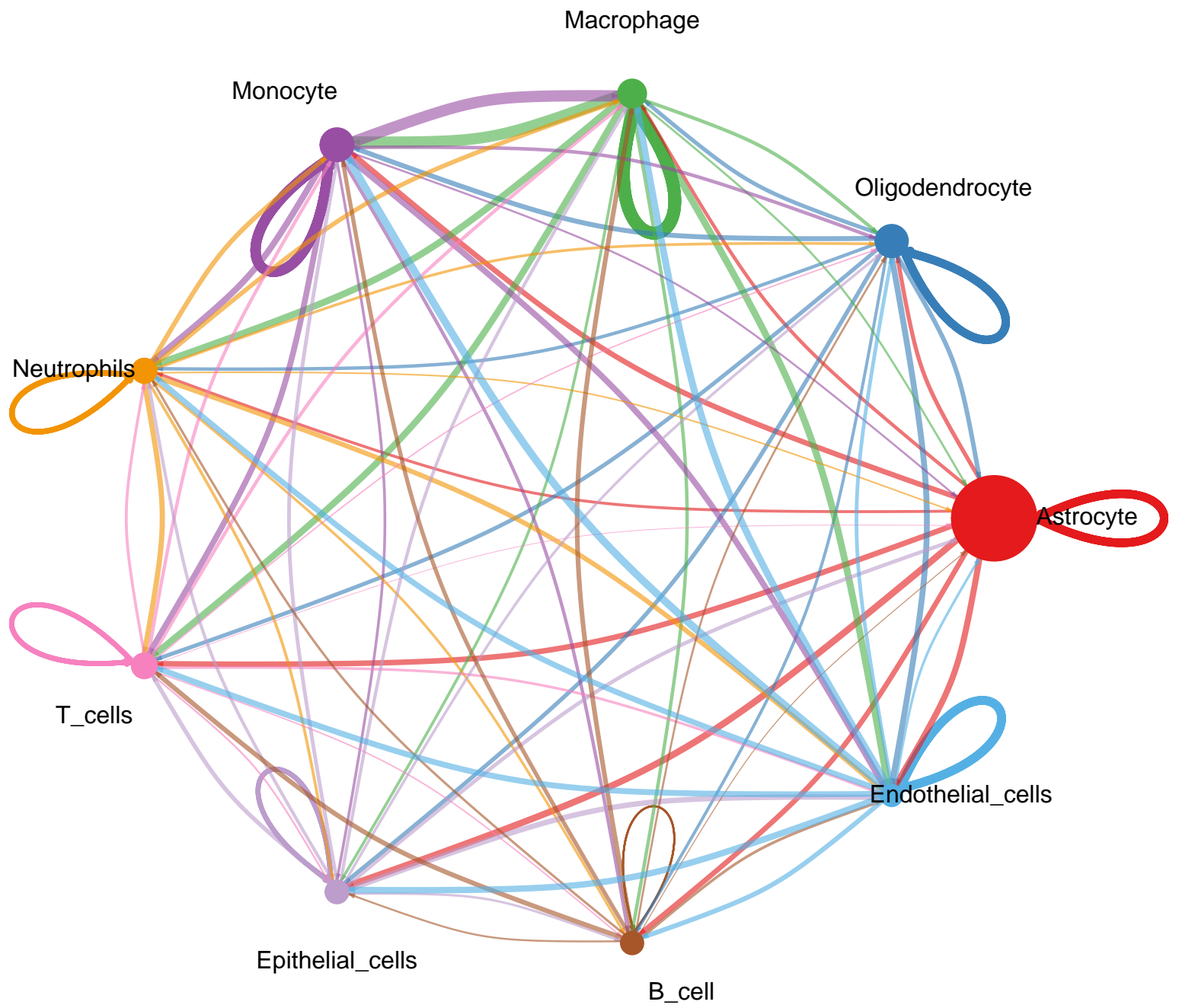

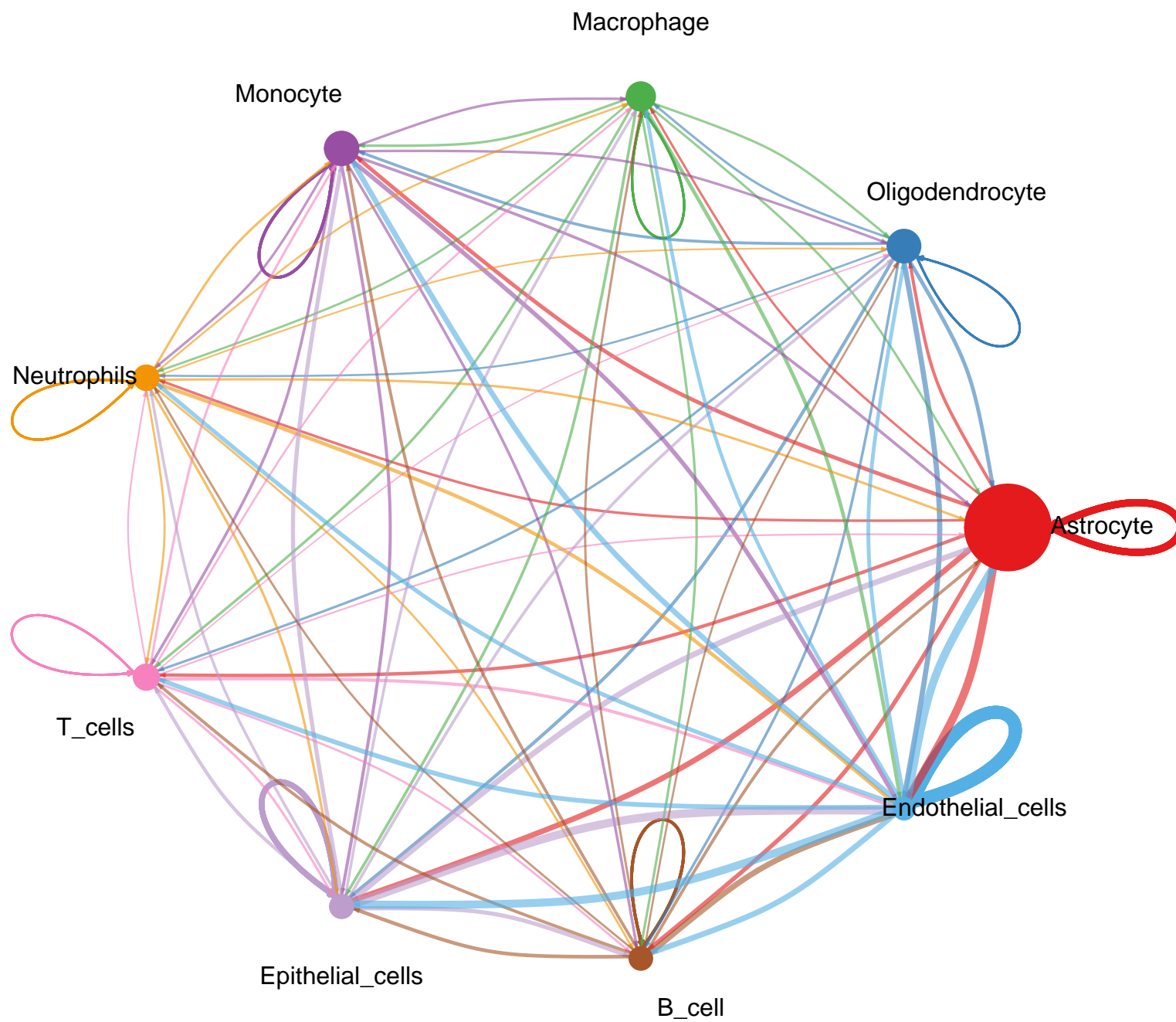

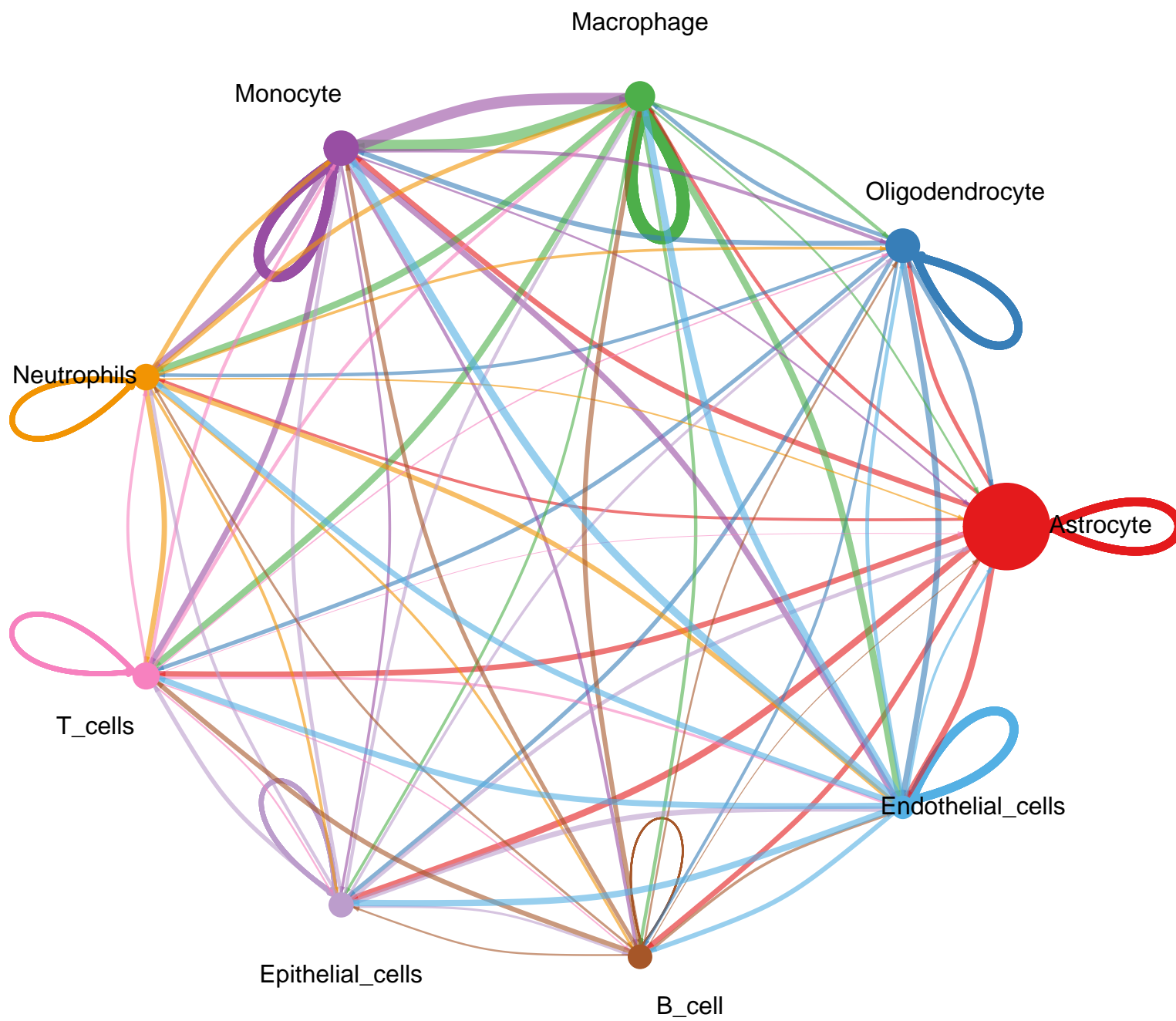

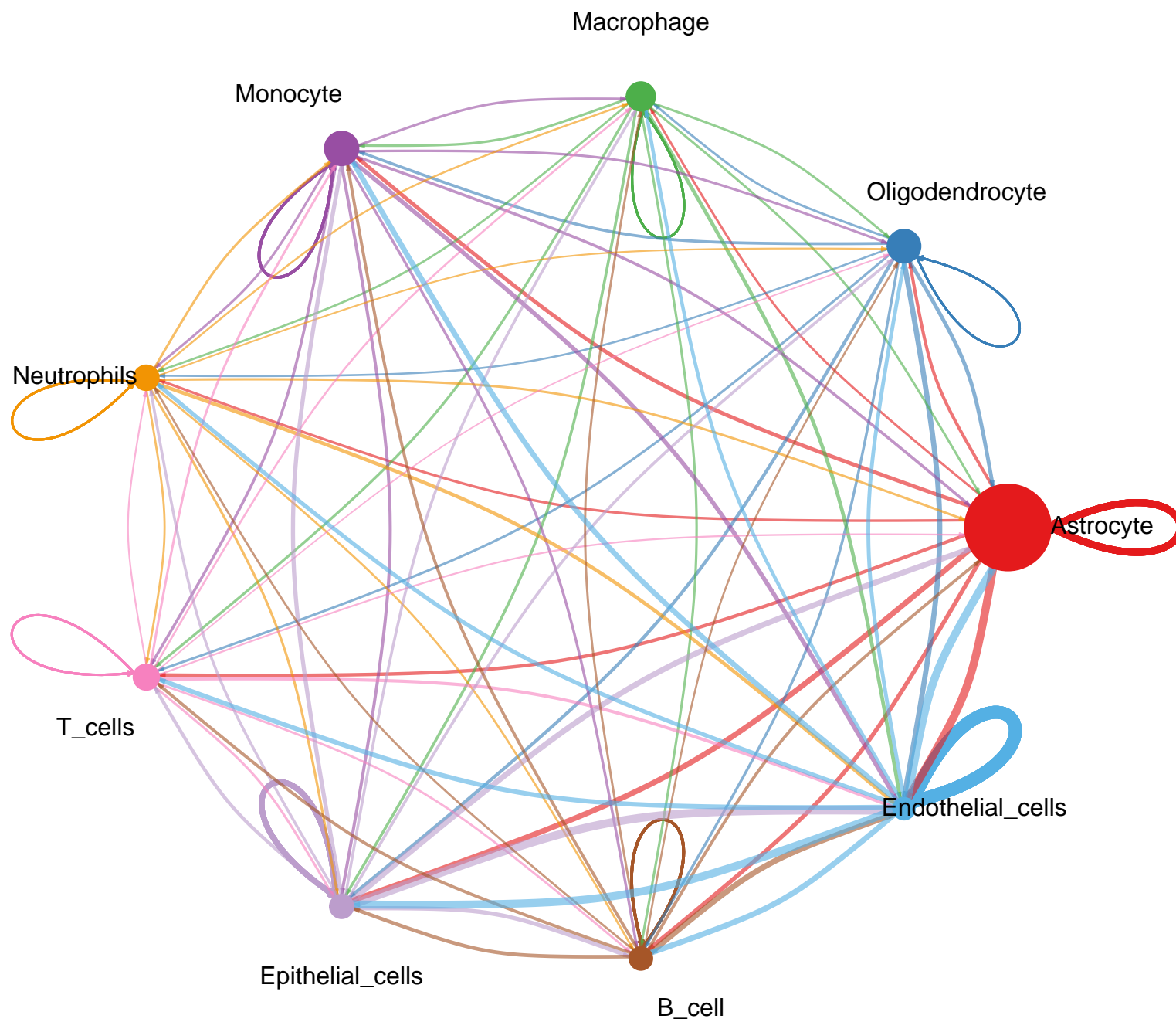

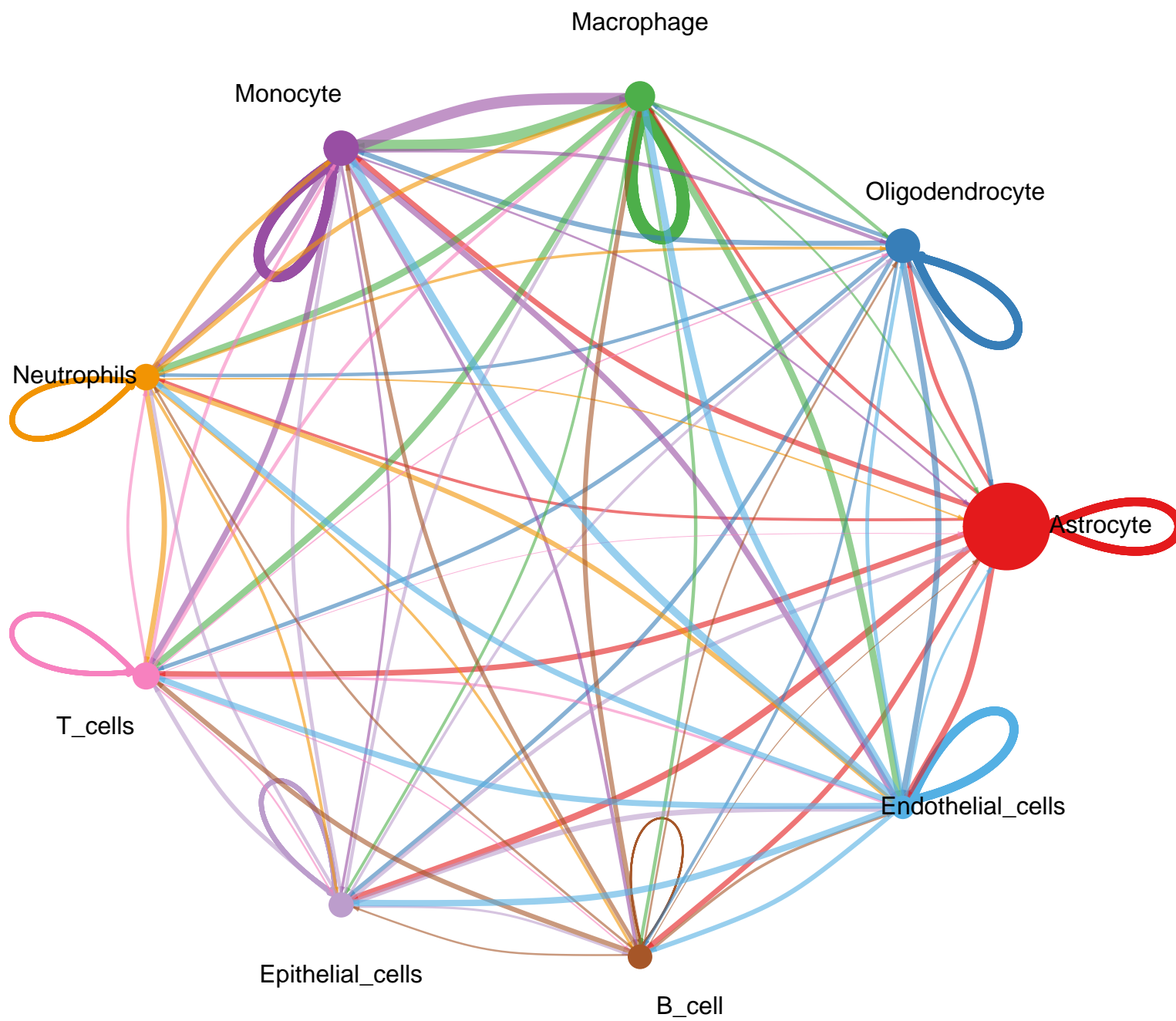

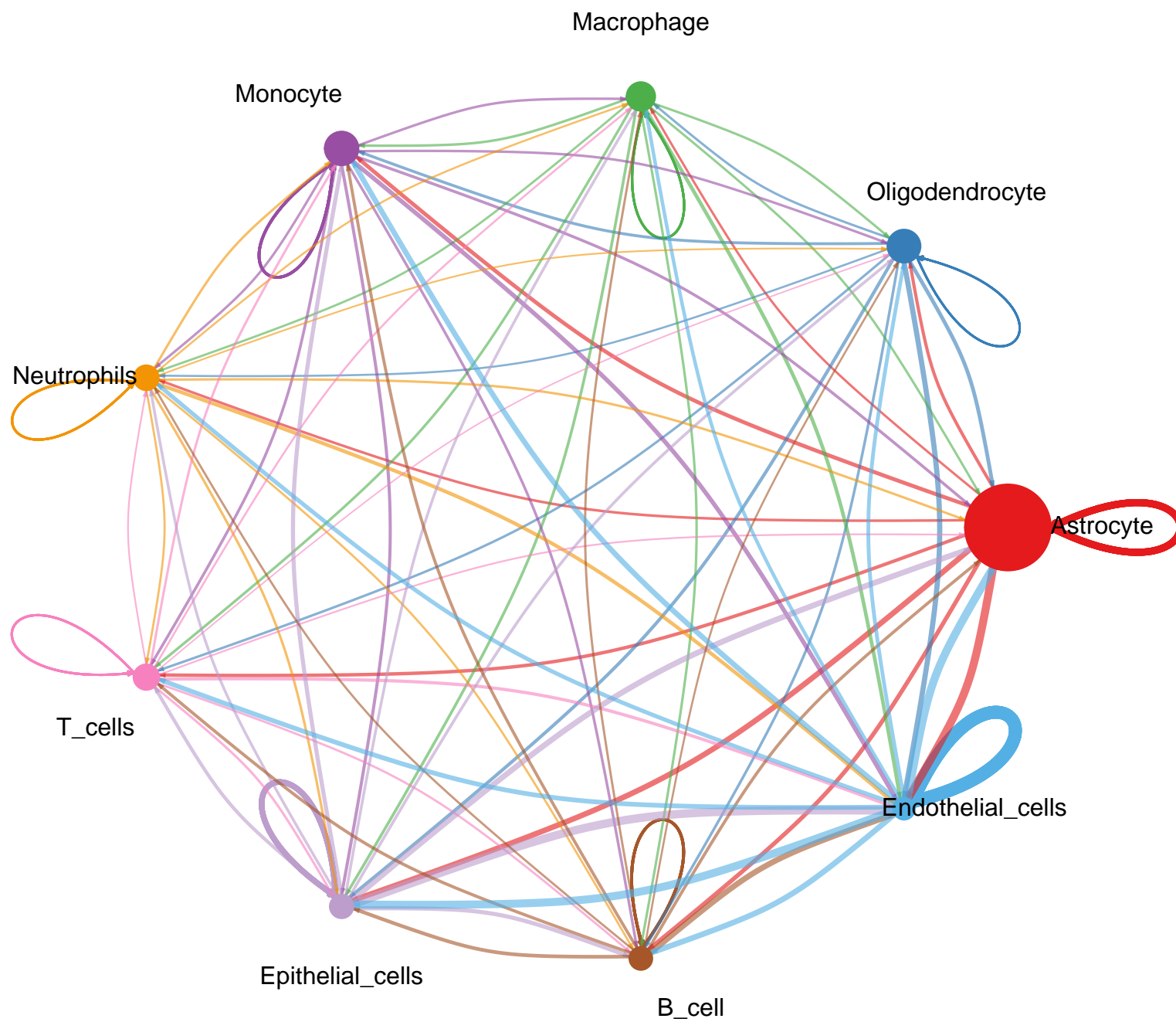

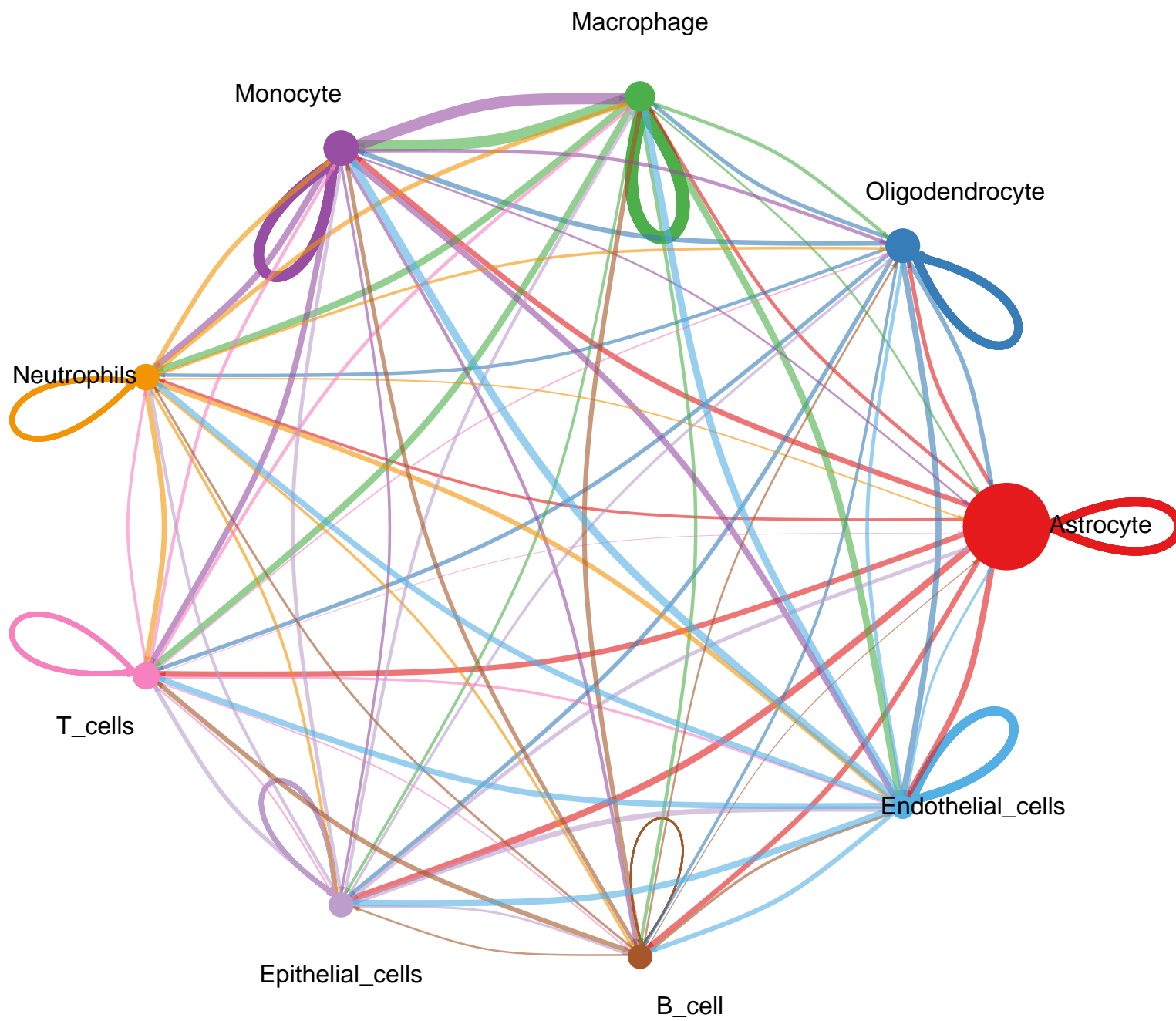

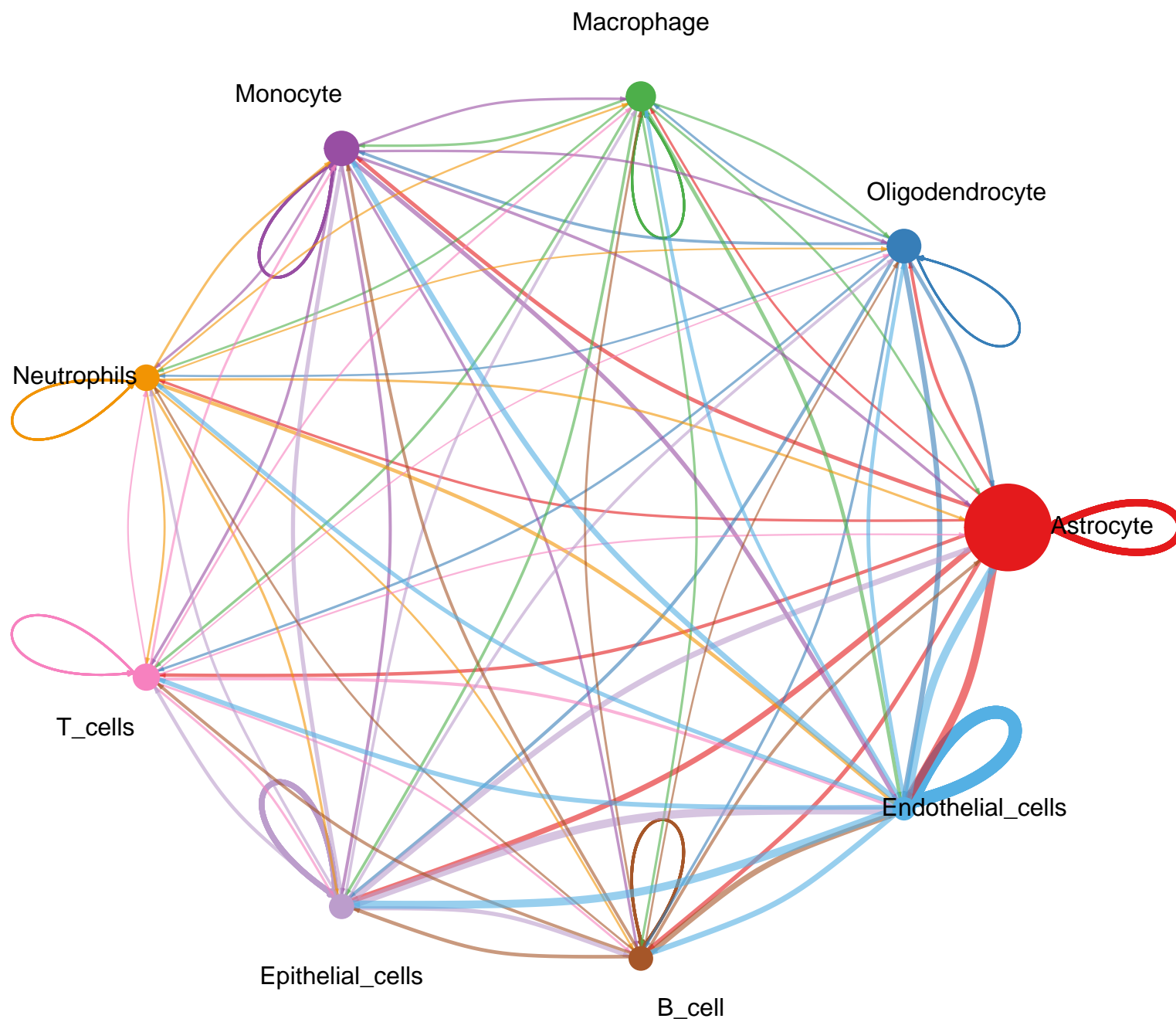

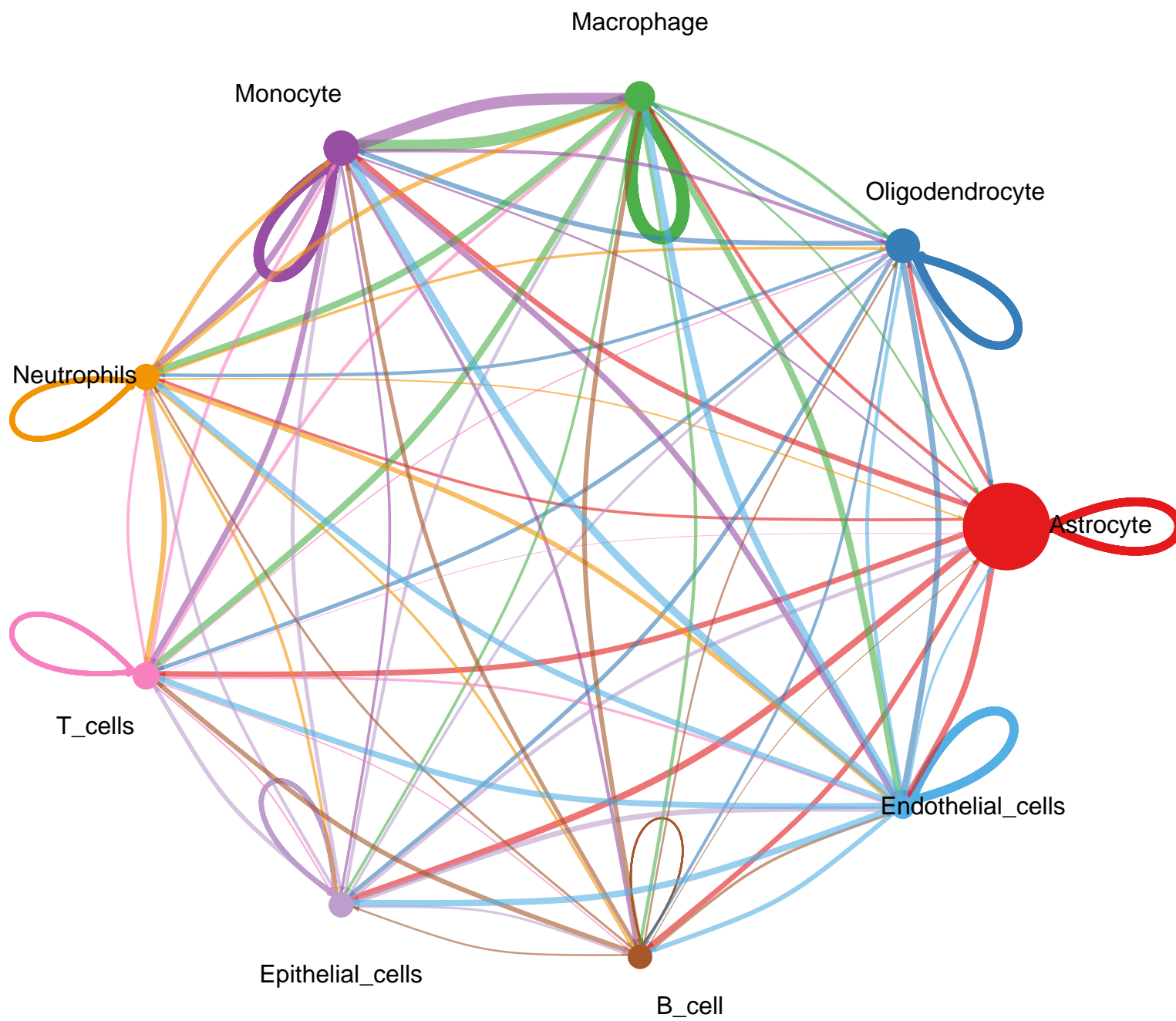

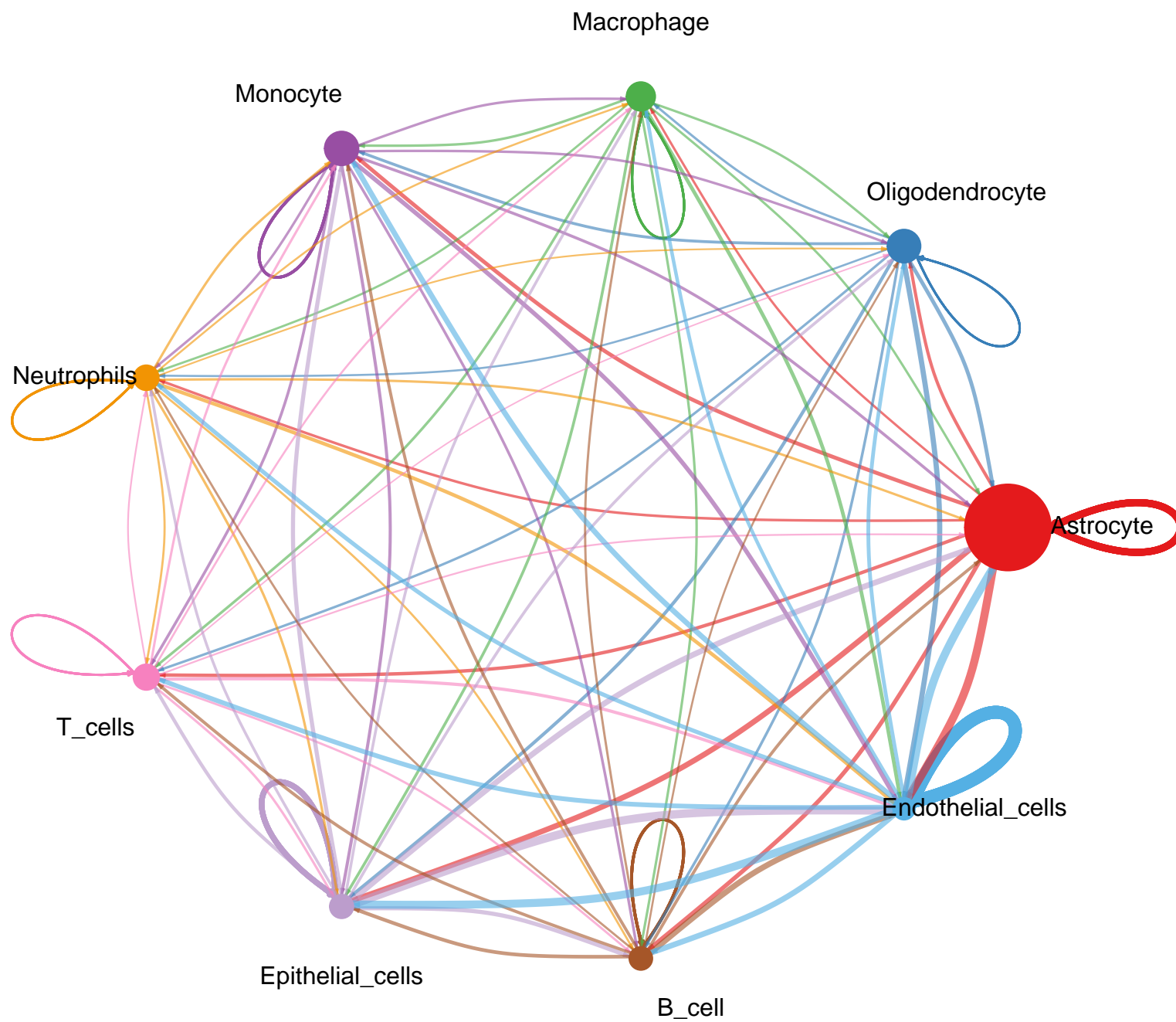

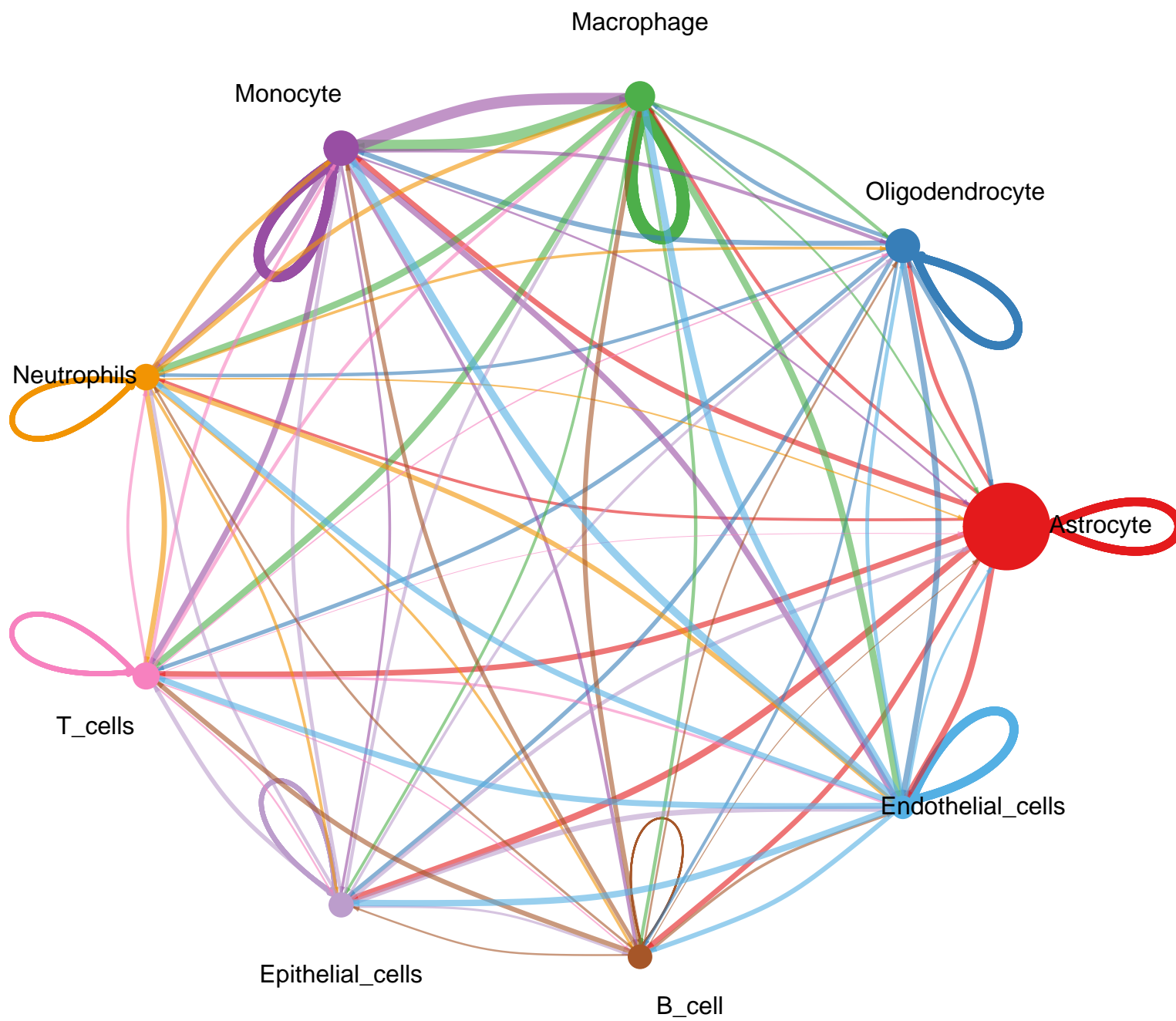

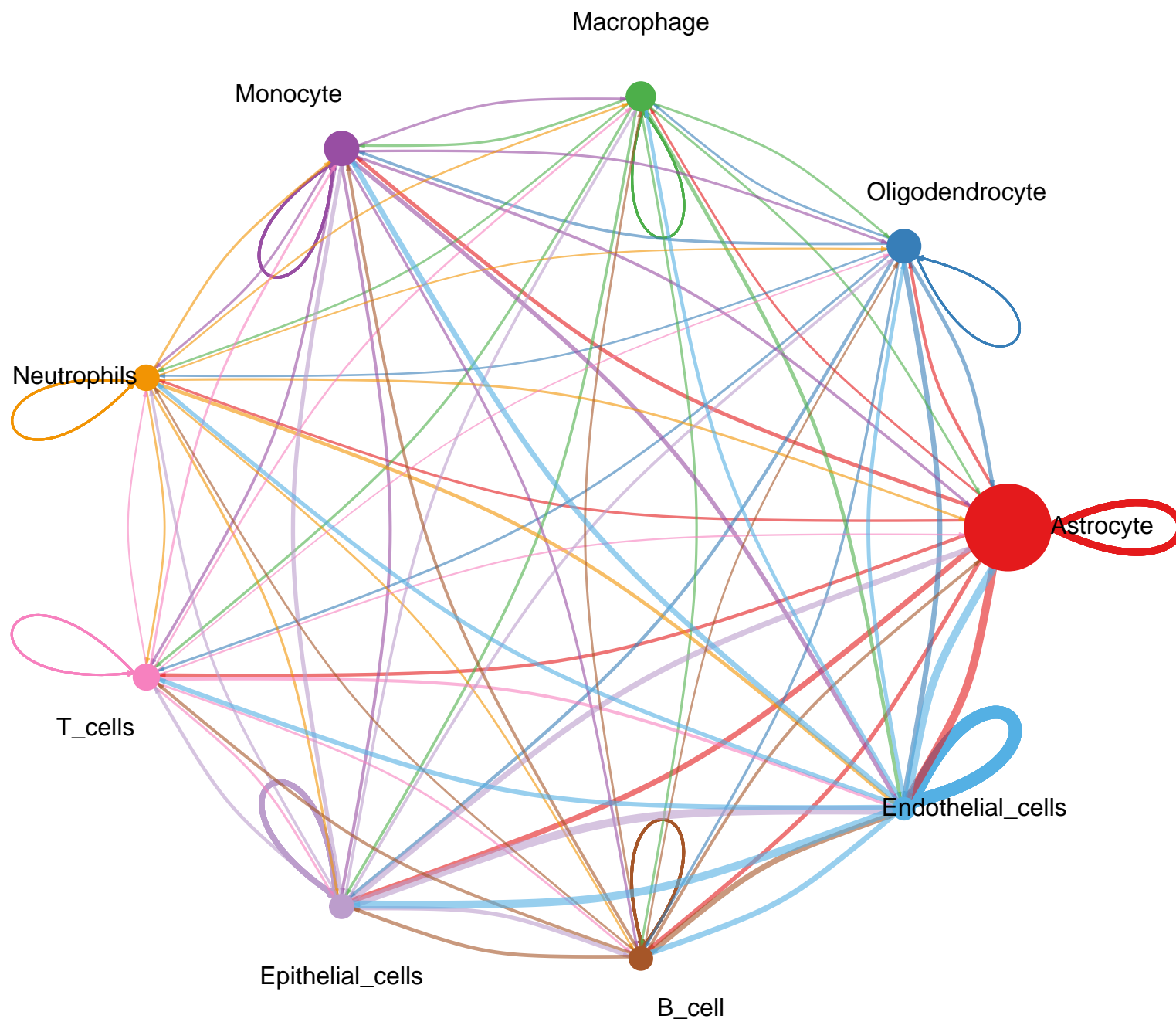

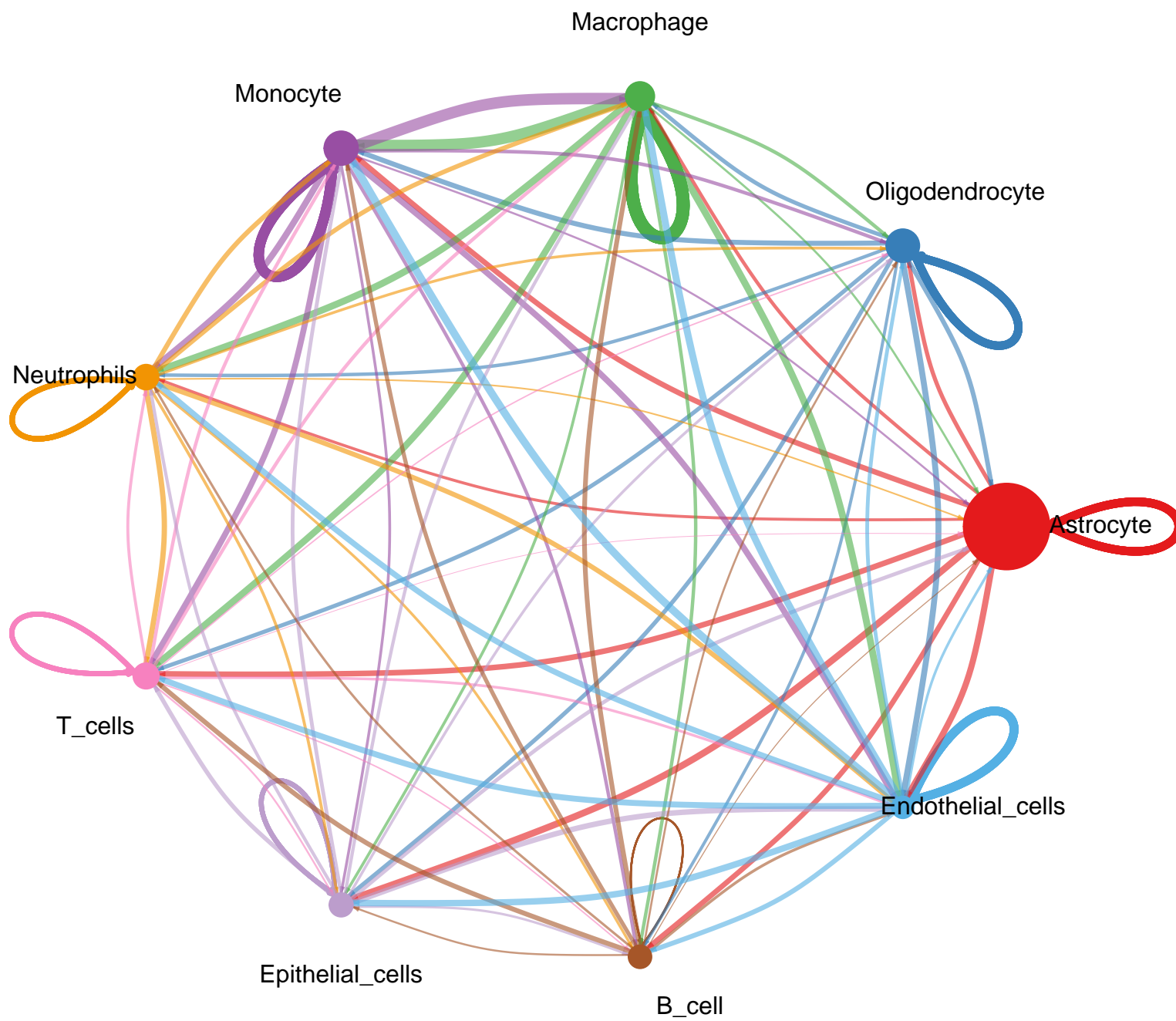

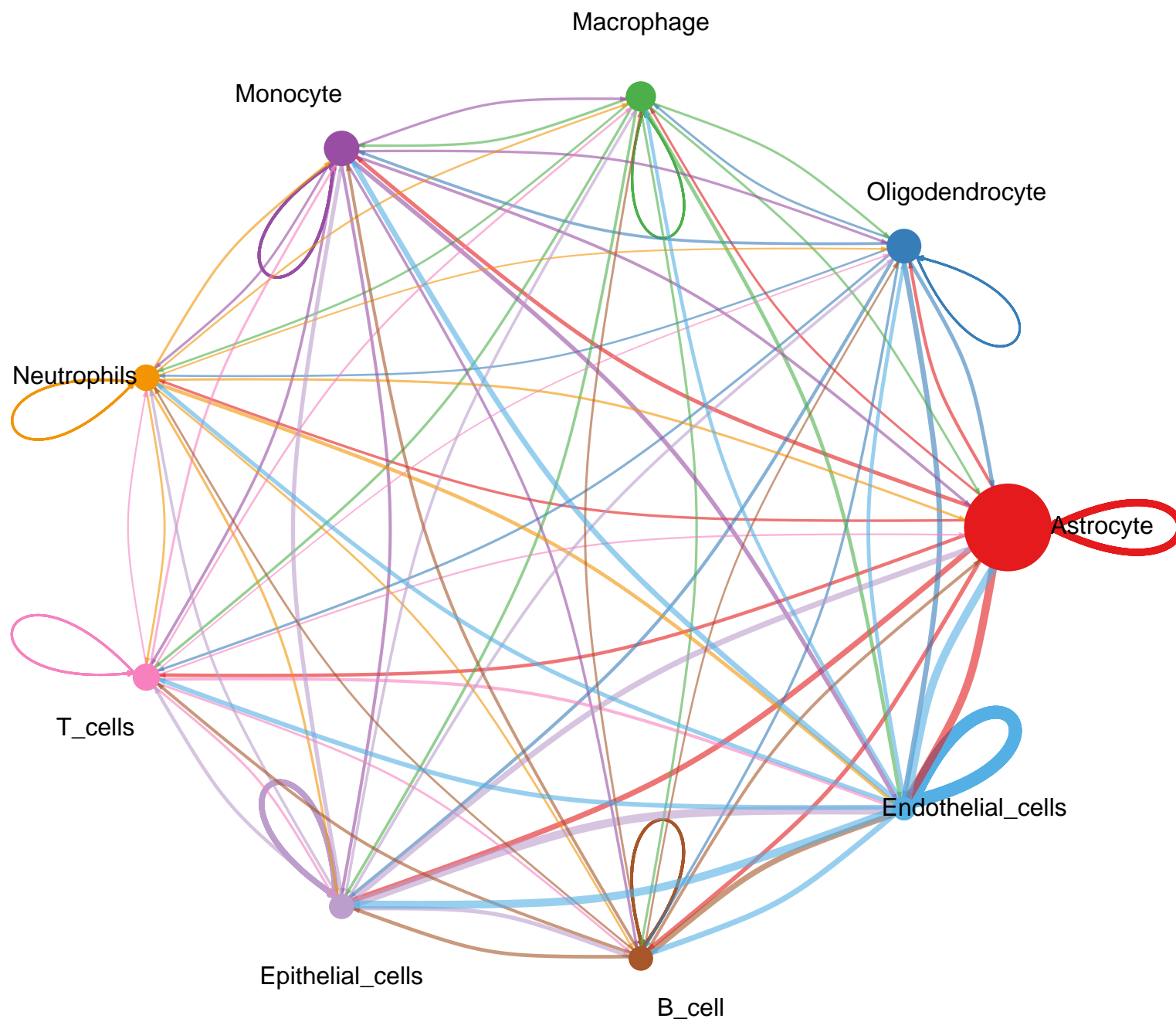

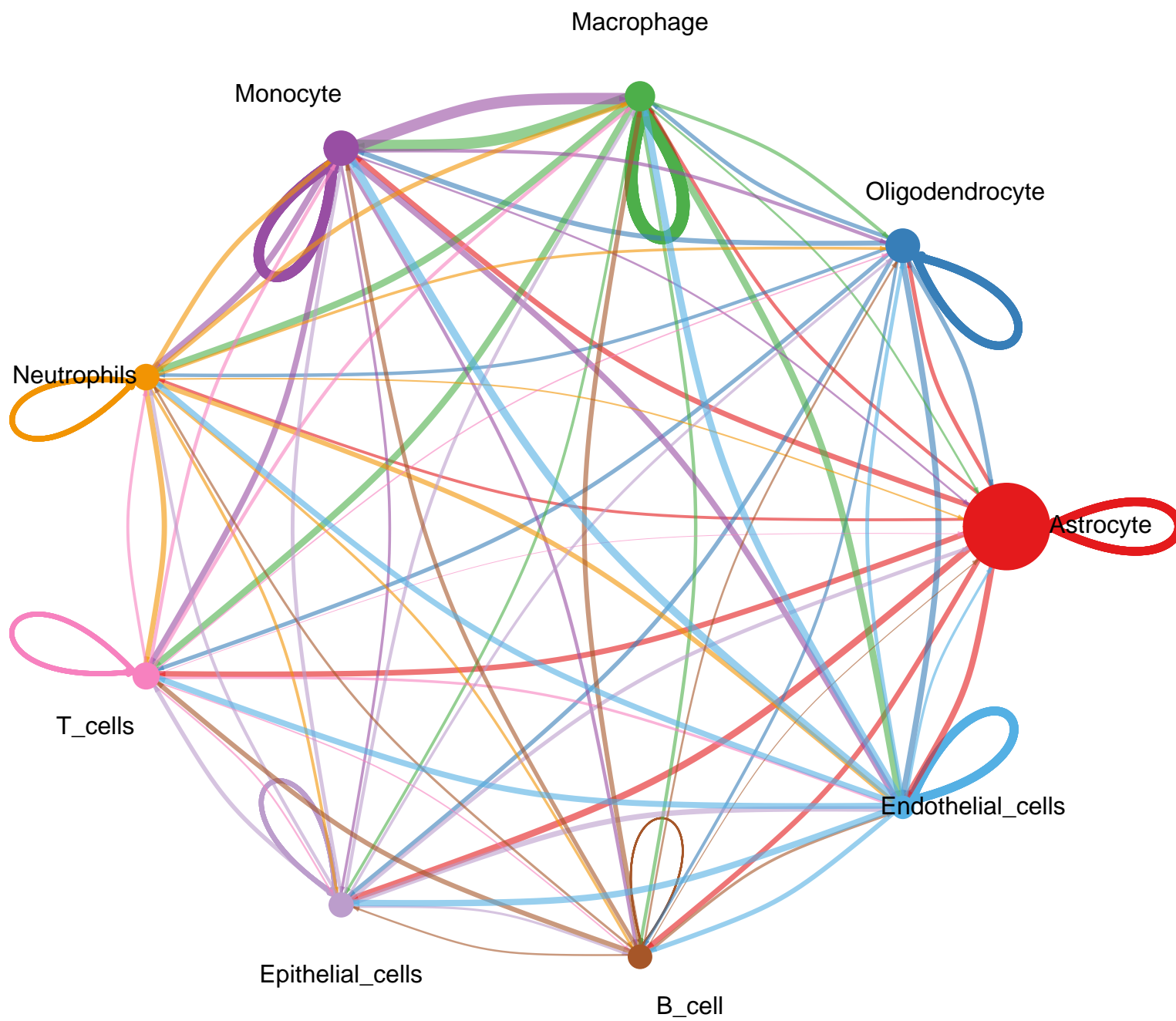

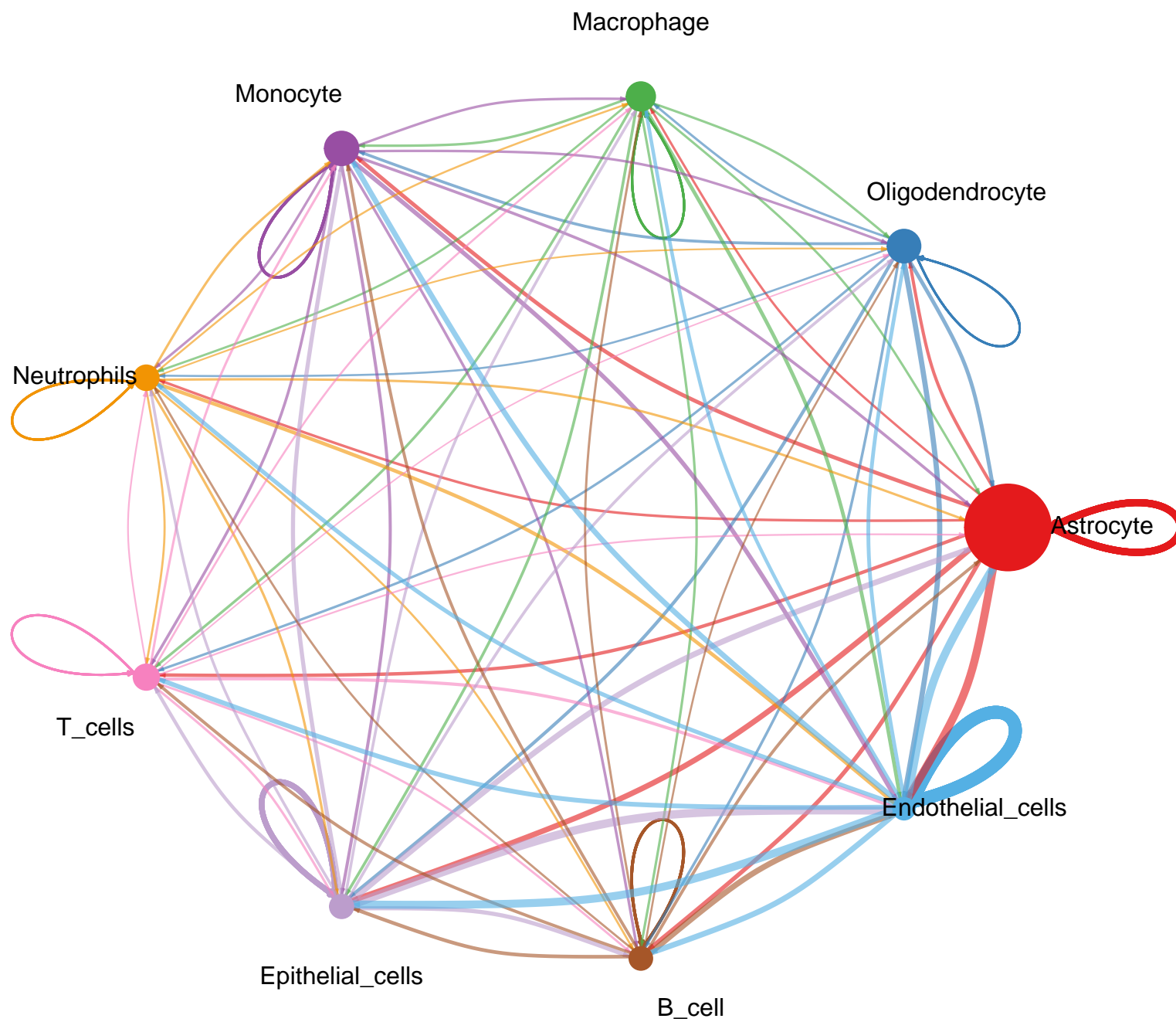

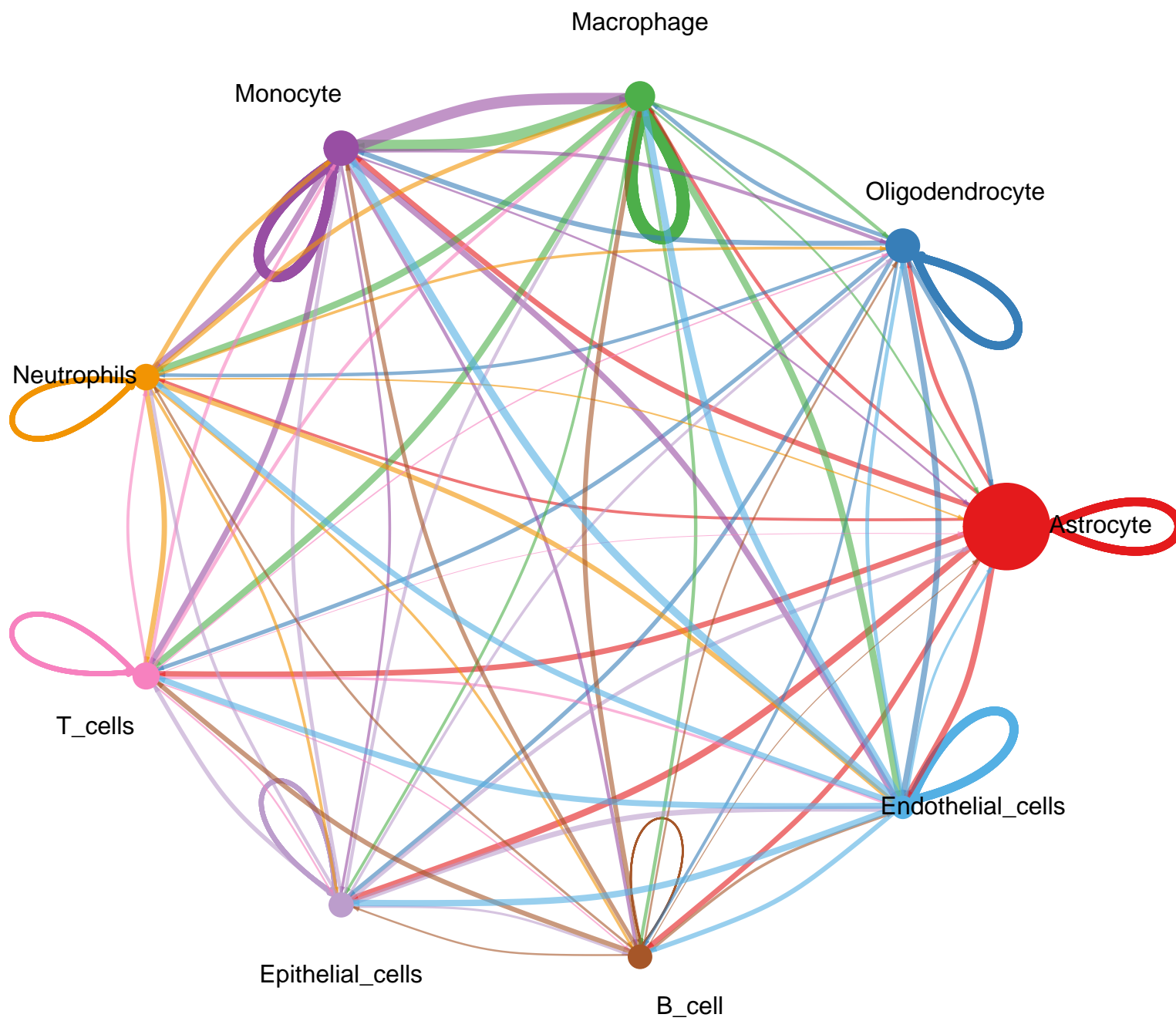

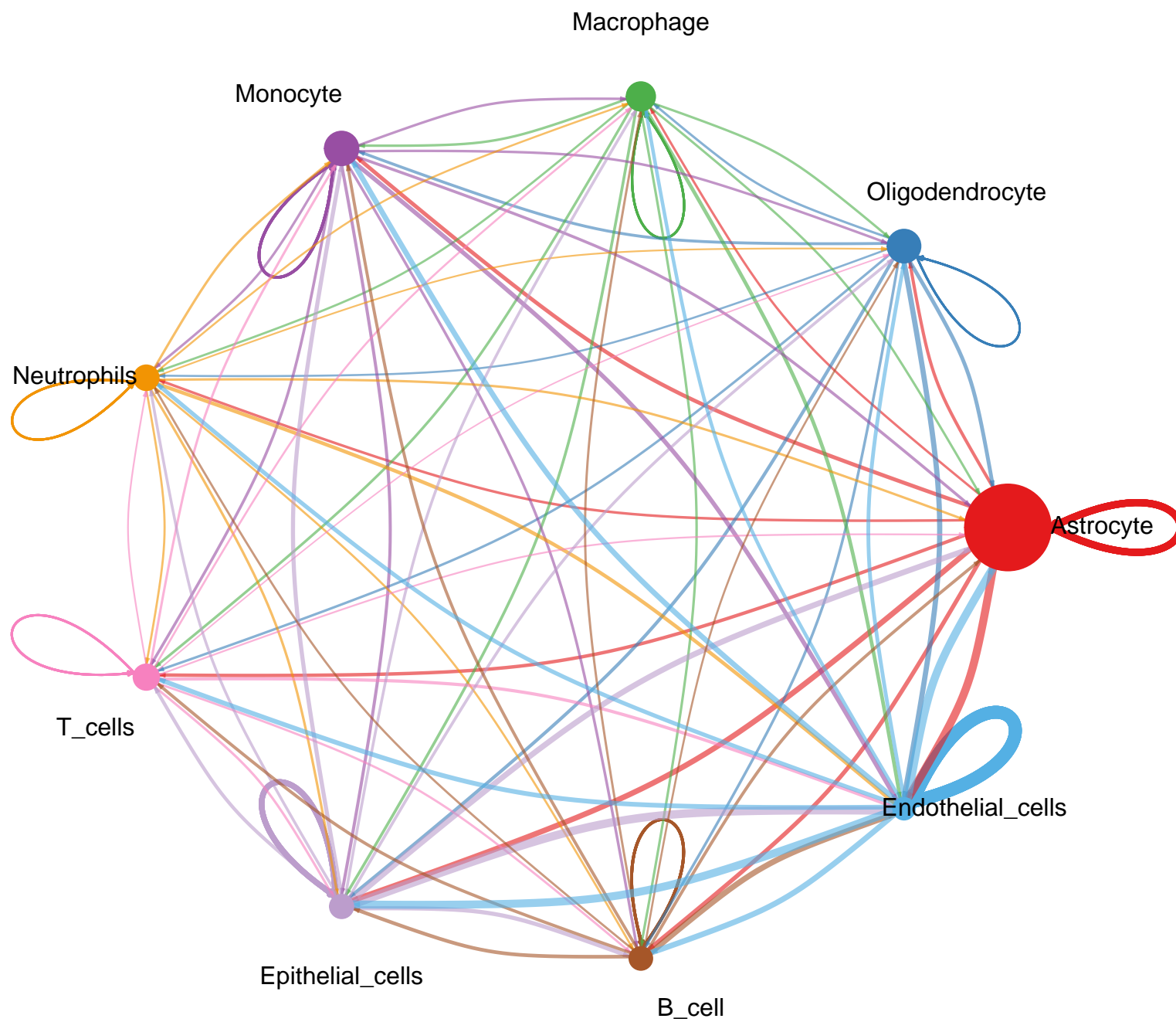

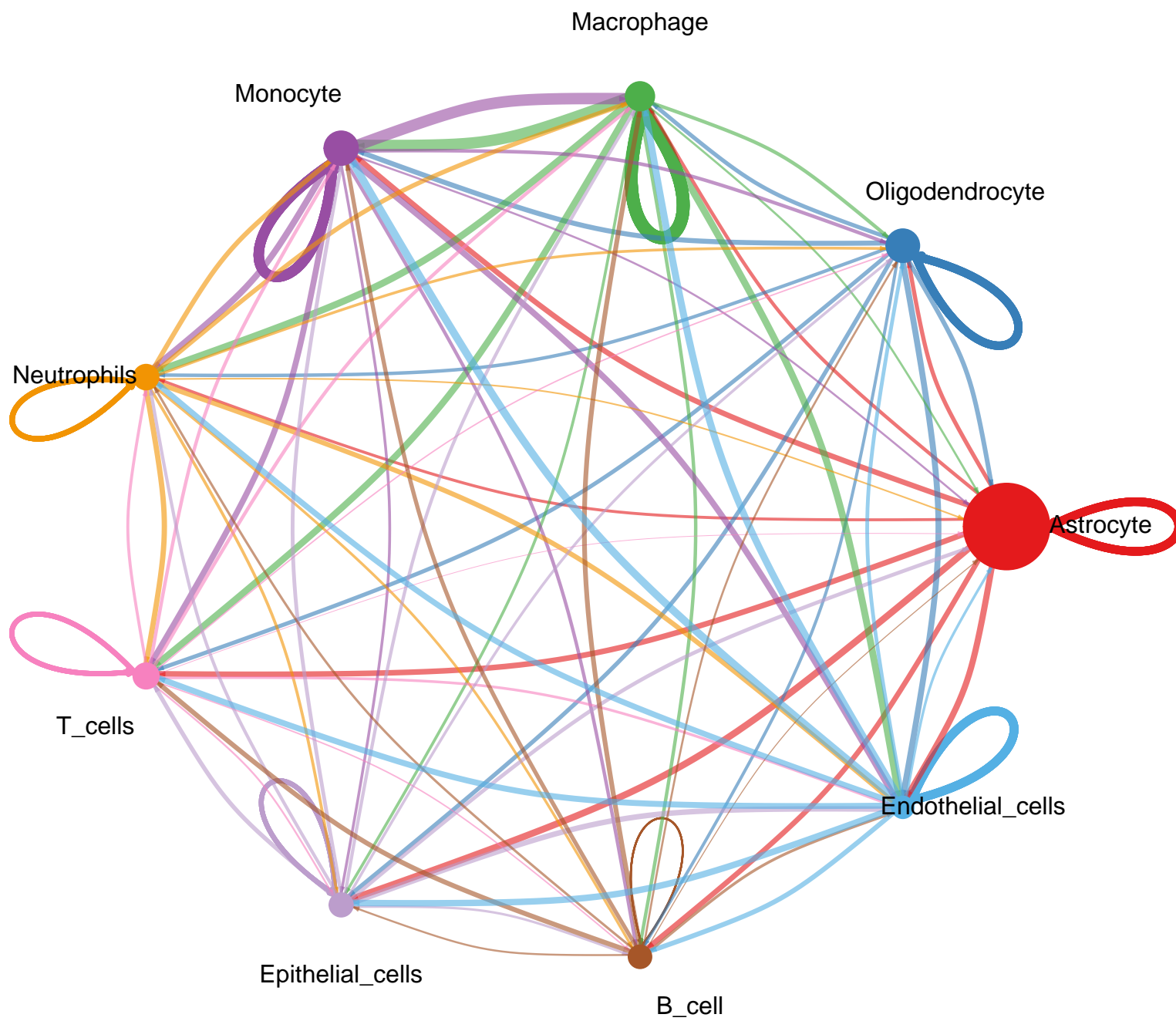

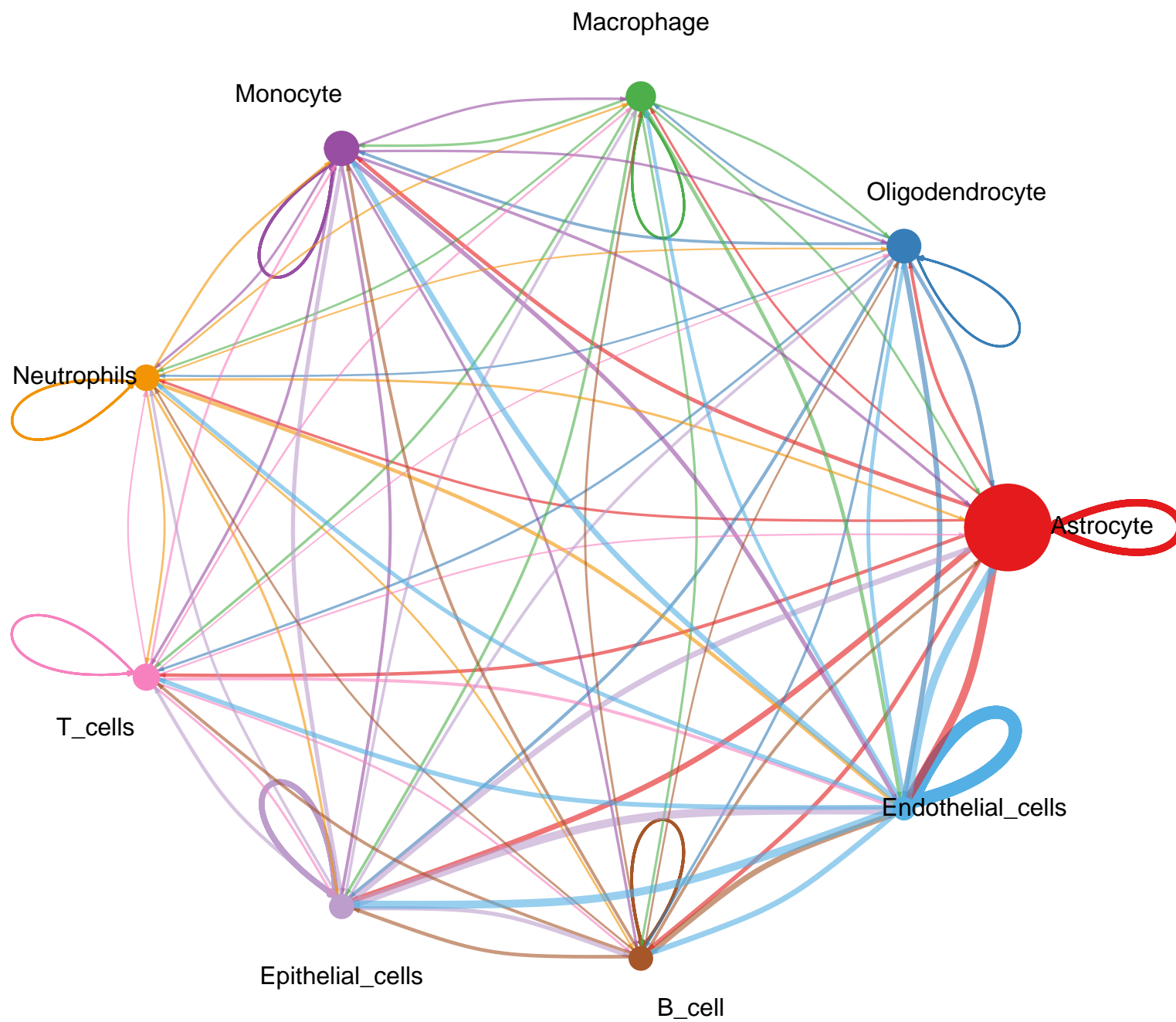

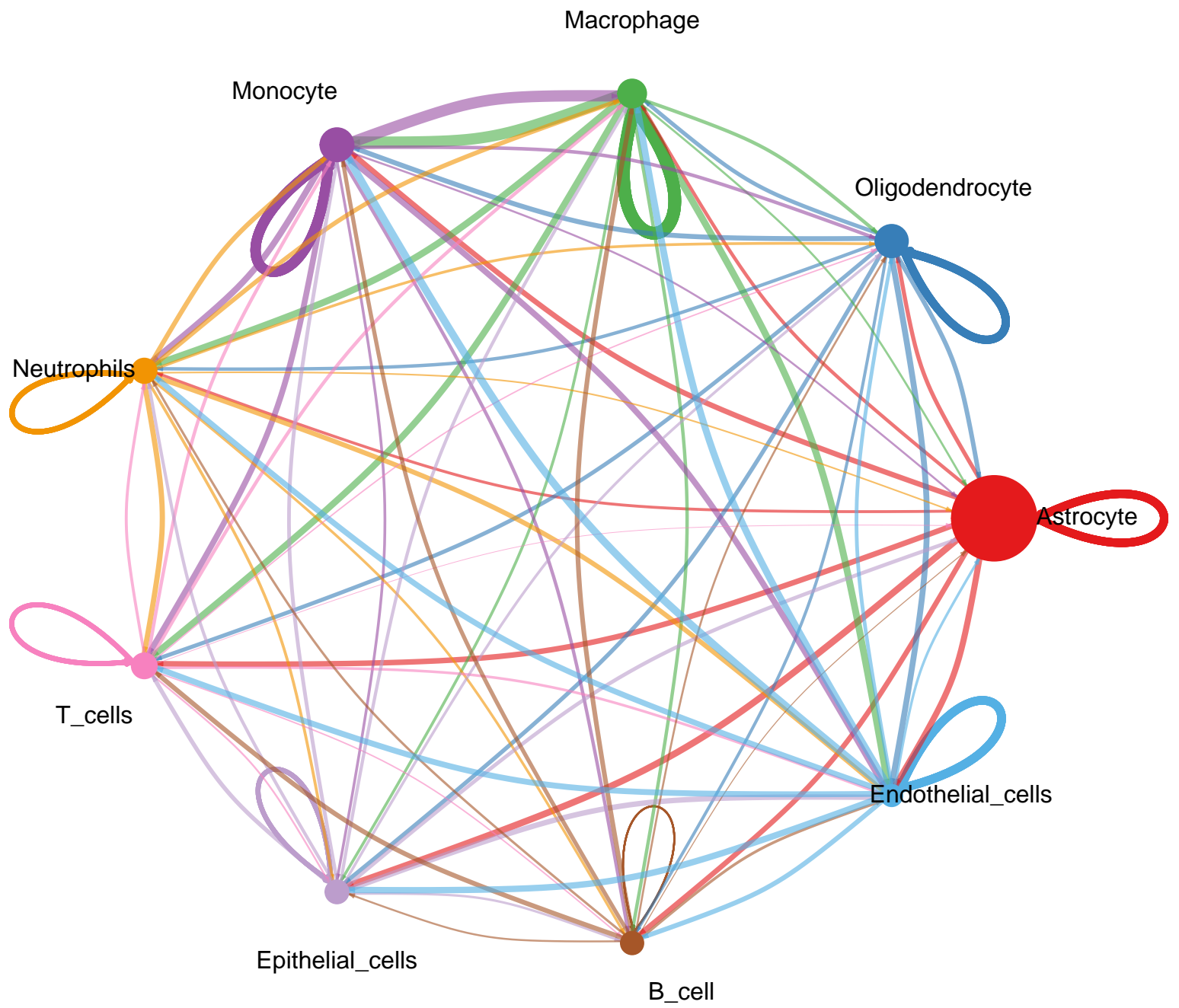

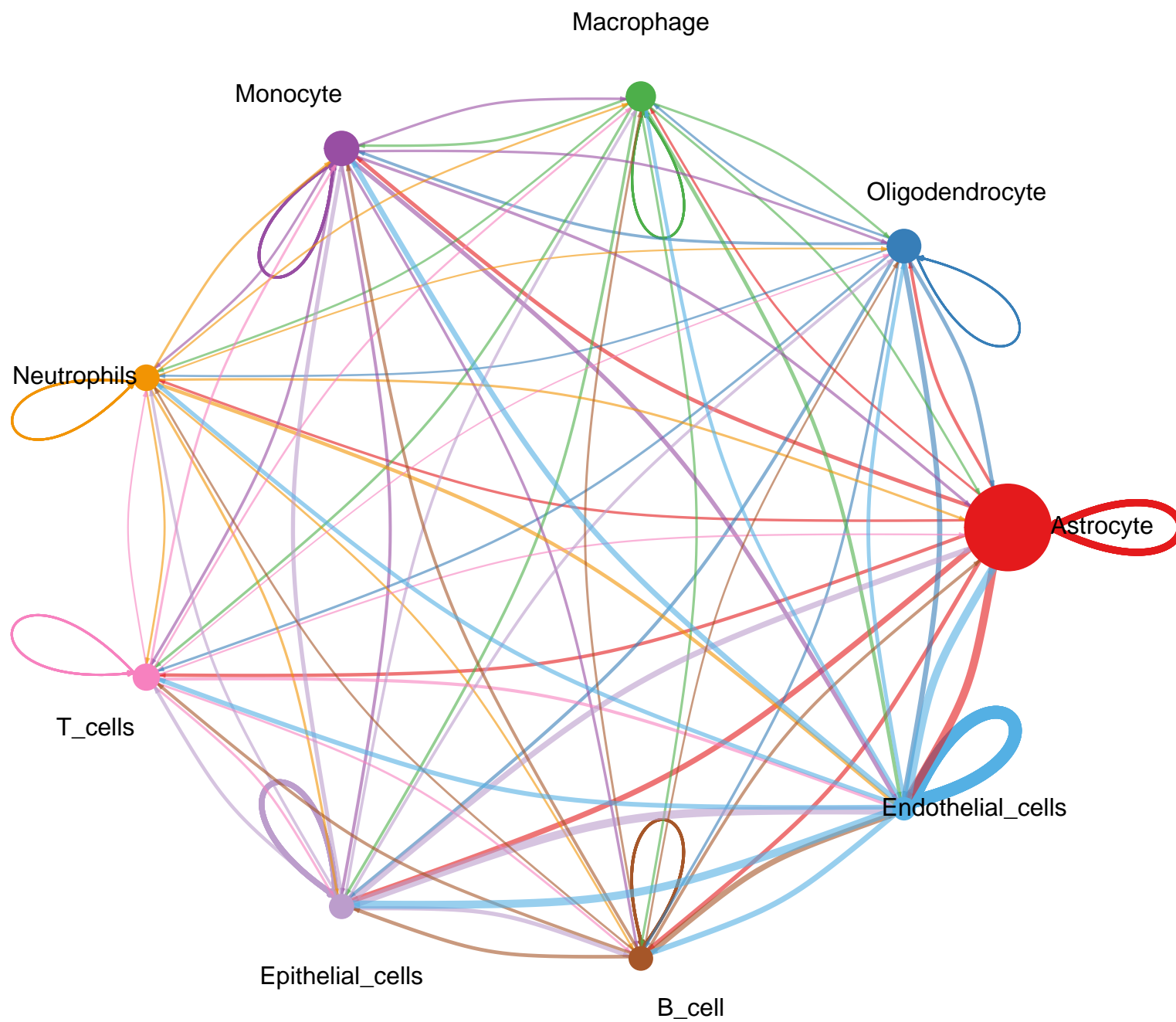

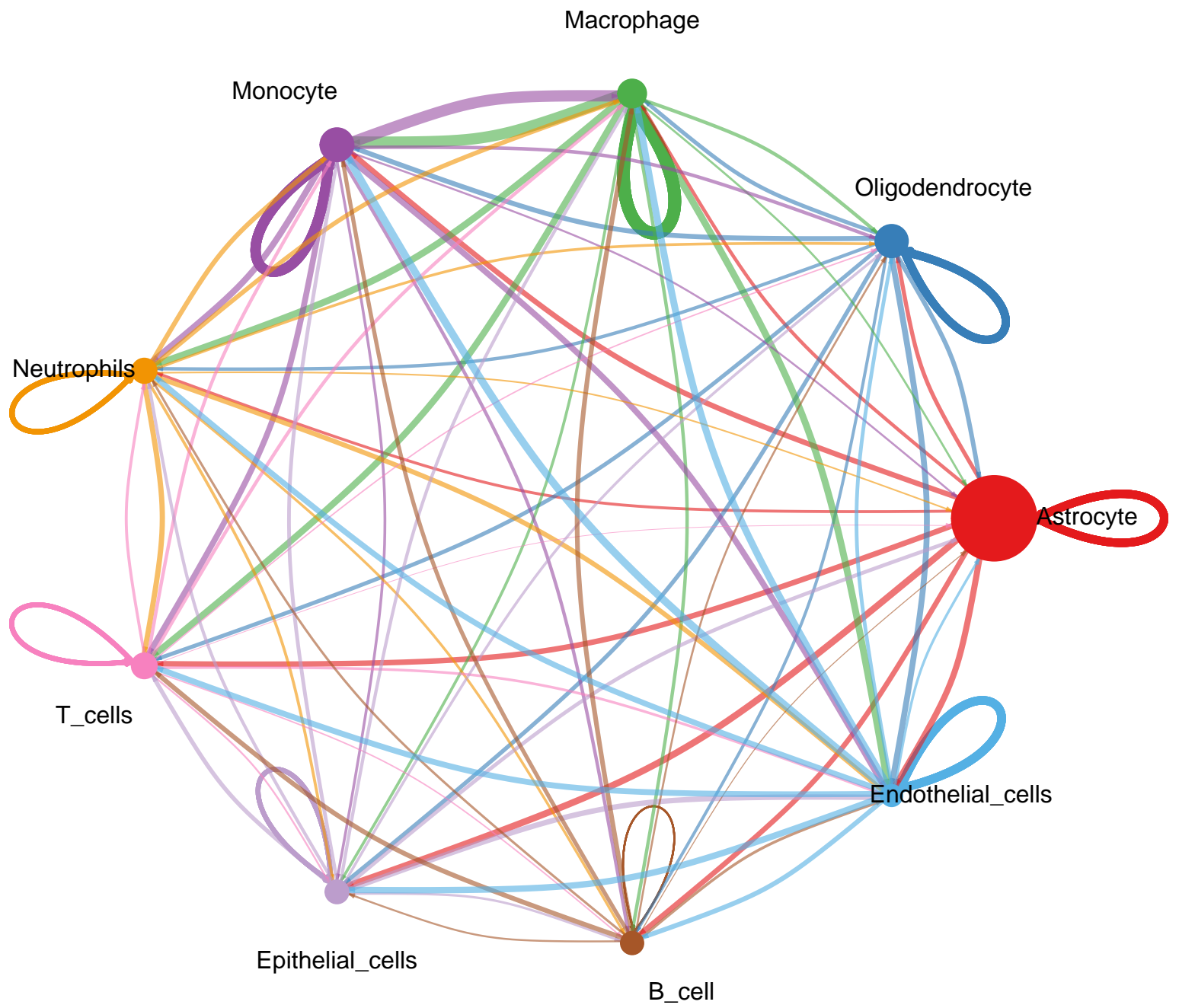

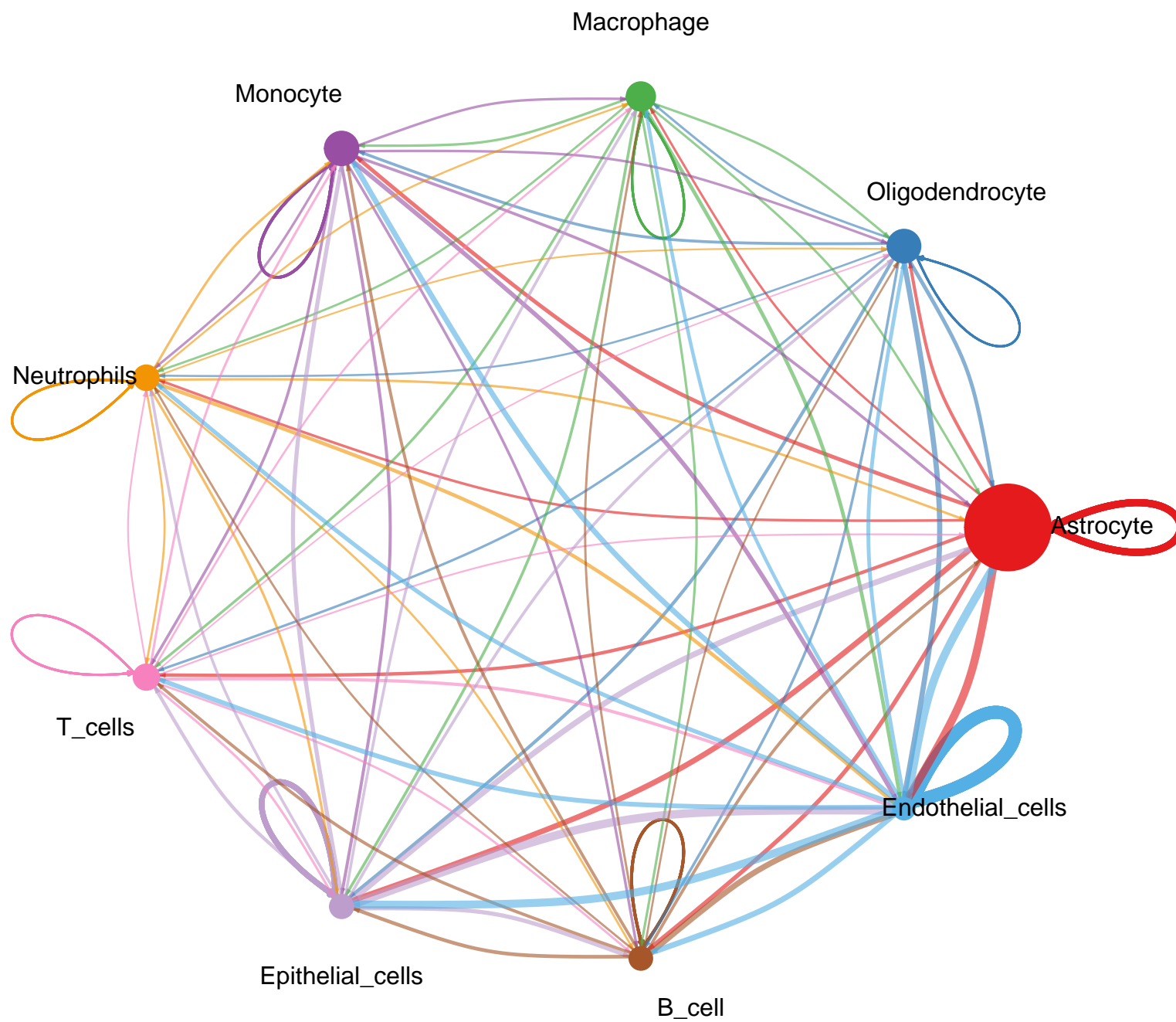

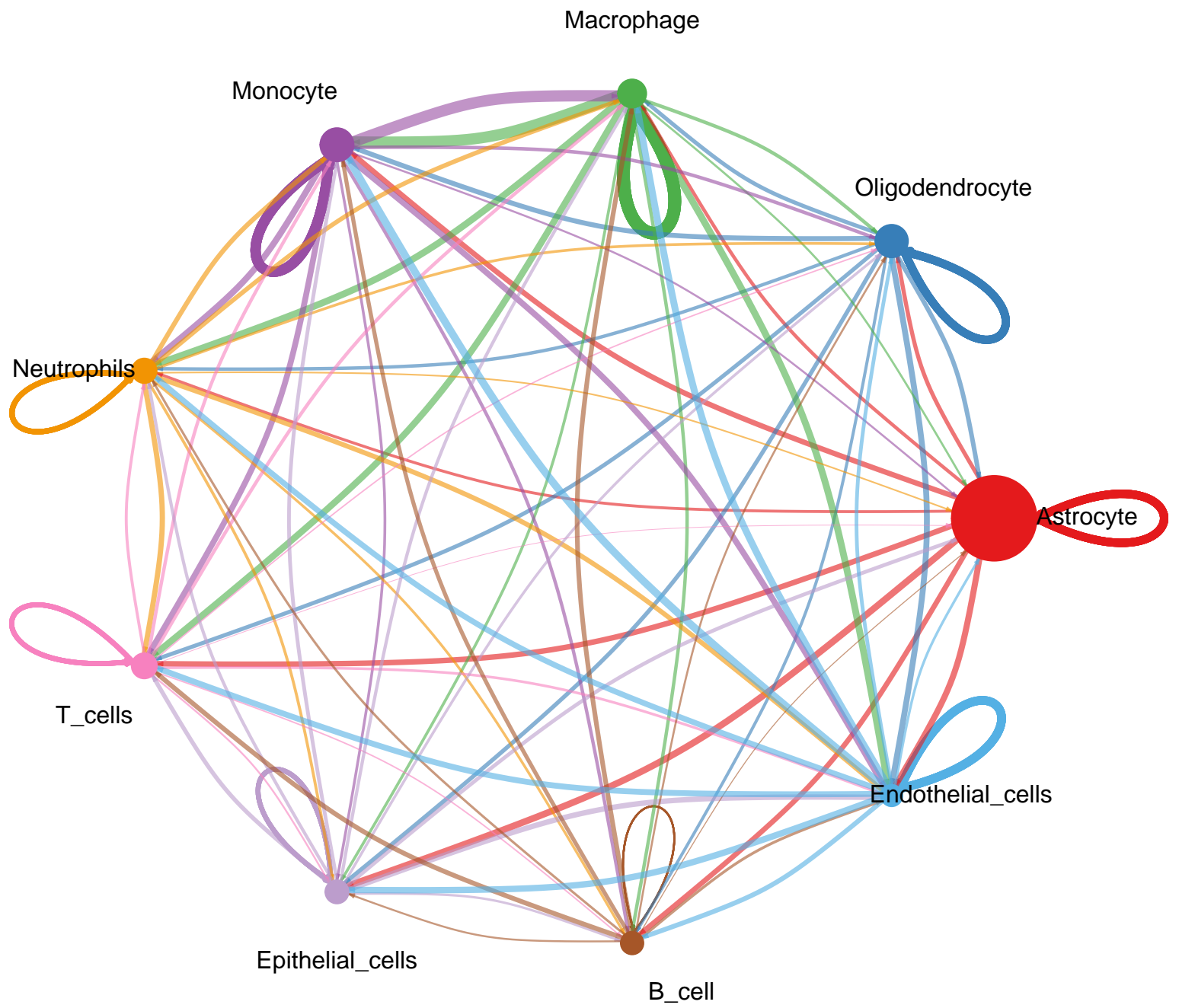

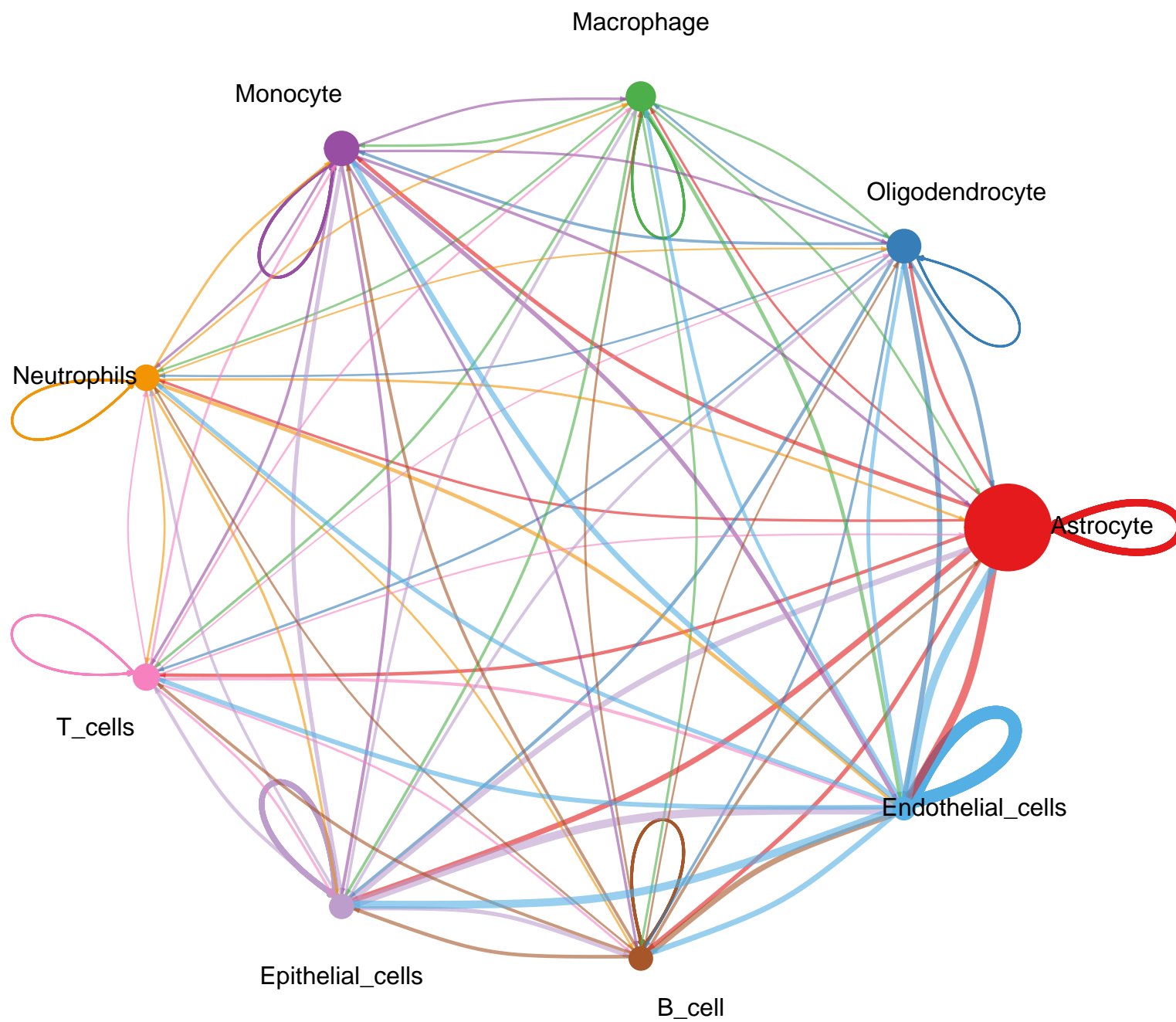

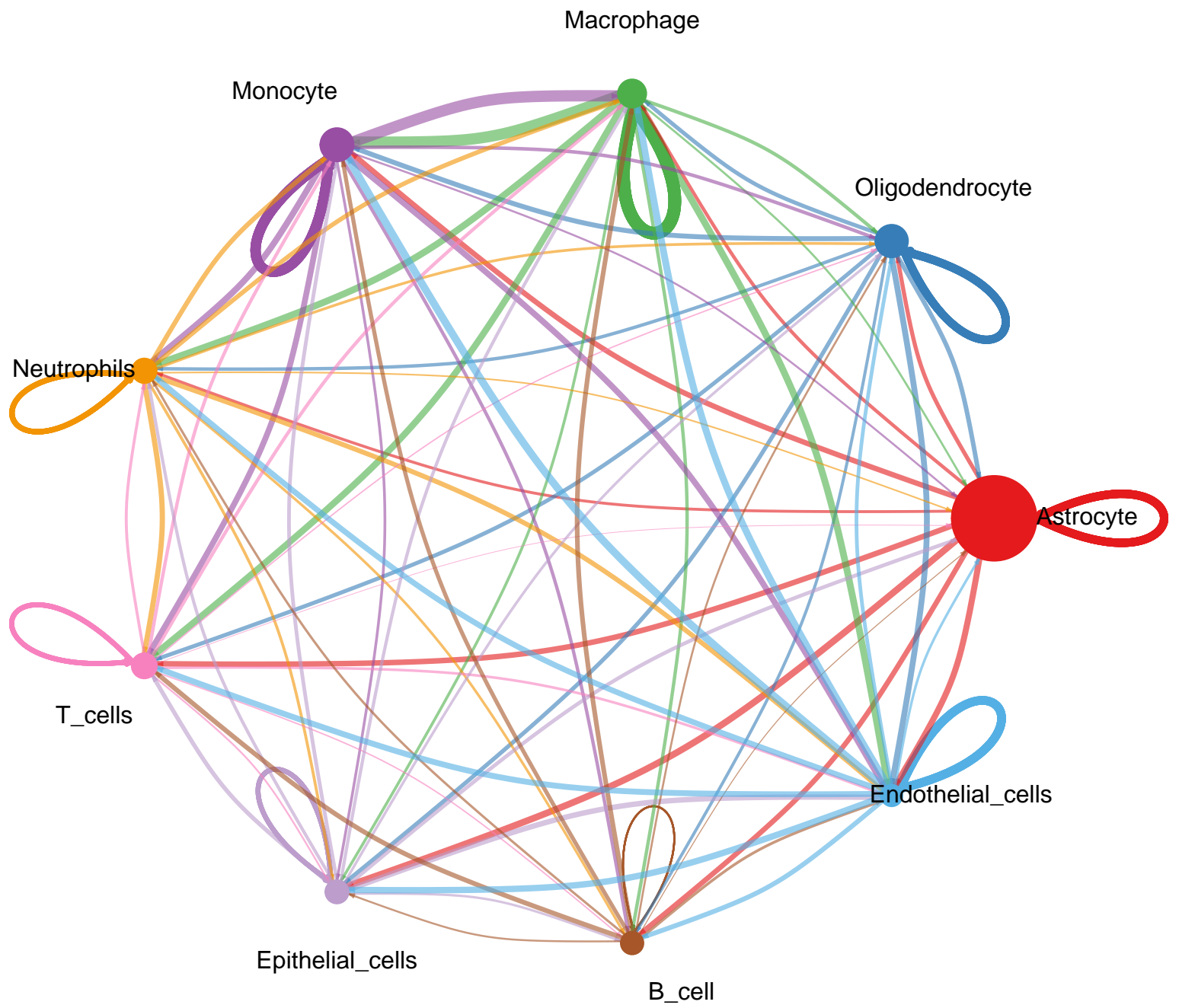

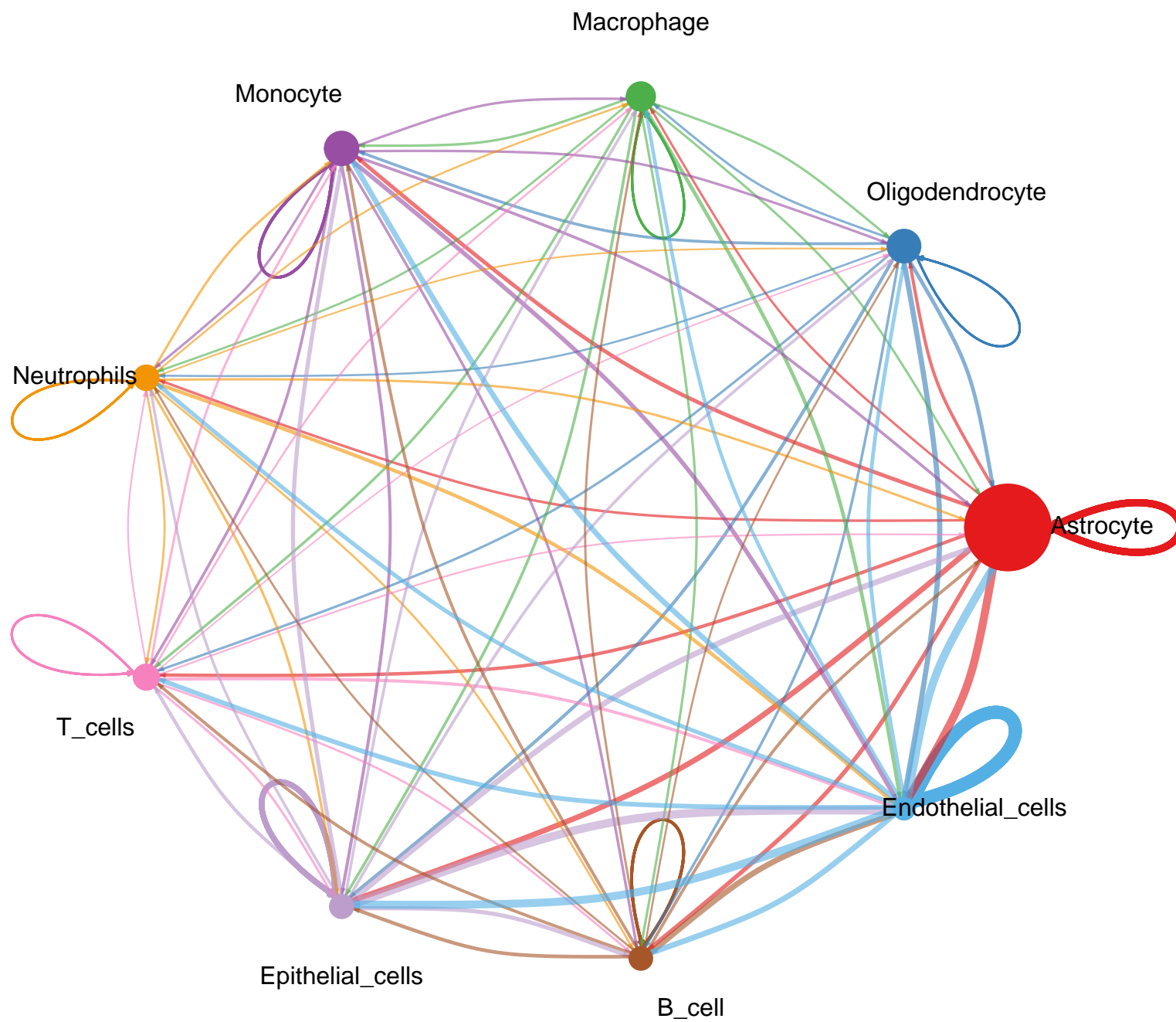

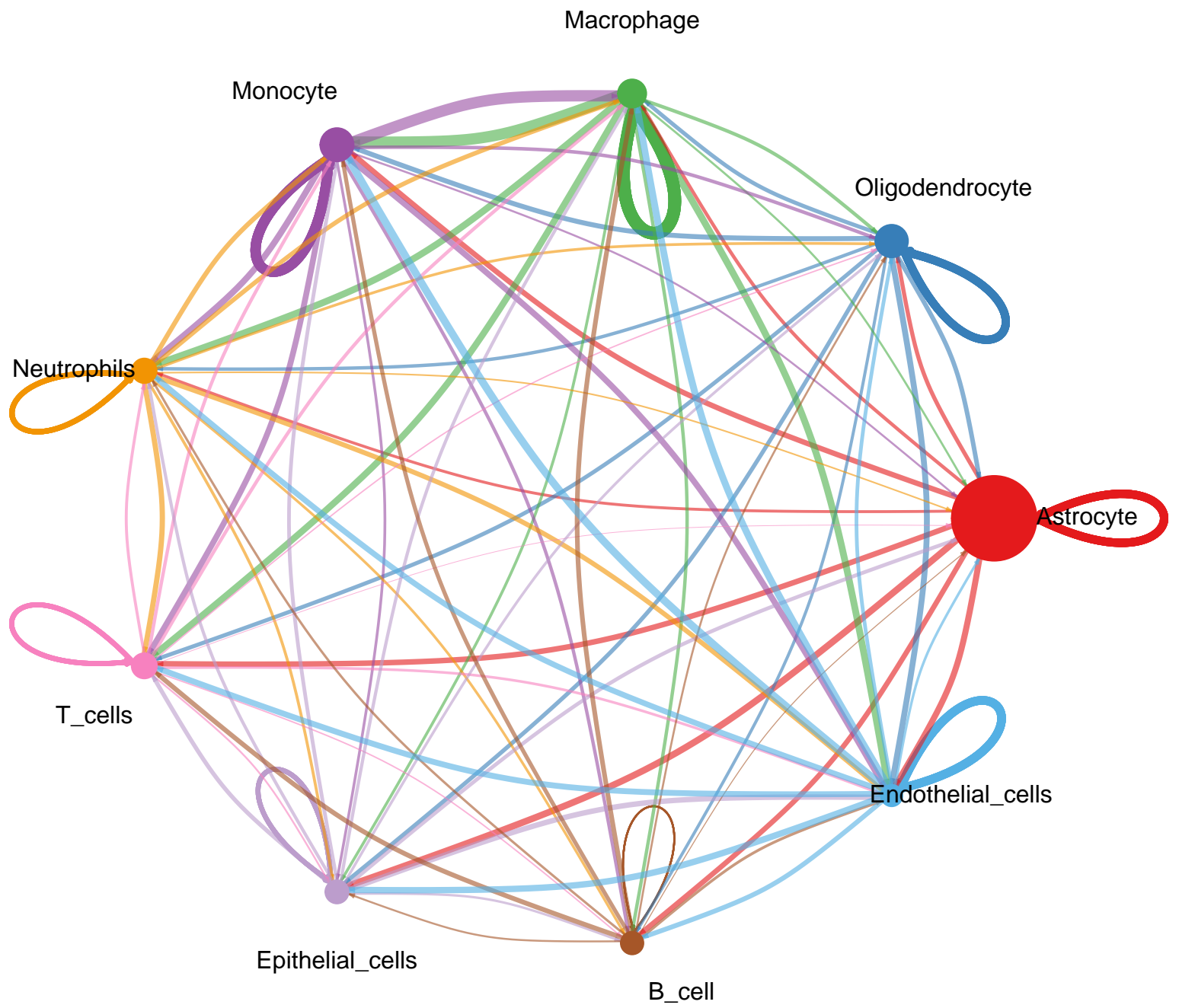

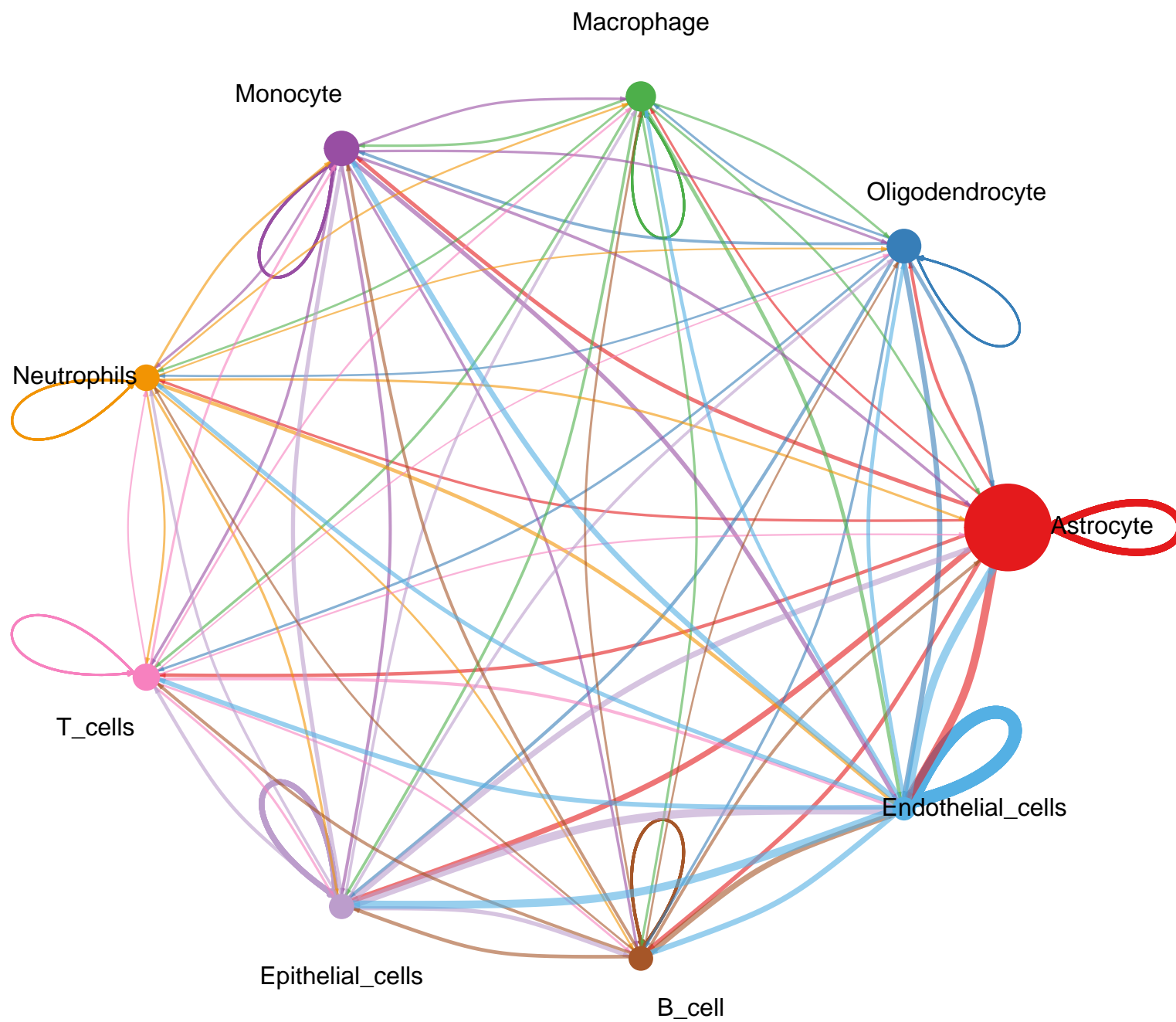

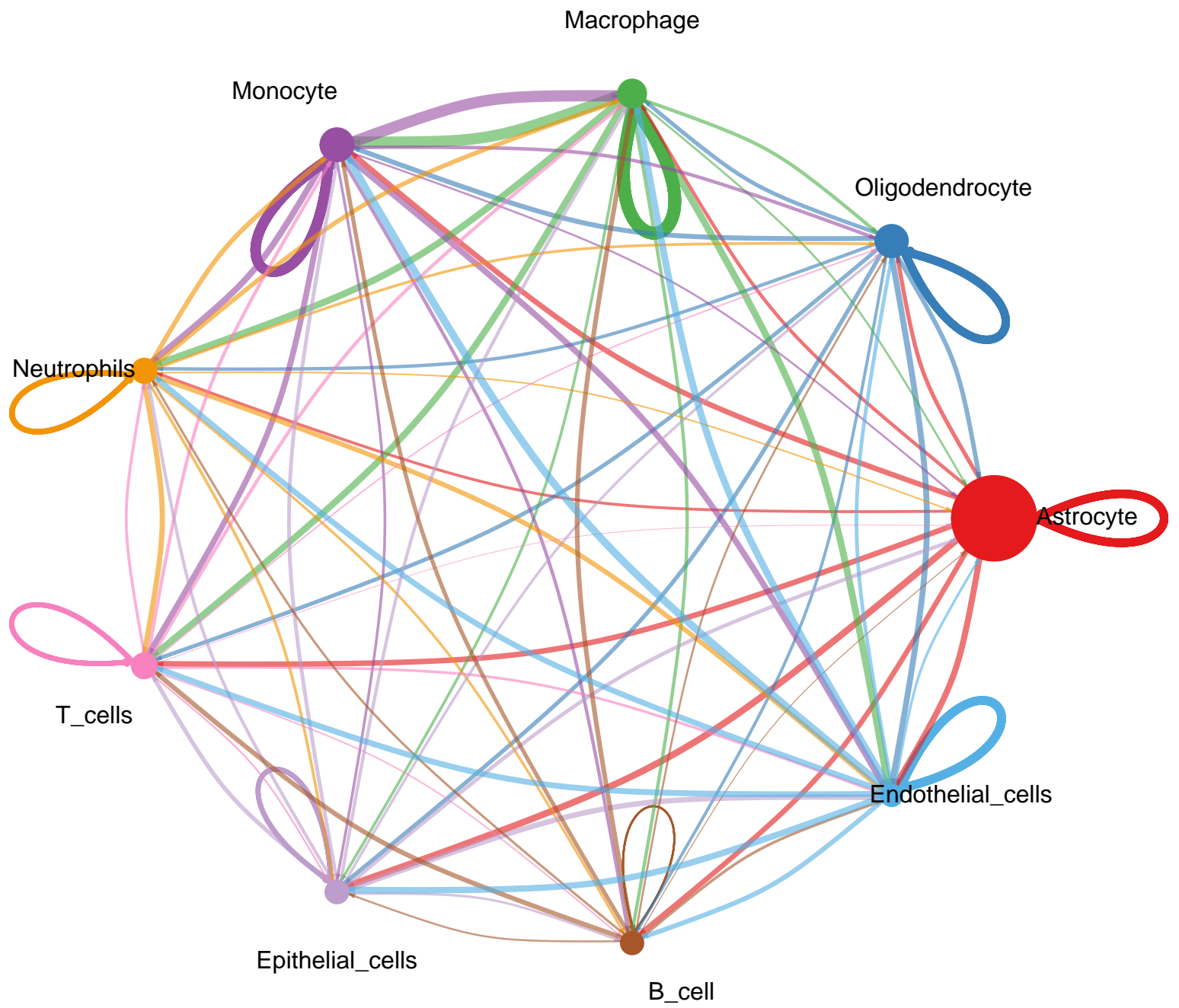

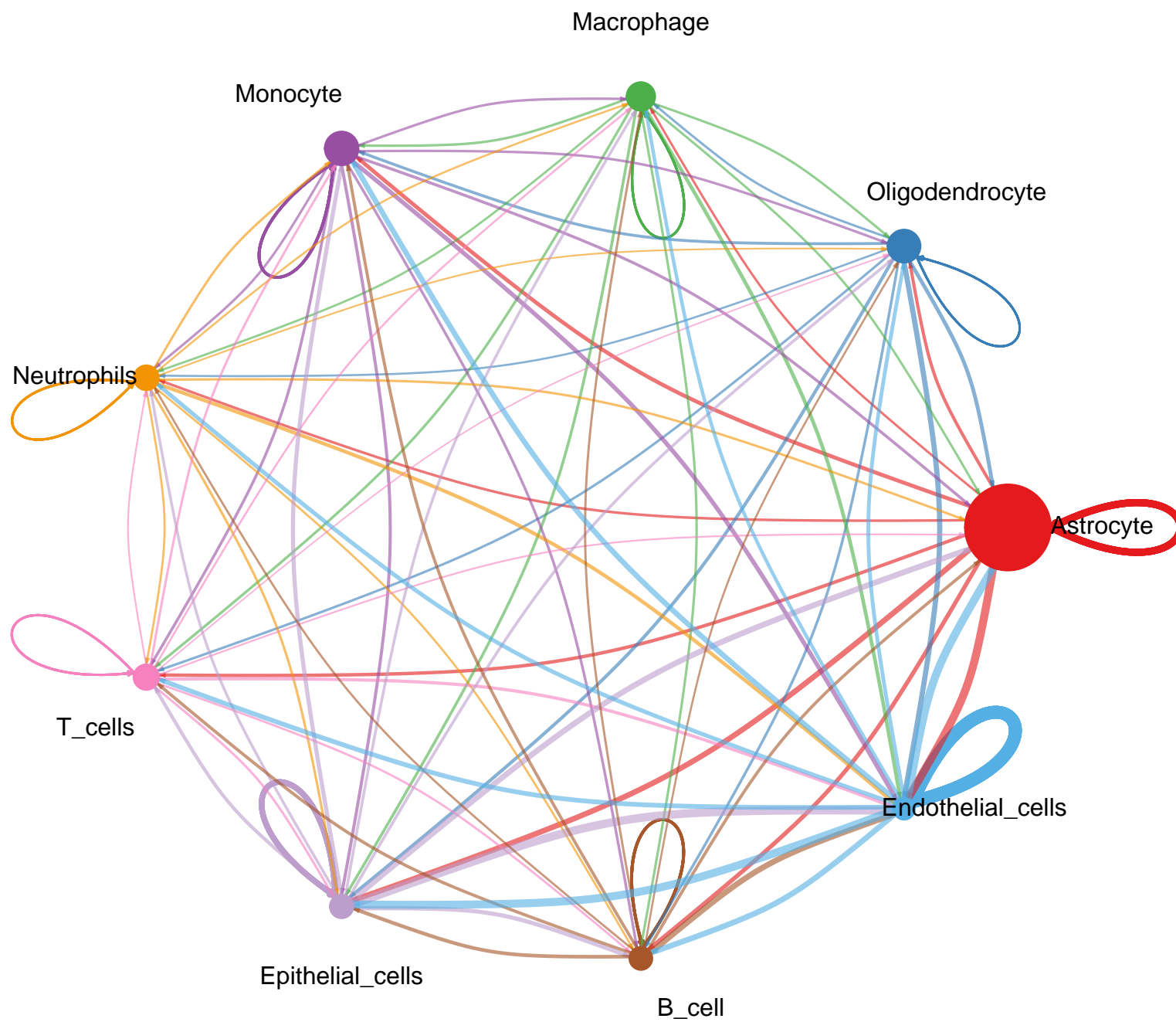

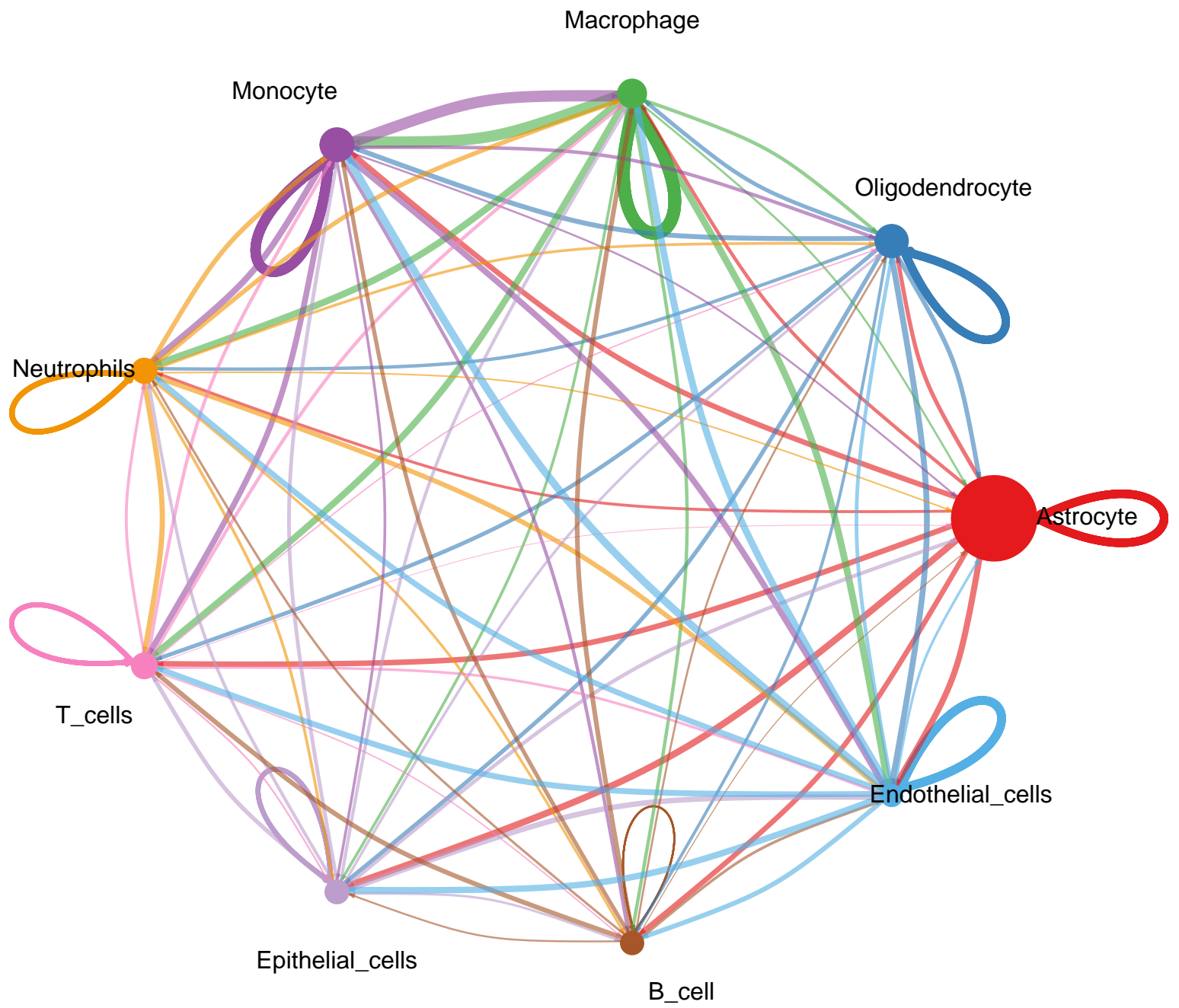

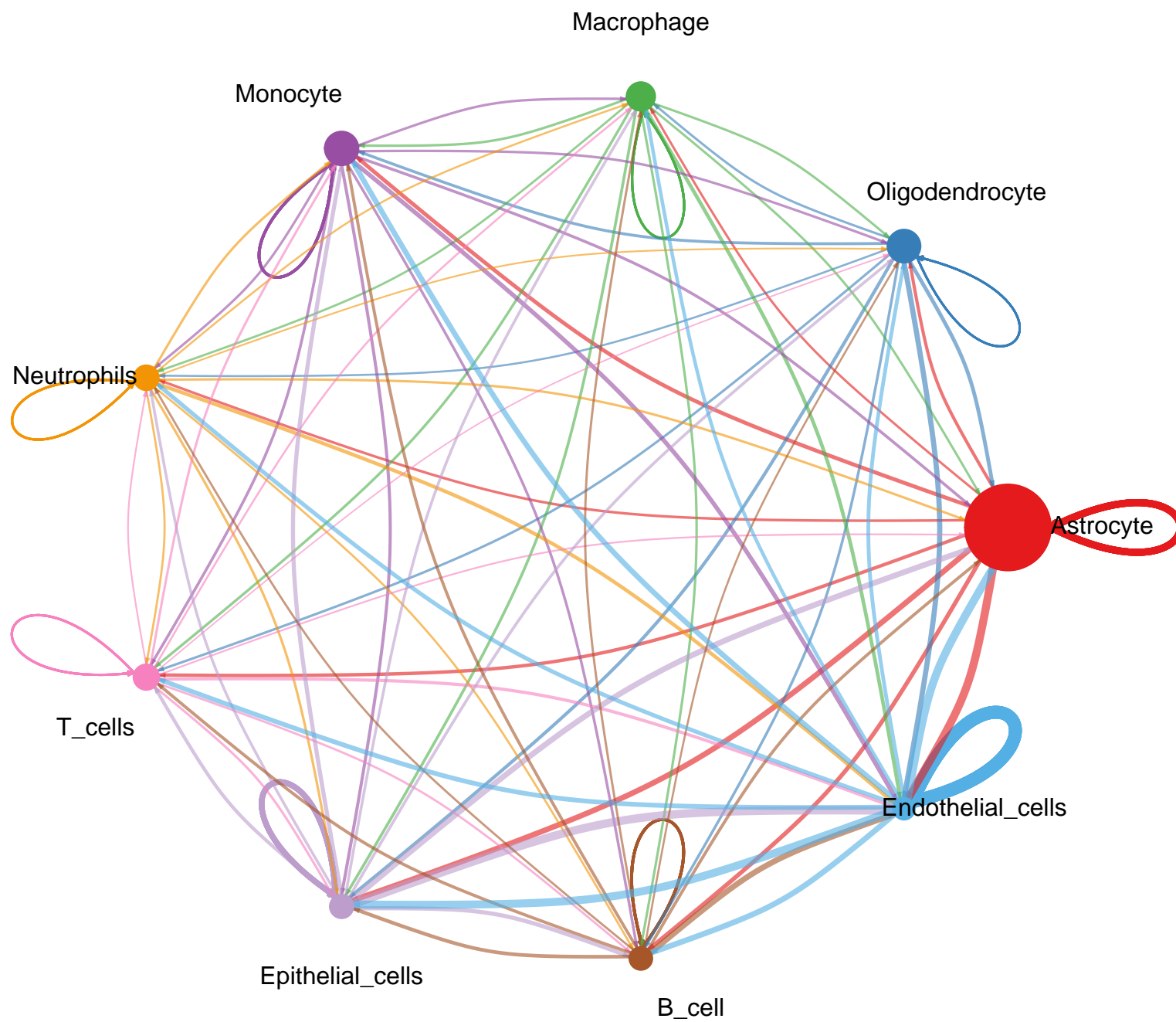

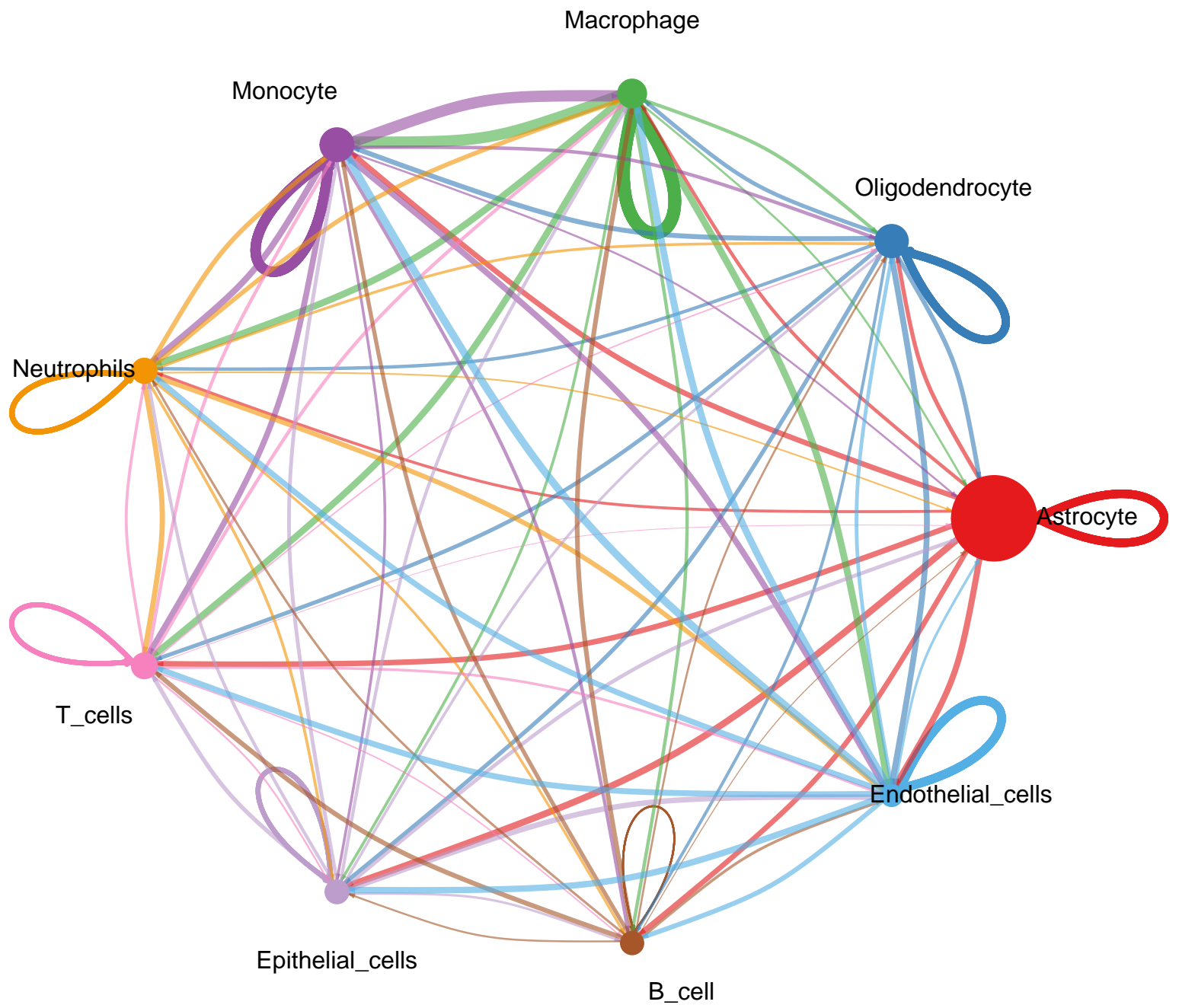

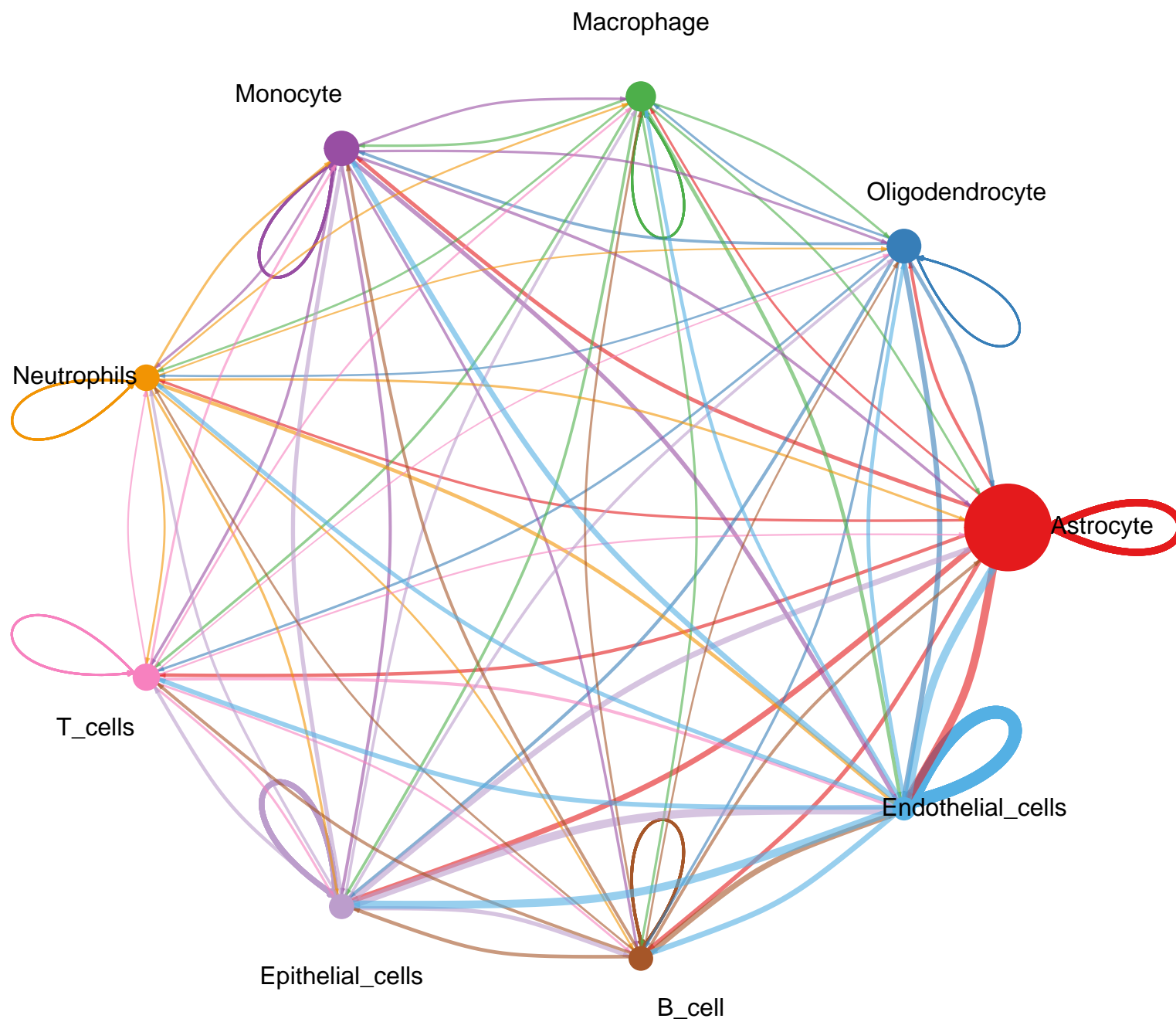

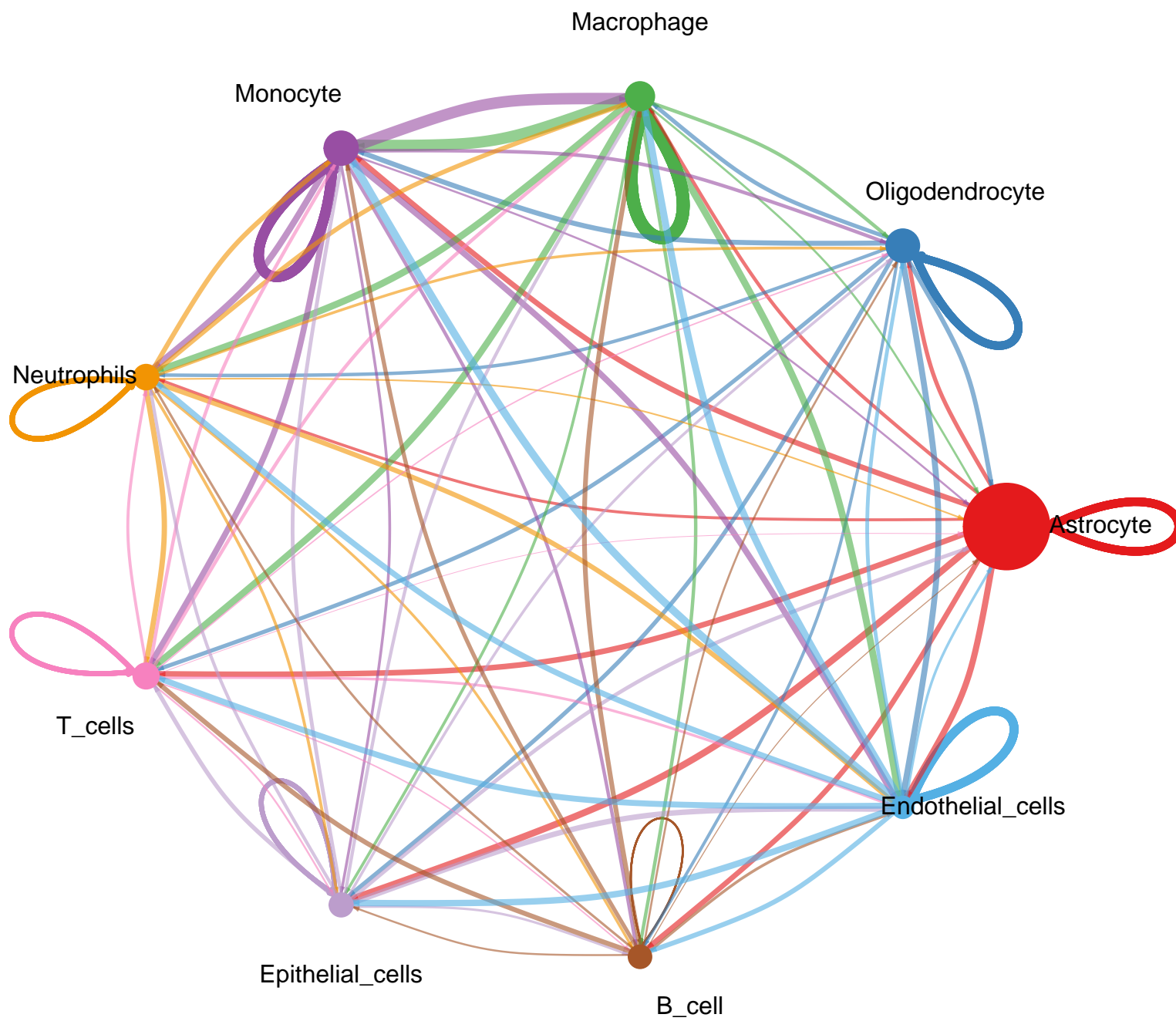

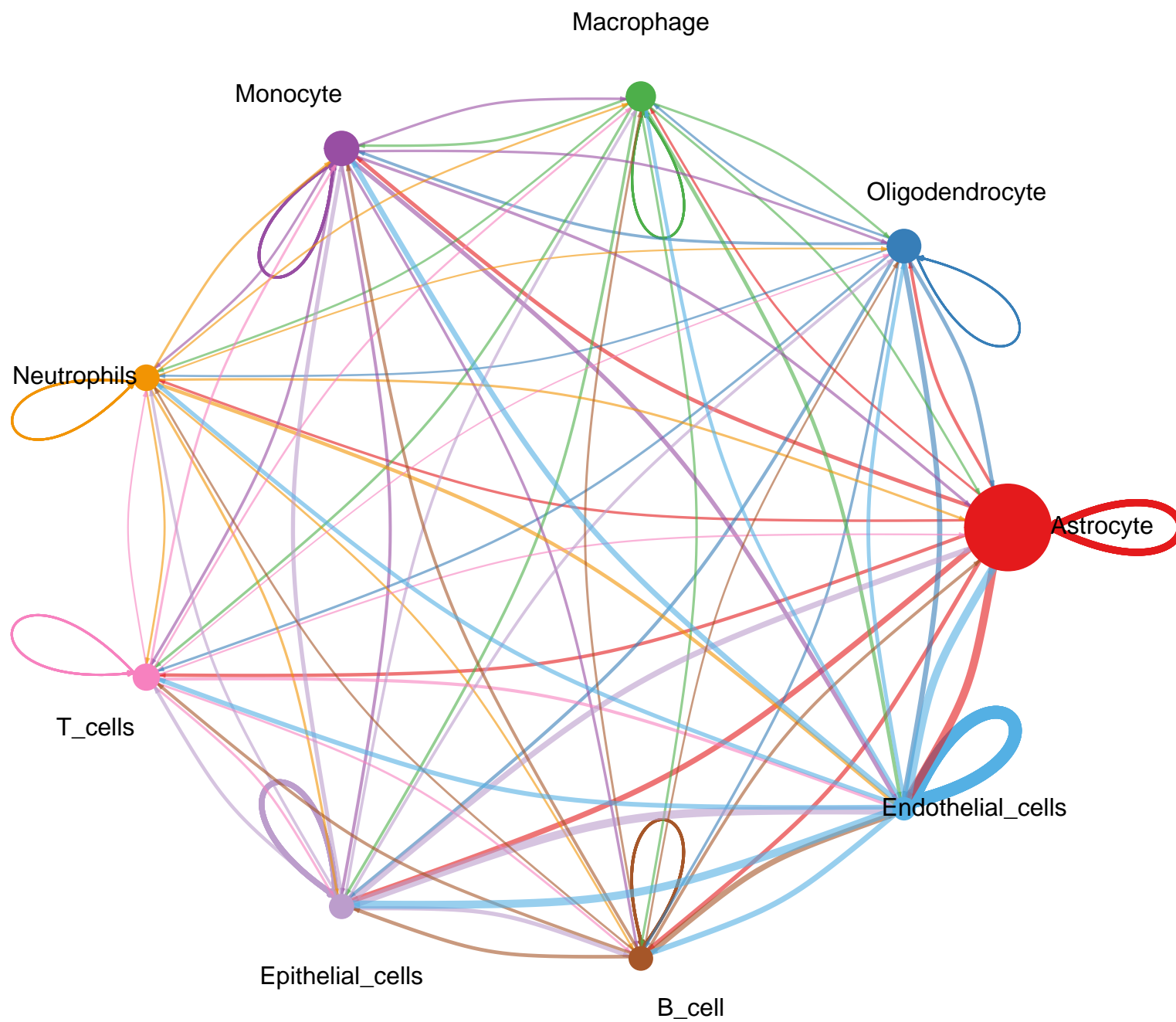

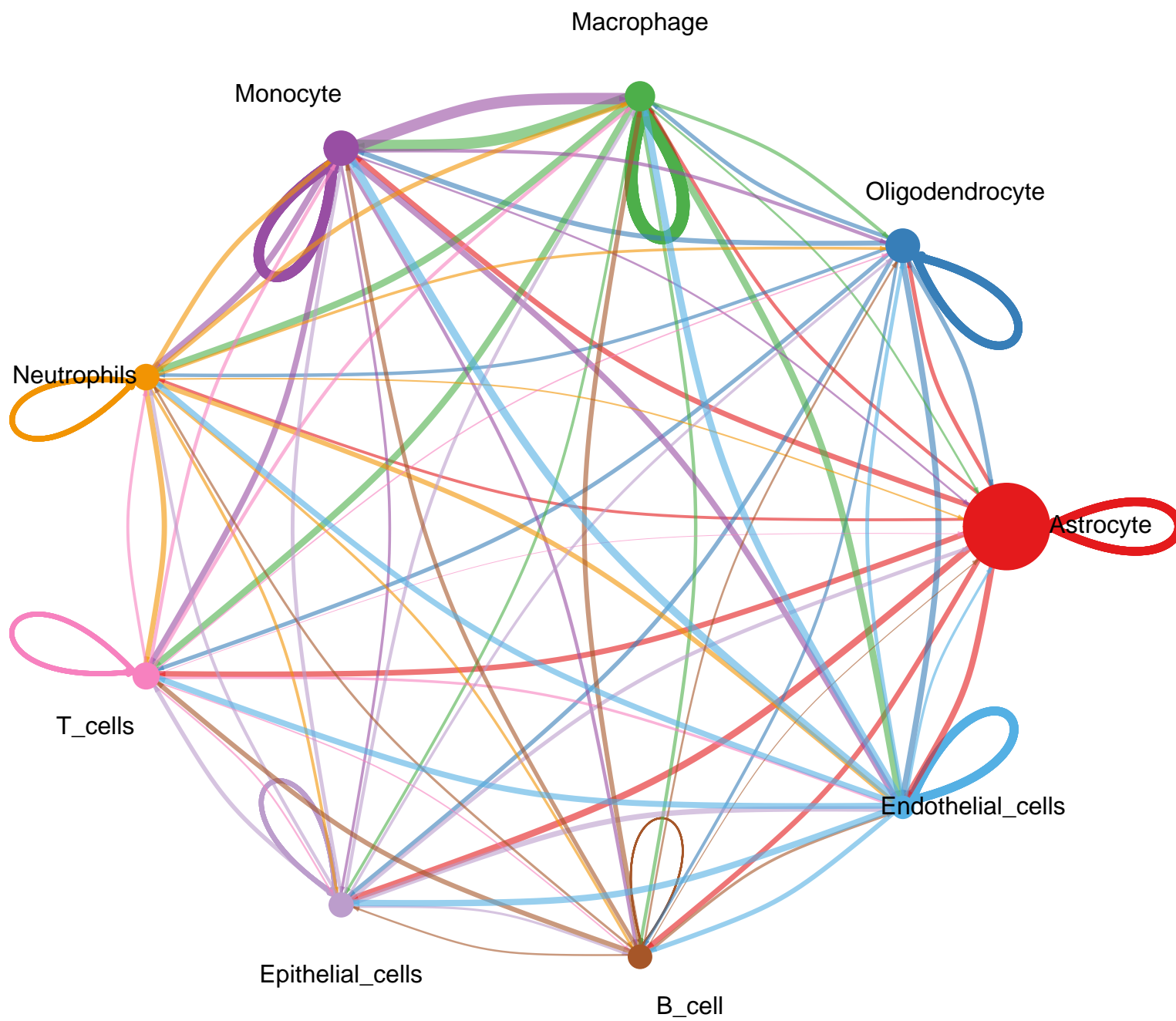

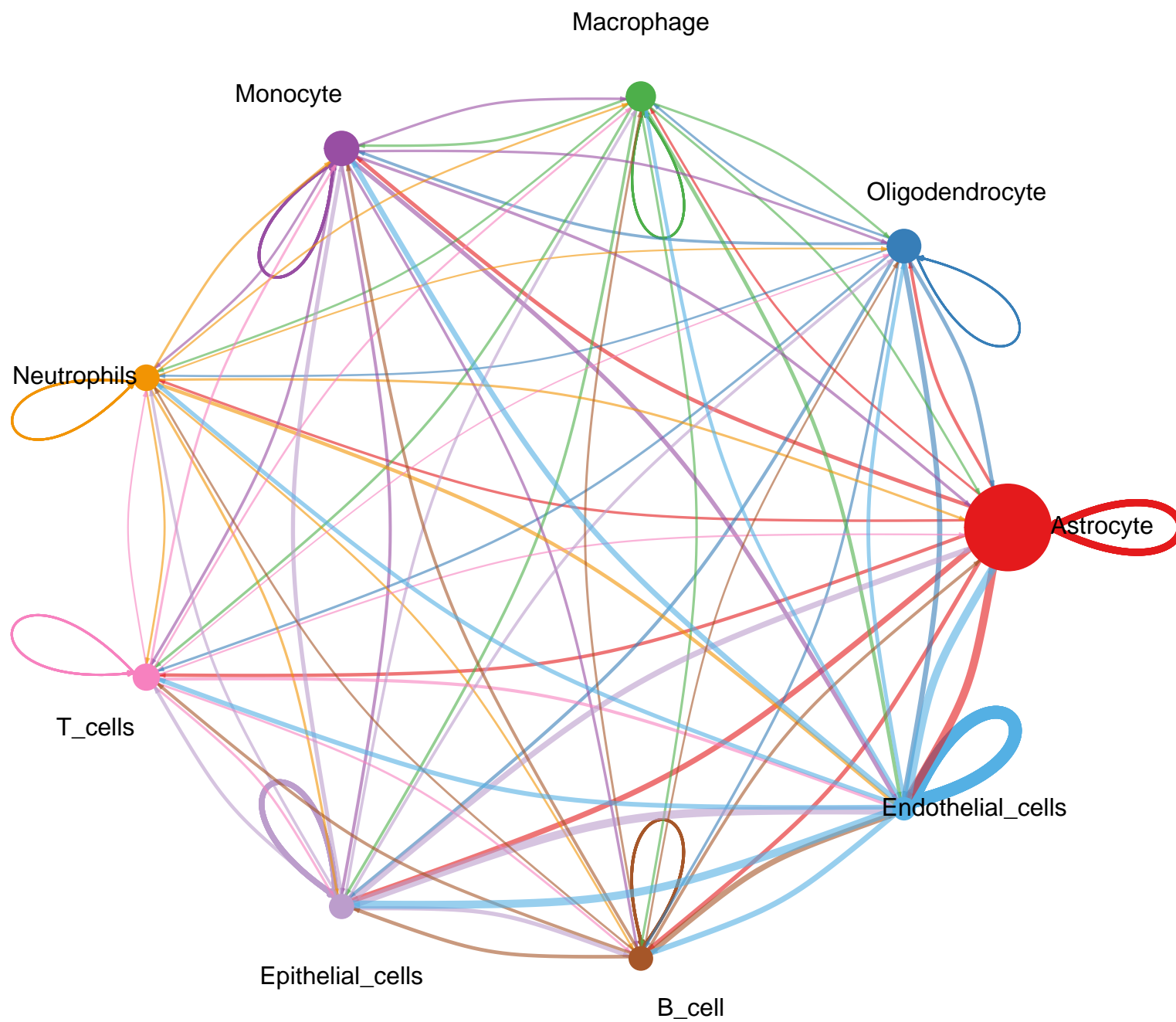

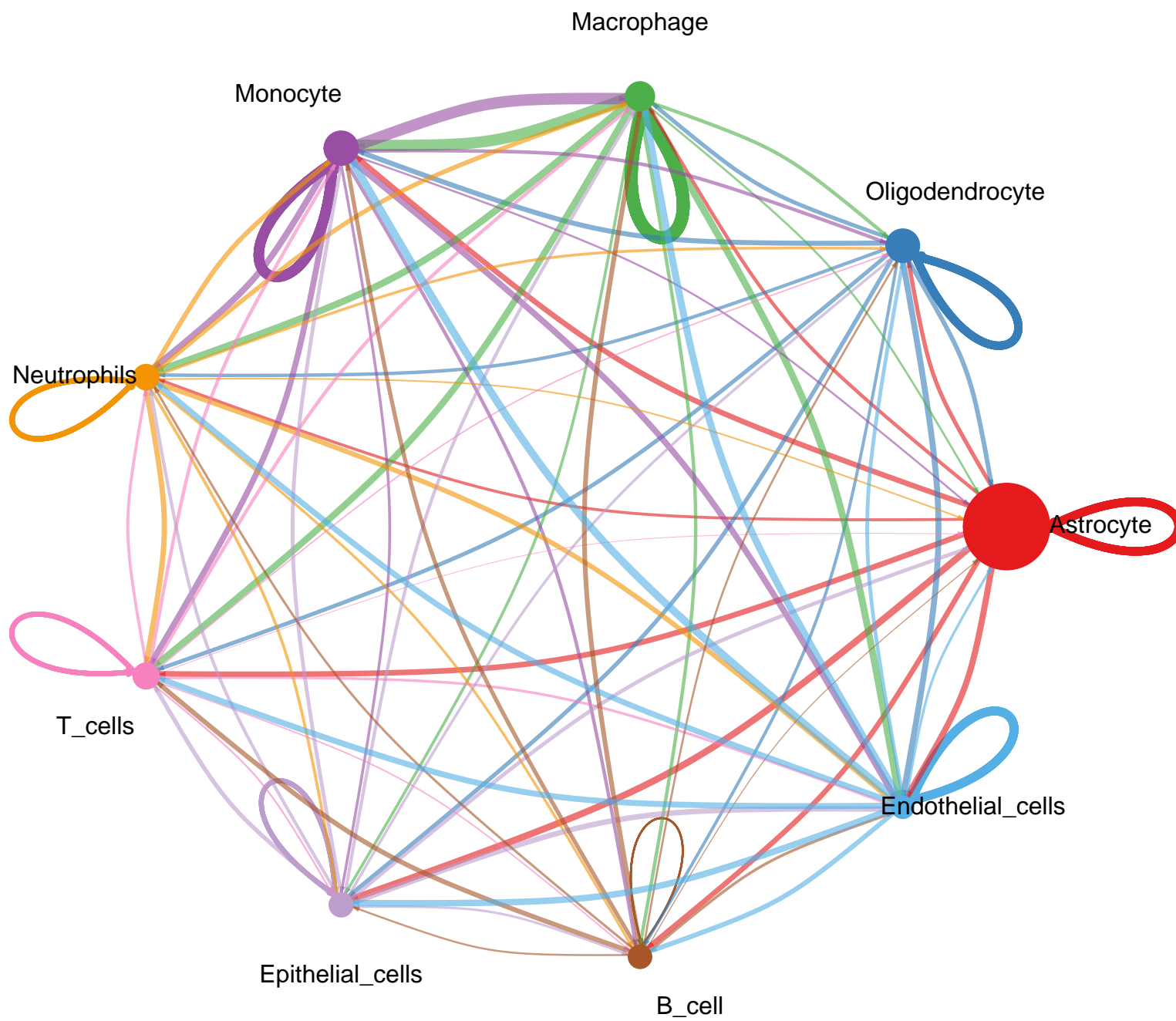

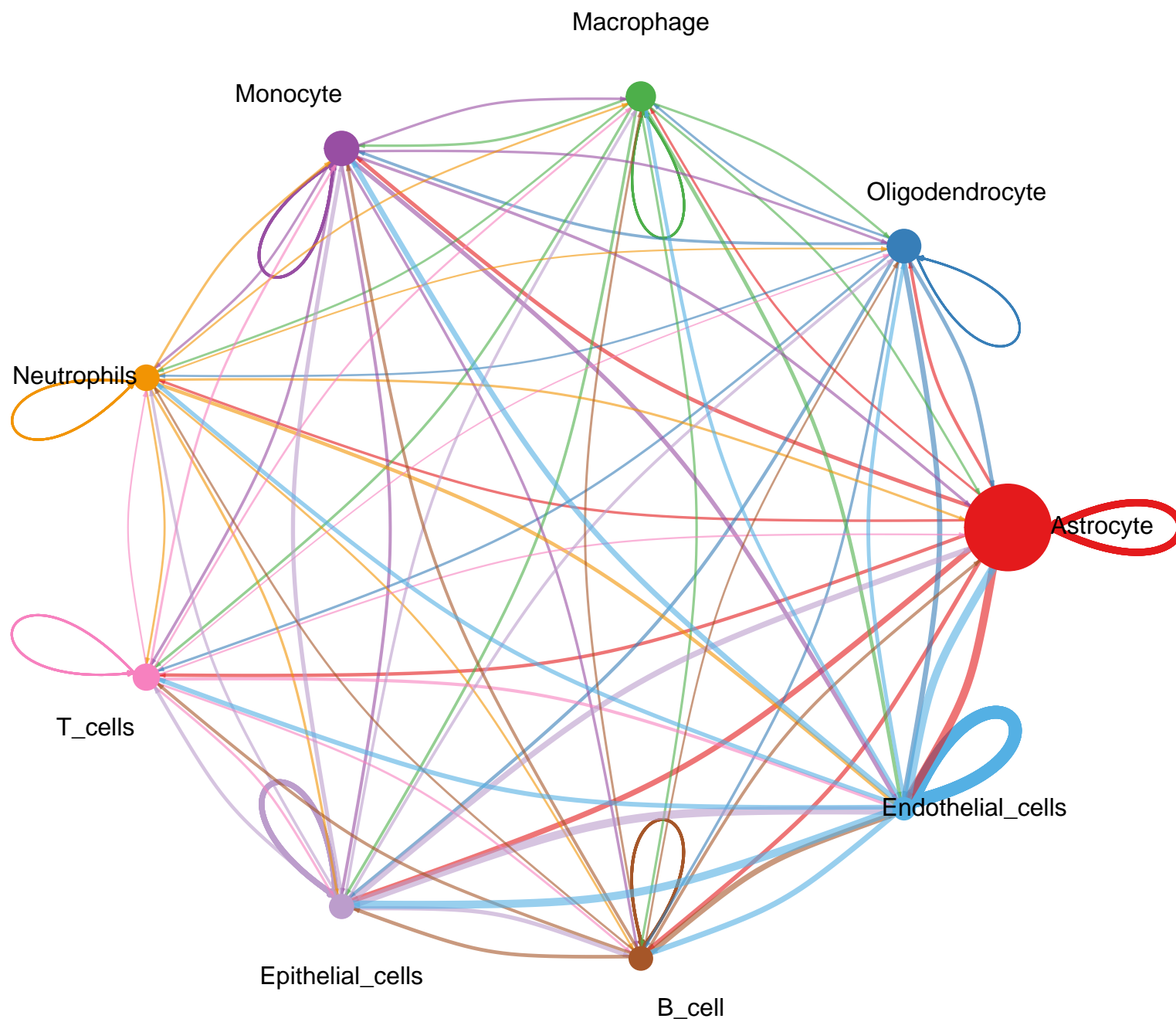

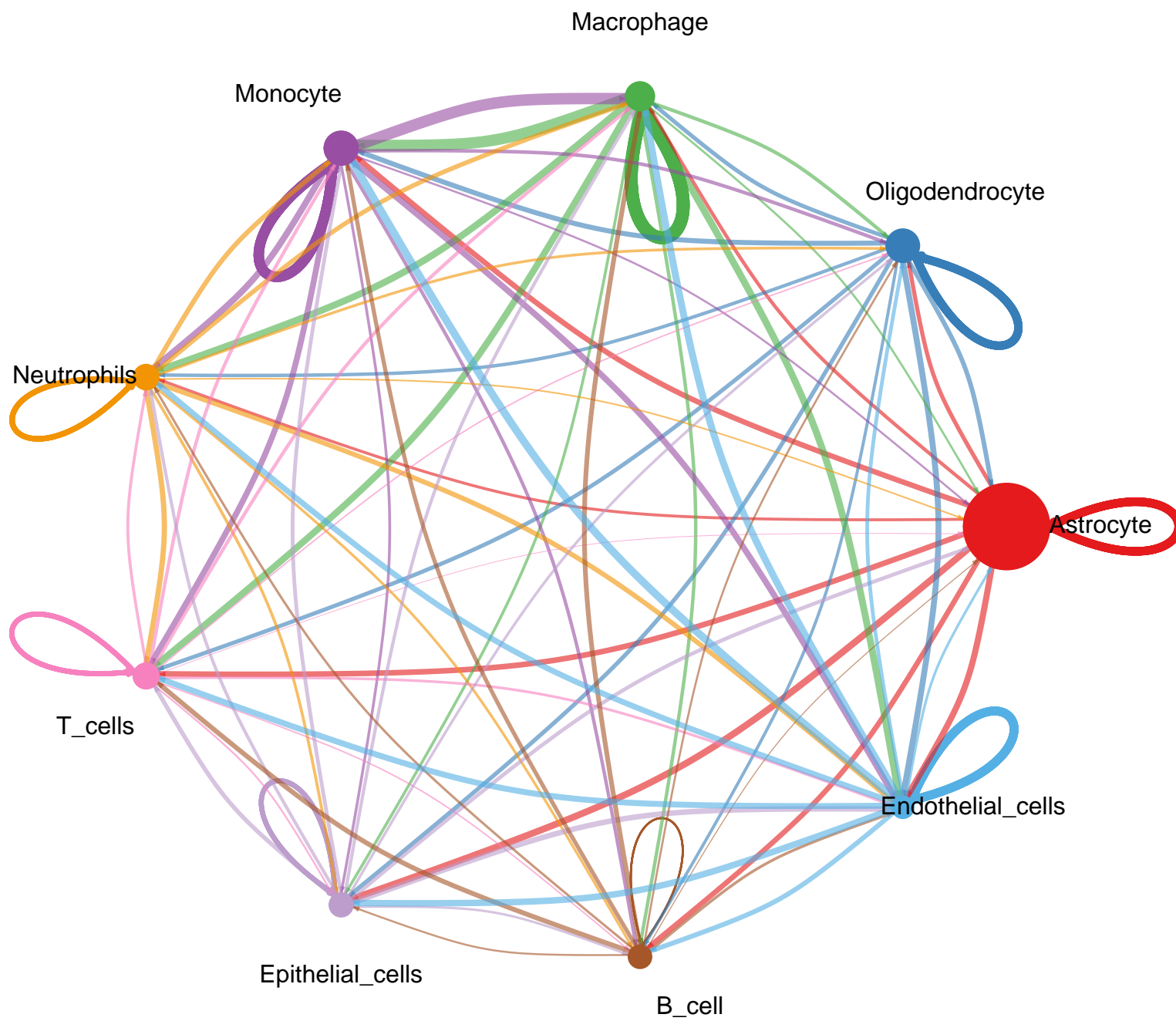

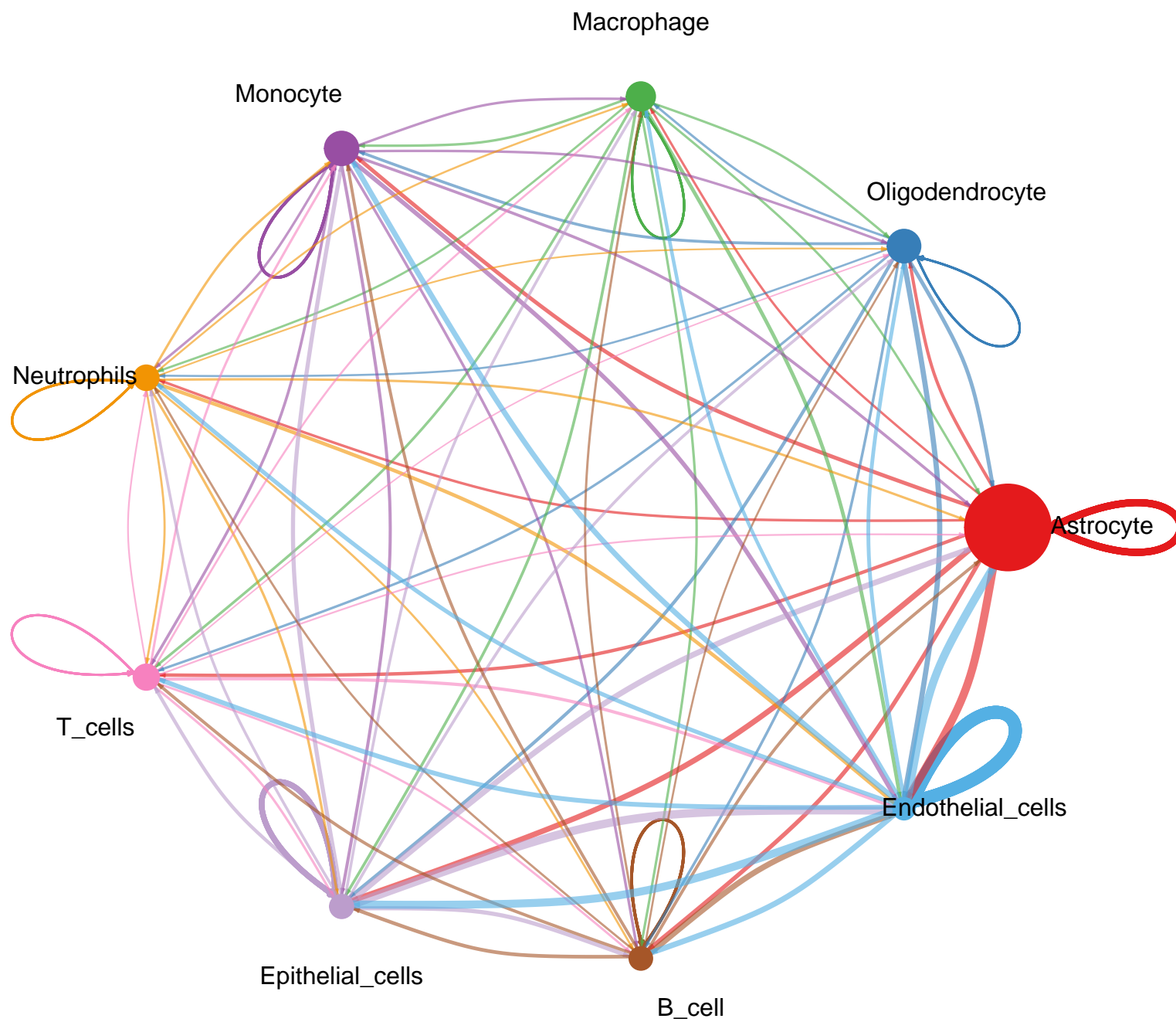

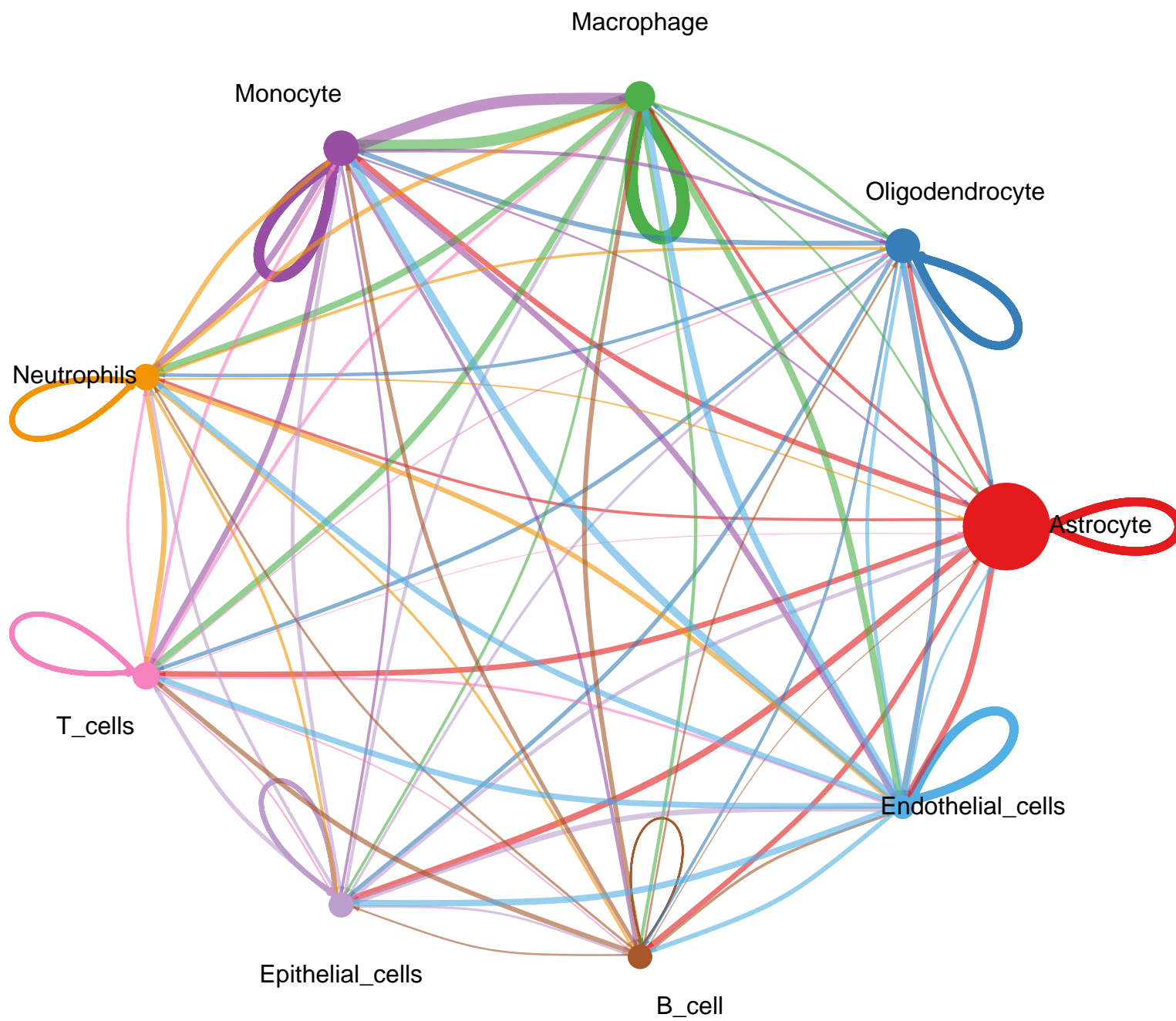

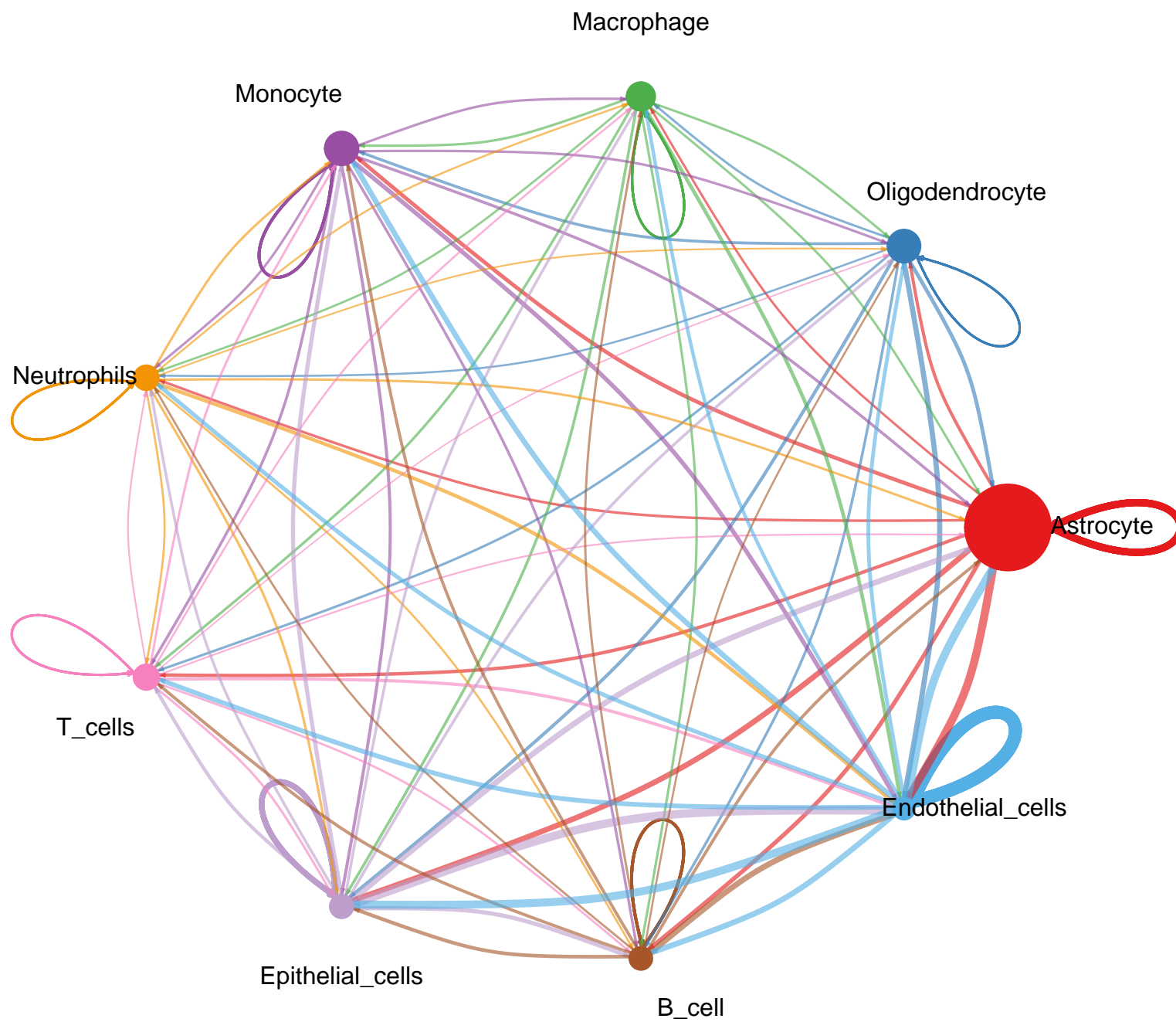

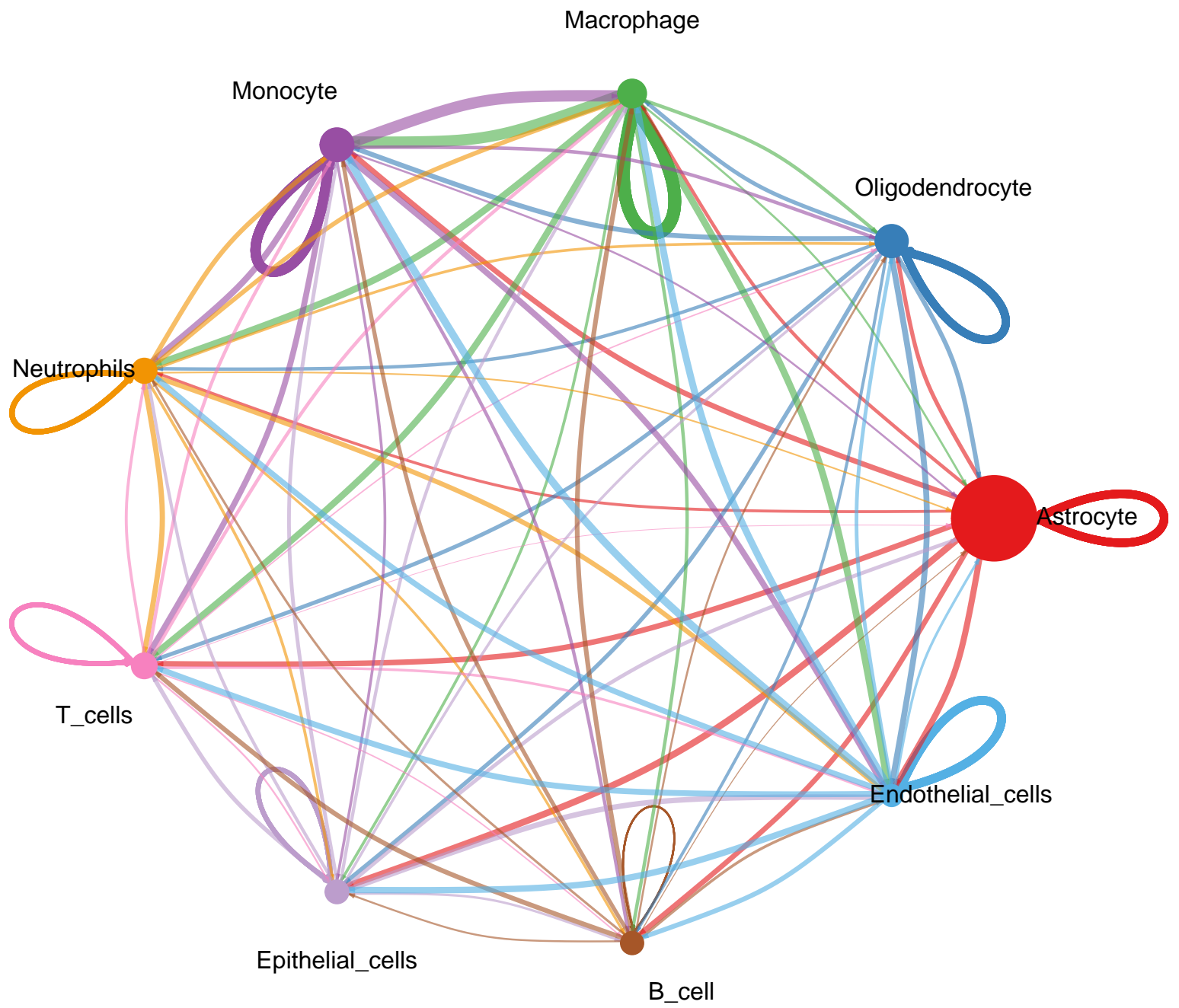

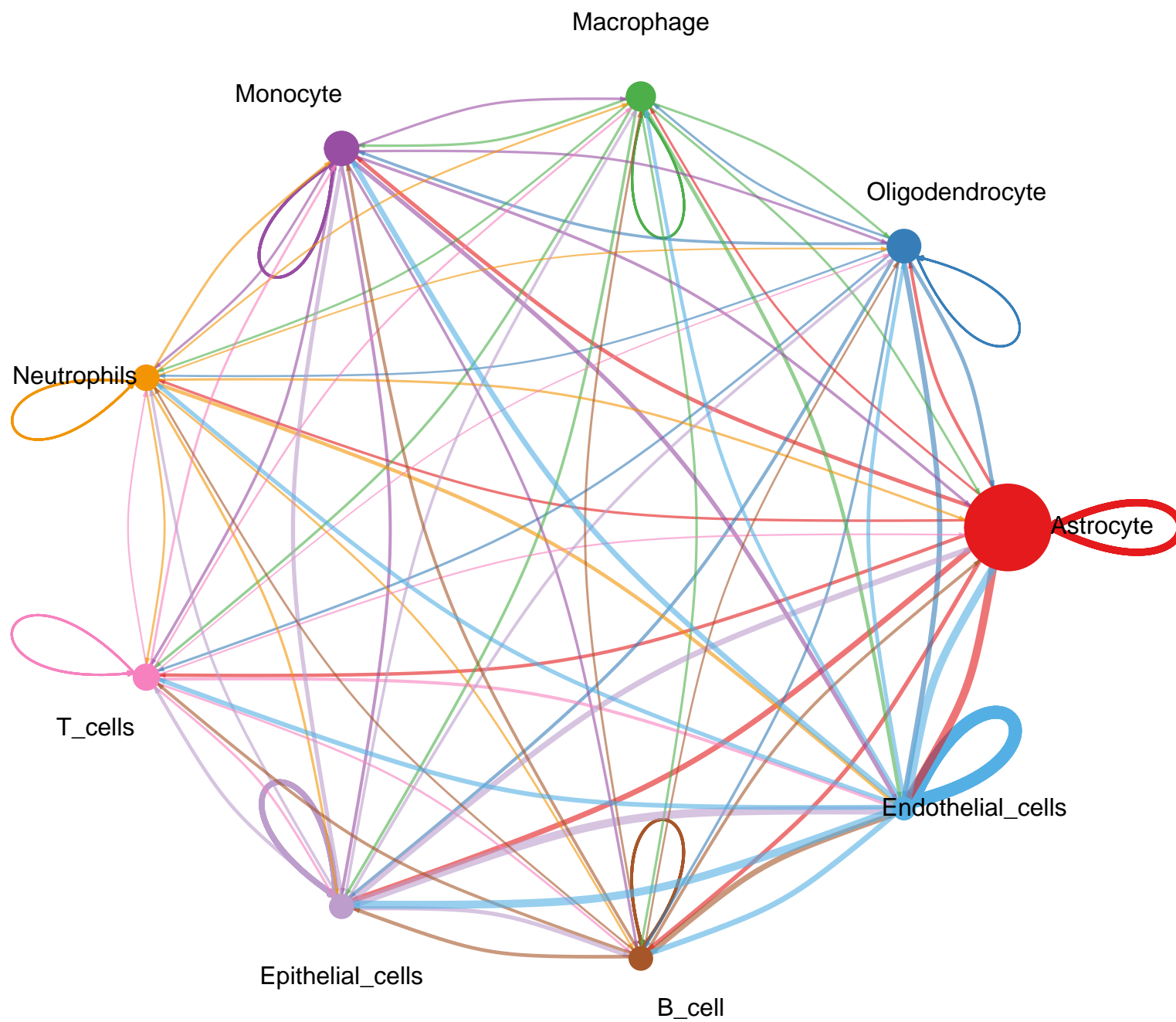

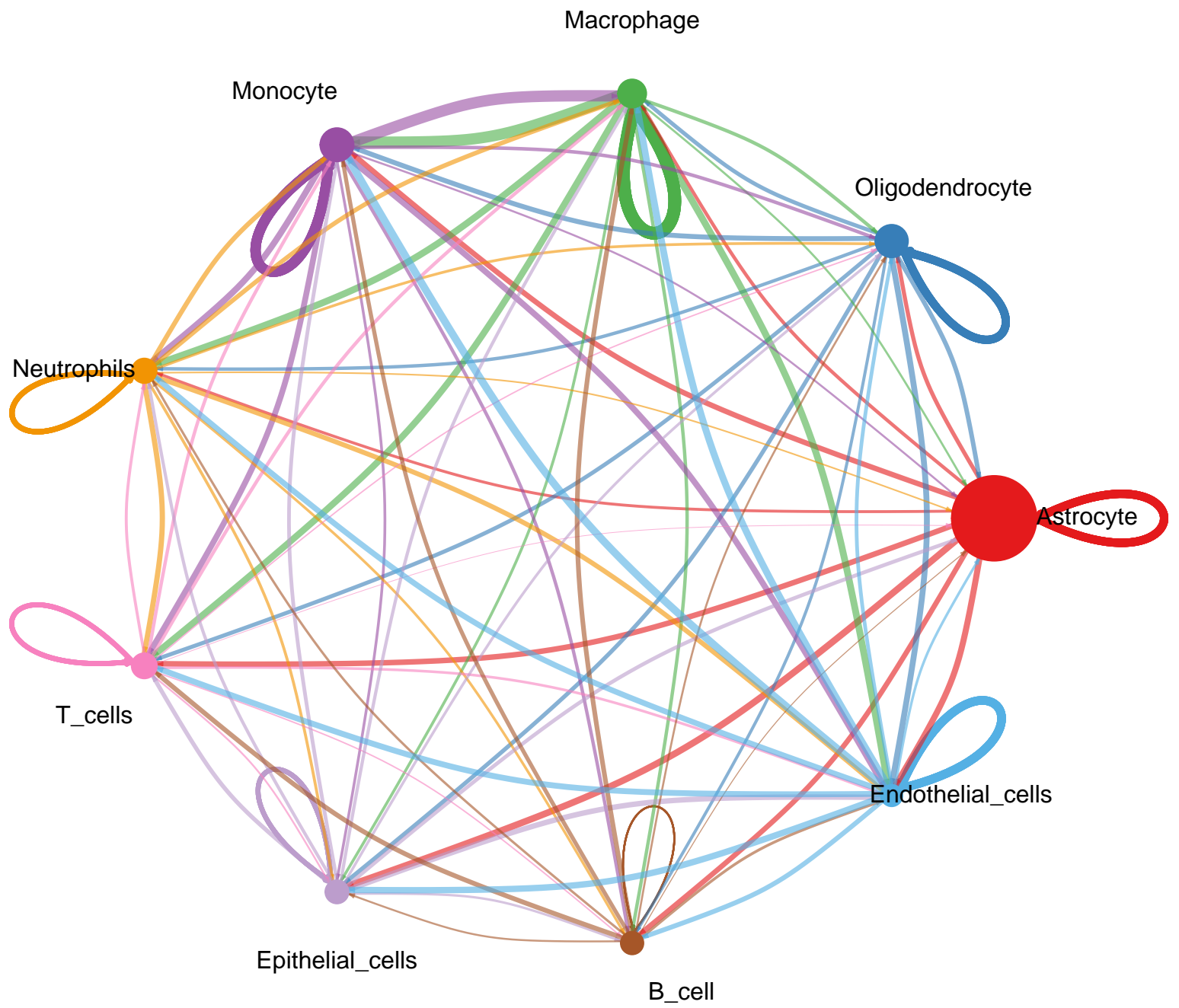

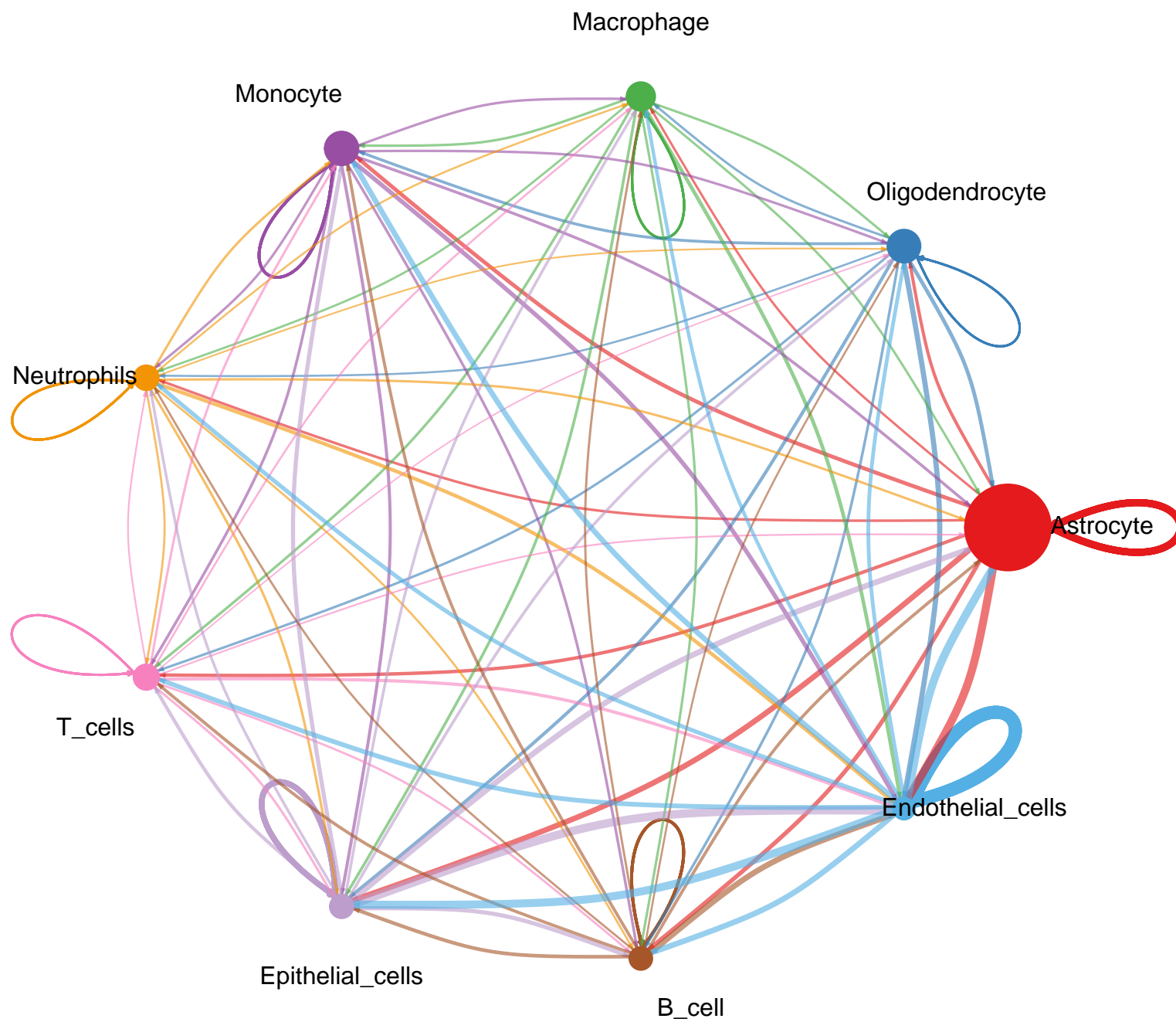

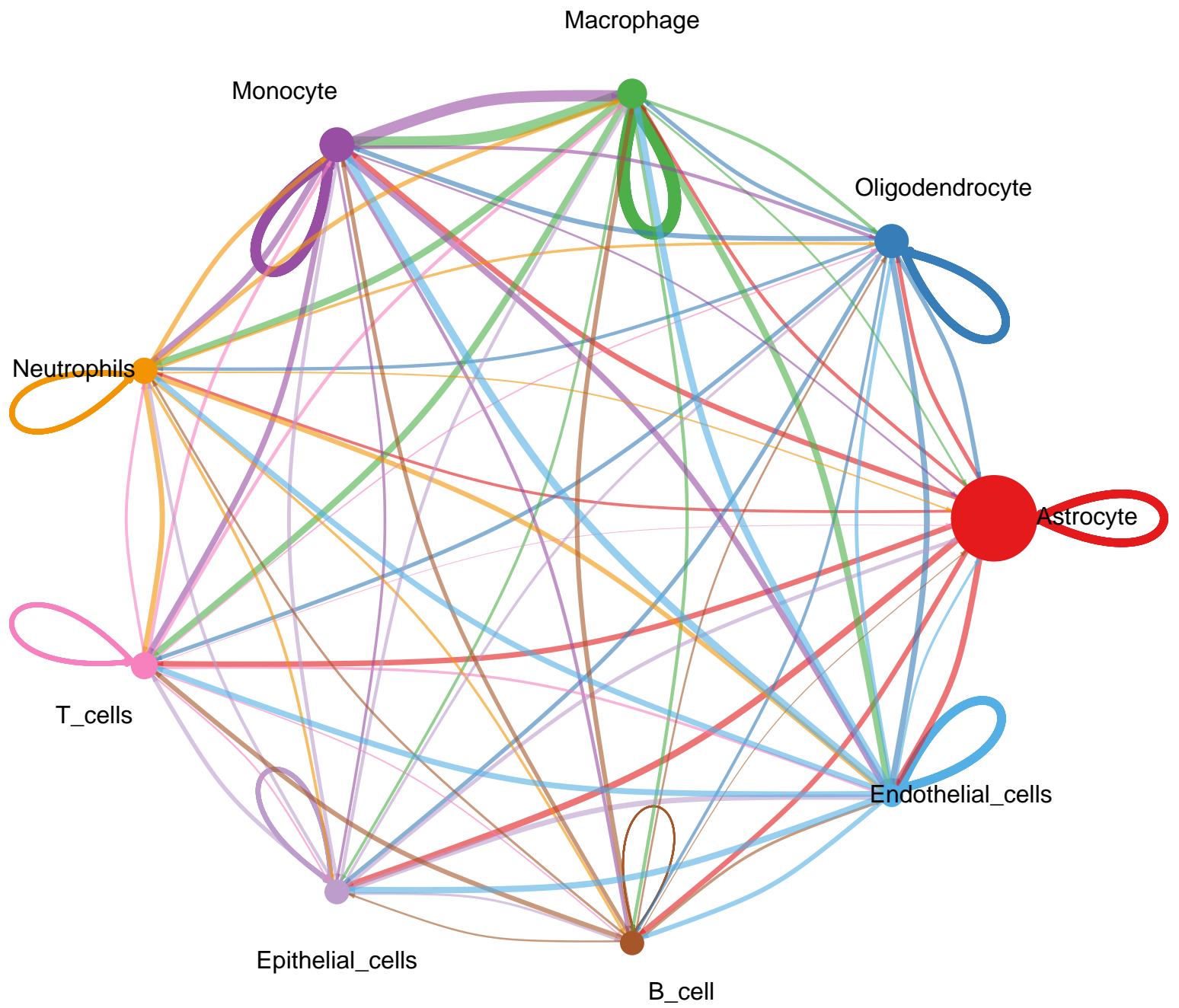

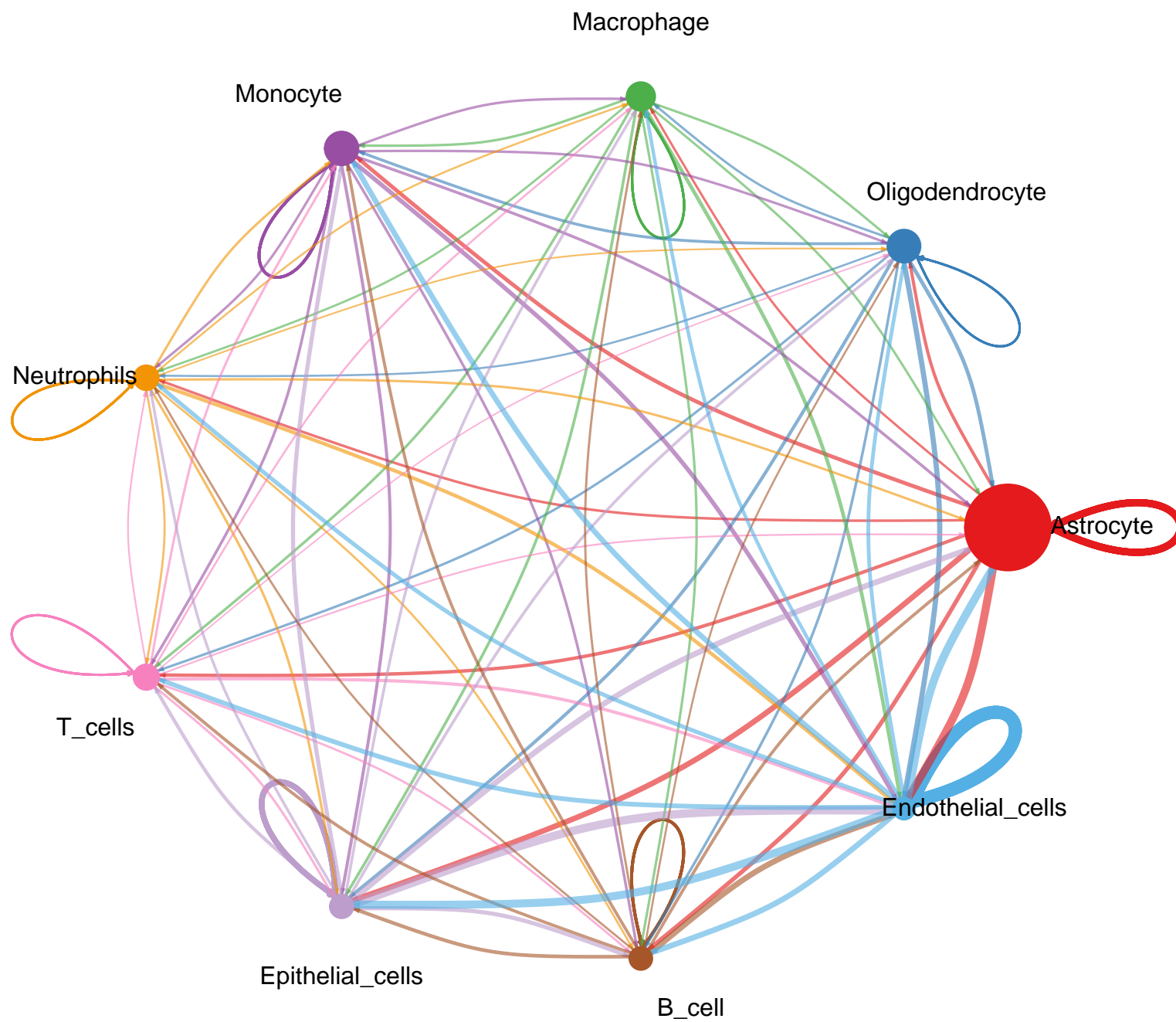

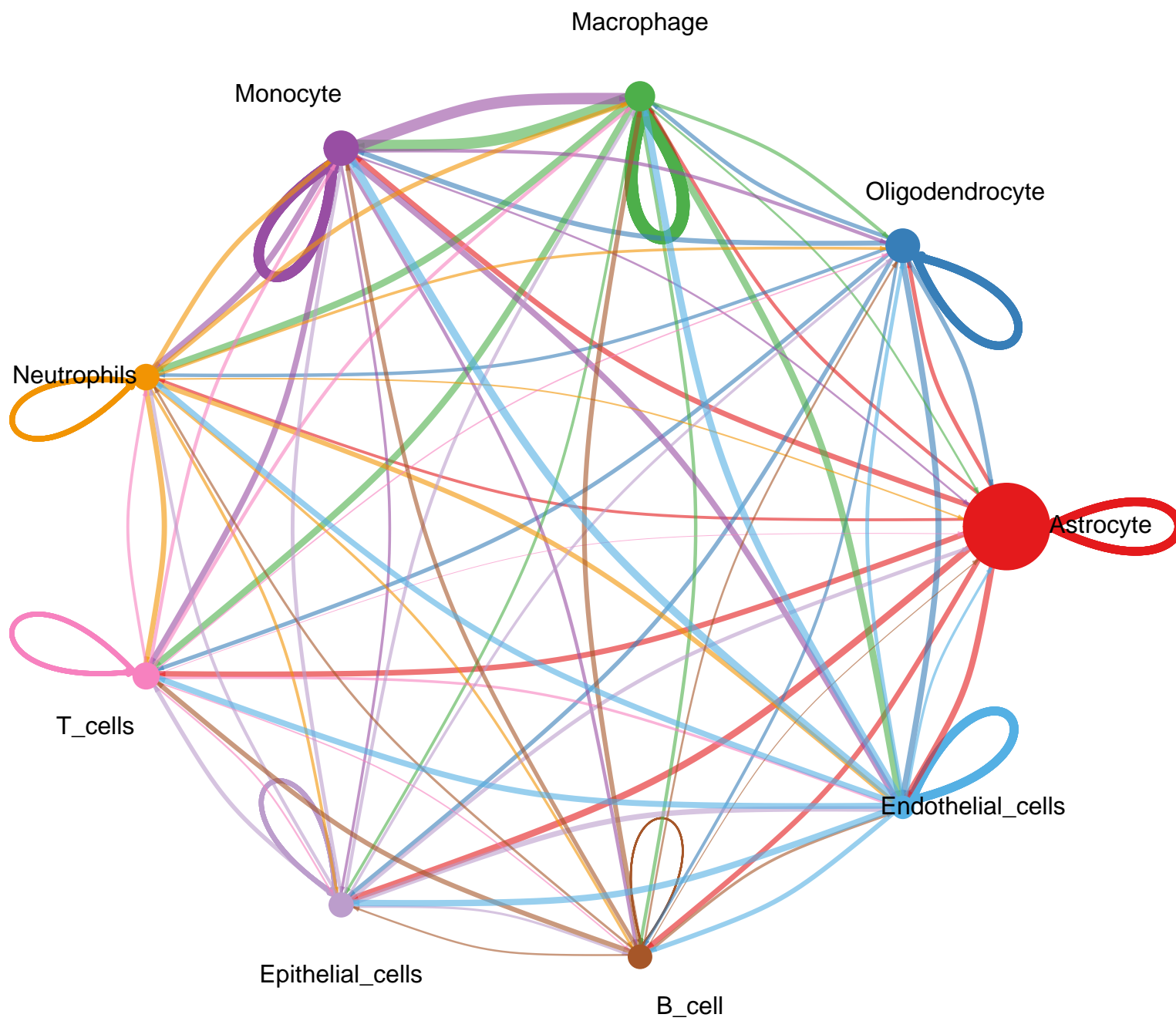

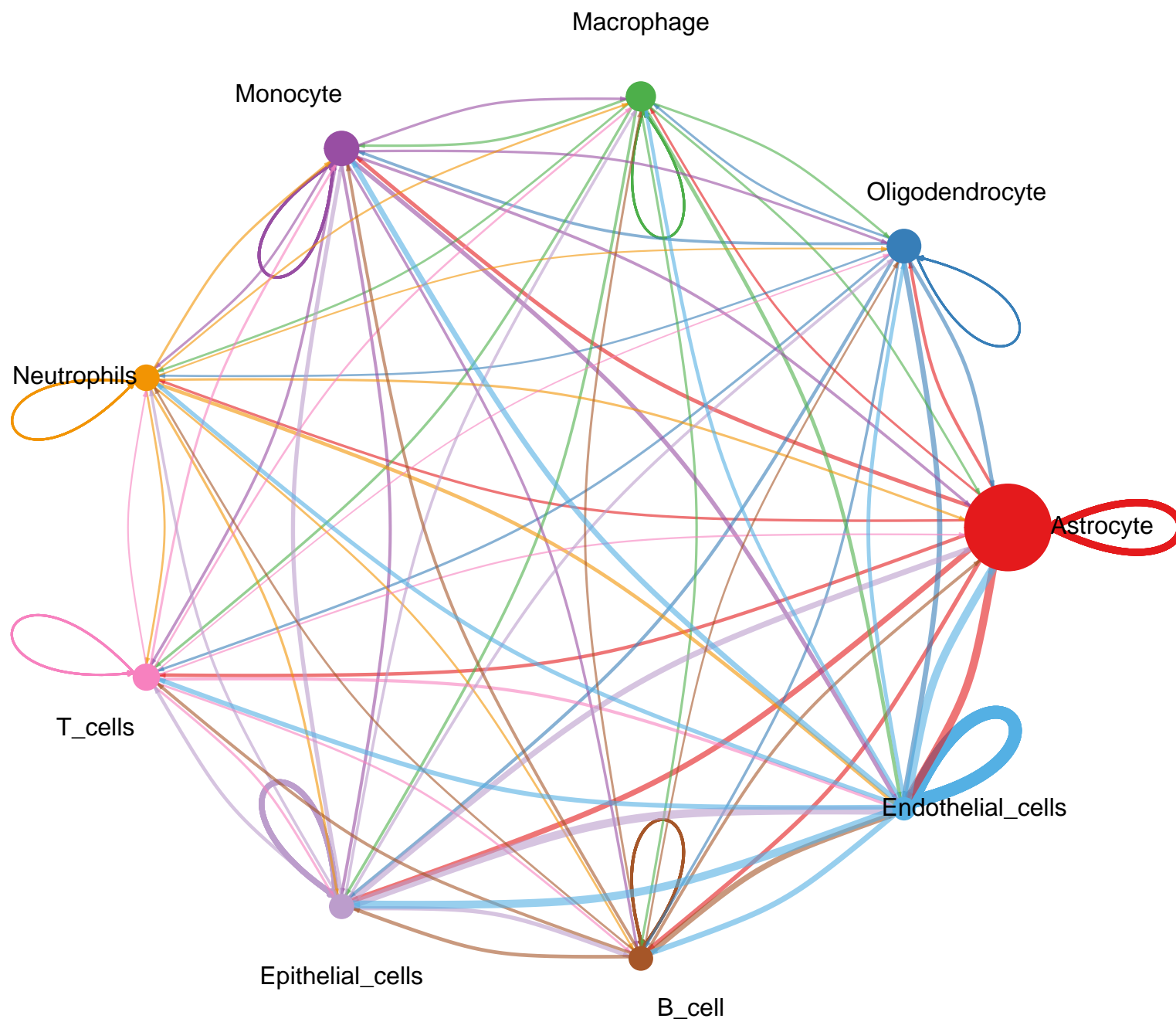

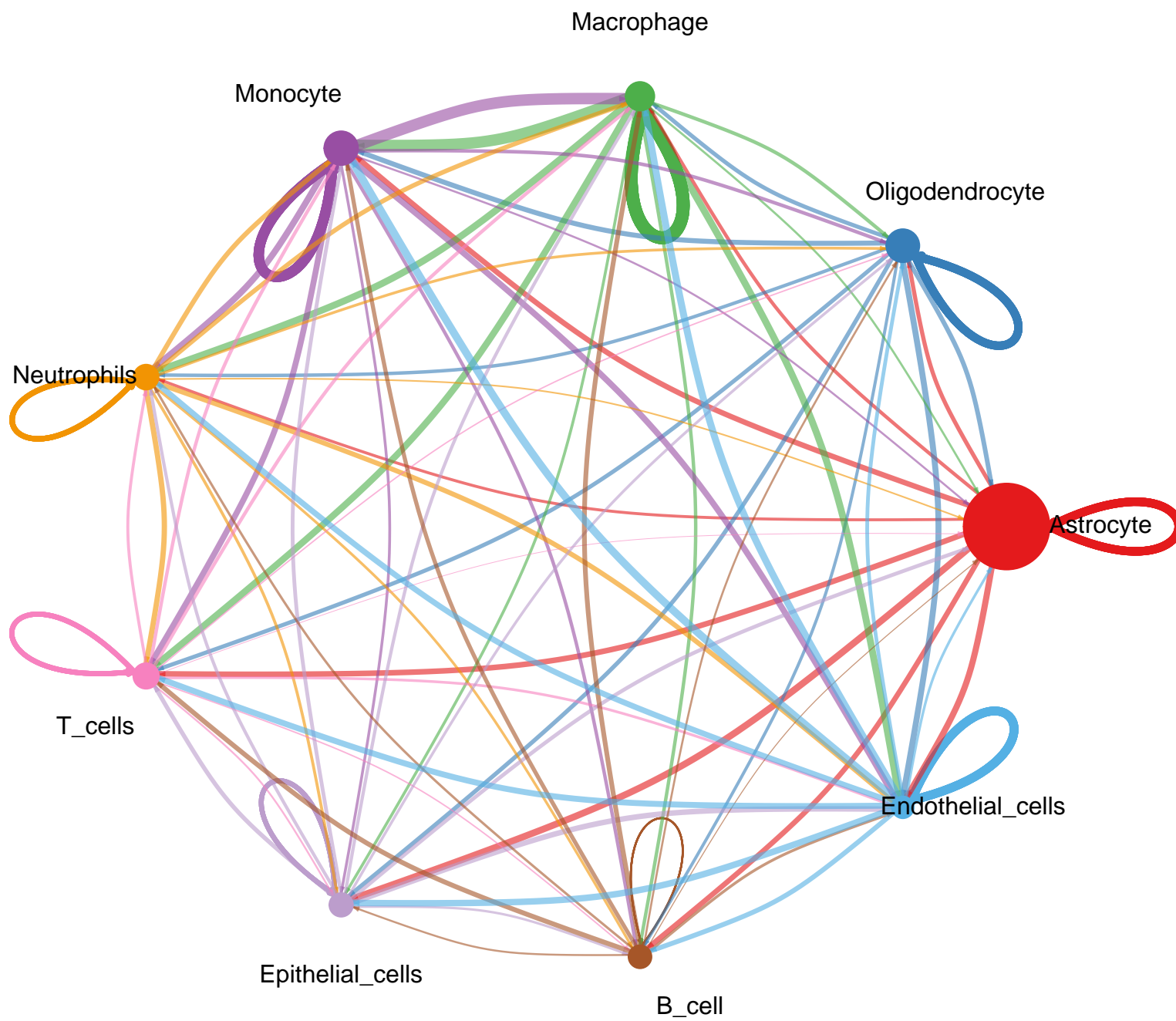

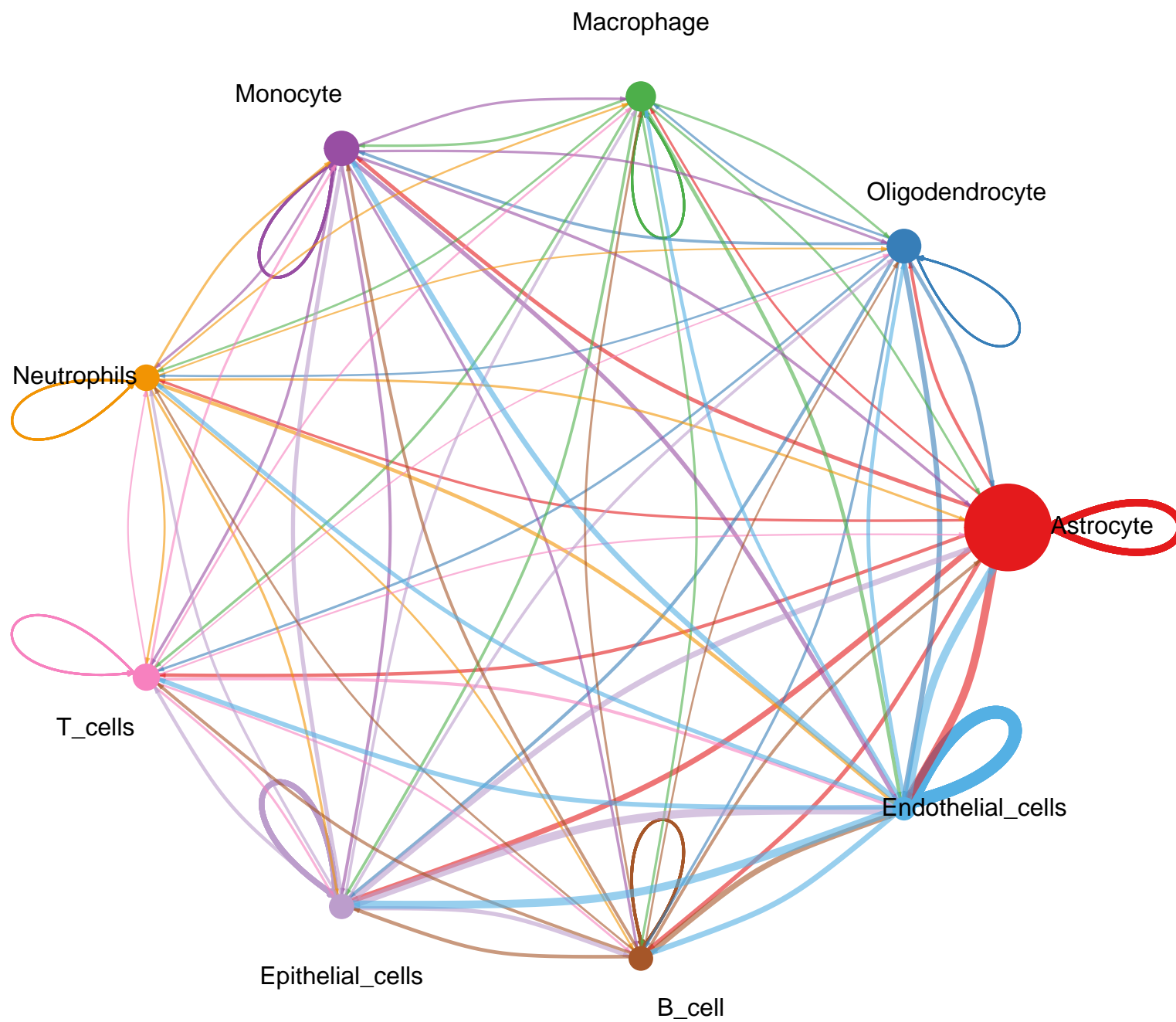

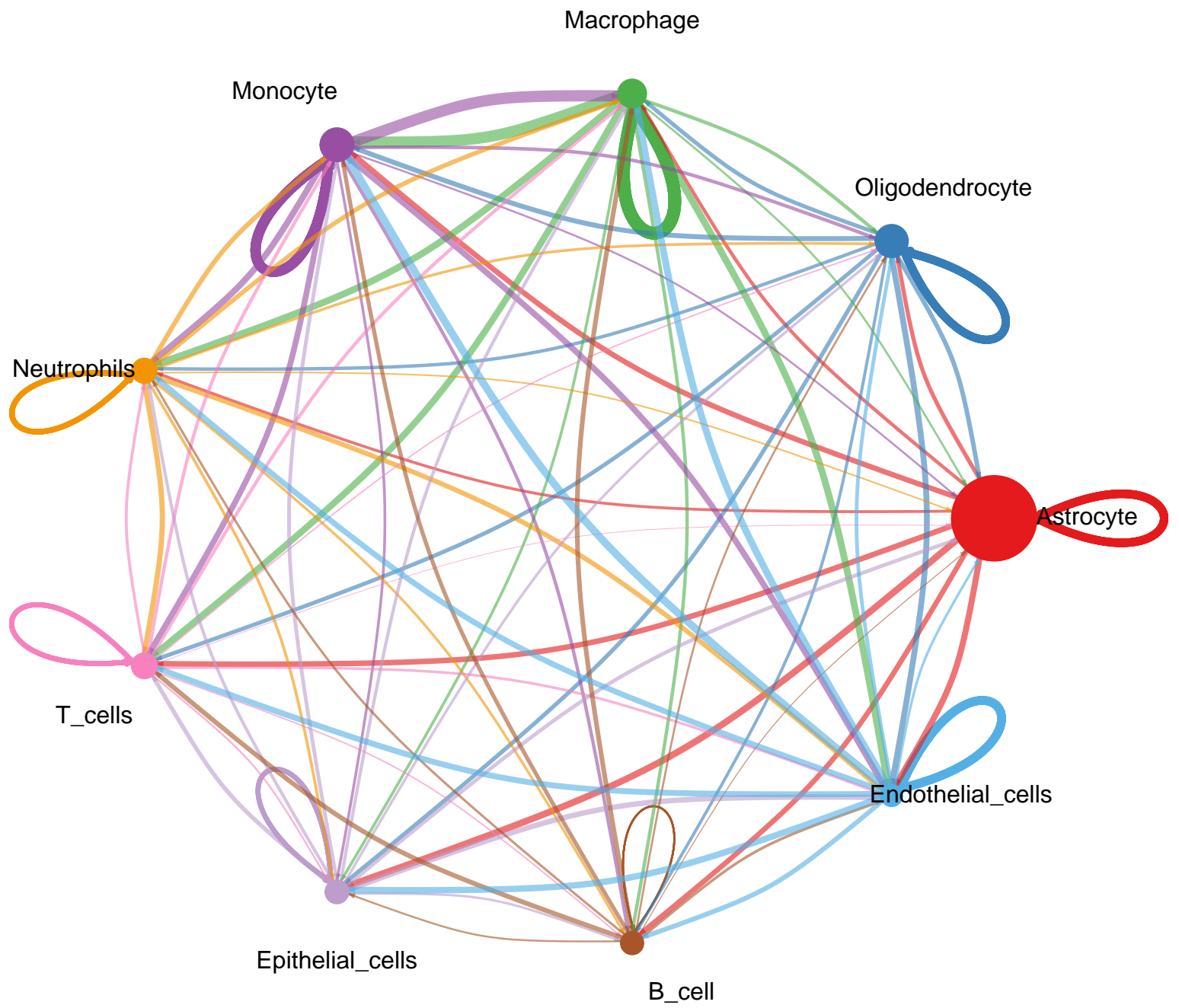

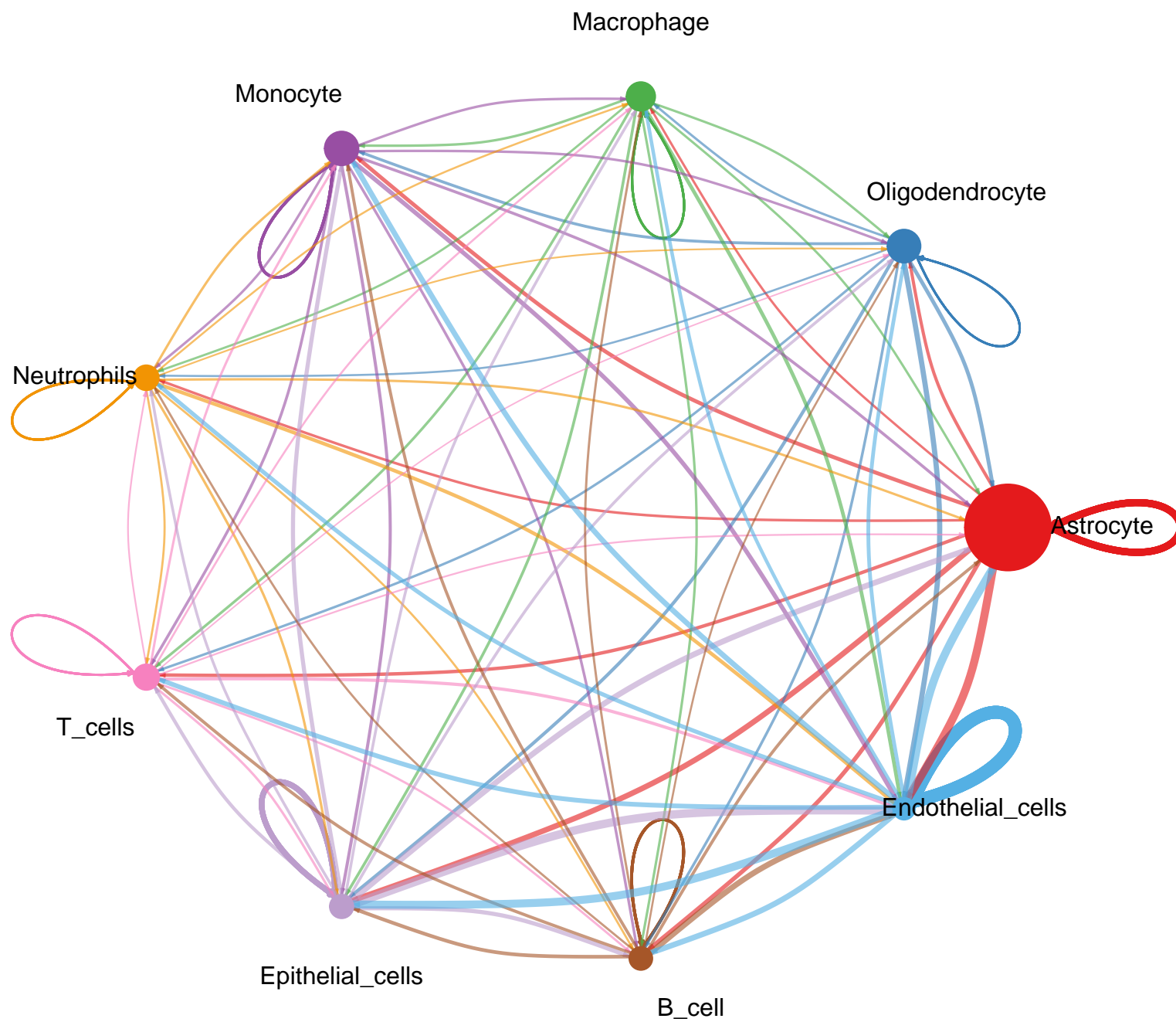

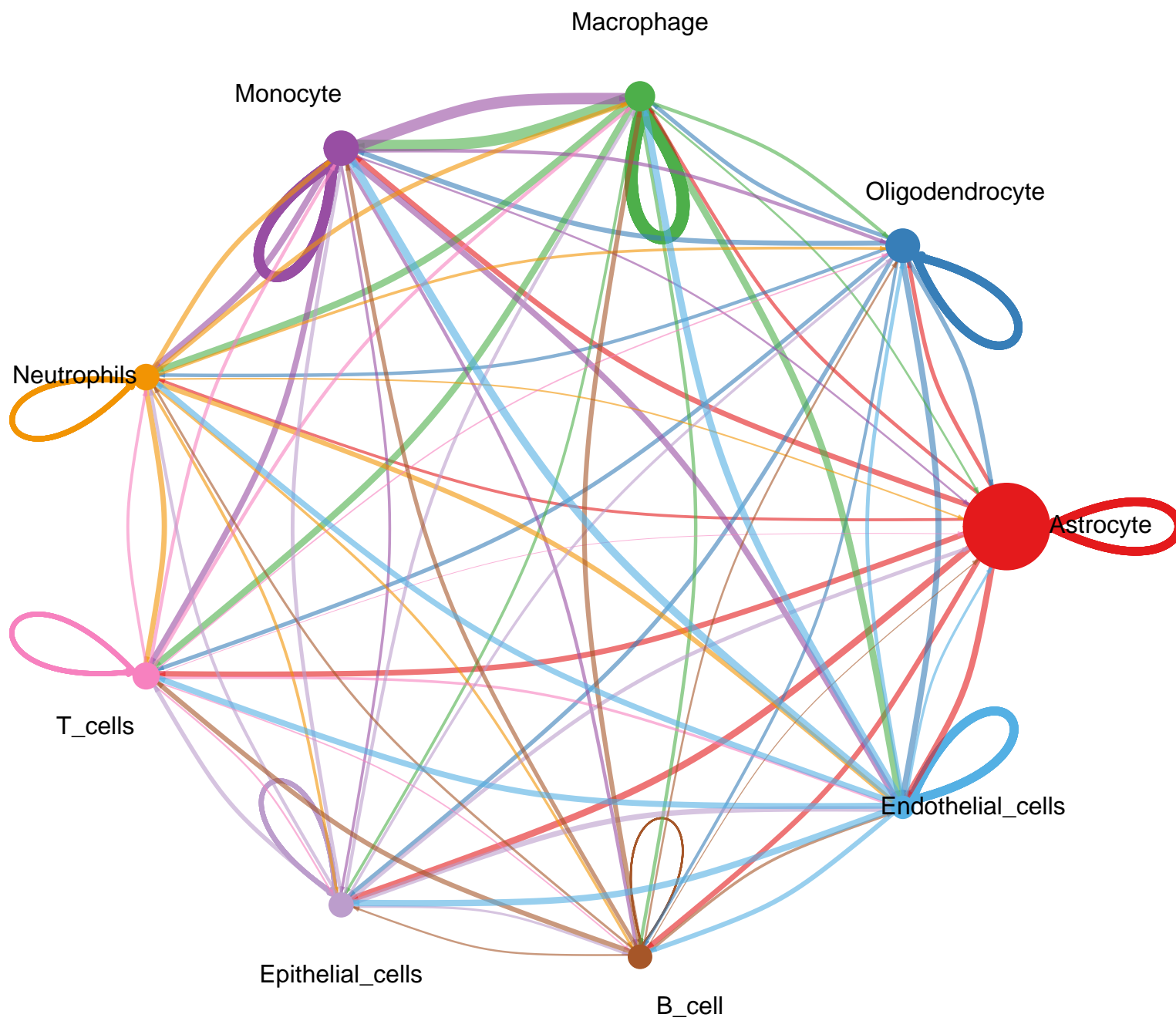

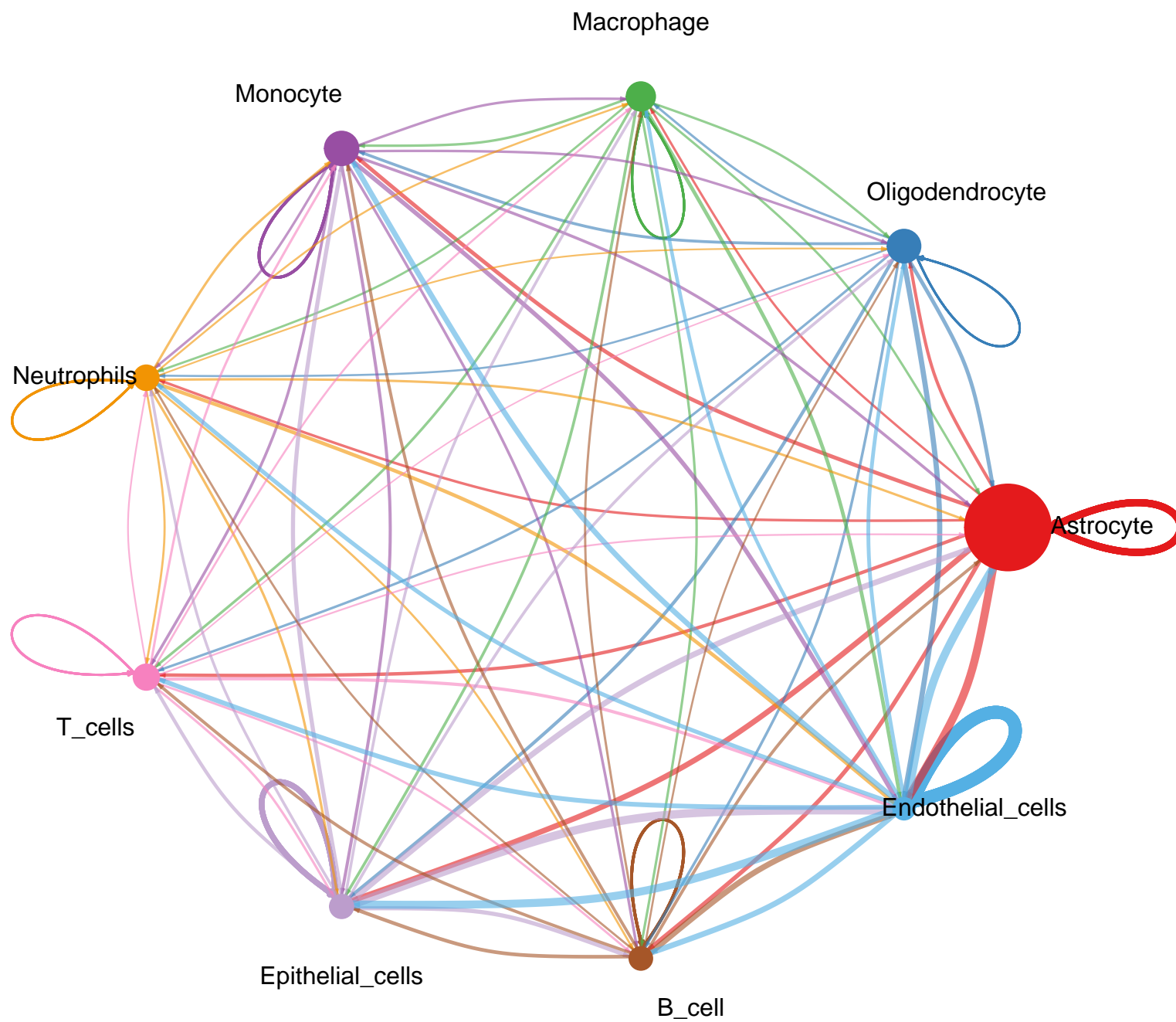

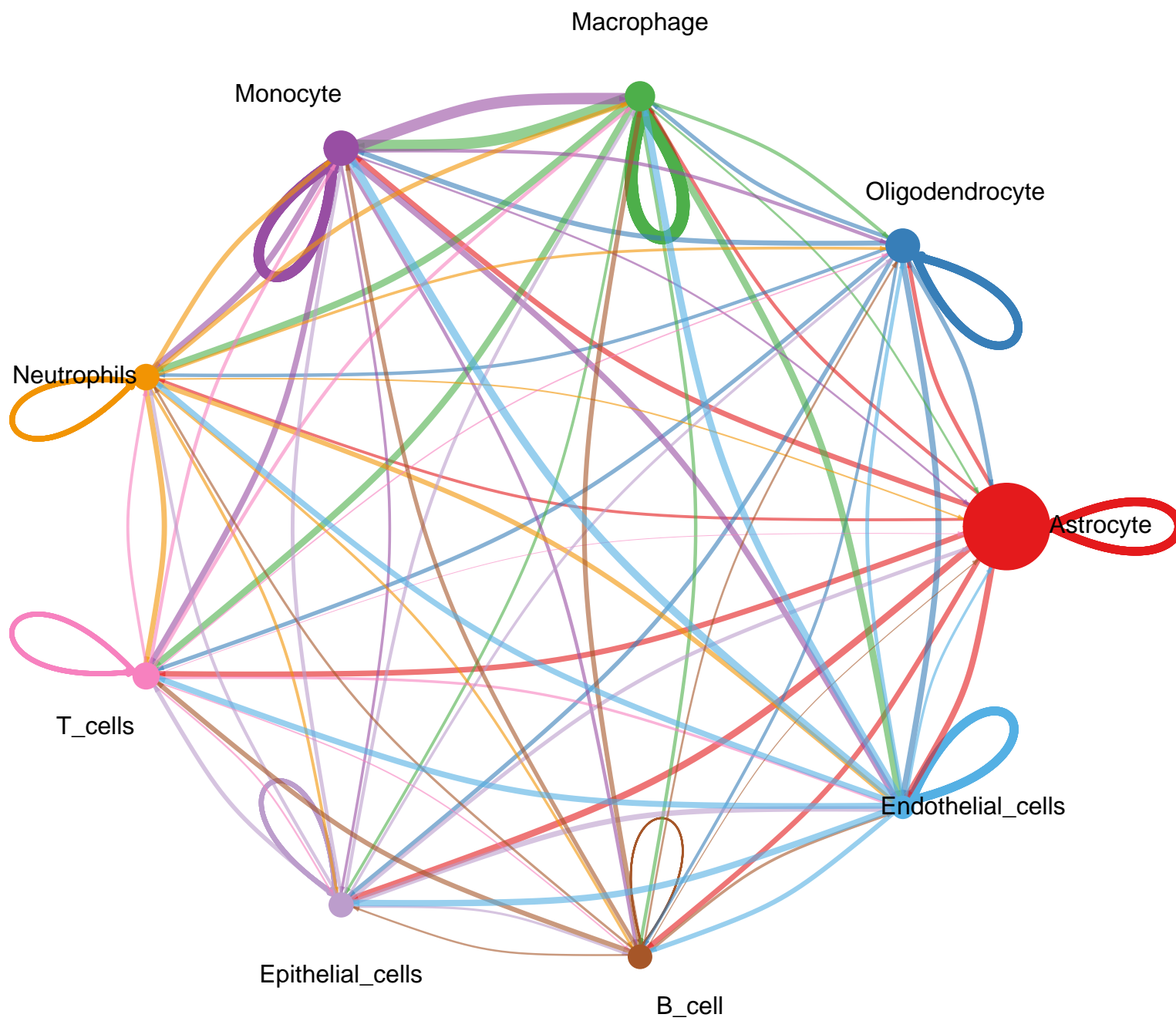

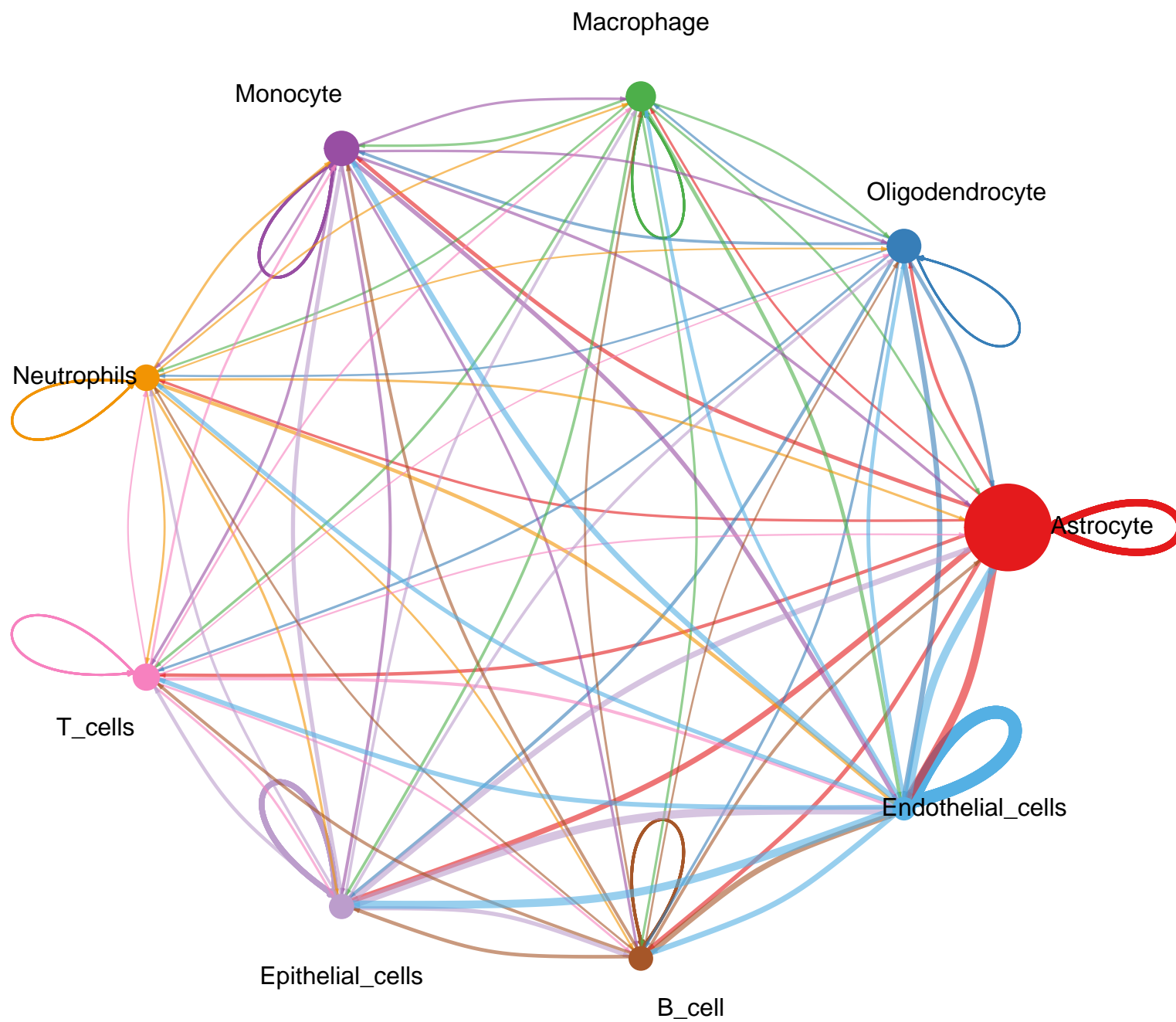

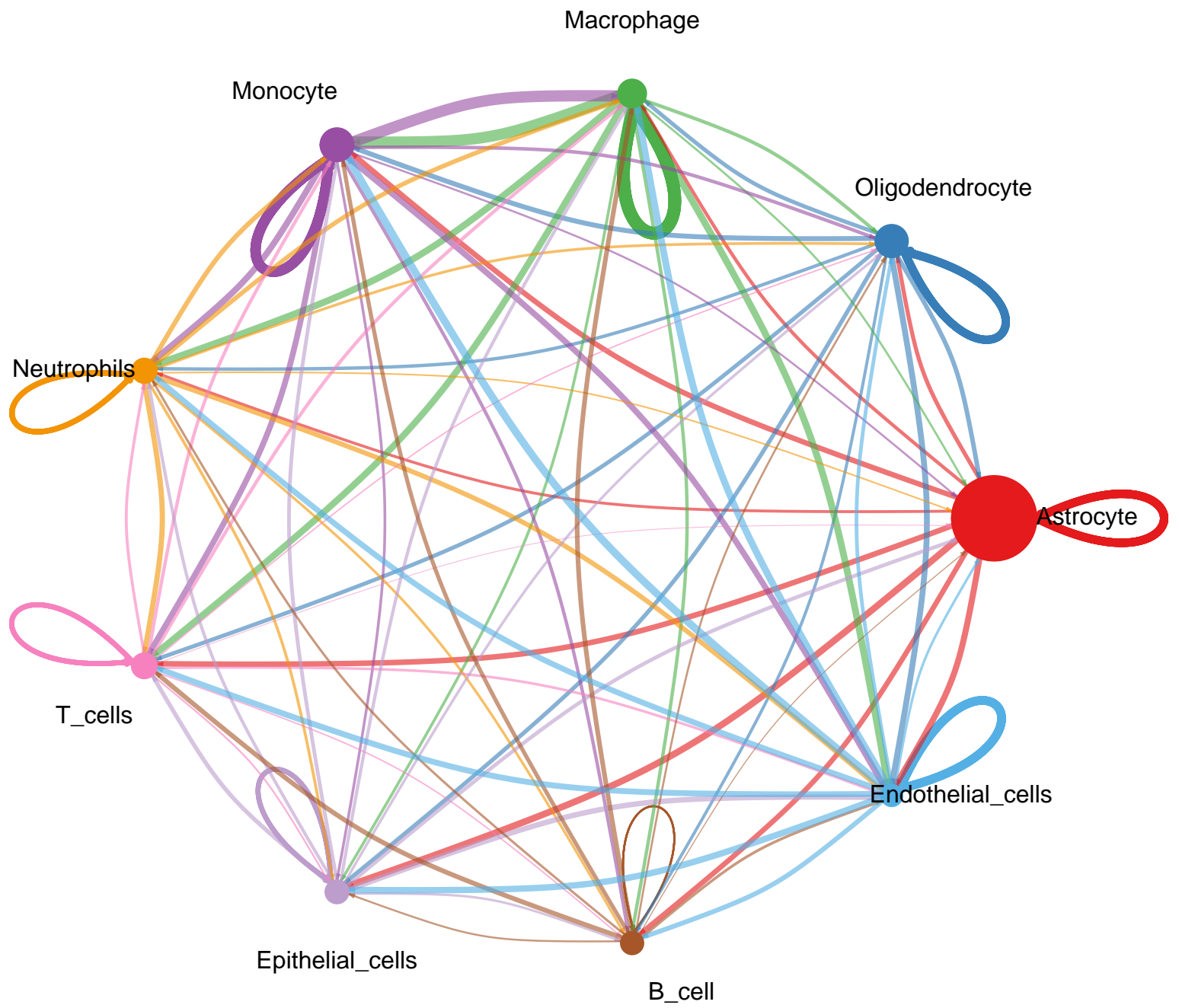

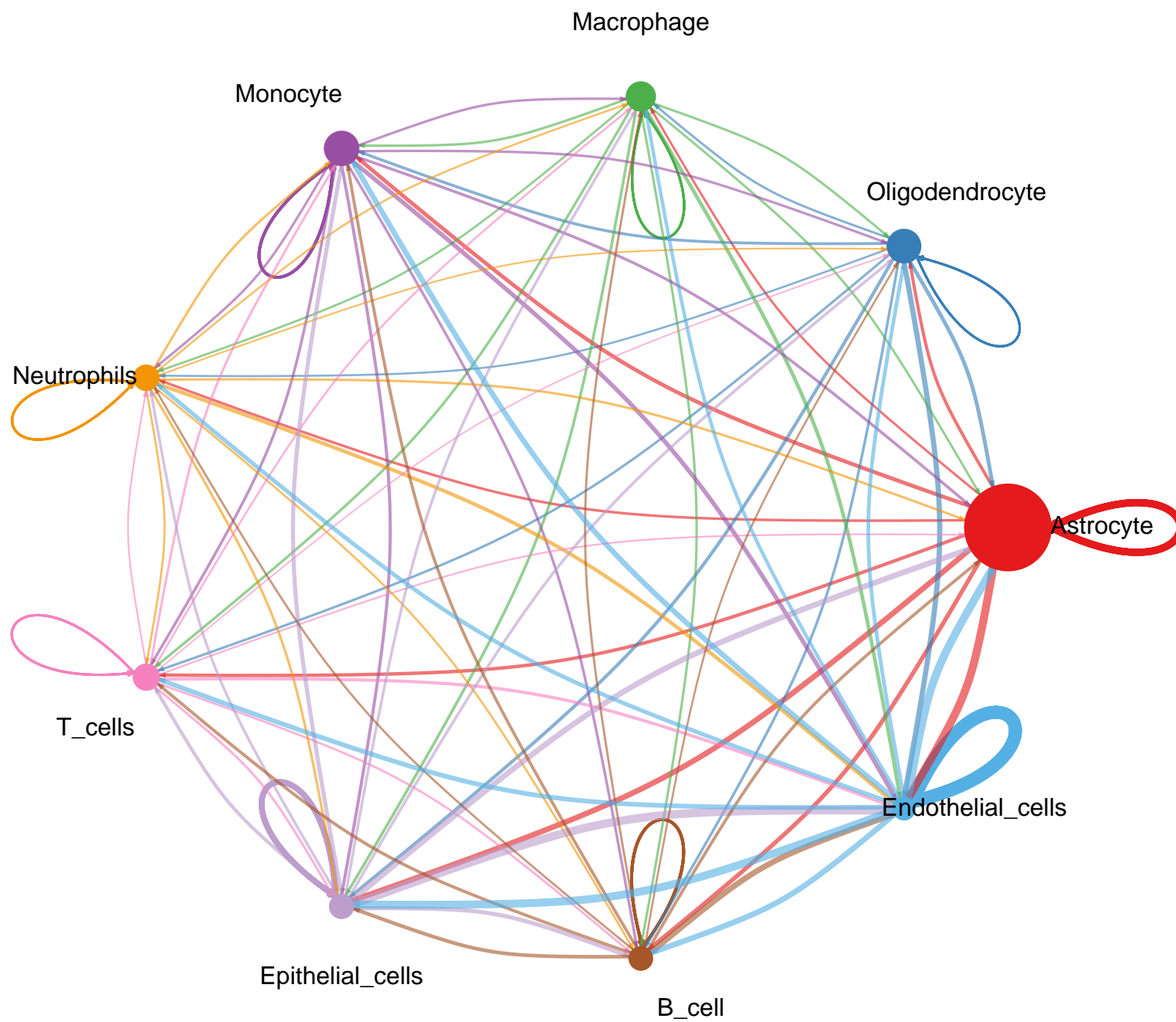

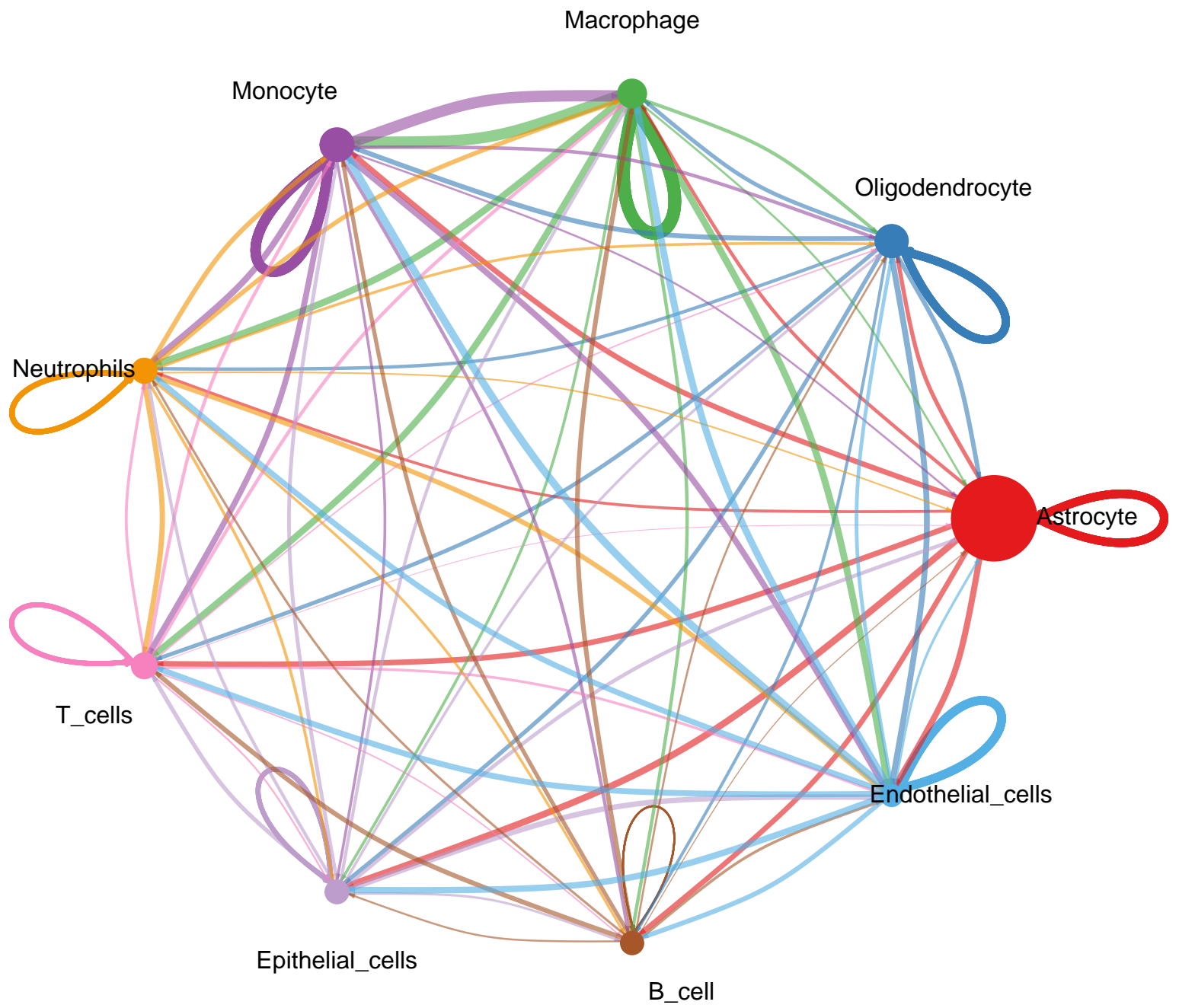

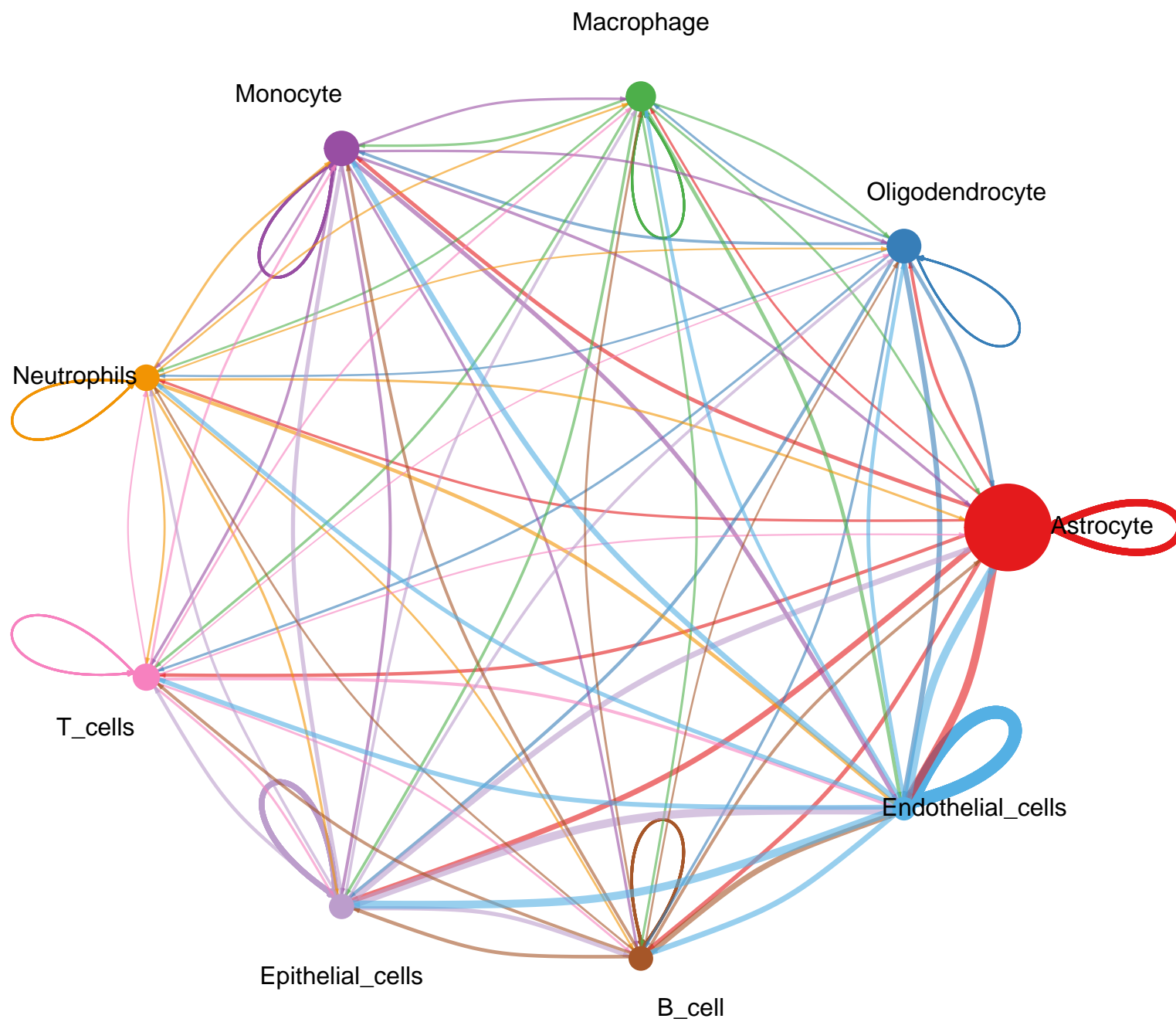

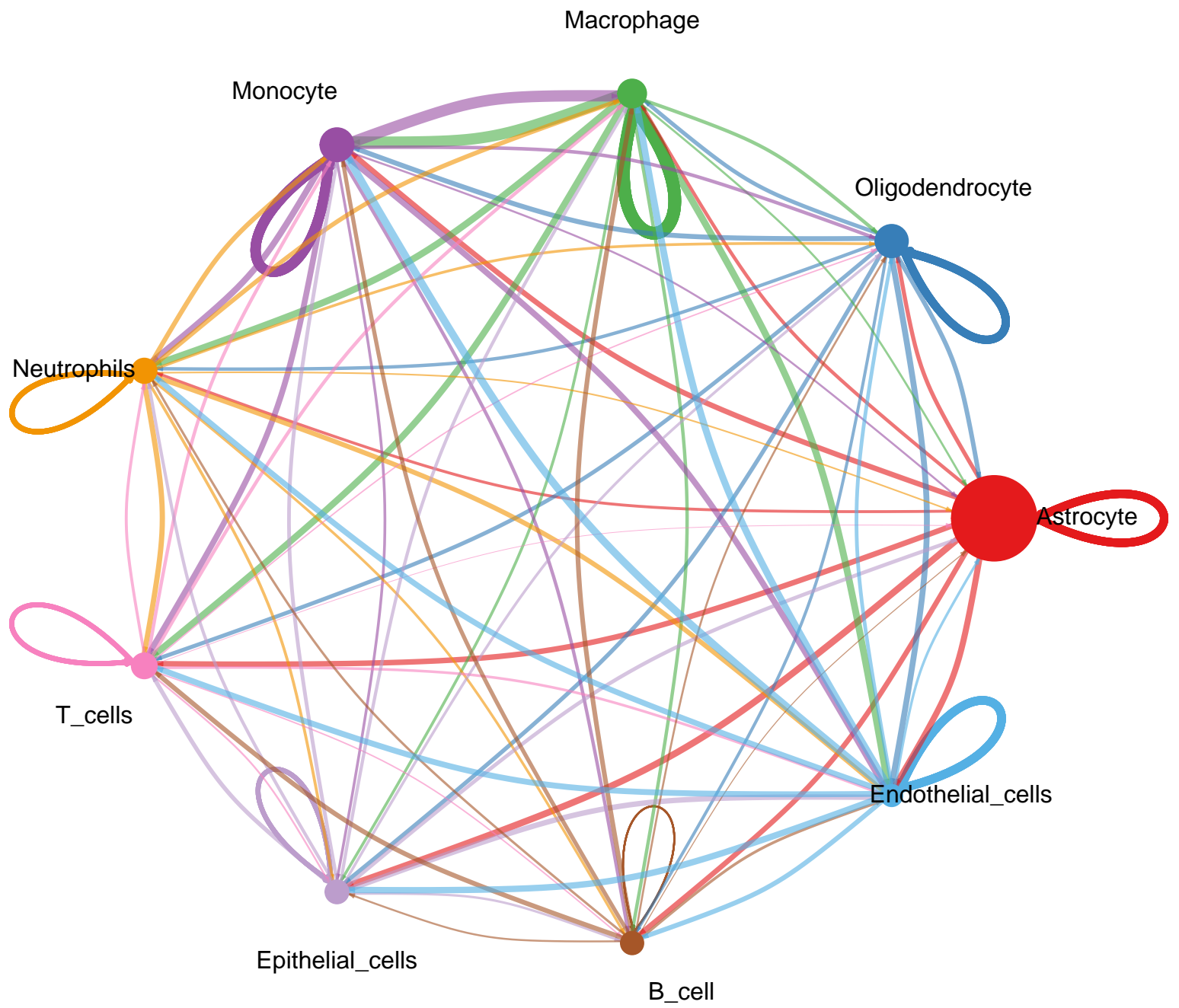

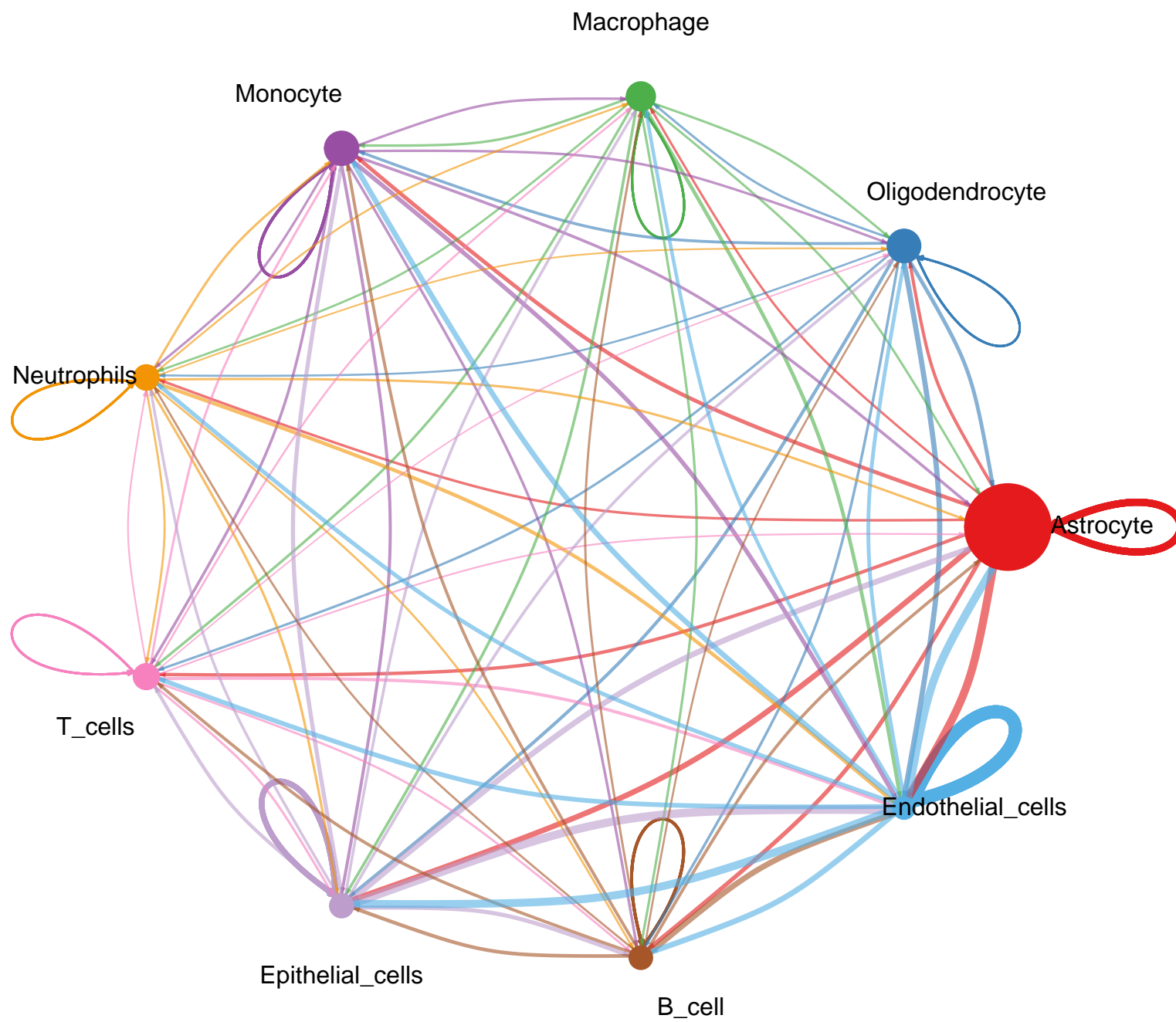

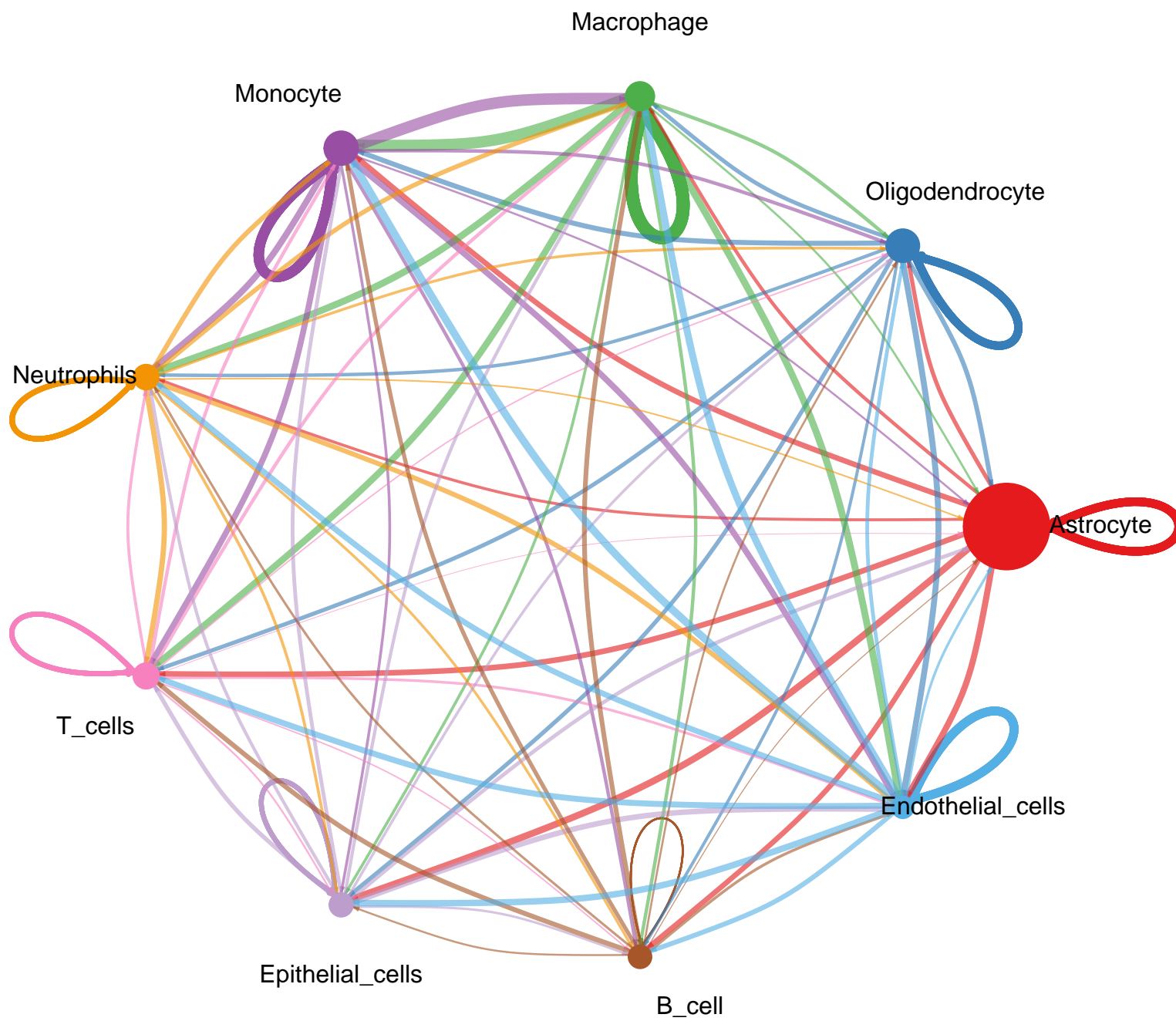

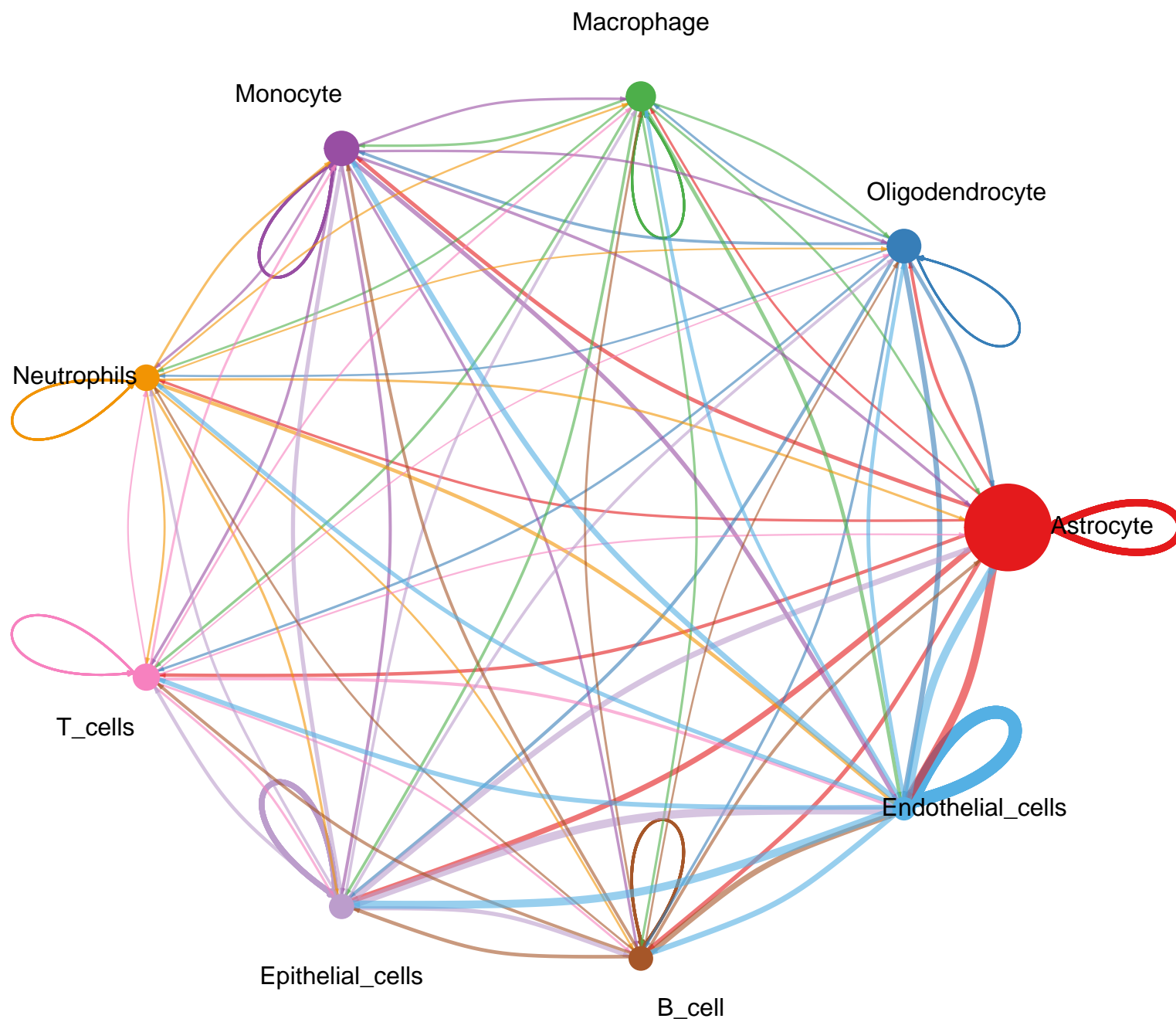

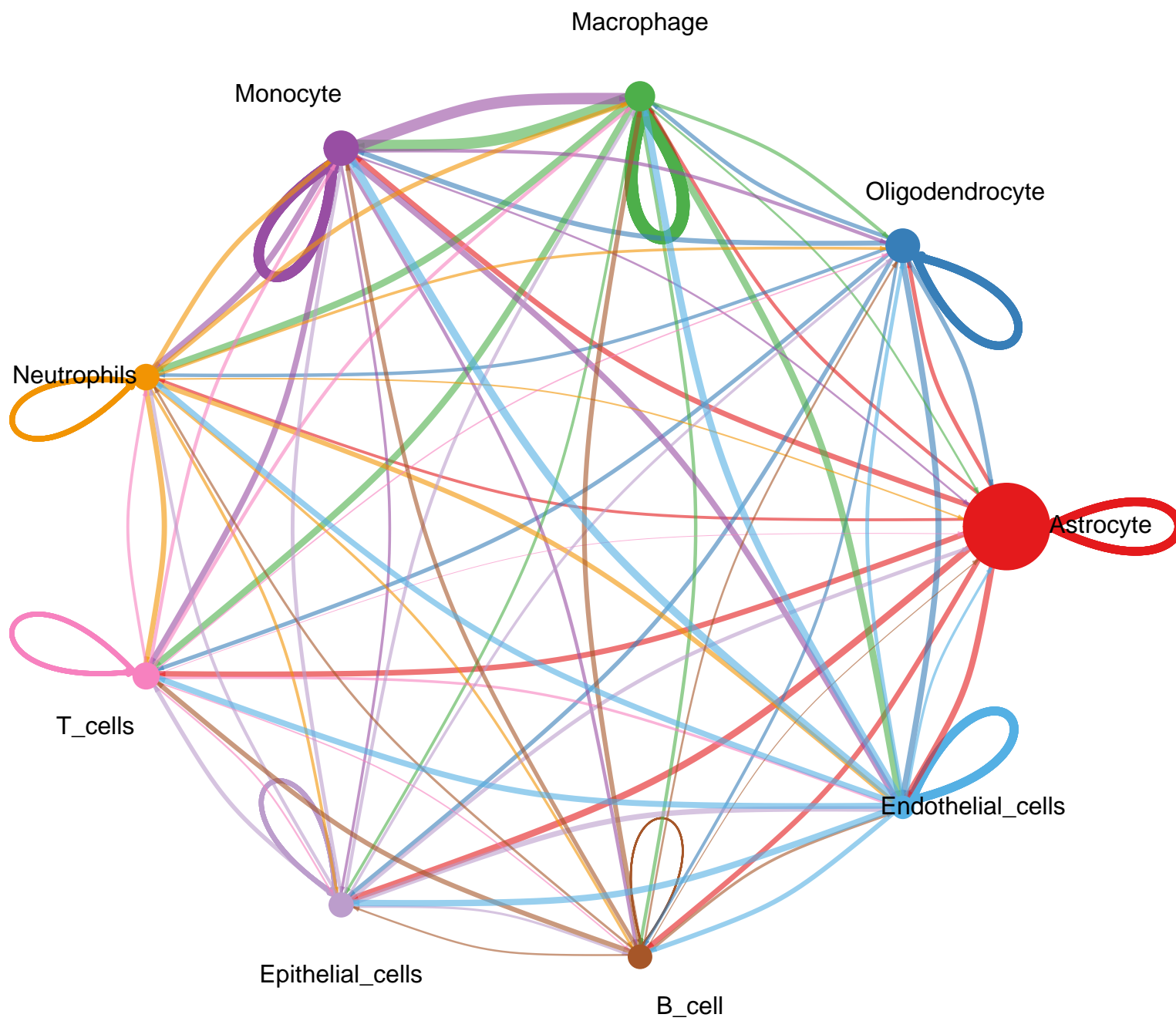

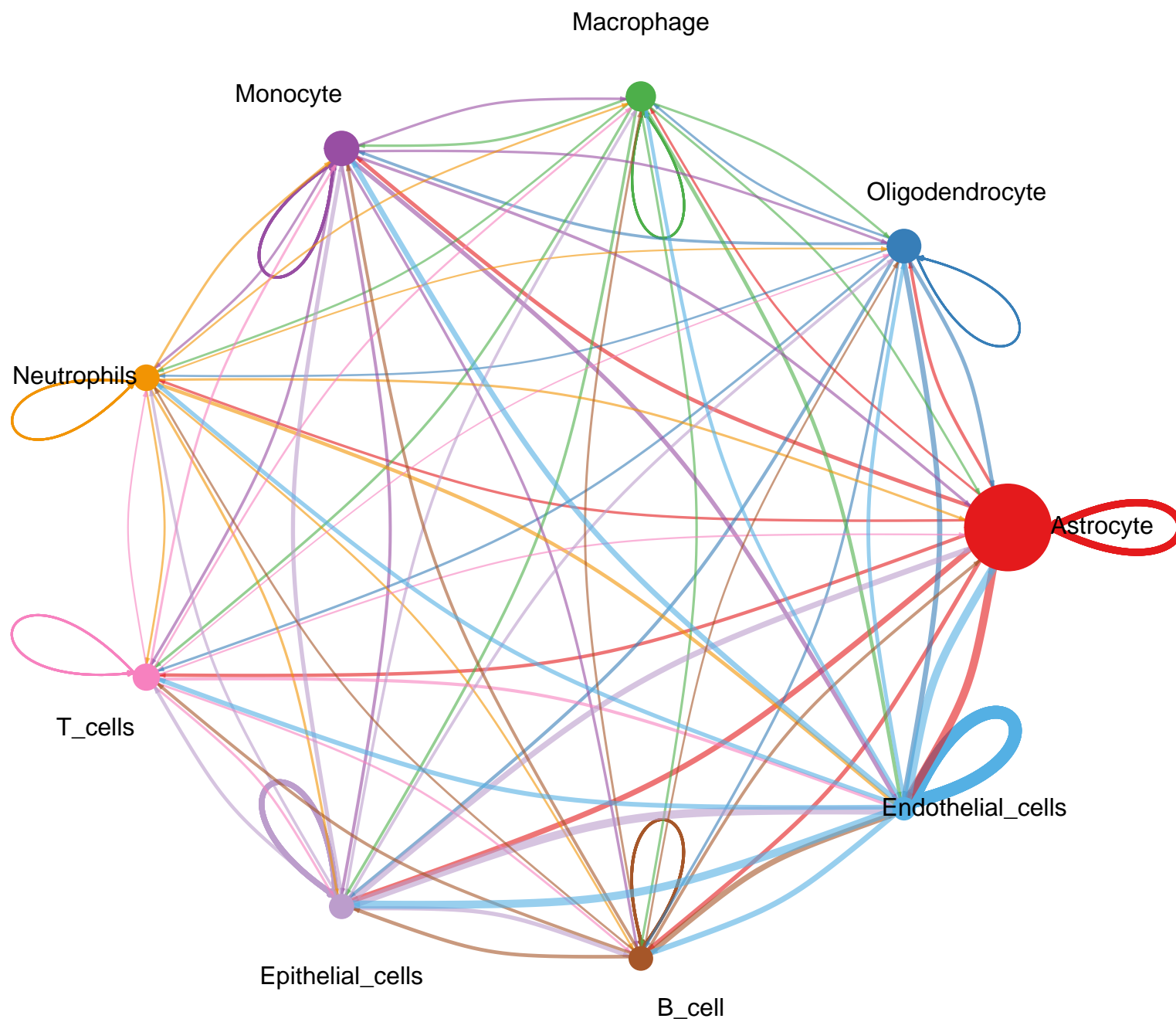

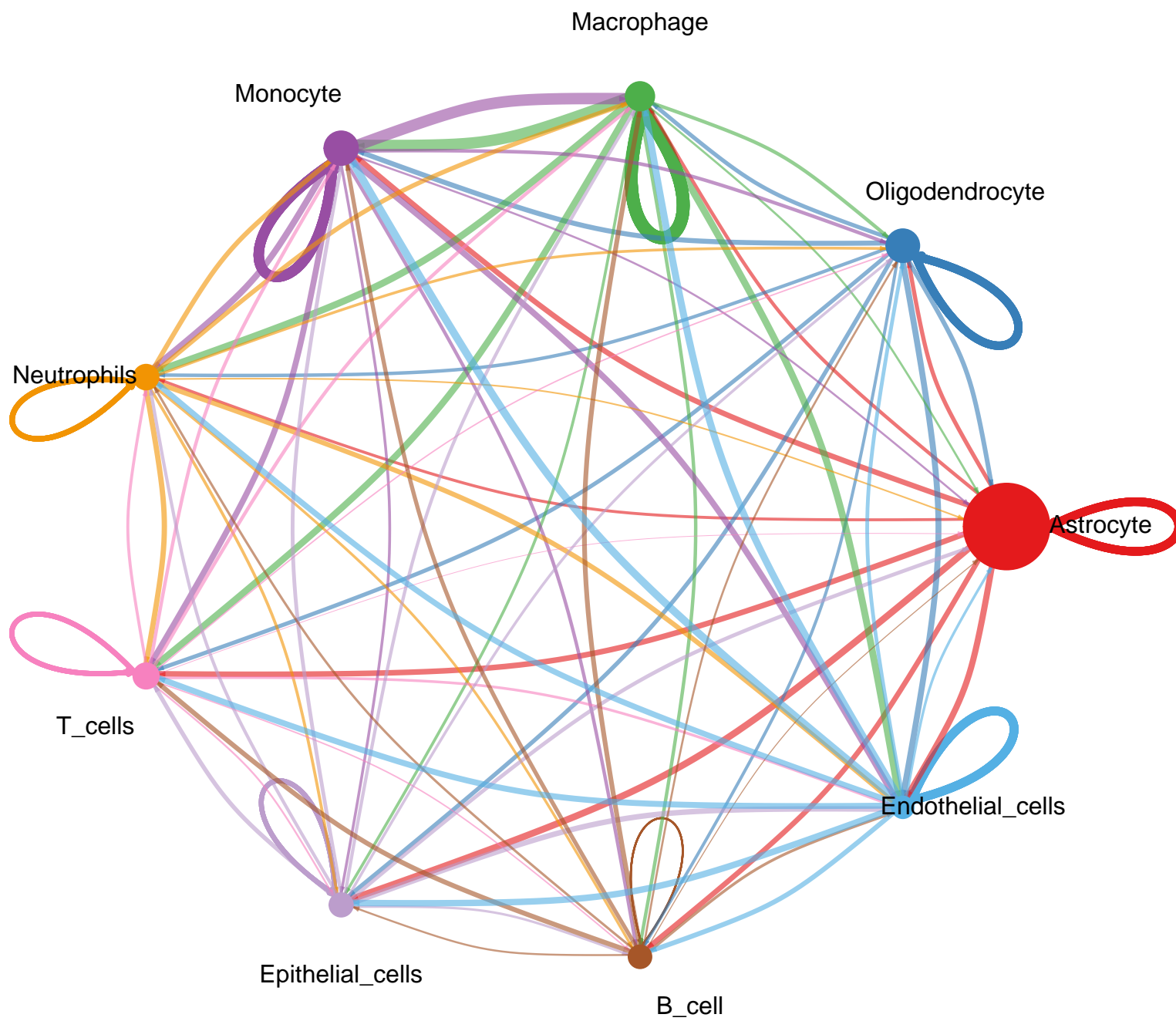

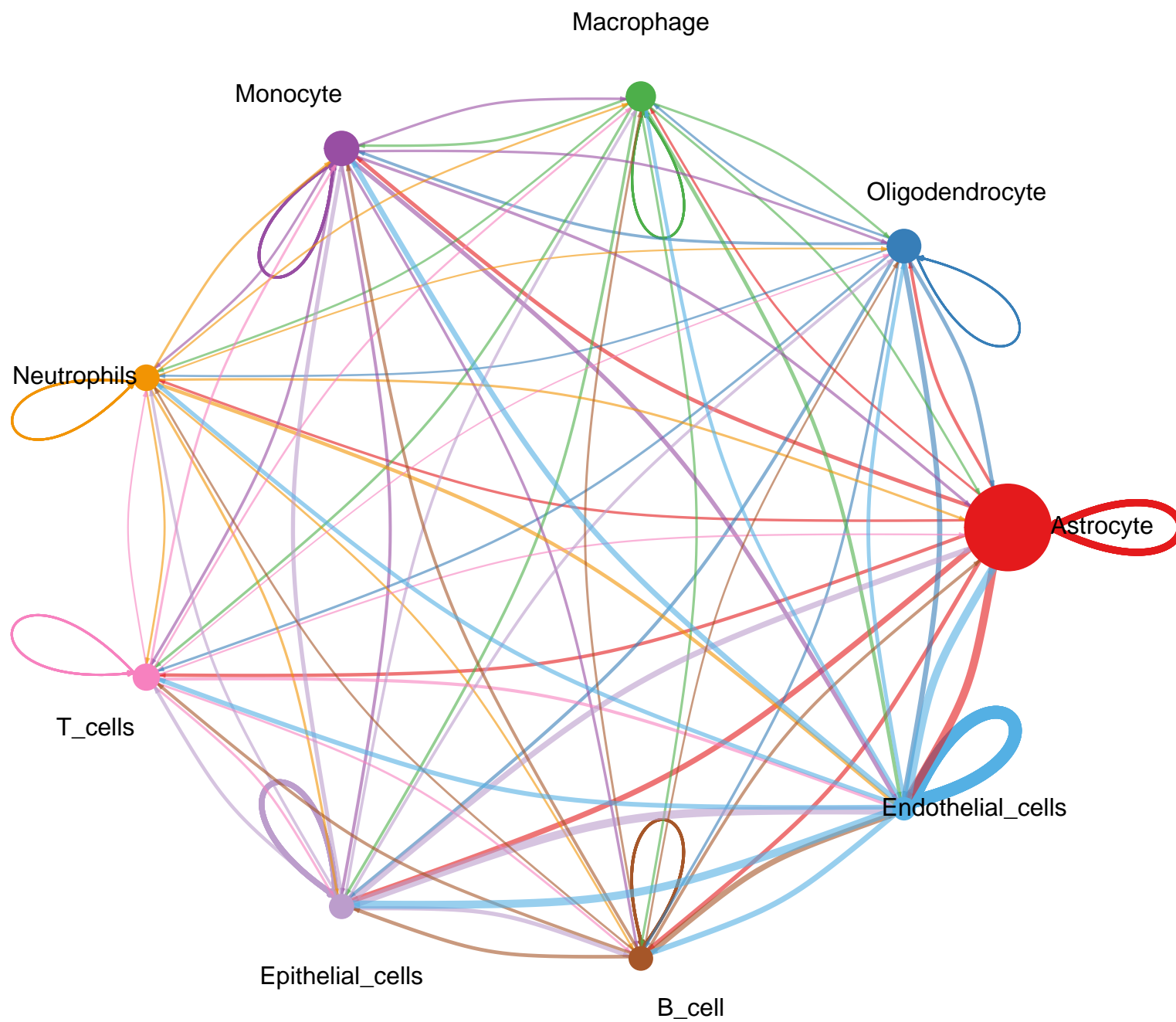

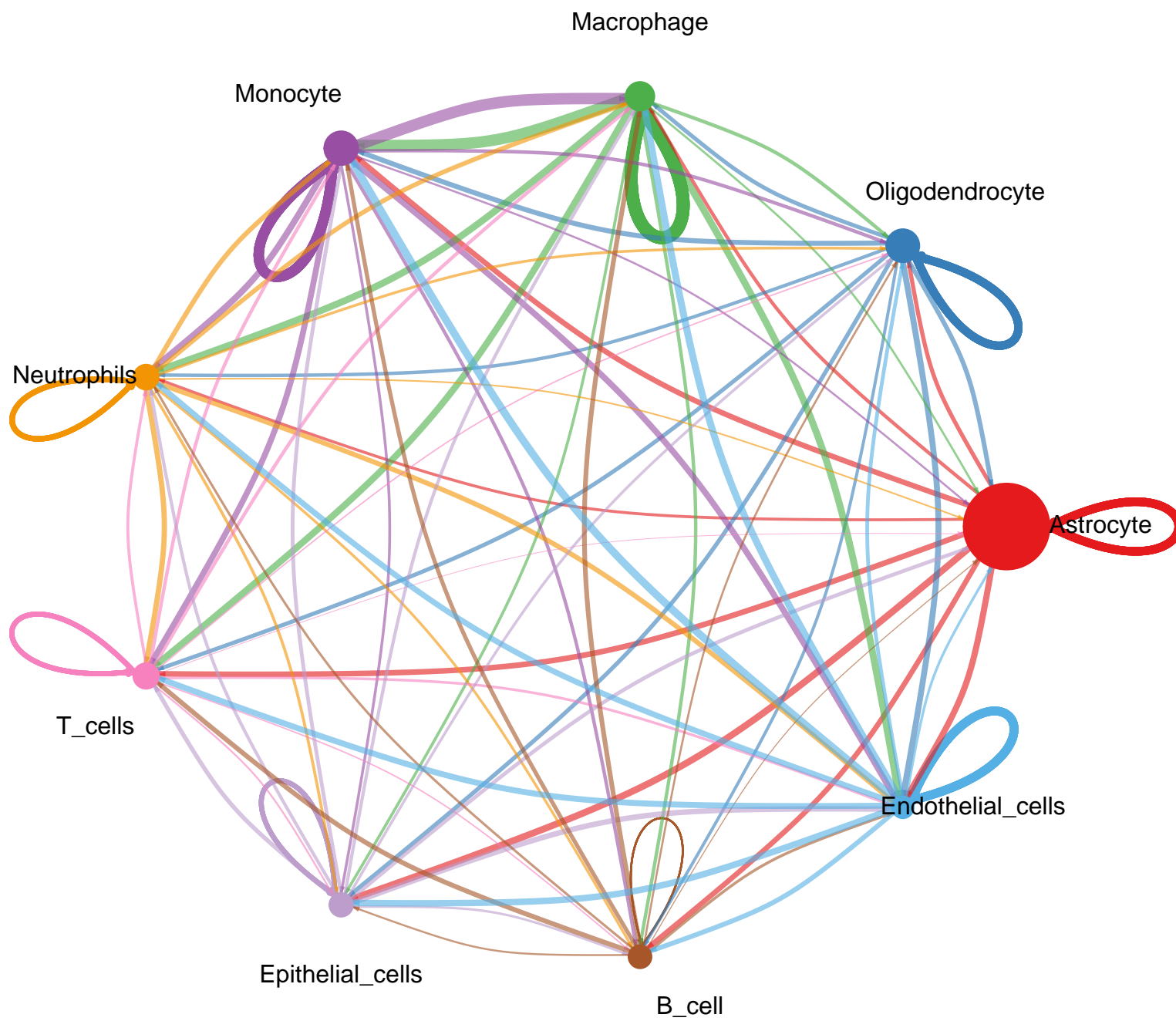

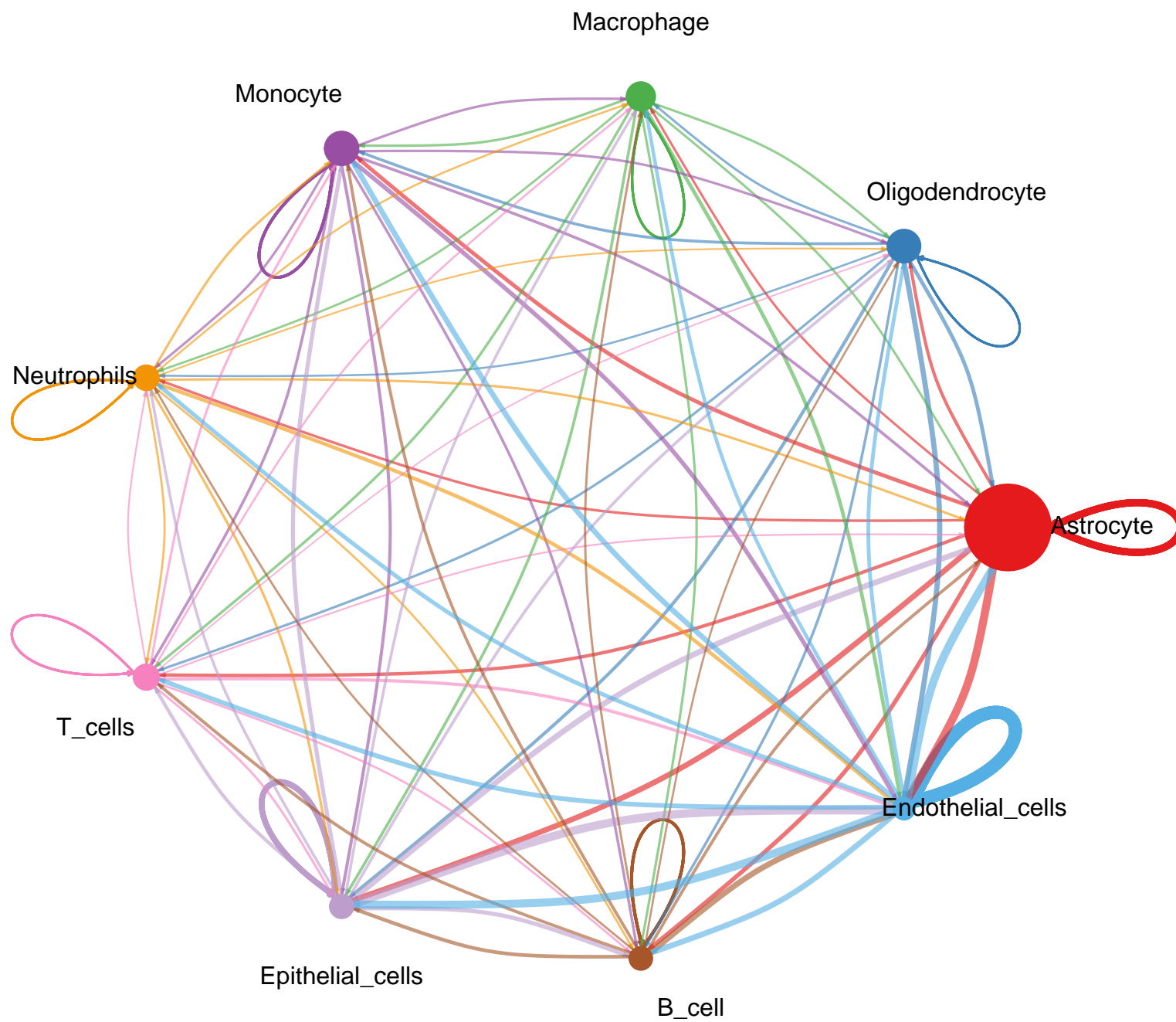

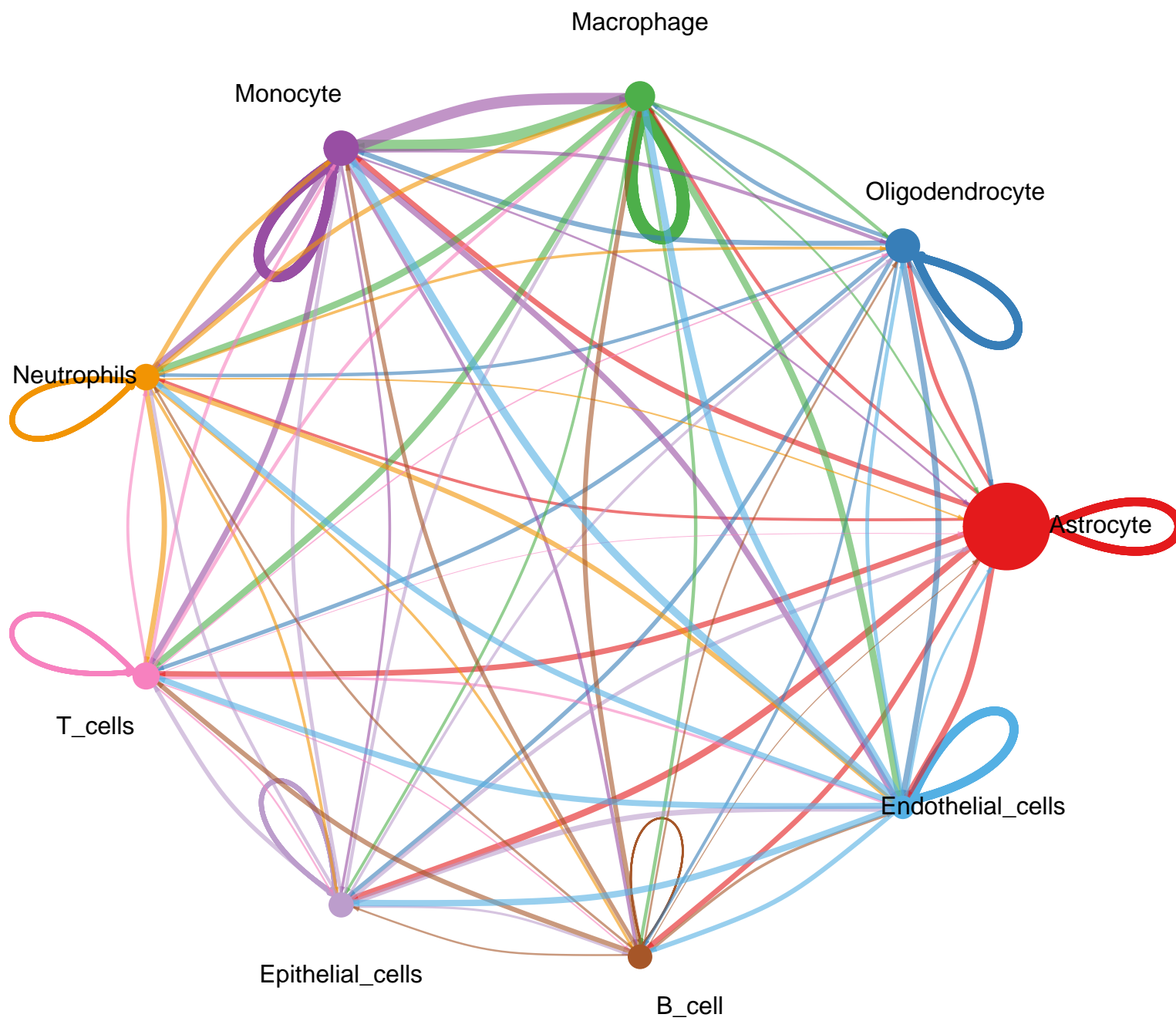

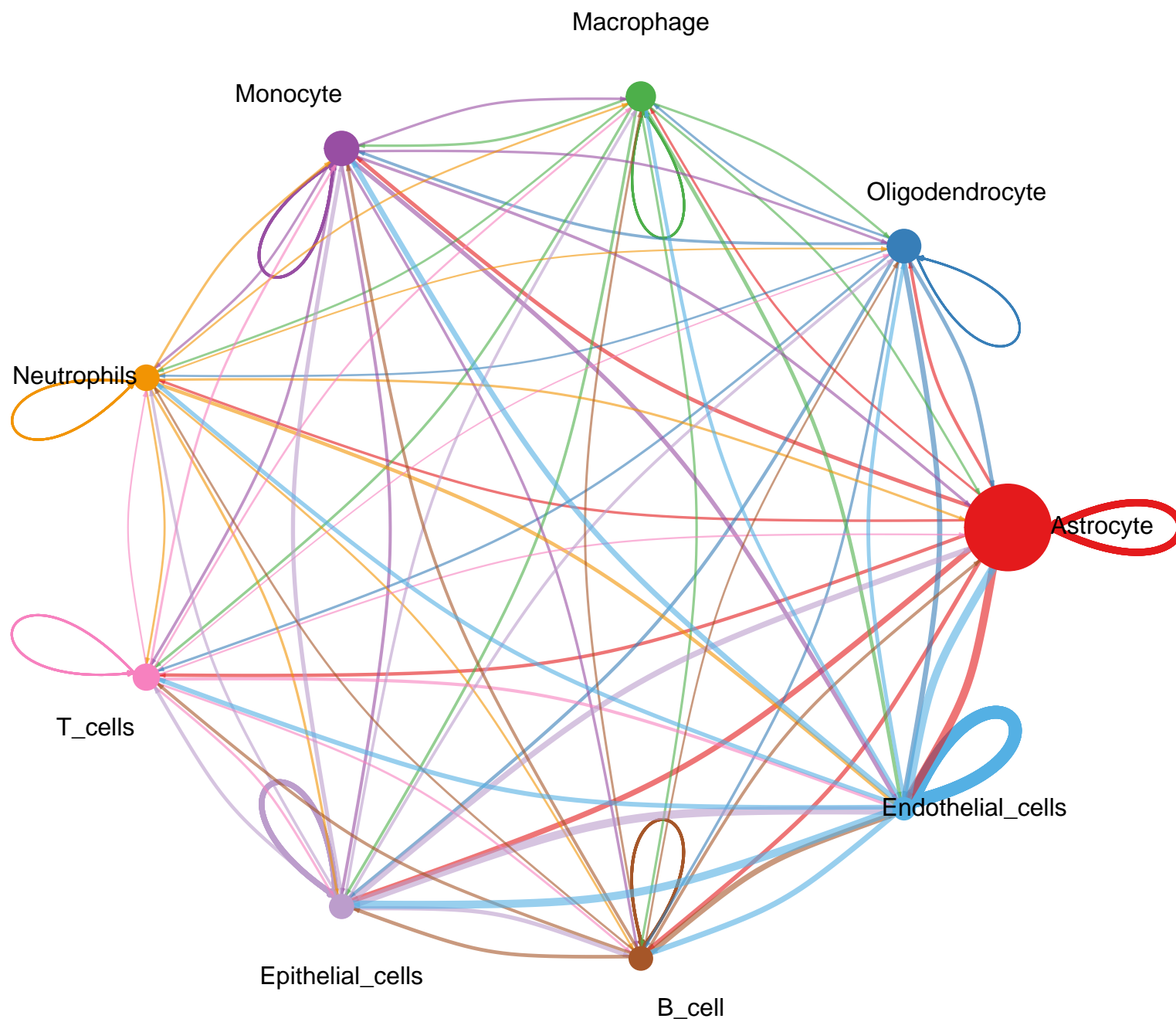

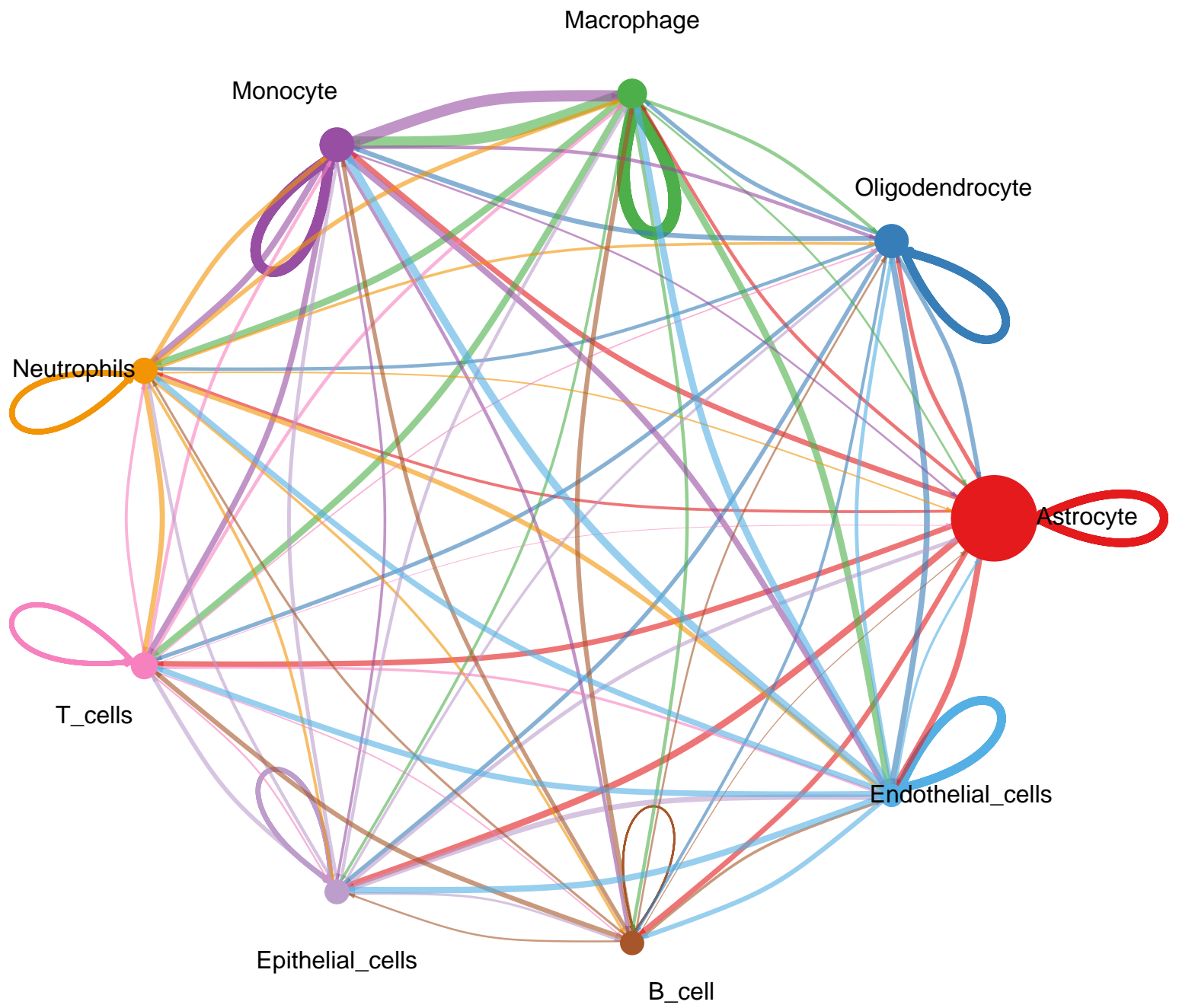

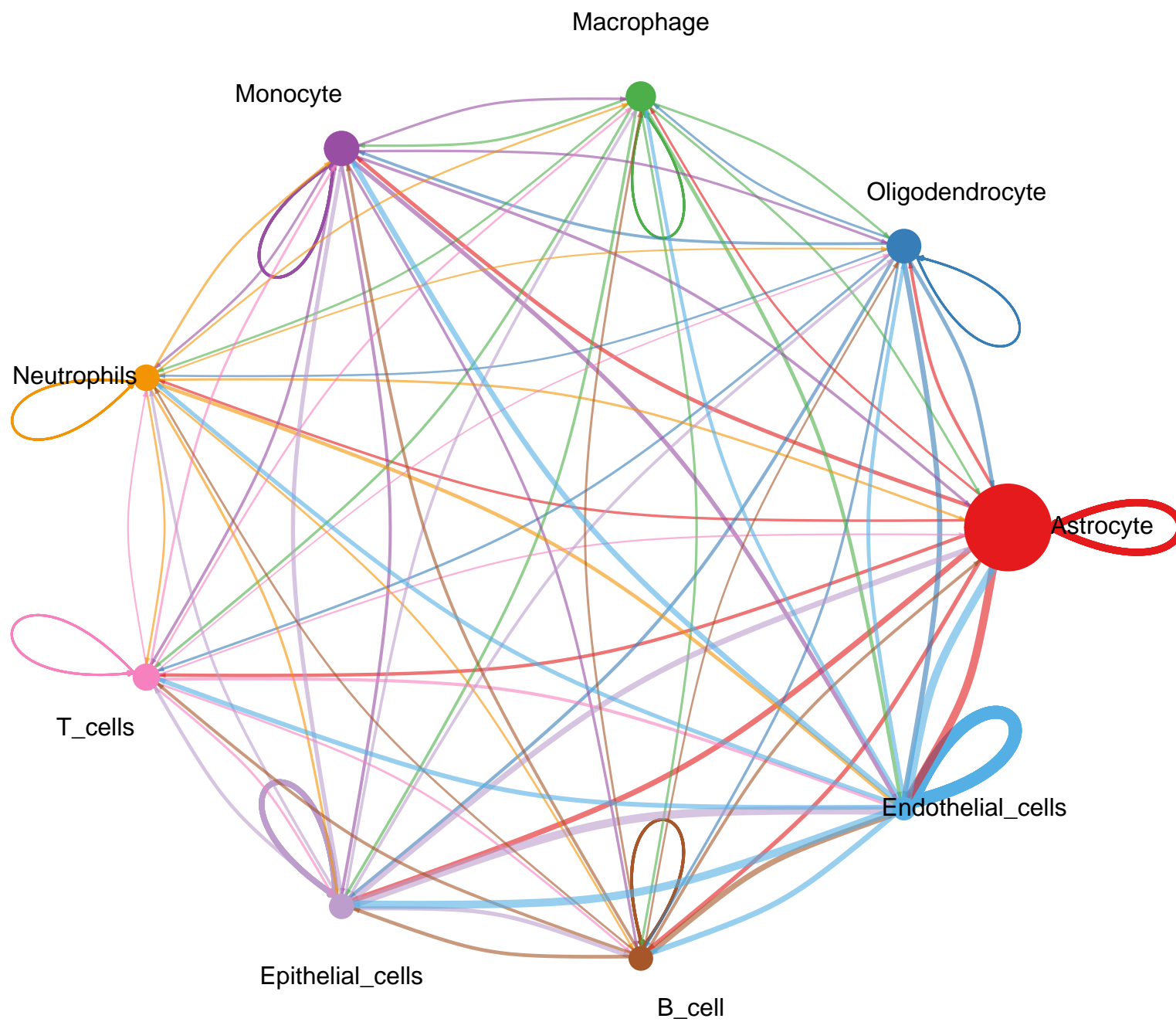

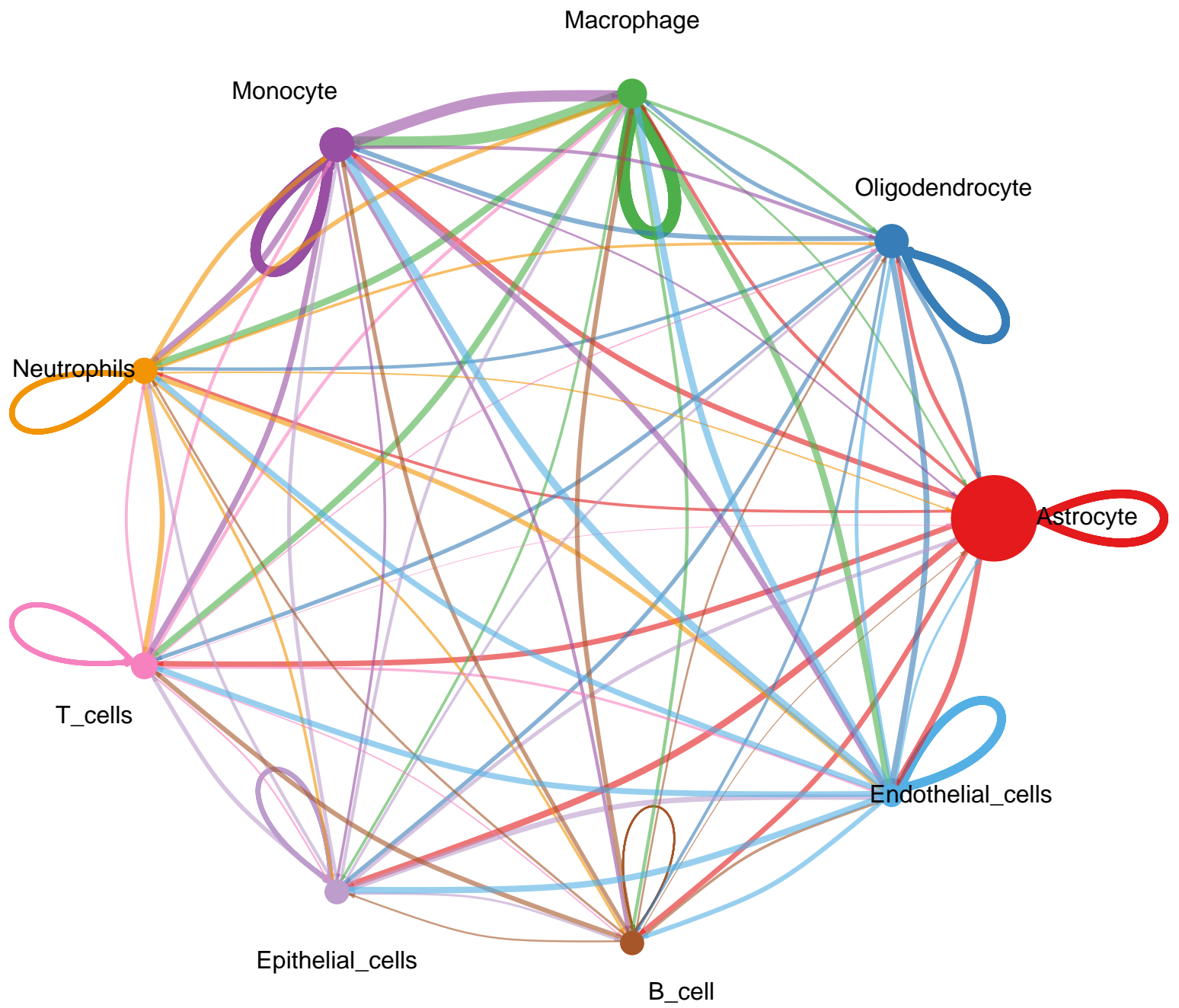

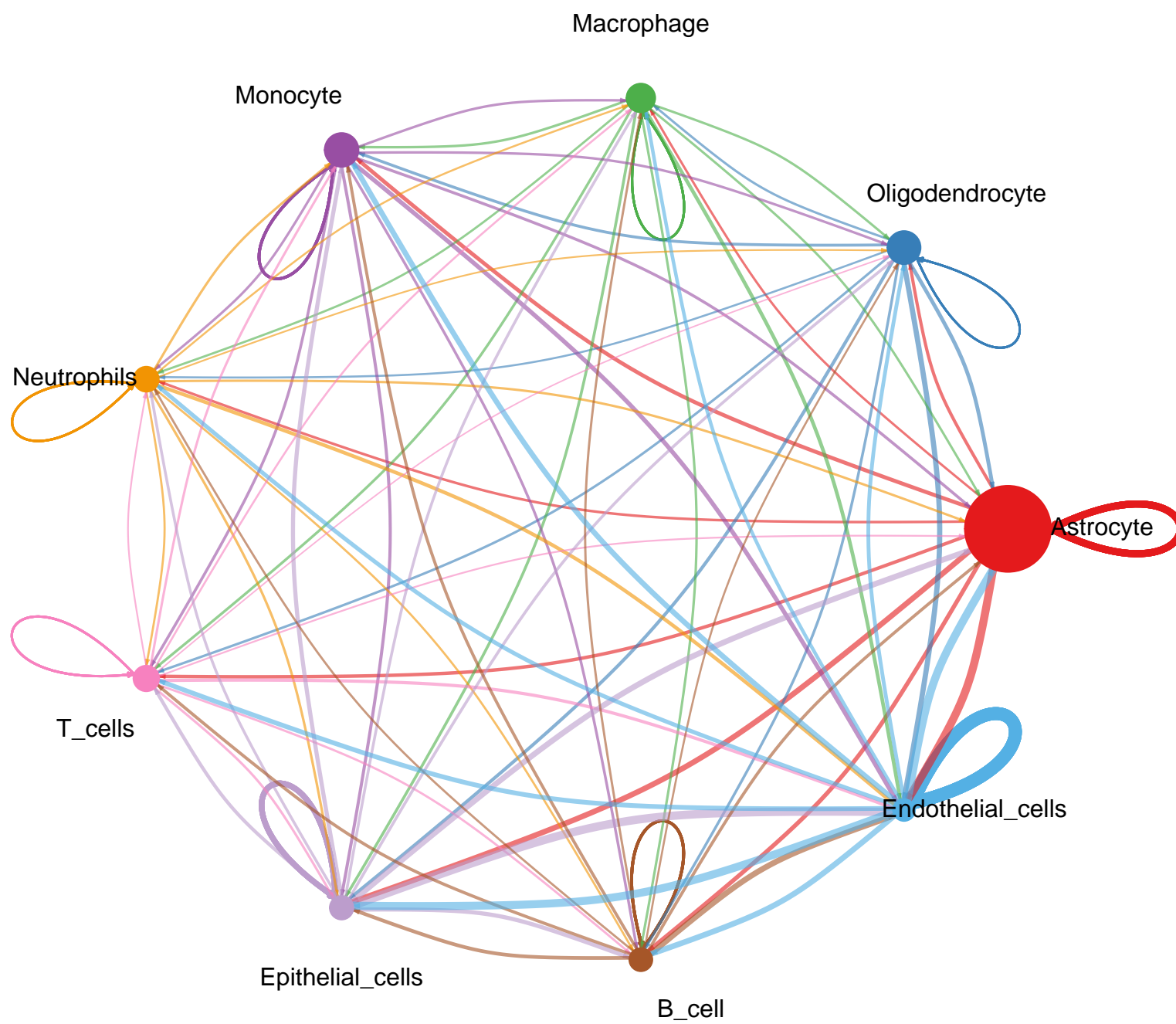

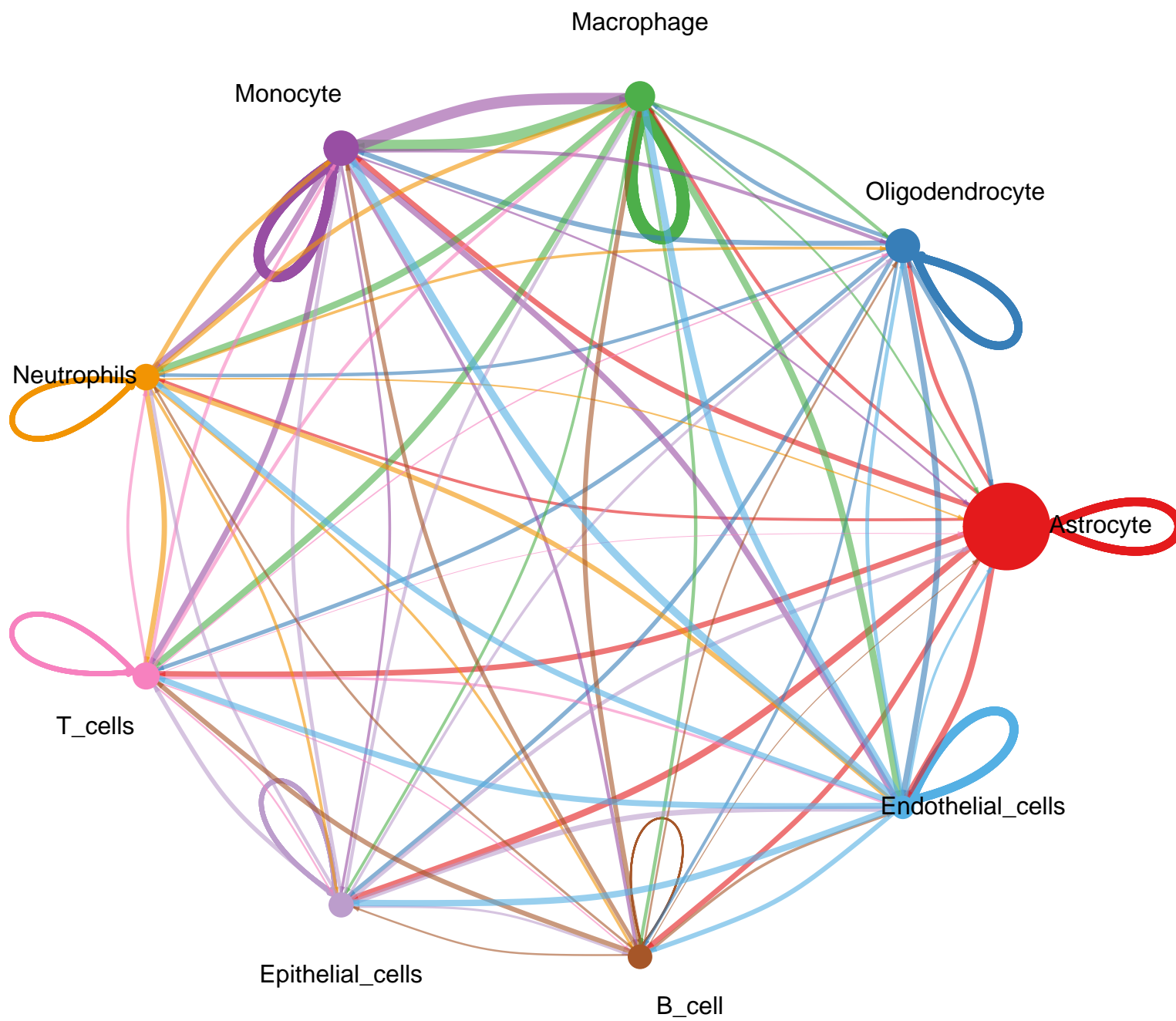

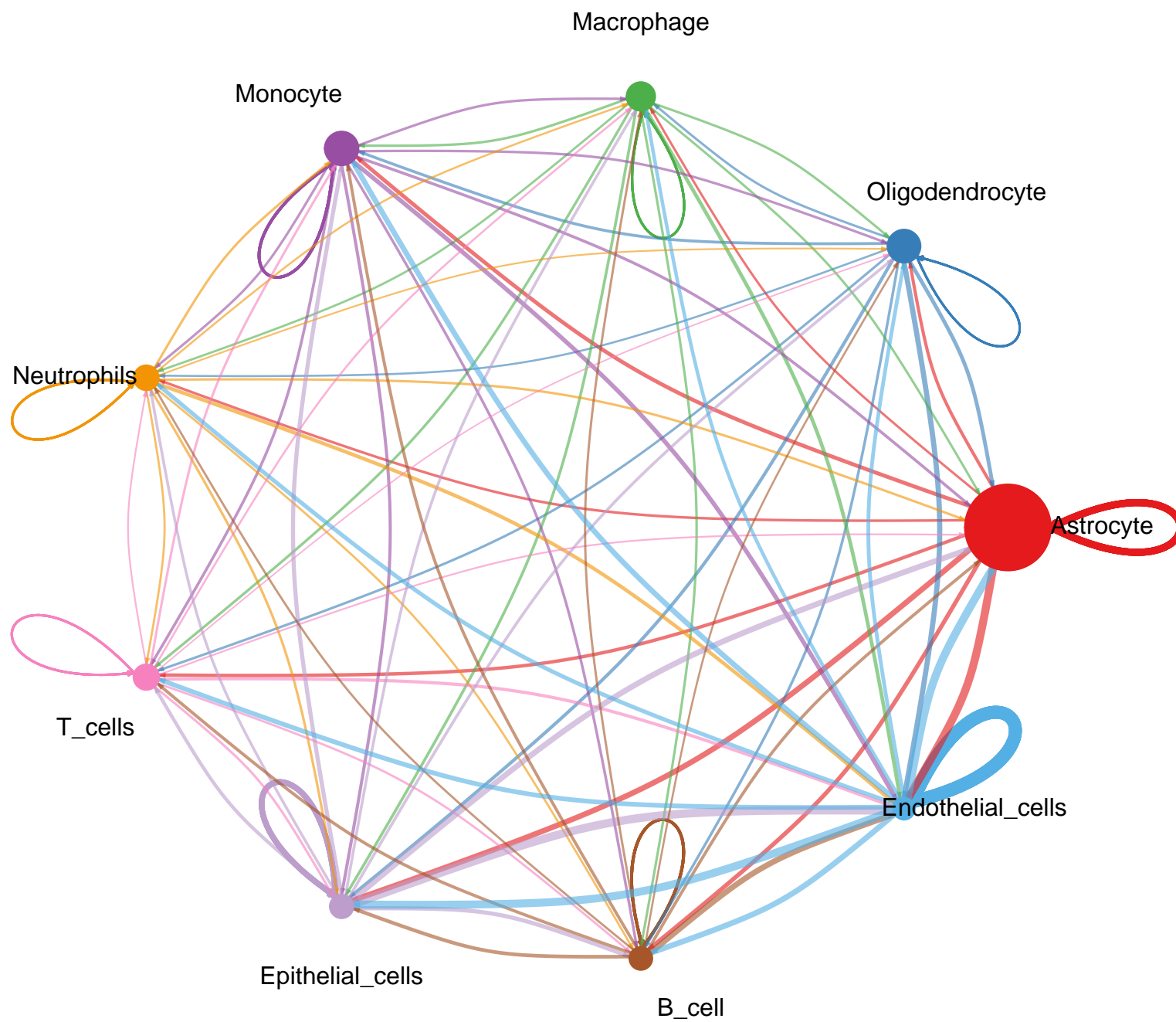

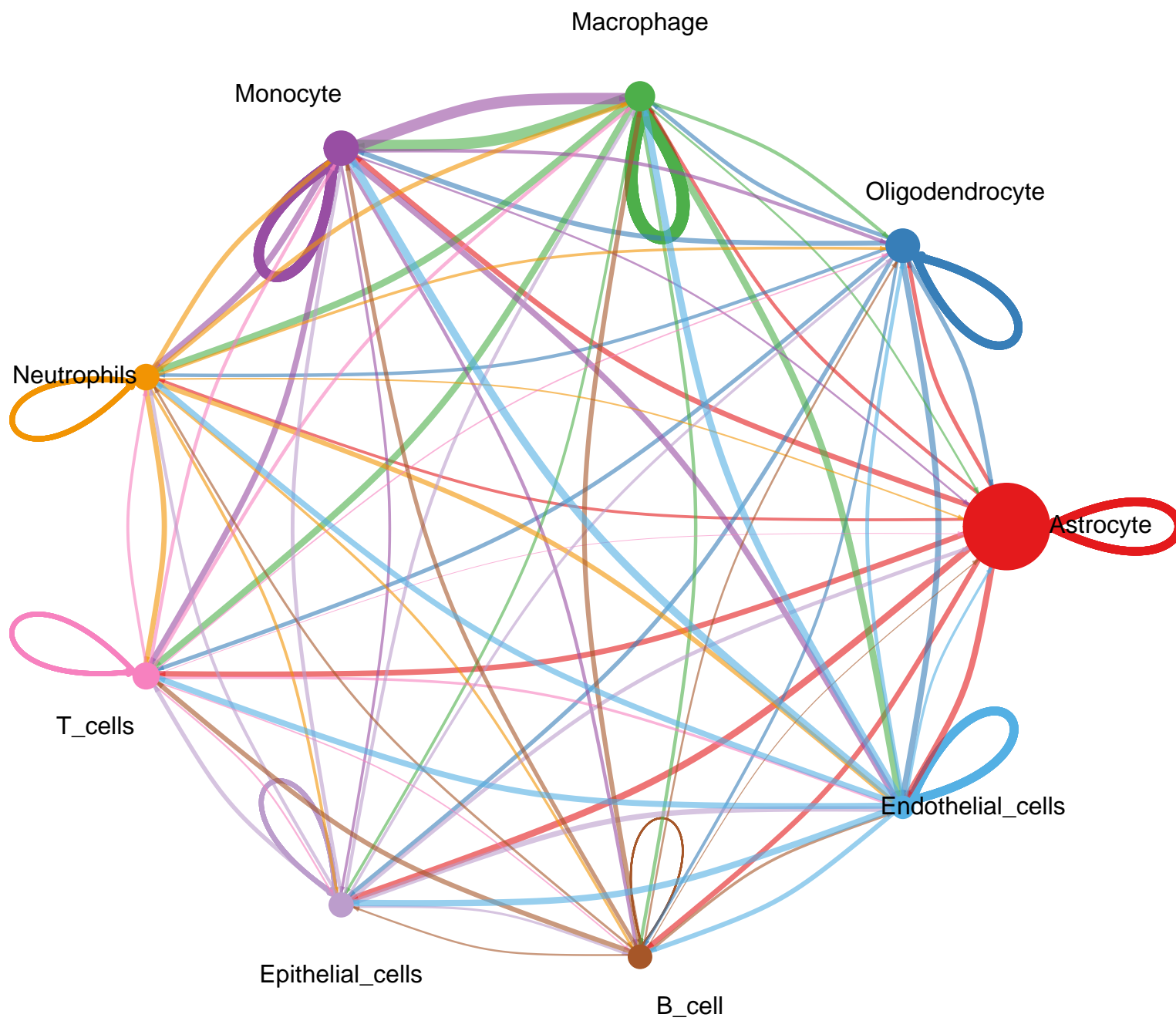

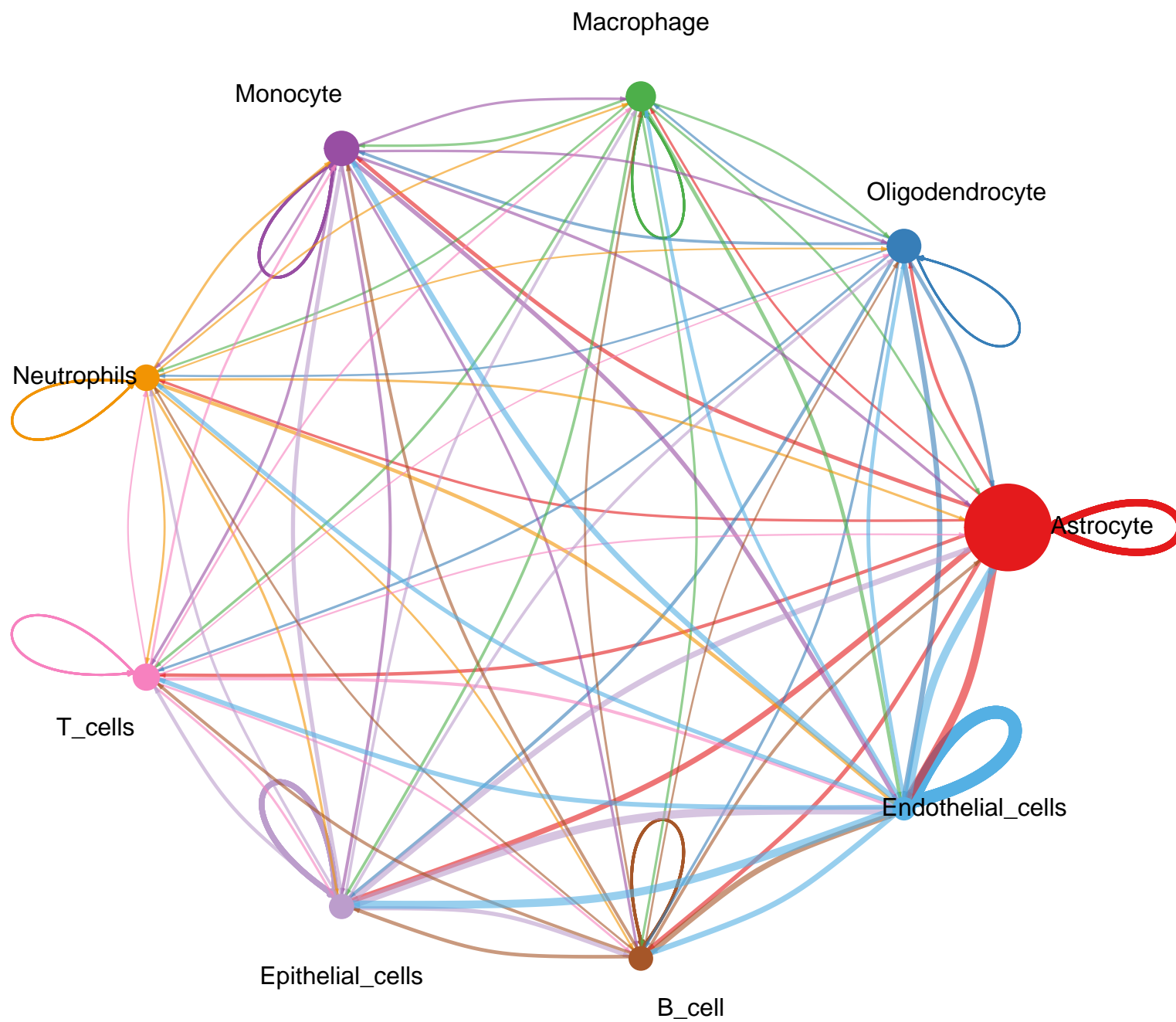

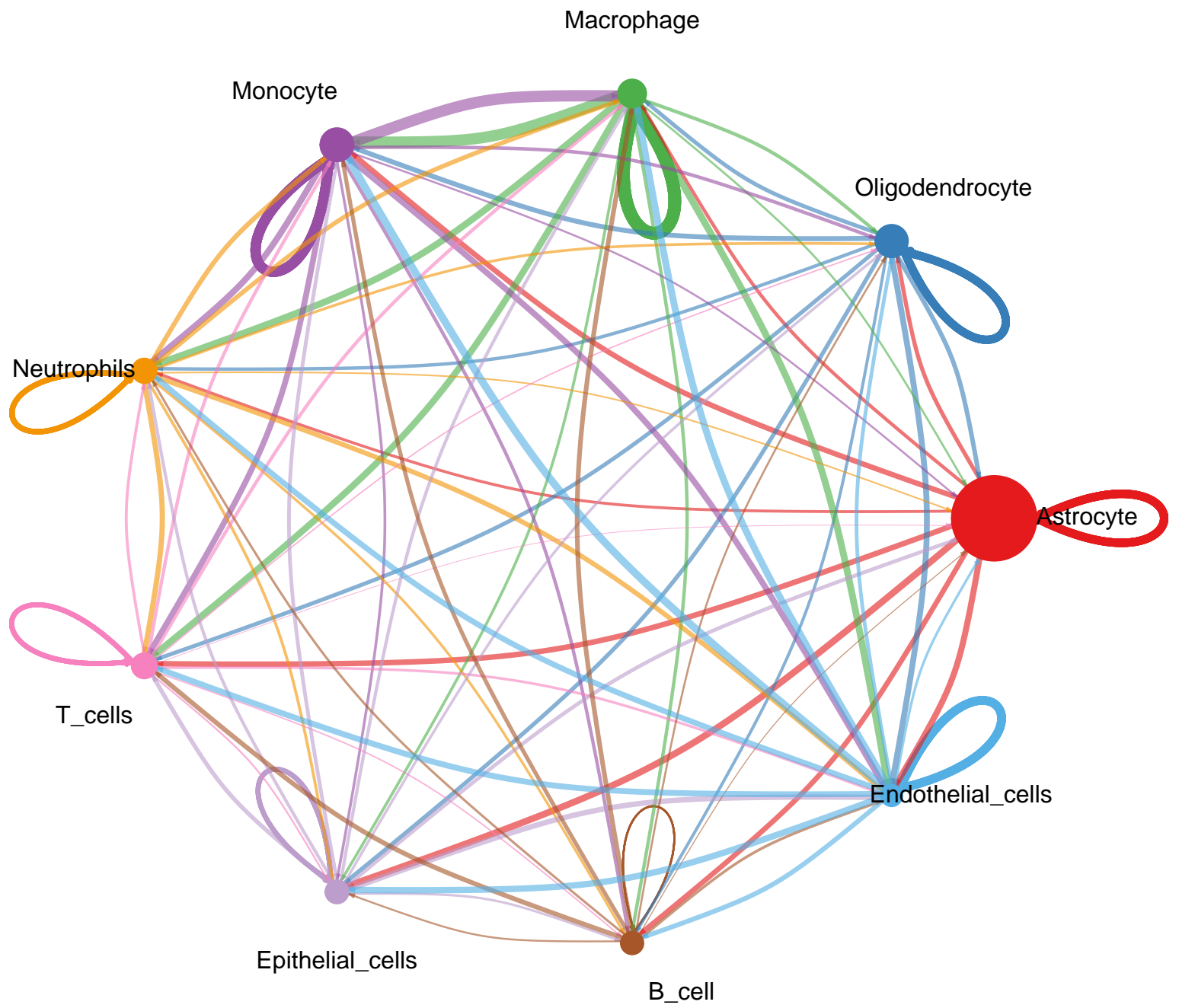

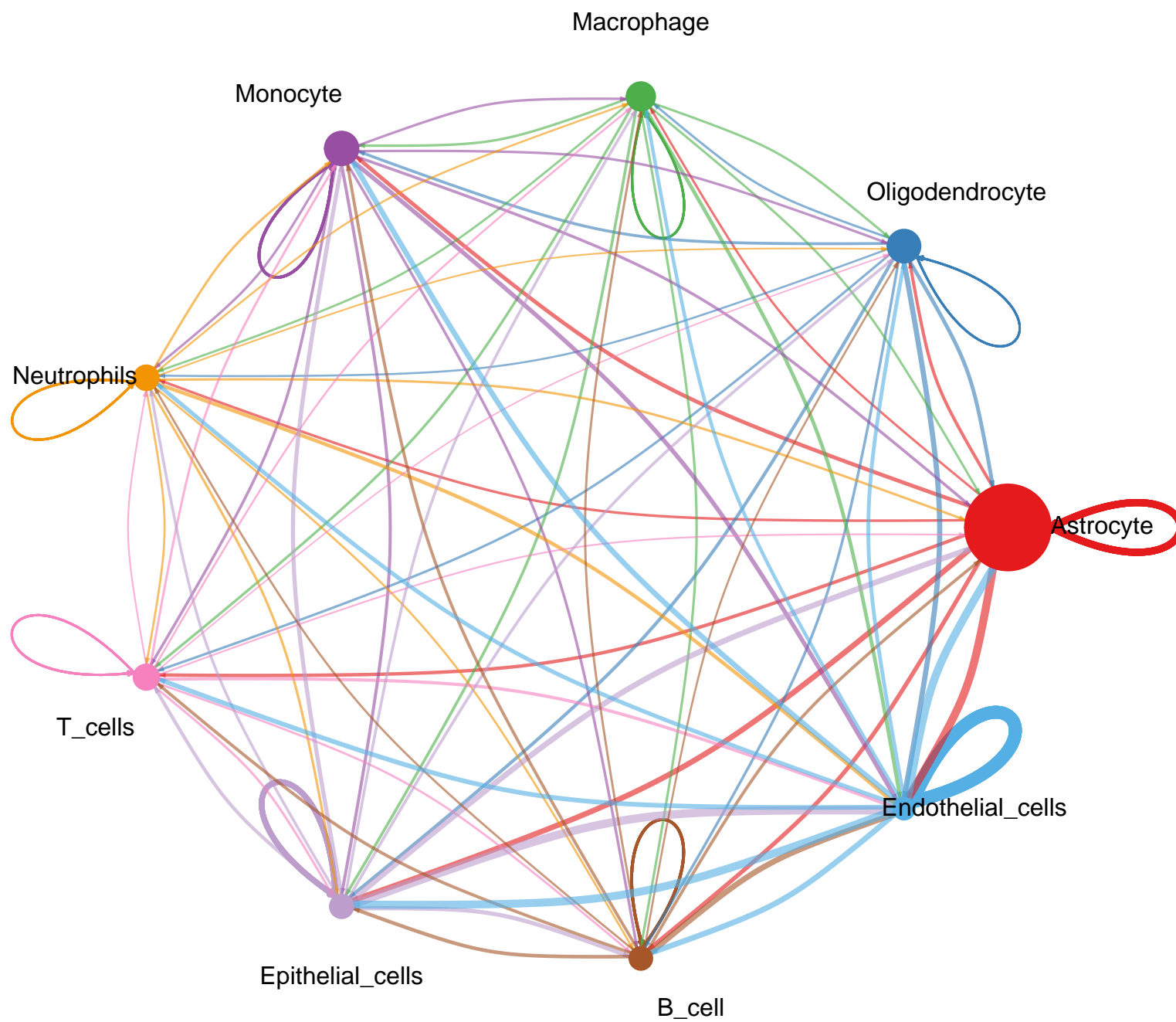

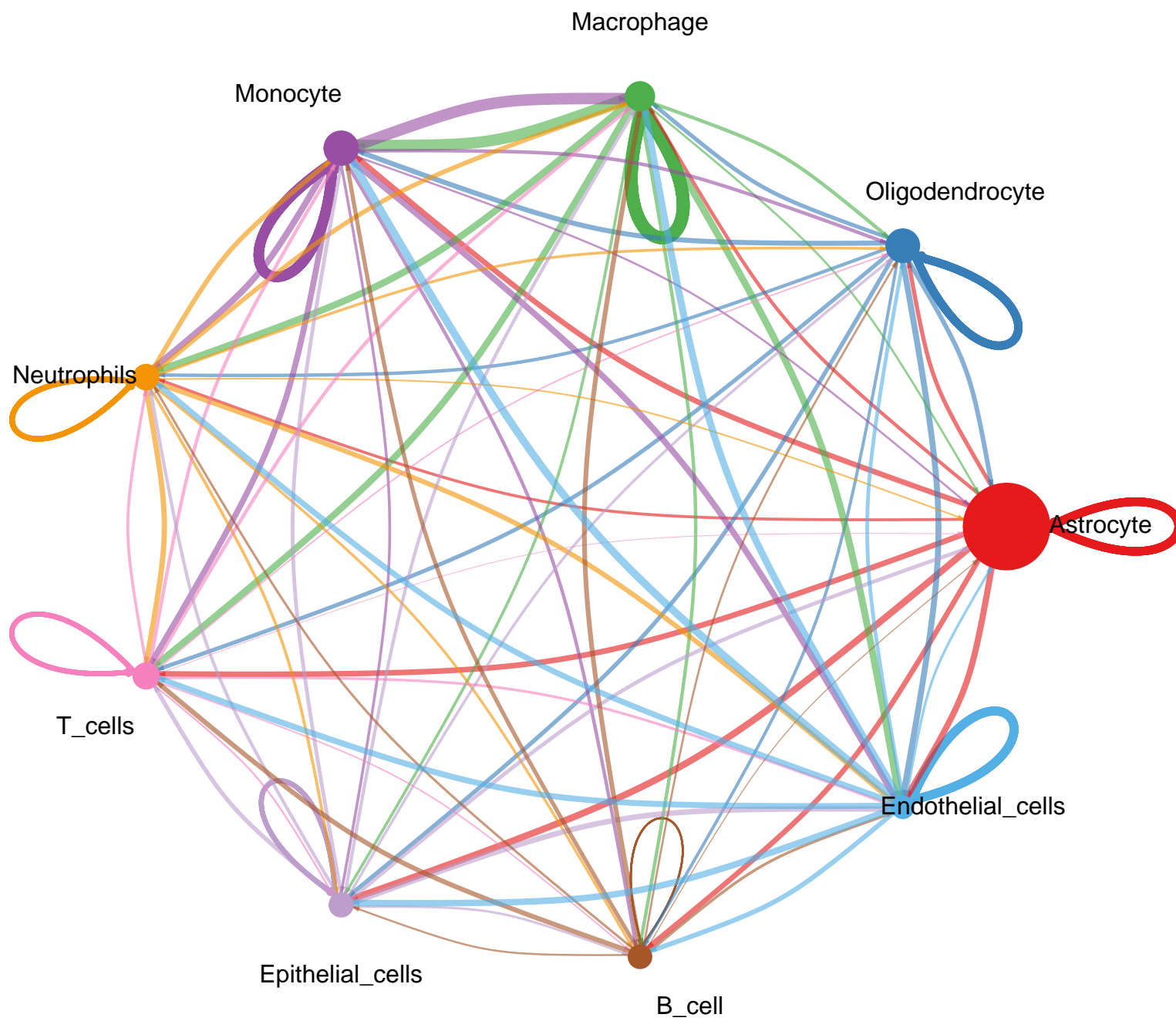

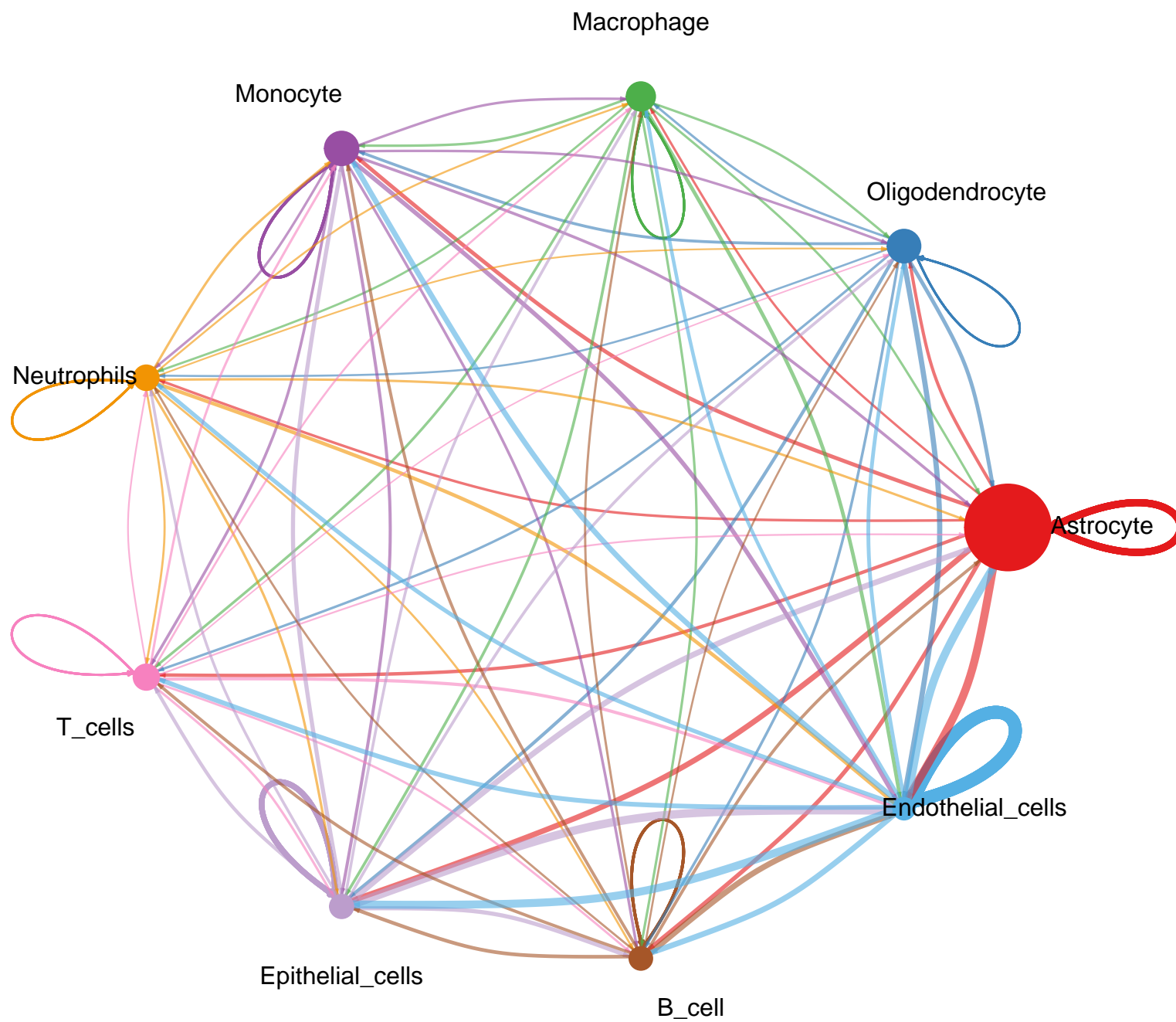

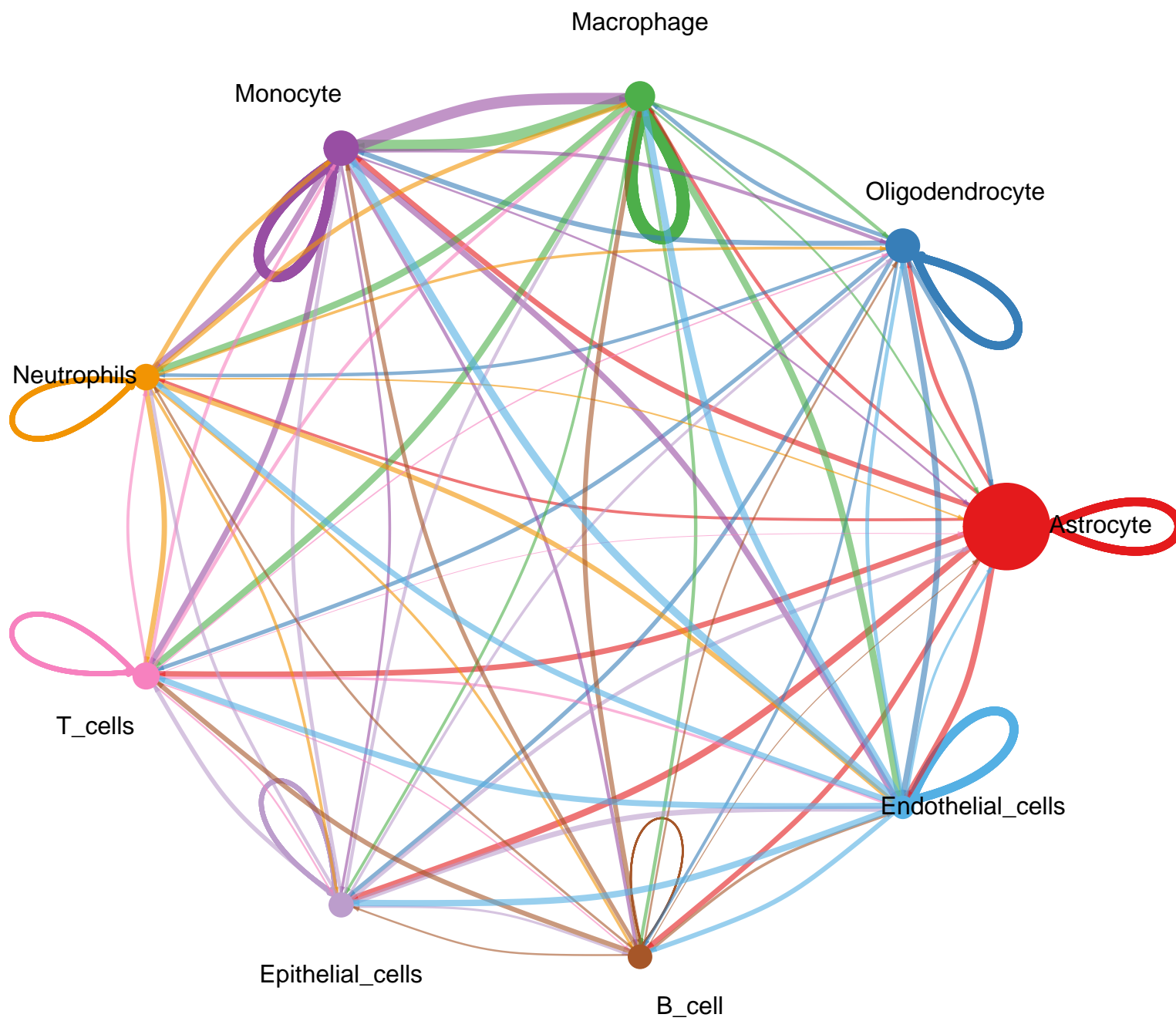

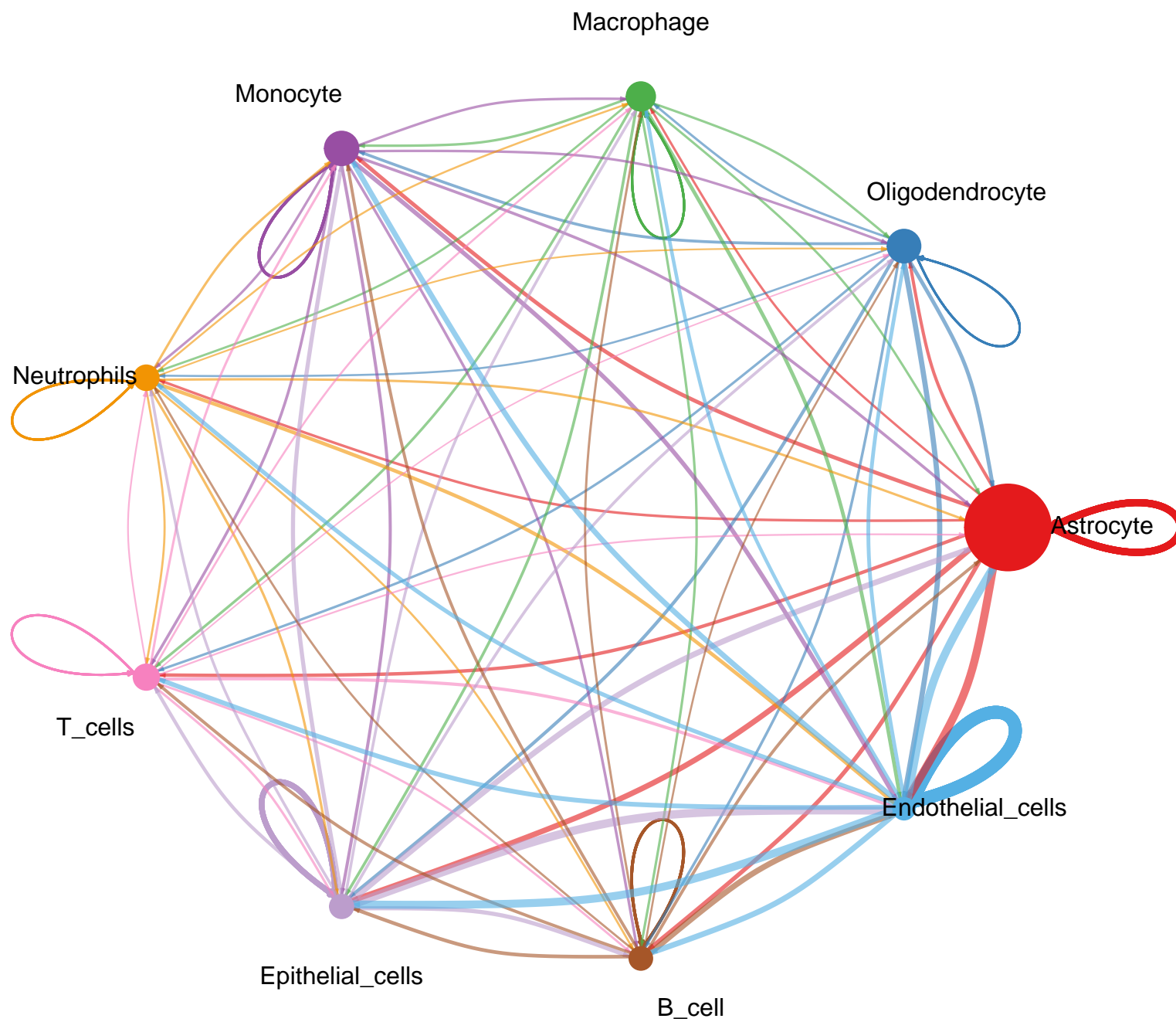

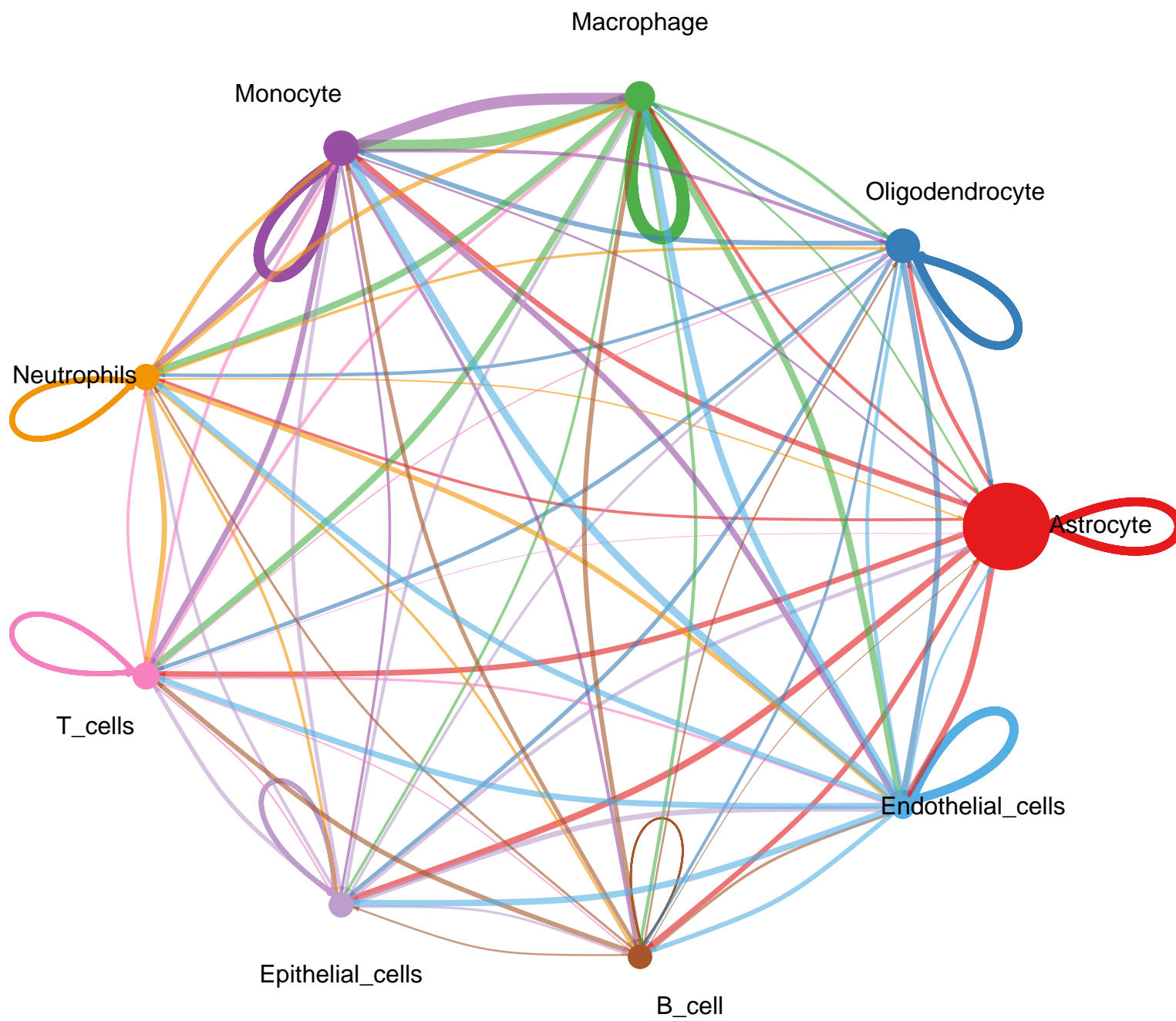

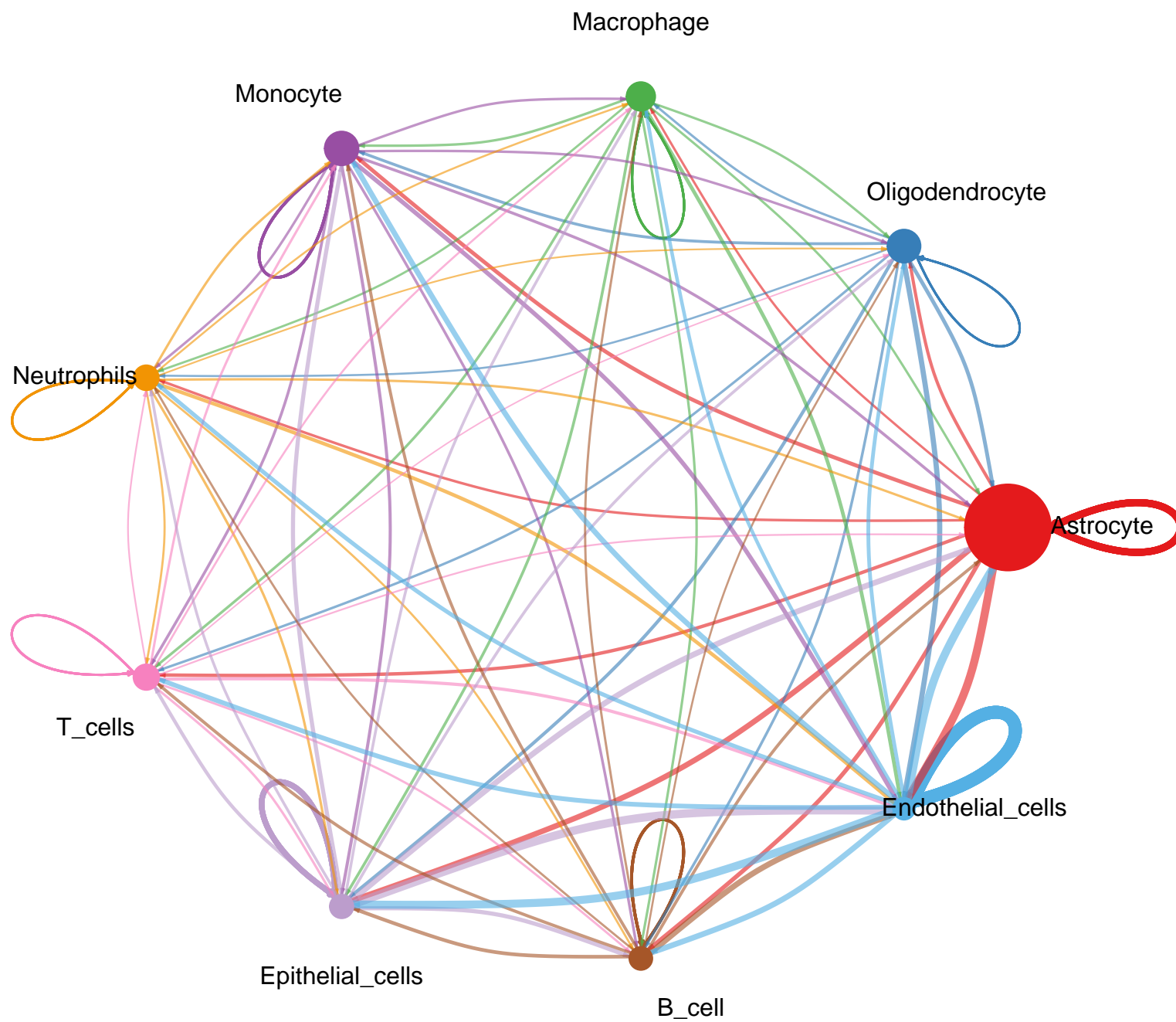

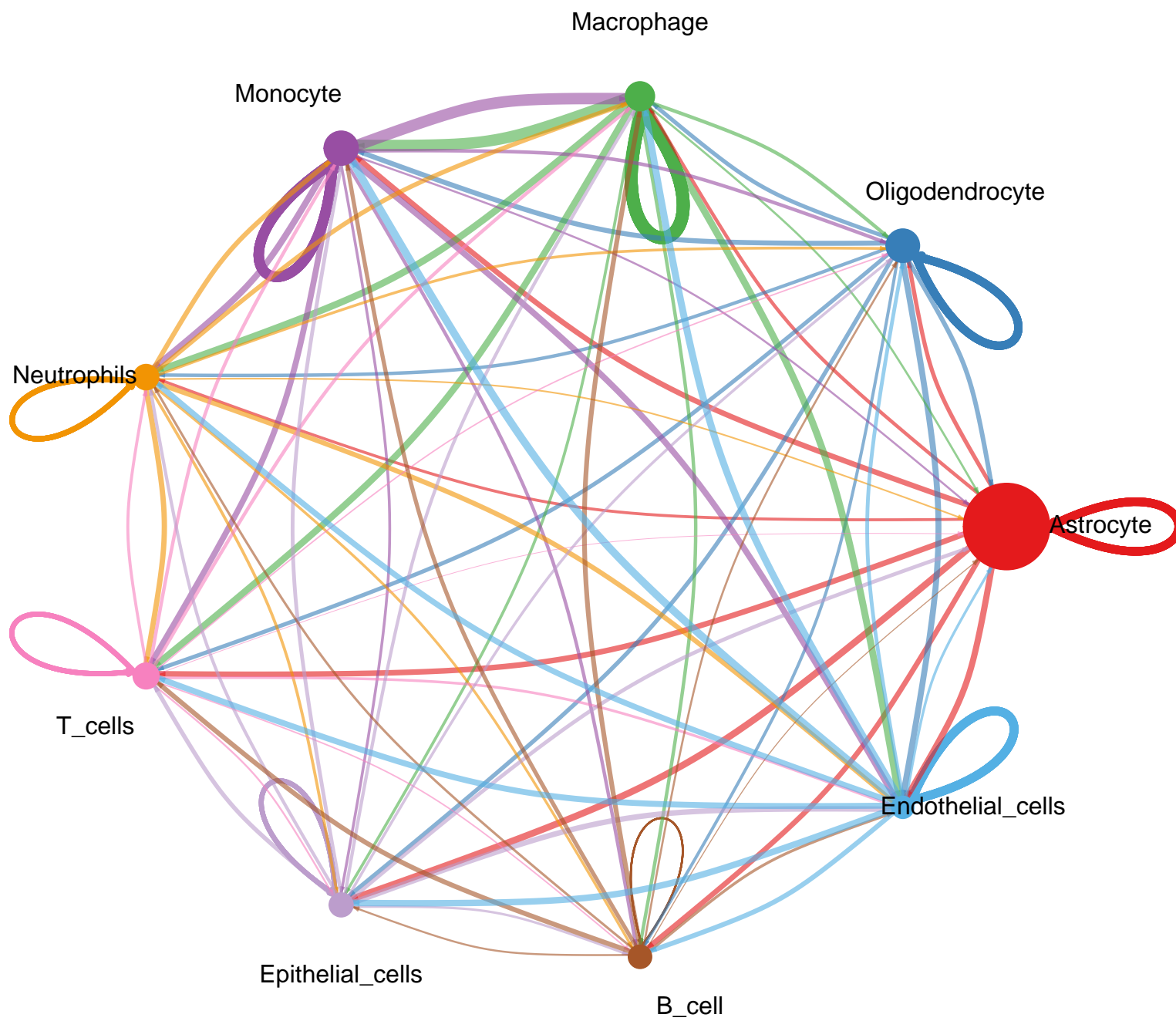

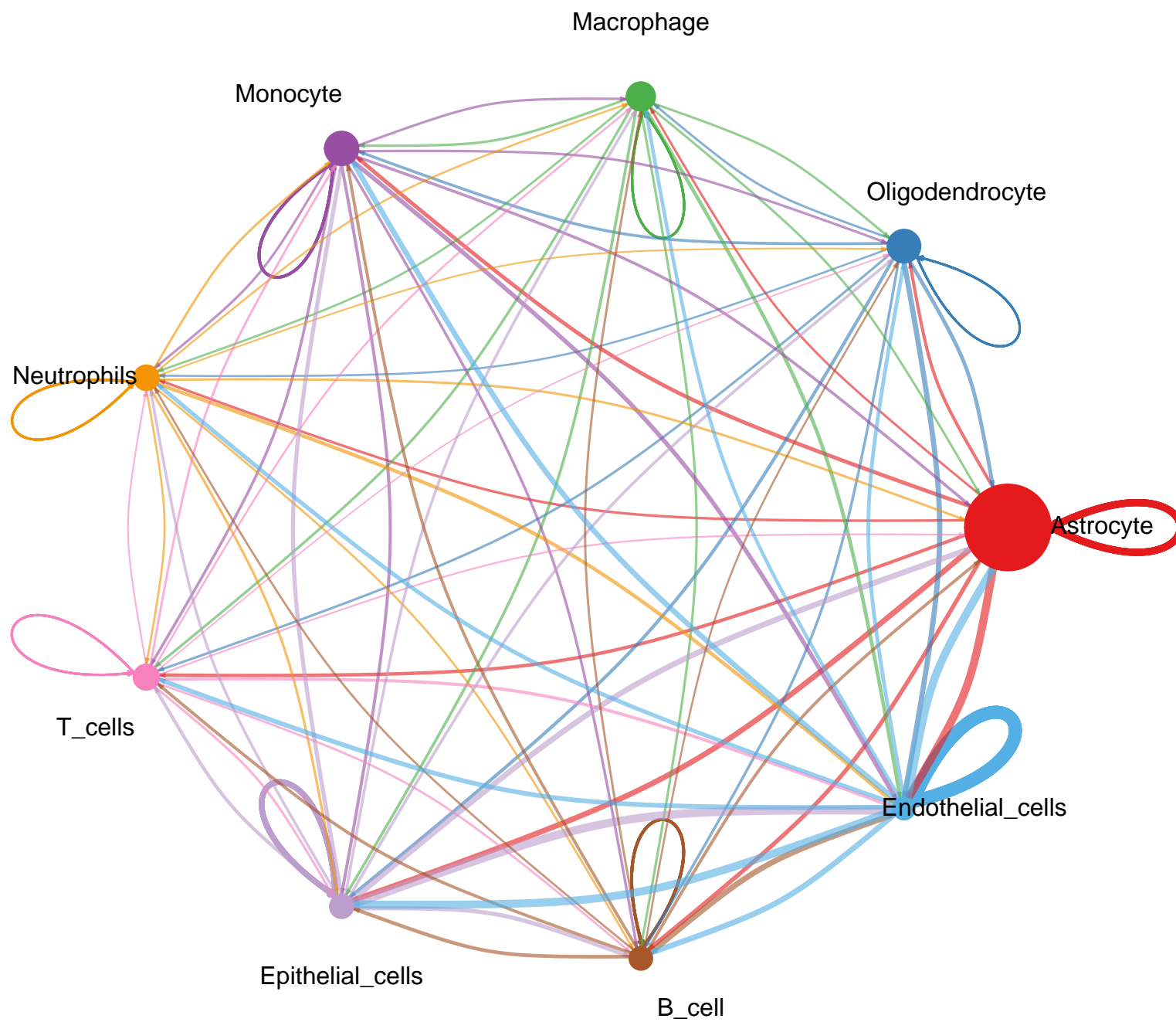

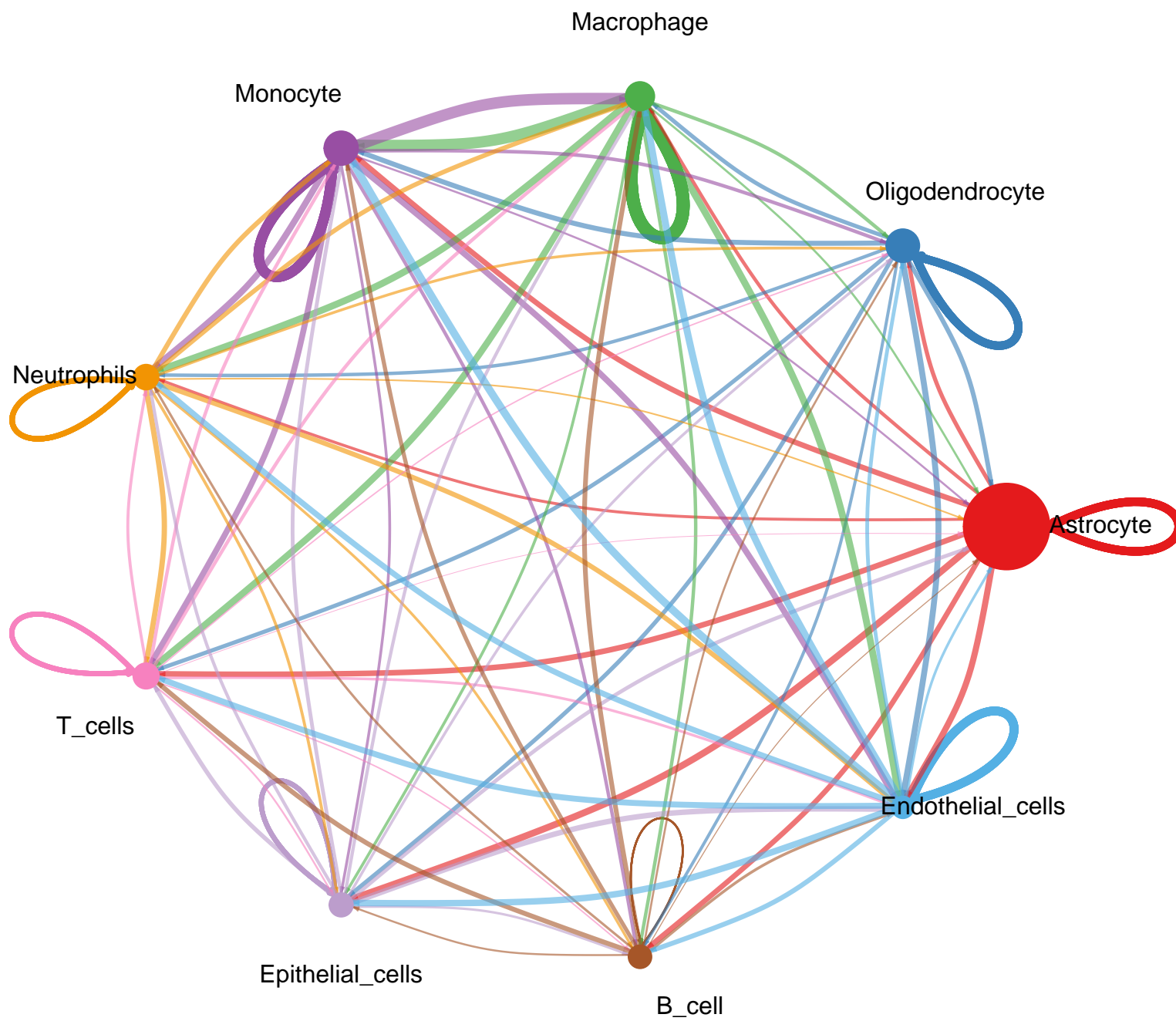

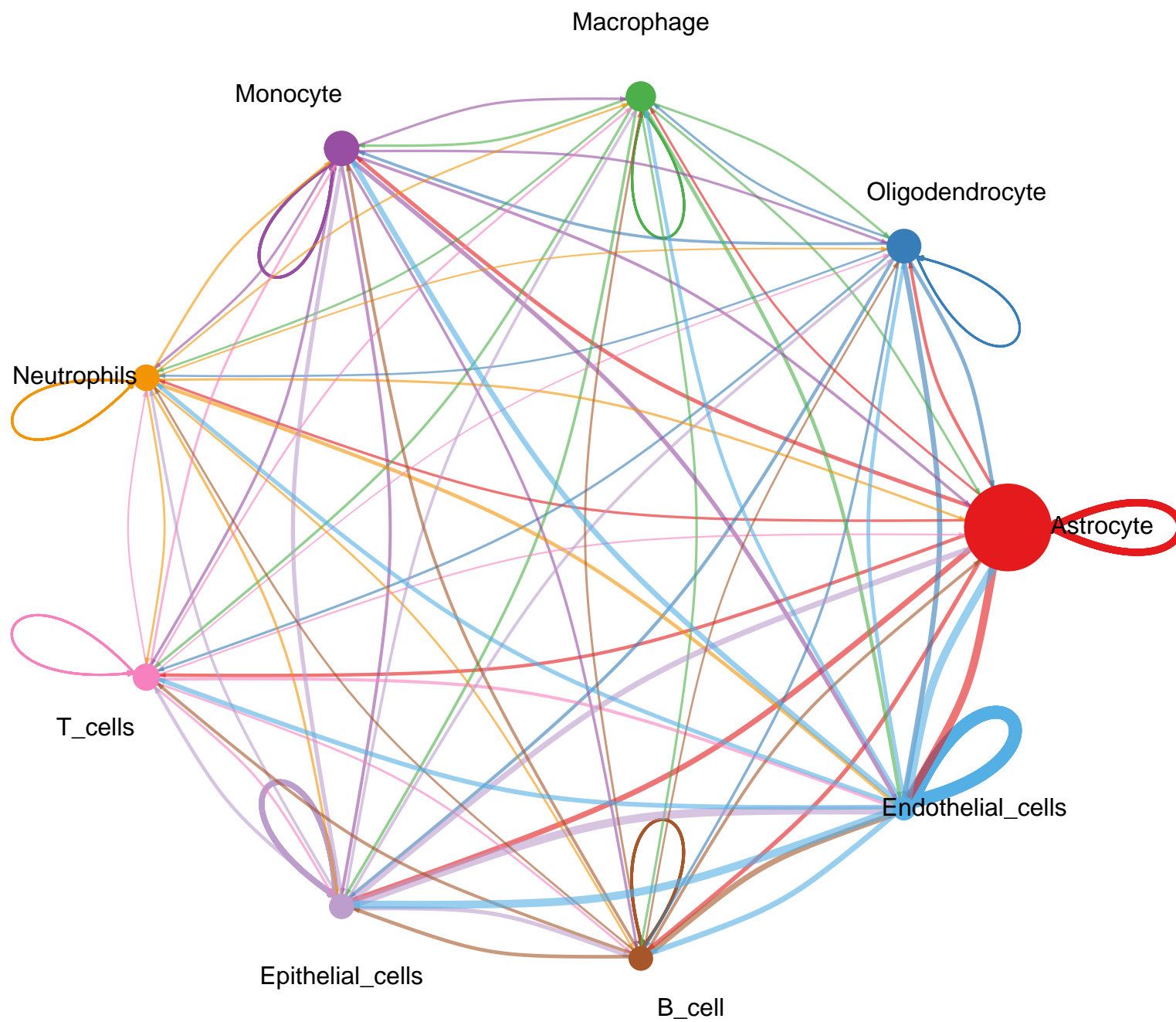

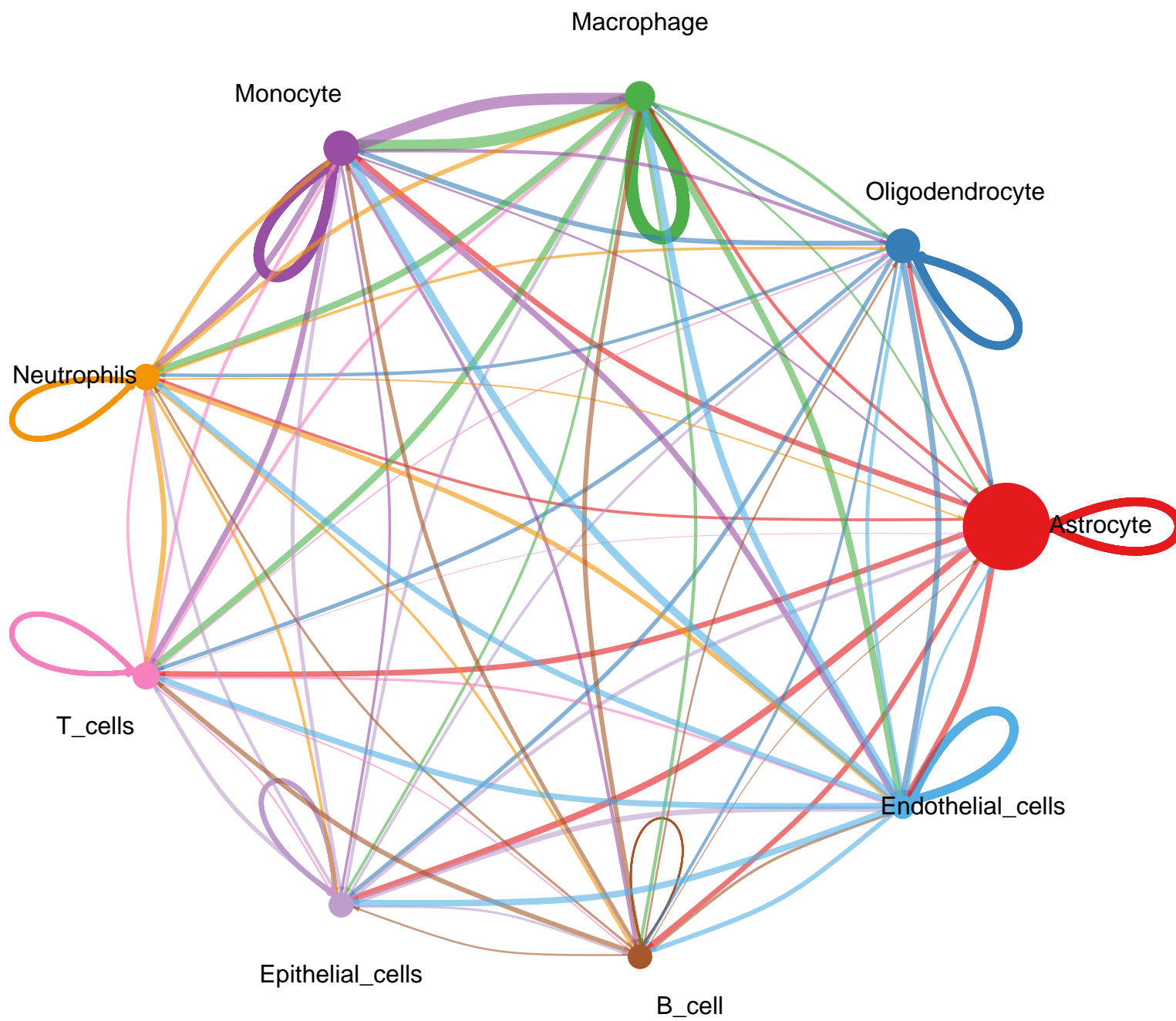

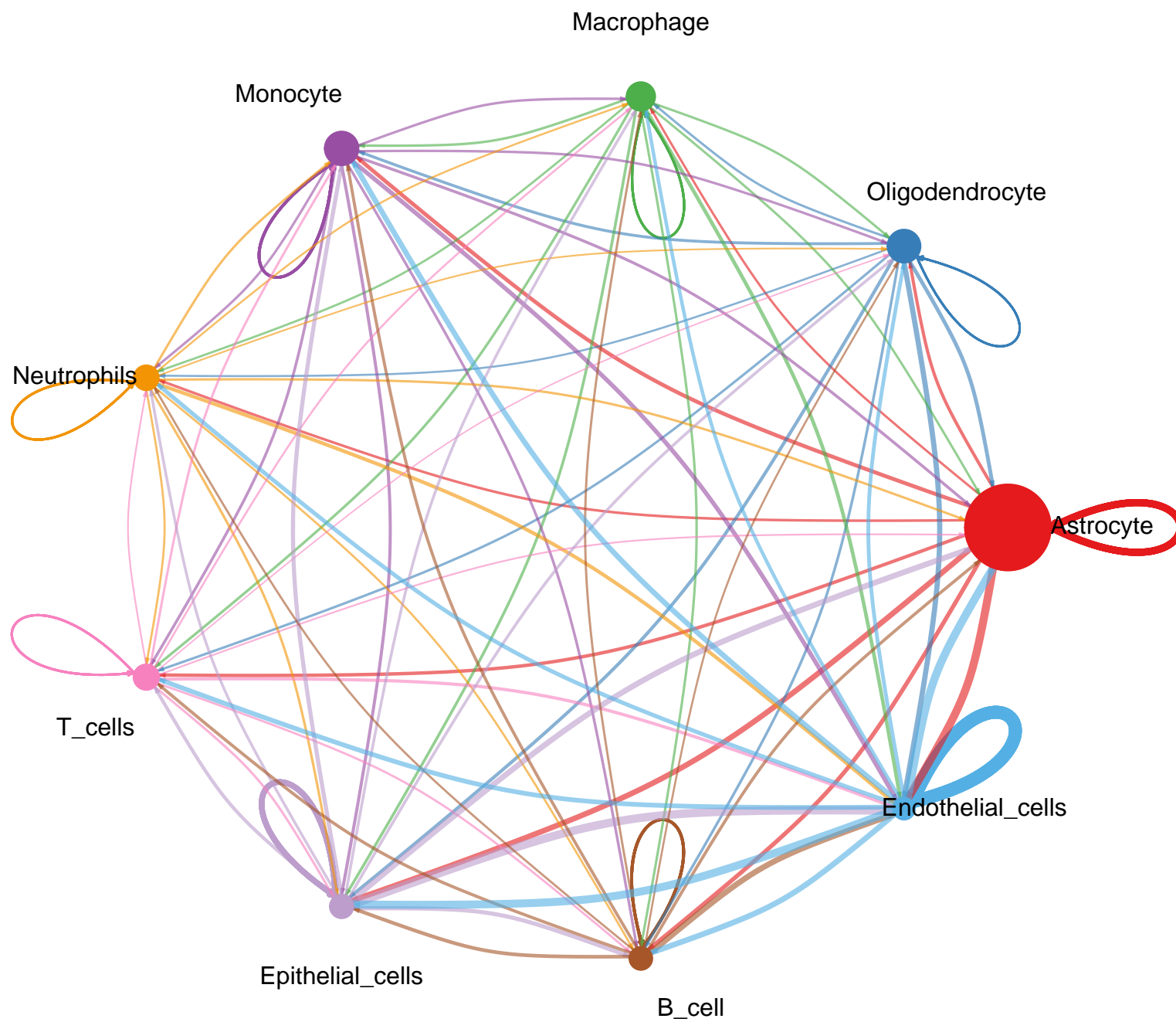

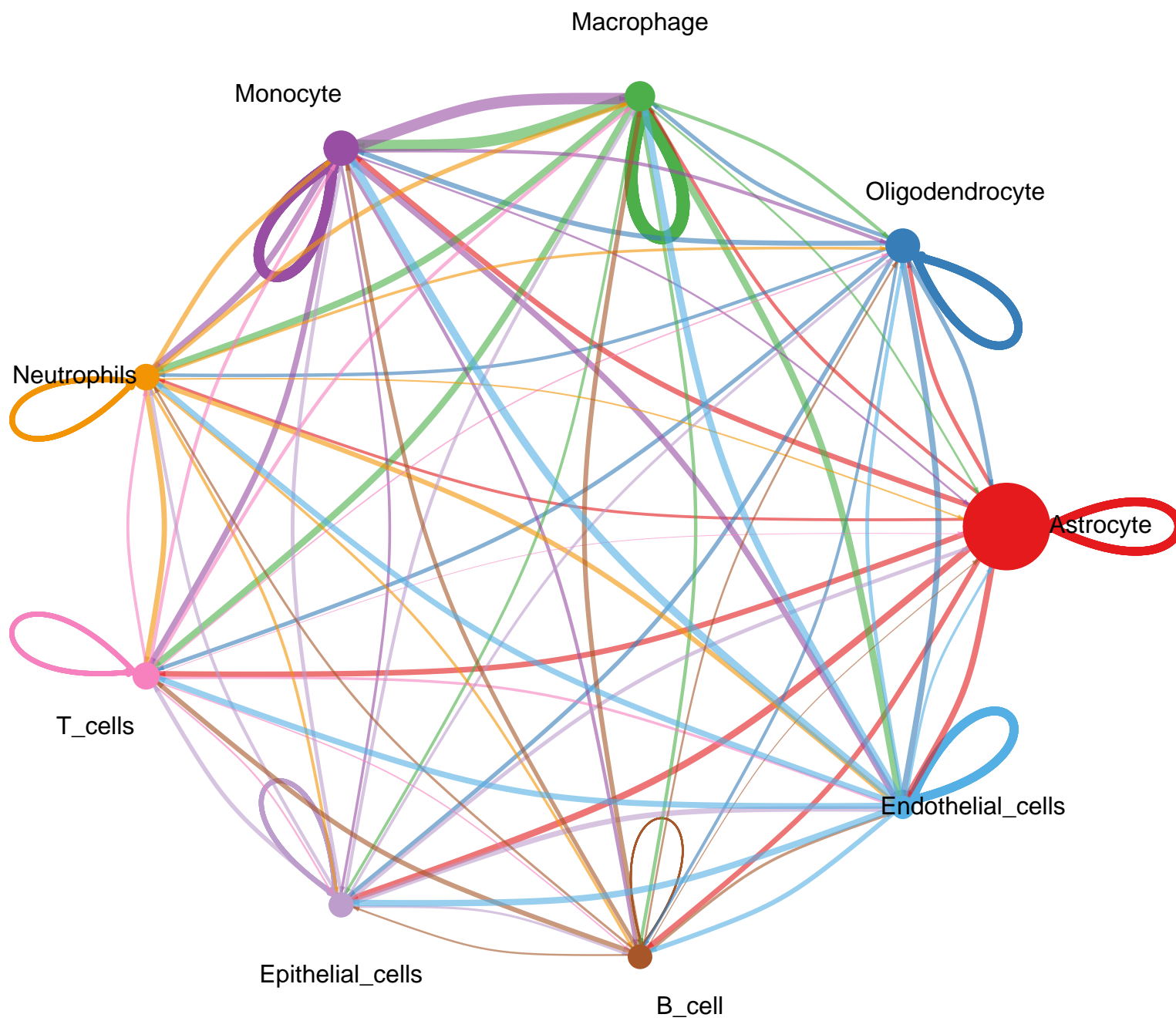

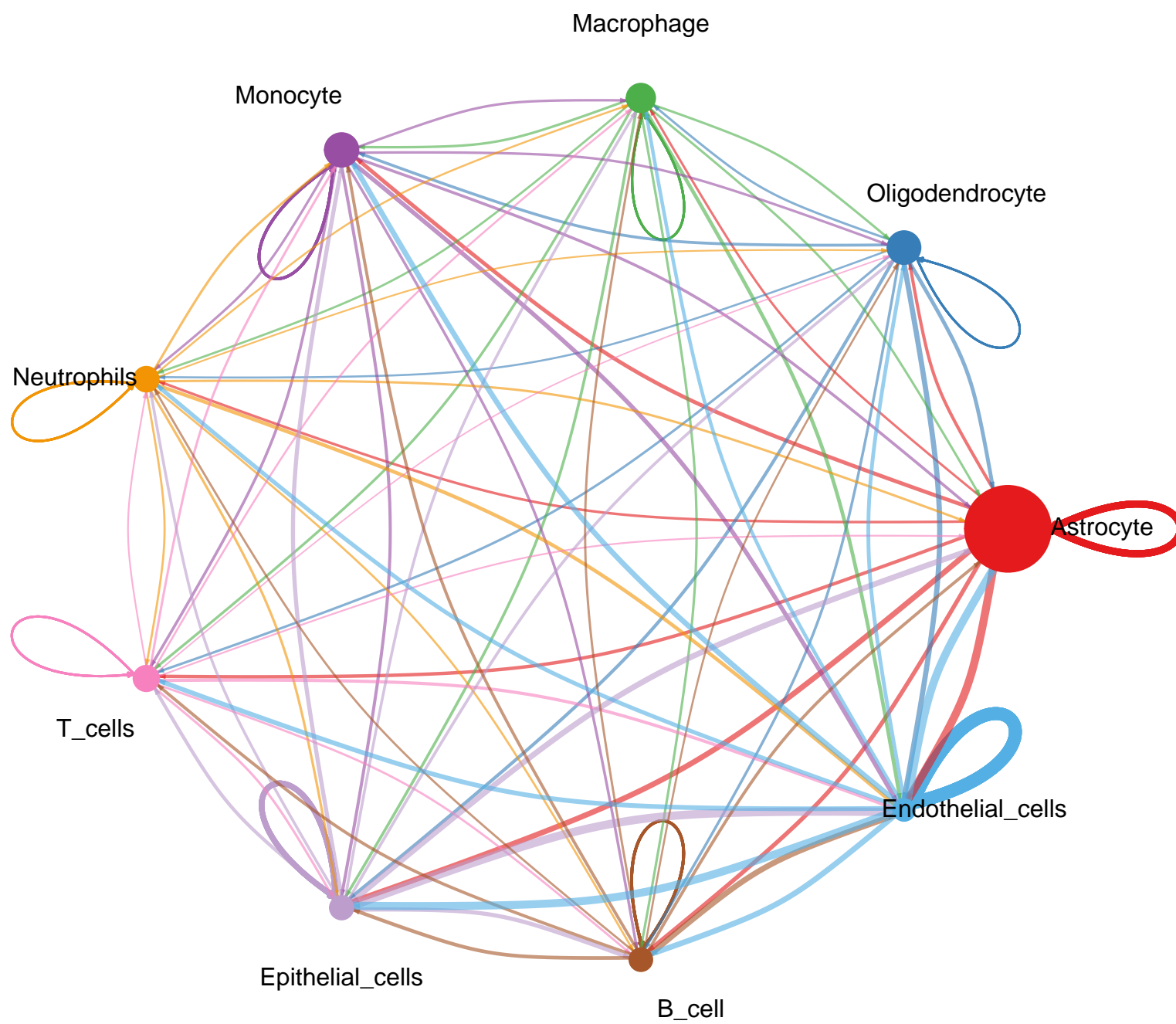

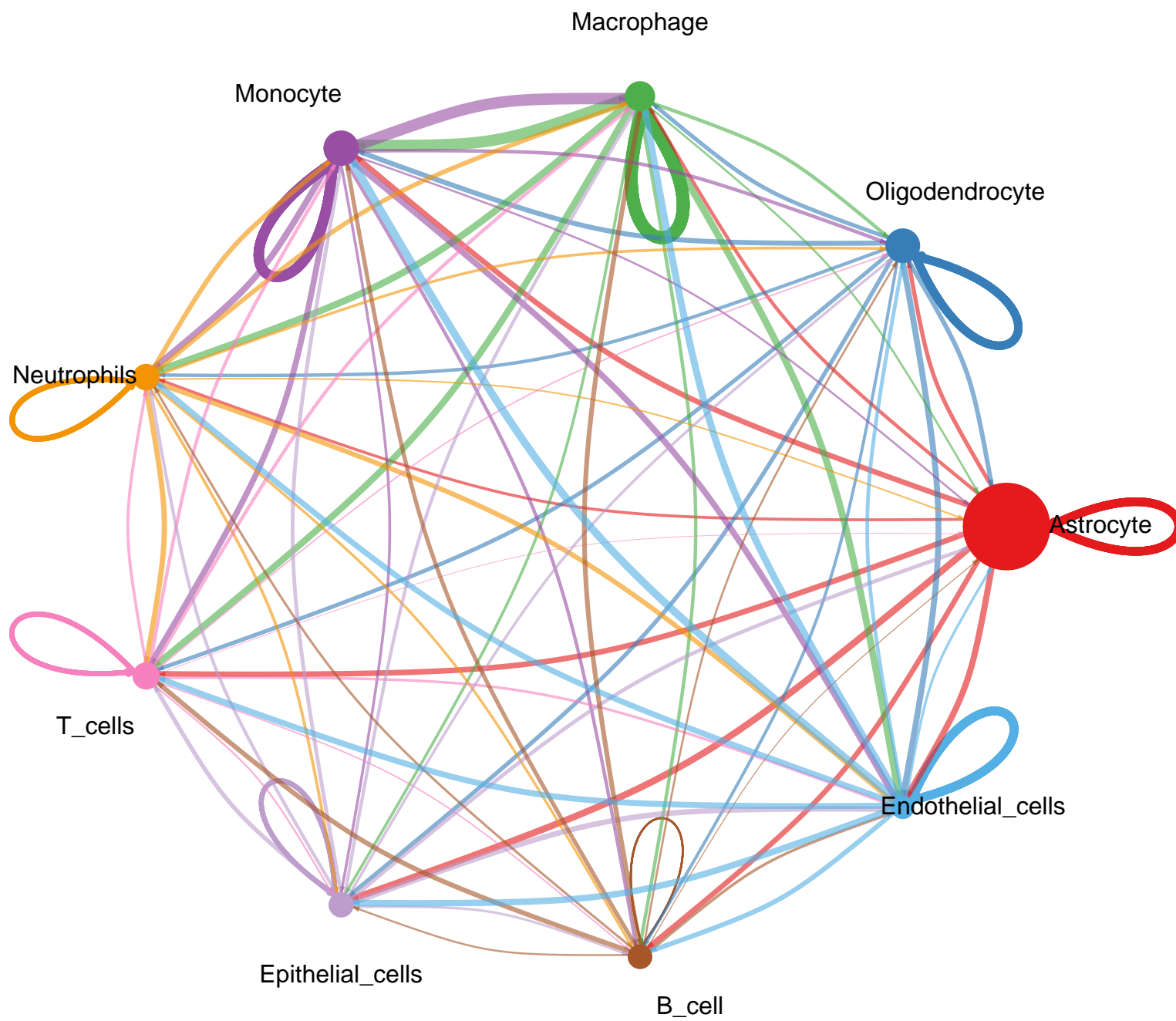

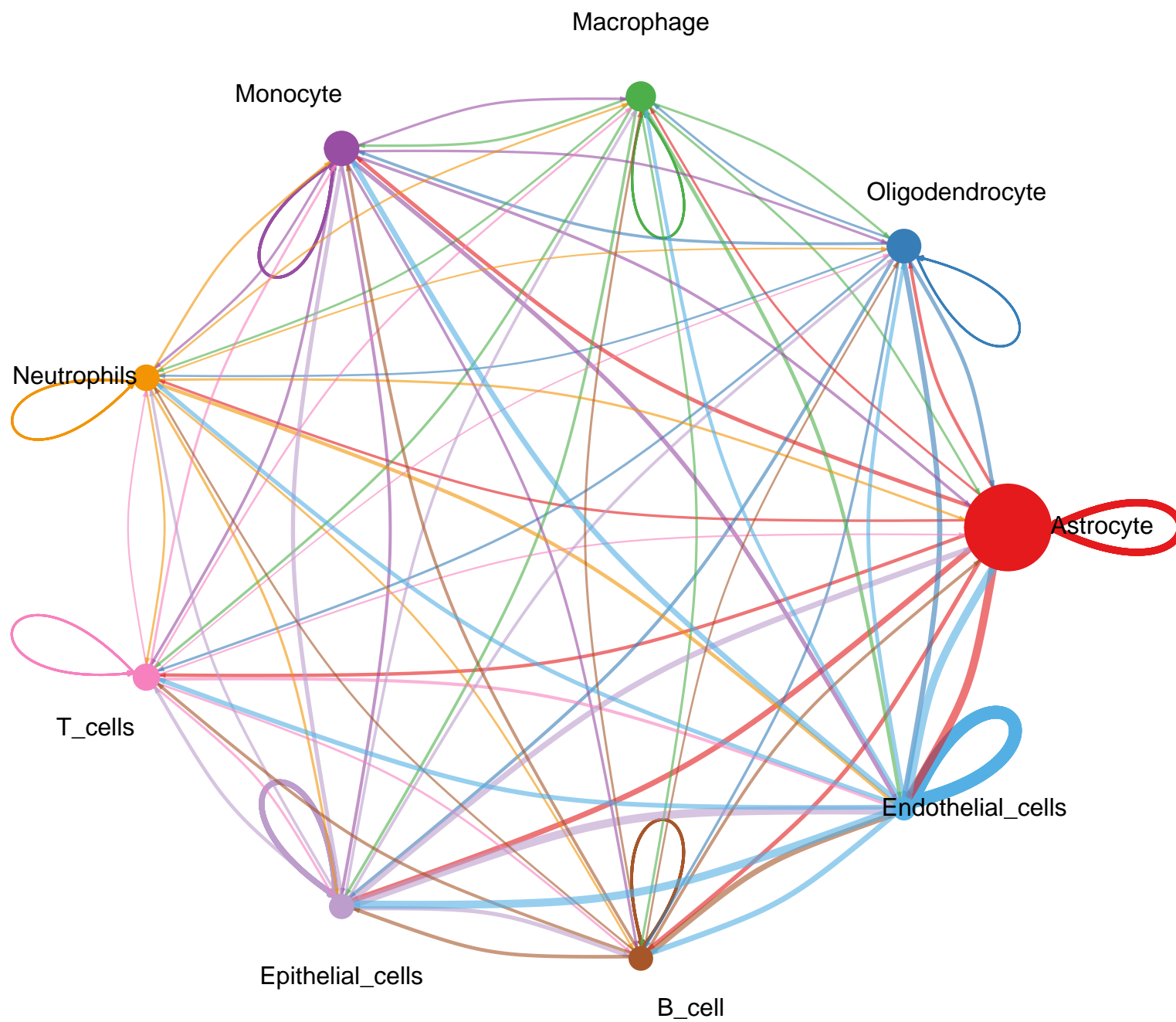

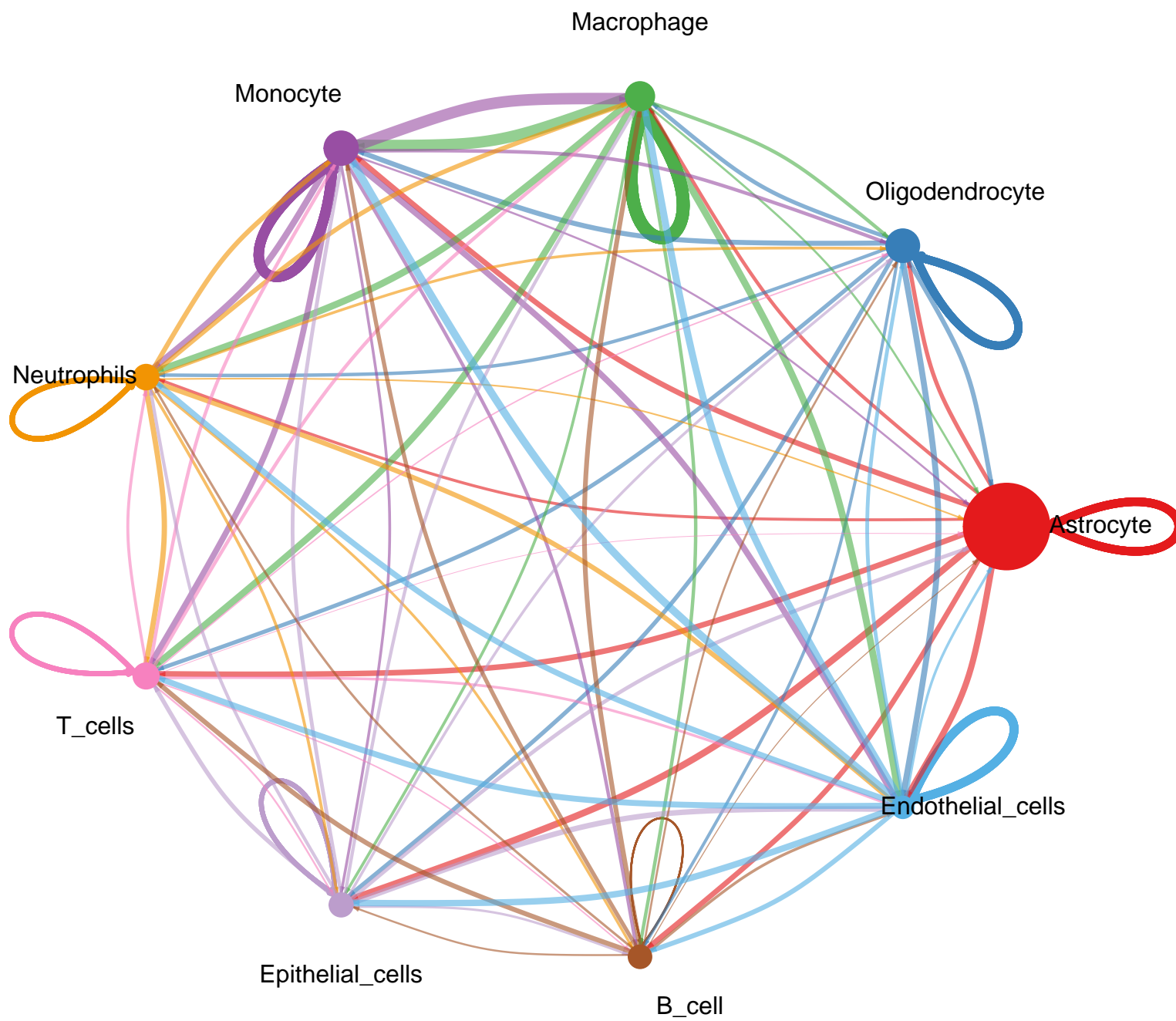

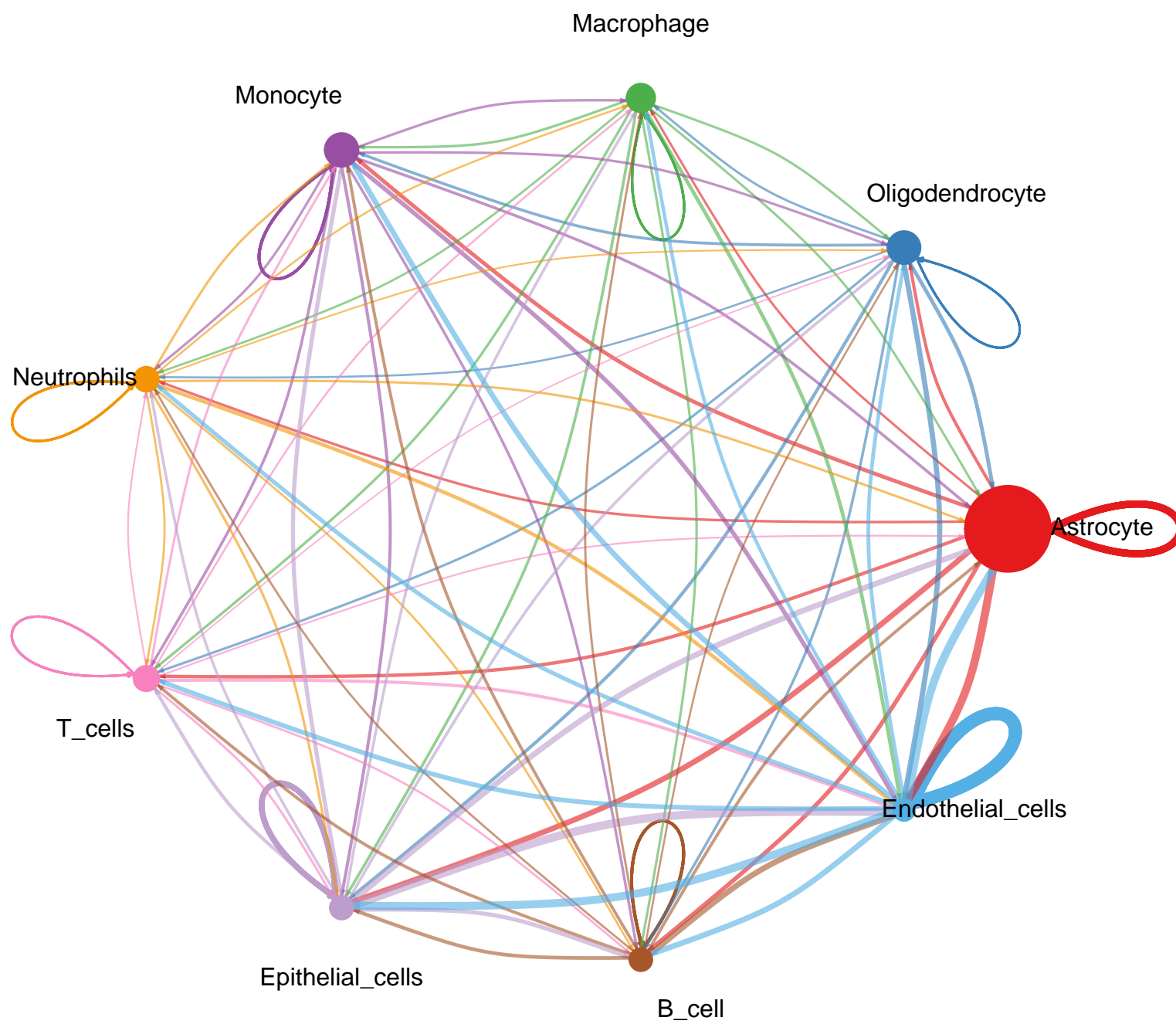

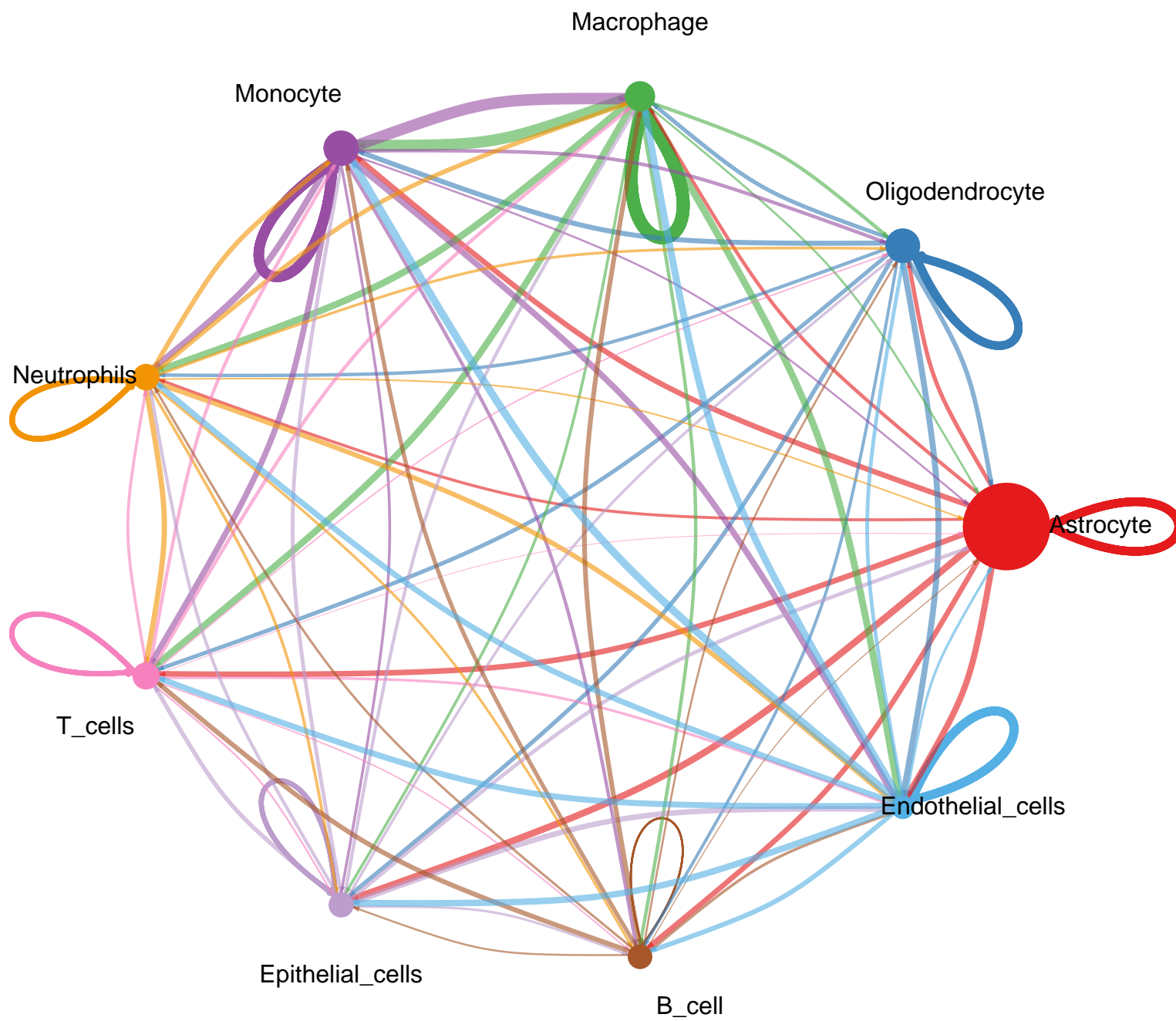

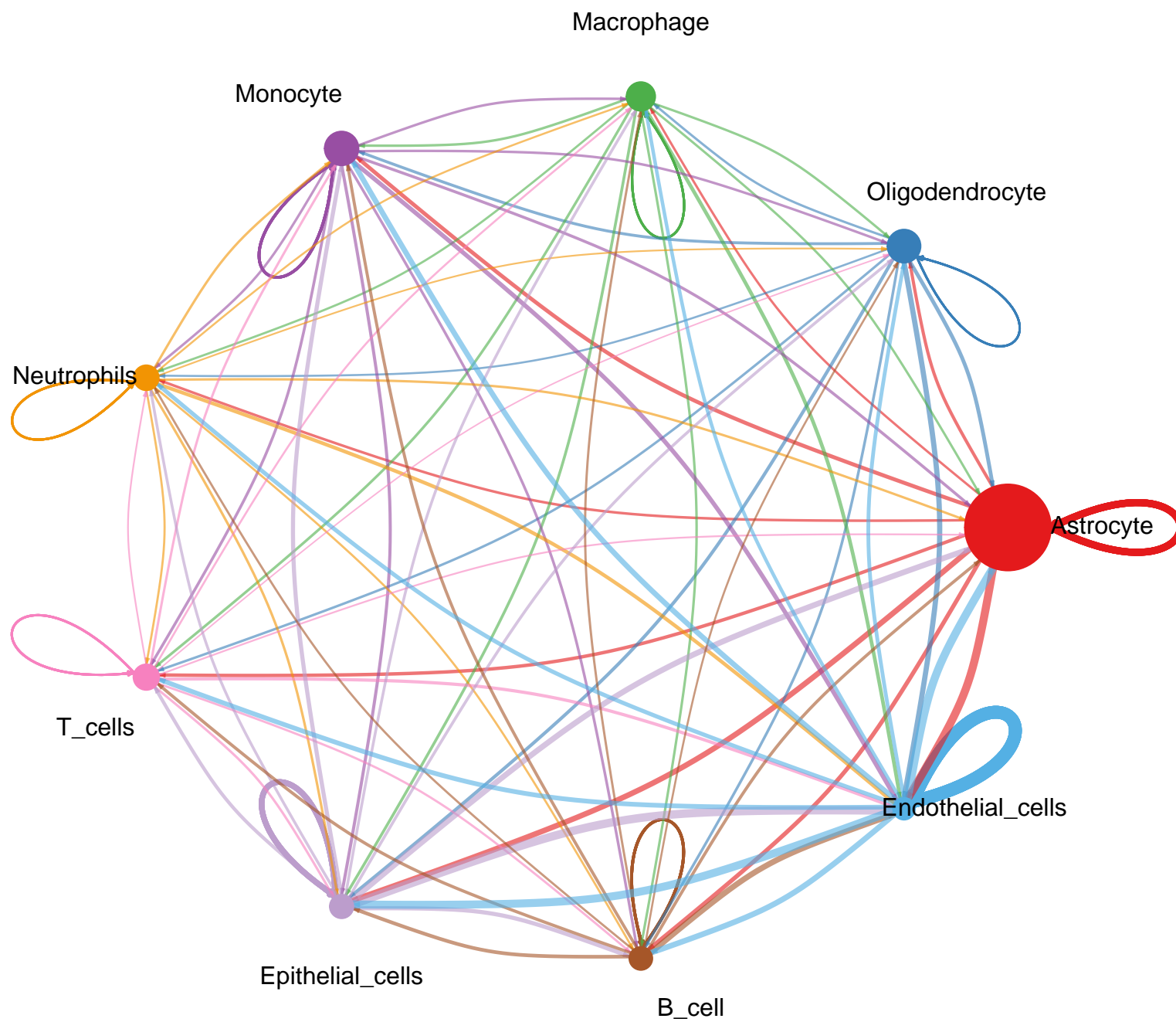

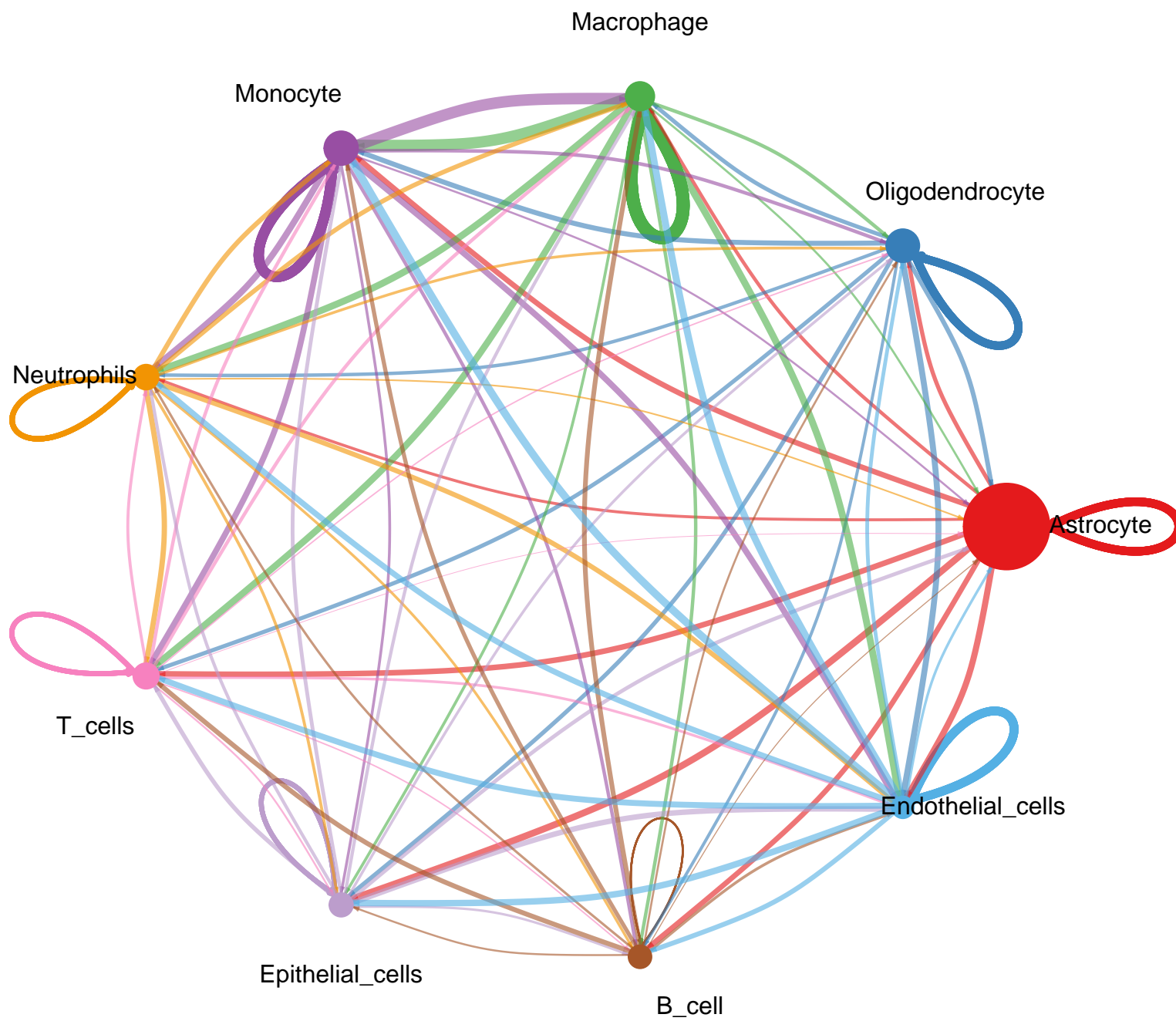

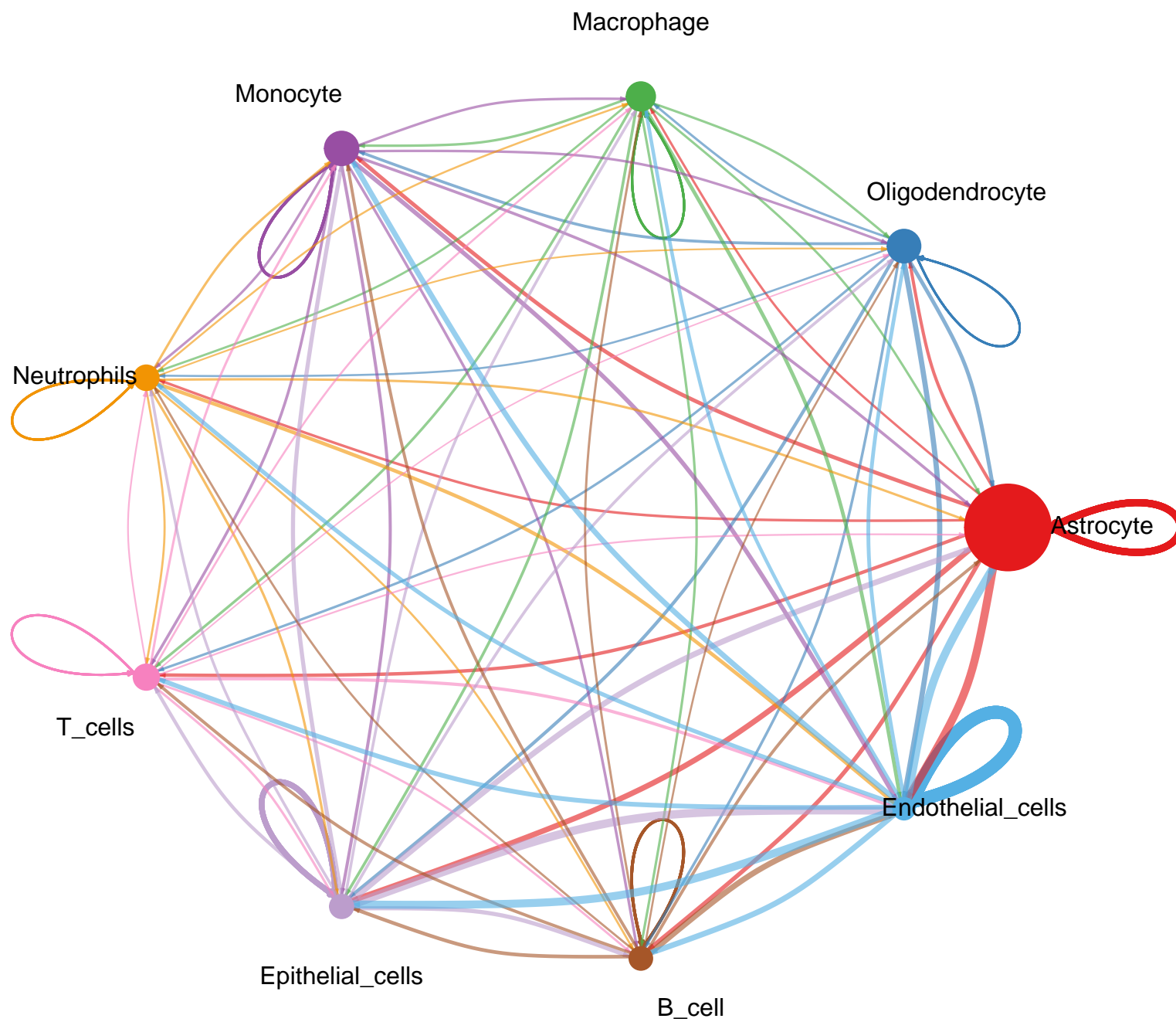

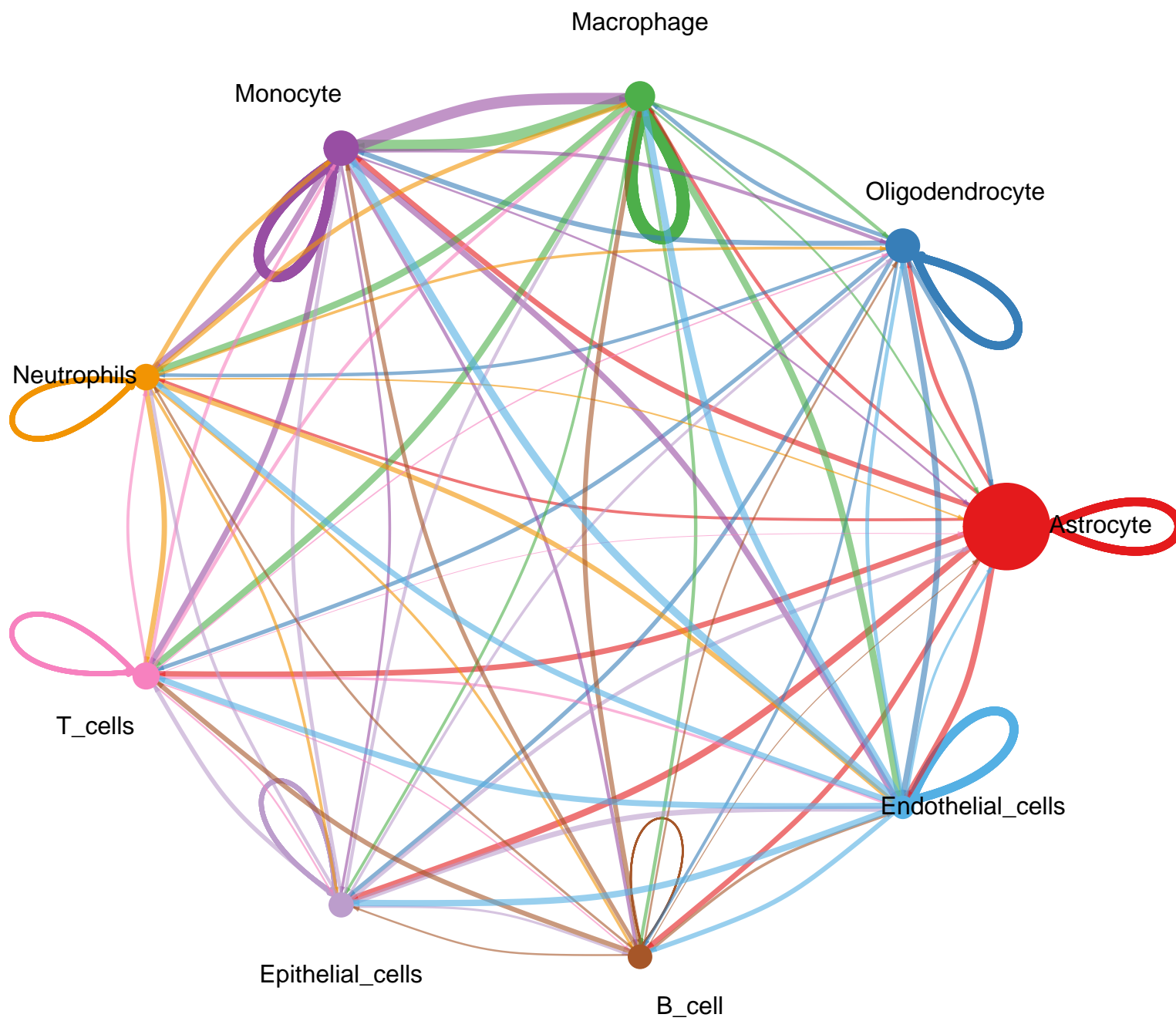

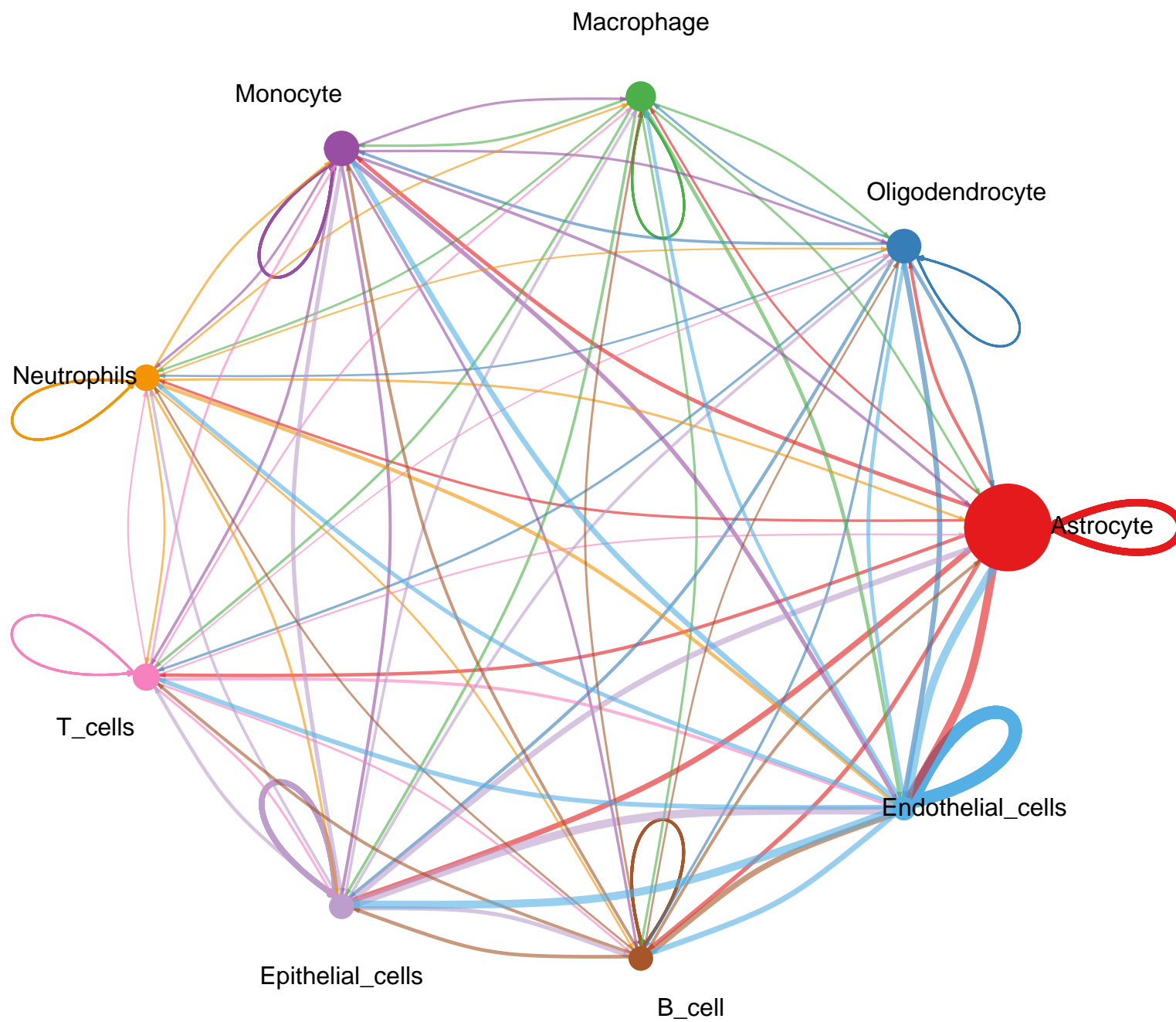

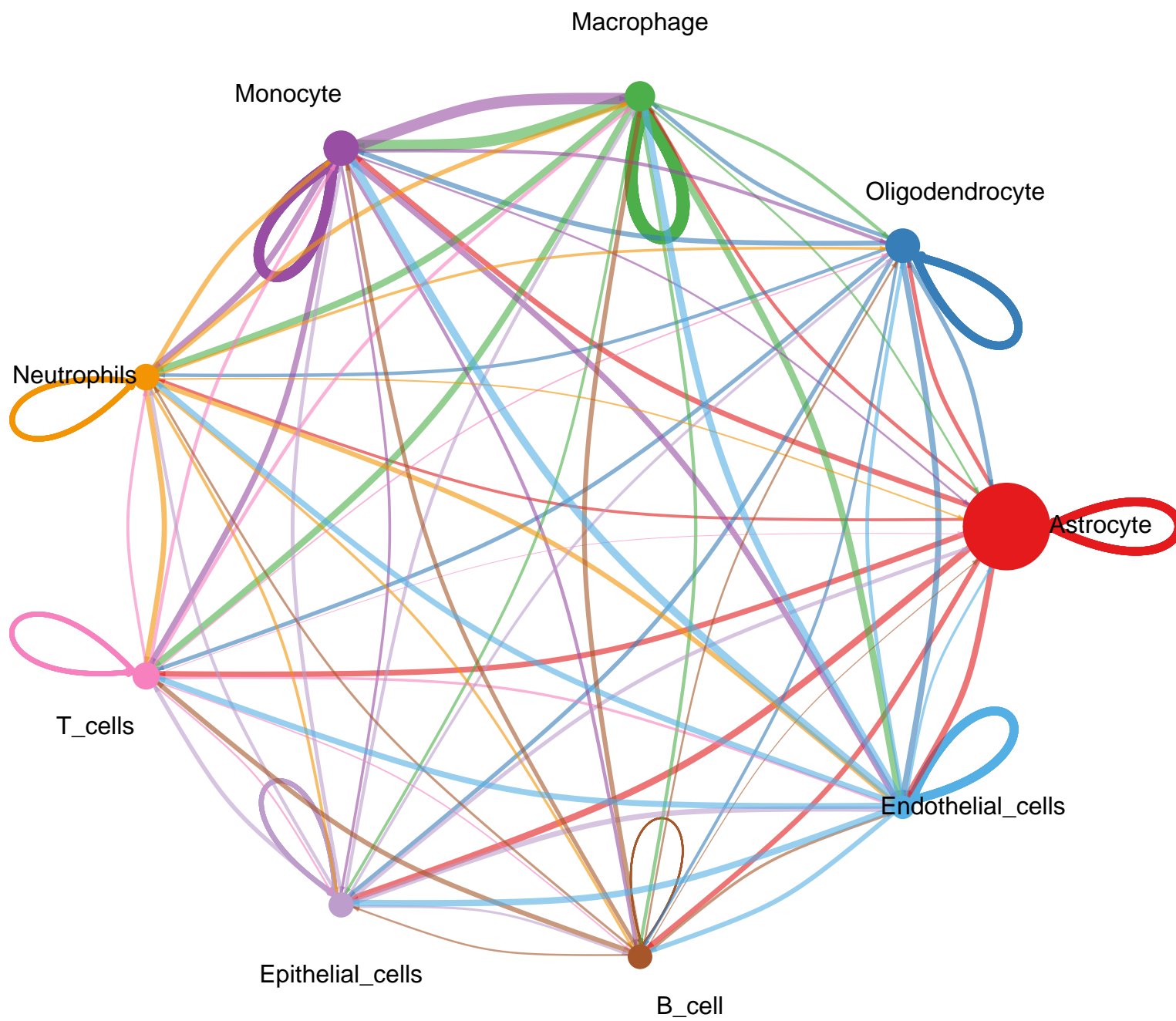

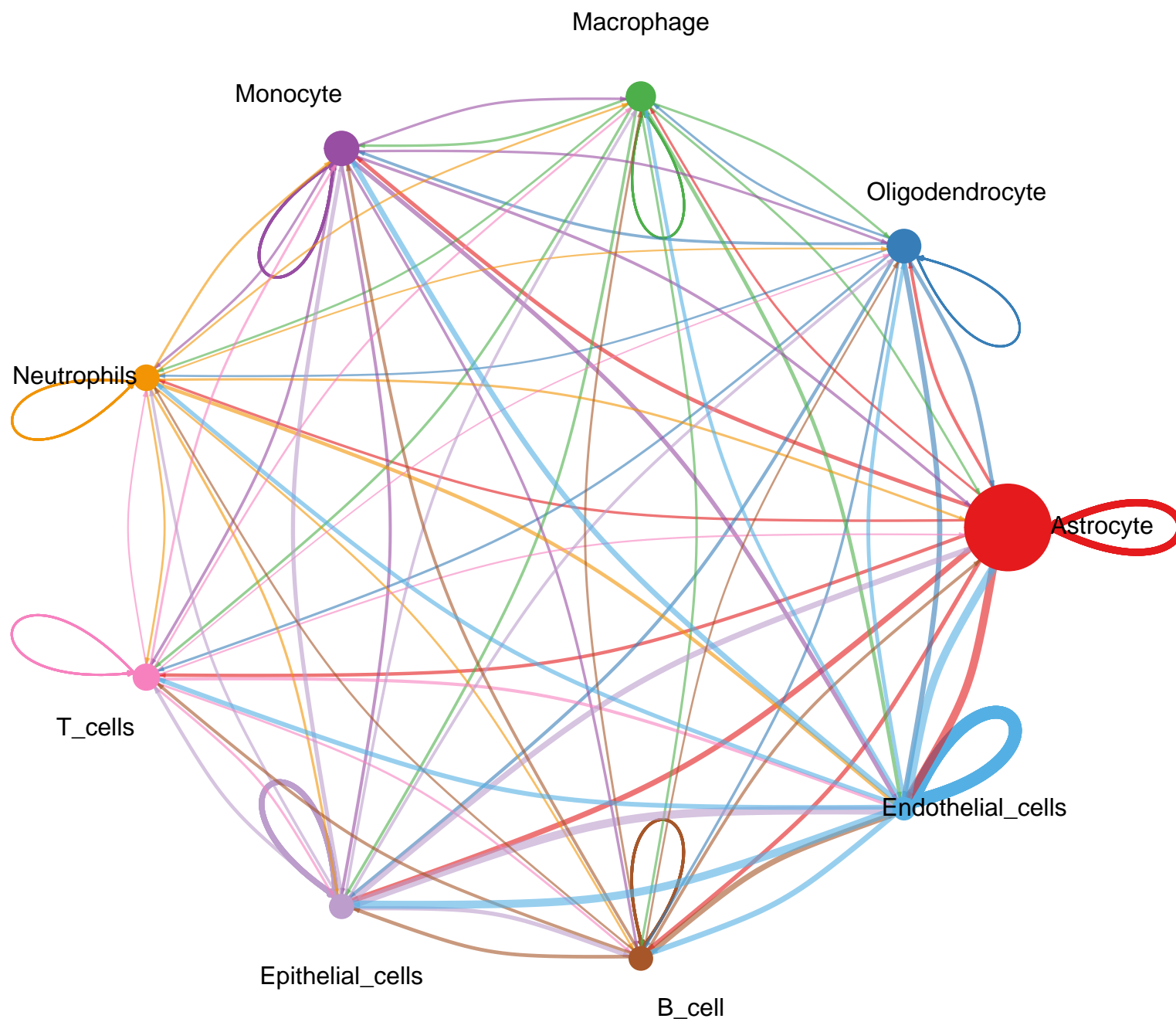

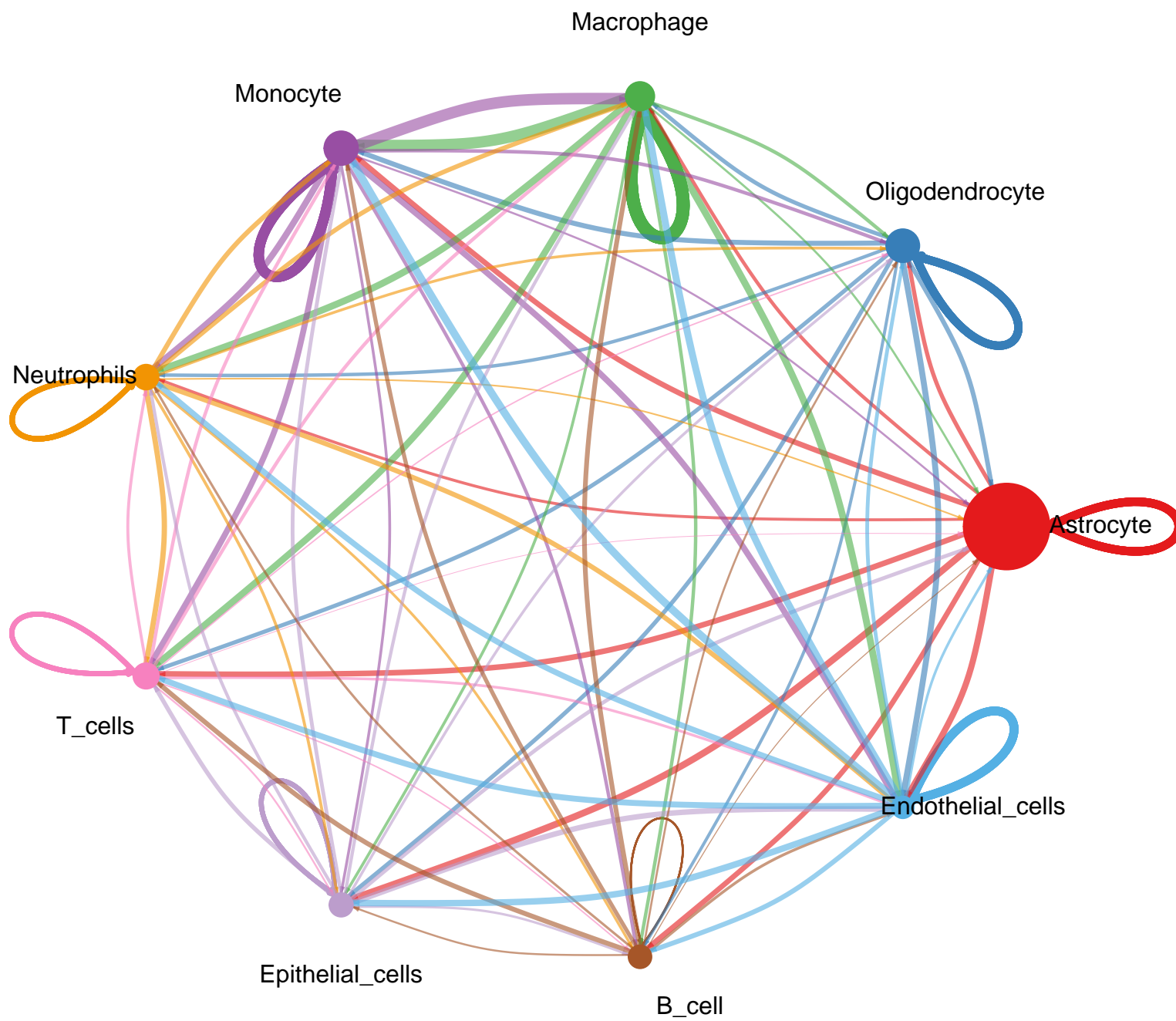

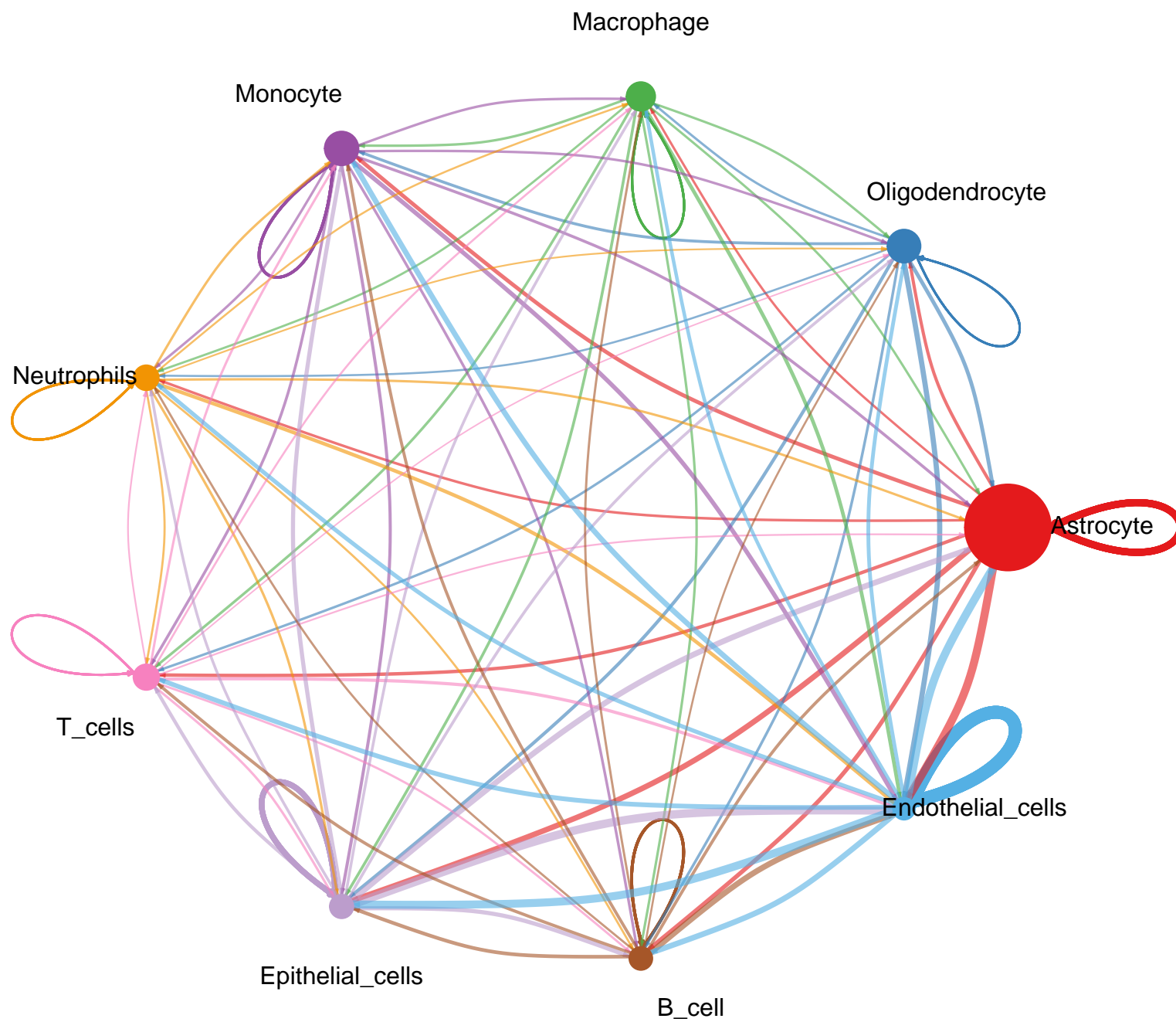

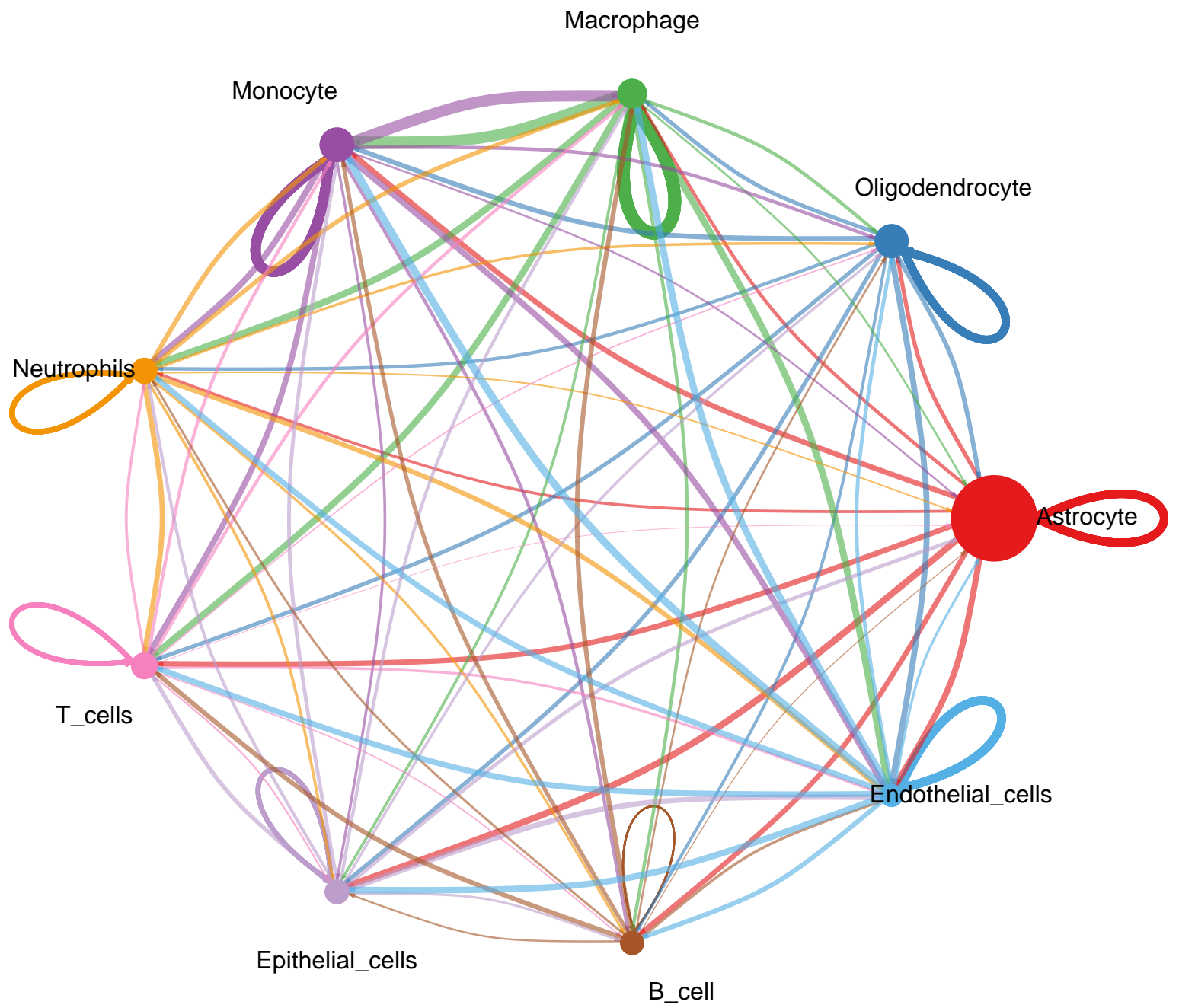

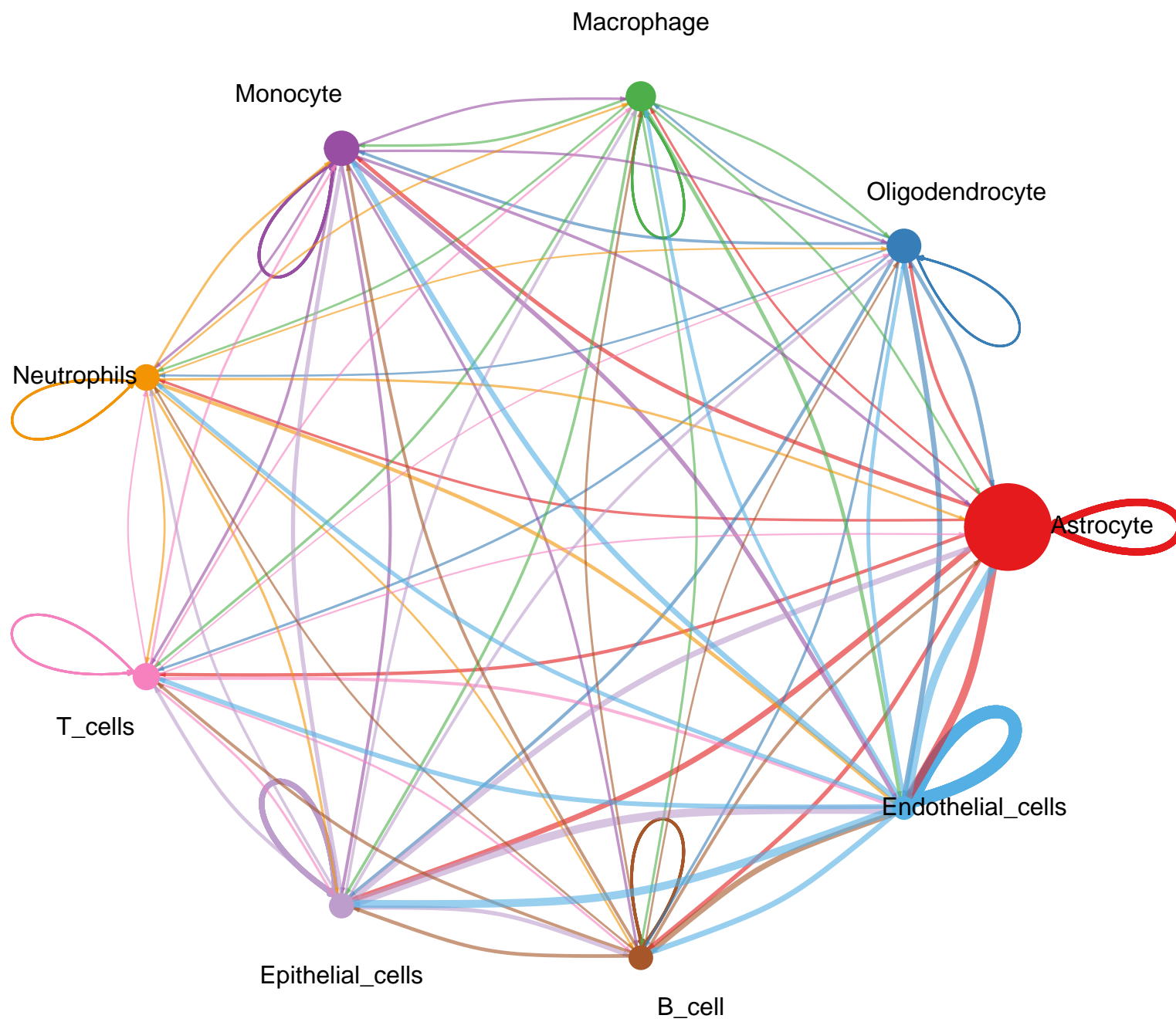

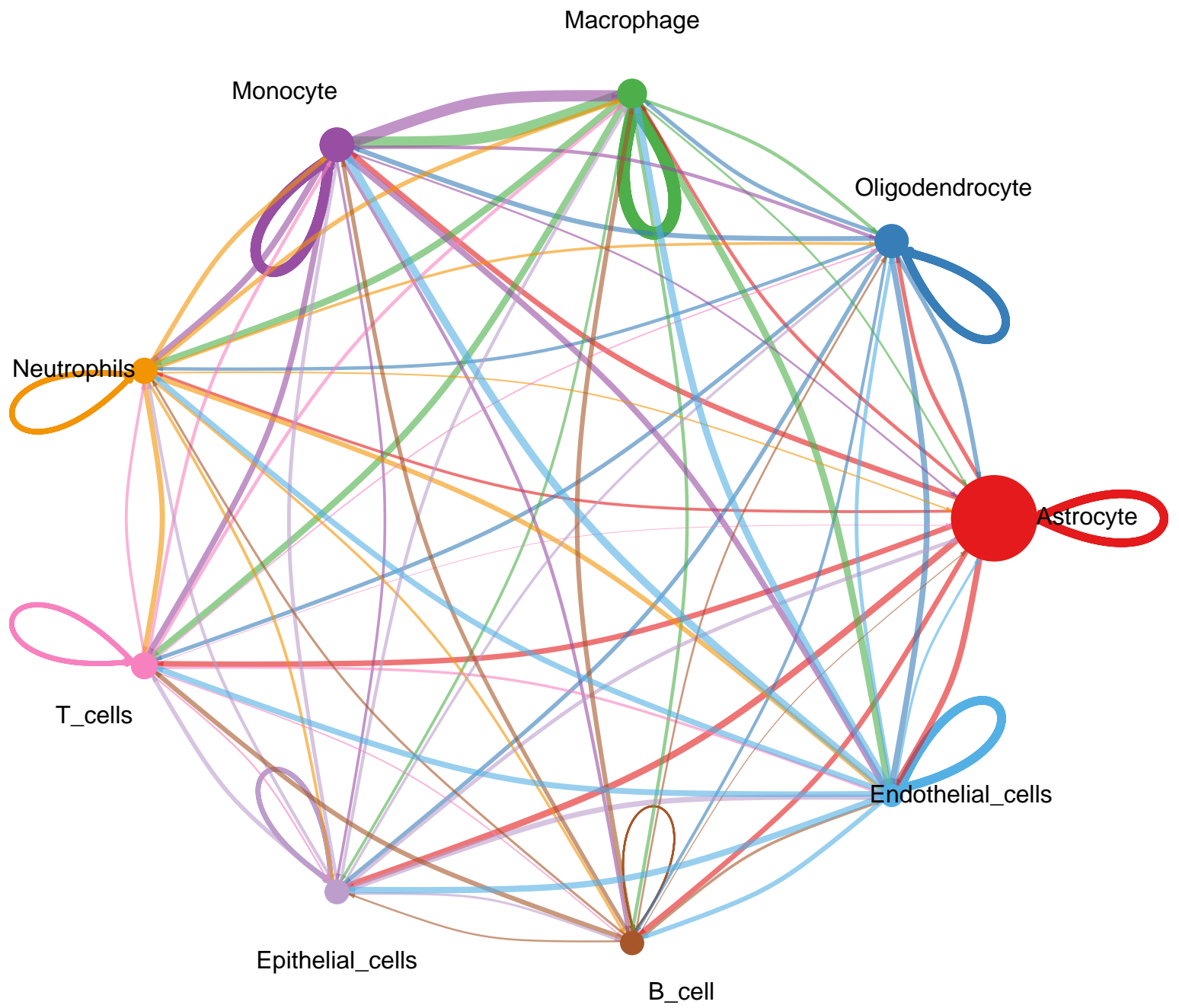

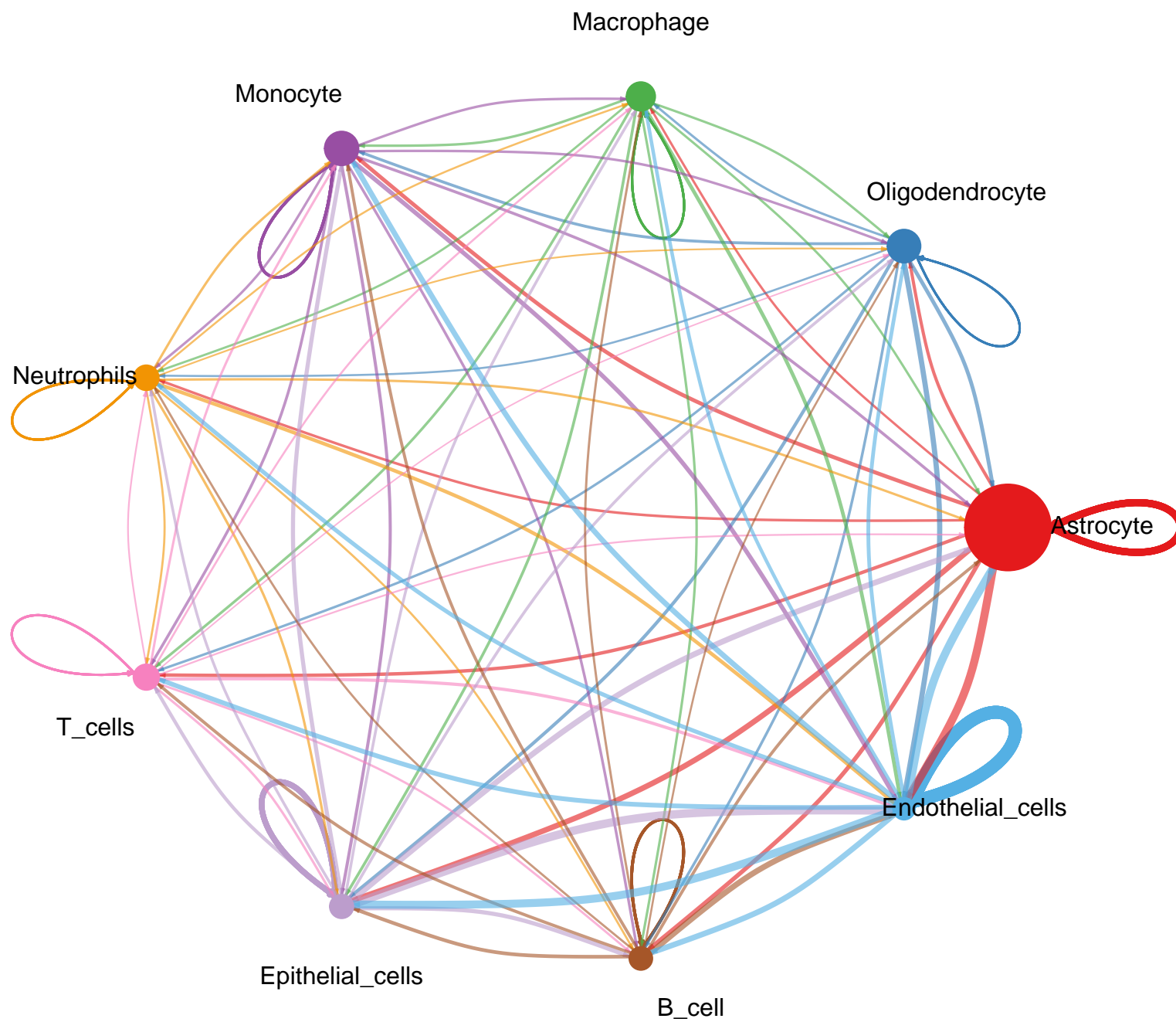

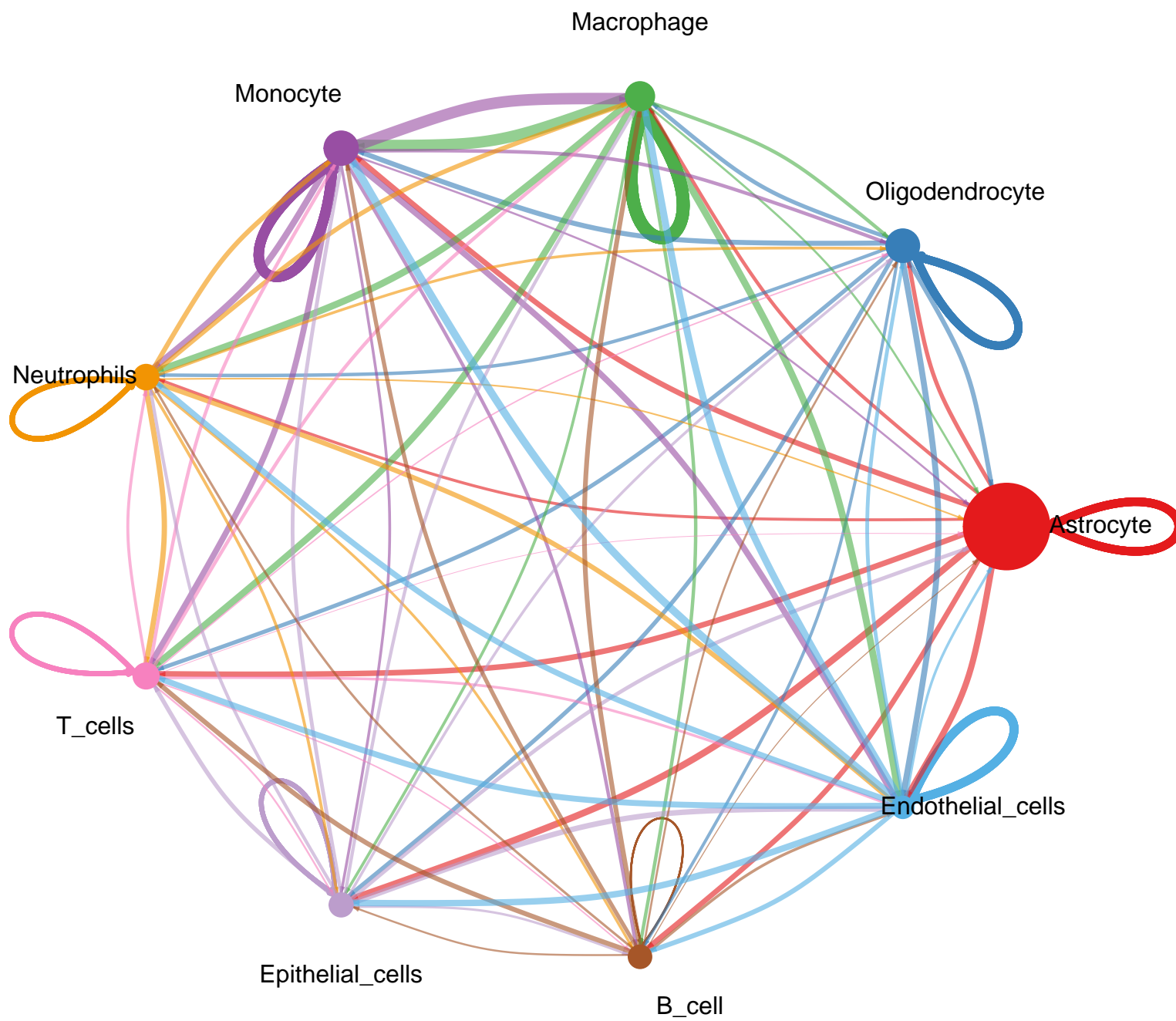

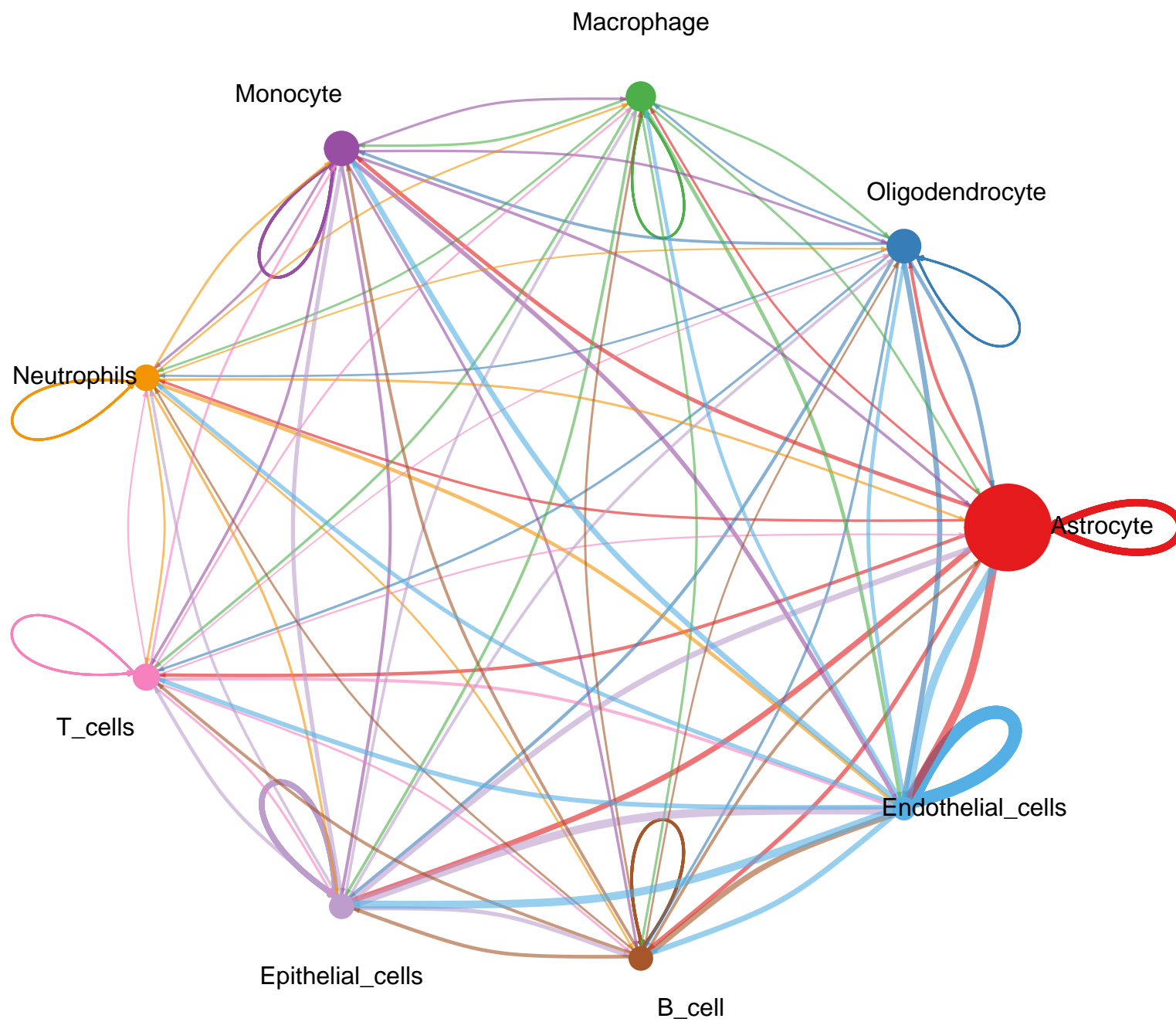

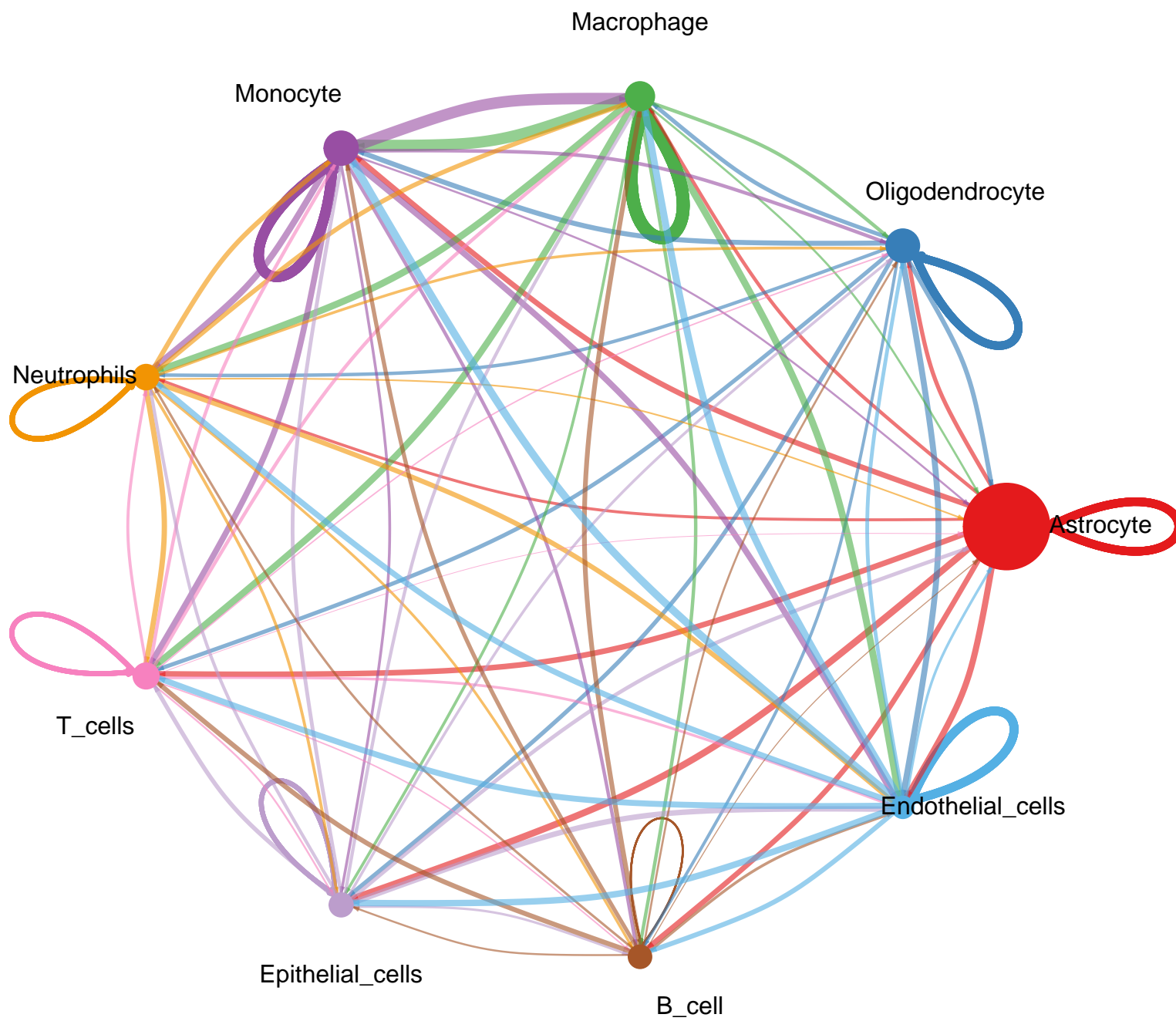

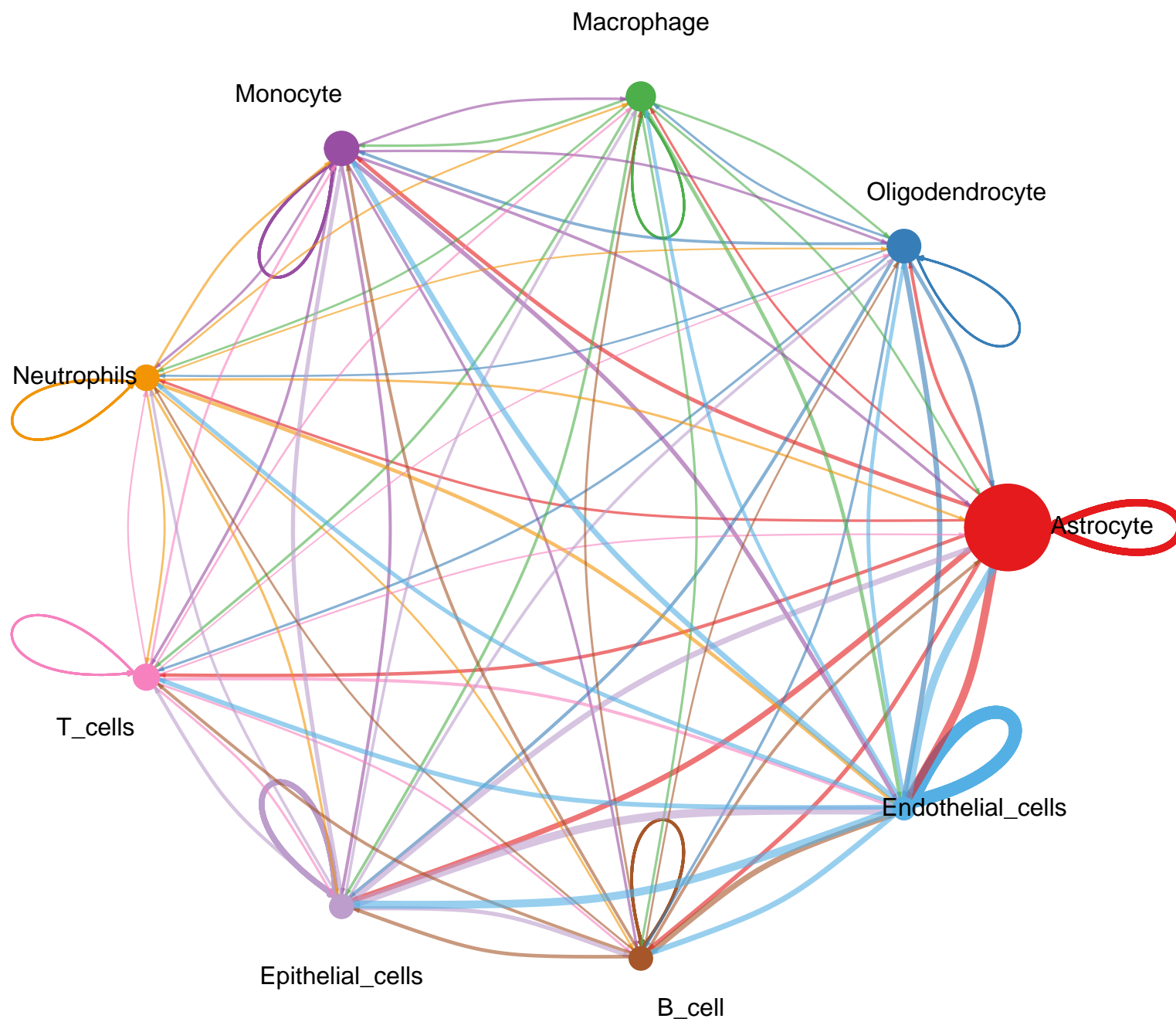

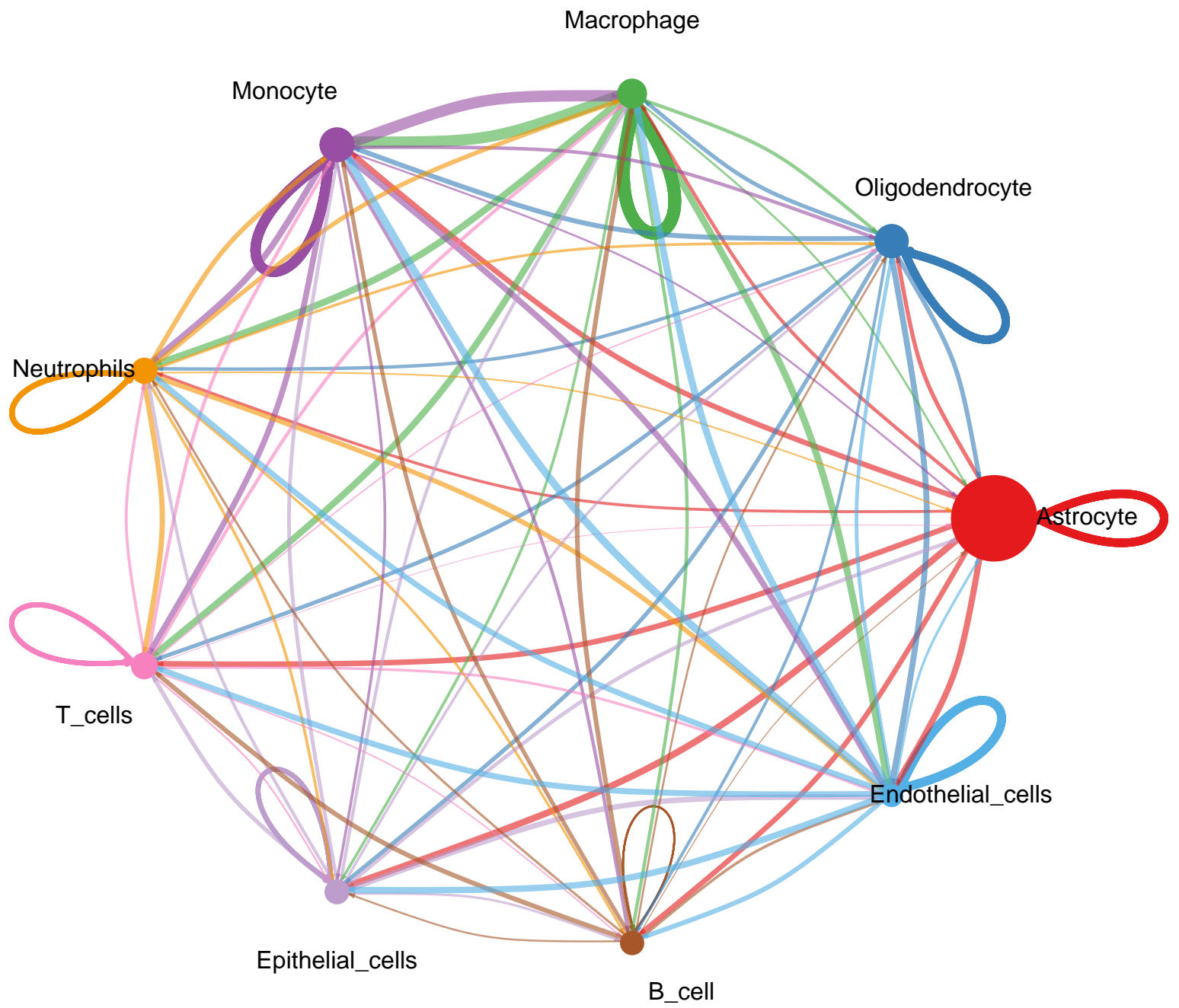

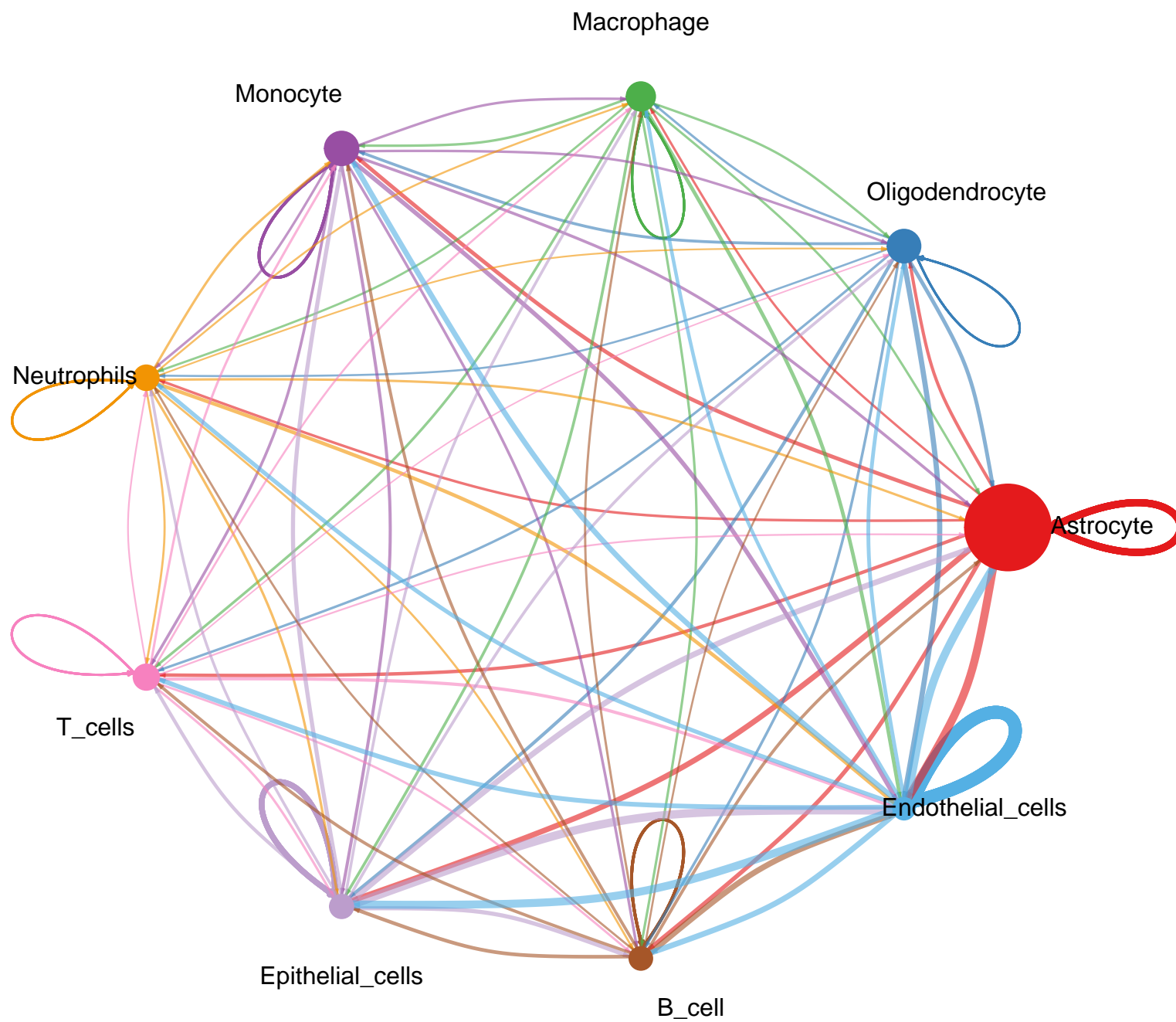

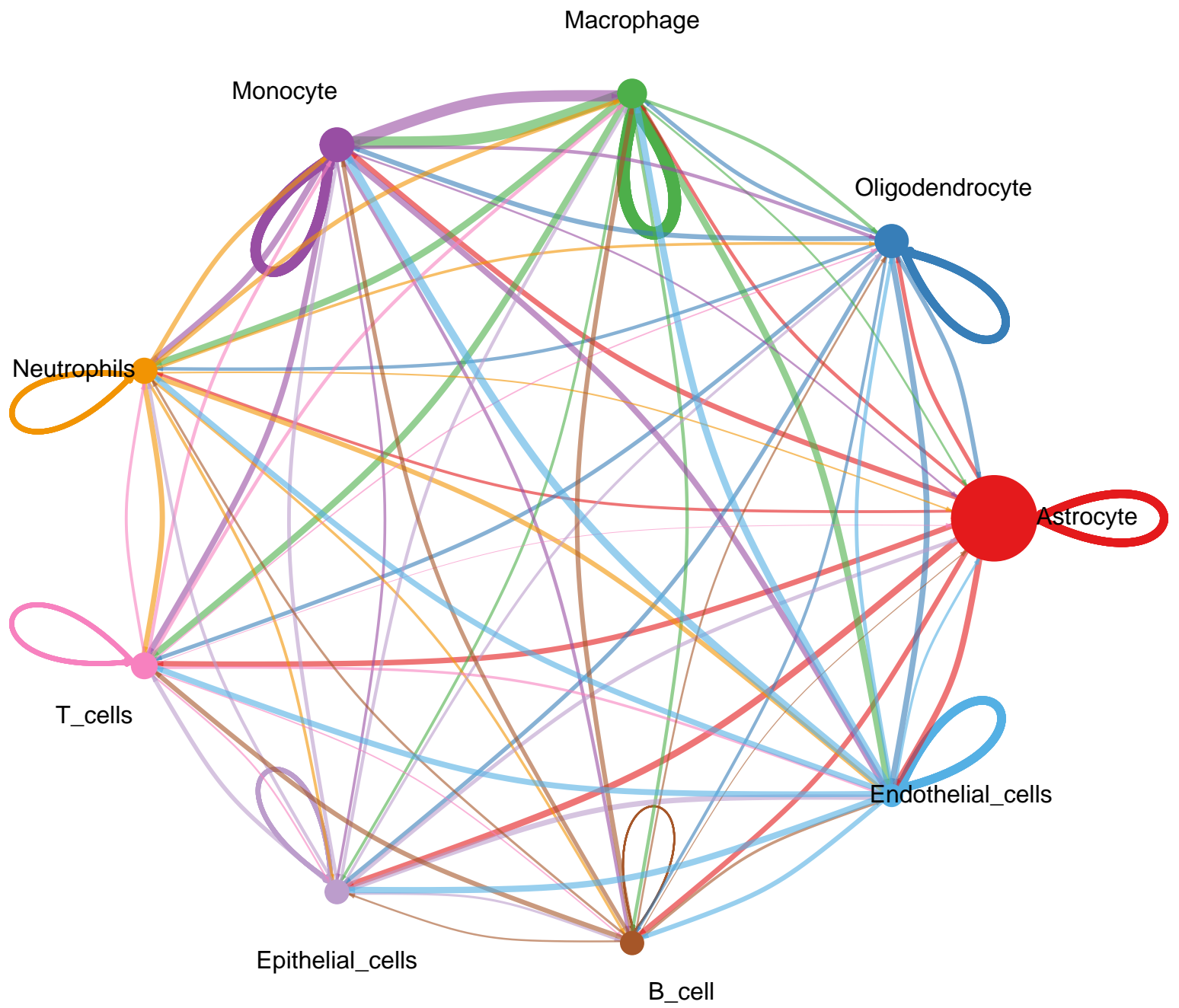

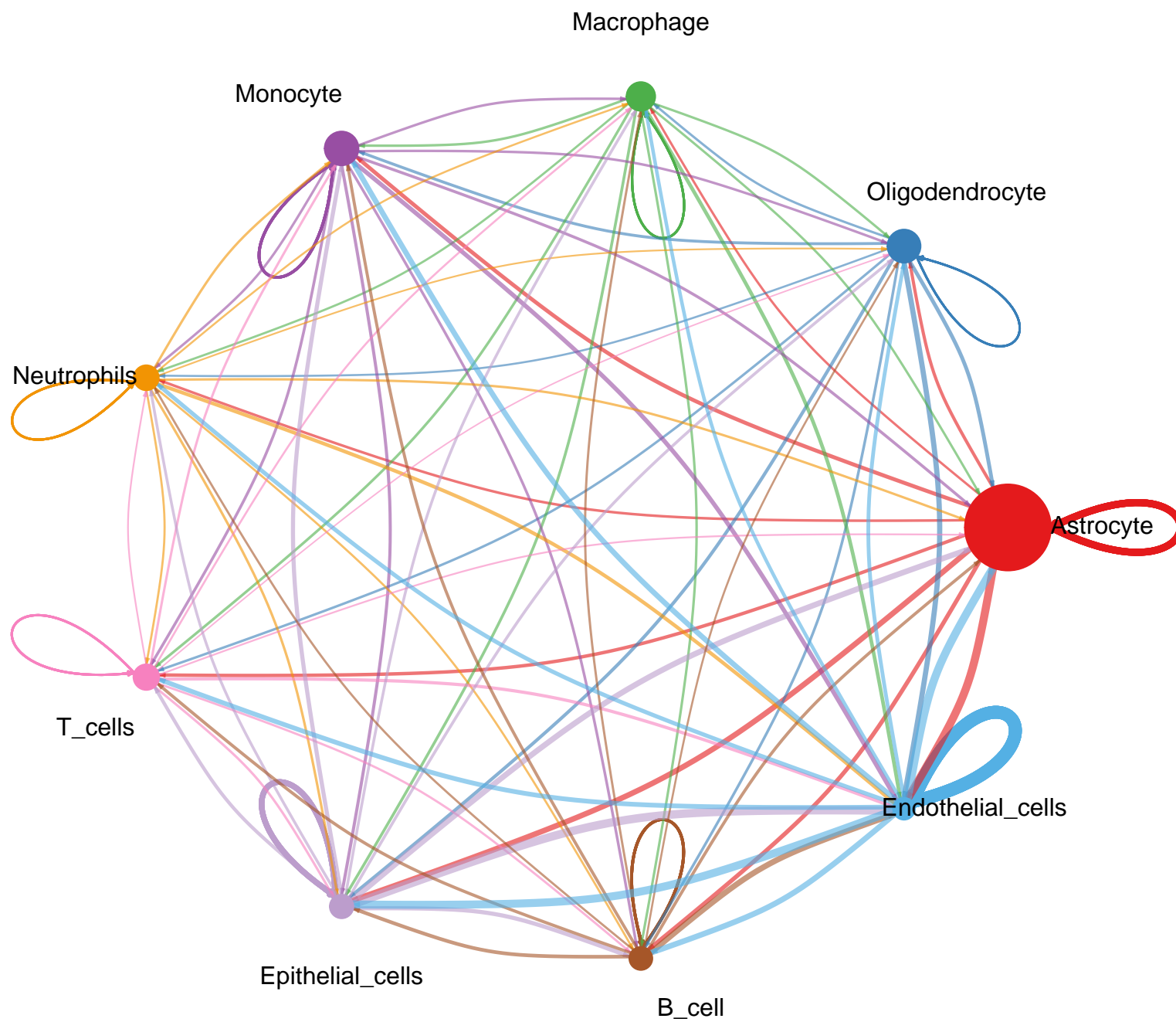

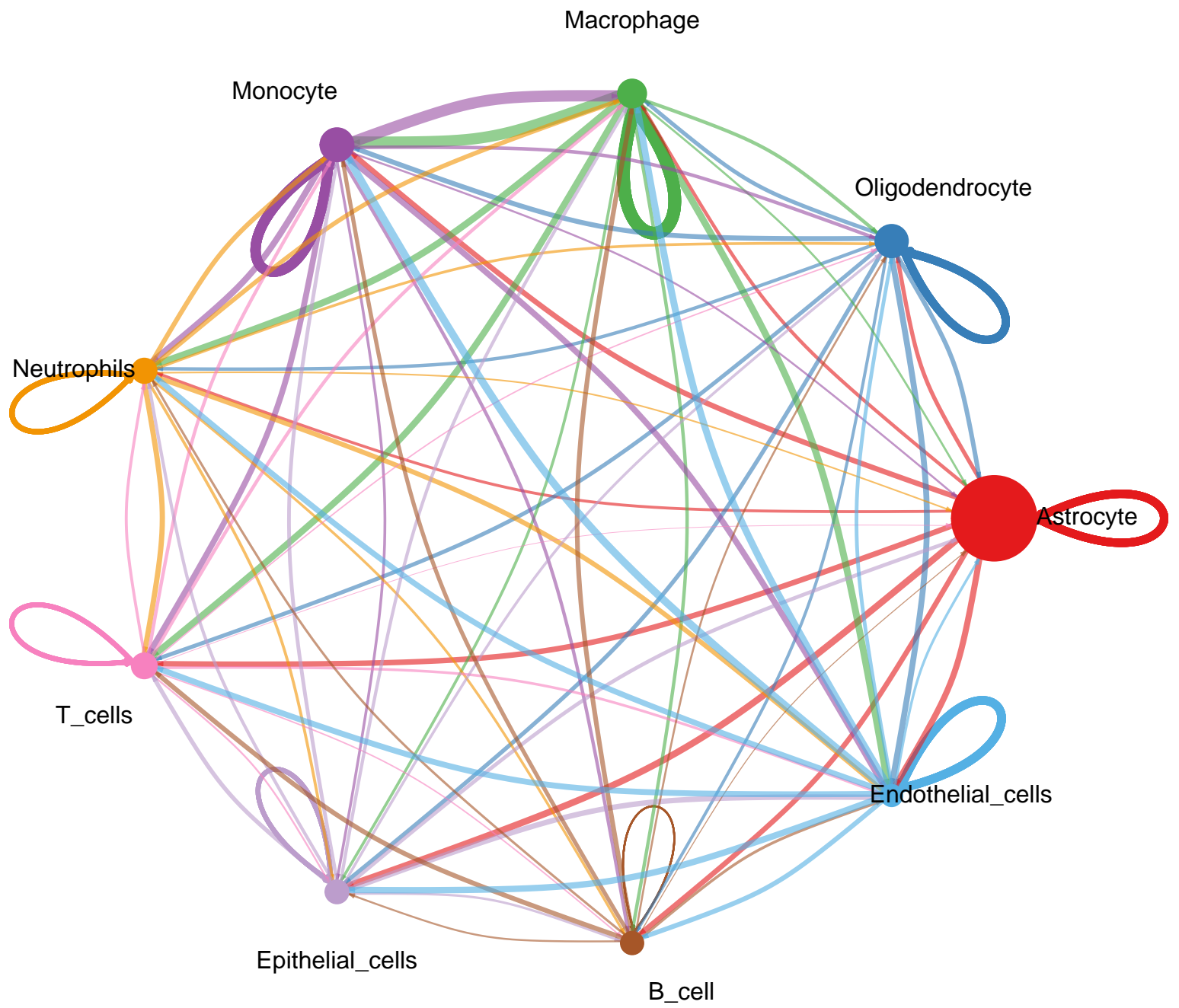

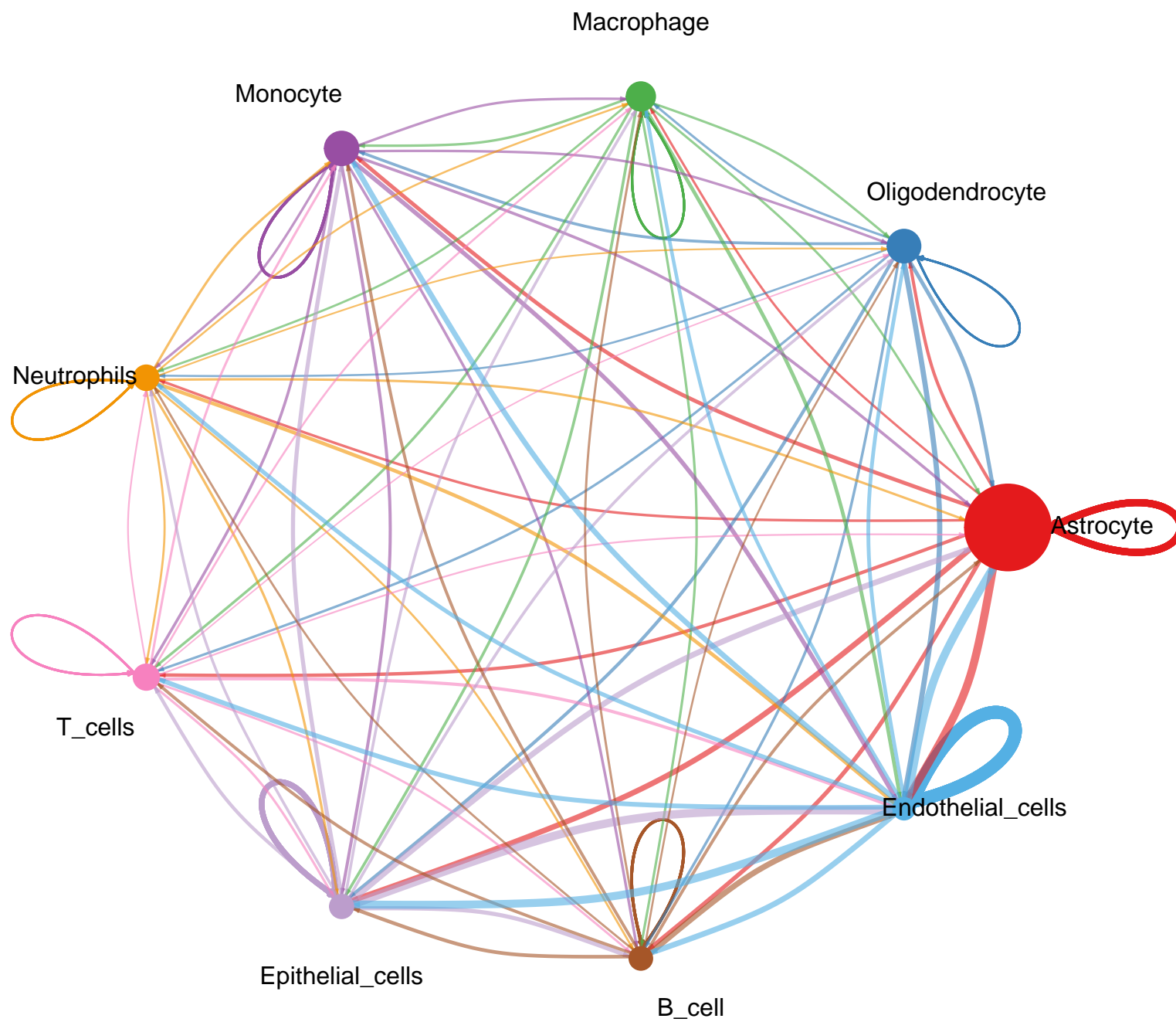

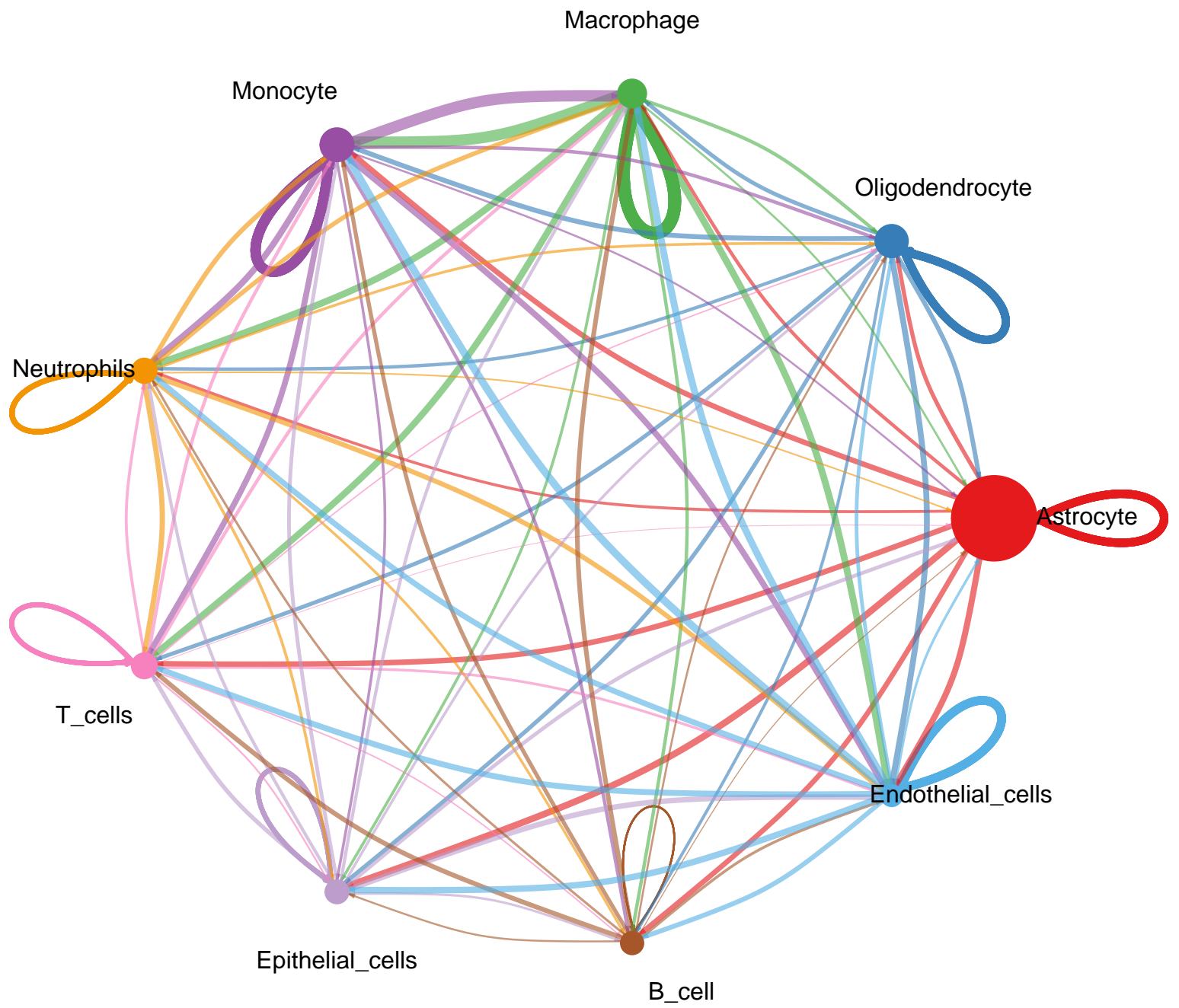

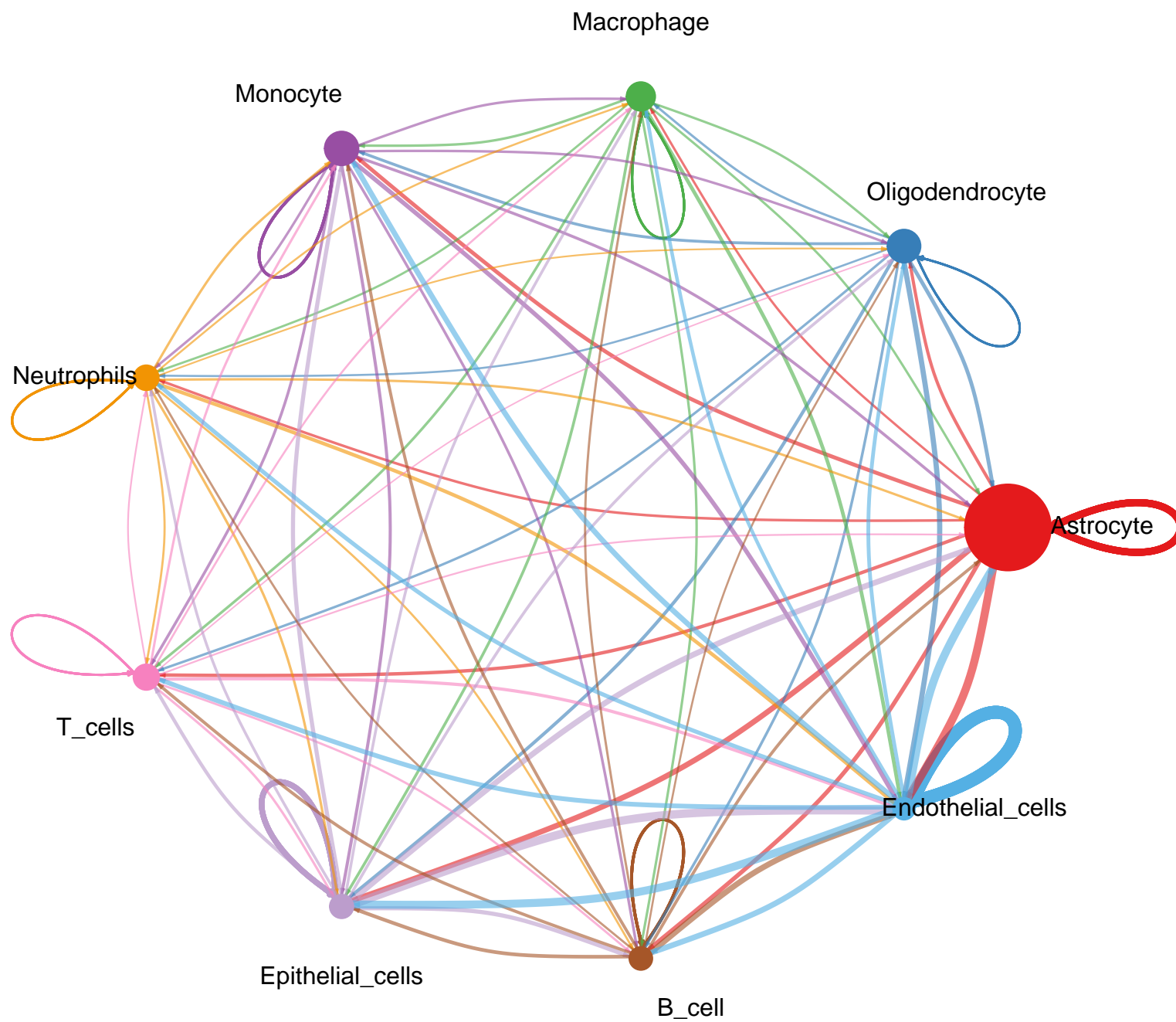

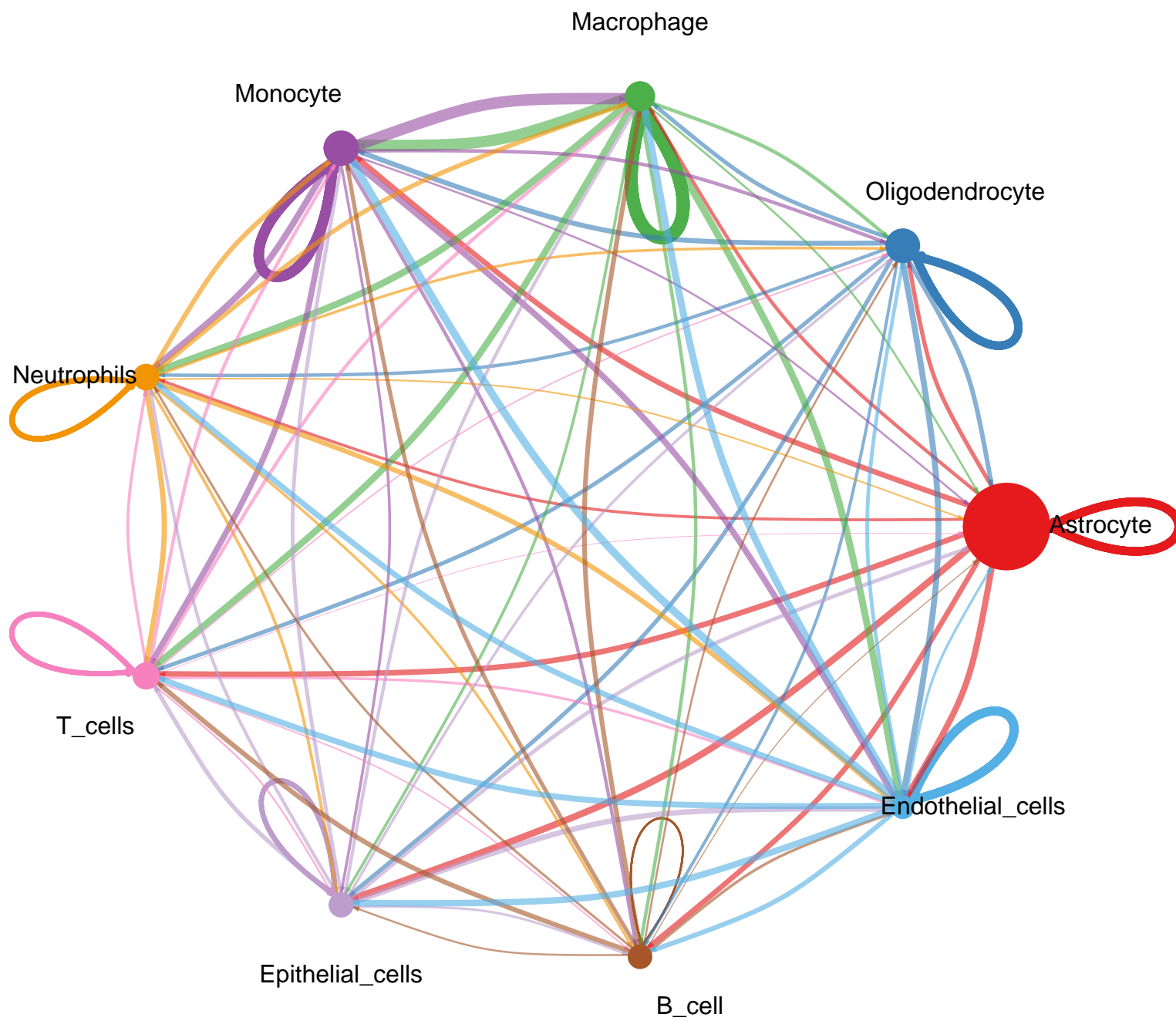

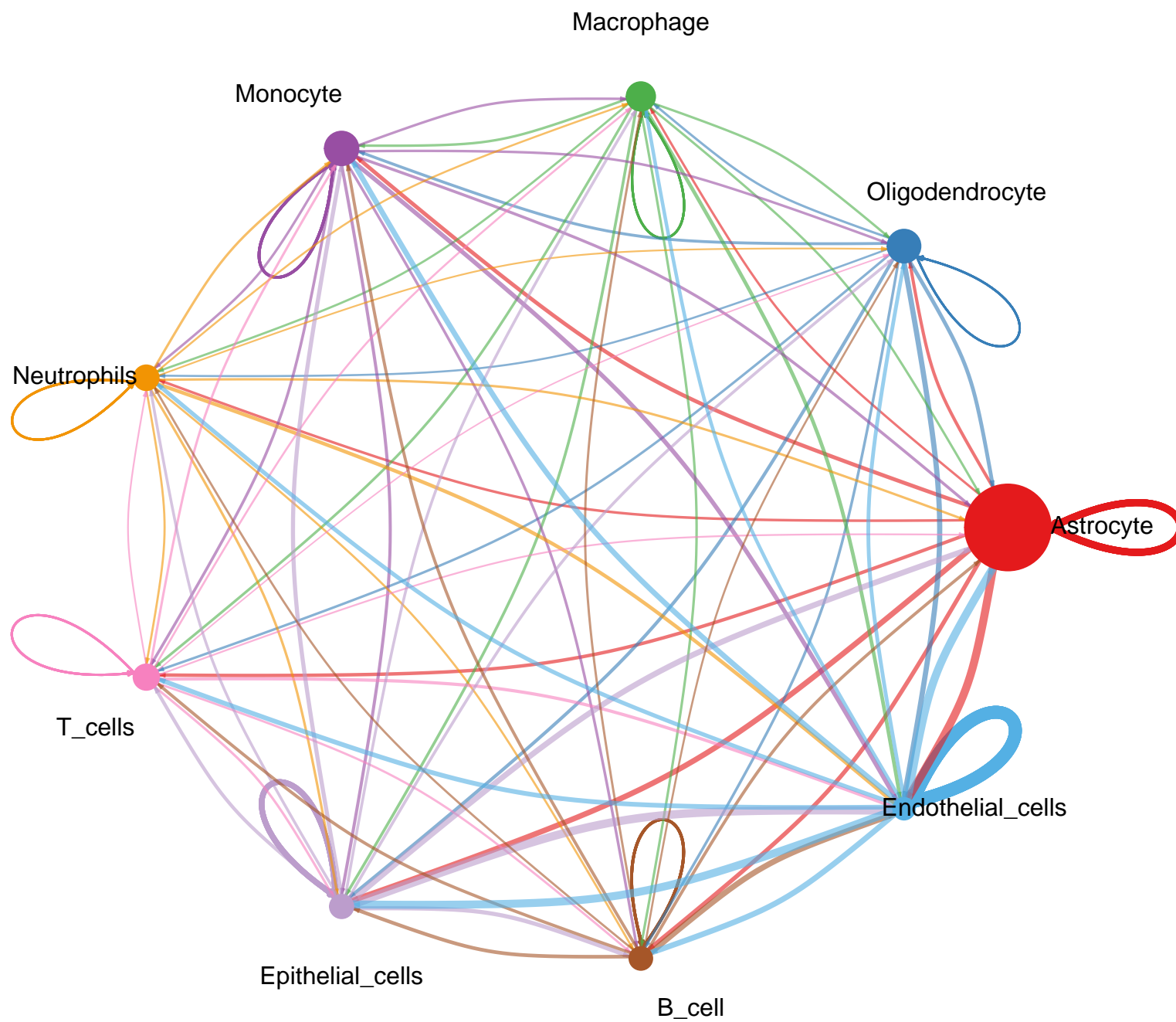

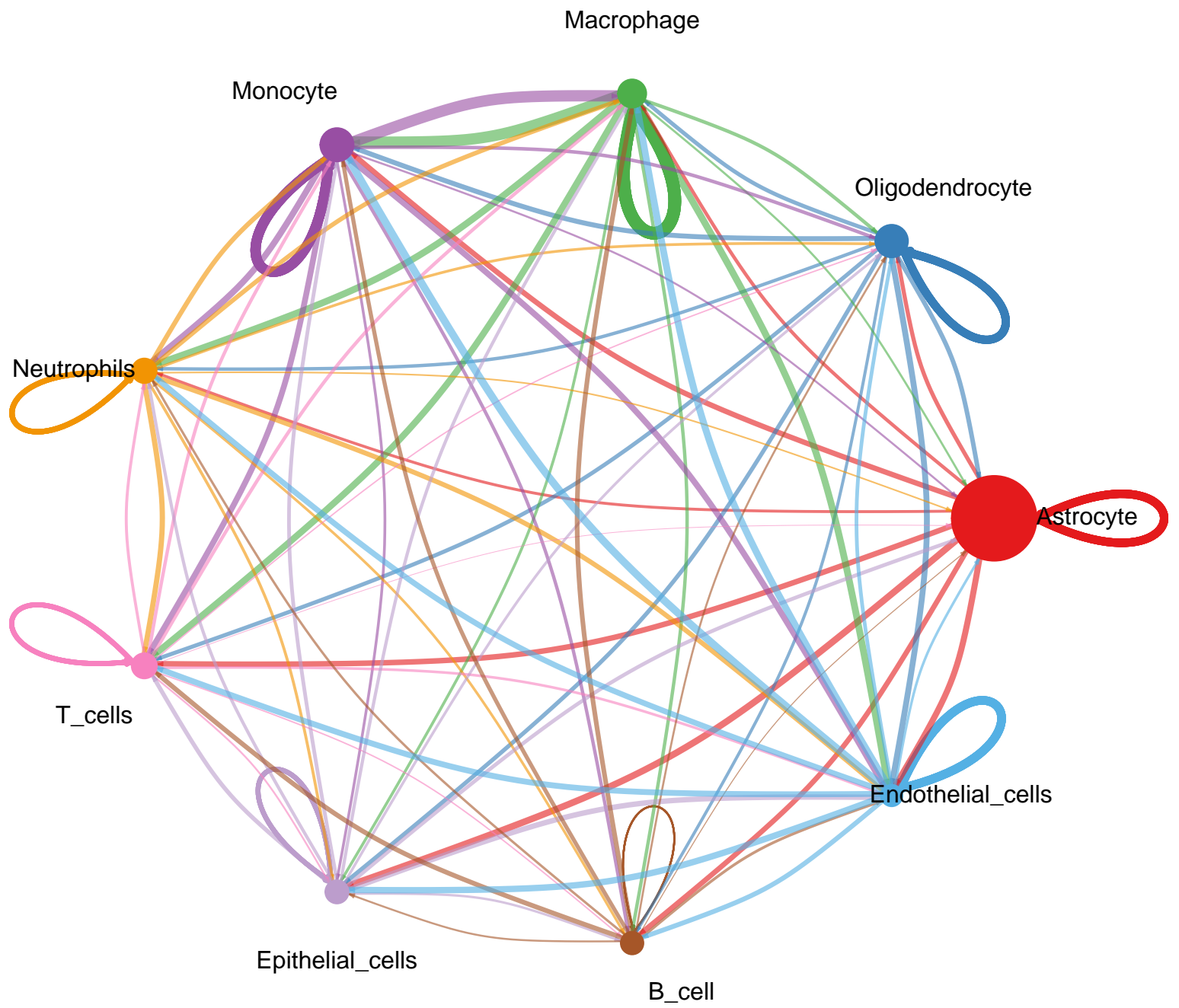

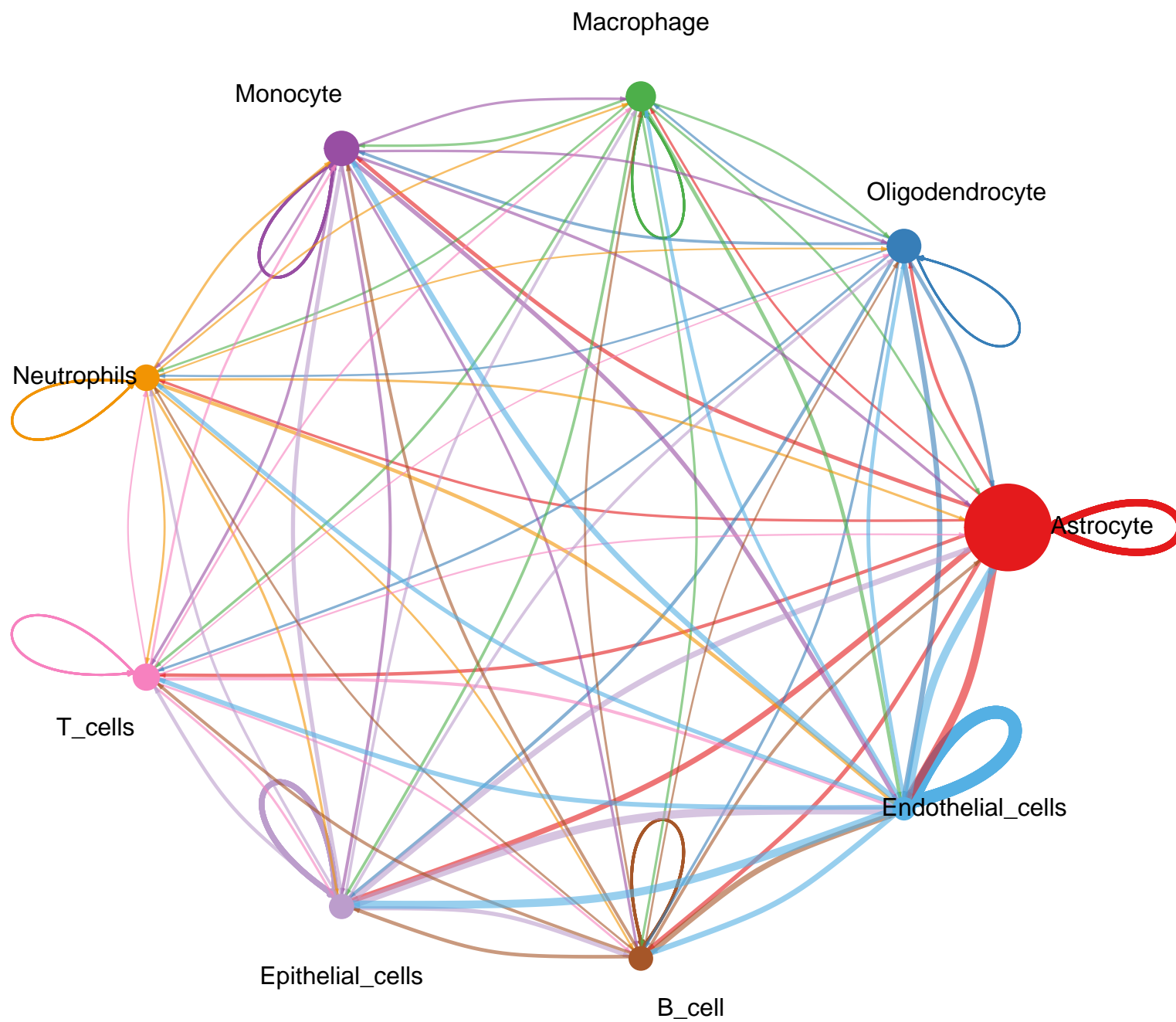

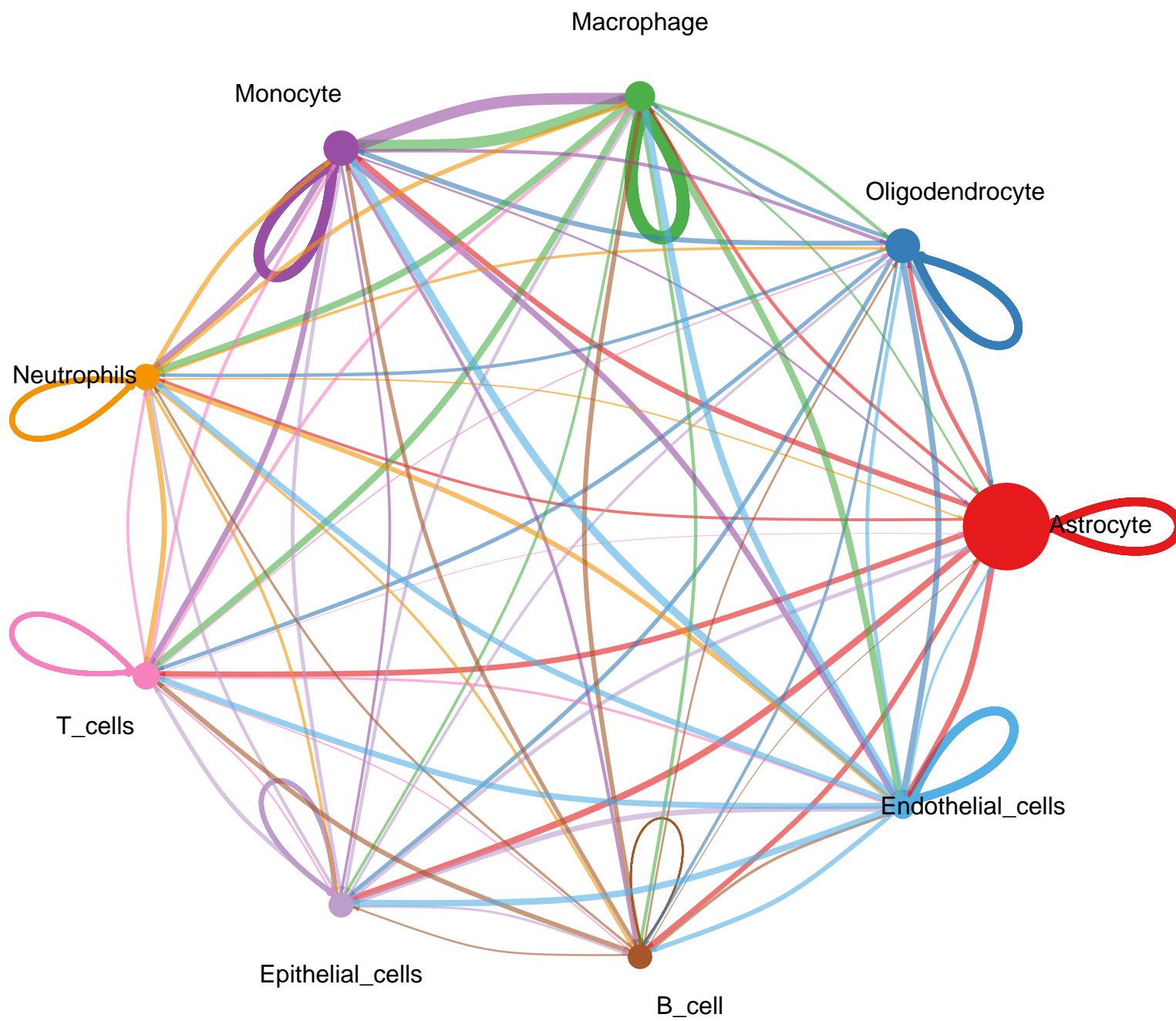

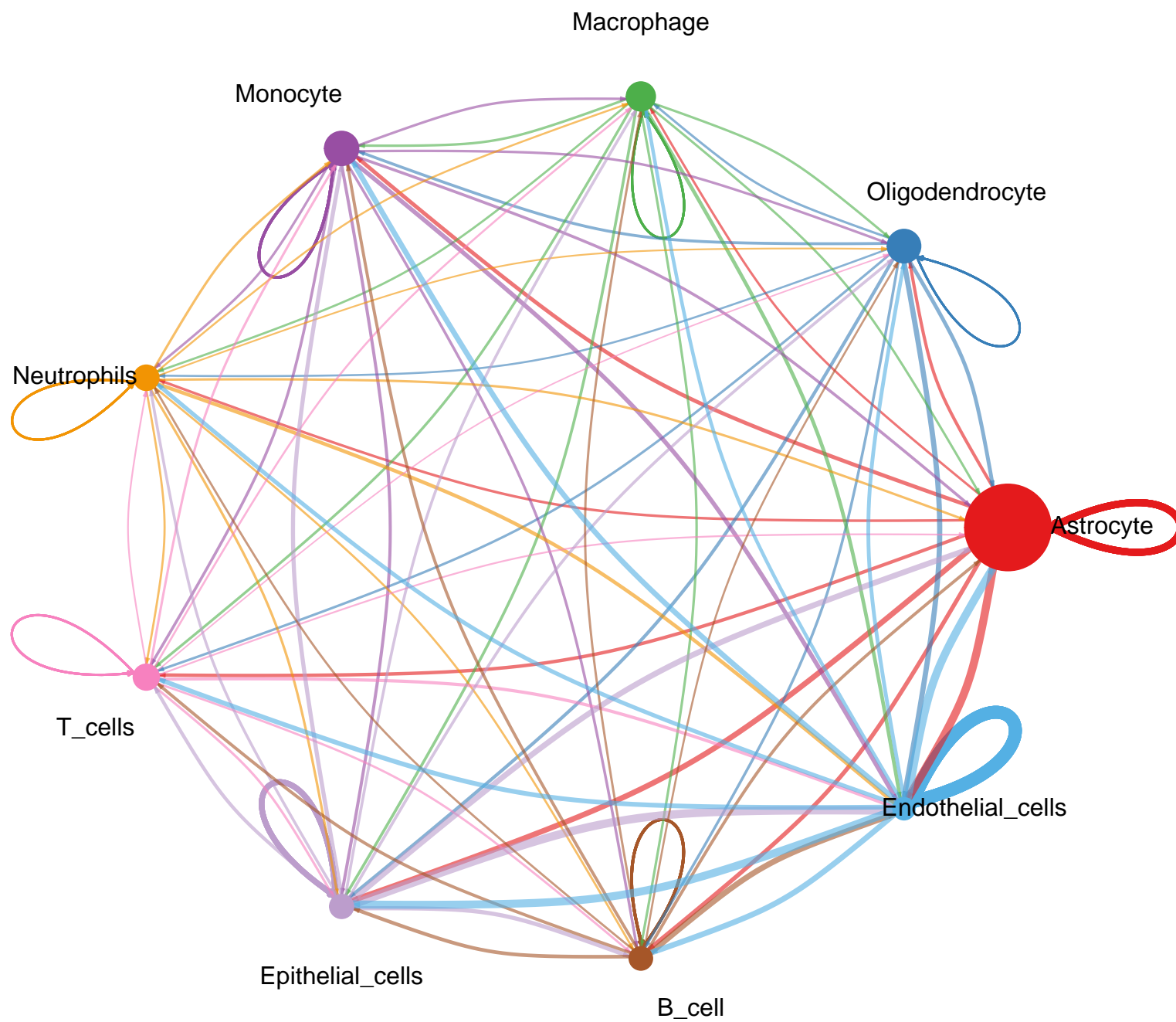

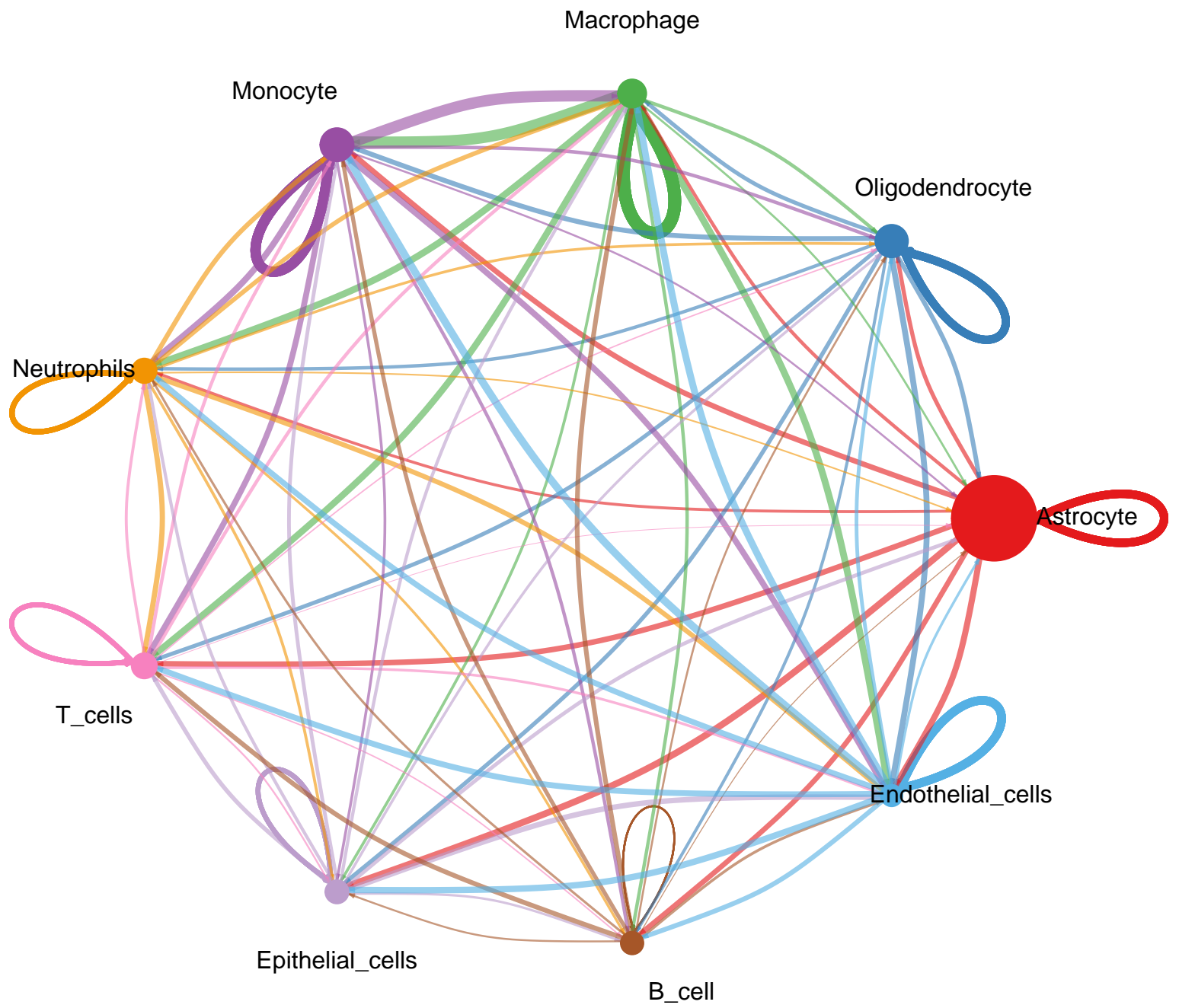

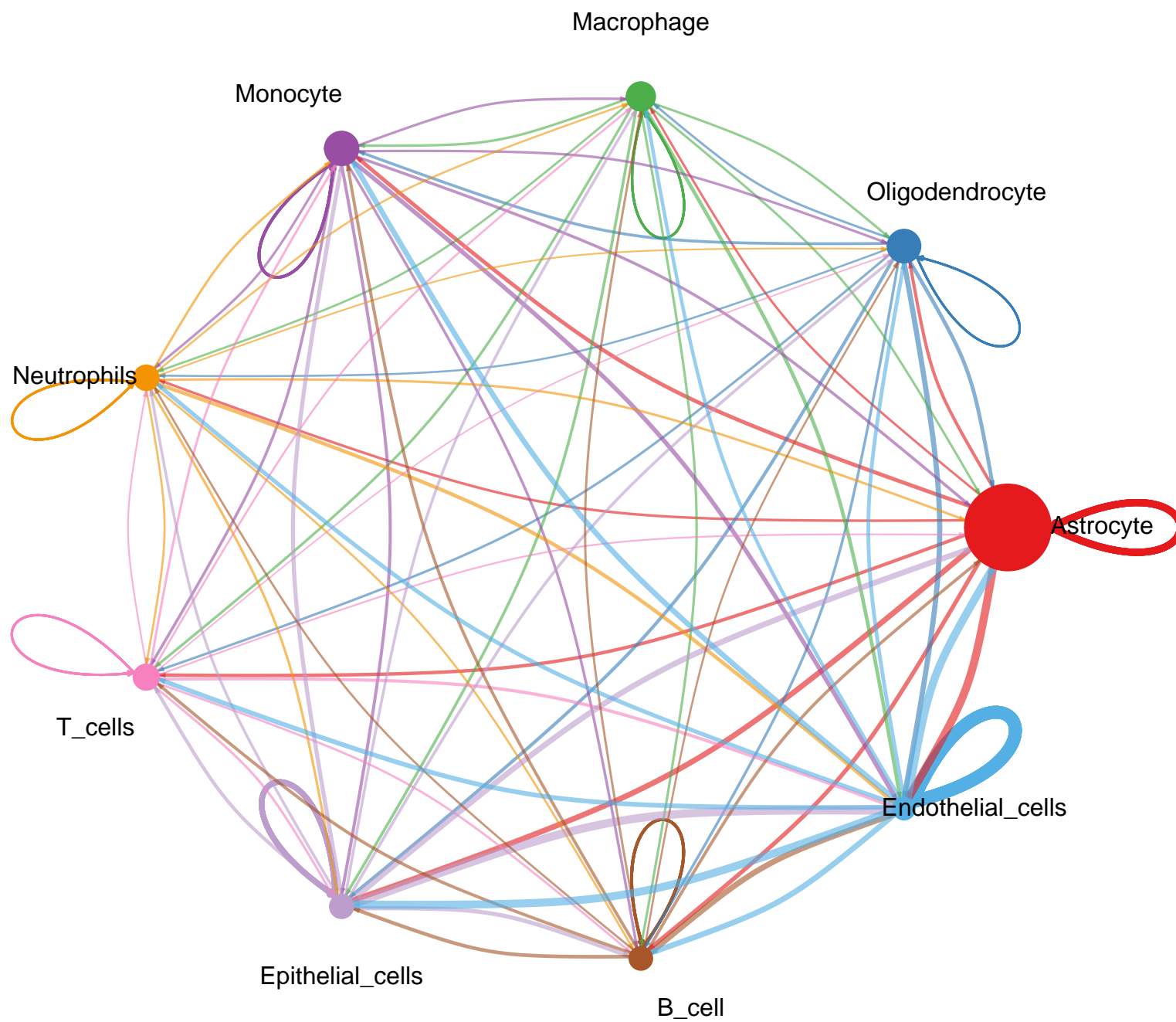

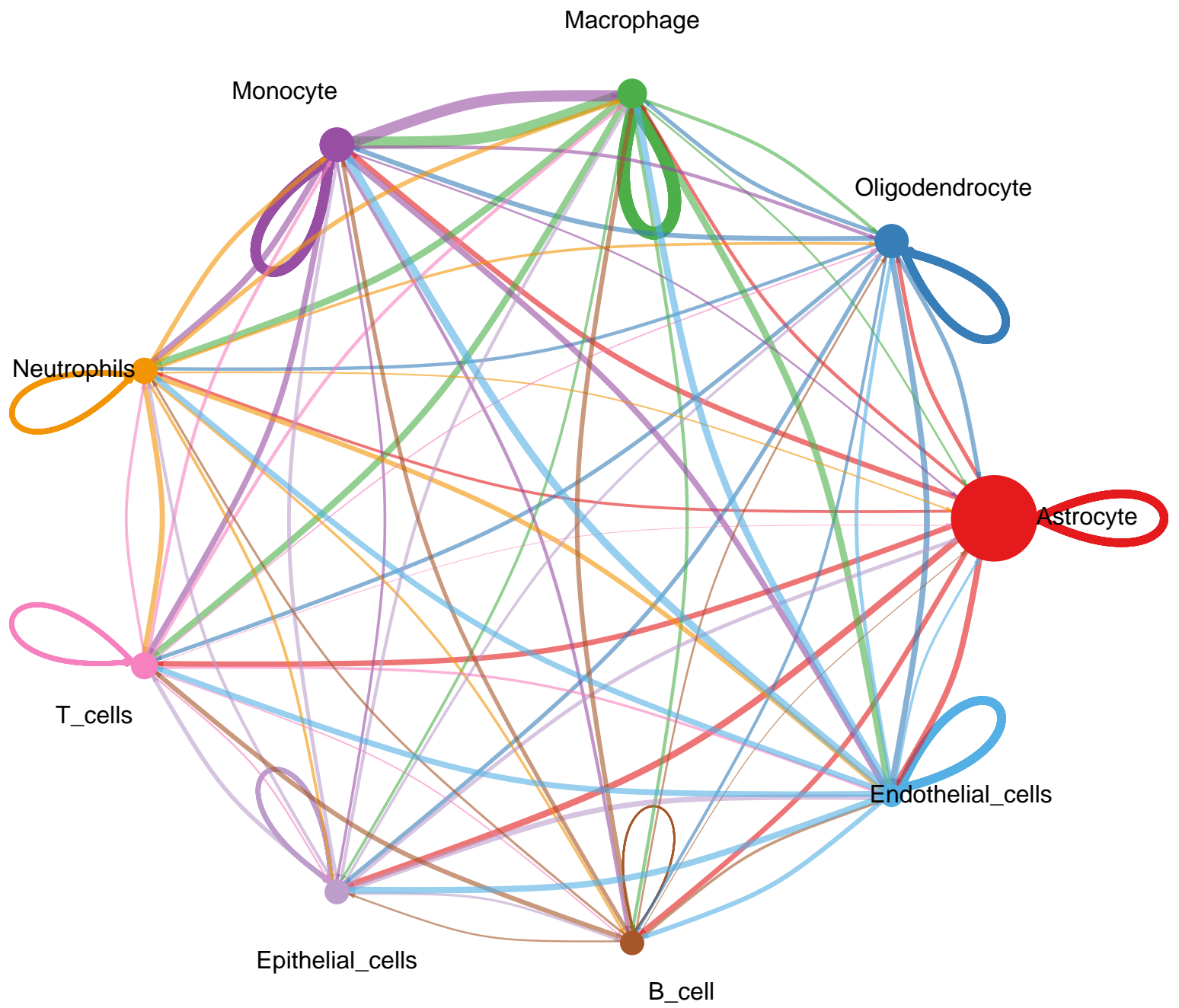

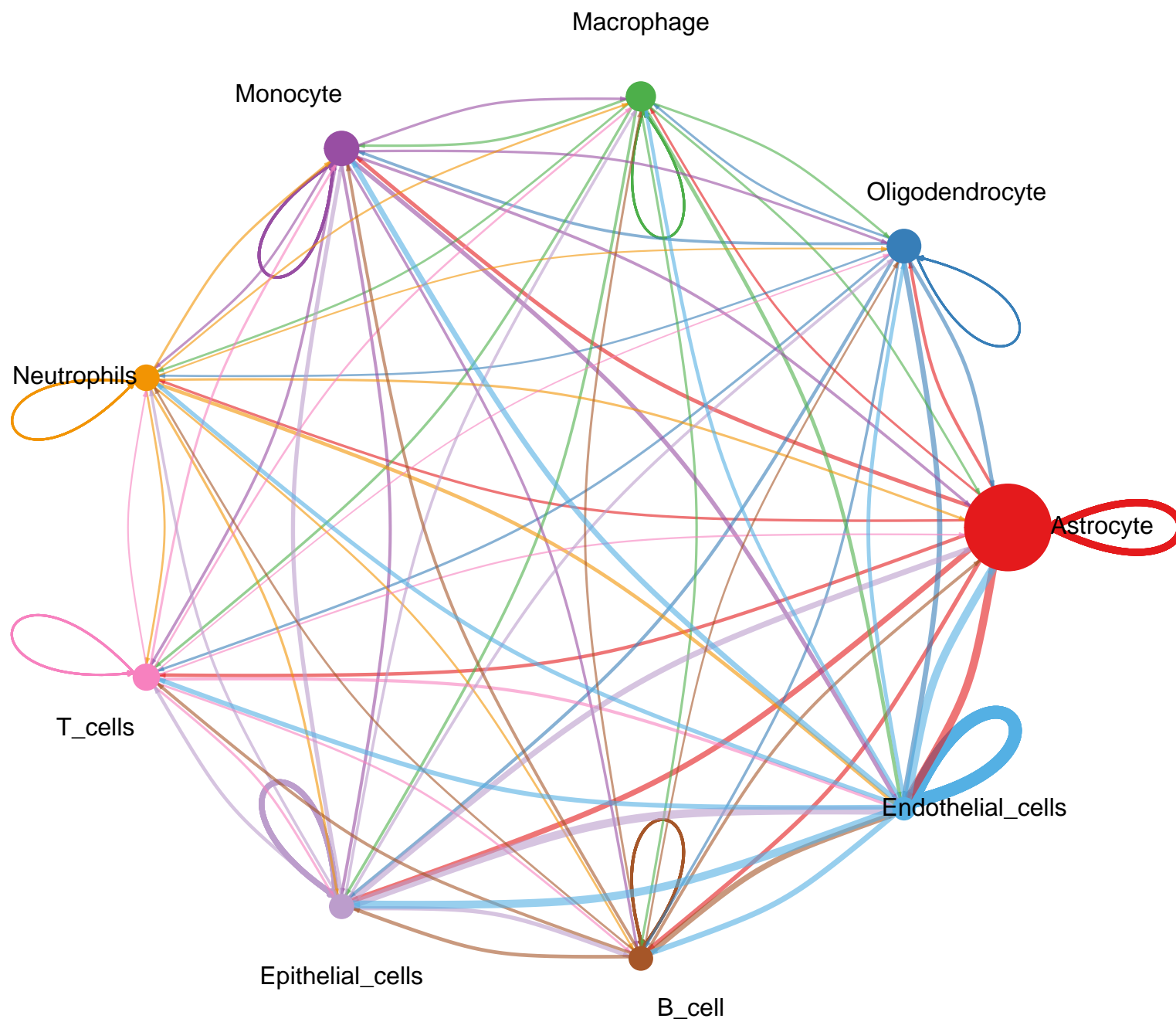

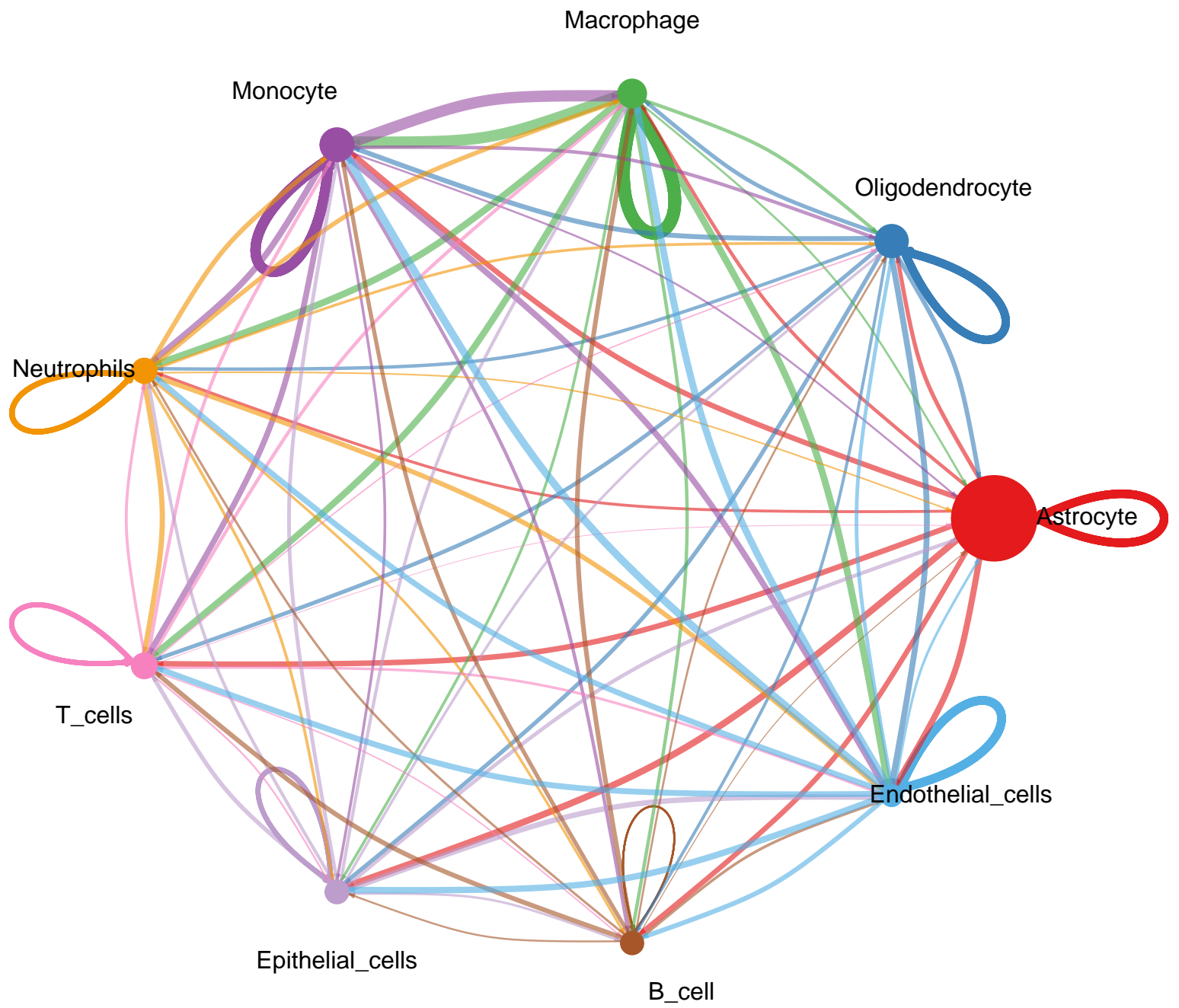

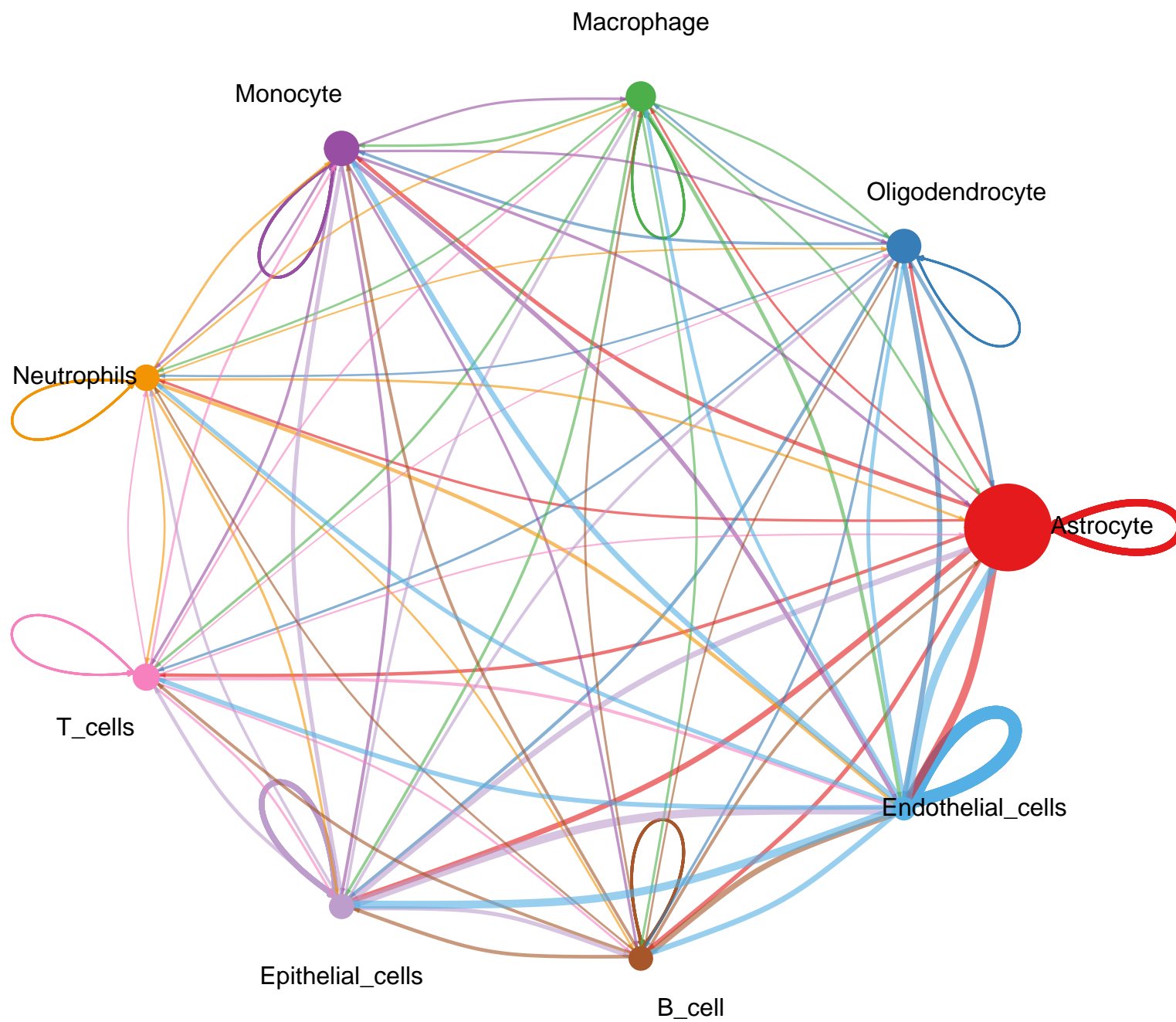

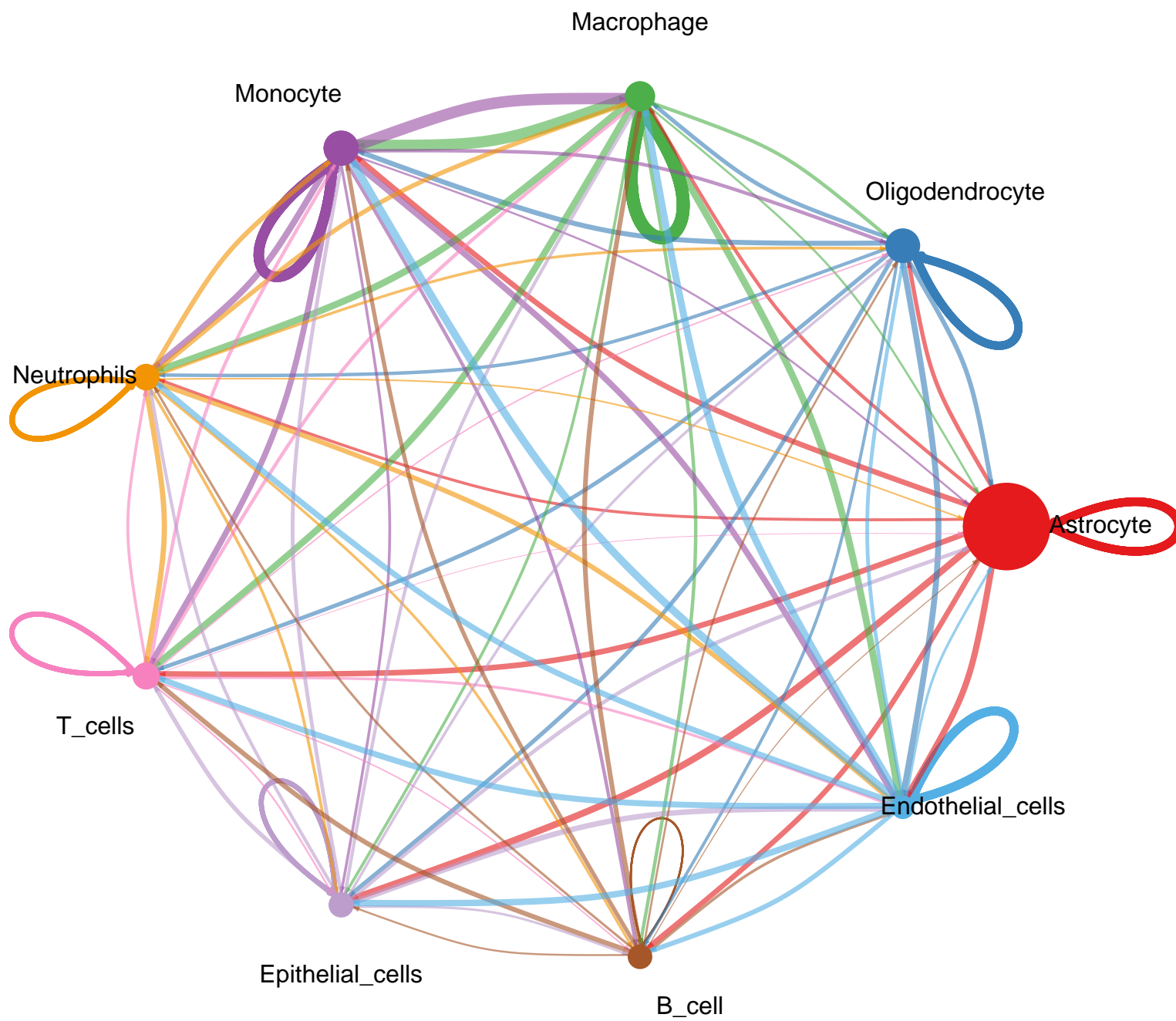

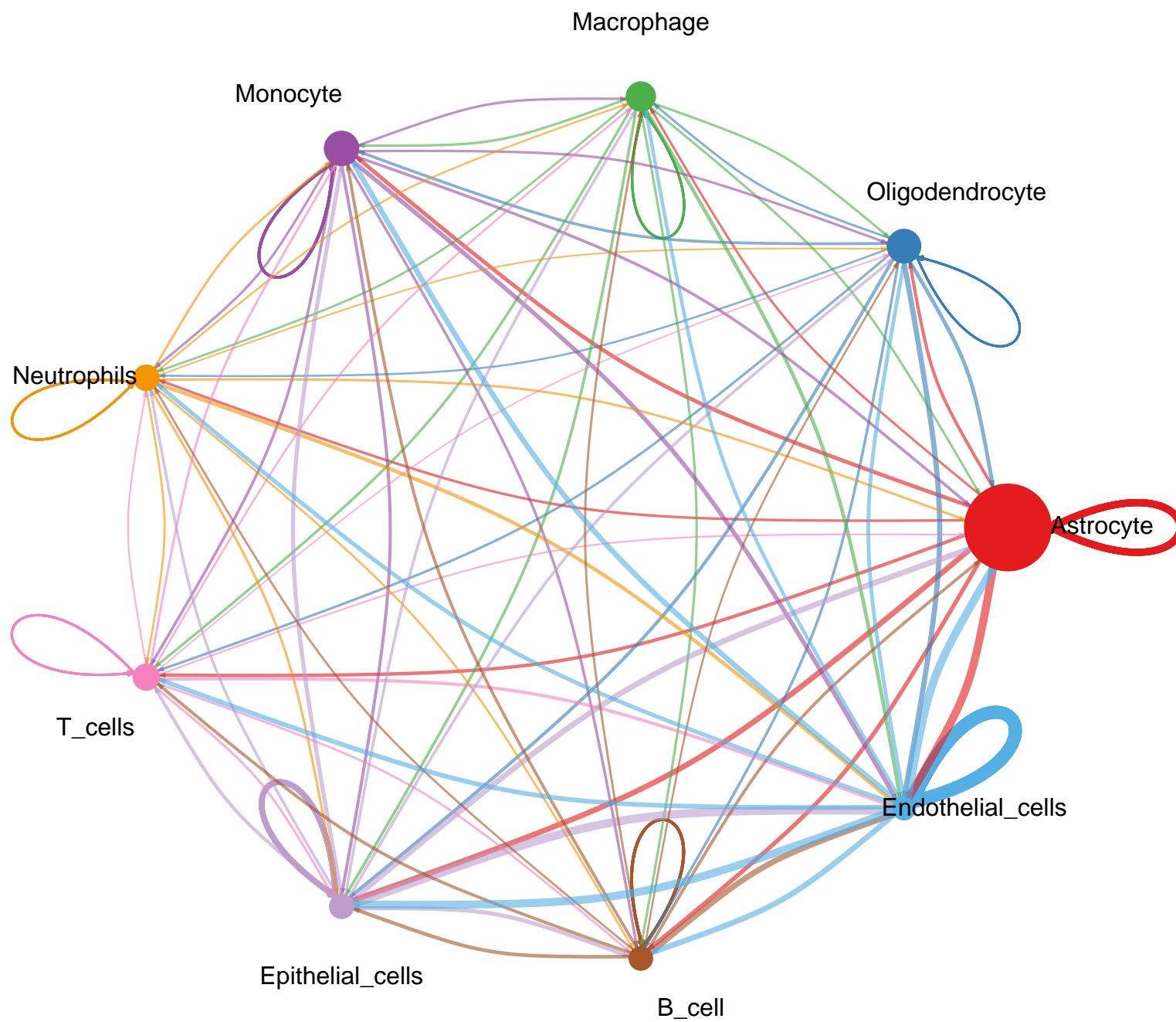

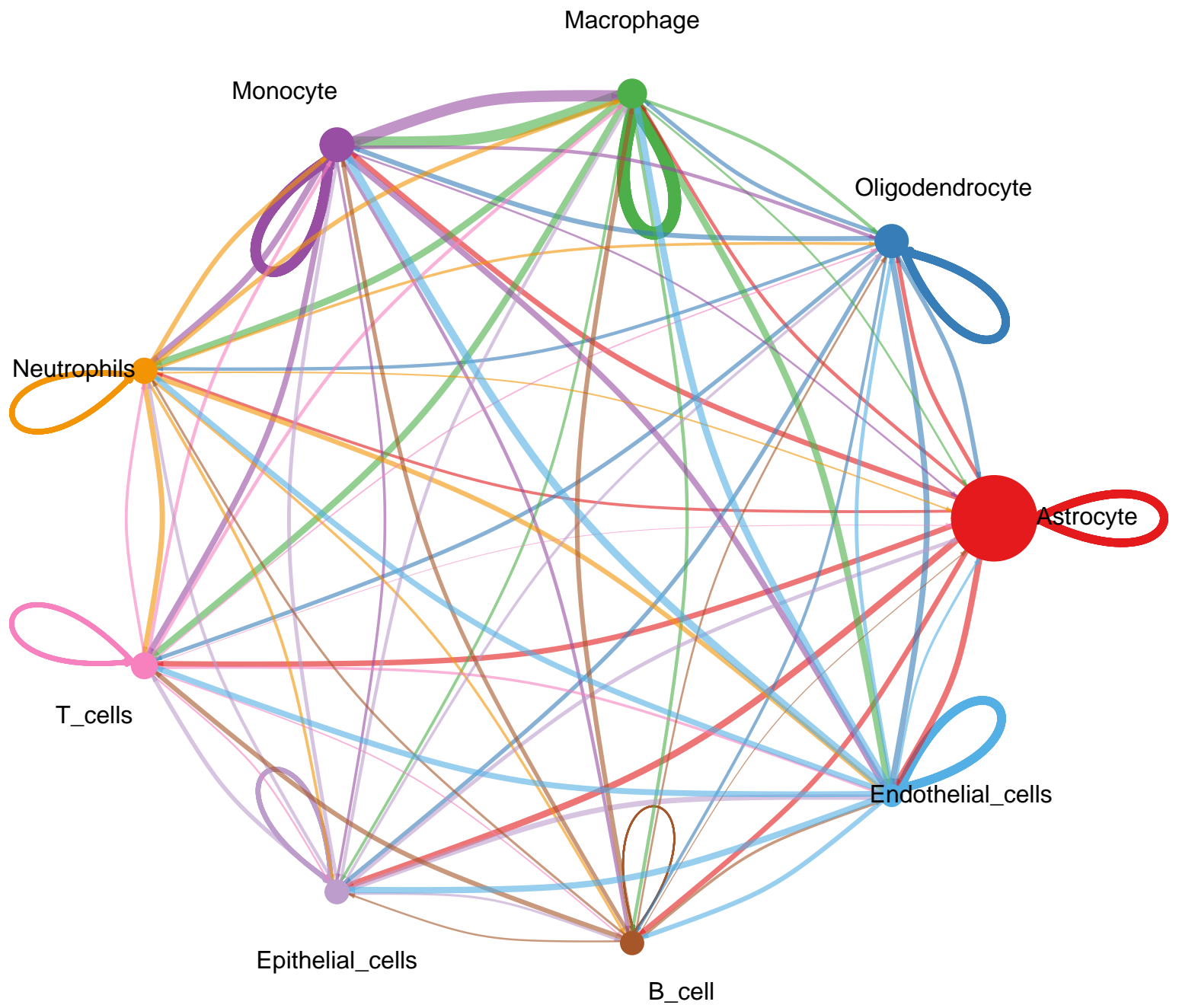

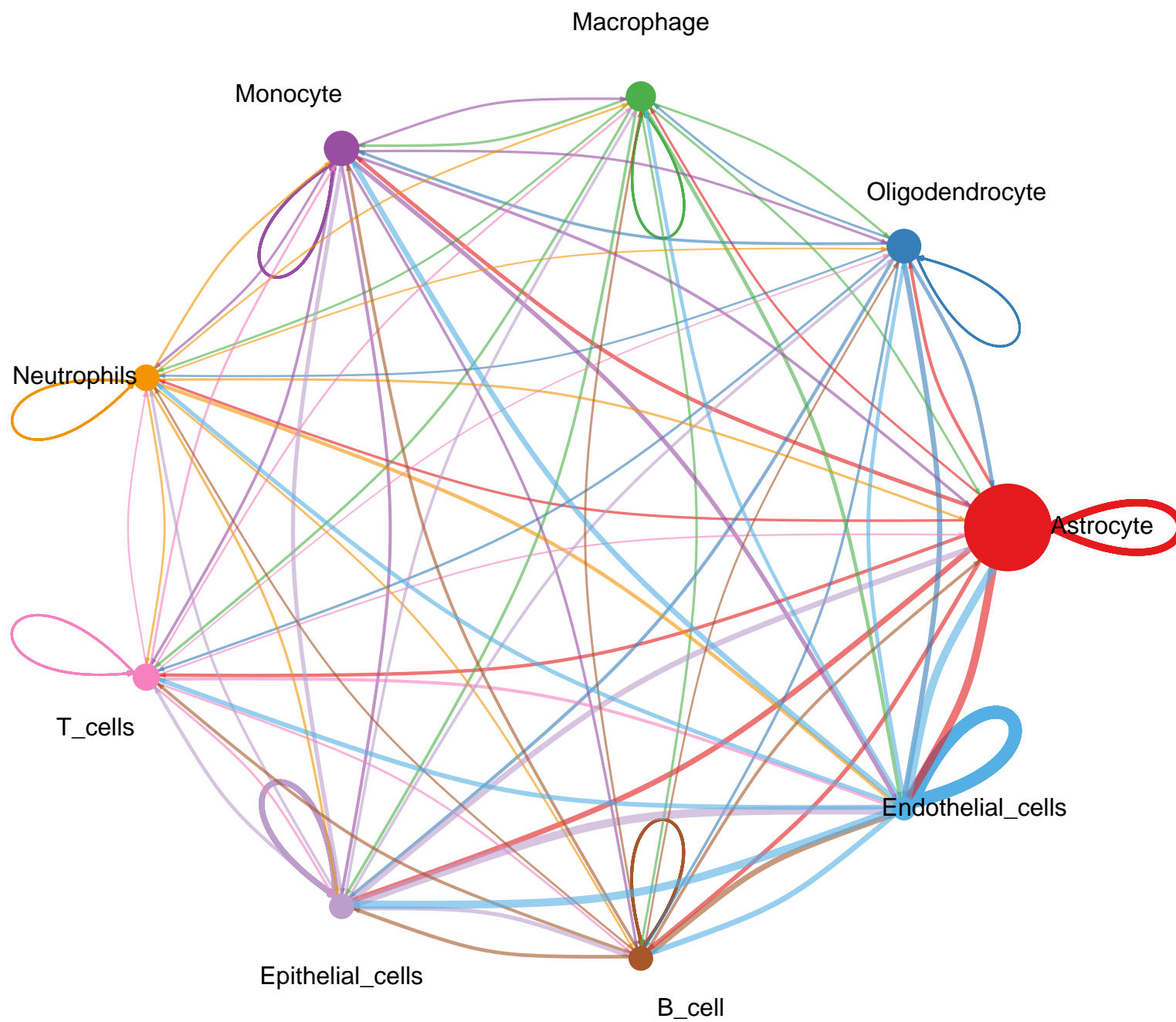

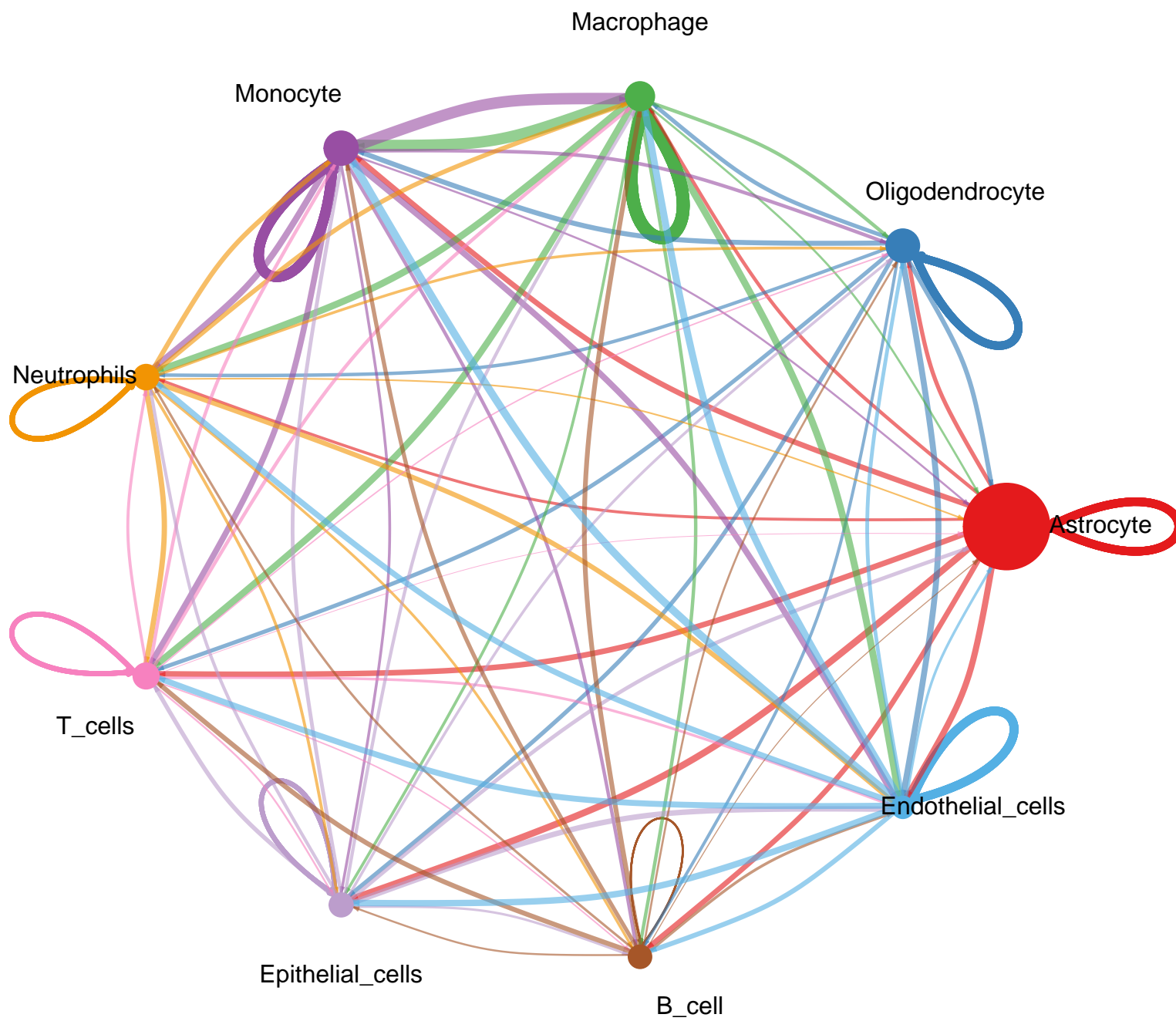

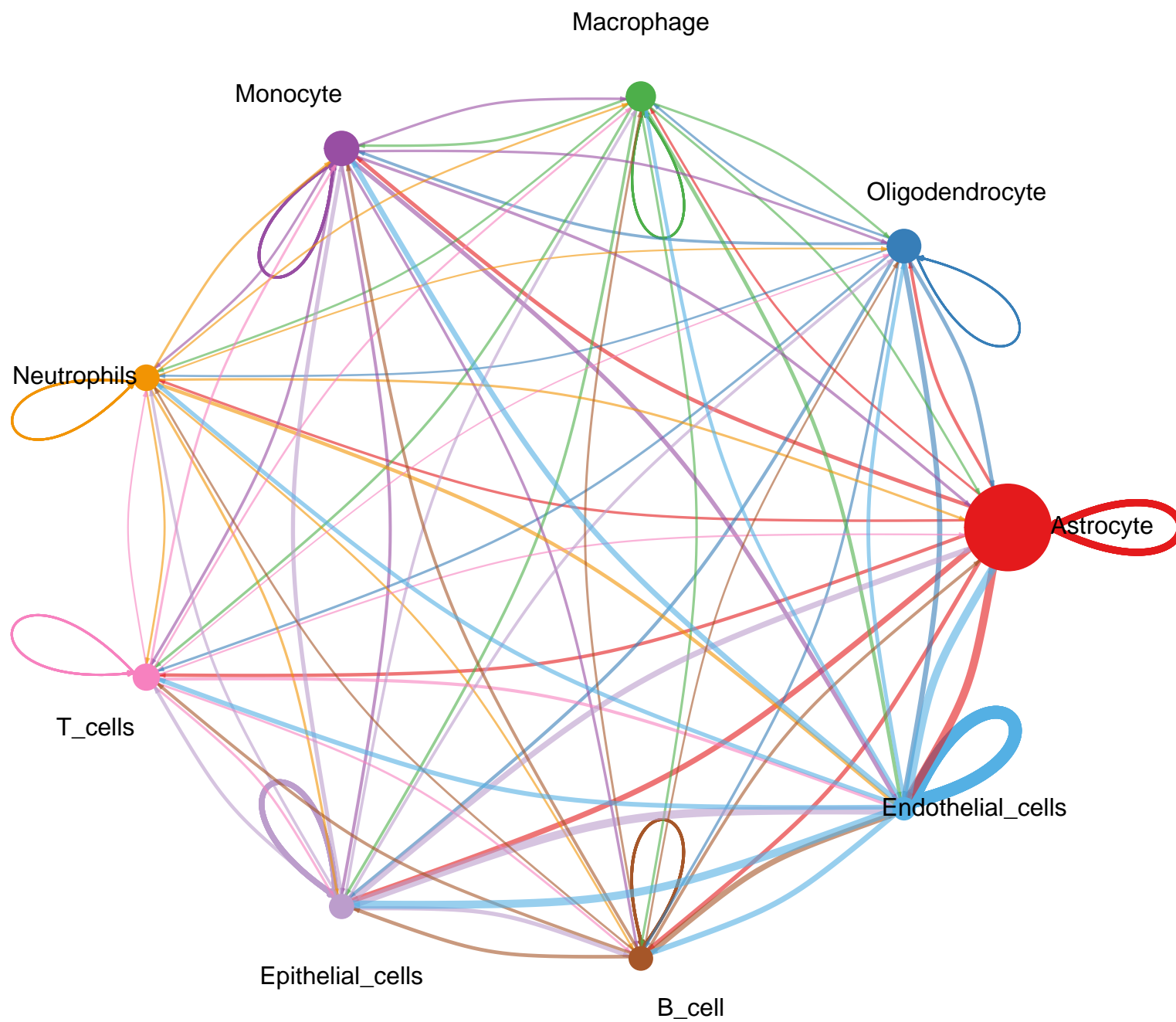

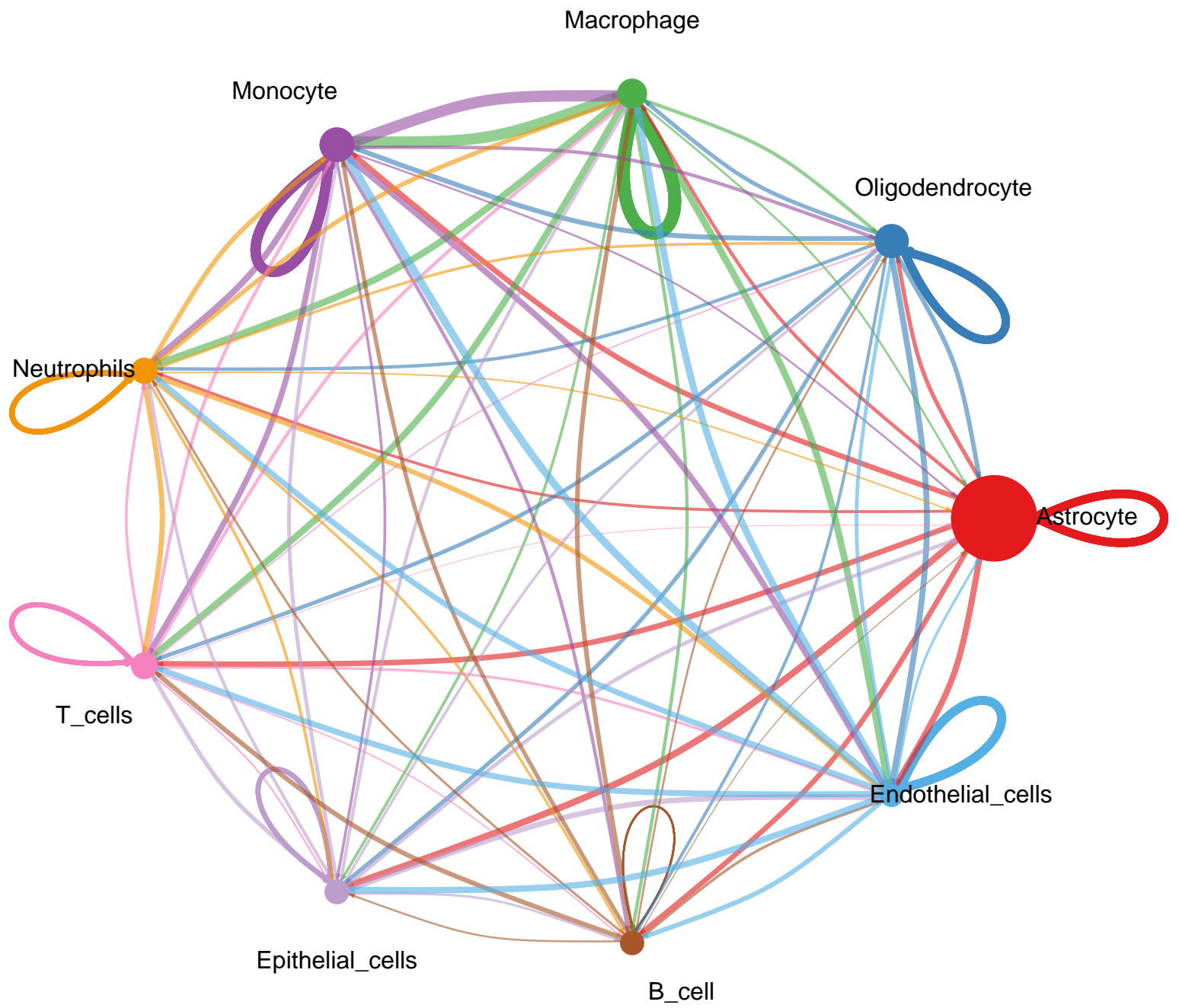

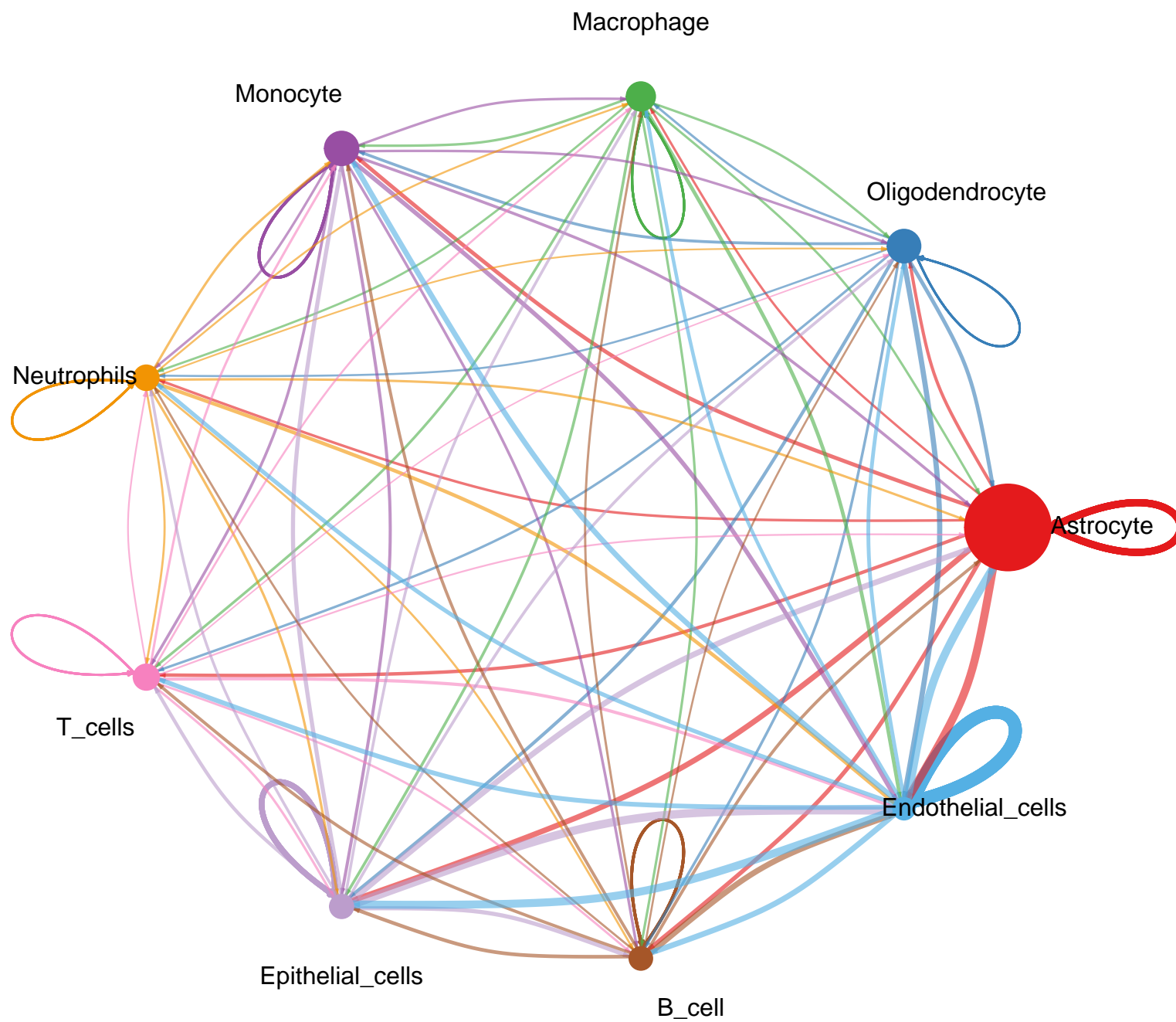

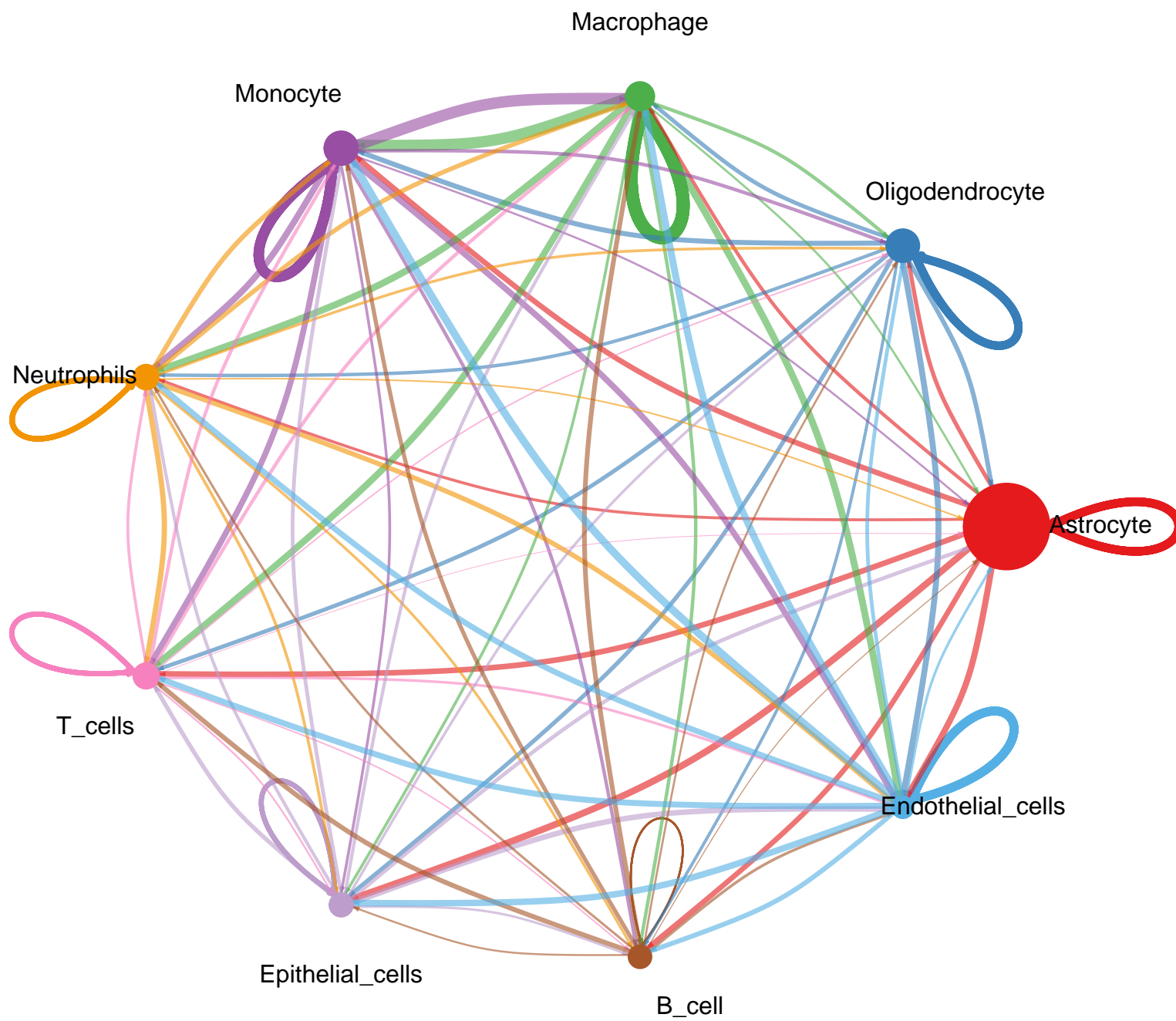

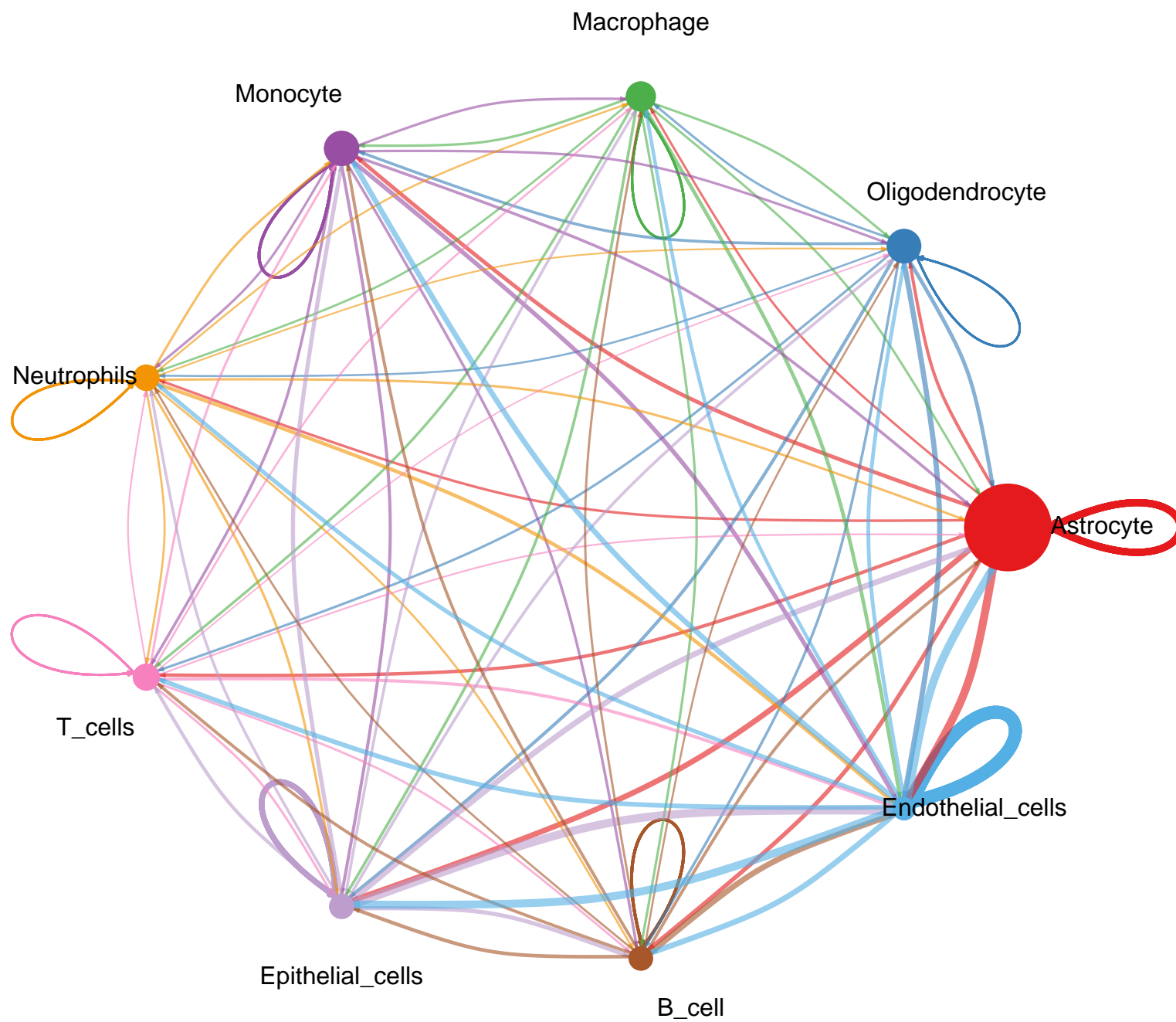

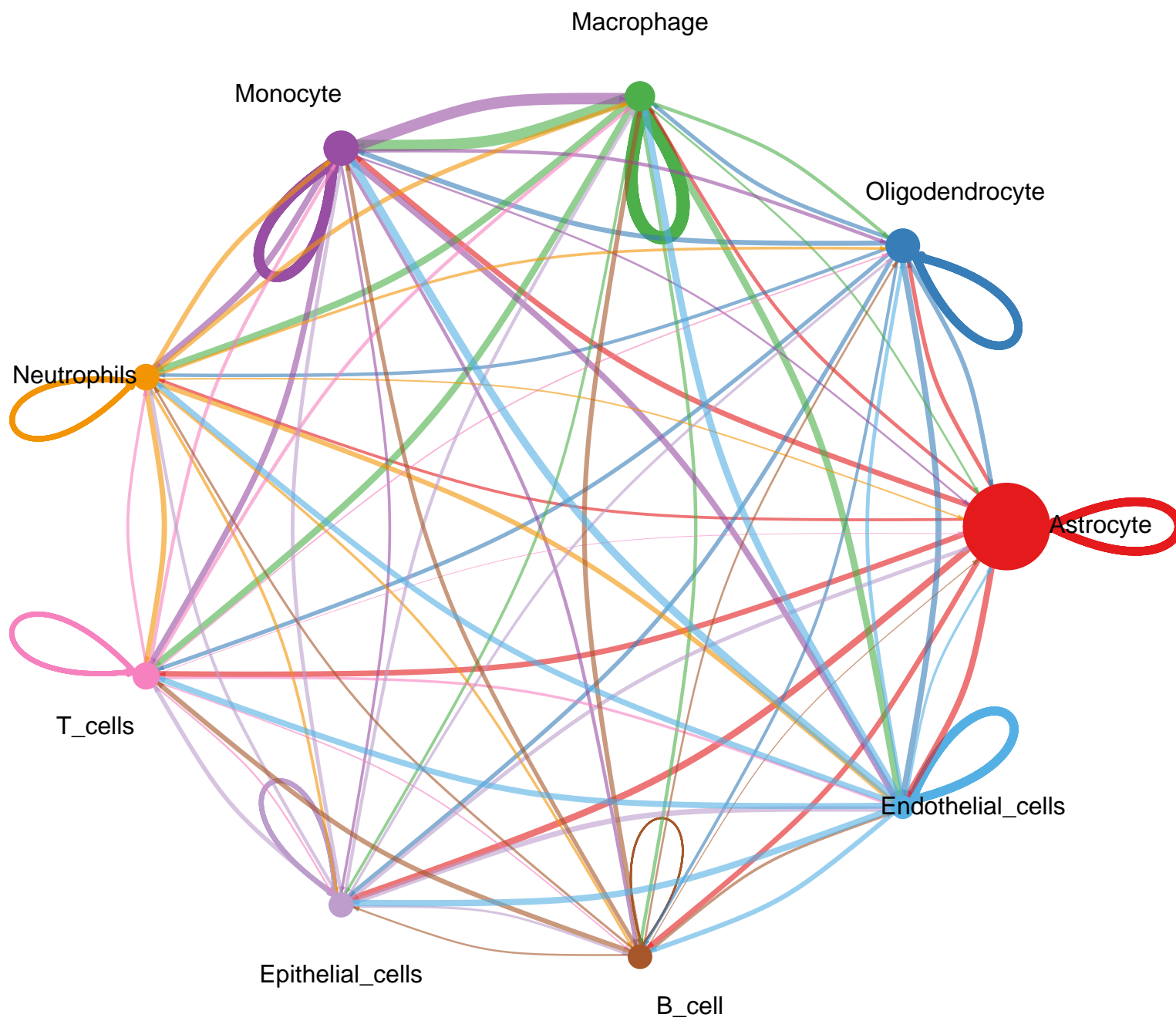

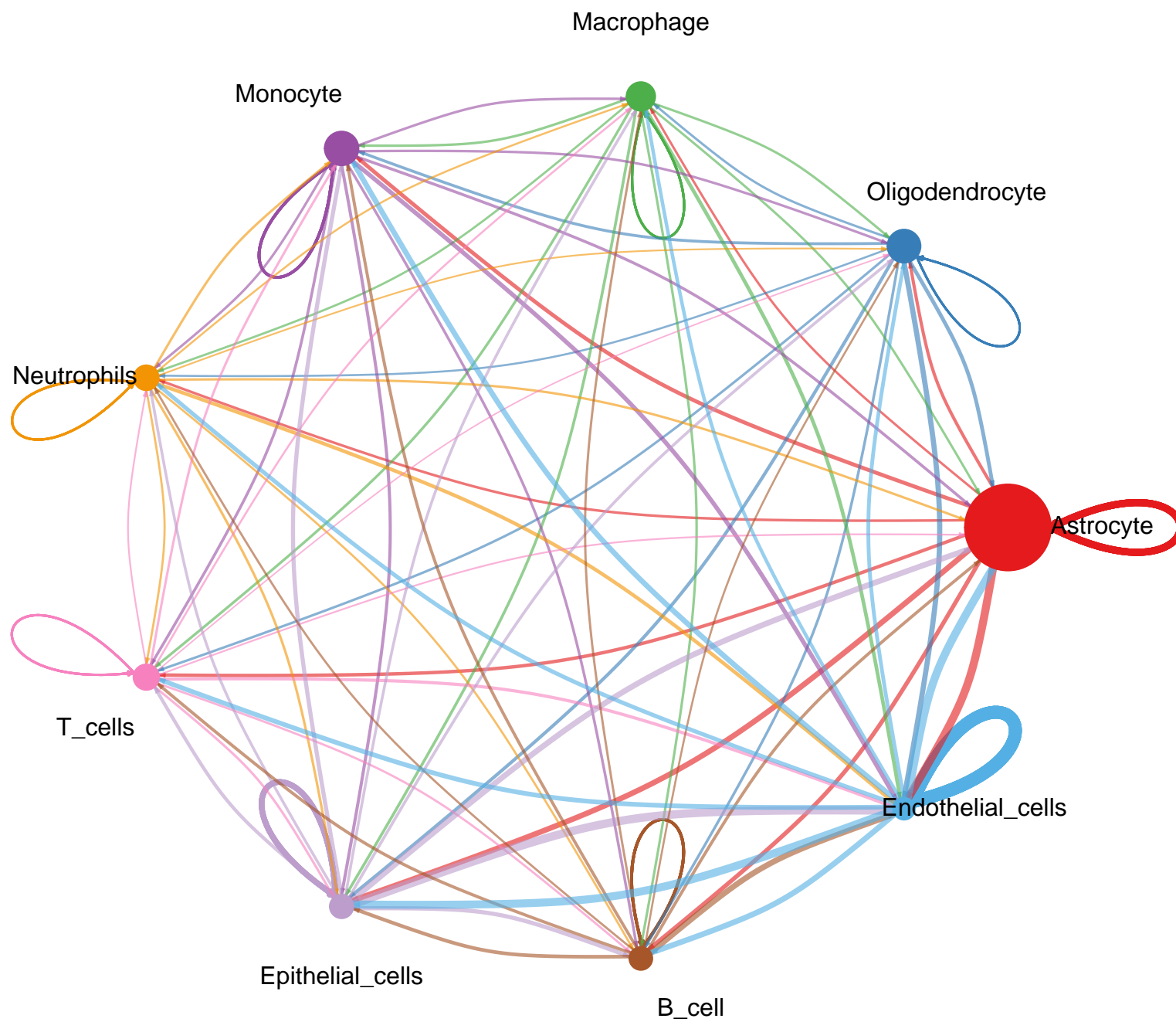

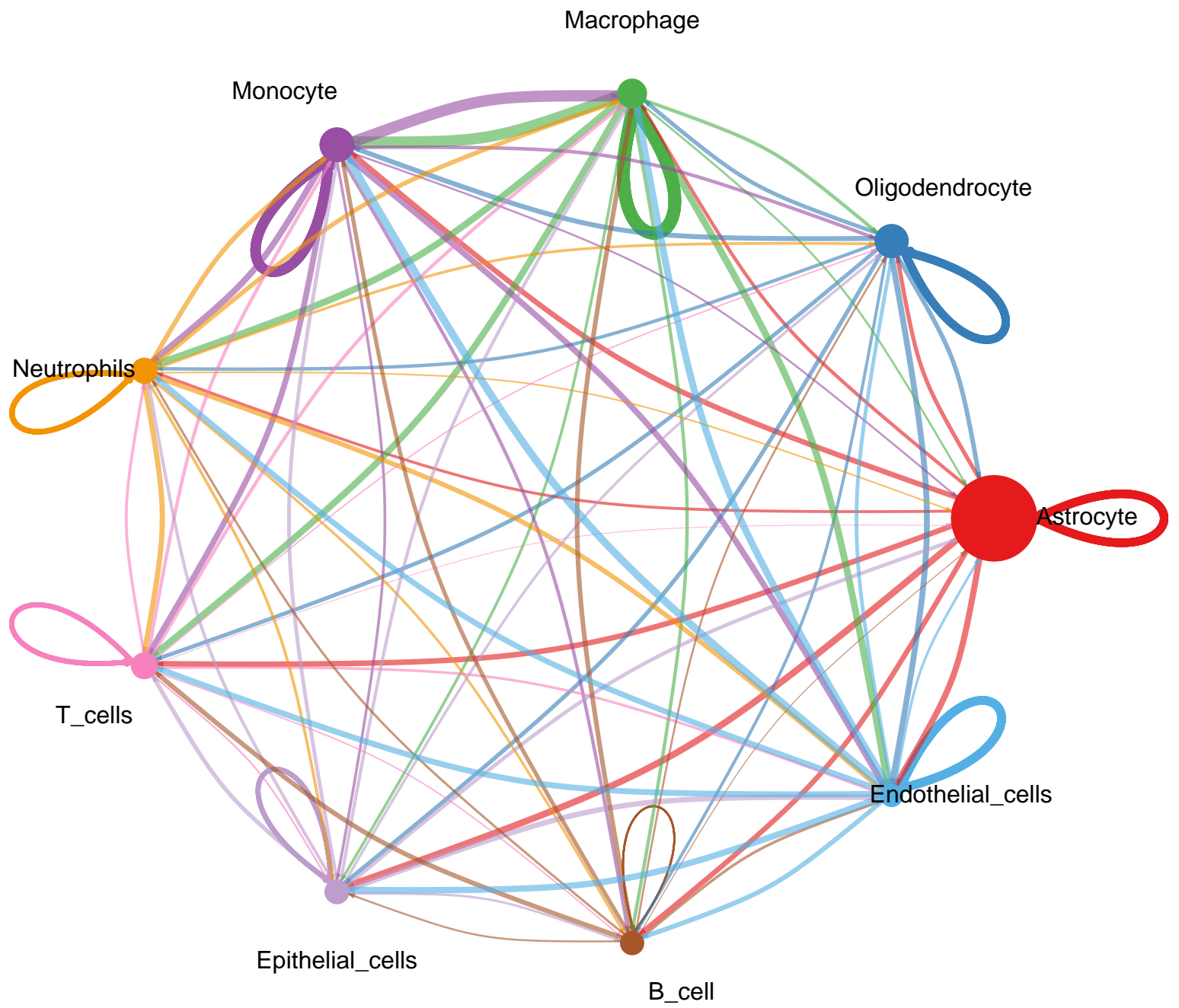

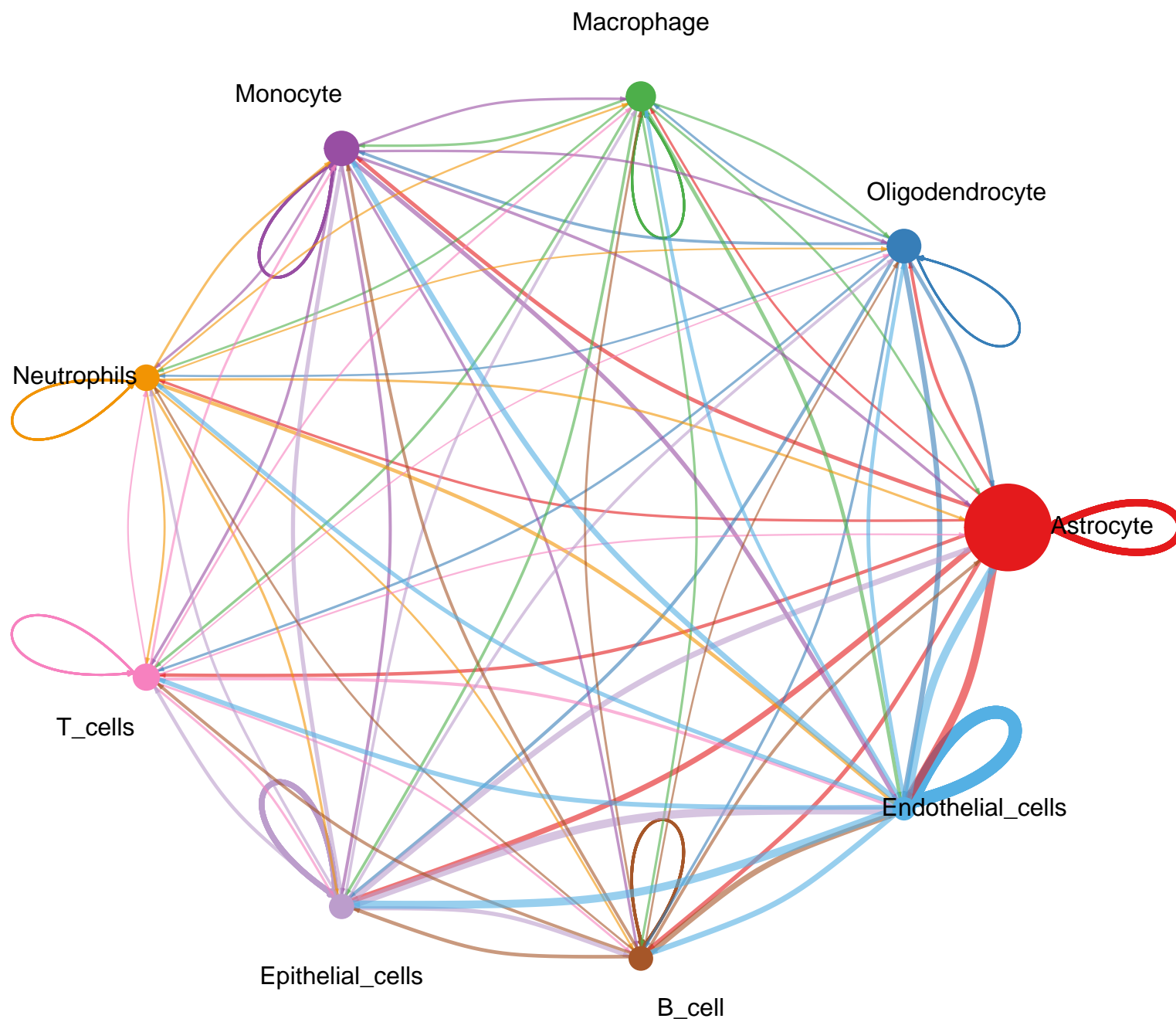

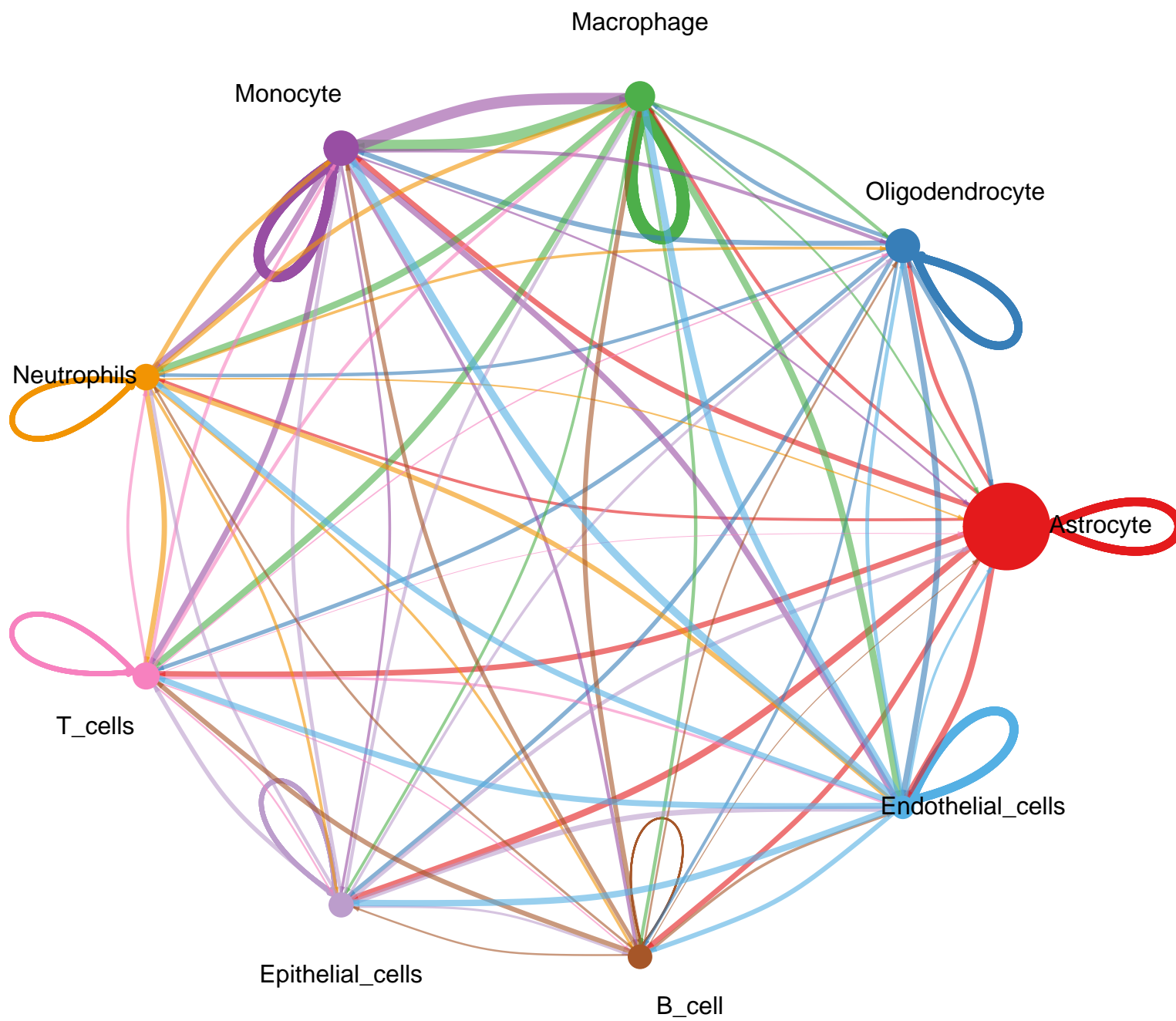

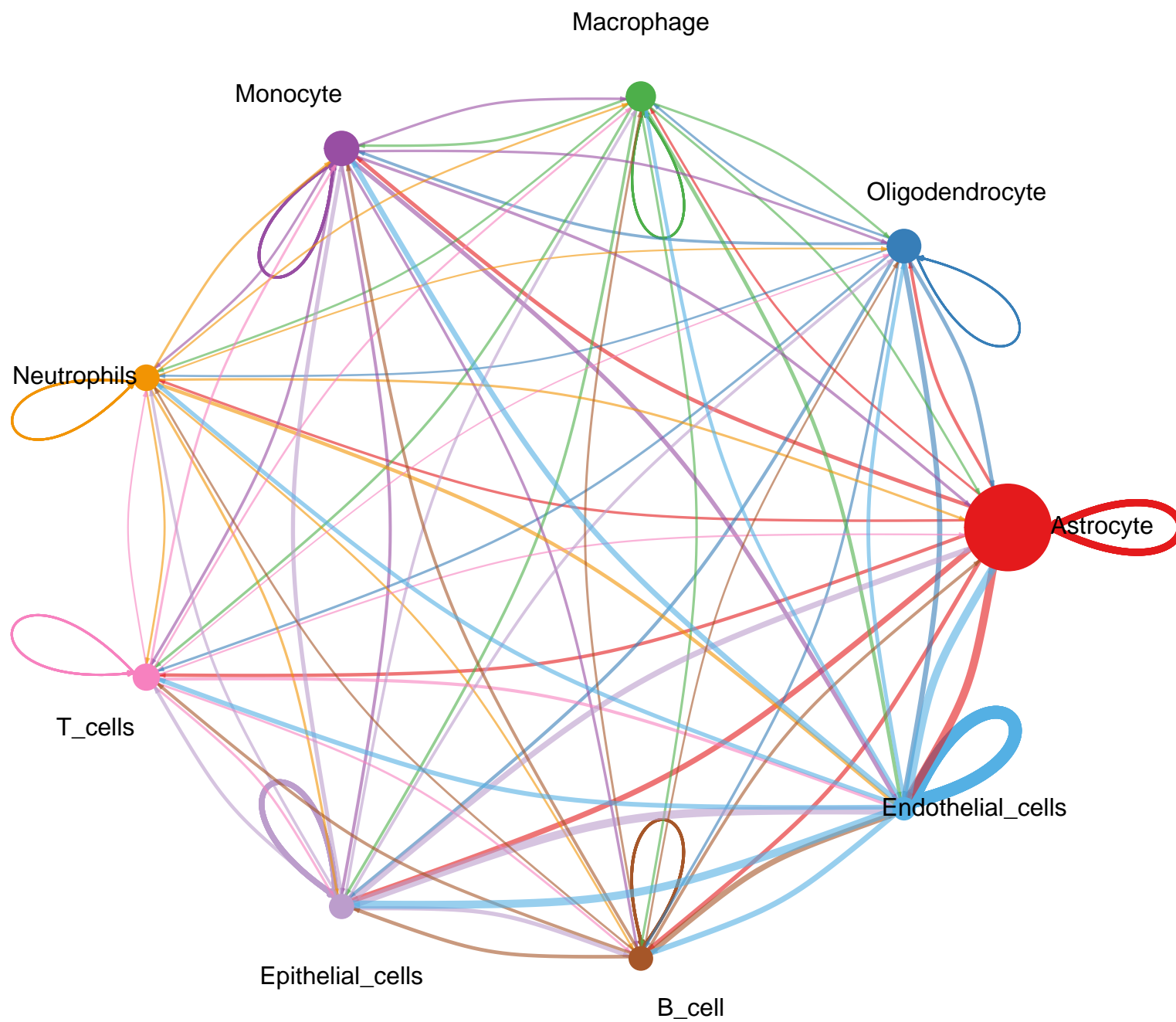

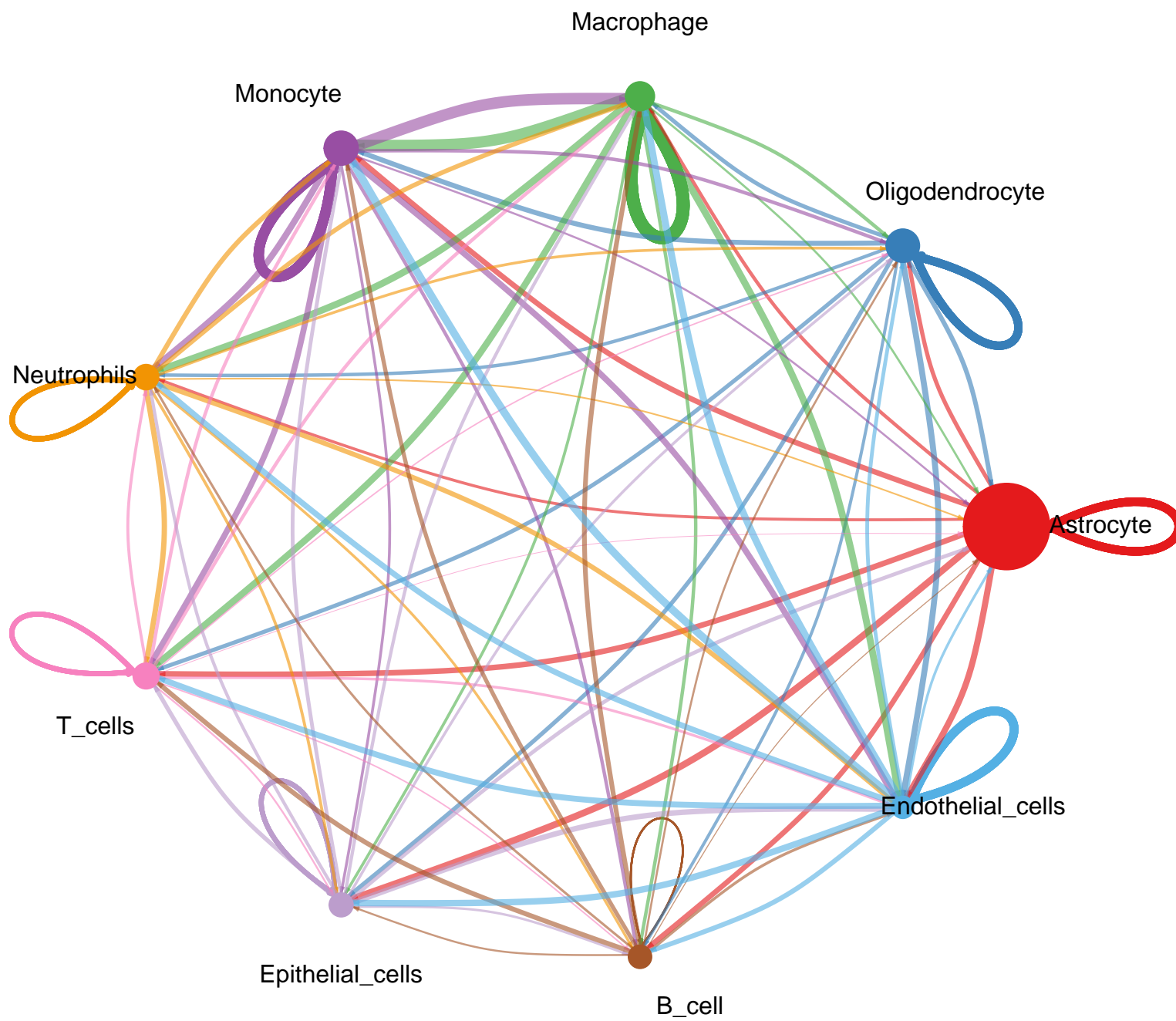

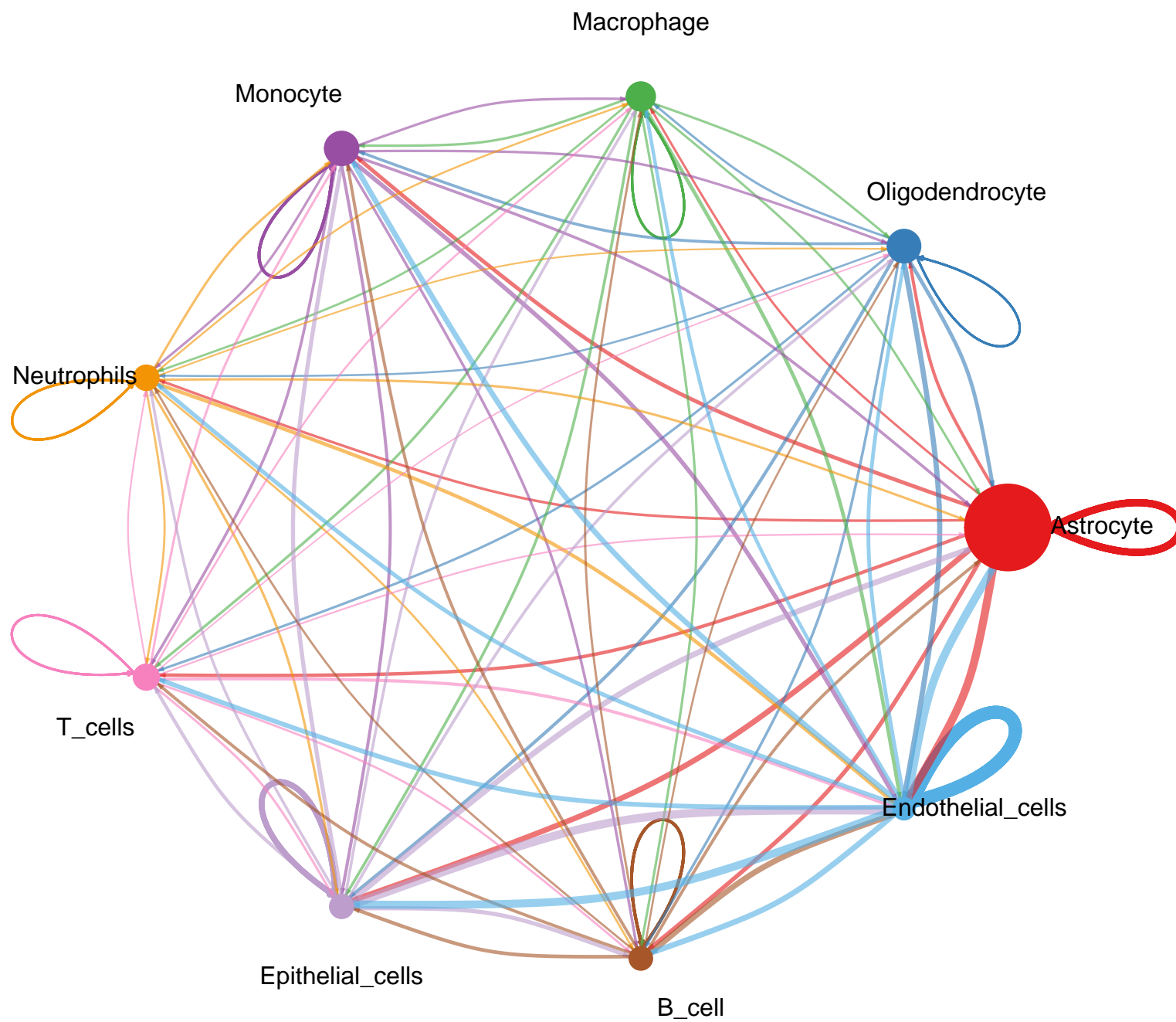

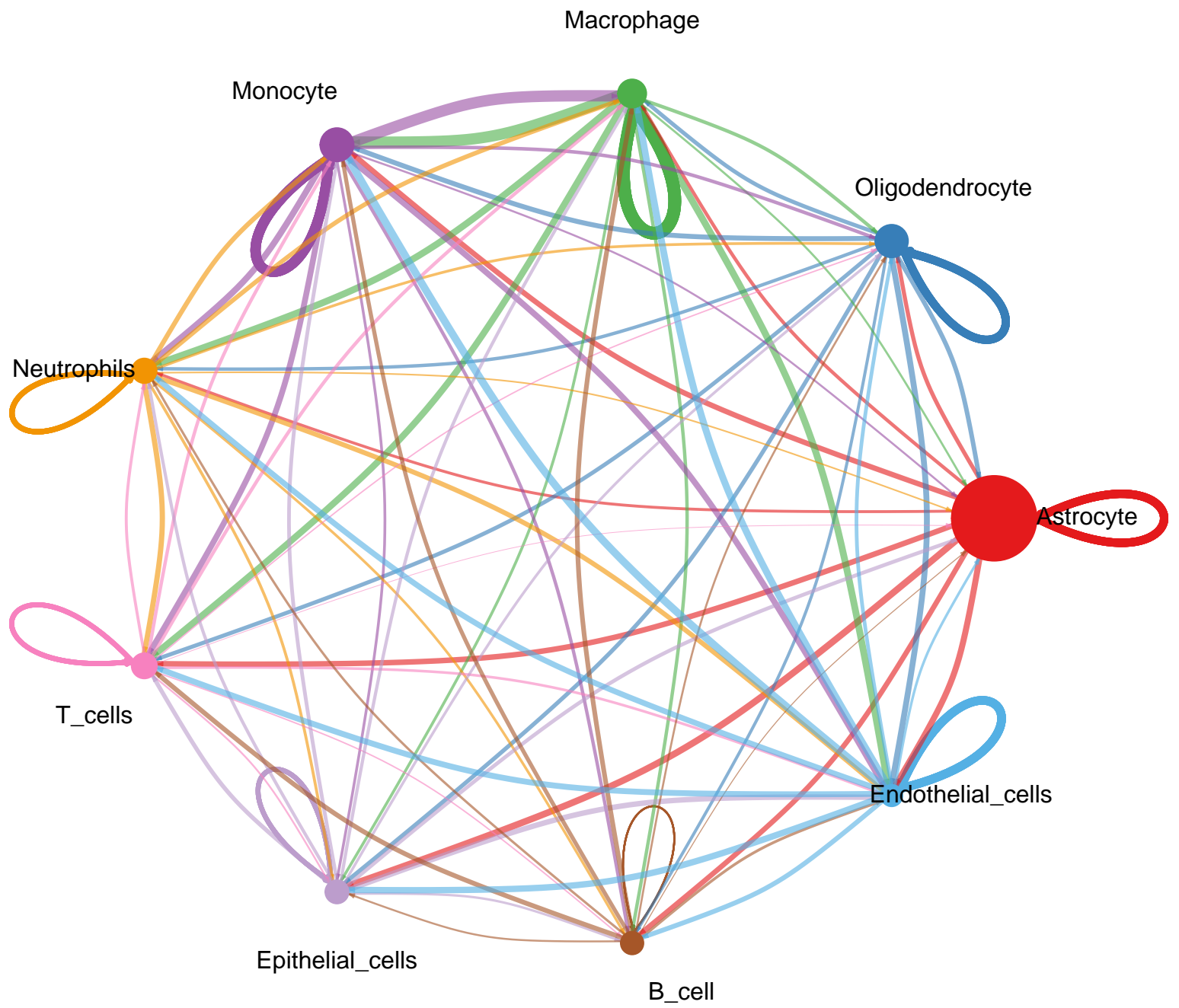

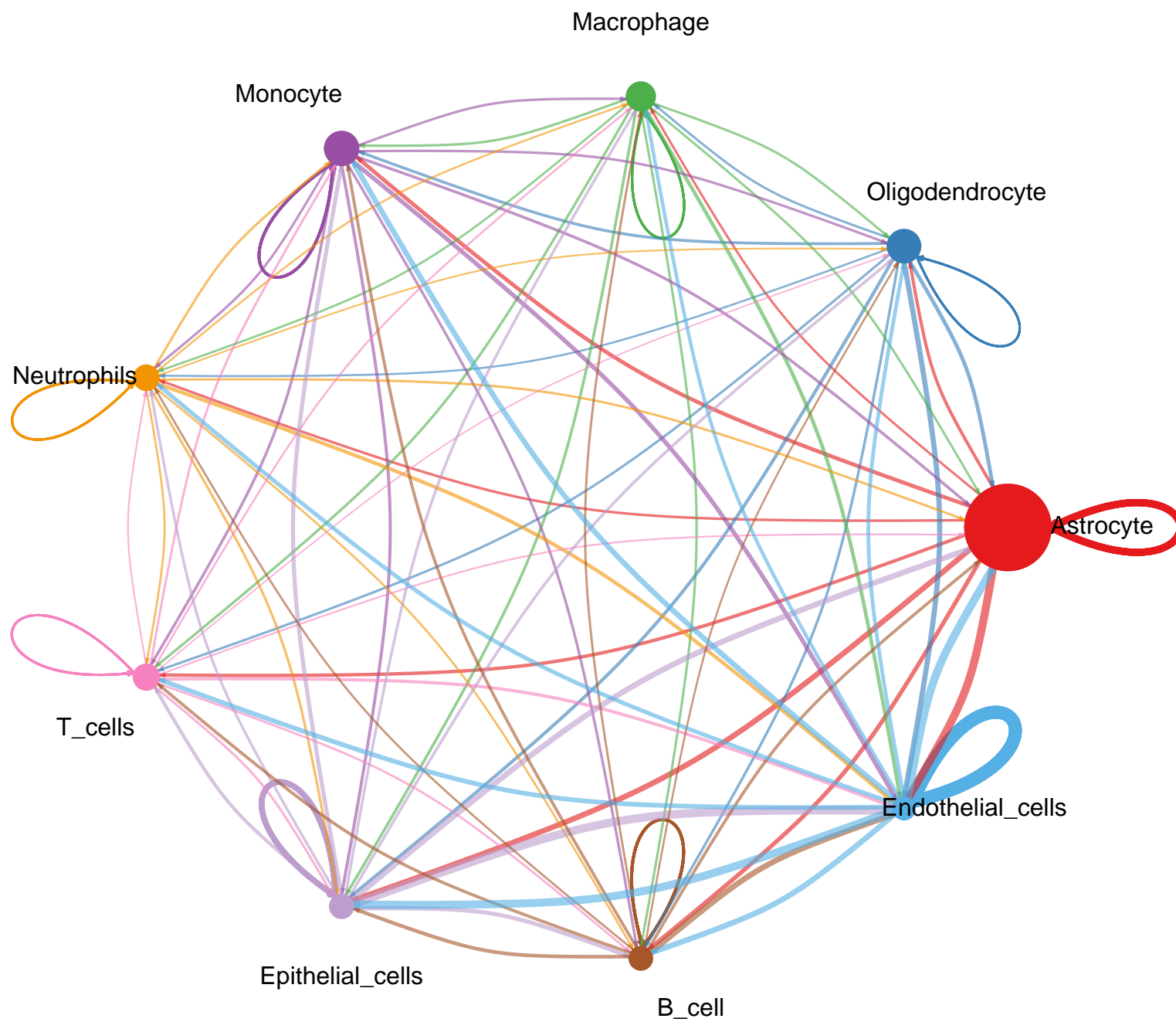

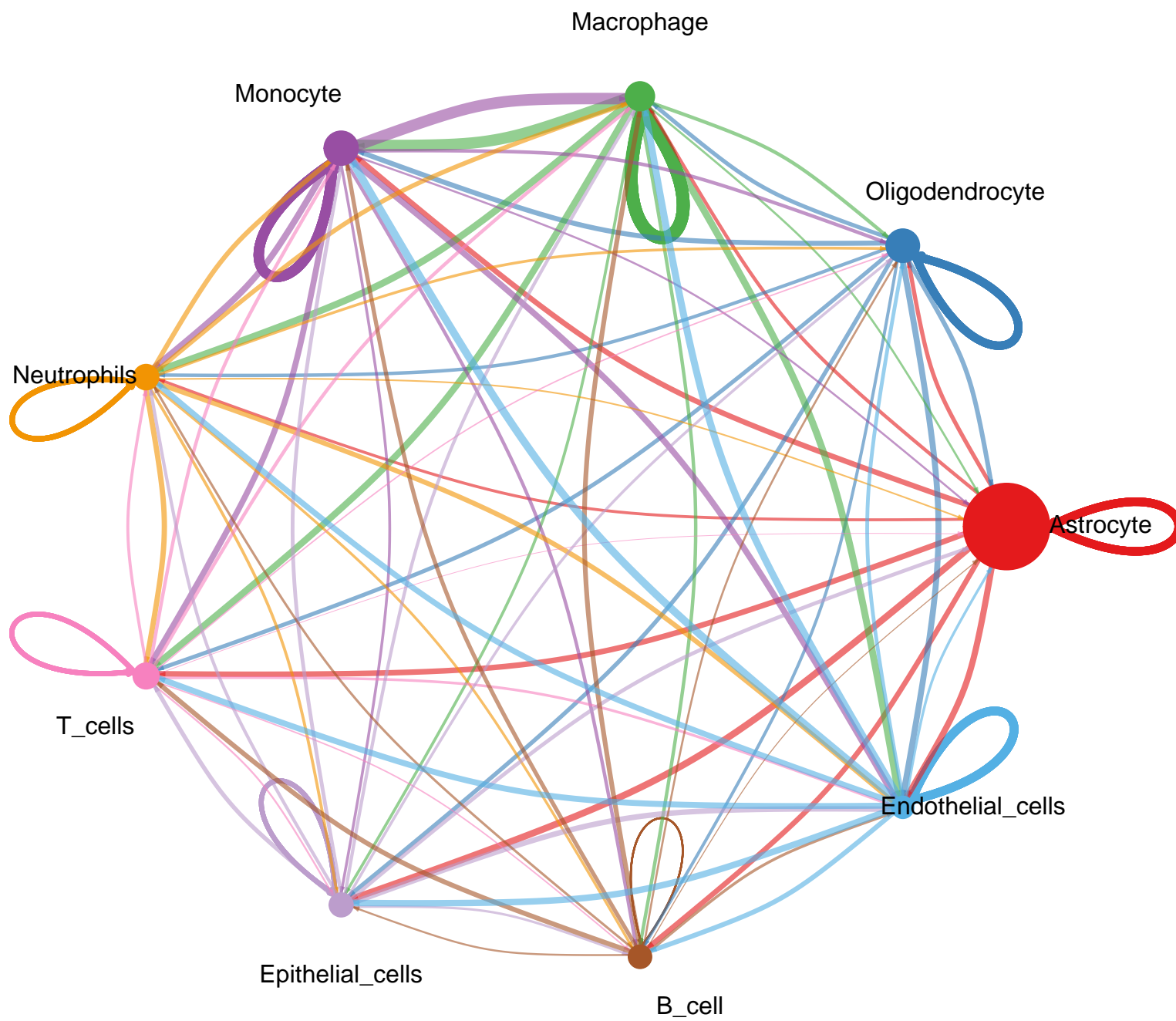

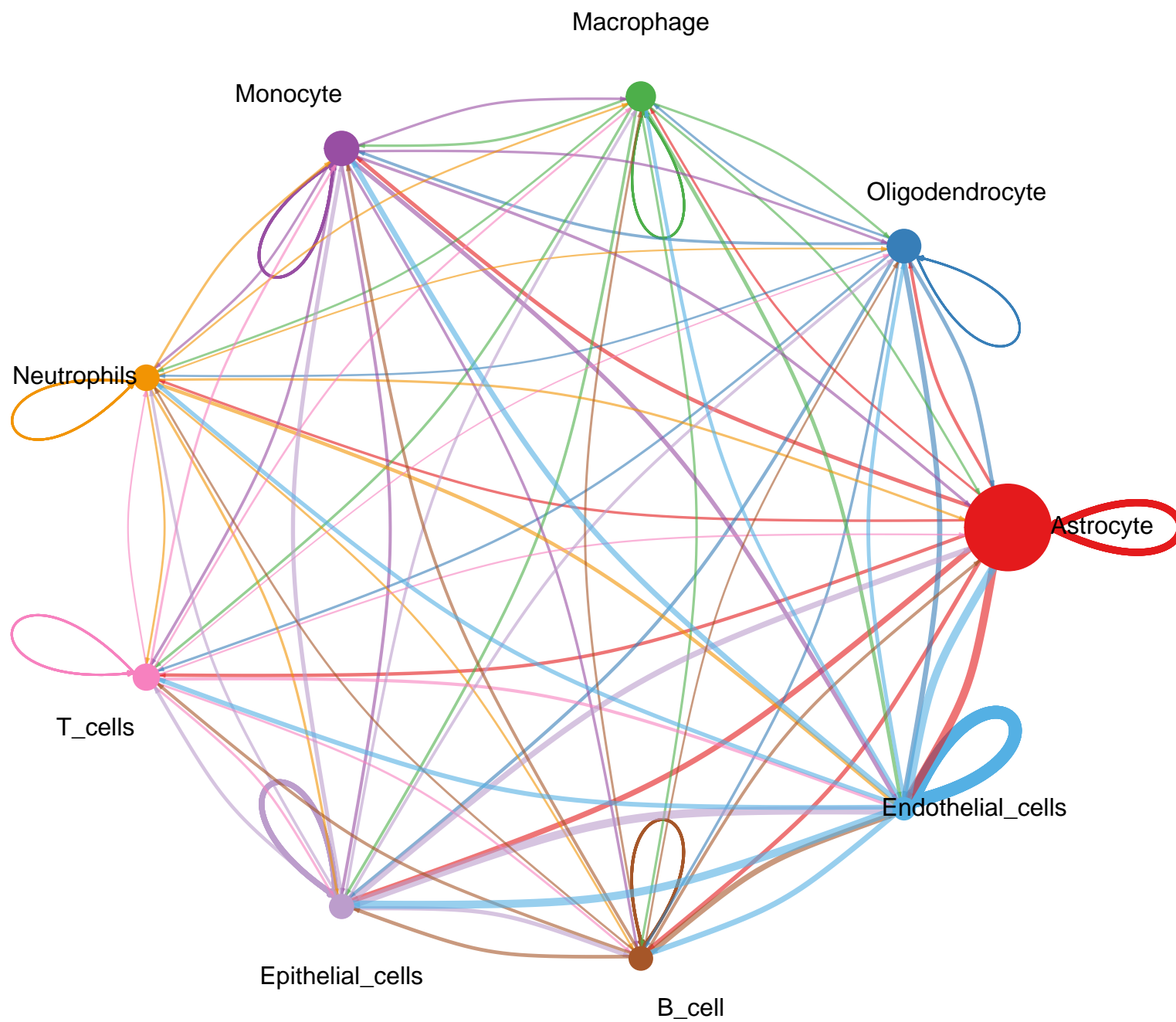

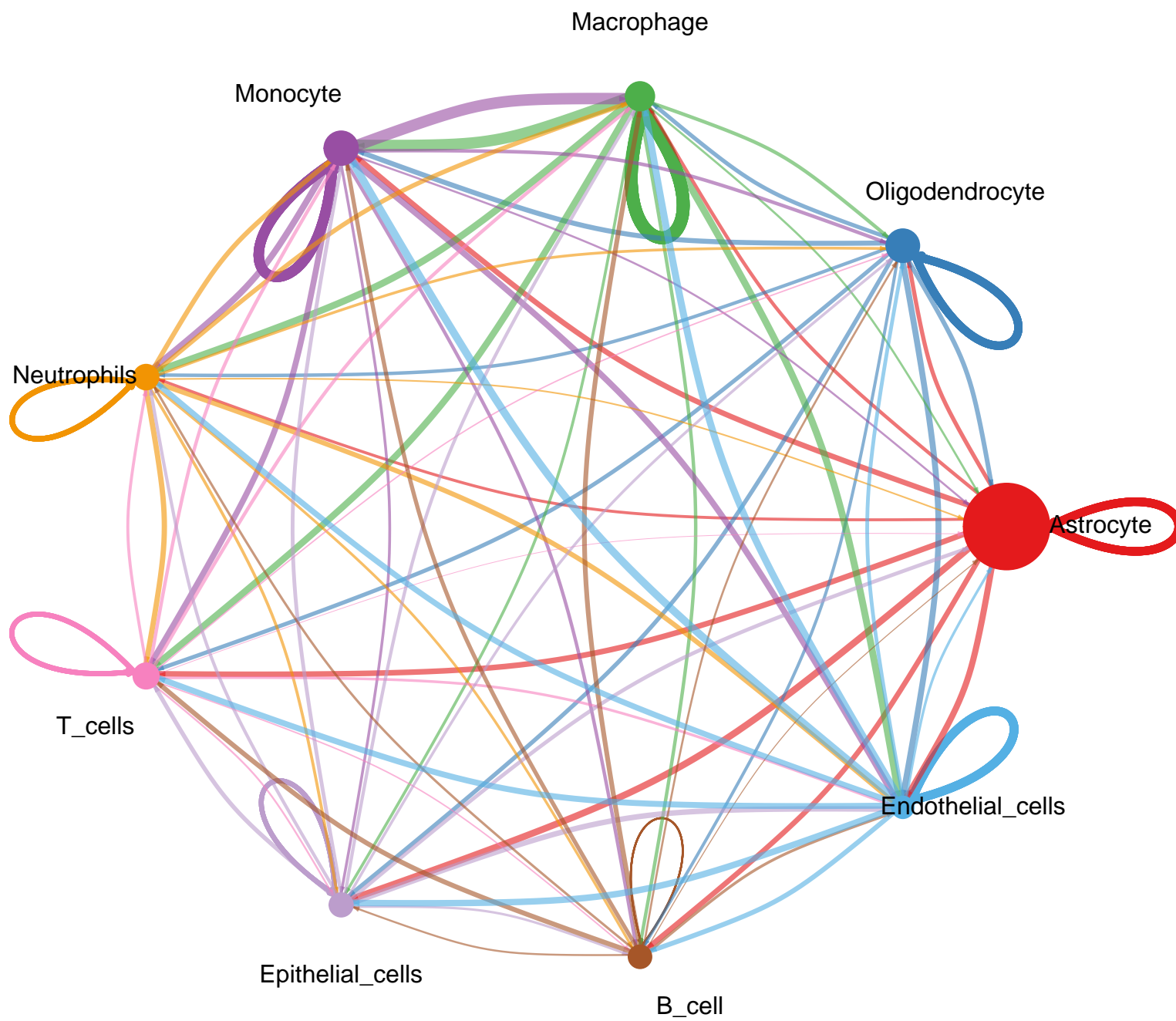

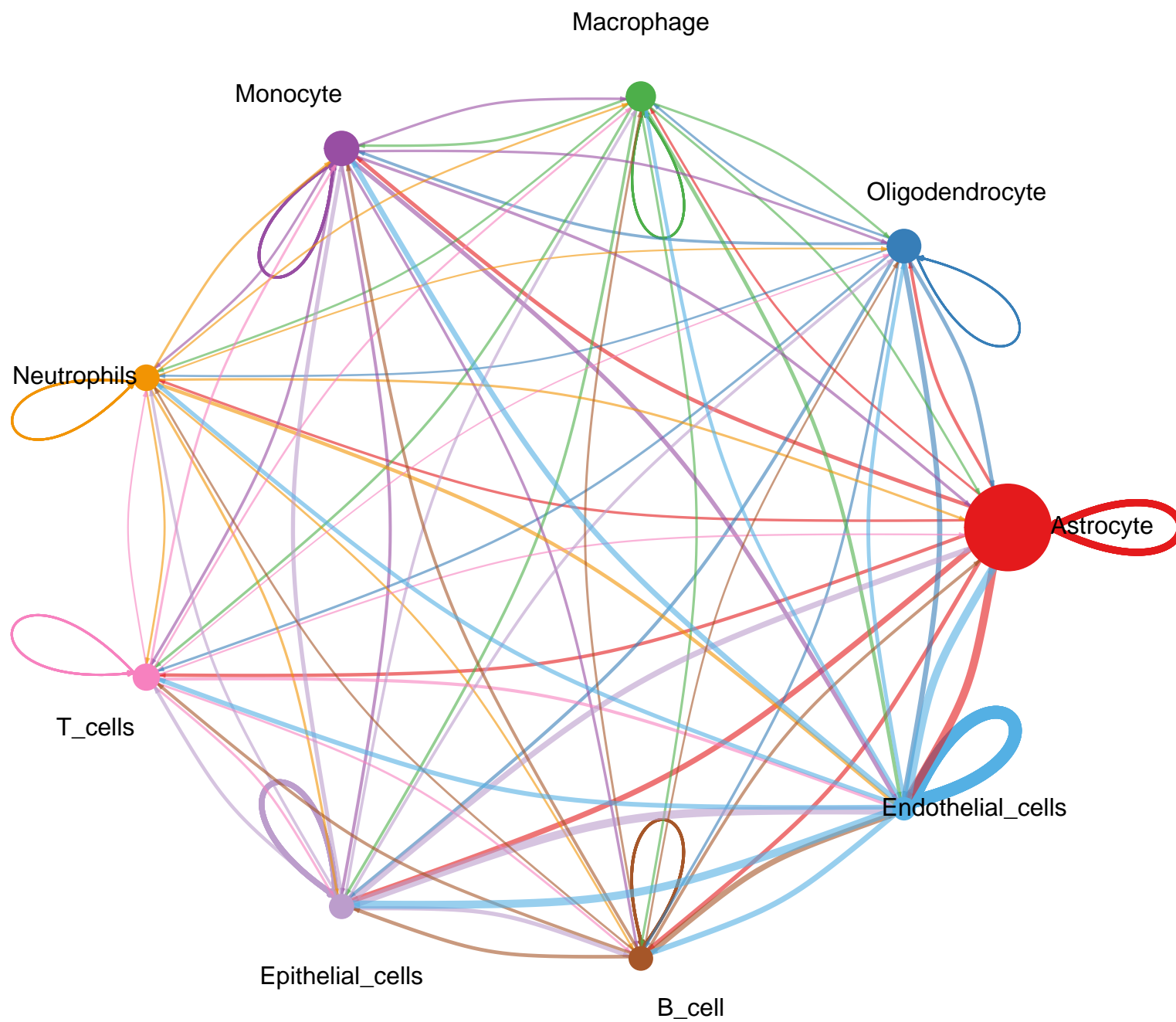

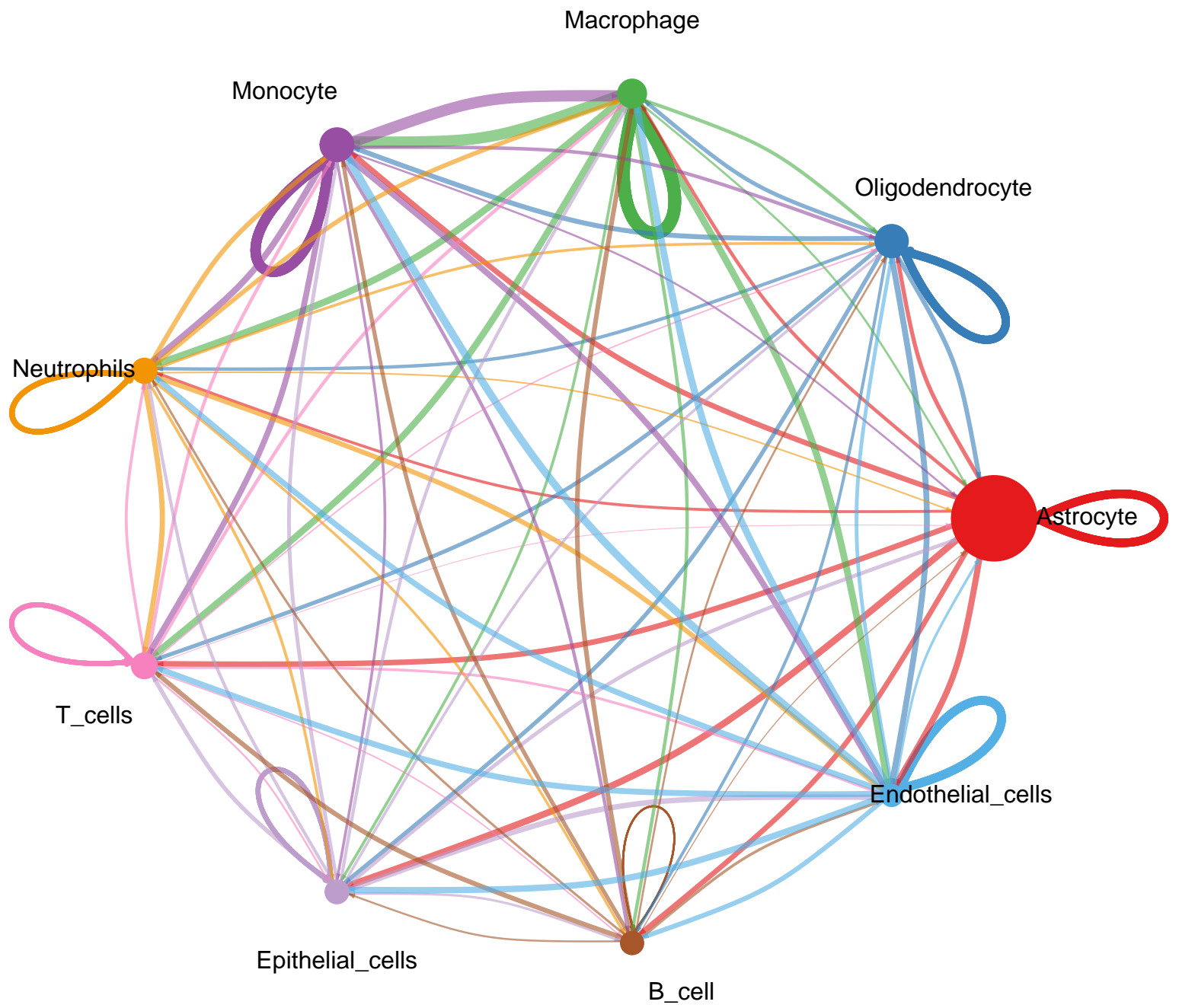

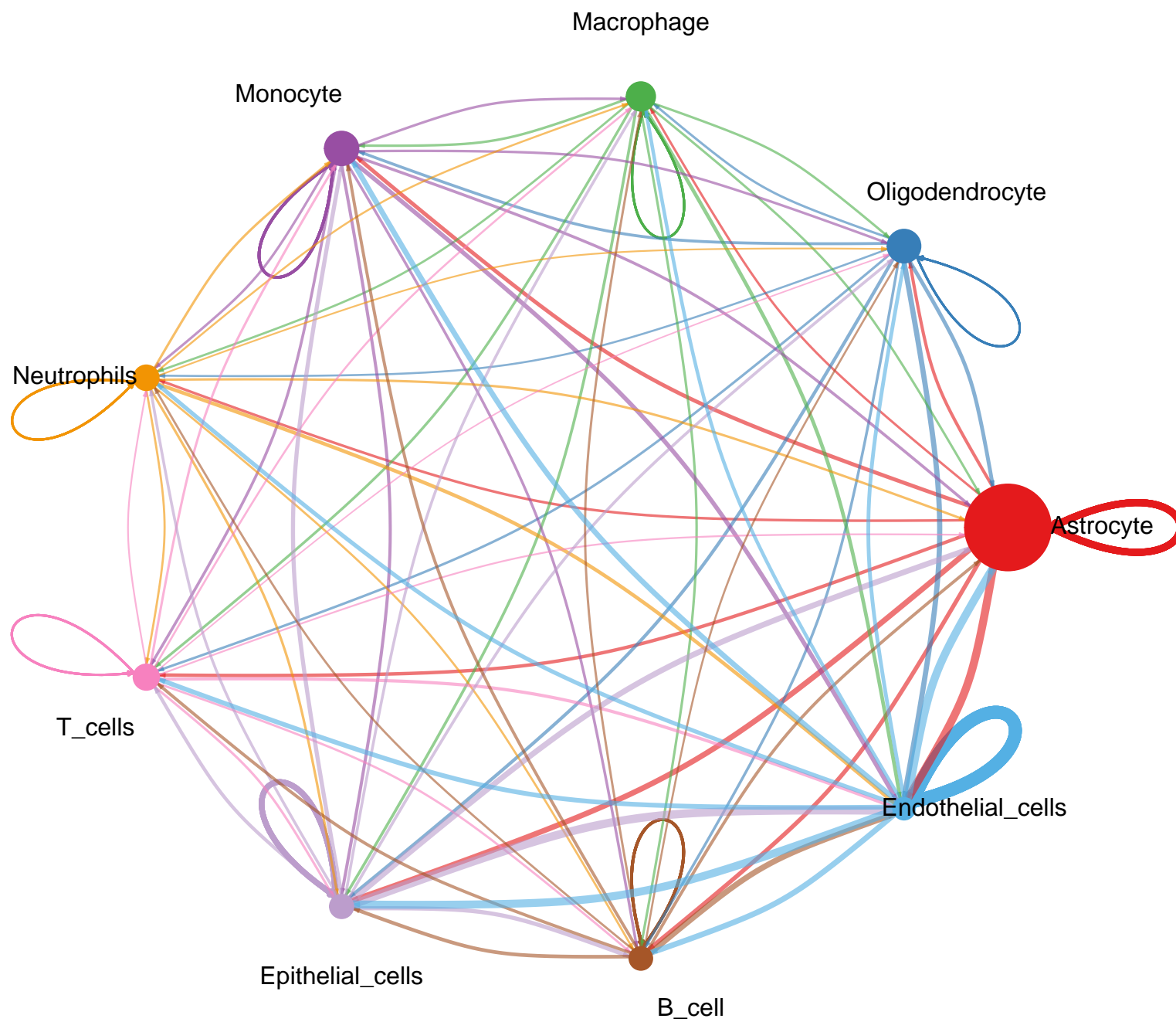

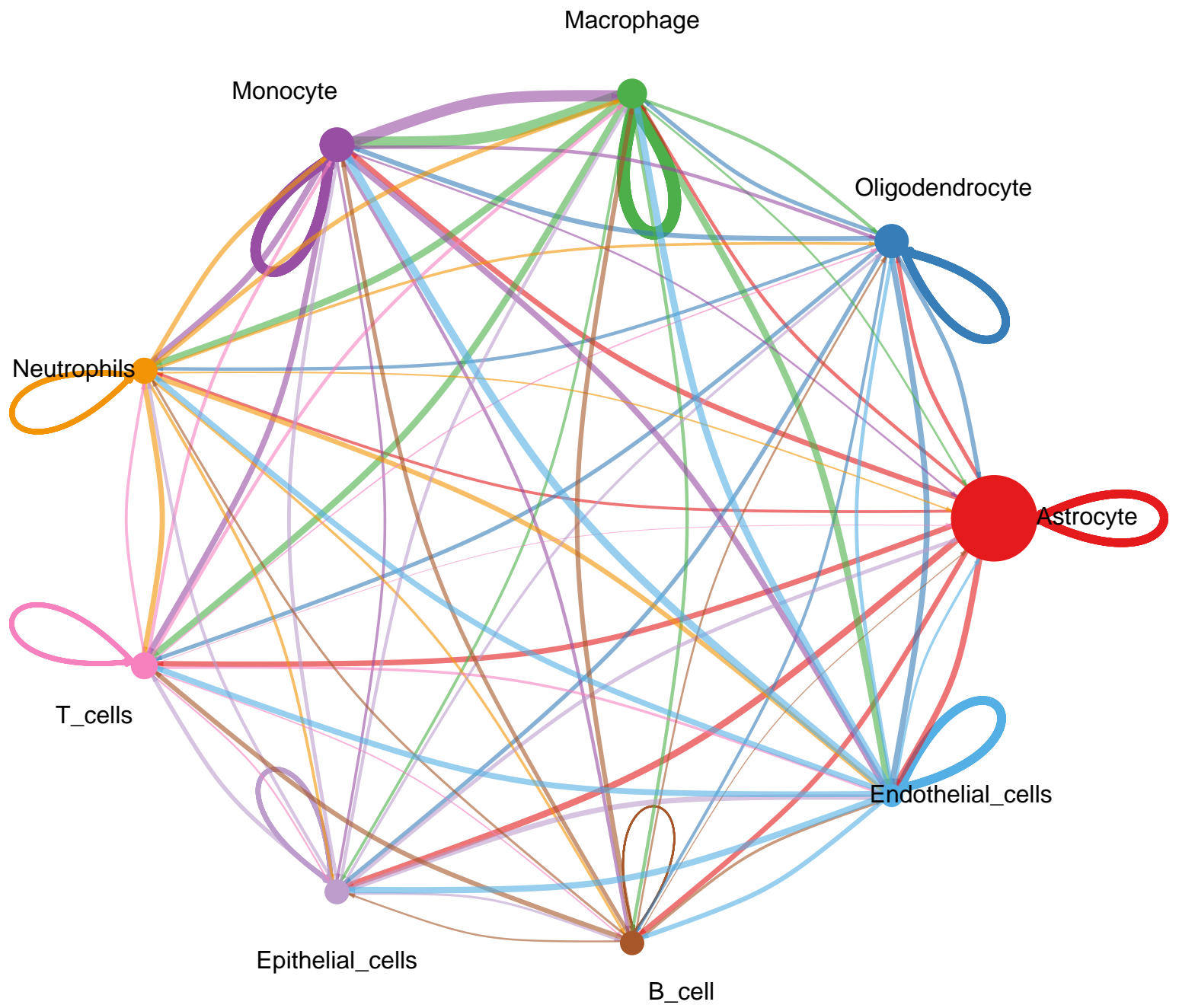

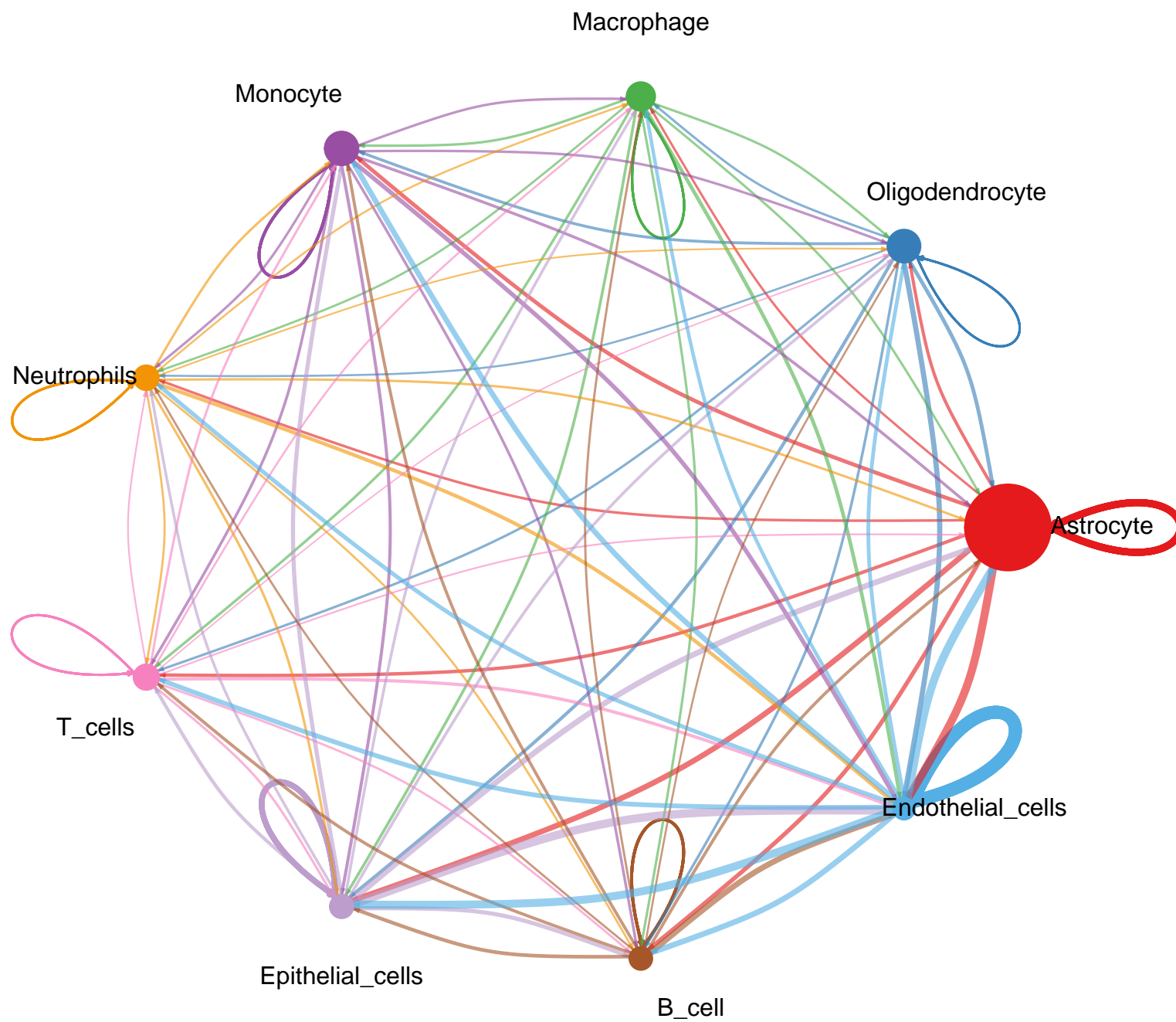

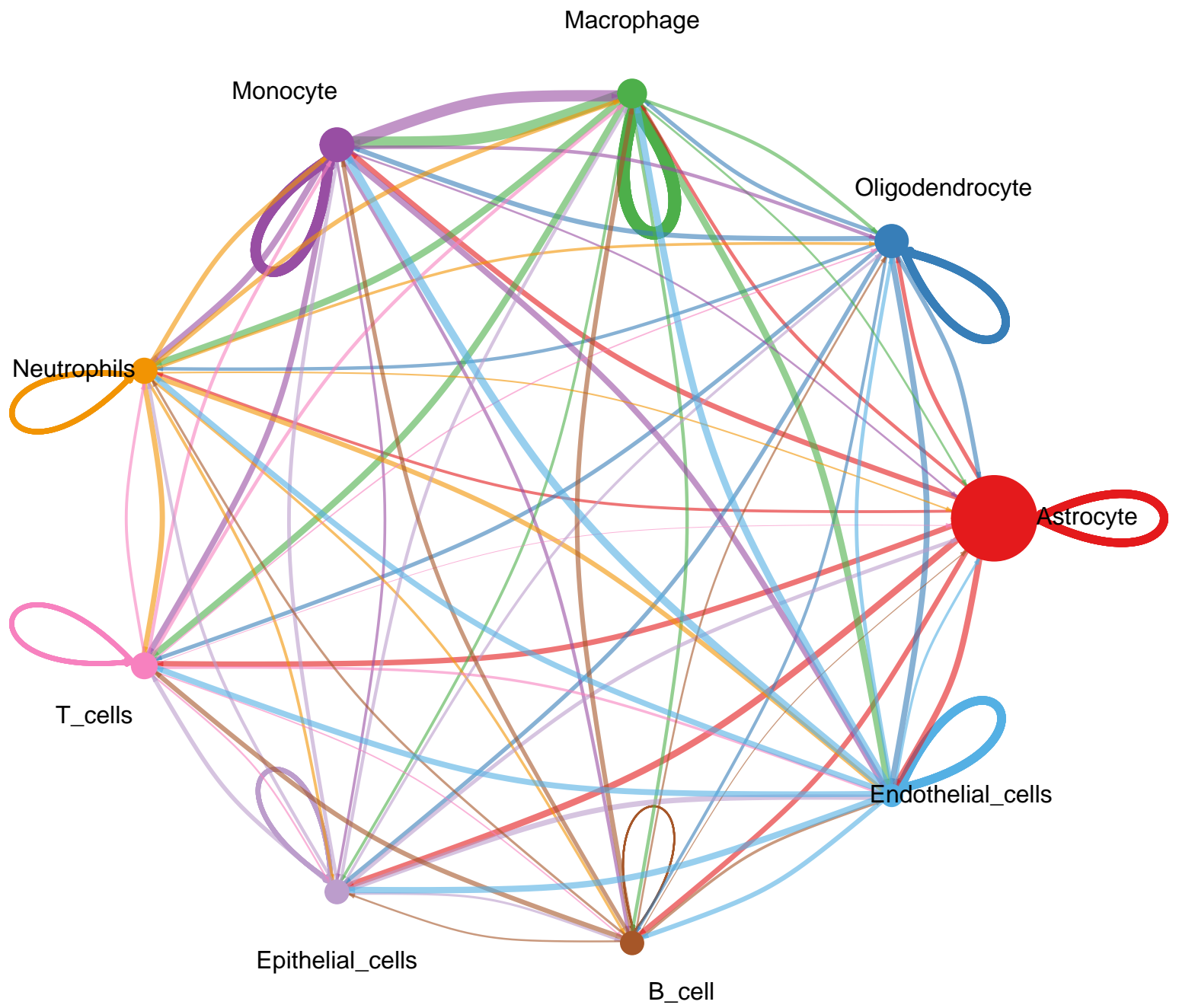

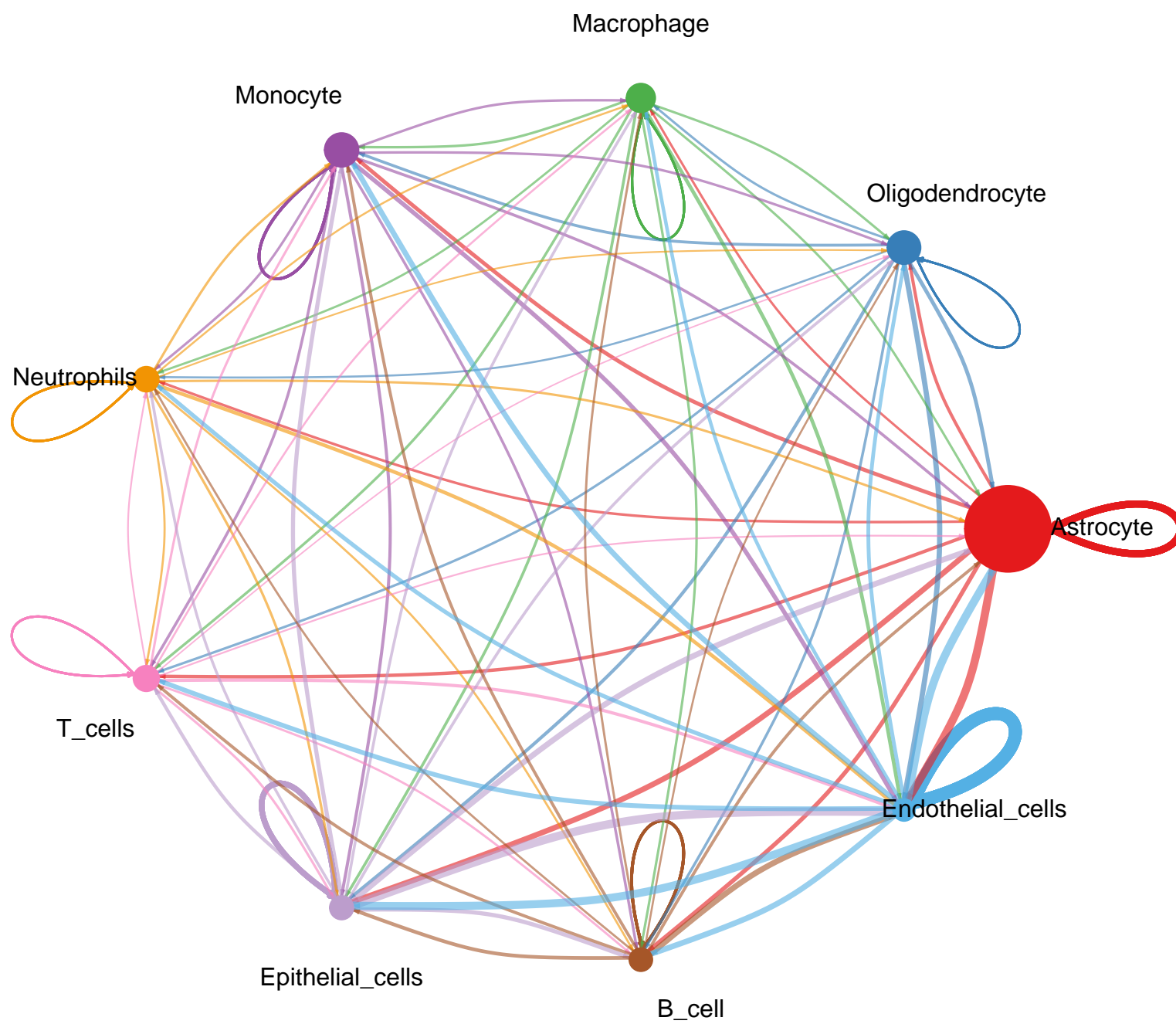

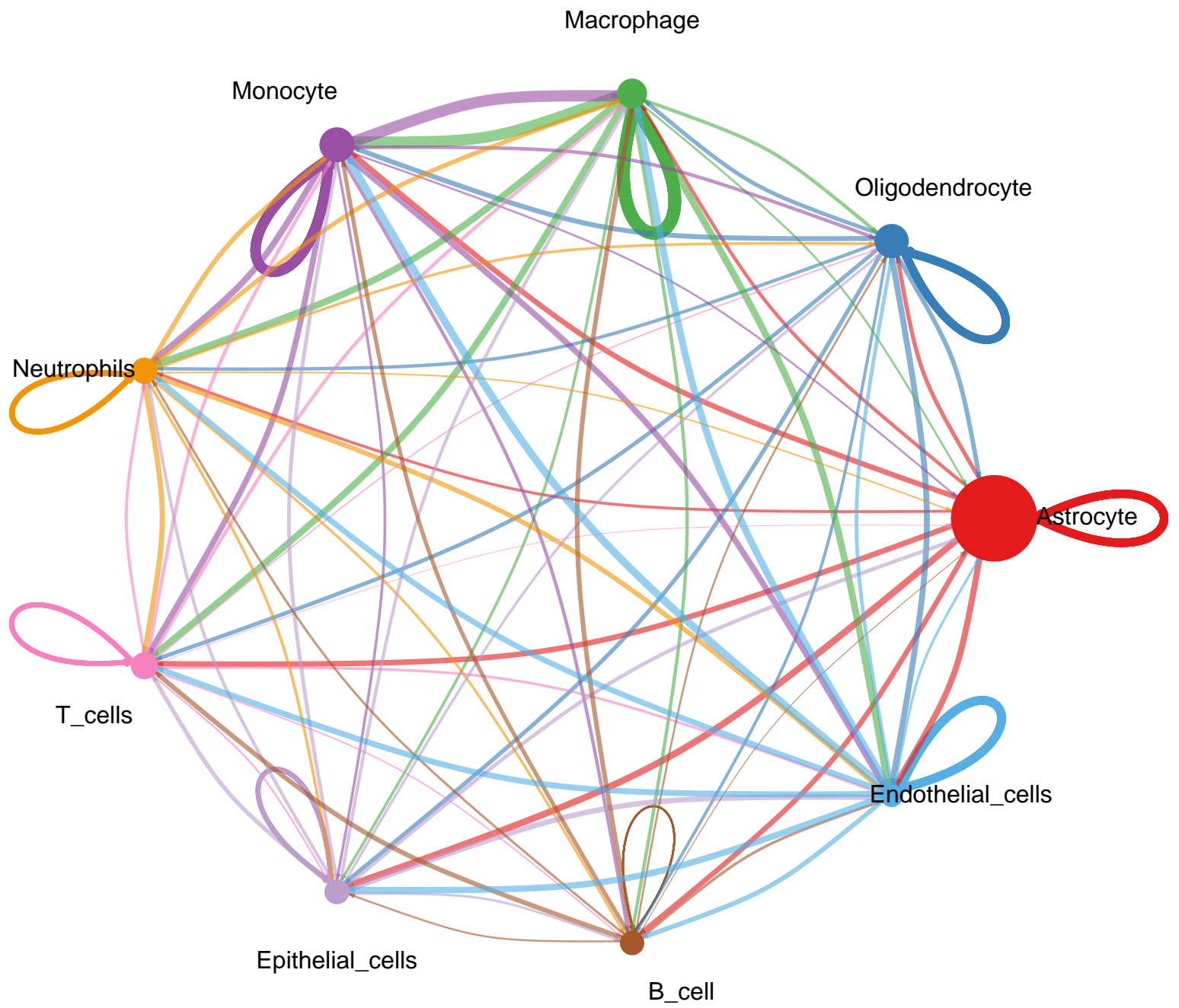

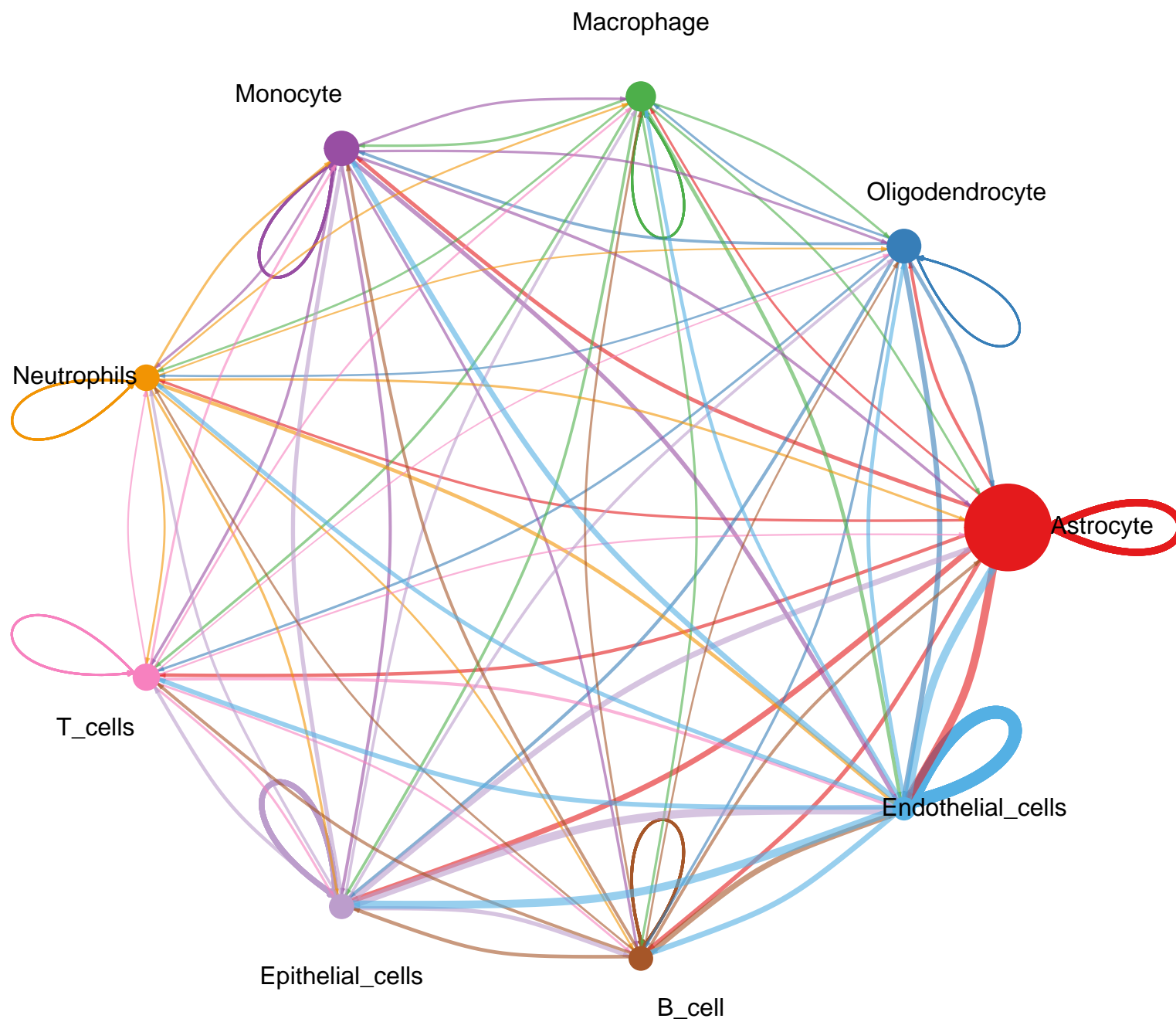

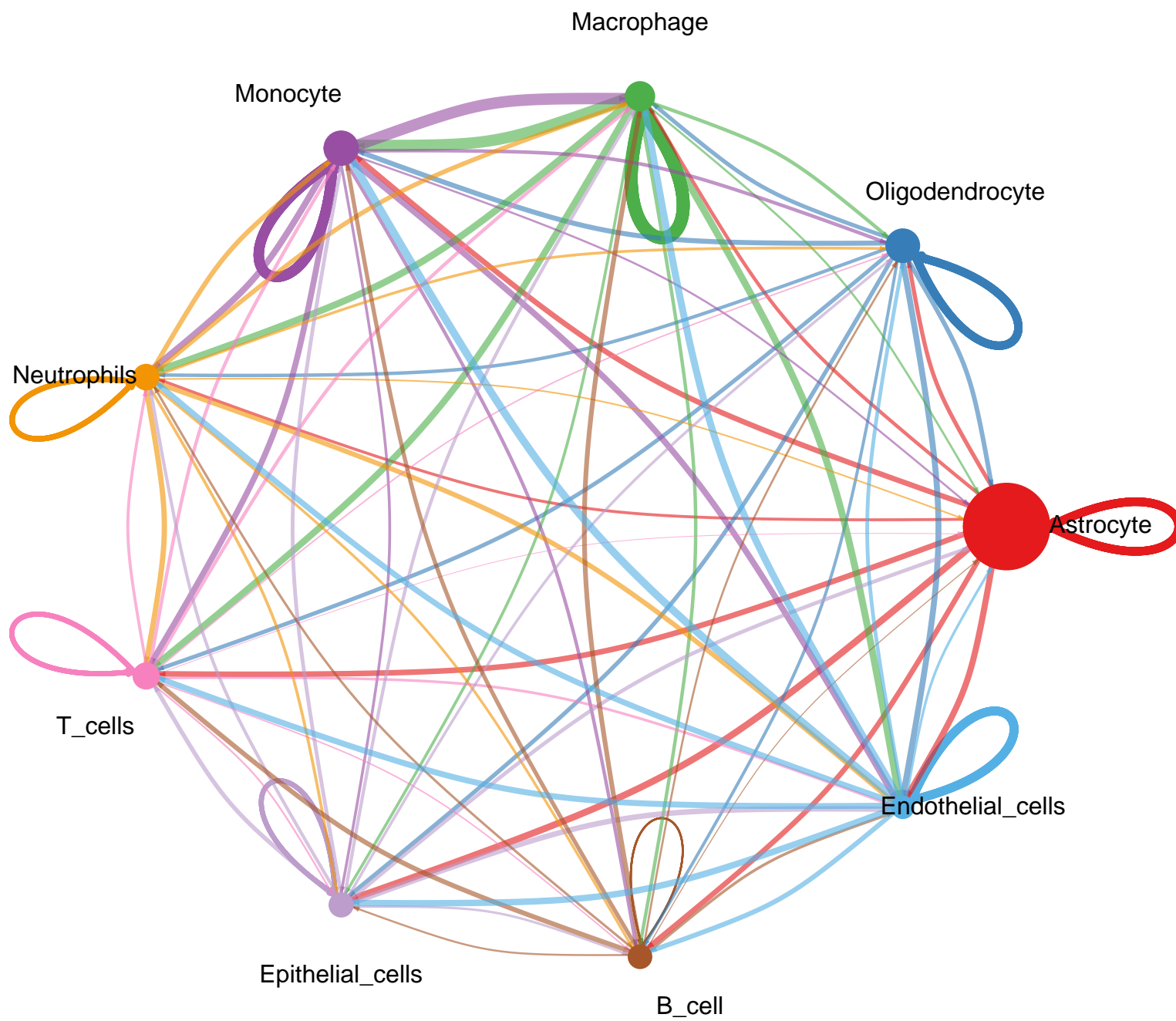

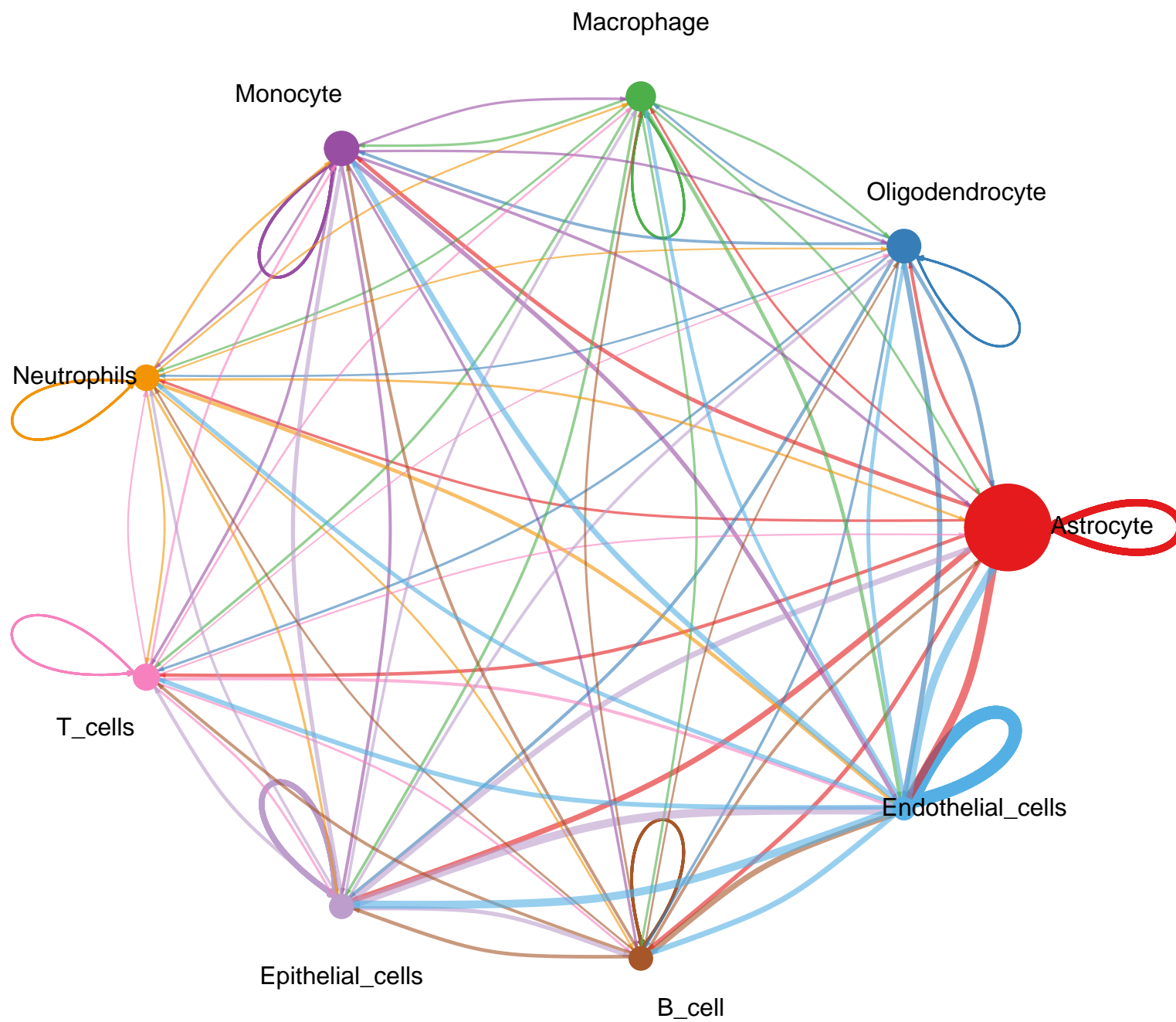

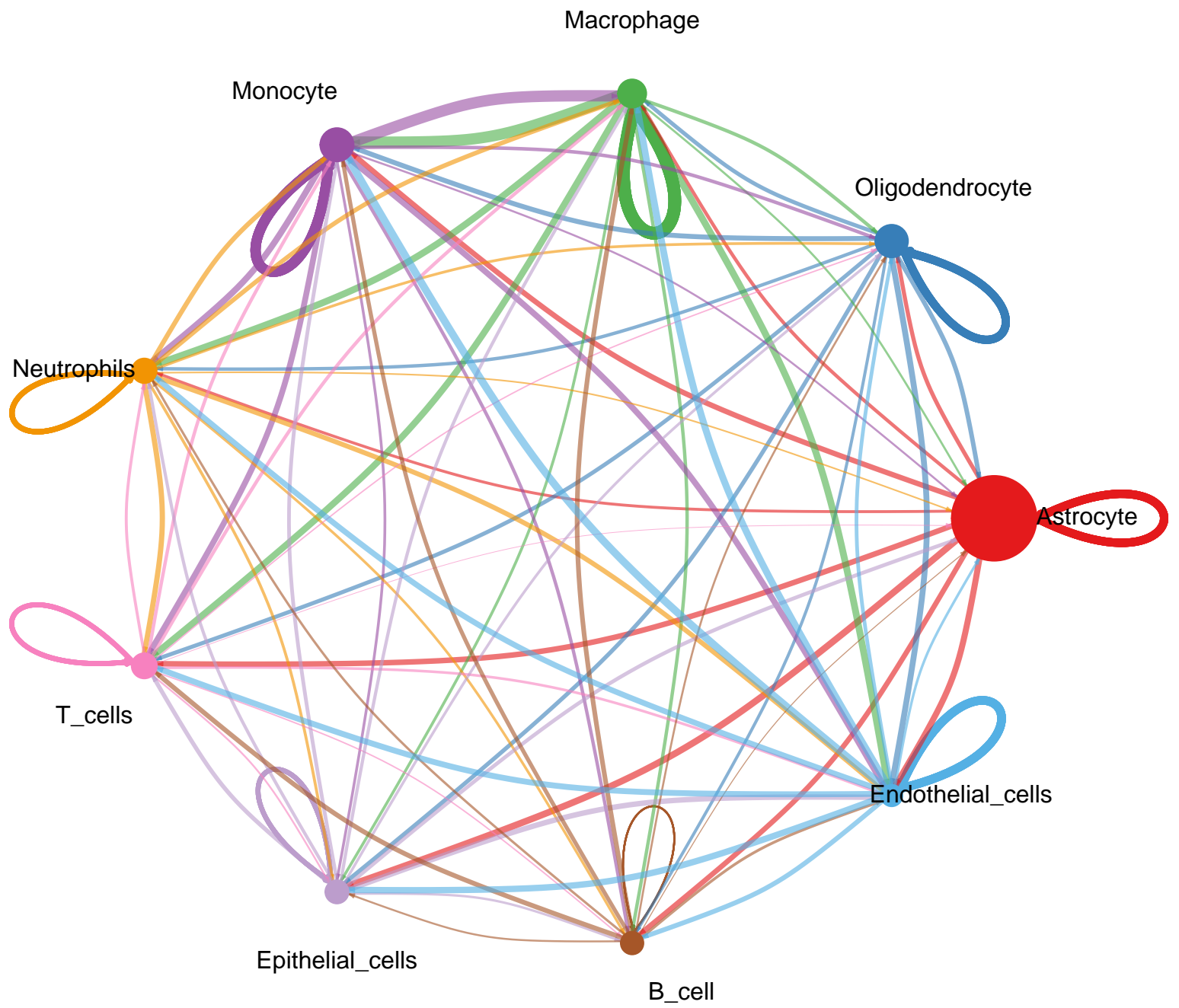

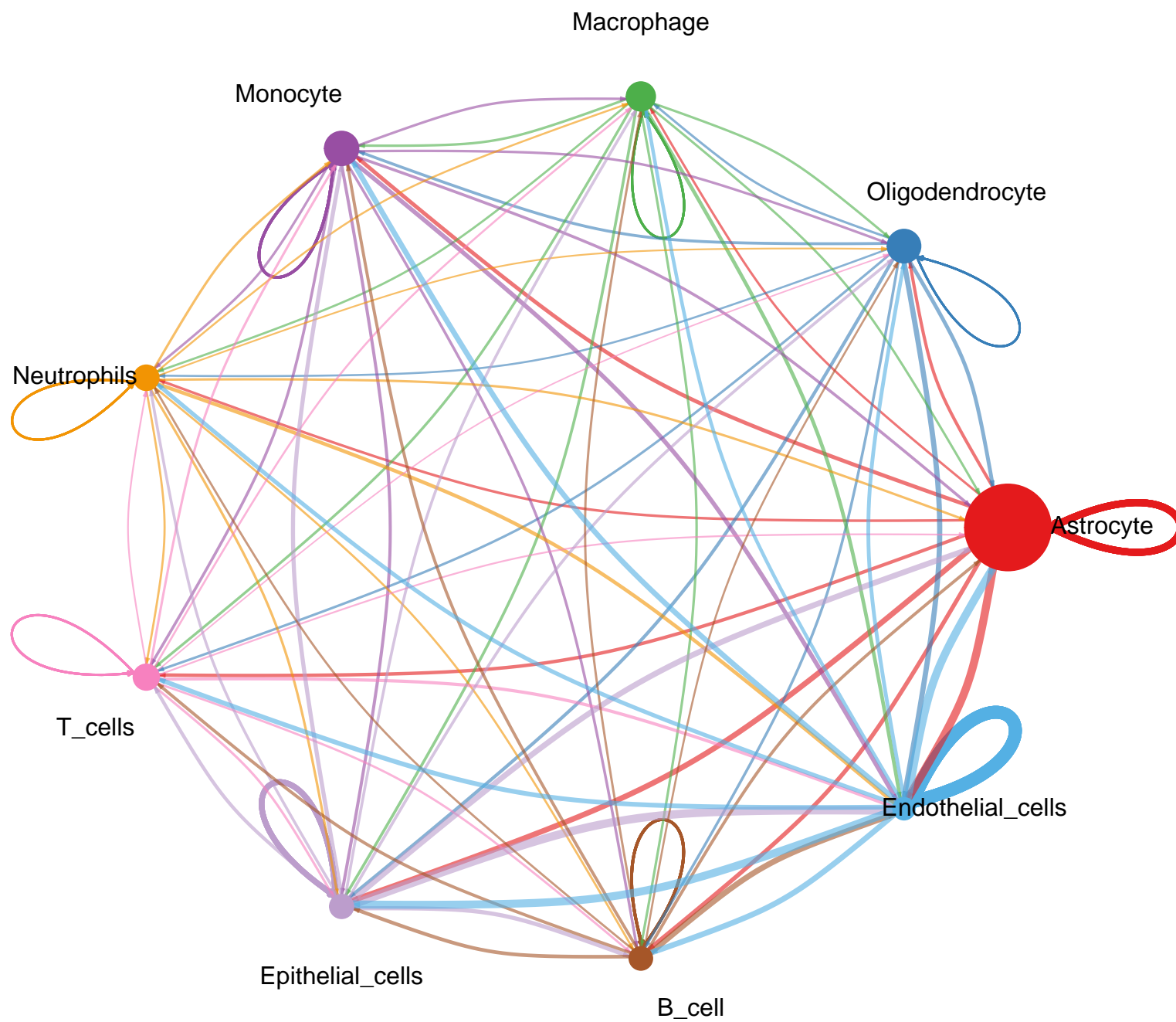

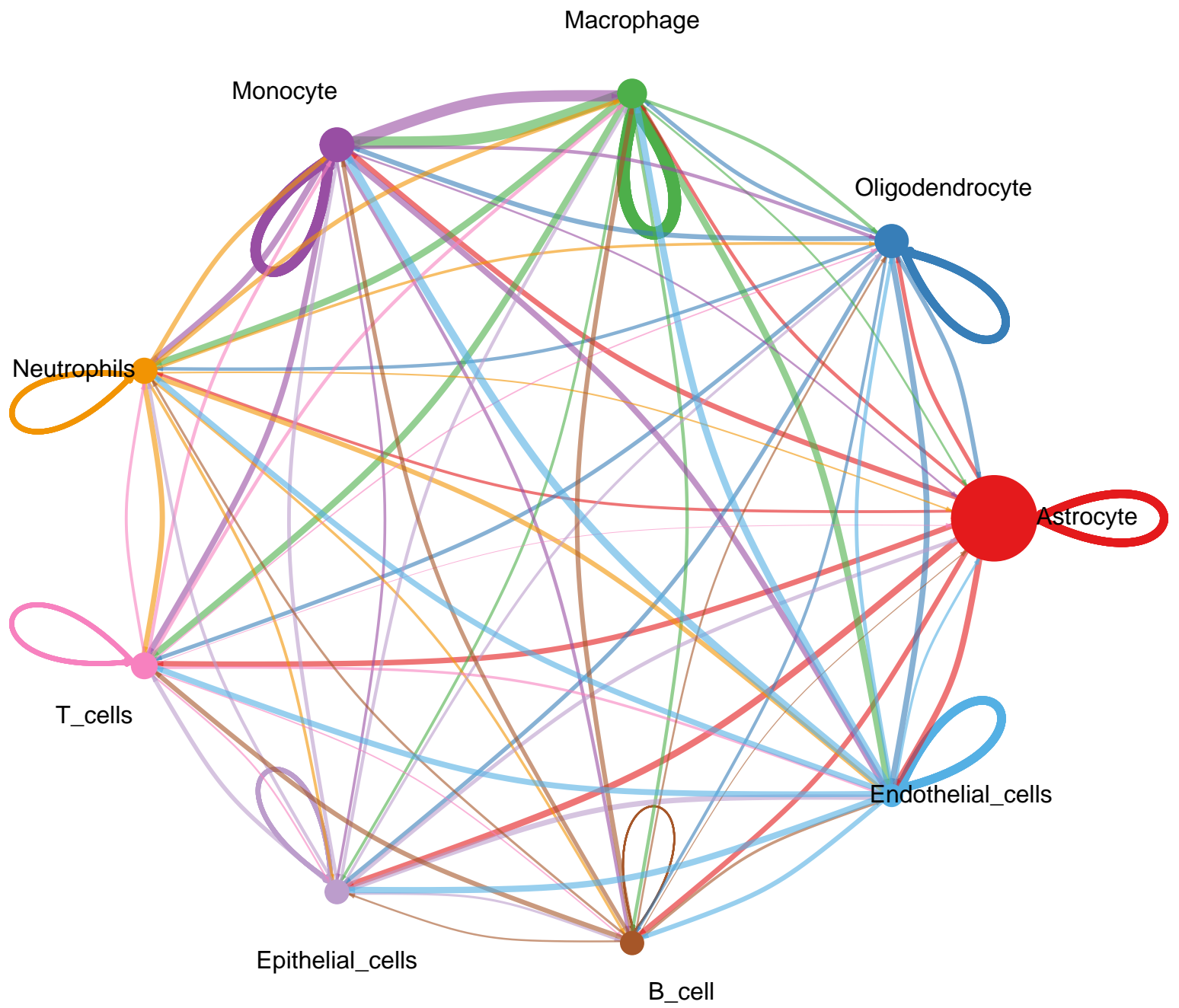

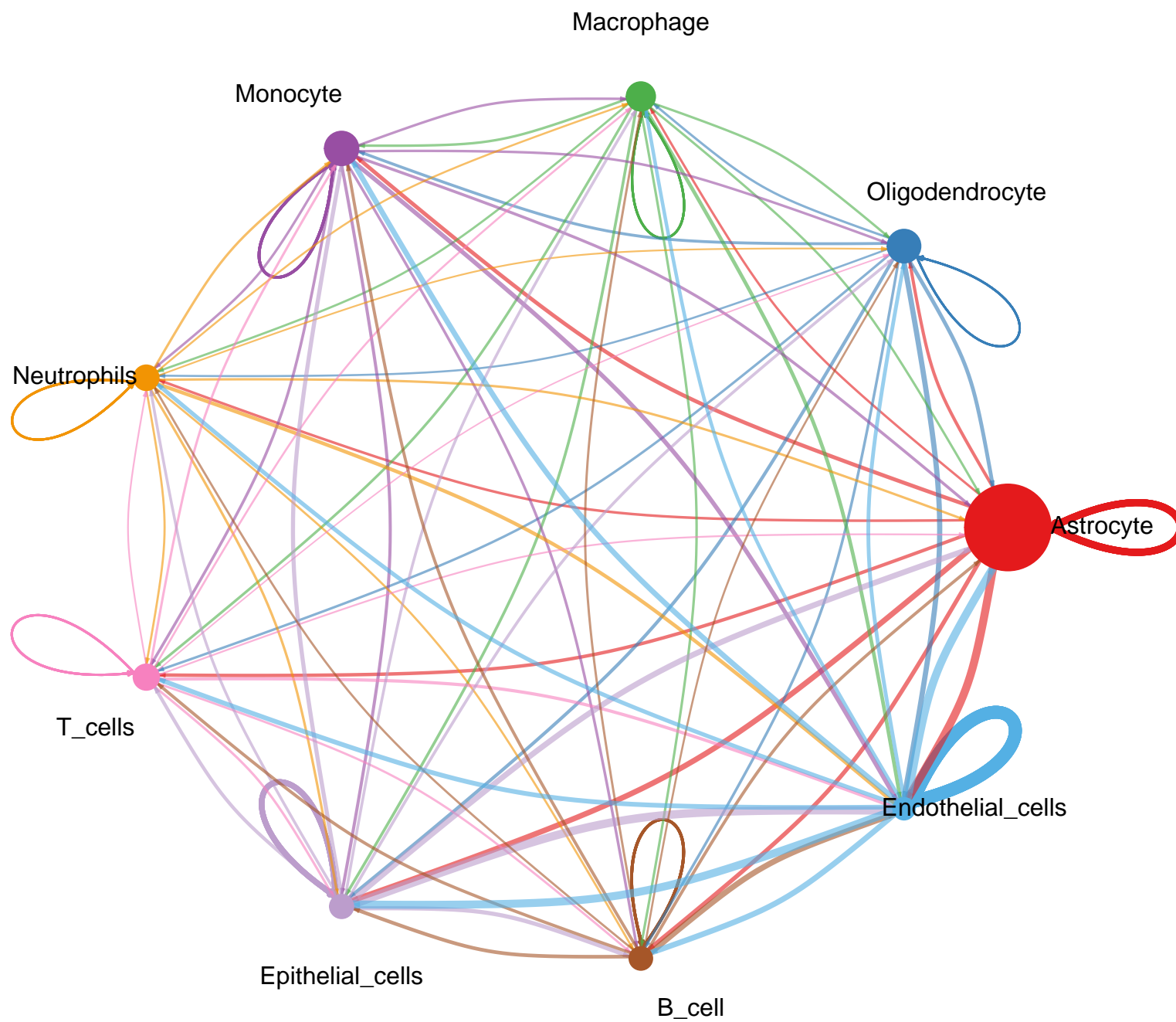

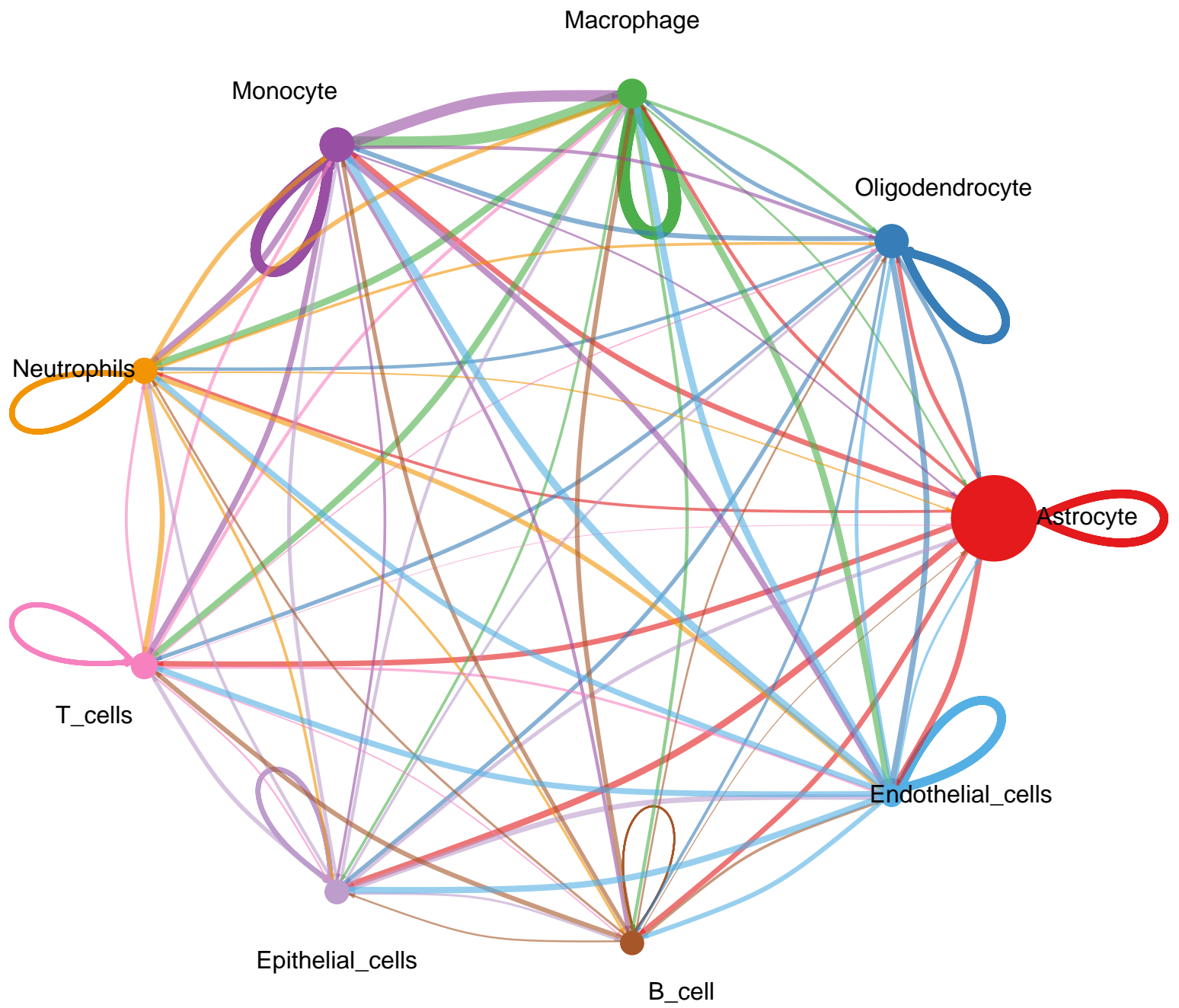

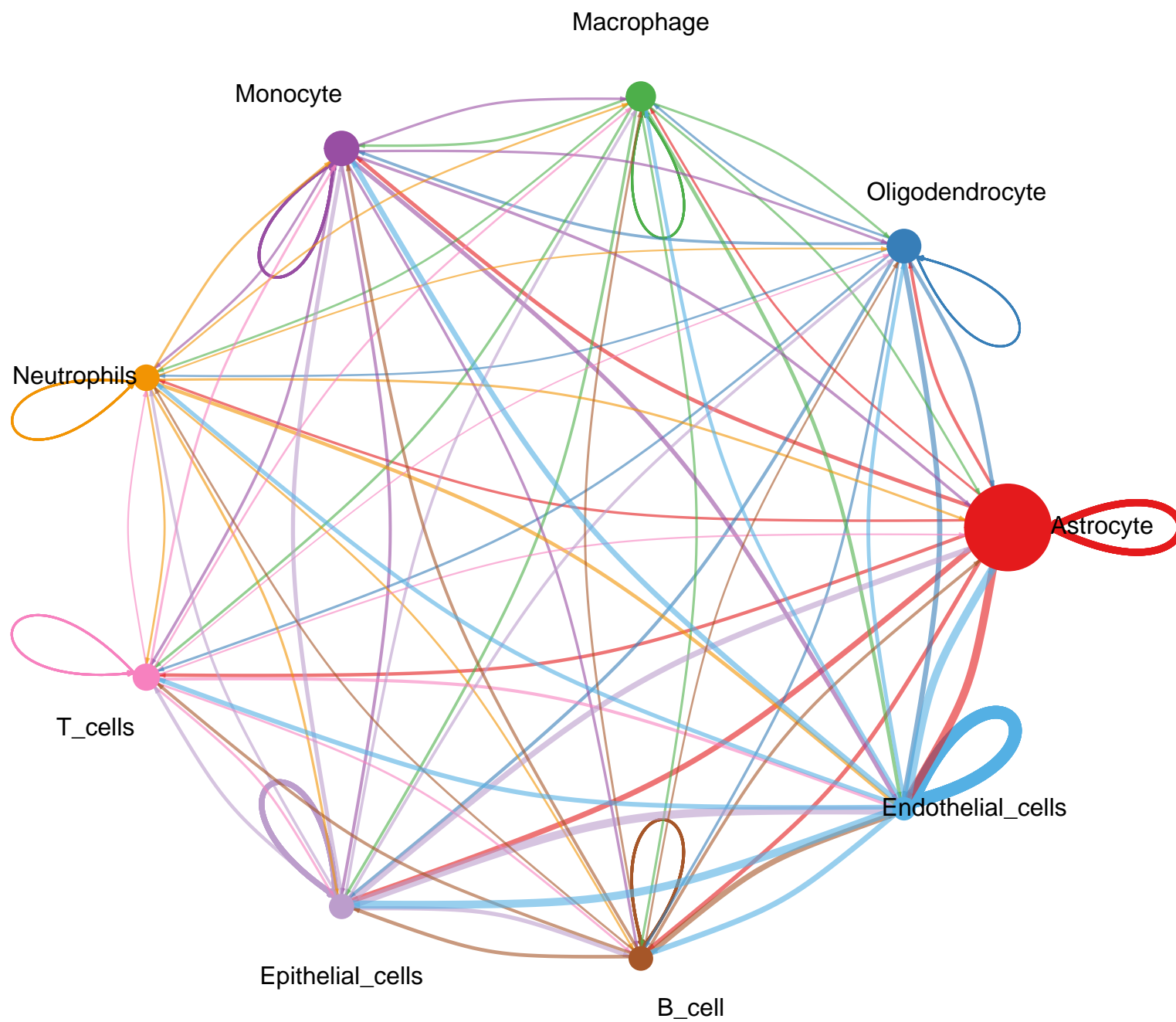

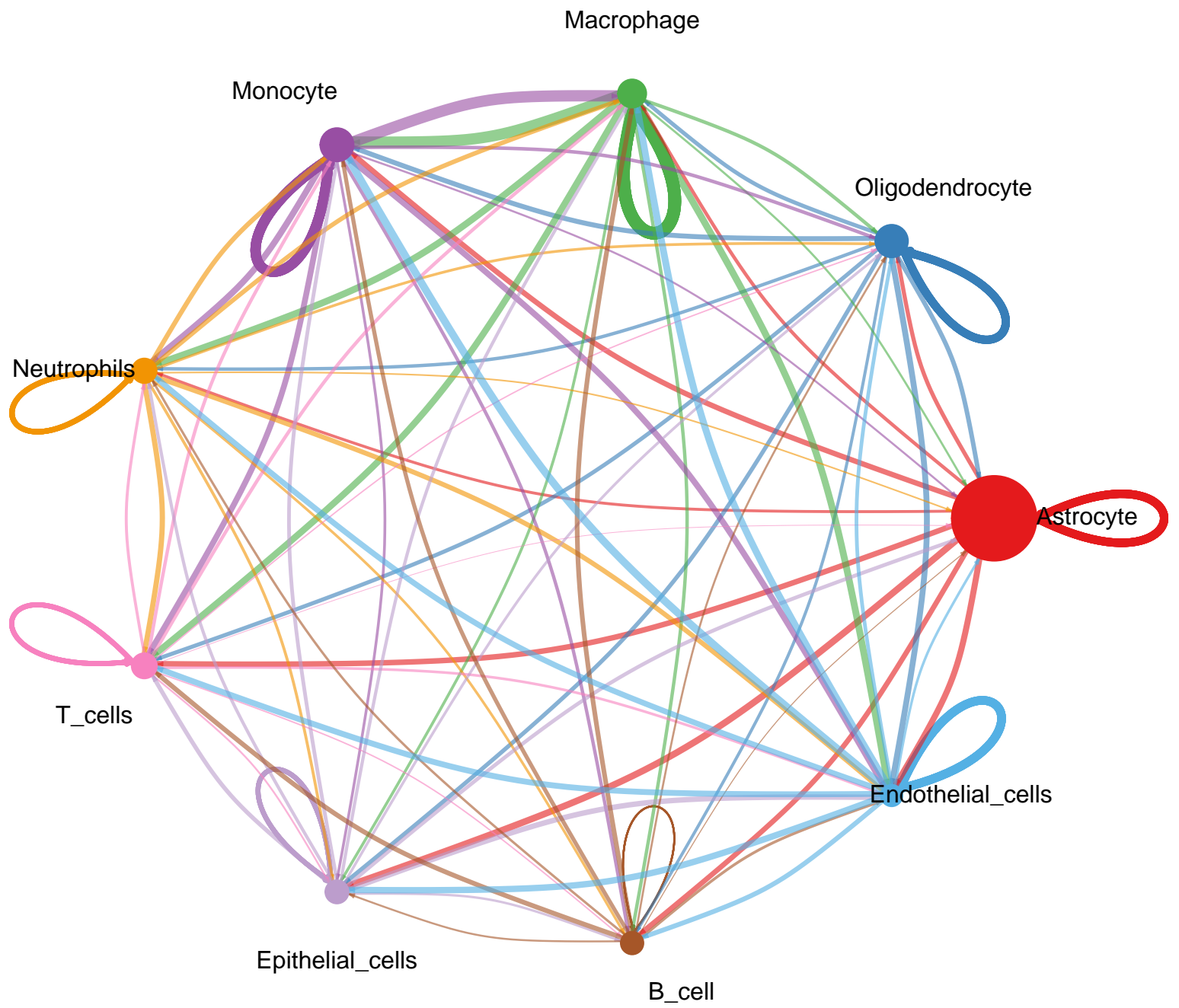

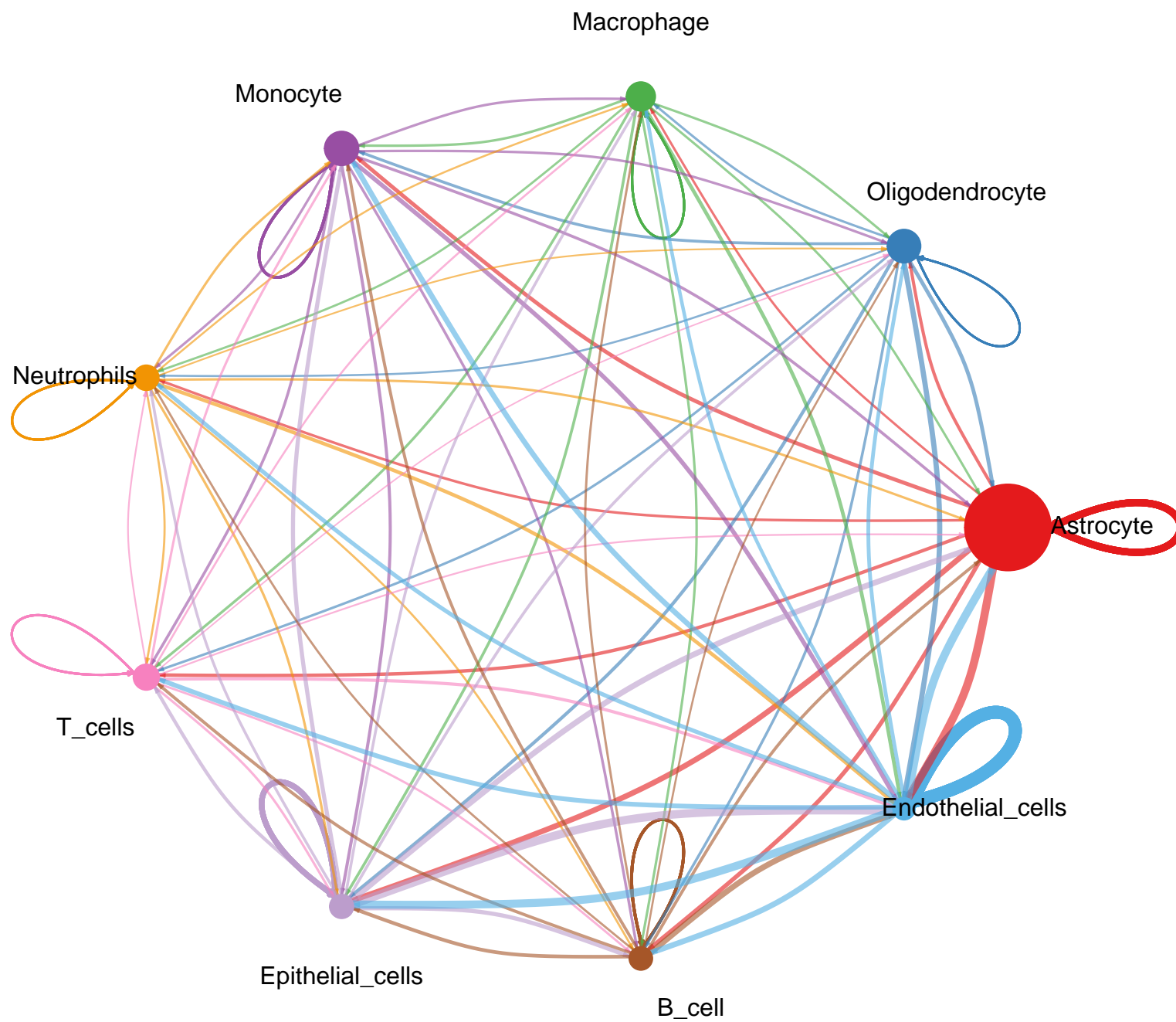

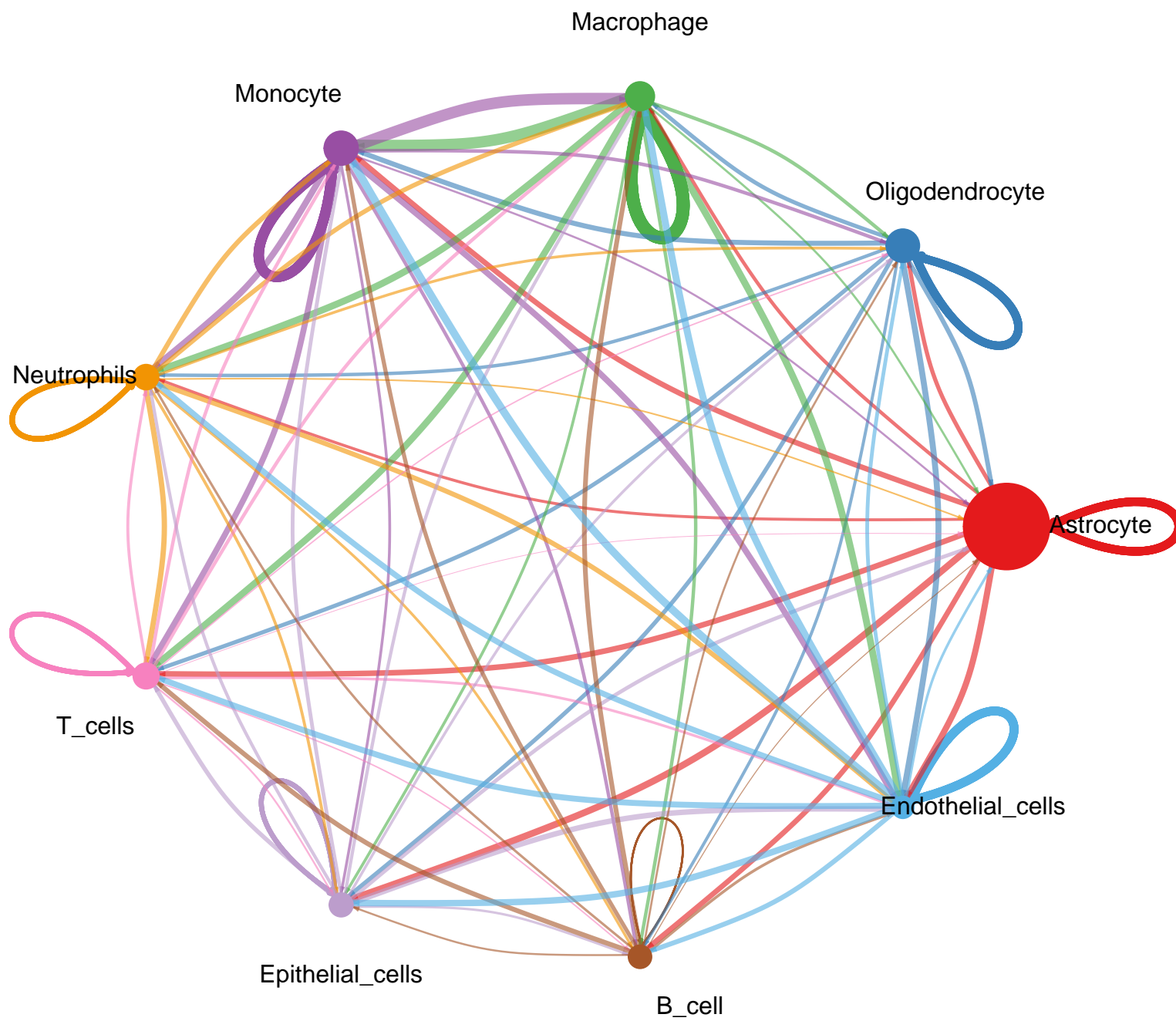

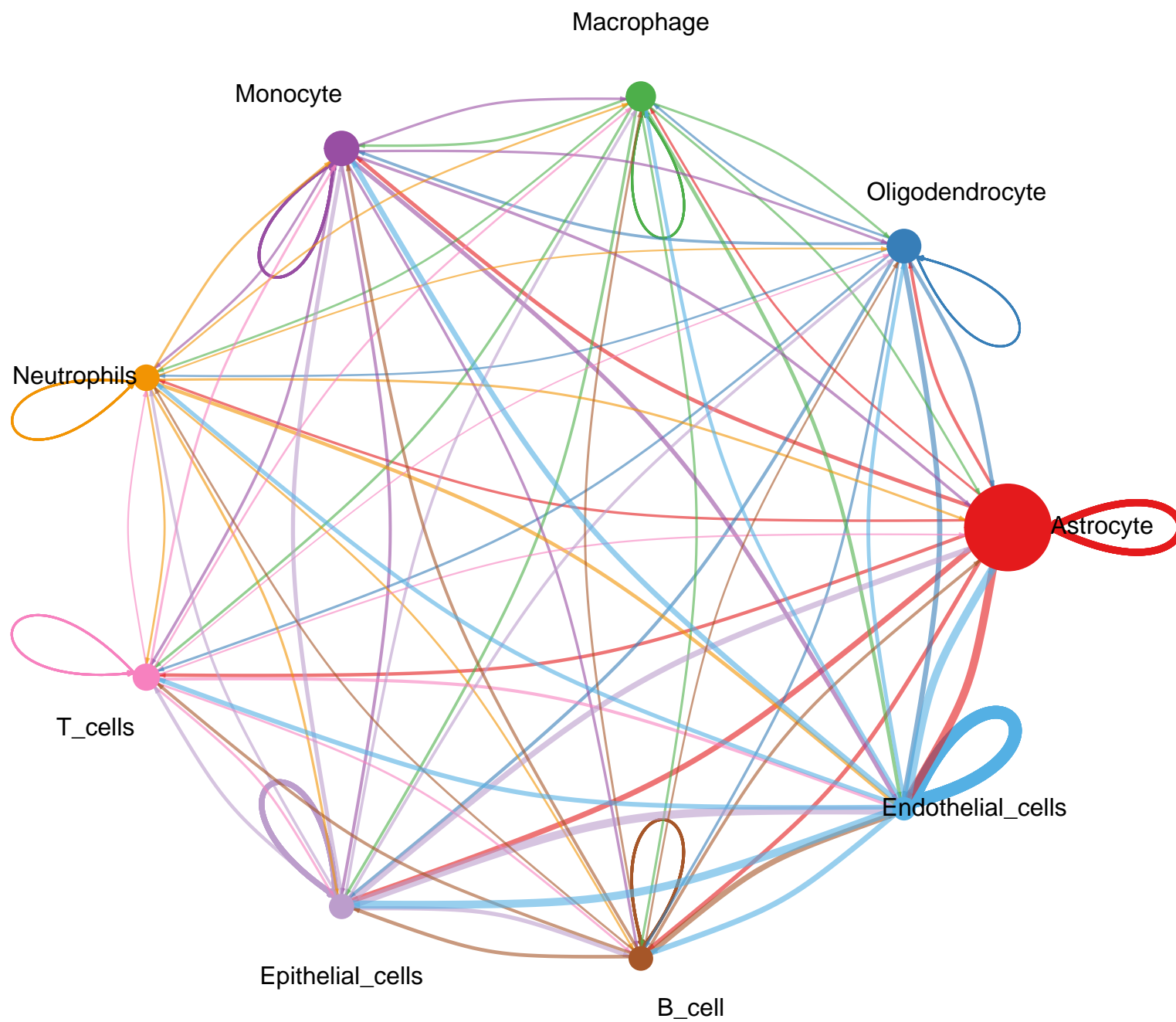

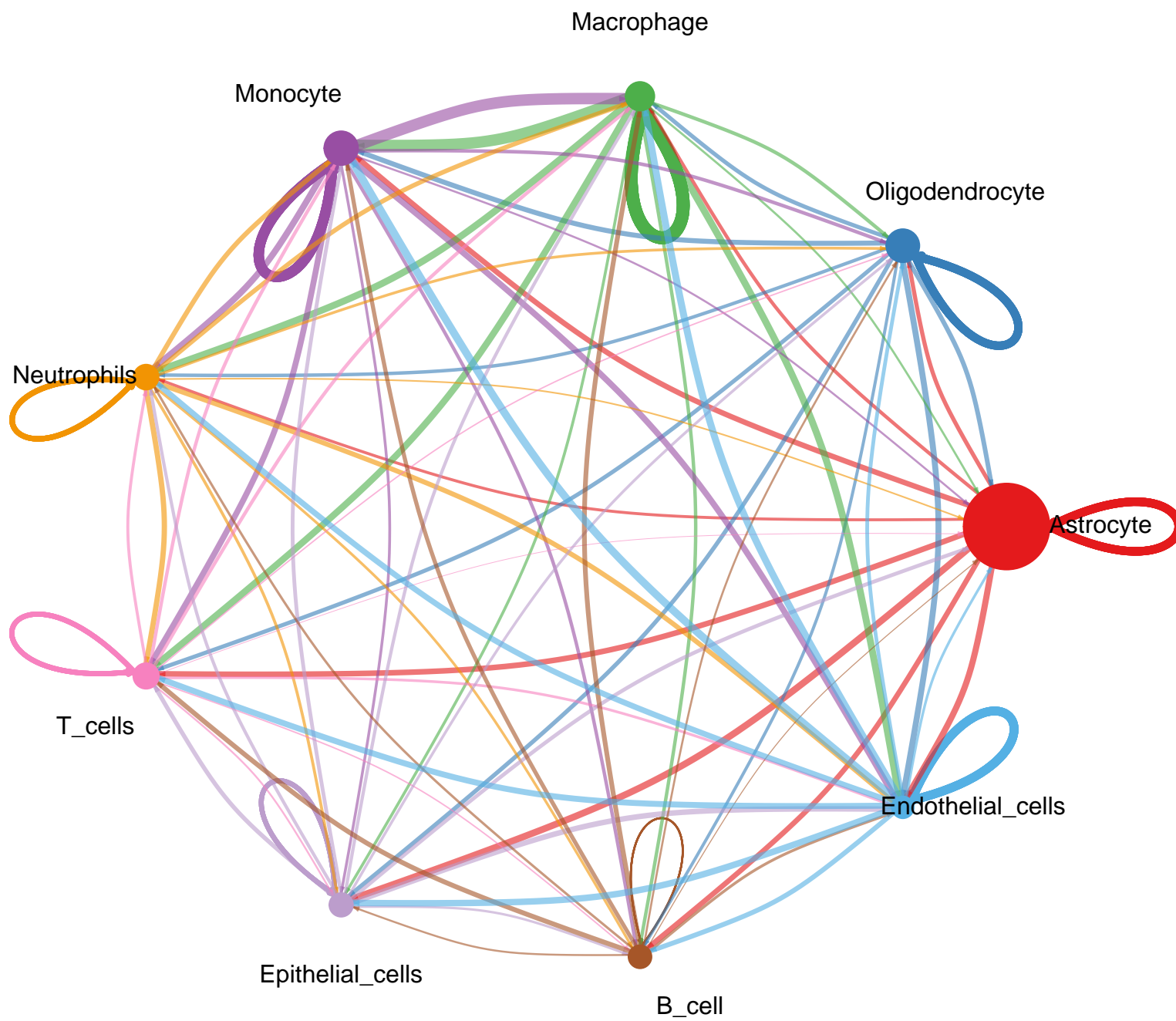

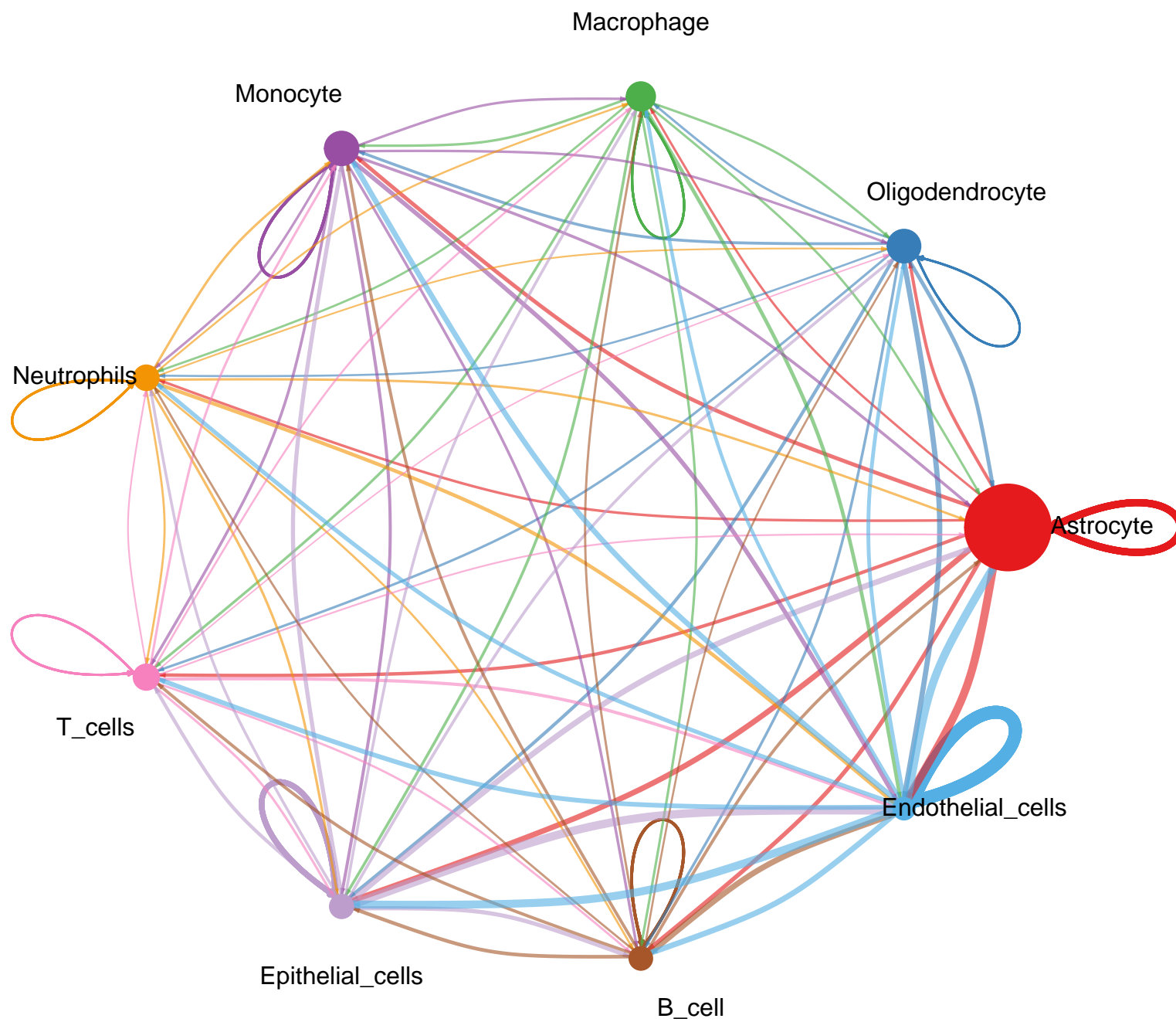

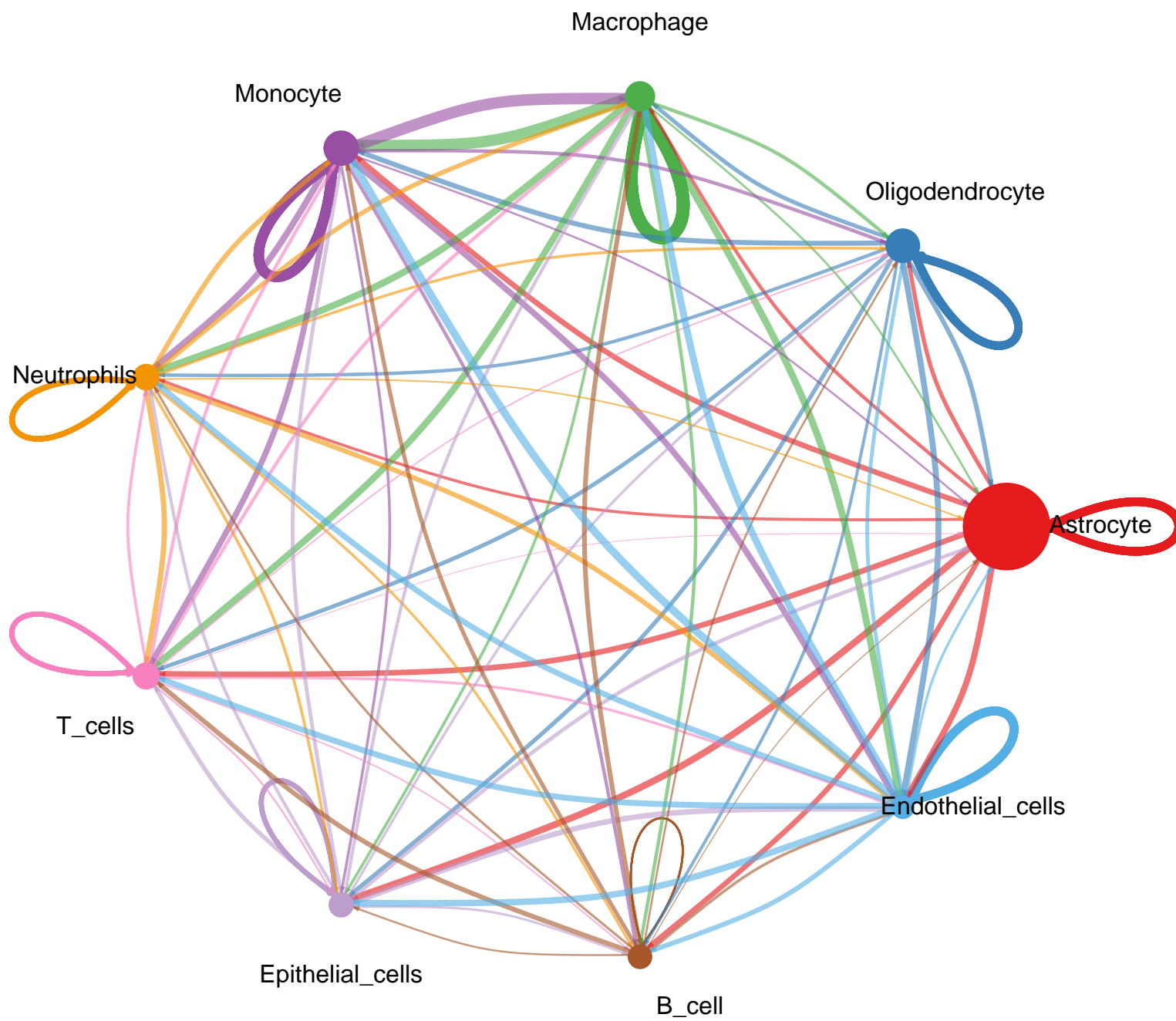

Supplement: Supplementary file 4 [file DataSheet2.pdf]
